# Supplementary material for: RBMS1 orchestrates cardiac hypertrophy by facilitating CTTN splice-switching and sarcomere dynamics
Source: EMBO Mol Med. 2025 Nov 10;17(12):3555–85. doi: 10.1038/s44321-025-00334-z (PMC12686484; doi:10.1038/s44321-025-00334-z)
Supplement: Supplementary file 1 — Appendix [file 44321_2025_334_MOESM1_ESM.docx]

**Appendix for**

**RBMS1 Orchestrates Cardiac Hypertrophy by Facilitating CTTN Splice-switching and Sarcomere Dynamics**

**Table of contents**

[Appendix Figure S1](#_Toc103954329) 2

[Appendix Figure S2](#_Toc103954329) 3

[Appendix Figure S3](#_Toc103954329) 5

[Appendix Figure S4](#_Toc103954329) 7

[Appendix Figure S5](#_Toc103954329) 8

[Appendix Figure S6 10](#_Toc103954329)

[Appendix Figure S7 1](#_Toc103954329)2

[Appendix Figure S8 1](#_Toc103954329)3

[Appendix Figure S9](#_Toc103954329) 15

[Appendix Figure S10](#_Toc103954329) 17

[Appendix Figure S11](#_Toc103954329) 18

[Appendix Table S1](#_Toc103954329) 20

[Appendix Table S2 2](#_Toc103954329)1


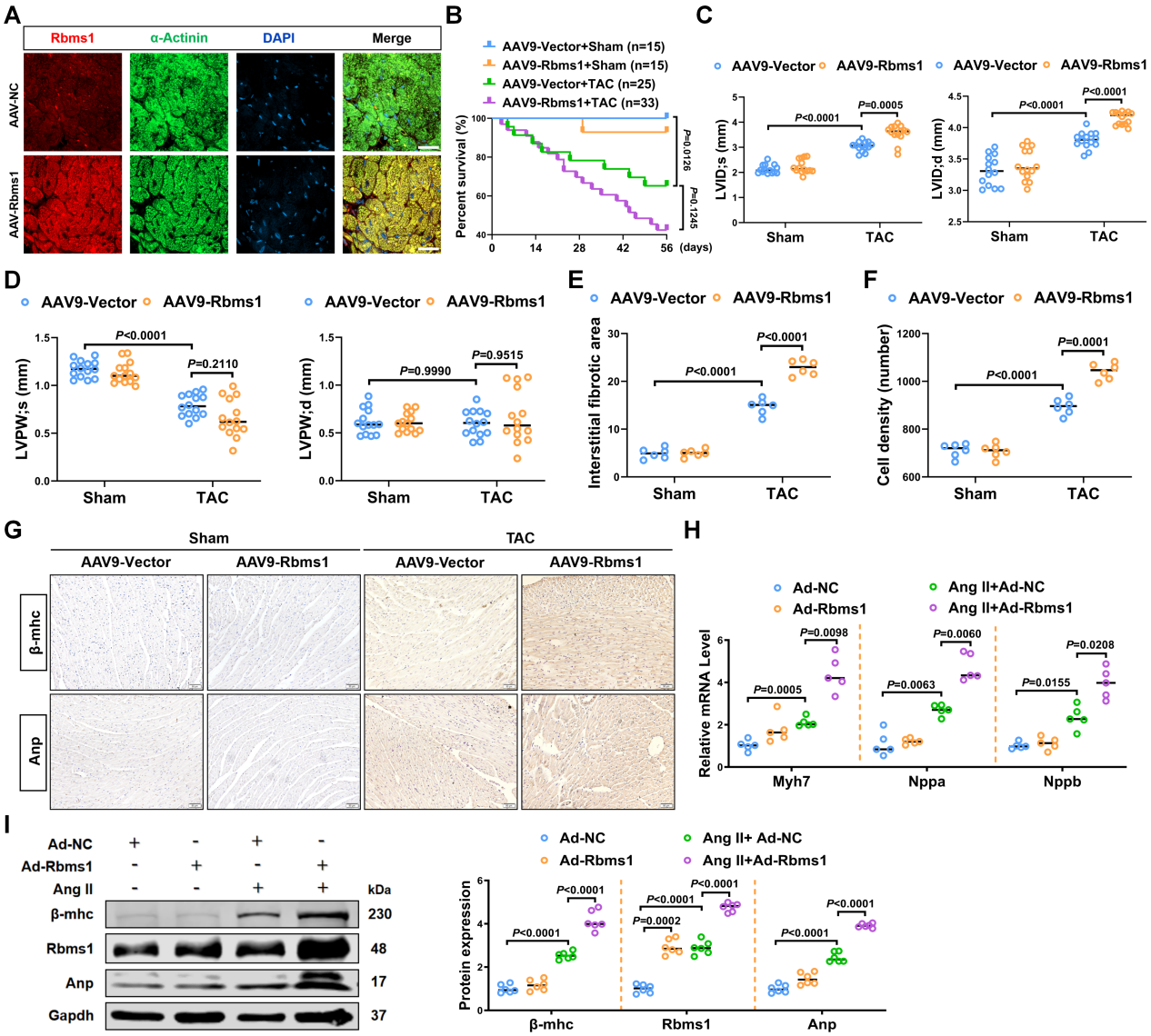


**Appendix Figure S1. Overexpression of RBMS1 aggravates cardiac hypertrophy in response to hypertrophic stimulation.**

(**A**) Representative immunofluorescence staining of RBMS1 and α-Actinin in AAV9-RBMS1 mice. Scale bar=20 μm. (**B**) Kaplan-Meier analysis of TAC mice injected with AAV9-RBMS1 (n=14 to 17). (**C** and **D**) Quantification of LVID;s, LVID;d, LVPW;s, and LVPW;d (n=14). (**E**) Quantification of the interstitial fibrotic area (n=6). (**F**) Quantification of cell density in fibrotic area to assess inflammatory infiltration of the mouse heart (n=6). (**G**) IHC staining of heart samples. scale bar=50 μm. (**H**) Quantification of mRNA levels of β-MHC, ANP, and BNP in NMCMs transfected with Ad-RBMS1 in response to Ang II stimulation (n=5). (**I**) Western blotting and quantification showing protein levels of β-MHC, RBMS1, and ANP (n=6). A dot represents an independent biological sample.


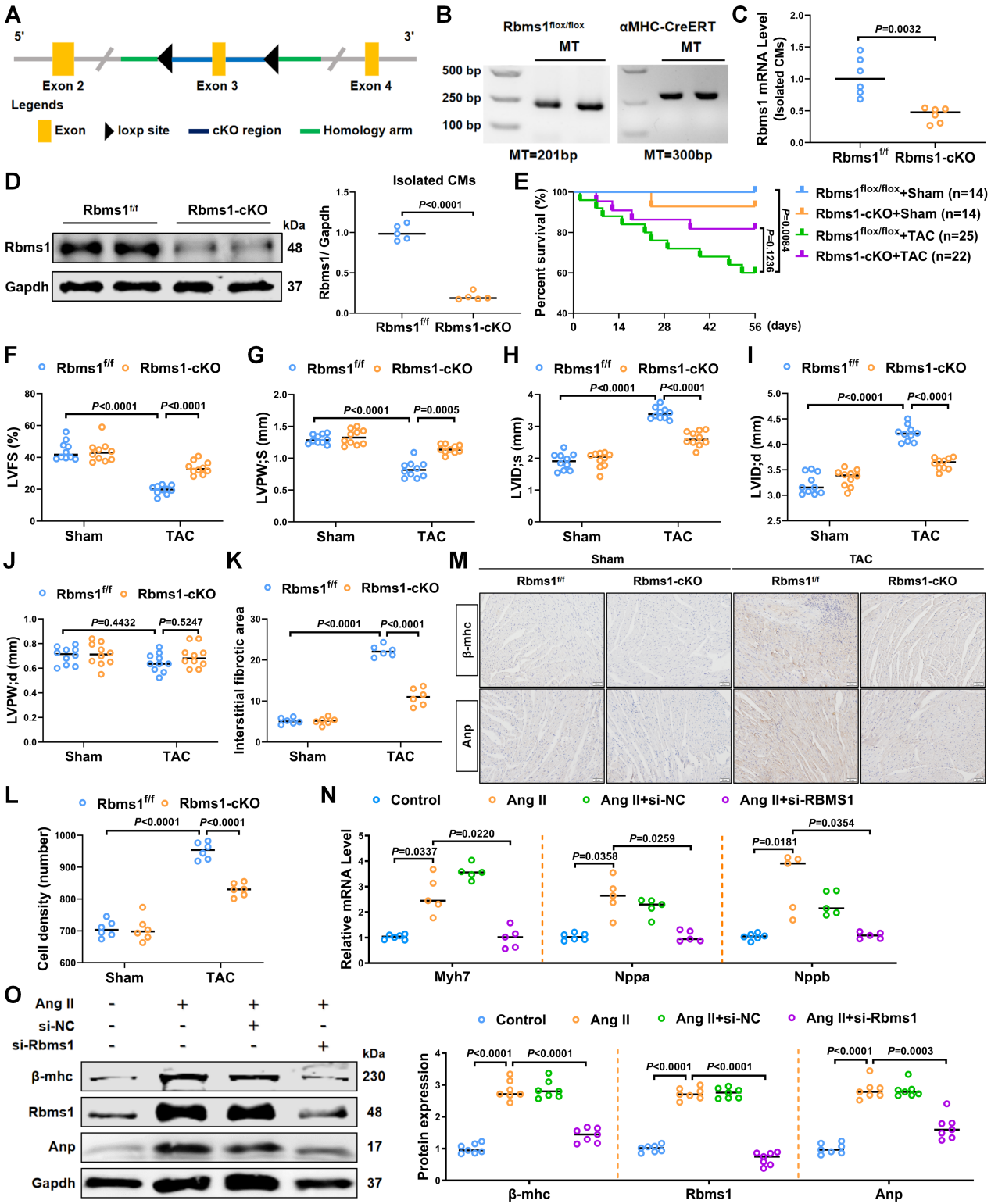


**Appendix Figure S2. Knockout of RBMS1 attenuates cardiac hypertrophy.**

1. Schematic diagram showing the construction of RBMS1-cKO mice. (**B**) Tail identification of RBMS1^flox/flox^ and MYH6-Cre mice. (**C**) Quantification of mRNA levels of RBMS1 in cardiomyocytes isolated from WT and RBMS1-cKO mice (n=6). (**D**) Western blotting and quantification showing protein levels of RBMS1 (n=5). (**E**) Kaplan-Meier analysis of RBMS1-cKO mice with TAC surgery (n=13 to 18). (**F** to **J**) Quantification of LVFS, LVPW;s, LVID;s, LVID;d, and LVPW;d (n=10). (**K**) Quantification of the interstitial fibrotic area (n=6). (**L**) Quantification of cell density in fibrotic area to assess inflammatory infiltration of the mouse heart (n=6). (**M**) IHC staining of heart samples (n=6). scale bar=50 μm. (**N**) Quantification of mRNA levels of β-MHC, ANP, and BNP in NMCMs transfected with si-RBMS1 and subsequently treated with Ang II (n=5). (**O**) Western blotting and quantification showing protein levels of β-MHC, RBMS1, and ANP (n=7). A dot represents an independent biological sample.


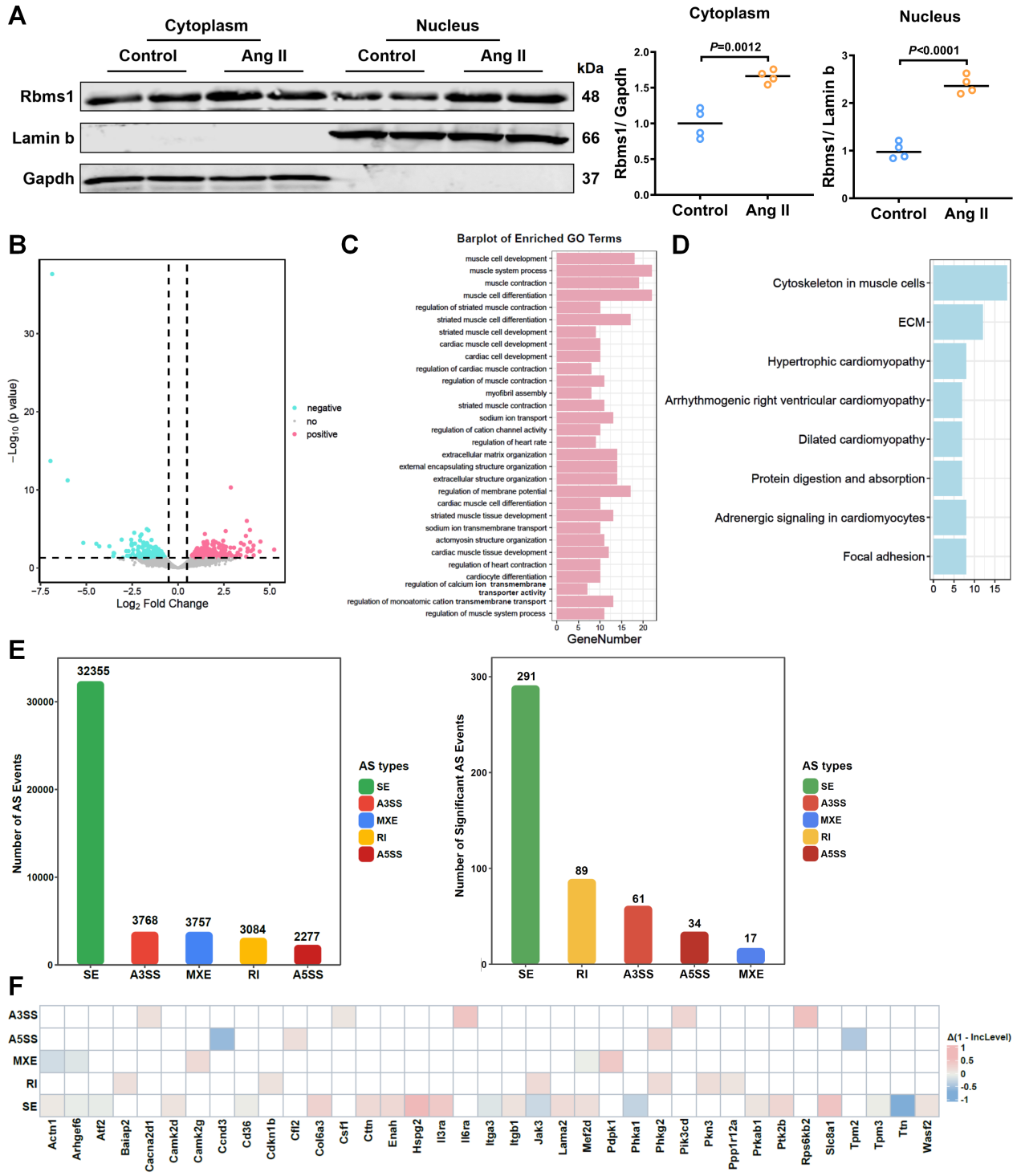


**Appendix Figure S3. High-throughput RNA sequencing analysis of transcriptome differentially expressed and alternative splicing genes in NMCMs.**

(**A**) Western blotting and quantification showing protein expression levels of RBMS1 in the cytoplasm and nucleus of NMCMs induced by Ang II (n=4). (**B**) Volcanic plot elucidated the differentially expressed genes in NMCMs transfected with Ad-RBMS1. Pink represents up-regulated, and blue represents down-regulated. (**C**) GO enrichment analysis (false discovery rate-corrected *P*<0.05) of differentially expressed genes in NMCMs transfected with Ad-RBMS1. (**D**) KEGG pathway enrichment analysis (false discovery rate-corrected *P*<0.05) of differentially expressed genes. (**E**) Numbers of AS genes in NMCMs transfected with Ad-RBMS1. (**F**) Schematic diagrams showed the detailed information of differentially expressed splicing genes. Skipping Exon (SE), Alternative 3′ Splice Site (A3SS), Mutually Exclusive Exons (MXE), Retained intron (RI), Alternative 5′ Splice Site (A5SS). A dot represents an independent biological sample.

**
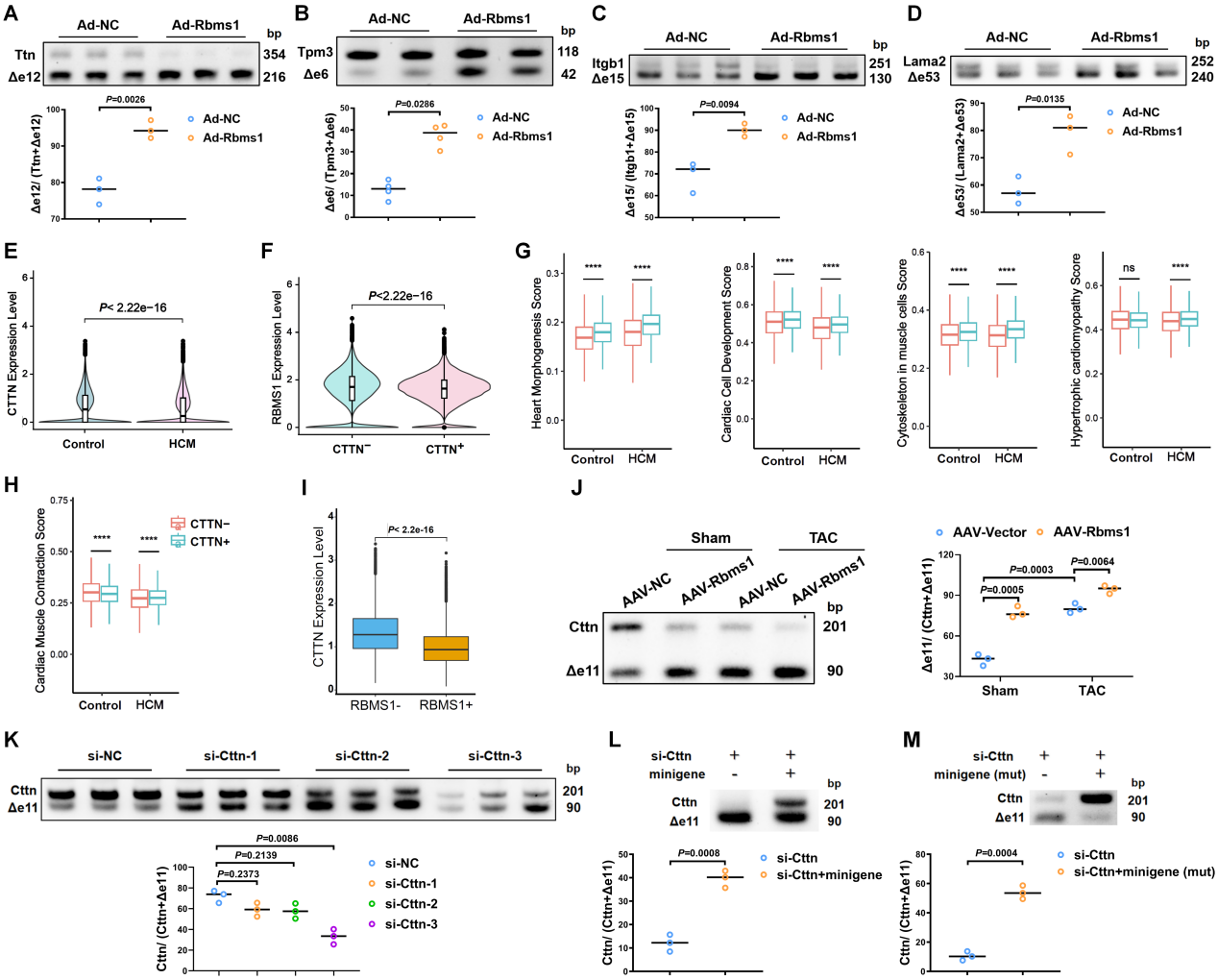
**

**Appendix Figure S4. Analysis of CTTN expression and functional enrichment in HCM patients.**

(**A** to **D**) Splicing pattern and quantification of TTN, TPM3, ITGB1, and LAMA2 in NMCMs transfected with Ad-RBMS1 (n=3 or 4). (**E**) The expression of CTTN in HCM patients. (**F**) The expression of RBMS1 in cardiomyocytes with high expression of CTTN. (**G** and **H**) Scores of GO and KEGG gene sets in cardiomyocytes. (**I**) The expression of CTTN in cardiomyocytes with high expression of RBMS1. (**J**) Splicing pattern and quantification of CTTN in RBMS1 overexpression mice treatment with TAC (n=3). (**K**) Knockdown efficiency of CTTN in NMCMs transfected with si-CTTN (n=3). (**L**) Transfection efficiency of minigene in NMCMs transfected with si-CTTN and minigene (n=3). (**M**) Transfection efficiency of minigene (mut) in NMCMs transfected with si-CTTN and minigene (mut) (n=3). A dot represents an independent biological sample.


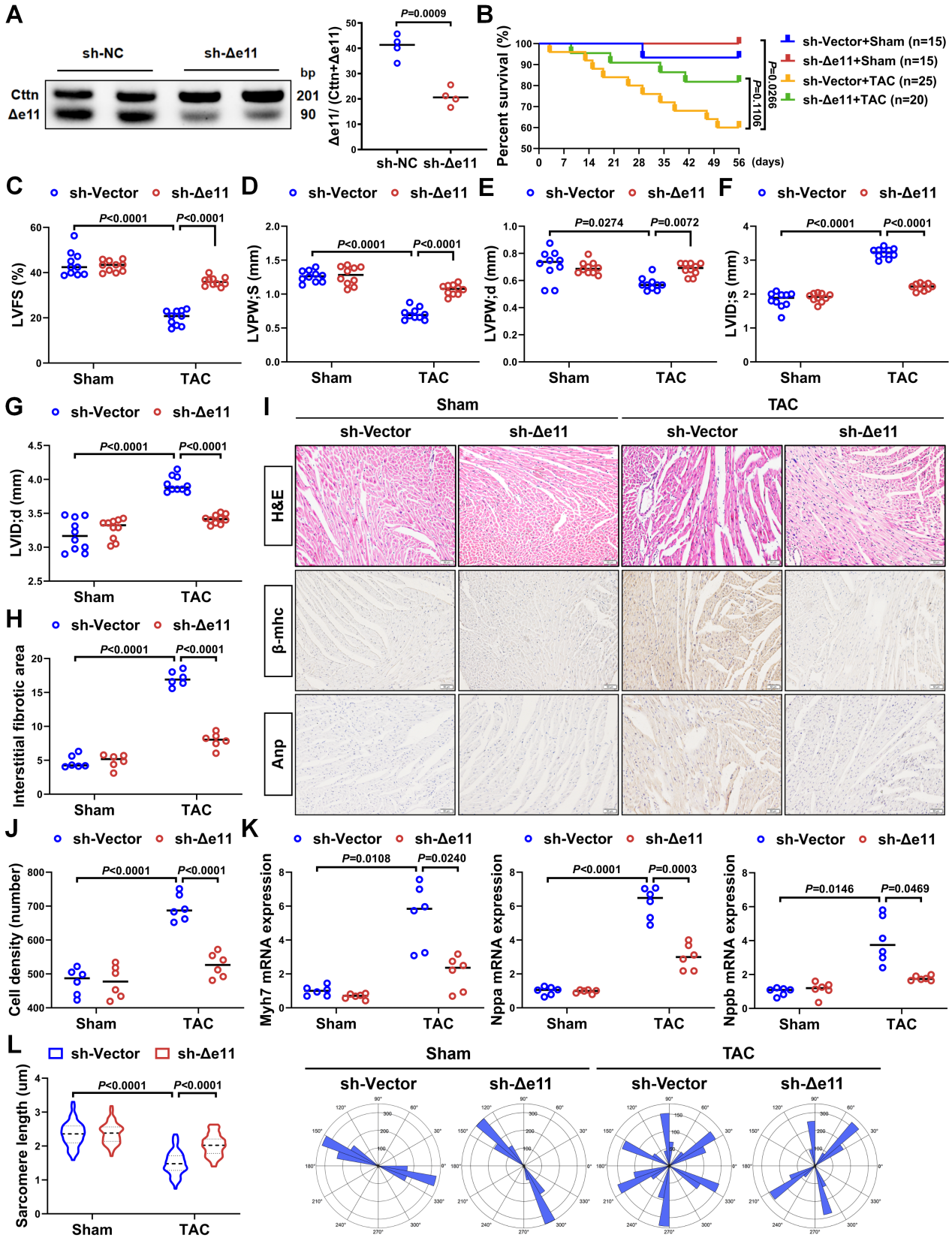


**Appendix Figure S5. Cardiac-specific knockout of CTTN-Δe11 attenuates TAC-induced cardiac hypertrophy.**

(**A**) Knockdown efficiency of CTTN-Δe11 in cardiomyocytes isolated from sh-Δe11 mice (n=4). (**B**) Kaplan-Meier analysis of WT mice injected with sh-Δe11 for 1 week and subsequently subjected to TAC surgery for 8 weeks (n=14 to 16). (**C-G**) Quantification of LVFS, LVPW;s, LVPW;d, LVID;s, and LVID;d (n=10). (**H**) Quantification of the interstitial fibrotic area (n=6). (**I**) H&E and IHC staining of heart samples (n=6). scale bar=50 μm. (**J**) Quantification of cell density in fibrotic area to assess inflammatory infiltration (n=6). (**K**) Quantification of mRNA levels of β-MHC, ANP, and BNP (n=6). (**L**) Quantification of sarcomere length and representative polarity histogram of sarcomere organization in sh-Δe11 mice with sham or TAC surgery (n=70). A dot represents an independent biological sample.


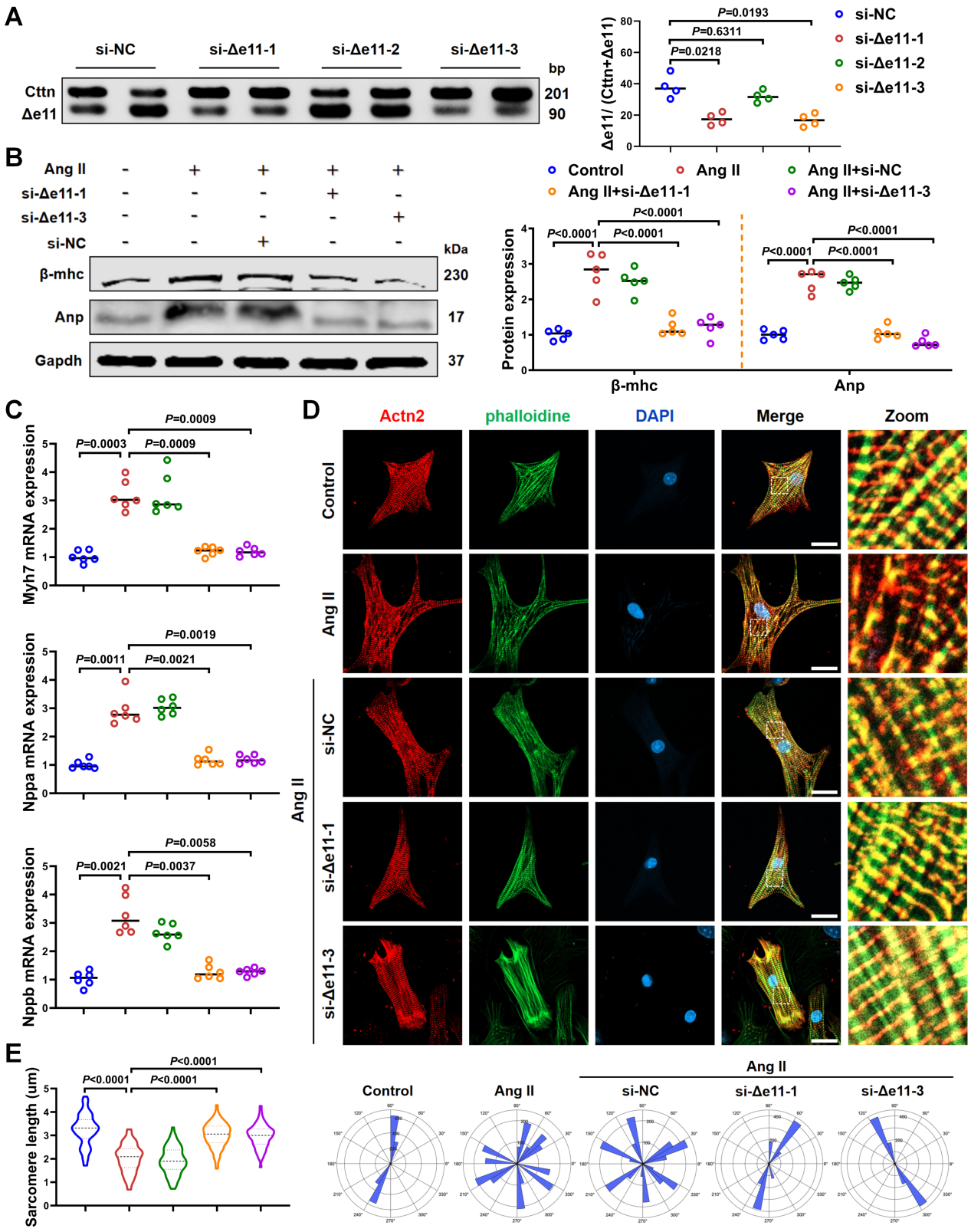


**Appendix Figure S6. Silencing of CTTN-Δe11 inhibits cardiac hypertrophy *in vitro*.**

(**A**) Knockdown efficiency of CTTN-Δe11 in NMCMs transfected with si-Δe11 was verified by RT-PCR (n=4). (**B**) Western blotting and quantification showing protein levels of β-MHC and ANP in NMCMs transfected with si-Δe11 in response to Ang II treatment (n=5). (**C**) Quantification of mRNA levels of β-MHC, ANP, and BNP (n=6). (**D**) Immunofluorescence staining of ACTN2 and phalloidine showed the disorganization of sarcomere and cytoskeleton of cardiomyocytes. scale bar=20 μm. (**E**) Quantification of sarcomere length and representative polarity histogram of sarcomere organization in NMCMs. n=70 samples per group. A dot represents an independent biological sample.


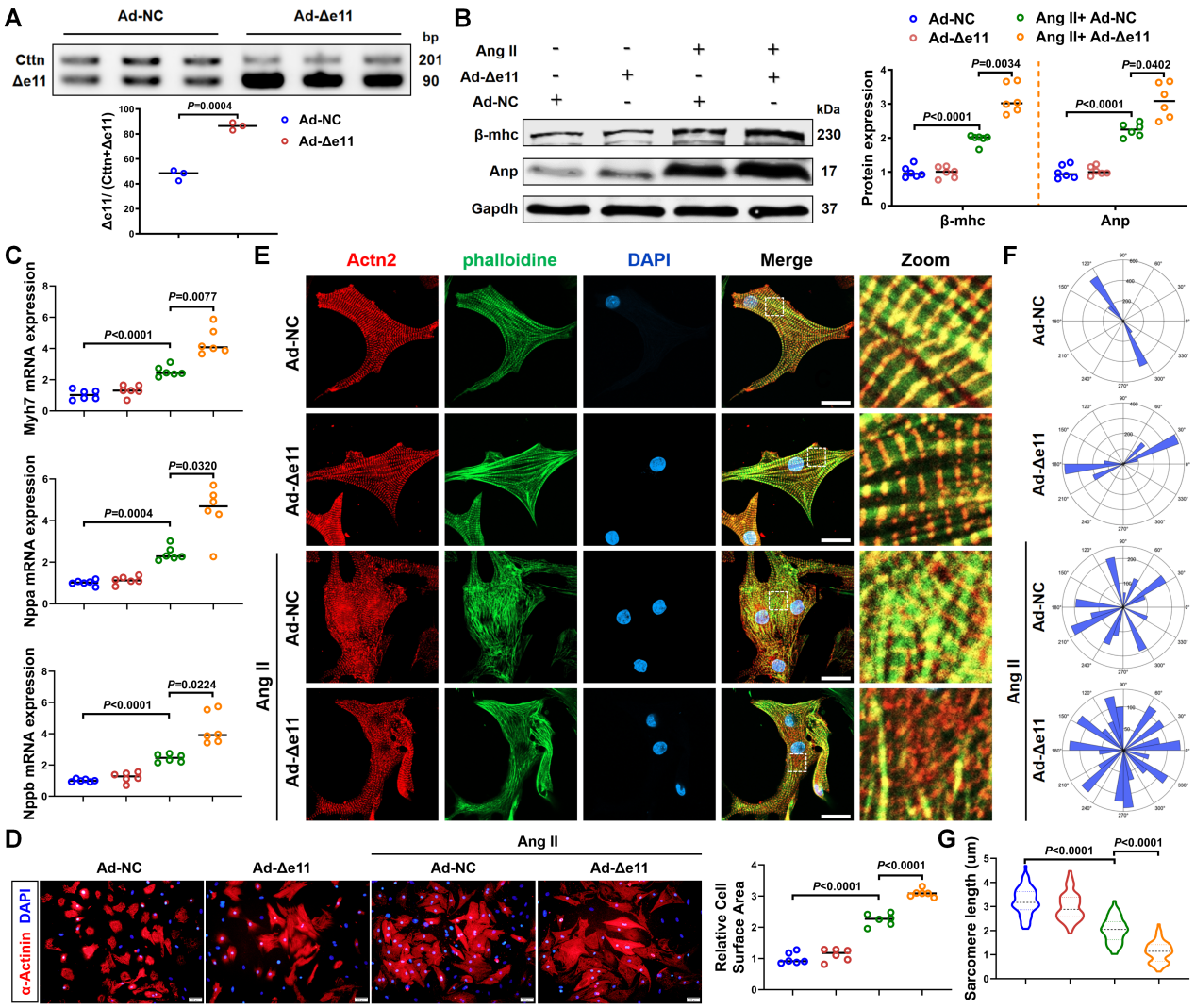


**Appendix Figure S7. CTTN-Δe11 promotes cardiac hypertrophy *in* *vitro*.**

(**A**) Overexpression efficiency of CTTN-Δe11 in NMCMs transfected with Ad-Δe11 (n=3). (**B**) Western blotting and quantification showing protein levels of β-MHC and ANP in NMCMs transfected with Ad-Δe11 in response to Ang II treatment (n=6). (**C**) Quantification of mRNA levels of β-MHC, ANP, and BNP in NMCMs (n=6). (**D**) Representative immunofluorescence staining of α-actinin and quantification of cell surface area in NMCMs (n=6), scale bar=50 μm. (**E**) Immunofluorescence staining of ACTN2 and phalloidine showed the disorganization of sarcomere and cytoskeleton (n=6), scale bar=20 μm. (**F** and **G**) Representative polarity histogram of sarcomere organization and quantification of sarcomere length in NMCMs (n=70). A dot represents an independent biological sample.


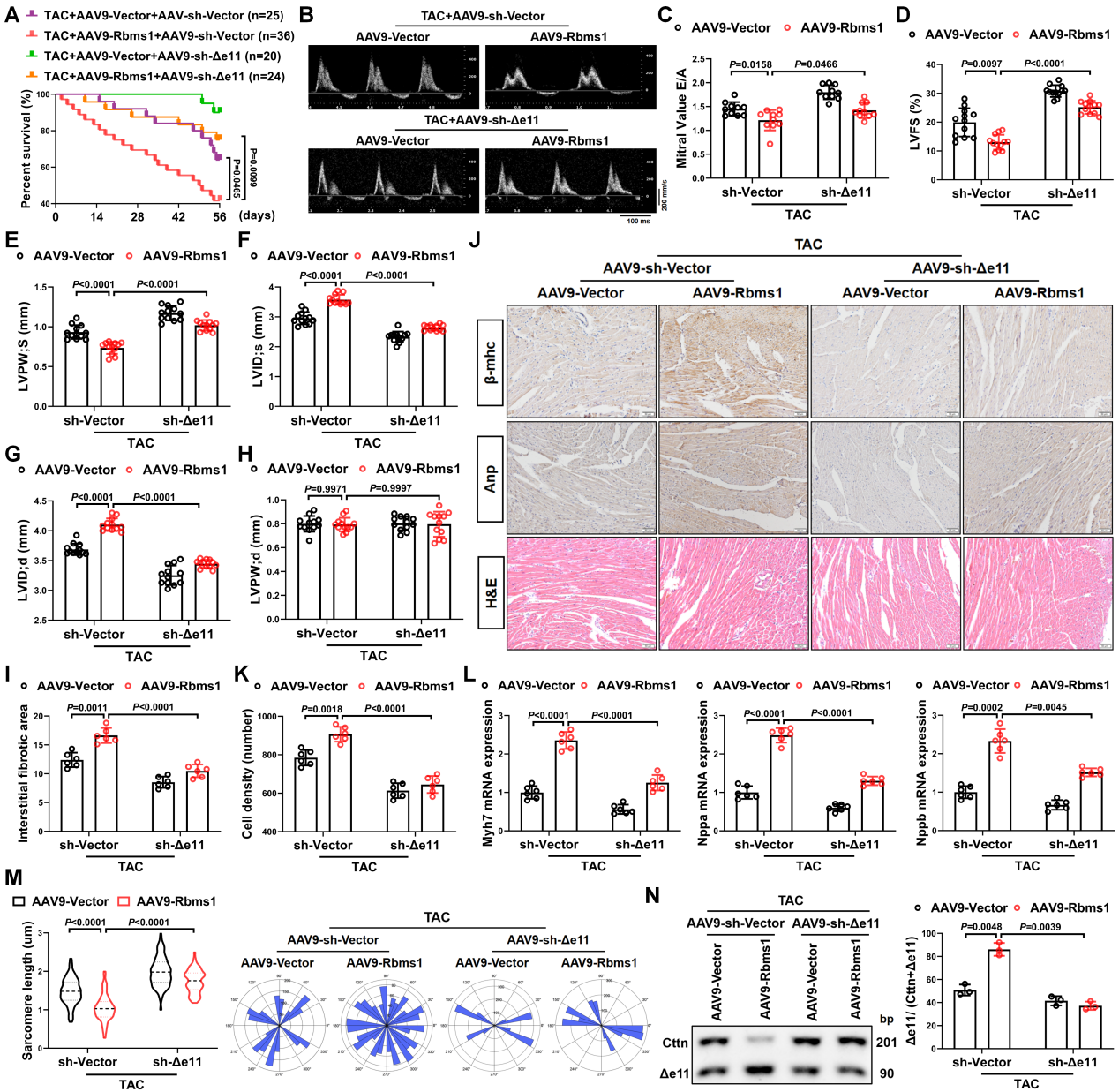


**Appendix Figure S8. RBMS1 promotes cardiac hypertrophy through splicing CTTN.**

(**A**) Kaplan-Meier analysis of AAV9-RBMS1 mice injected with AAV9-sh-Δe11 and subjected to TAC surgery (n=15 to 18). (**B**) Representative echocardiography of AAV9-RBMS1 mice injected with AAV9-sh-Δe11 and subjected to TAC surgery. (**C** to **H**) Quantification of LVFS, mitral value E/A, LVPW;s, LVID;s, LVID;d, and LVPW;d (n=12). (**I**) Quantification of the interstitial fibrotic area (n=6). (**J**) IHC and H&E staining of heart samples (n=6). scale bar=50 μm. (**K**) Quantification of cell density in fibrotic area to assess inflammatory infiltration (n=6). (**L**) Quantification of mRNA levels of β-MHC, ANP, and BNP (n=6). (**M**) Quantification of sarcomere length and representative polarity histogram of sarcomere organization in heart samples (n=70). (**N**) Splicing pattern and quantification of CTTN in RBMS1 overexpression and TAC mice treatment with AAV9-sh-Δe11 (n=3). A dot represents an independent biological sample.


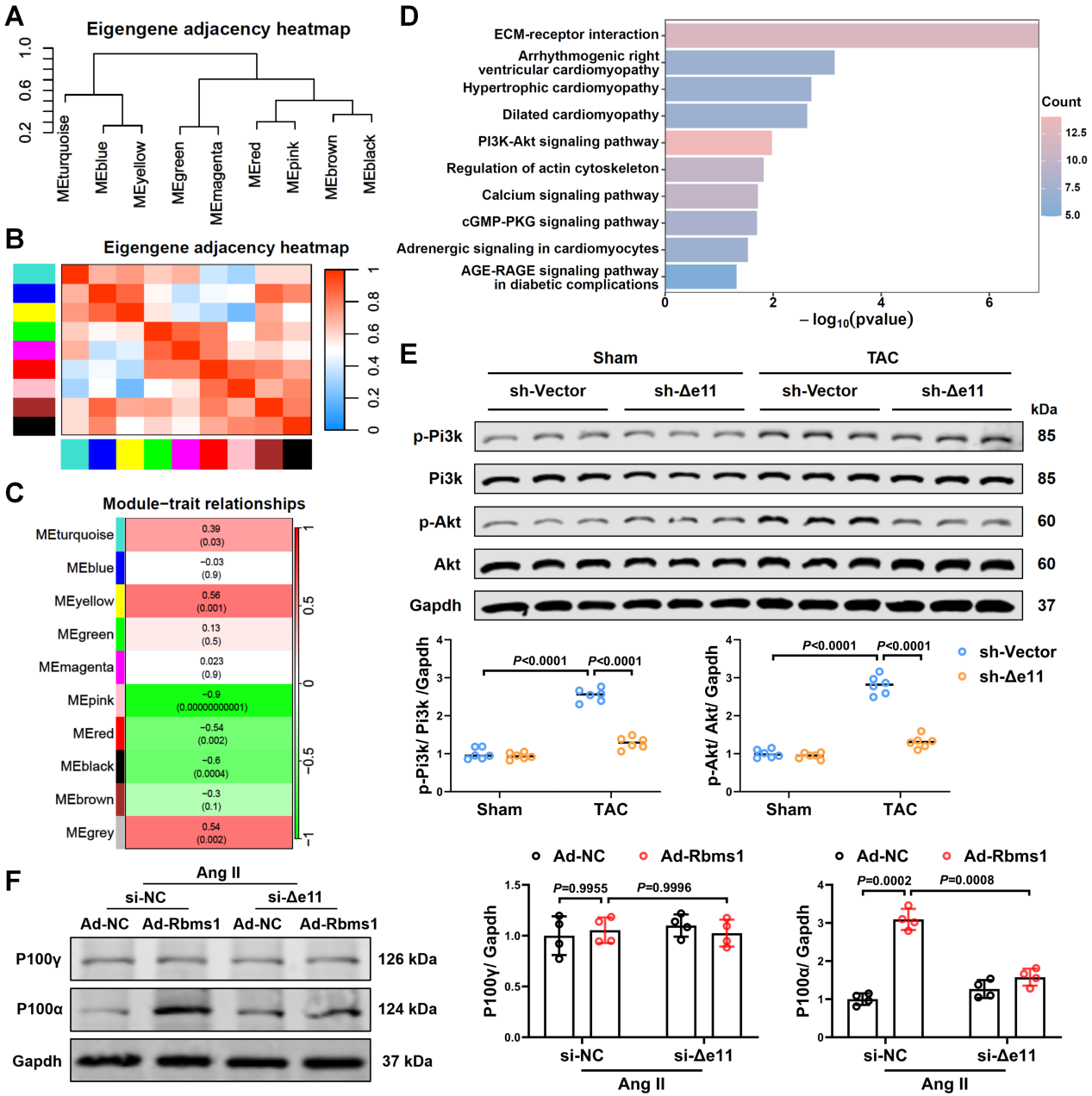


**Appendix Figure S9. RBMS1/CTTN regulates cardiac hypertrophy through the PI3K/AKT signaling pathway.**

(**A**-**C**) Based on GSE135055 data, the RBMS1 co-expression module was constructed using WGCNA method, and the 279 genes co-expressed with RBMS1 (MEyellow) were the most associated with heart failure. (**A**) Hierarchical clustering of module genes that summarize the modules yielded in the clustering analysis. (**B**) Heatmap plot of the adjacencies in the driving gene network. (**C**) Heatmap of the correlation between module eigengenes and the HF. (**D**) Results of GO enrichment analysis based on differentially expressed splicing genes in NMCMs transfected with Ad-RBMS1. (**E**) Western blotting and quantification showing protein levels of p-PI3K and p-AKT in AAV9-RBMS1 mice injected with AAV9-sh-Δe11 and subjected to TAC surgery (n=6). (**F**) Western blotting and quantification showing protein levels of P100α and P100γ in RBMS1 overexpression NMCMs transfected with si-Δe11 in response to Ang II stimulation (n=4). A dot represents an independent biological sample.


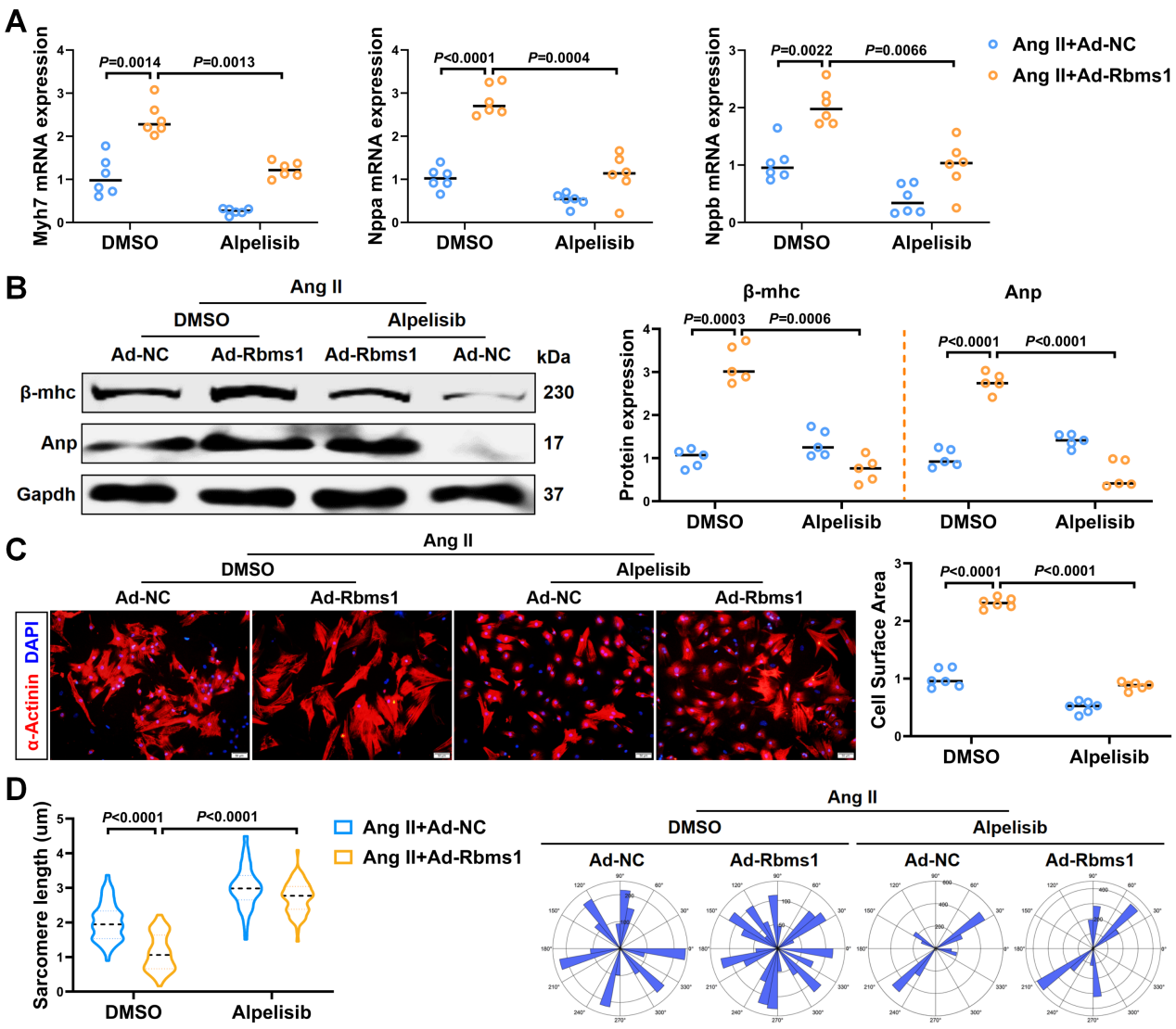


**Appendix Figure S10. RBMS1 promotes cardiac hypertrophy via activating the PI3K-AKT pathway.**

(**A**) Quantification of mRNA levels of β-MHC, ANP, and BNP in RBMS1 overexpression NMCMs treatment with Alpelisib in response to Ang II stimulation (n=6). (**B**) Western blotting and quantification showing protein levels of β-MHC and ANP (n=5). (**C**) Representative immunofluorescence staining of α-actinin and quantification of cell surface area (n=6), scale bar=50 μm. (**D**) Quantification of sarcomere length and representative polarity histogram of sarcomere organization in NMCMs (n=70). A dot represents an independent biological sample.


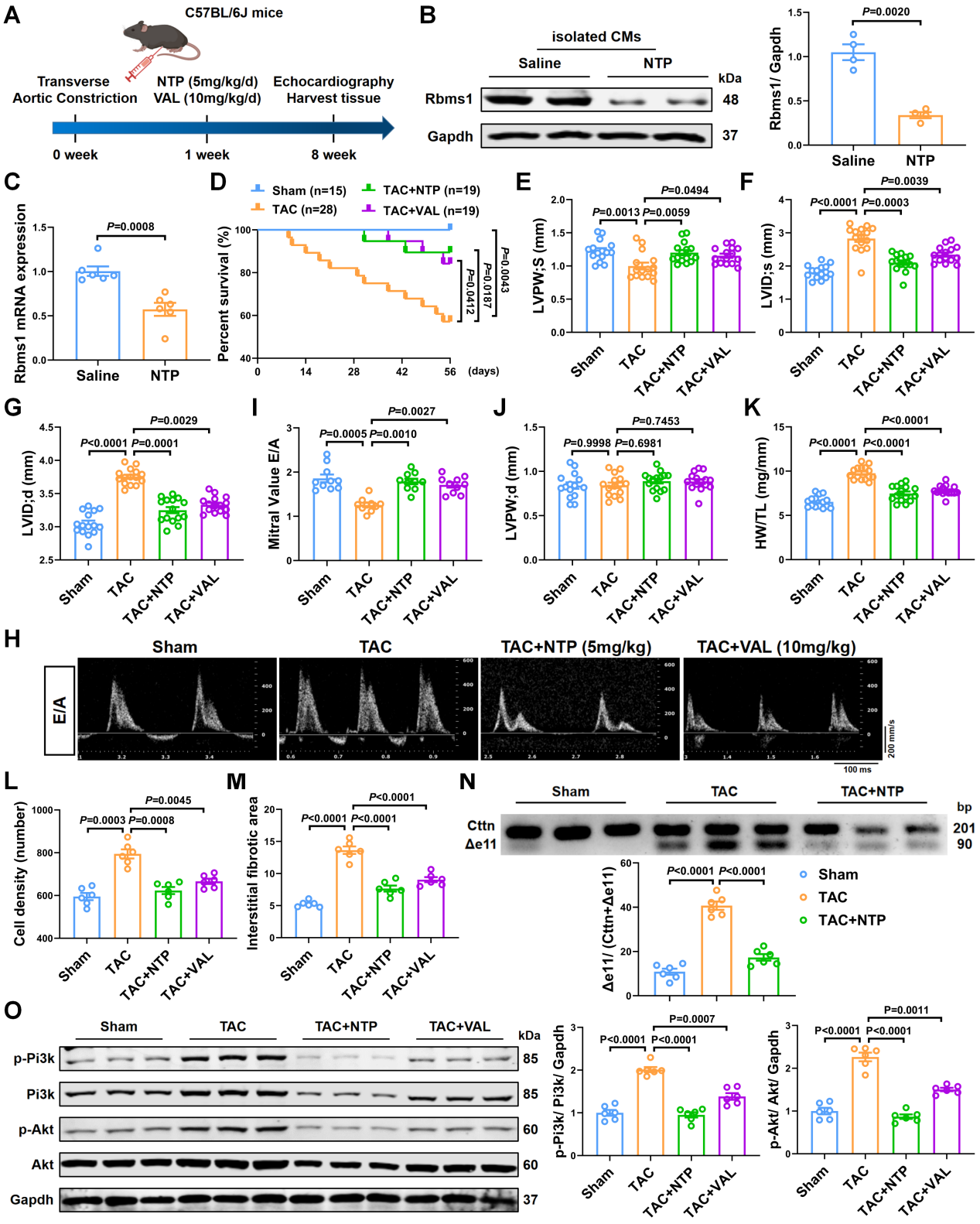


**Appendix Figure S11. Nortriptyline** **attenuates cardiac hypertrophy in mice.**

(**A**) Experimental protocol: Mice were subjected to sham or TAC surgery for 1 week, then were intraperitoneally injected with NTP (5 mg/kg) and VAL (10 mg/kg) for 7 weeks until harvesting. (**B**) The protein expression of RBMS1 in cardiomyocytes isolated from Saline and NTP mice (n=4). (**C**) The mRNA expression of RBMS1 in cardiomyocyte isolated from WT and NTP mice (n=6). (**D**) Kaplan-Meier analysis of TAC mice injected with NTP or VAL. (**E**-**G**) Quantification of LVPW;s, LVID;s, and LVID;d (n=15). (**H** and **I**) Representative echocardiography and quantification of E/A (n=10). (**J**) Quantification of LVPW;d (n=15). (**K**) Quantification of HW/TL in NTP and VAL mice (n=15). (**L**) Quantification of cell density in fibrotic area to assess inflammatory infiltration (n=6). (**M**) Quantification of the interstitial fibrotic area (n=6). (**N**) The expression of CTTN and Δe11 in TAC mice treatment with NTP (n=6). (**O**) Western blotting and quantification showing protein levels of p-PI3K and p-AKT (n=6). A dot represents an independent biological sample.

**Appendix Table S1. Characteristics of dilated cardiomyopathy patients.**

|  | **Previous history** | **WHO class** | **Prior treatment** | **Hospitalization** |
| --- | --- | --- | --- | --- |
| DCM-1 | Dilated cardiomyopathy (1 years) | IV | Anti-infection, cardiotonic, diuretic, anti-heart failure, and anti-ventricular remodeling | Strong cardiac diuresis, anti-heart failure, anti-ventricular remodeling, hypoglycemic, anti-inflammatory, and for heart transplantation |
| DCM-2 | Dilated cardiomyopathy (5 years) | IV | Robust cardiac function, diuretic effect, anti-heart failure property, and anti-ventricular remodeling feature | Strong cardiac diuresis, anti-heart failure, anti-ventricular remodeling, anti-infection, and for heart transplantation |
| DCM-3 | Dilated cardiomyopathy (8 years) | IV | Robust cardiac function, diuretic effect, anti-heart failure property, and anti-ventricular remodeling feature | Strong cardiac diuresis, anti-heart failure, anti-ventricular remodeling, anti-infection, and for heart transplantation |
| DCM-4 | Unspecificness | IV | Unspecificness | Strong cardiac diuresis, anti-heart failure, anti-ventricular remodeling, and for heart transplantation |
| DCM-5 | Diabetes (3 years) | IV | Robust cardiac function, diuretic effect, anti-heart failure property, and anti-ventricular remodeling feature | Strong cardiac diuresis, anti-heart failure, anti-ventricular remodeling, hypoglycemic, anti-inflammatory, and for heart transplantation |

**Appendix Table S2. Demographics of patients with dilated cardiomyopathy.**

| **Variable**  **Count [%] or Median [±SD]** | **DCM N=5** |
| --- | --- |
| **General Characteristics** | |
| Age (Years) | 47.2 [±11.03] |
| Weight (kg) | 63.2 [±21.41] |
| **Gender** | |
| Female (%) | 2 (40) |
| Male (%) | 3 (60) |
| **Race** | |
| Yellow (%) | 5 (100) |
| **Clinical parameters** | |
| BMI (kg/m^2^) | 22.31 [±5.40] |
| LVEF (%) | 24.4 [±9.02] |
| Hypersensitive troponin I (pg/ml) | 777.78 [±1565.98] |
| NT-proBNP (pg/ml) | 10480 [±7503.08] |
