# Supplementary figures and images for: RBMS1 orchestrates cardiac hypertrophy by facilitating CTTN splice-switching and sarcomere dynamics (part 1 of 4)
Source: EMBO Mol Med. 2025 Nov 10;17(12):3555–85. doi: 10.1038/s44321-025-00334-z (PMC12686484; doi:10.1038/s44321-025-00334-z)

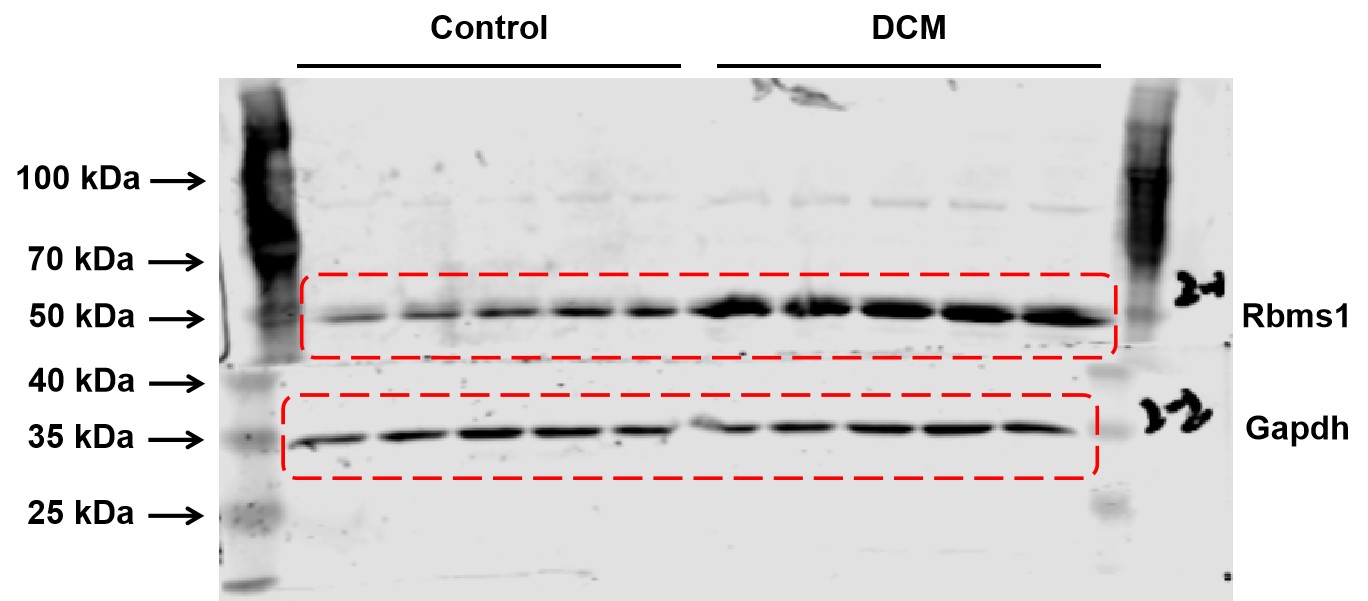

Supplement: Supplementary file 4 — Source data Fig. 1 [file 44321_2025_334_MOESM4_ESM.zip › Figure 1/1D/1D.jpg]

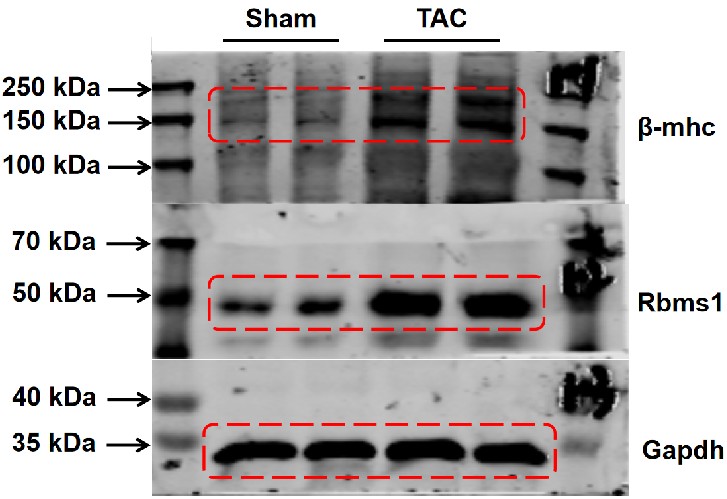

Supplement: Supplementary file 4 — Source data Fig. 1 [file 44321_2025_334_MOESM4_ESM.zip › Figure 1/1G/1G.jpg]

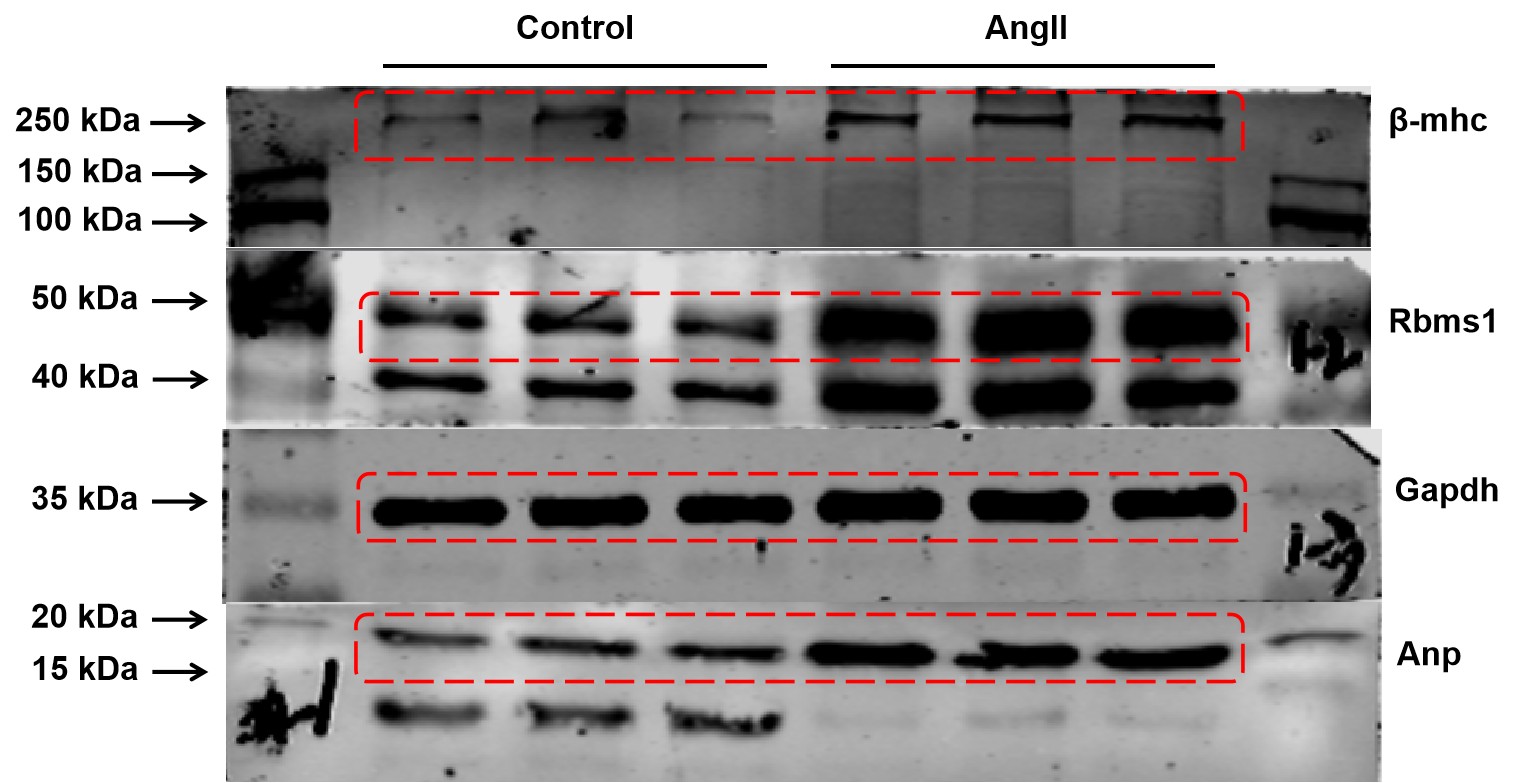

Supplement: Supplementary file 4 — Source data Fig. 1 [file 44321_2025_334_MOESM4_ESM.zip › Figure 1/1L/1L.jpg]

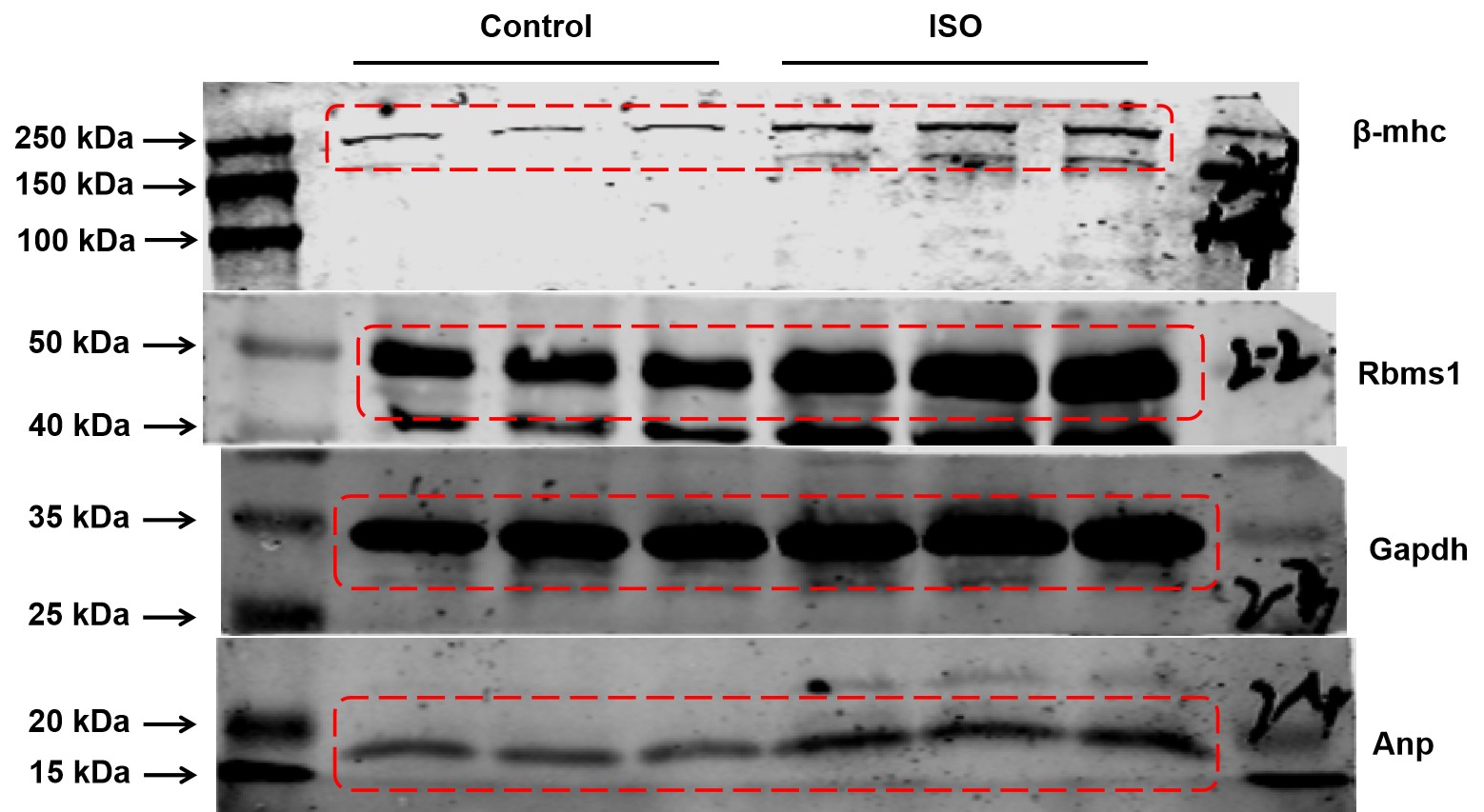

Supplement: Supplementary file 4 — Source data Fig. 1 [file 44321_2025_334_MOESM4_ESM.zip › Figure 1/1N/1N.jpg]

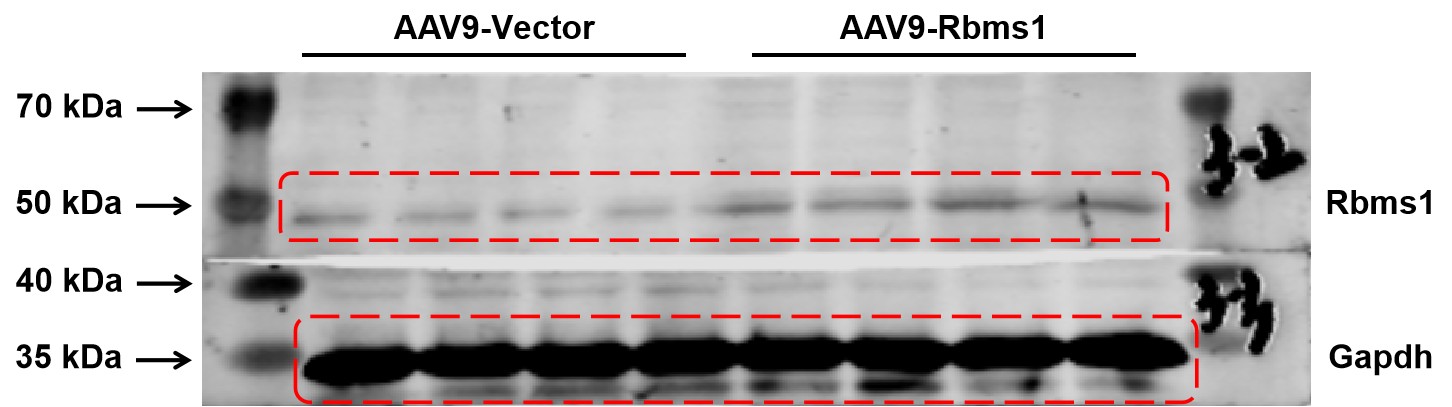

Supplement: Supplementary file 5 — Source data Fig. 2 [file 44321_2025_334_MOESM5_ESM.zip › Figure 2/2B/2B.jpg]

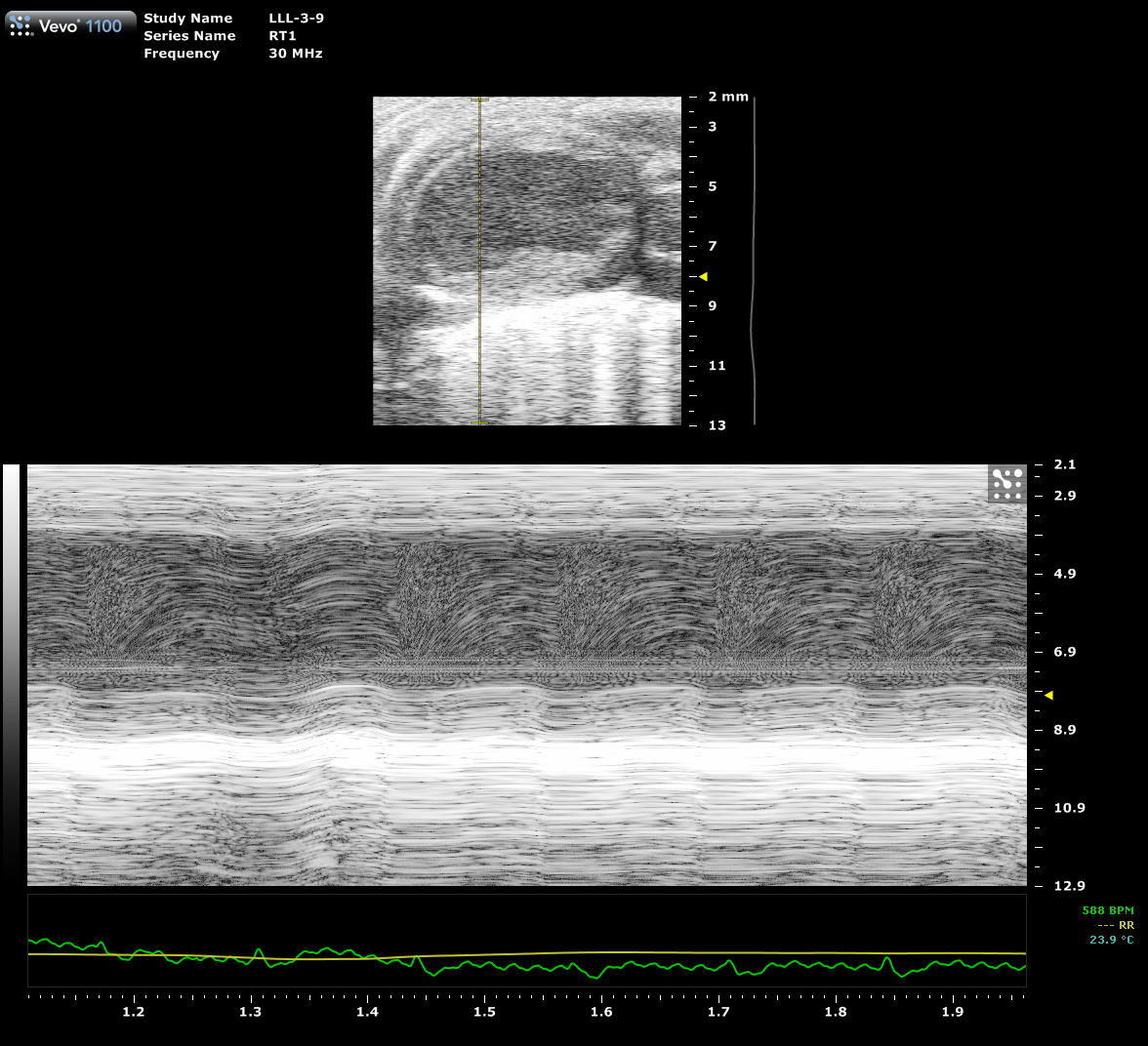

Supplement: Supplementary file 5 — Source data Fig. 2 [file 44321_2025_334_MOESM5_ESM.zip › Figure 2/2D/B Mode/AAV9-RBMS1+TAC.tif]

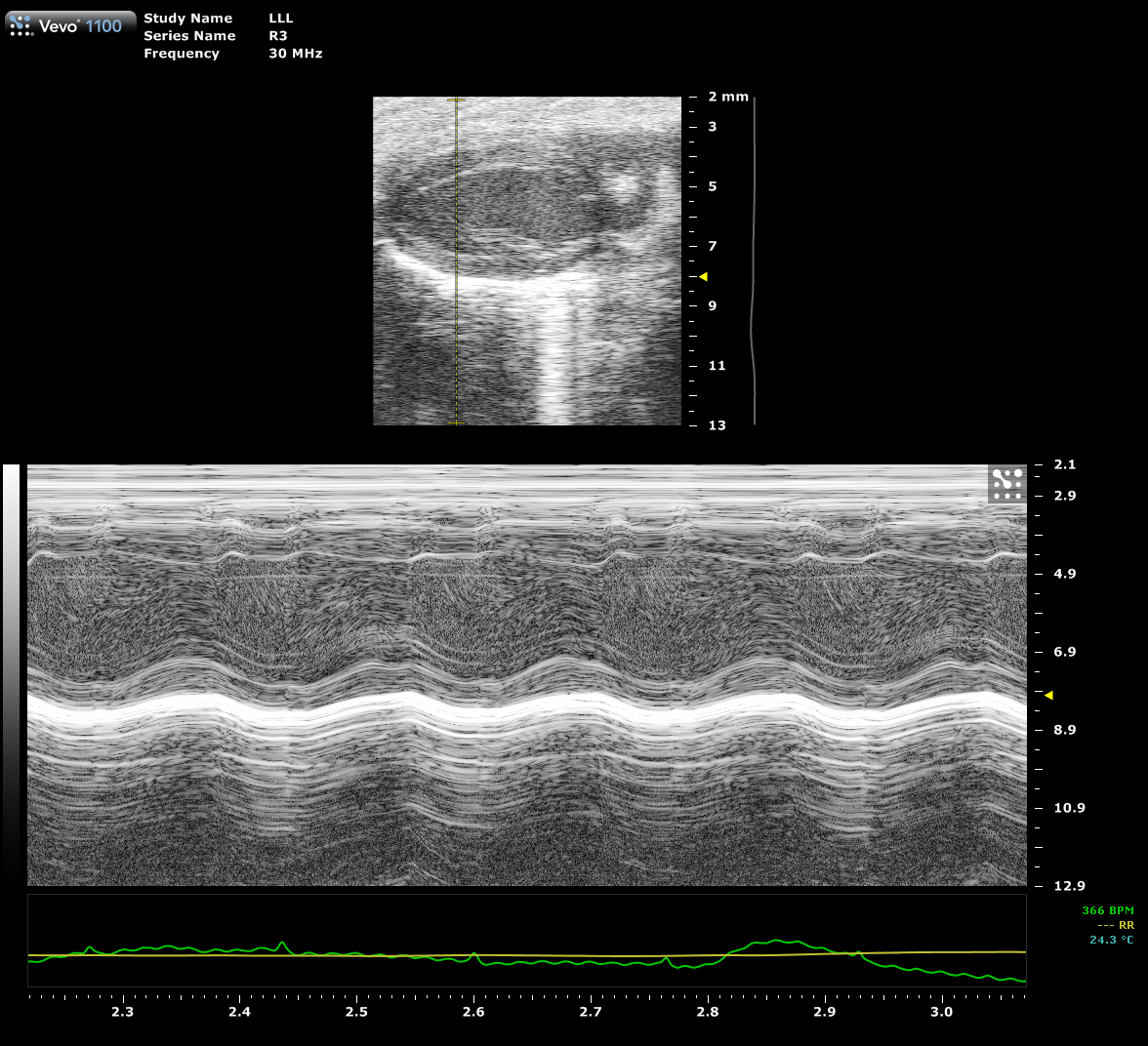

Supplement: Supplementary file 5 — Source data Fig. 2 [file 44321_2025_334_MOESM5_ESM.zip › Figure 2/2D/B Mode/AAV9-RBMS1.tif]

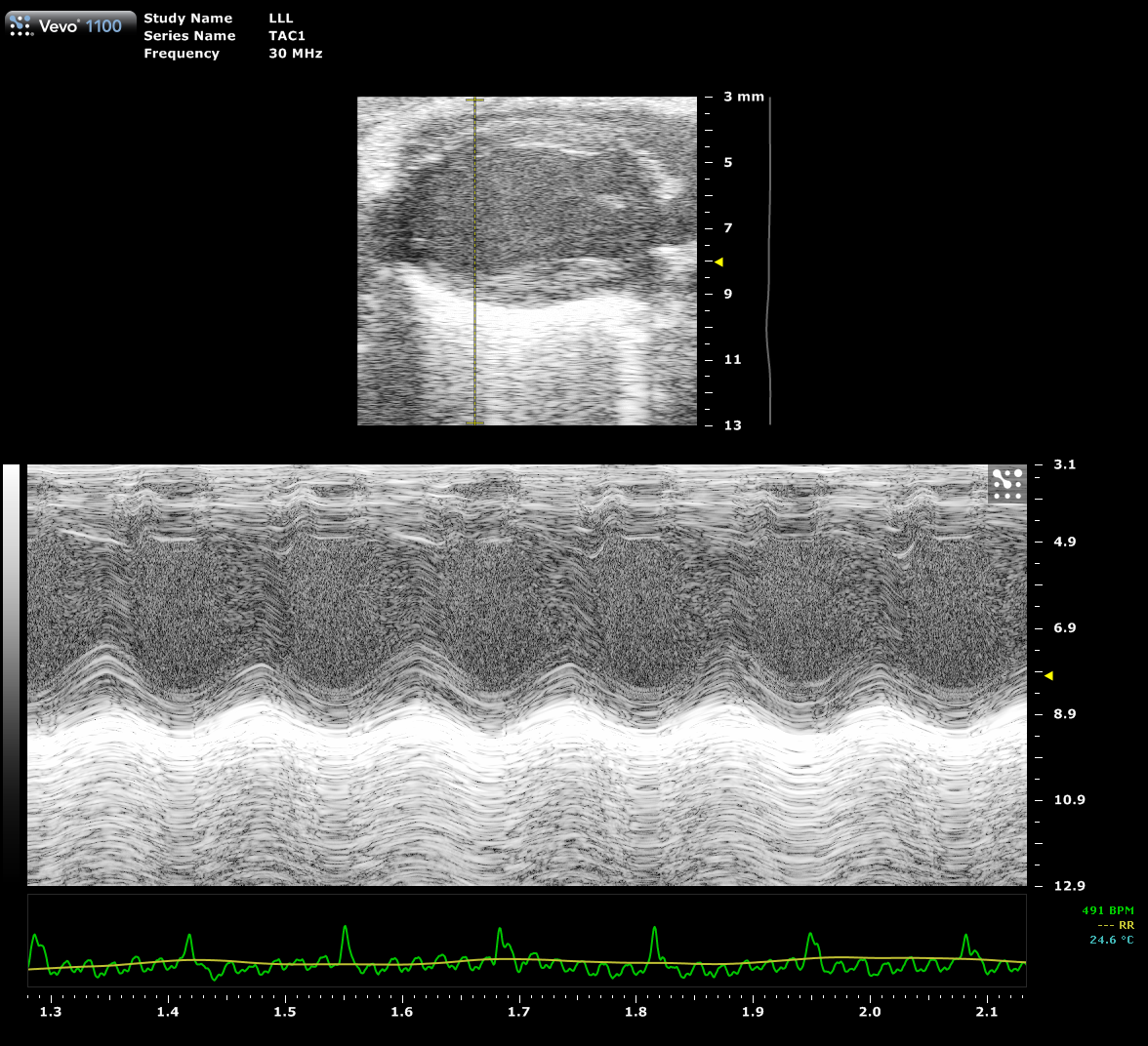

Supplement: Supplementary file 5 — Source data Fig. 2 [file 44321_2025_334_MOESM5_ESM.zip › Figure 2/2D/B Mode/AAV9-Vector+TAC.tif]

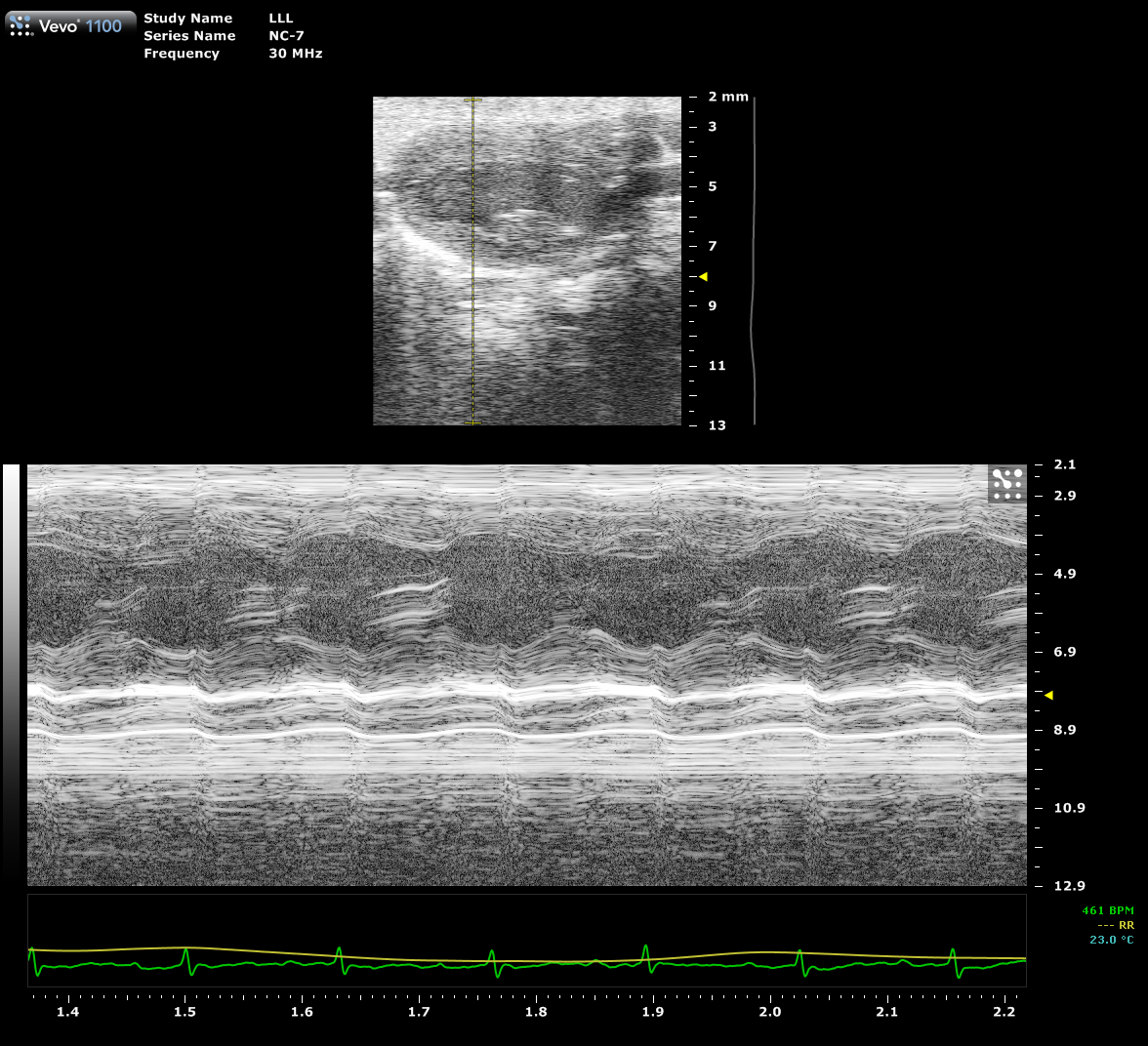

Supplement: Supplementary file 5 — Source data Fig. 2 [file 44321_2025_334_MOESM5_ESM.zip › Figure 2/2D/B Mode/AAV9-Vector.tif]

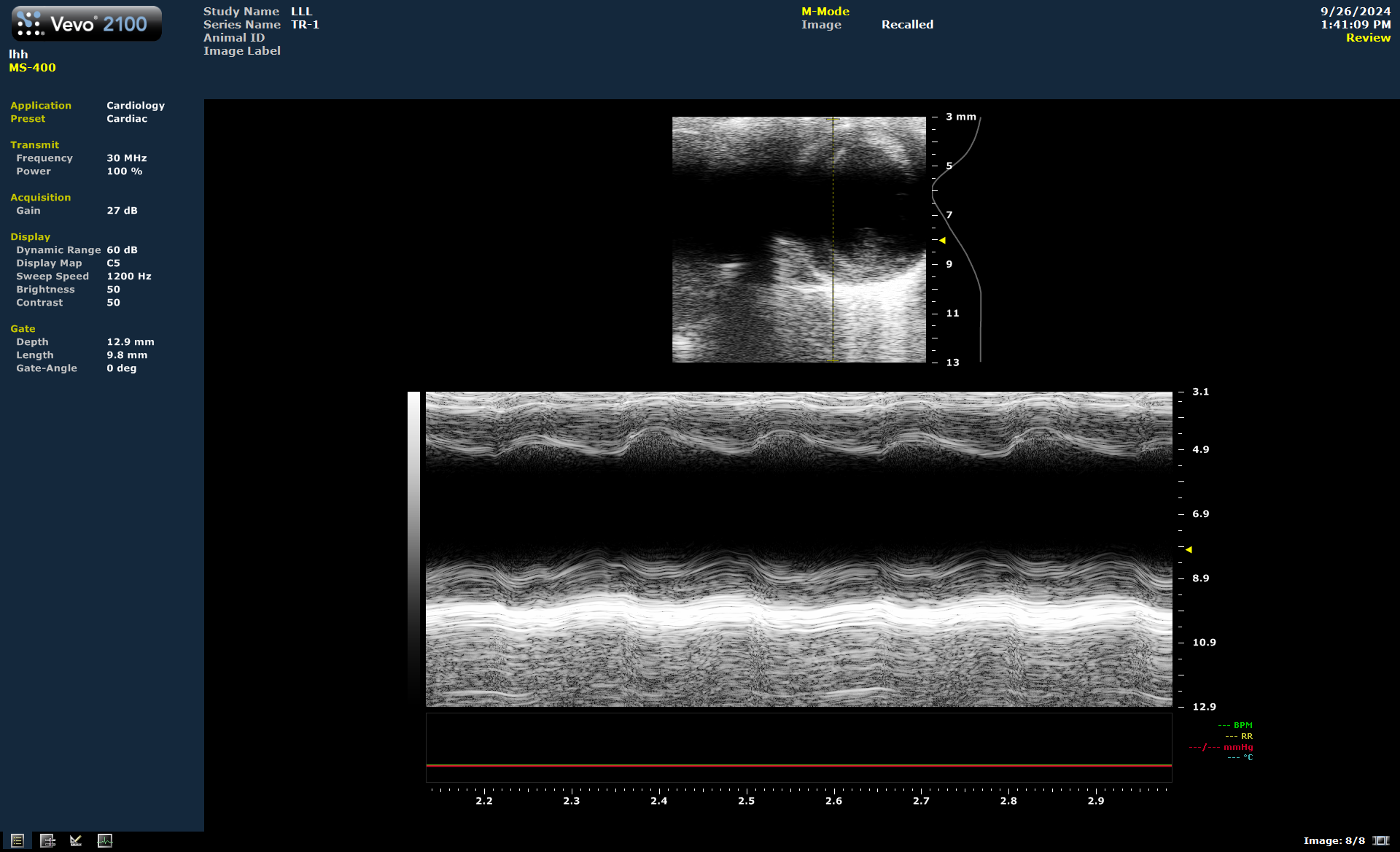

Supplement: Supplementary file 5 — Source data Fig. 2 [file 44321_2025_334_MOESM5_ESM.zip › Figure 2/2D/M Mode/AAV9-RBMS1+TAC.tif]

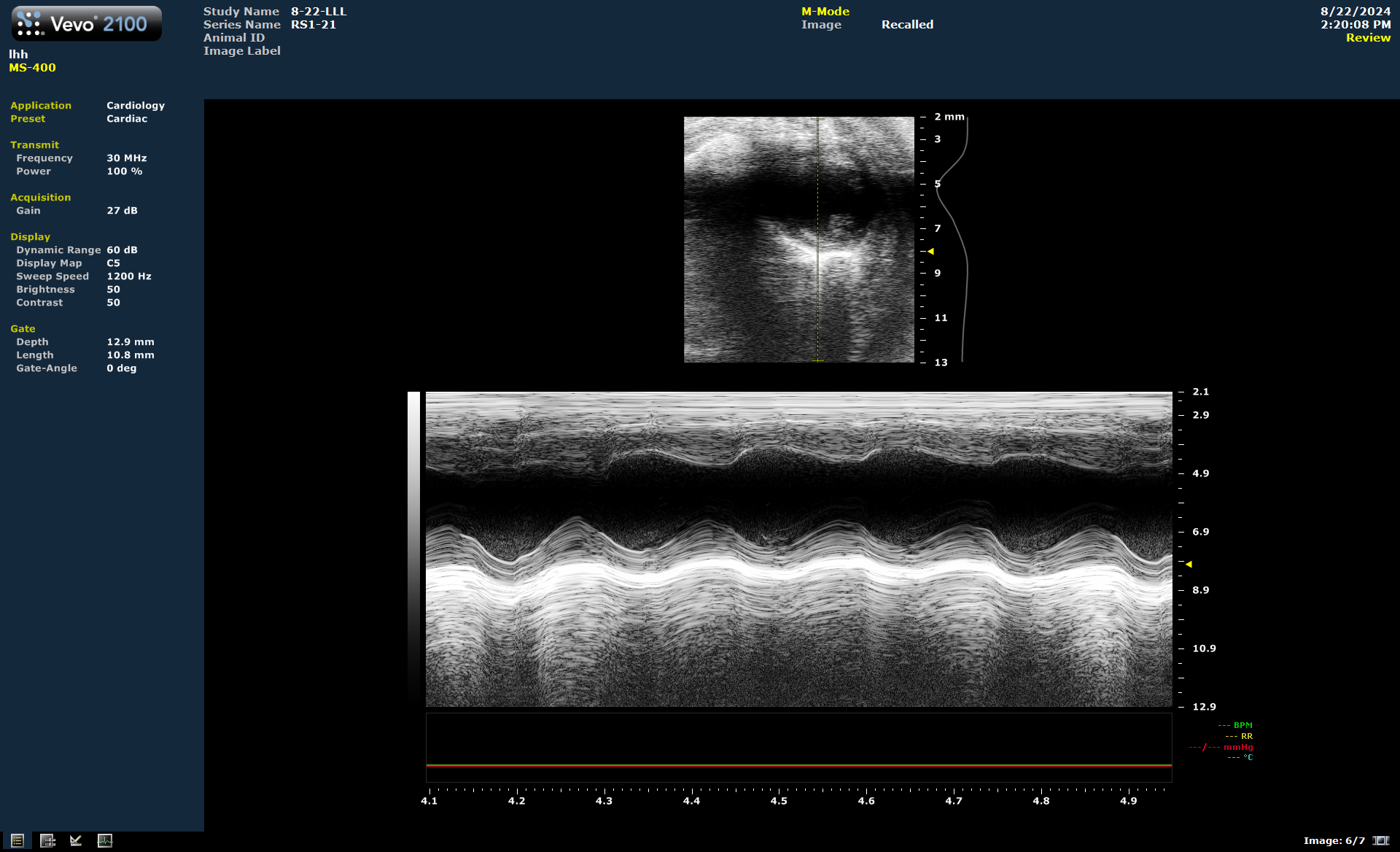

Supplement: Supplementary file 5 — Source data Fig. 2 [file 44321_2025_334_MOESM5_ESM.zip › Figure 2/2D/M Mode/AAV9-RBMS1.tif]

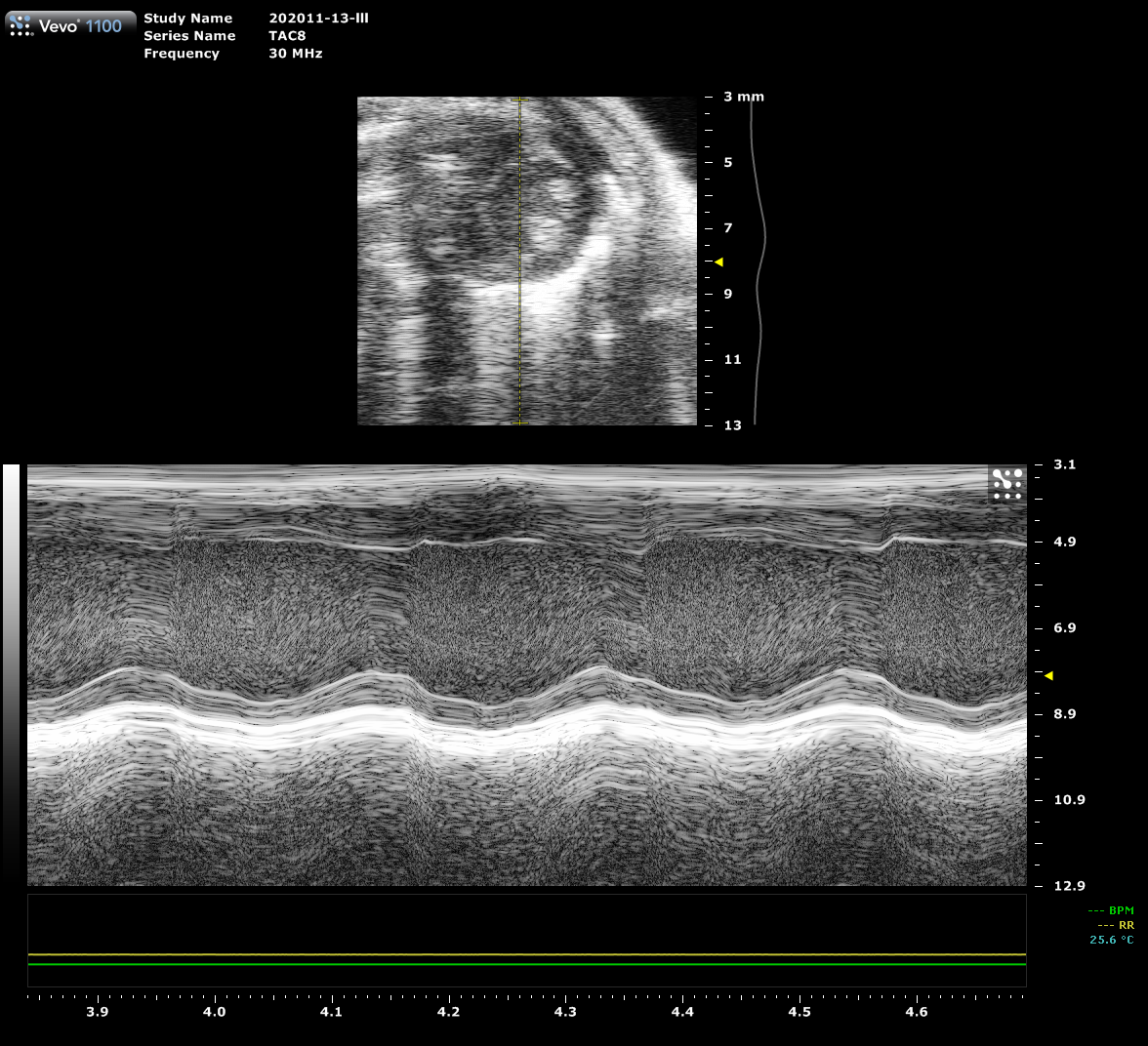

Supplement: Supplementary file 5 — Source data Fig. 2 [file 44321_2025_334_MOESM5_ESM.zip › Figure 2/2D/M Mode/AAV9-Vector+TAC.tif]

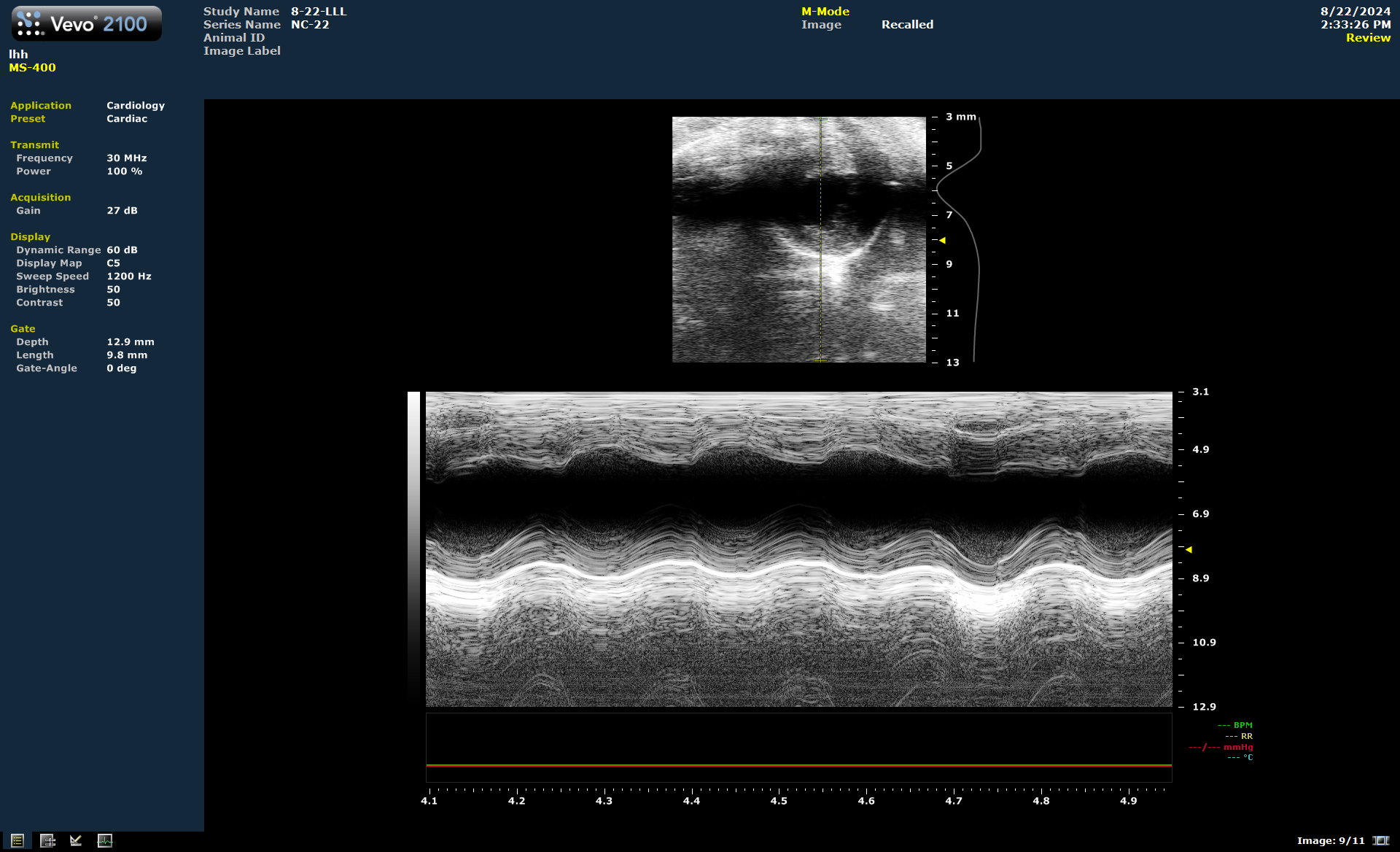

Supplement: Supplementary file 5 — Source data Fig. 2 [file 44321_2025_334_MOESM5_ESM.zip › Figure 2/2D/M Mode/AAV9-Vector.tif]

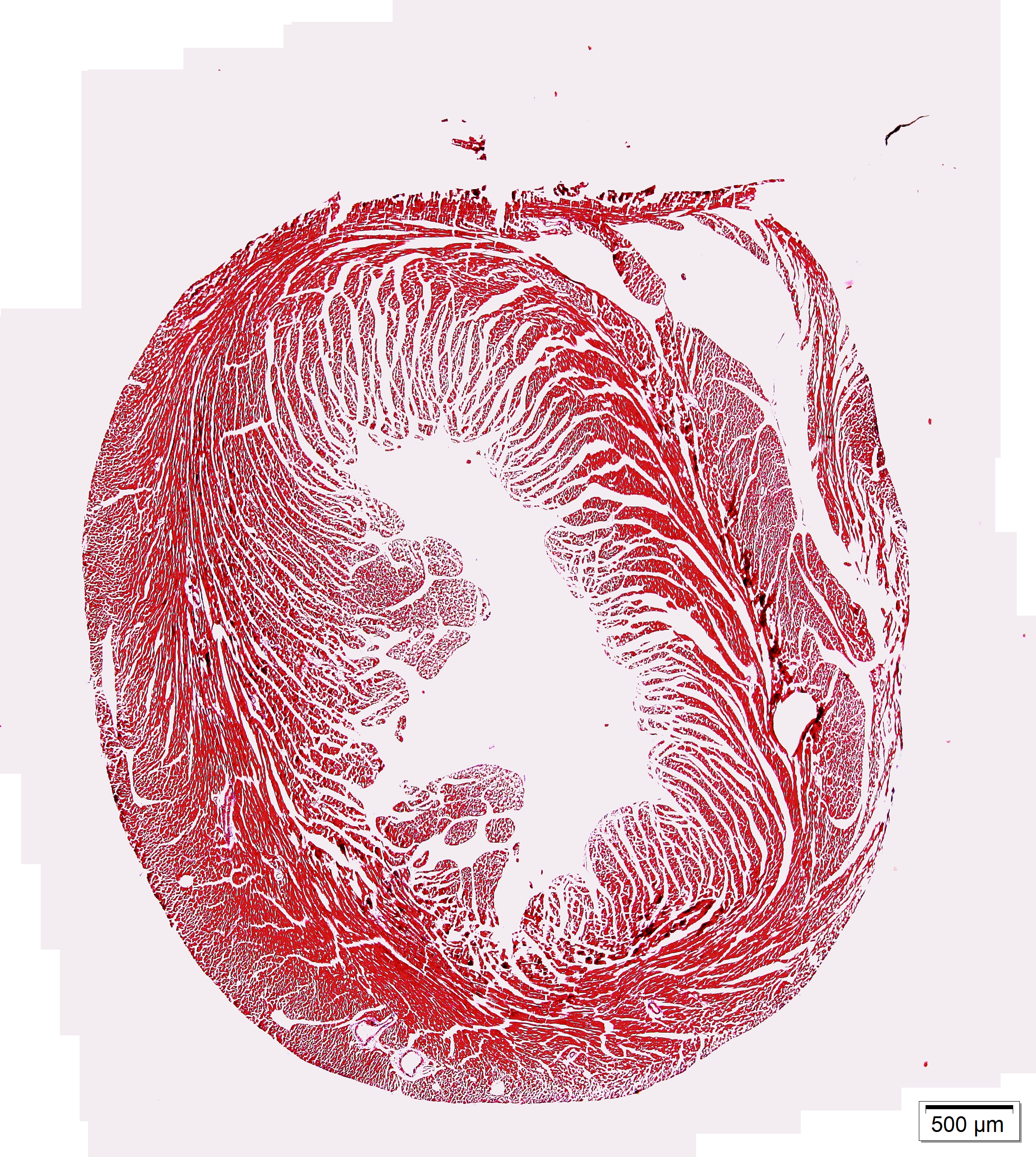

Supplement: Supplementary file 5 — Source data Fig. 2 [file 44321_2025_334_MOESM5_ESM.zip › Figure 2/2F/Cross/AAV9-RBMS1+TAC.tif]

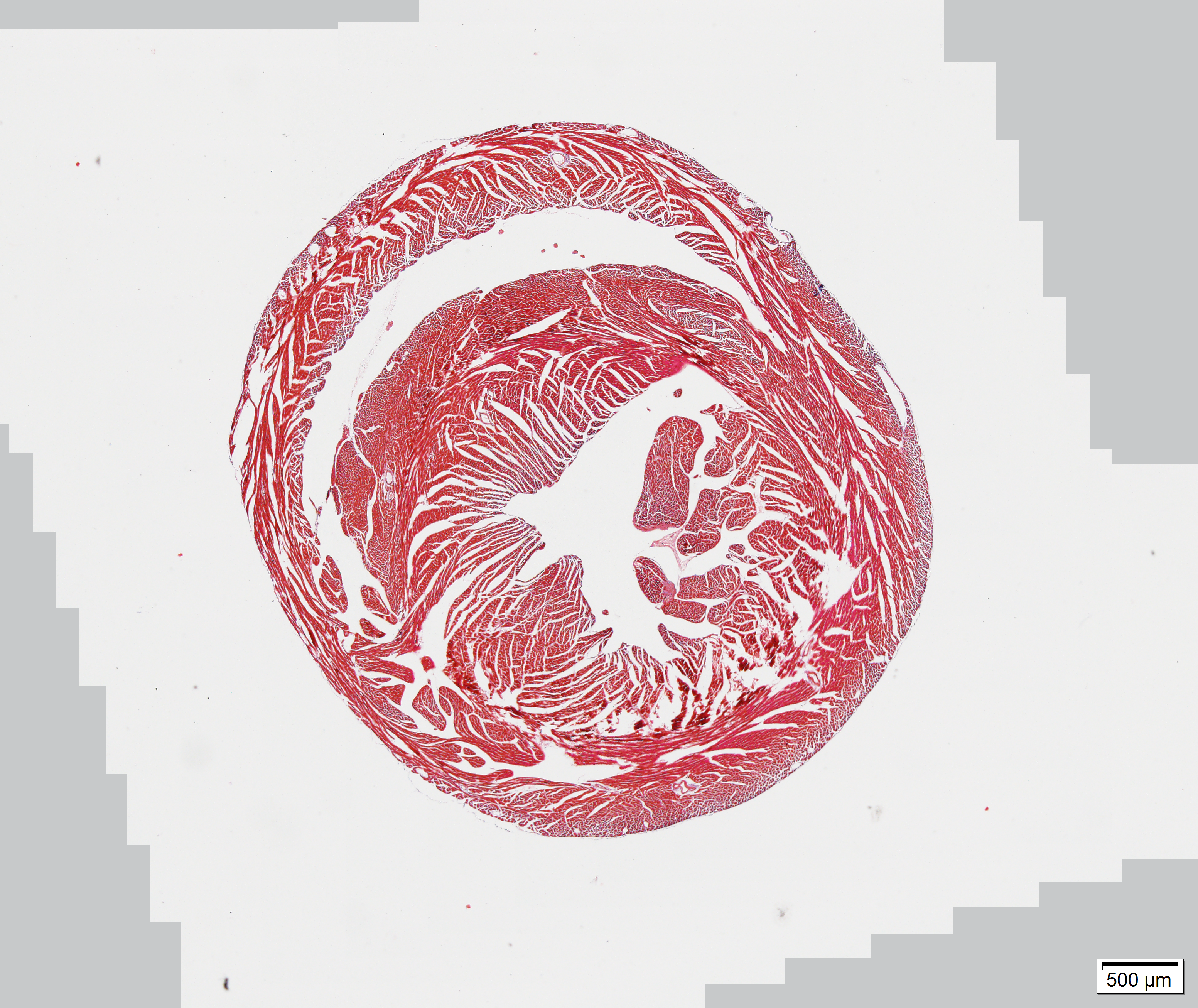

Supplement: Supplementary file 5 — Source data Fig. 2 [file 44321_2025_334_MOESM5_ESM.zip › Figure 2/2F/Cross/AAV9-RBMS1.tif]

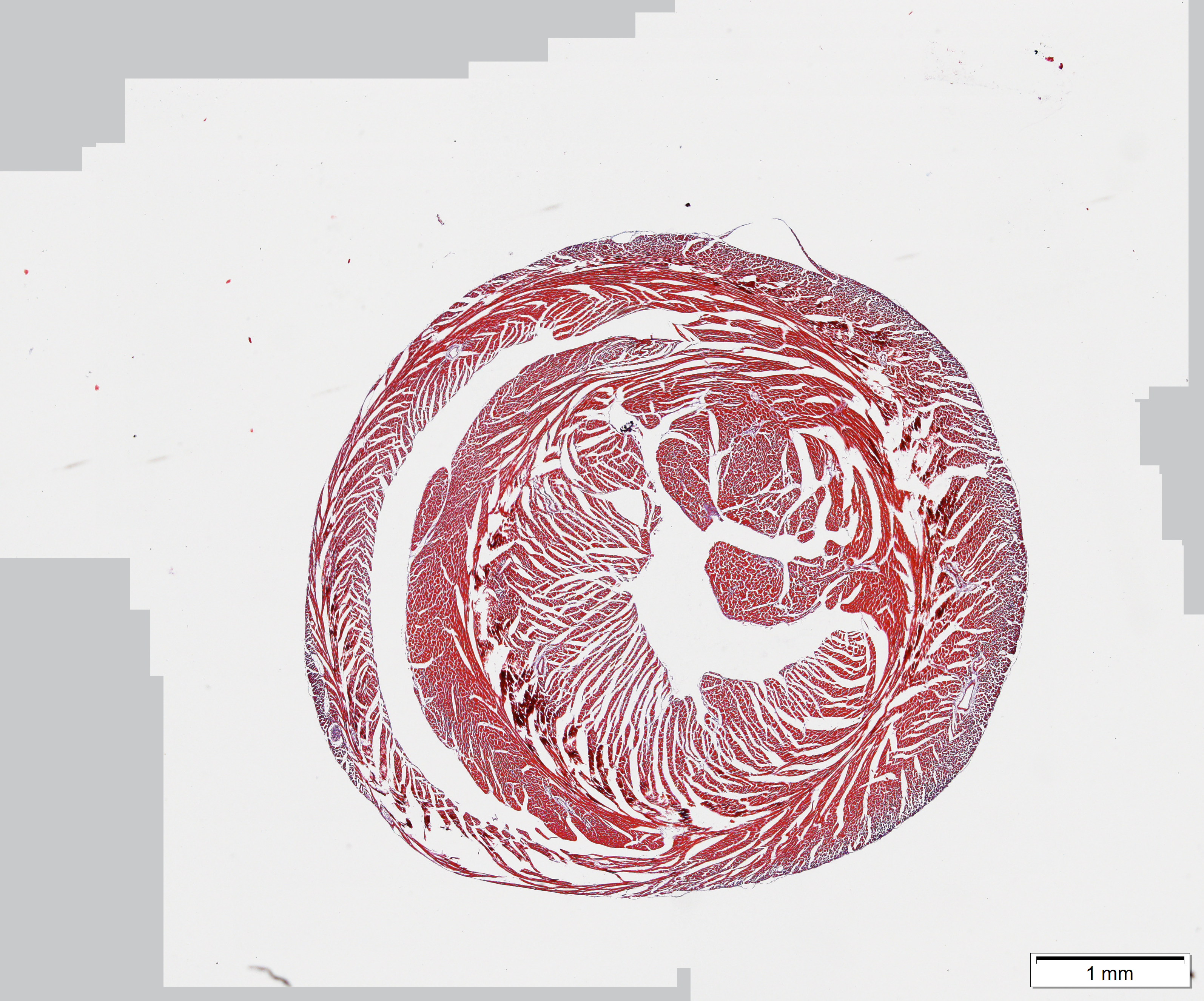

Supplement: Supplementary file 5 — Source data Fig. 2 [file 44321_2025_334_MOESM5_ESM.zip › Figure 2/2F/Cross/AAV9-Vector+TAC.tif]

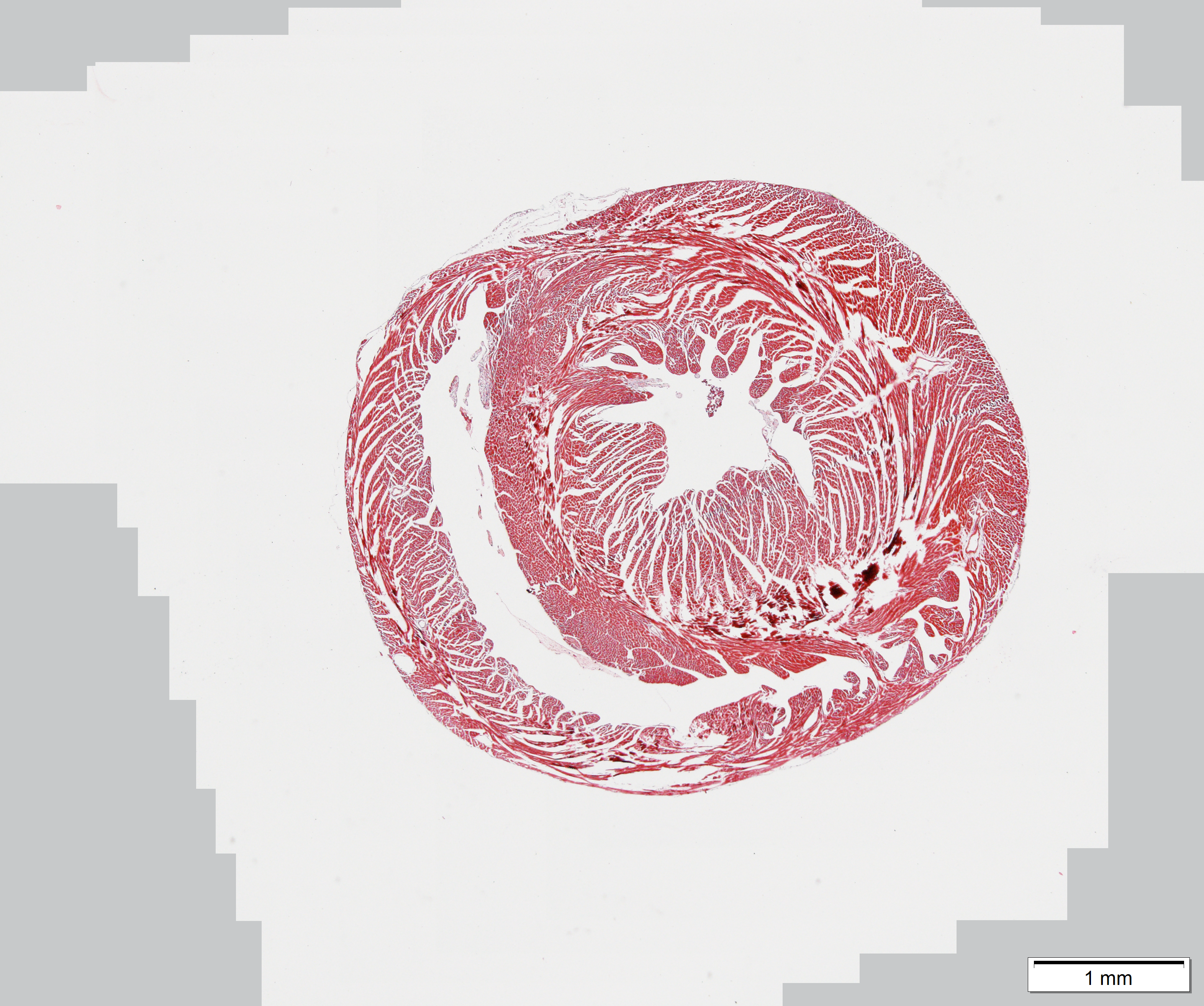

Supplement: Supplementary file 5 — Source data Fig. 2 [file 44321_2025_334_MOESM5_ESM.zip › Figure 2/2F/Cross/AAV9-Vector.tif]

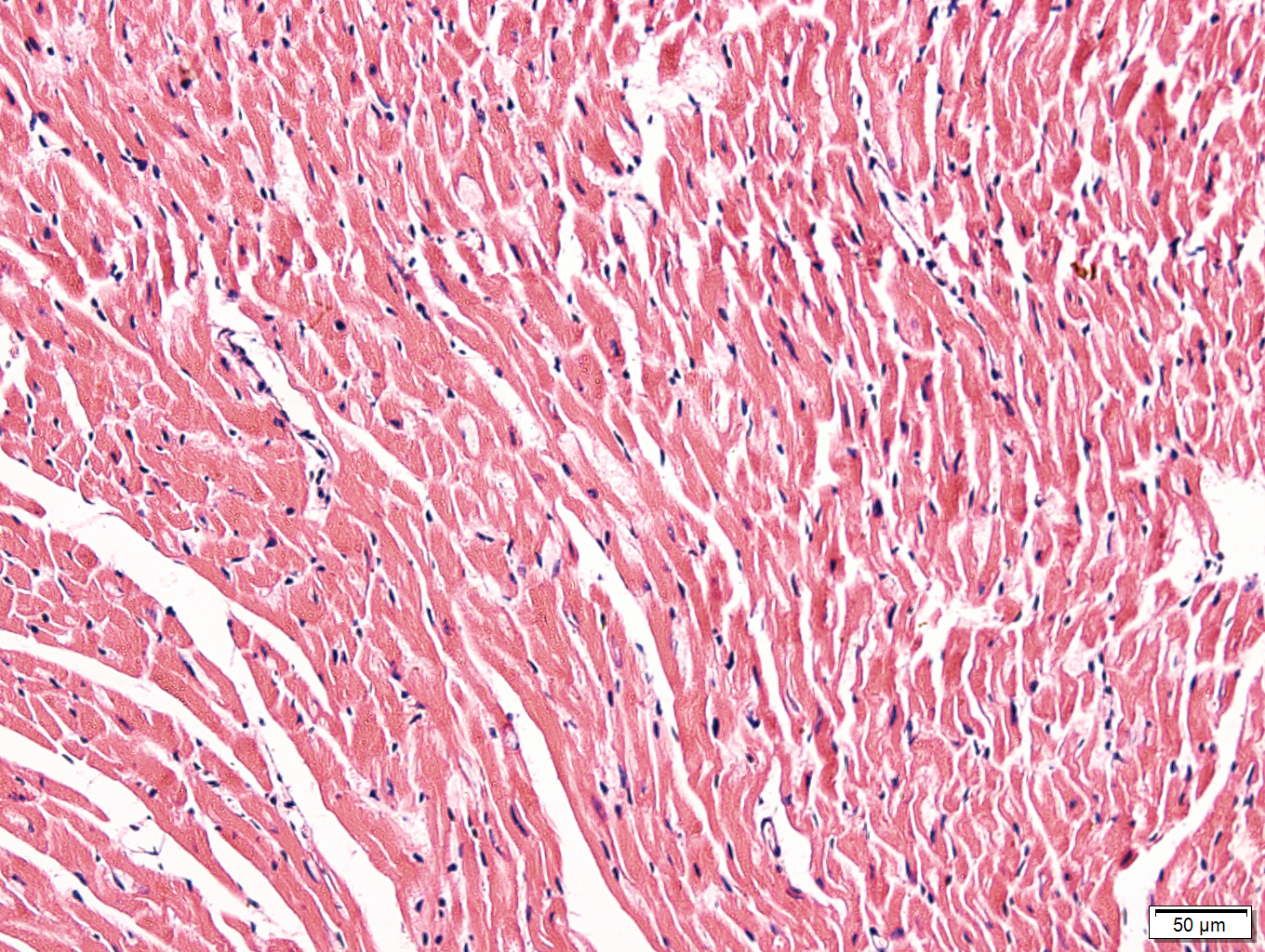

Supplement: Supplementary file 5 — Source data Fig. 2 [file 44321_2025_334_MOESM5_ESM.zip › Figure 2/2F/H&E/AAV9-RBMS1+TAC.tif]

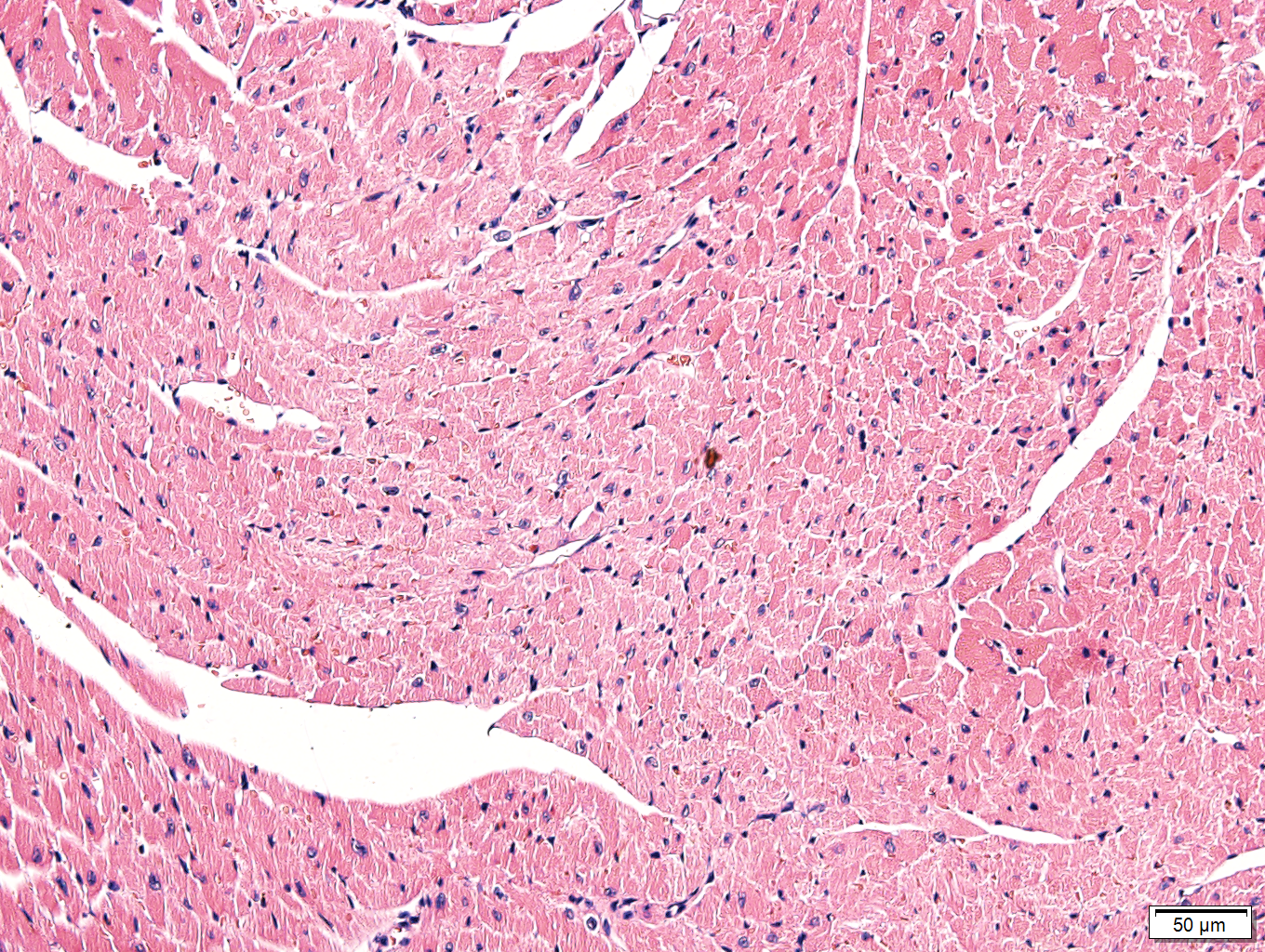

Supplement: Supplementary file 5 — Source data Fig. 2 [file 44321_2025_334_MOESM5_ESM.zip › Figure 2/2F/H&E/AAV9-RBMS1.tif]

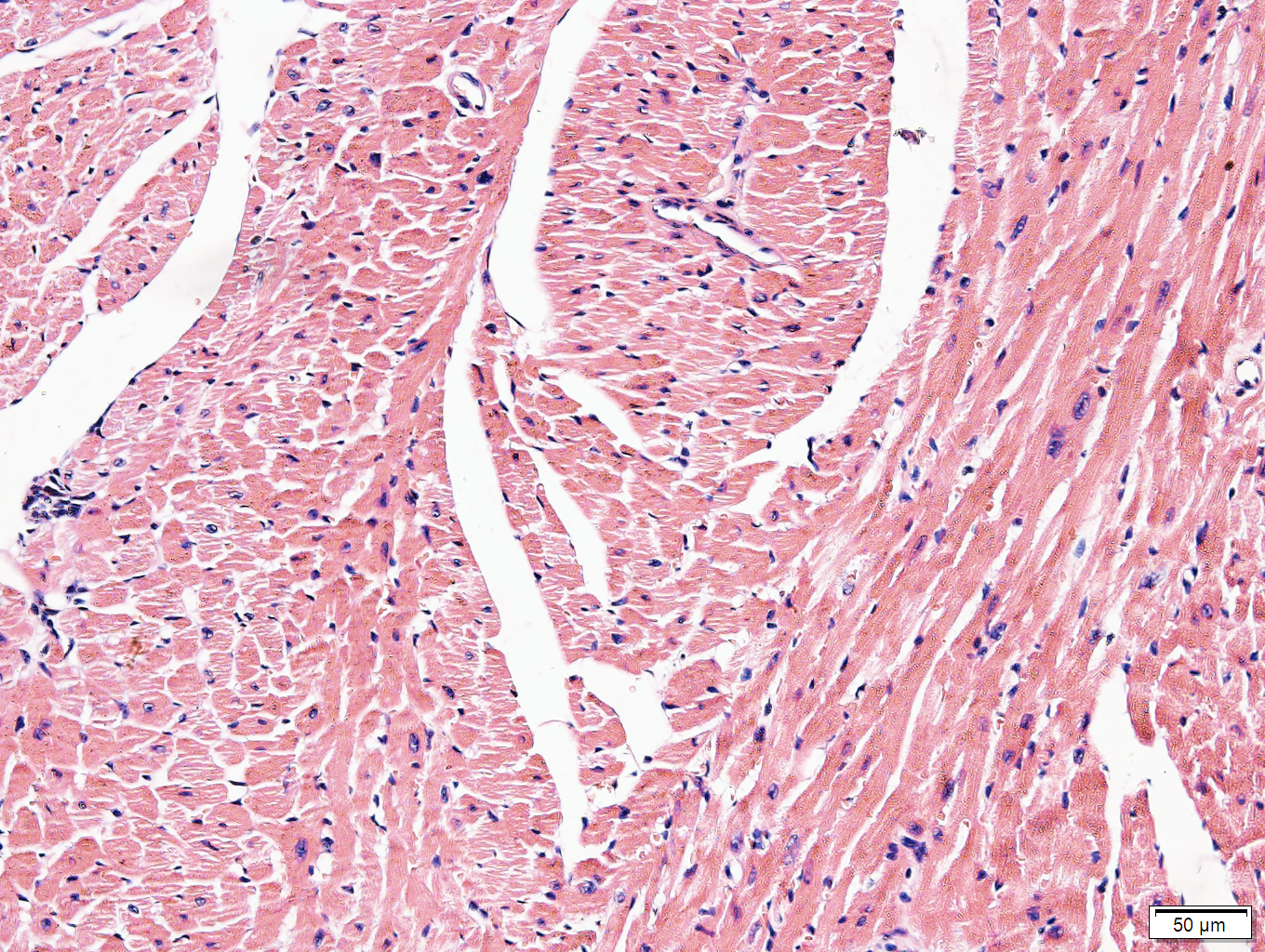

Supplement: Supplementary file 5 — Source data Fig. 2 [file 44321_2025_334_MOESM5_ESM.zip › Figure 2/2F/H&E/AAV9-Vector+TAC.tif]

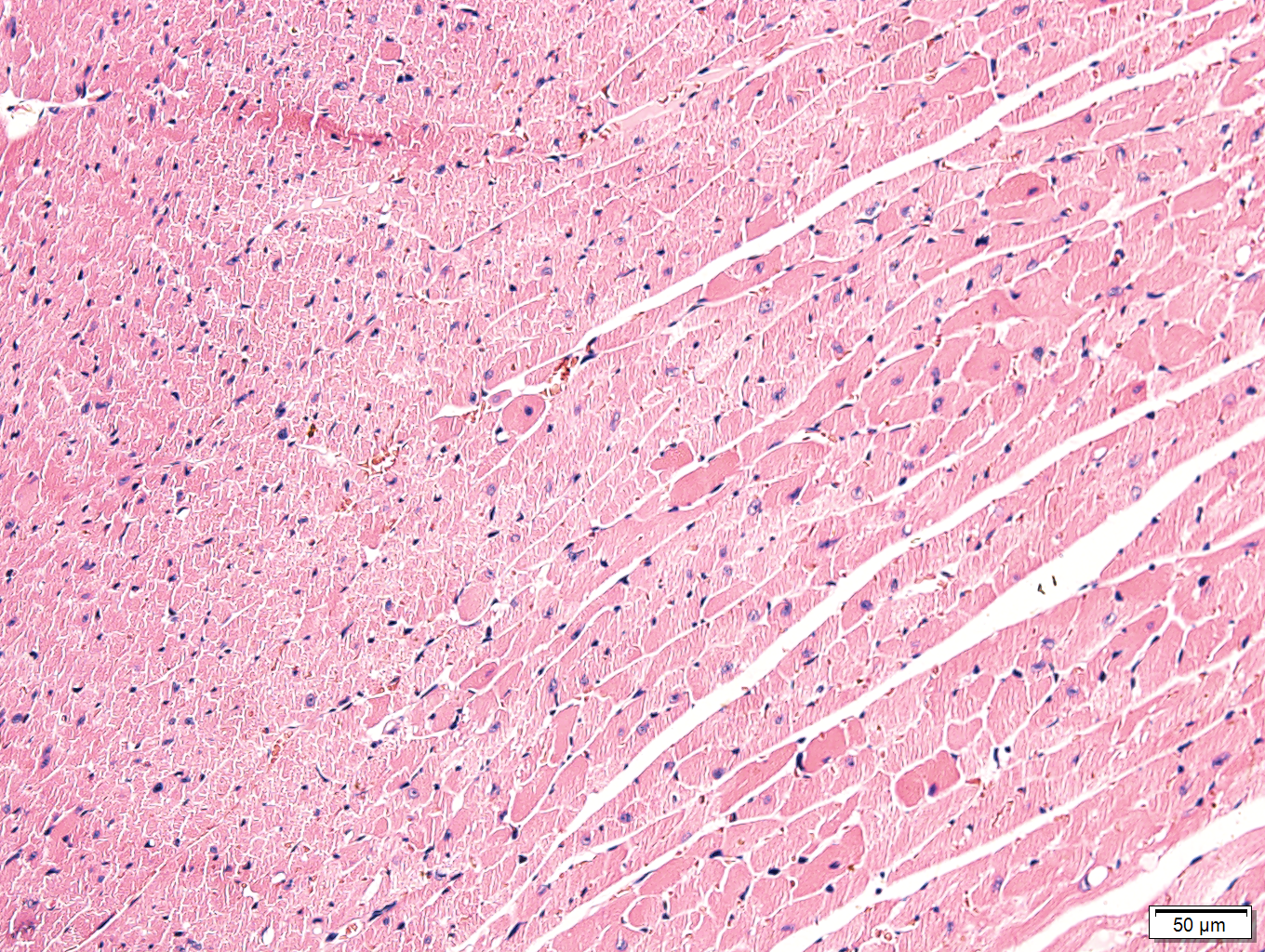

Supplement: Supplementary file 5 — Source data Fig. 2 [file 44321_2025_334_MOESM5_ESM.zip › Figure 2/2F/H&E/AAV9-Vector.tif]

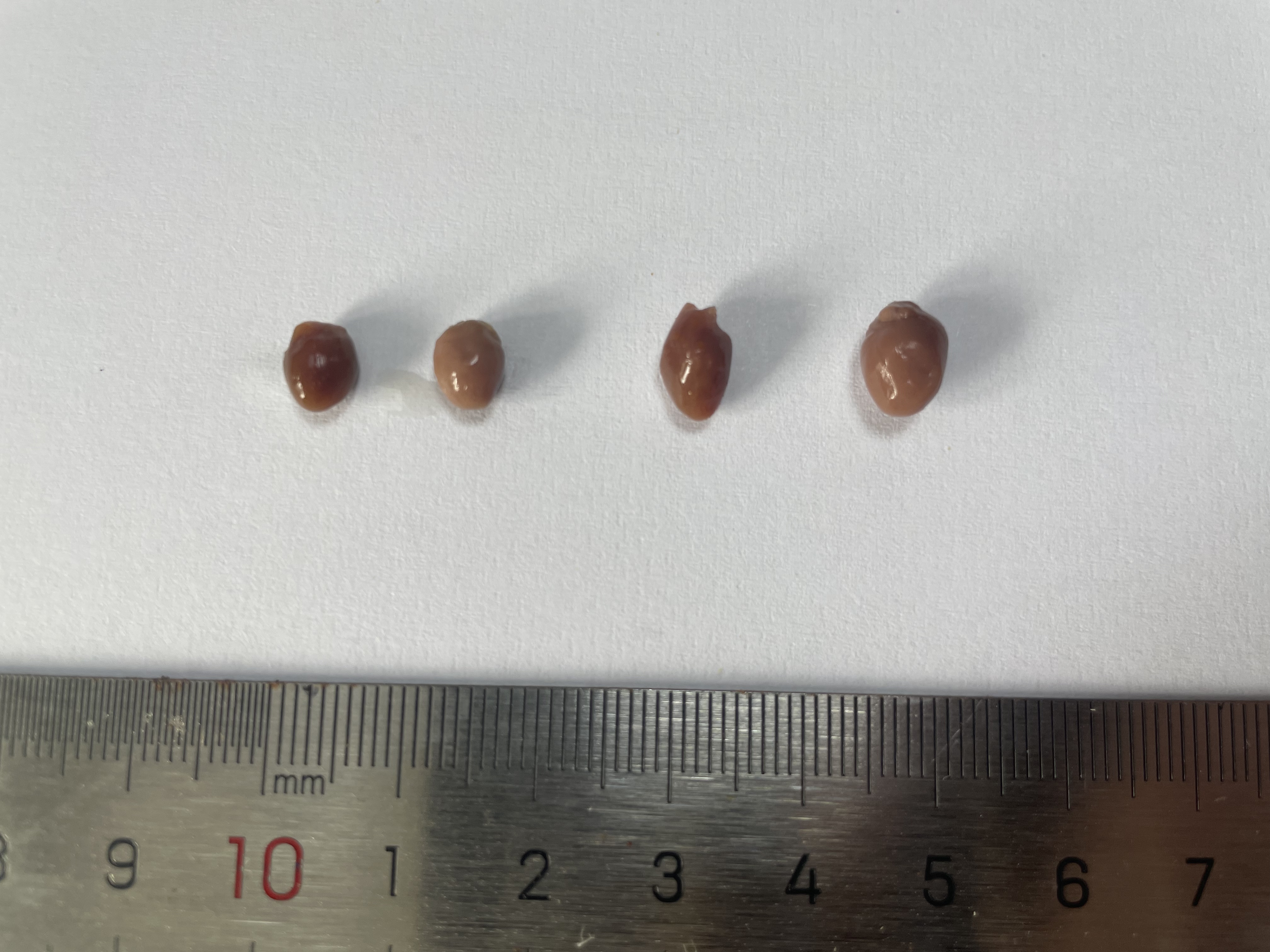

Supplement: Supplementary file 5 — Source data Fig. 2 [file 44321_2025_334_MOESM5_ESM.zip › Figure 2/2F/Heart size/Heart size.jpg]

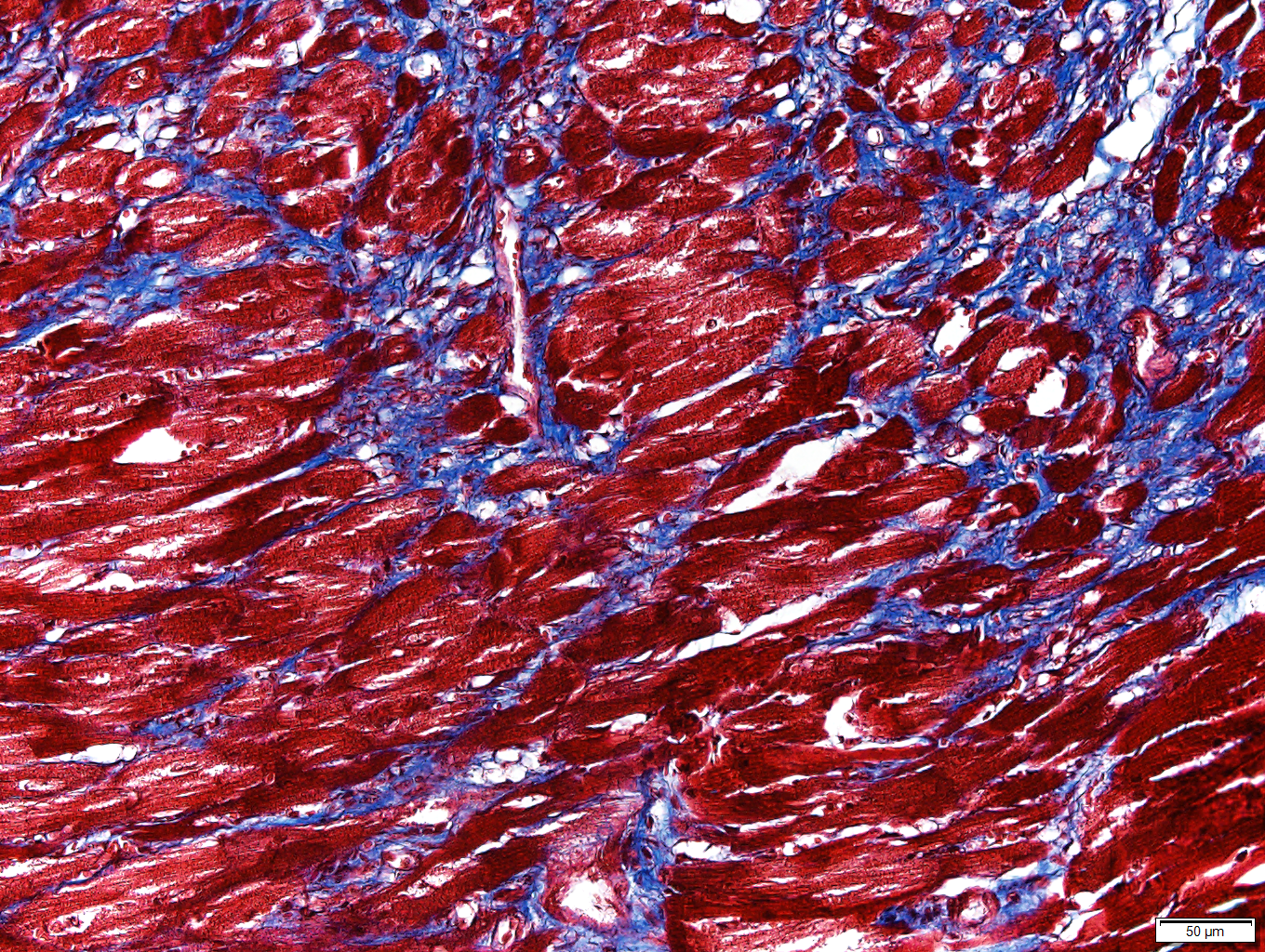

Supplement: Supplementary file 5 — Source data Fig. 2 [file 44321_2025_334_MOESM5_ESM.zip › Figure 2/2F/Interstital/AAV9-RBMS1+TAC.tif]

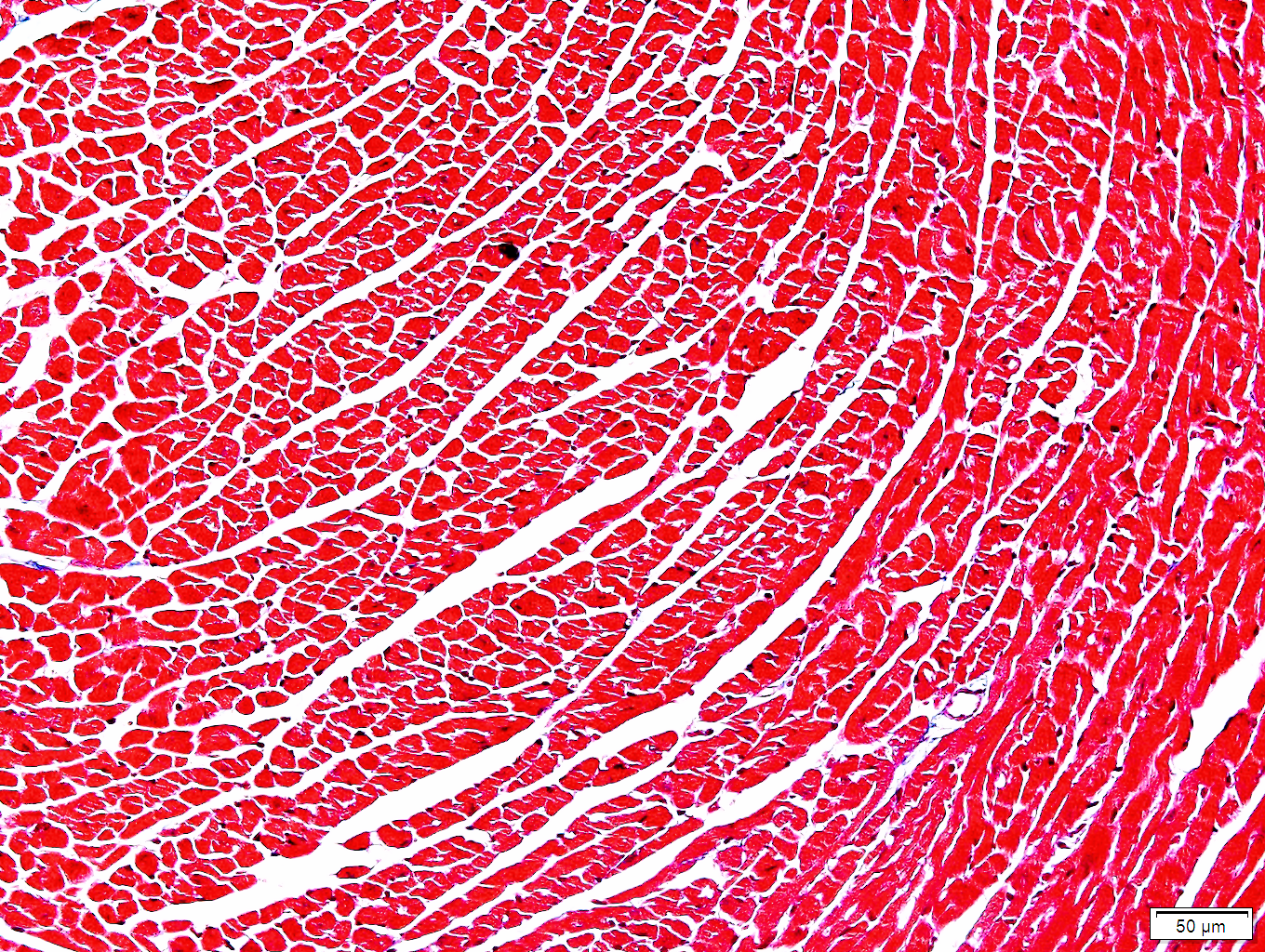

Supplement: Supplementary file 5 — Source data Fig. 2 [file 44321_2025_334_MOESM5_ESM.zip › Figure 2/2F/Interstital/AAV9-RBMS1.tif]

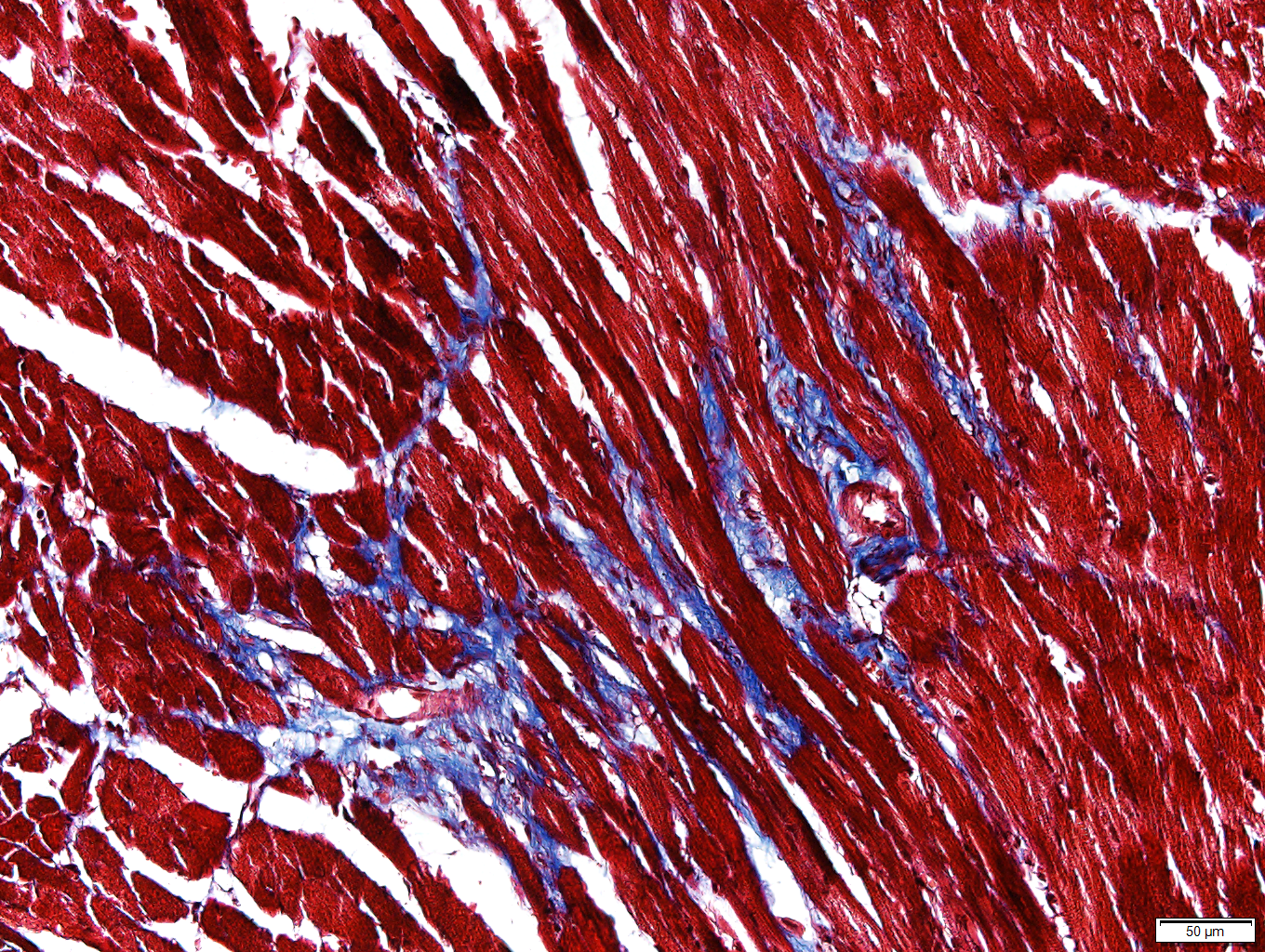

Supplement: Supplementary file 5 — Source data Fig. 2 [file 44321_2025_334_MOESM5_ESM.zip › Figure 2/2F/Interstital/AAV9-Vector+TAC.tif]

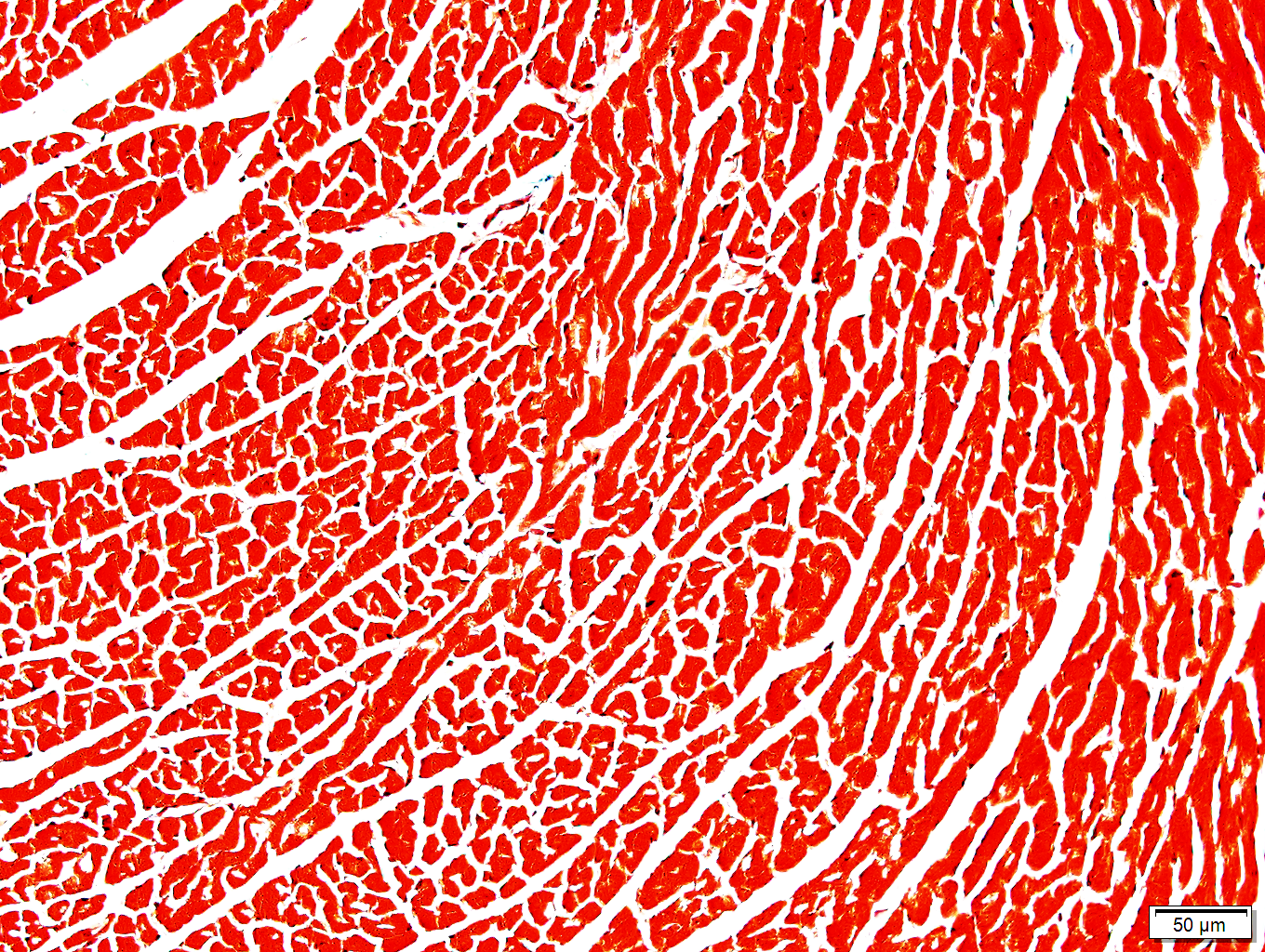

Supplement: Supplementary file 5 — Source data Fig. 2 [file 44321_2025_334_MOESM5_ESM.zip › Figure 2/2F/Interstital/AAV9-Vector.tif]

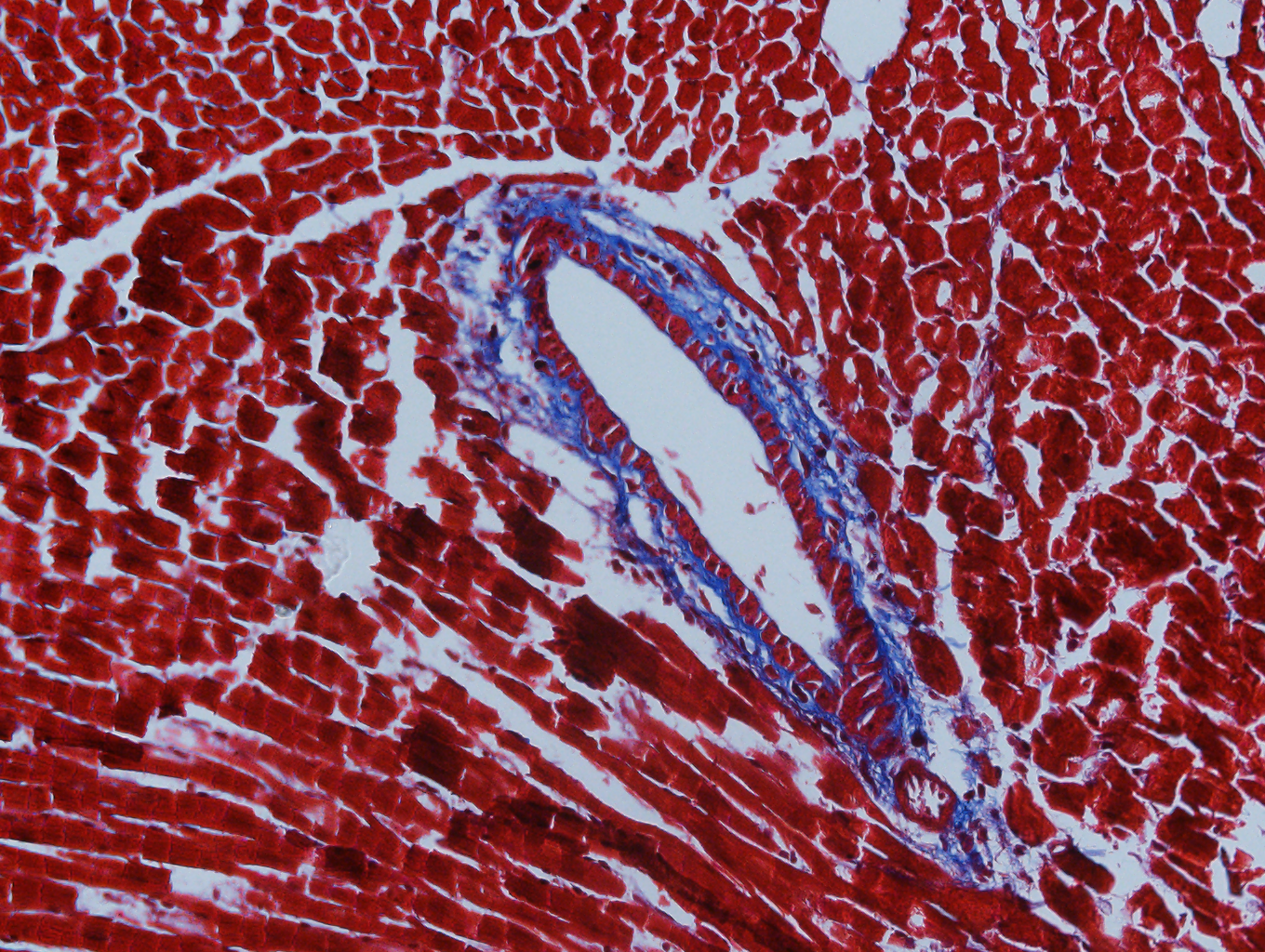

Supplement: Supplementary file 5 — Source data Fig. 2 [file 44321_2025_334_MOESM5_ESM.zip › Figure 2/2F/Perivascular/AAV9-RBMS1+TAC.tif]

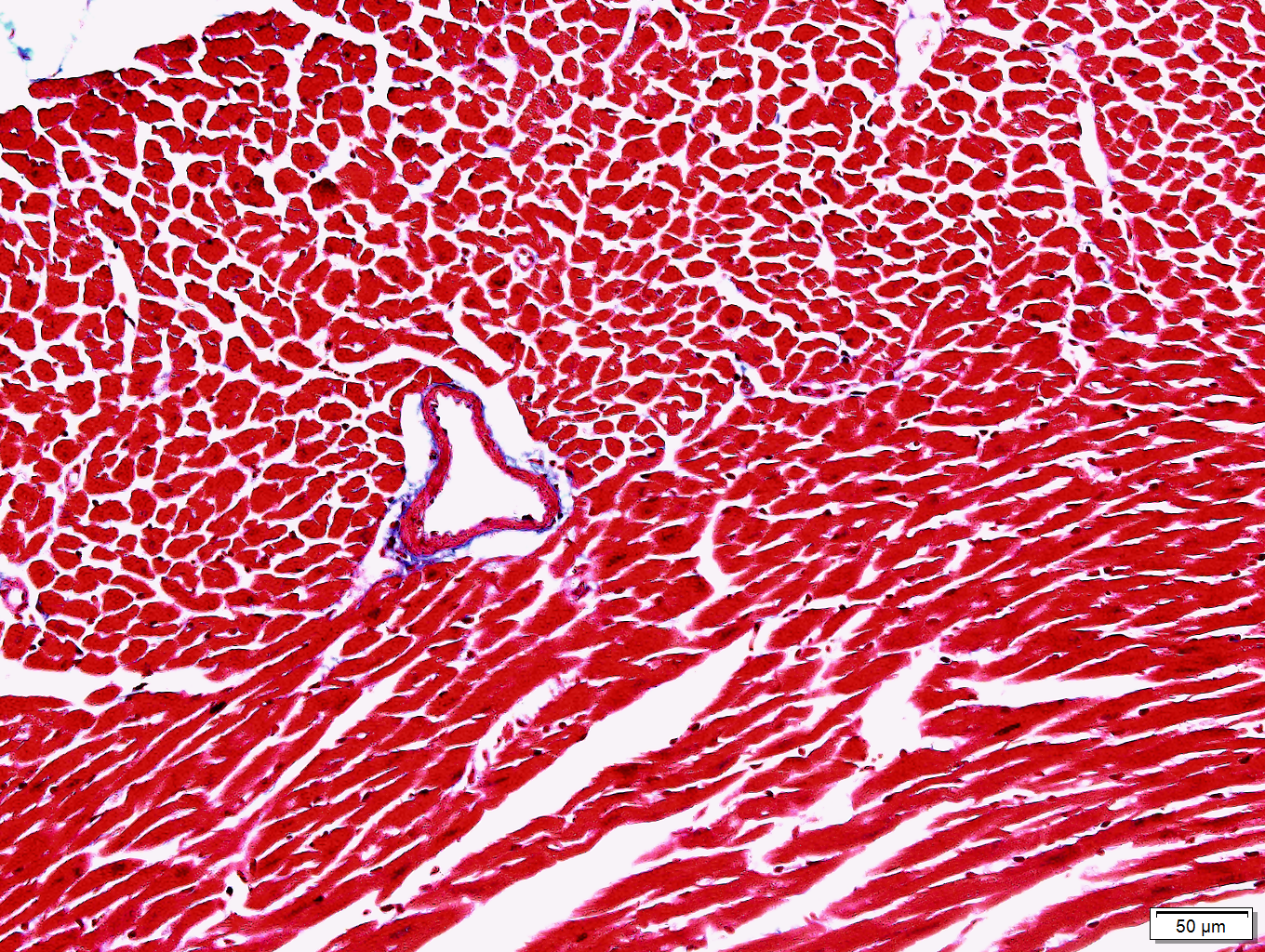

Supplement: Supplementary file 5 — Source data Fig. 2 [file 44321_2025_334_MOESM5_ESM.zip › Figure 2/2F/Perivascular/AAV9-RBMS1.tif]

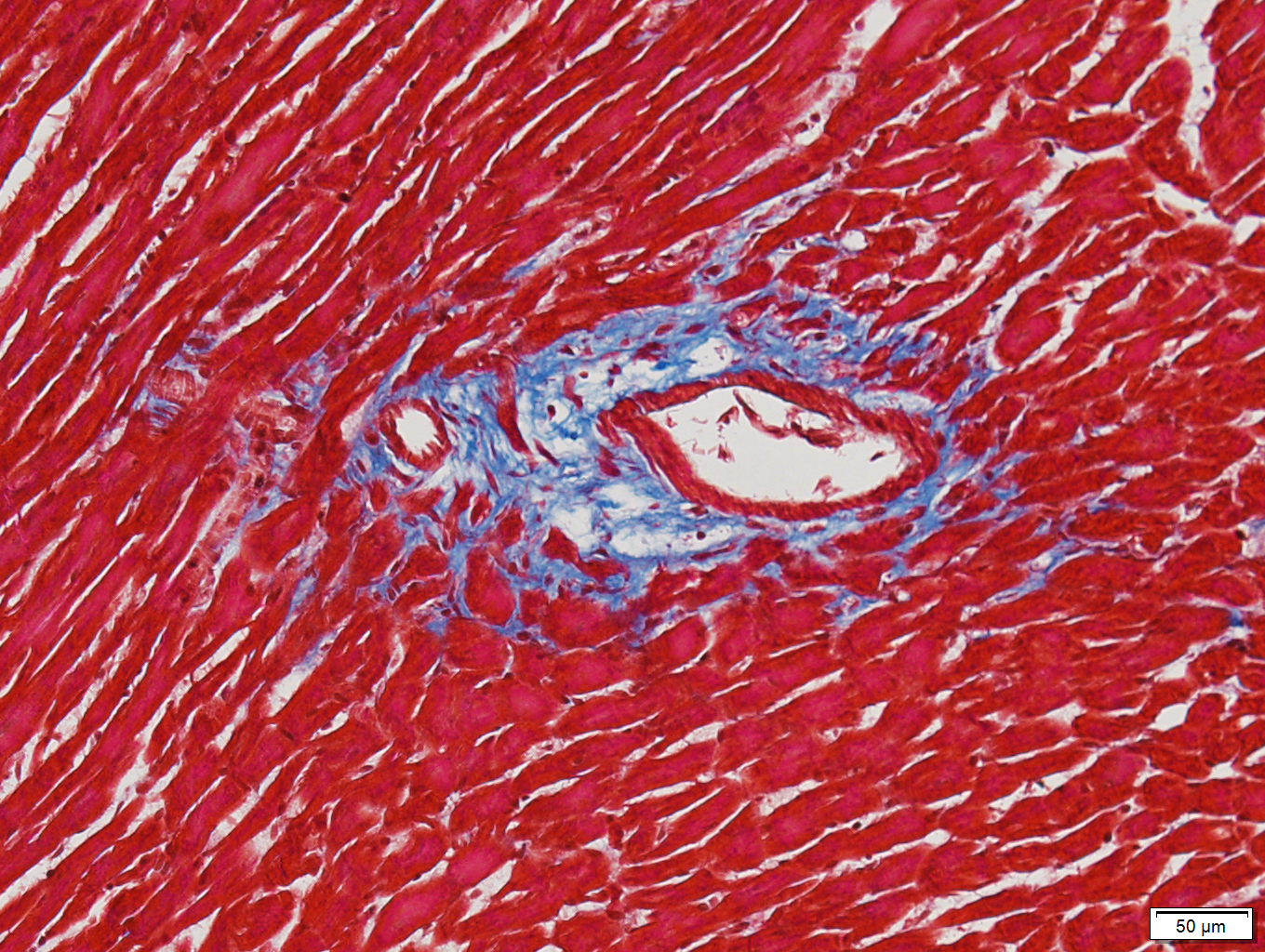

Supplement: Supplementary file 5 — Source data Fig. 2 [file 44321_2025_334_MOESM5_ESM.zip › Figure 2/2F/Perivascular/AAV9-Vector+TAC.tif]

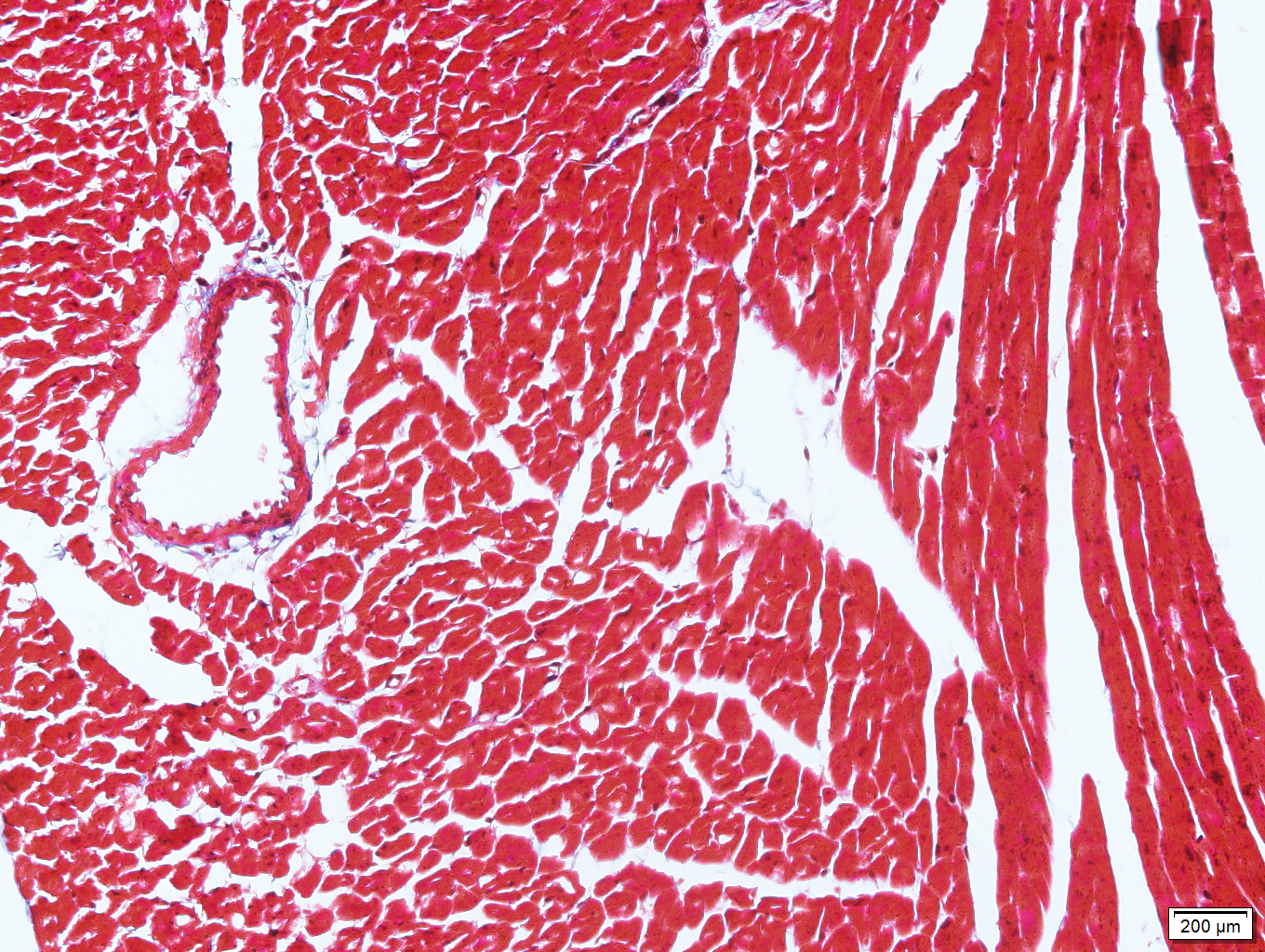

Supplement: Supplementary file 5 — Source data Fig. 2 [file 44321_2025_334_MOESM5_ESM.zip › Figure 2/2F/Perivascular/AAV9-Vector.tif]

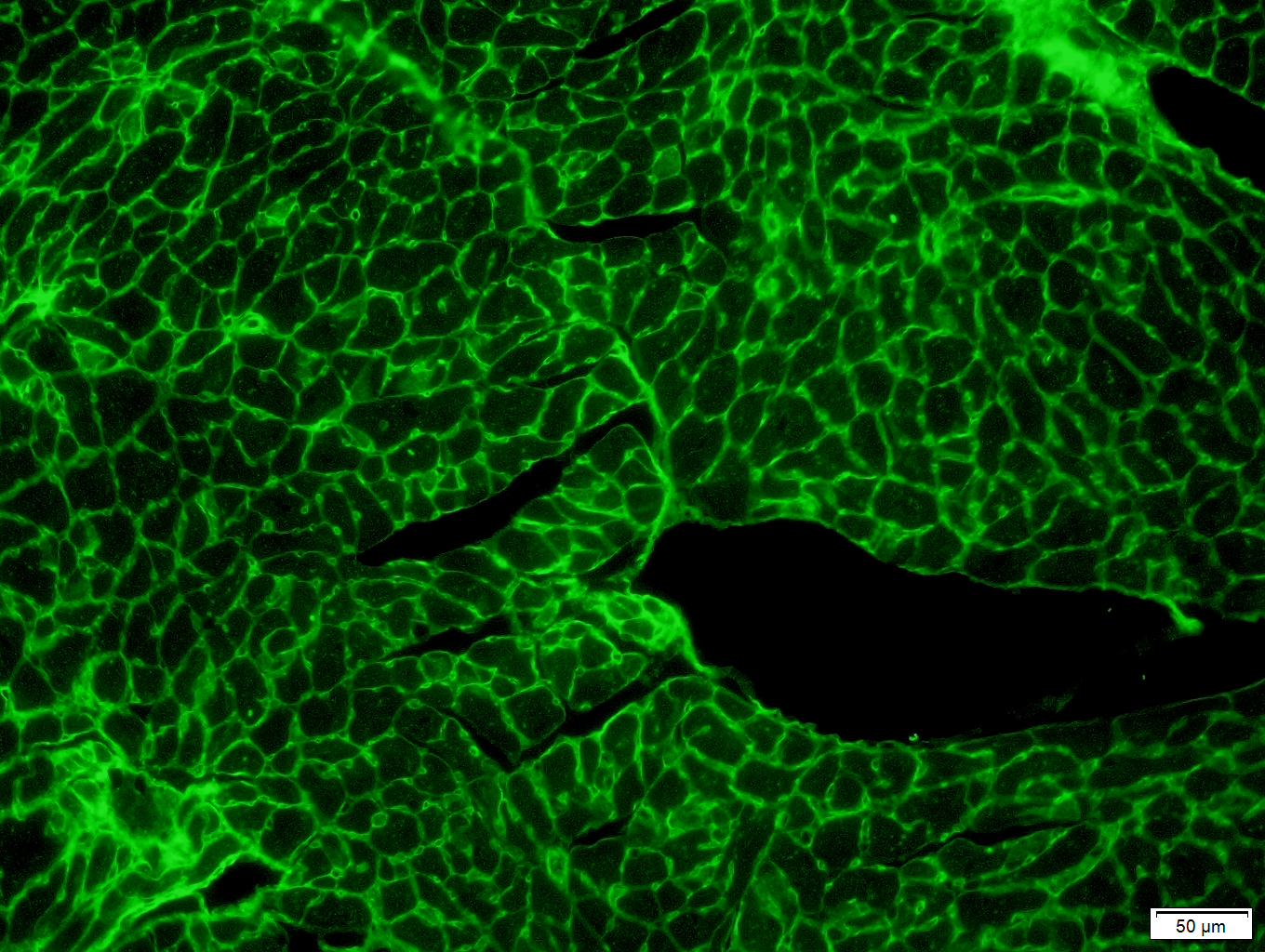

Supplement: Supplementary file 5 — Source data Fig. 2 [file 44321_2025_334_MOESM5_ESM.zip › Figure 2/2F/WGA/AAV9-RBMS1+TAC.tif]

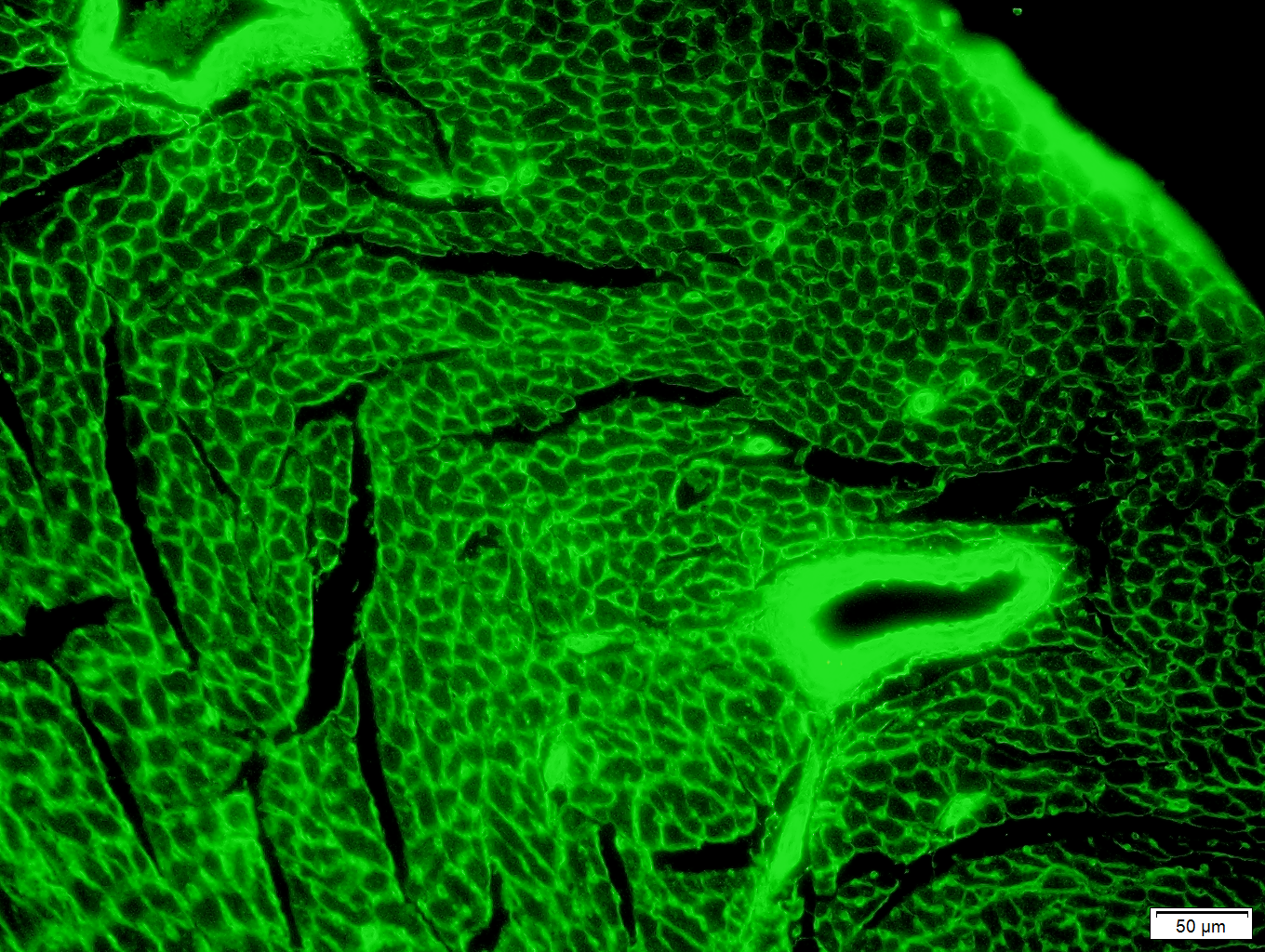

Supplement: Supplementary file 5 — Source data Fig. 2 [file 44321_2025_334_MOESM5_ESM.zip › Figure 2/2F/WGA/AAV9-RBMS1.tif]

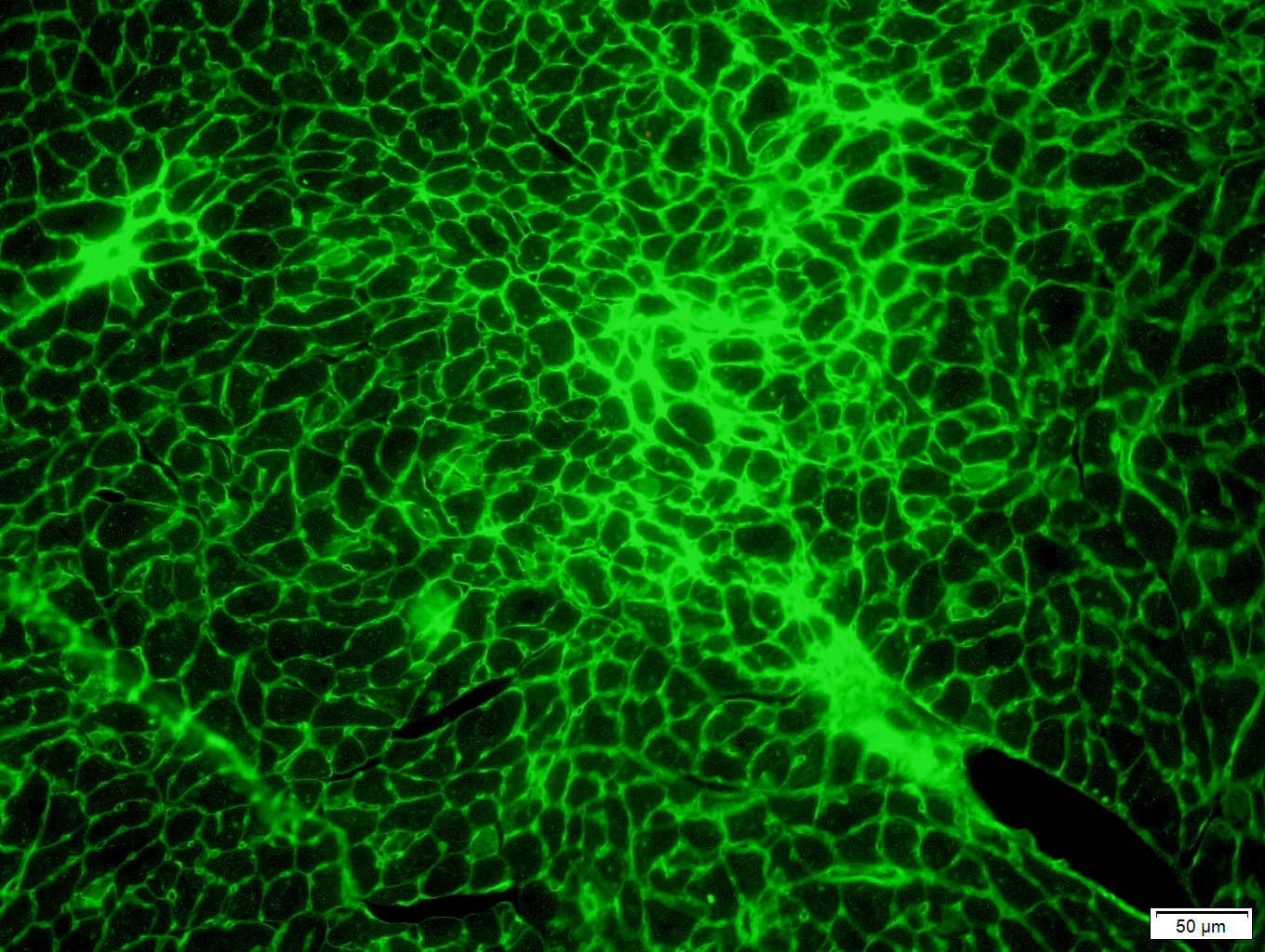

Supplement: Supplementary file 5 — Source data Fig. 2 [file 44321_2025_334_MOESM5_ESM.zip › Figure 2/2F/WGA/AAV9-Vector+TAC.tif]

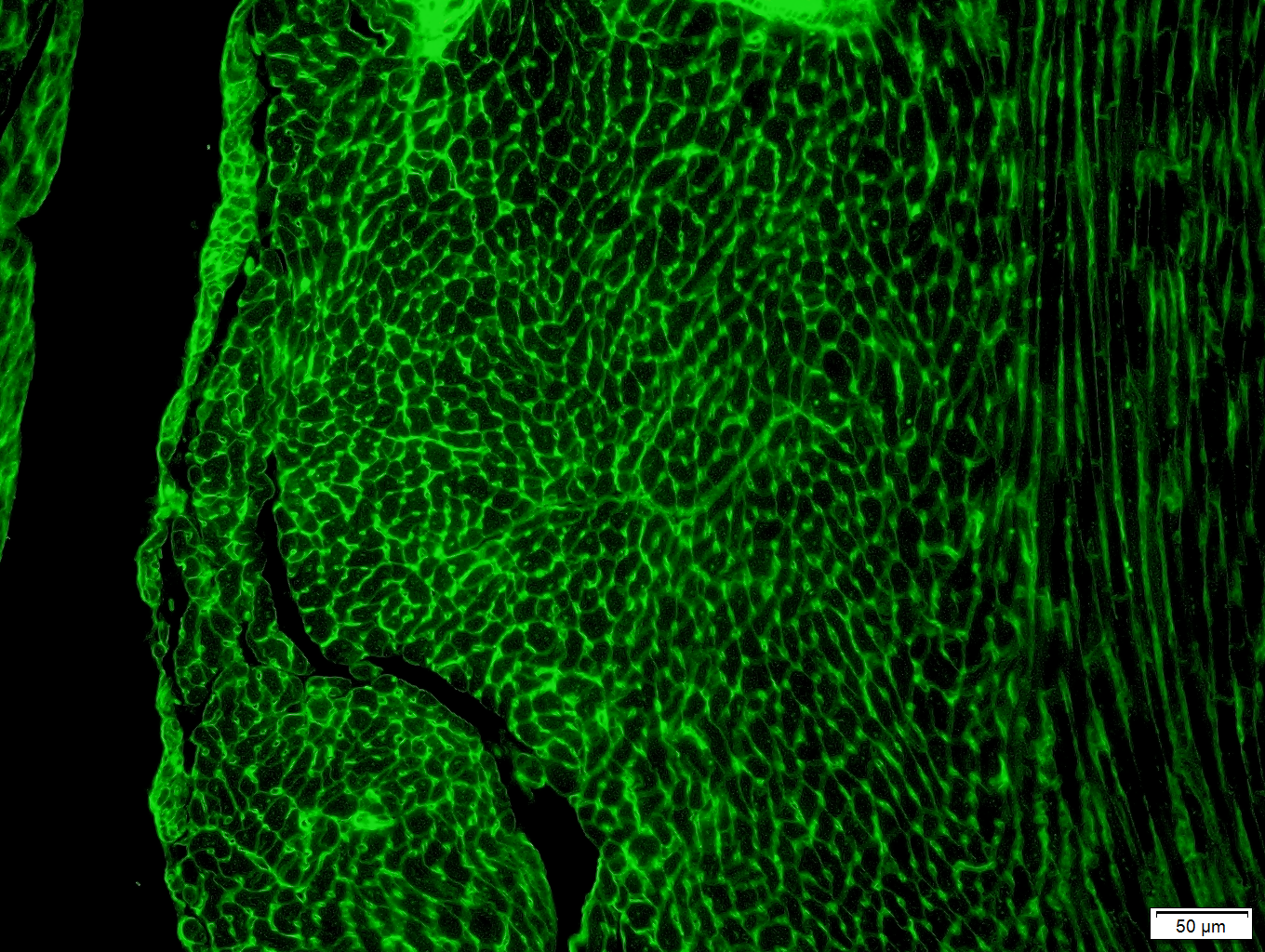

Supplement: Supplementary file 5 — Source data Fig. 2 [file 44321_2025_334_MOESM5_ESM.zip › Figure 2/2F/WGA/AAV9-Vector.tif]

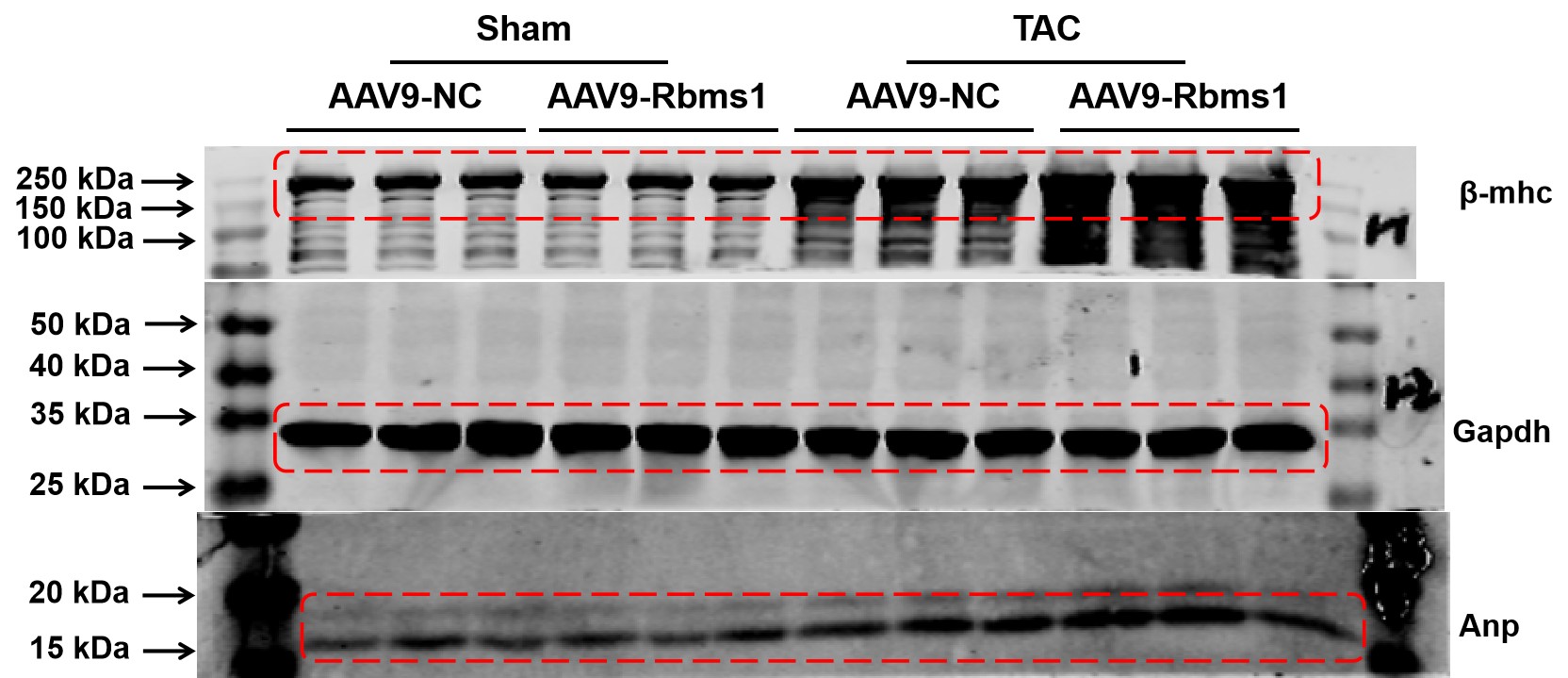

Supplement: Supplementary file 5 — Source data Fig. 2 [file 44321_2025_334_MOESM5_ESM.zip › Figure 2/2J/2J.jpg]

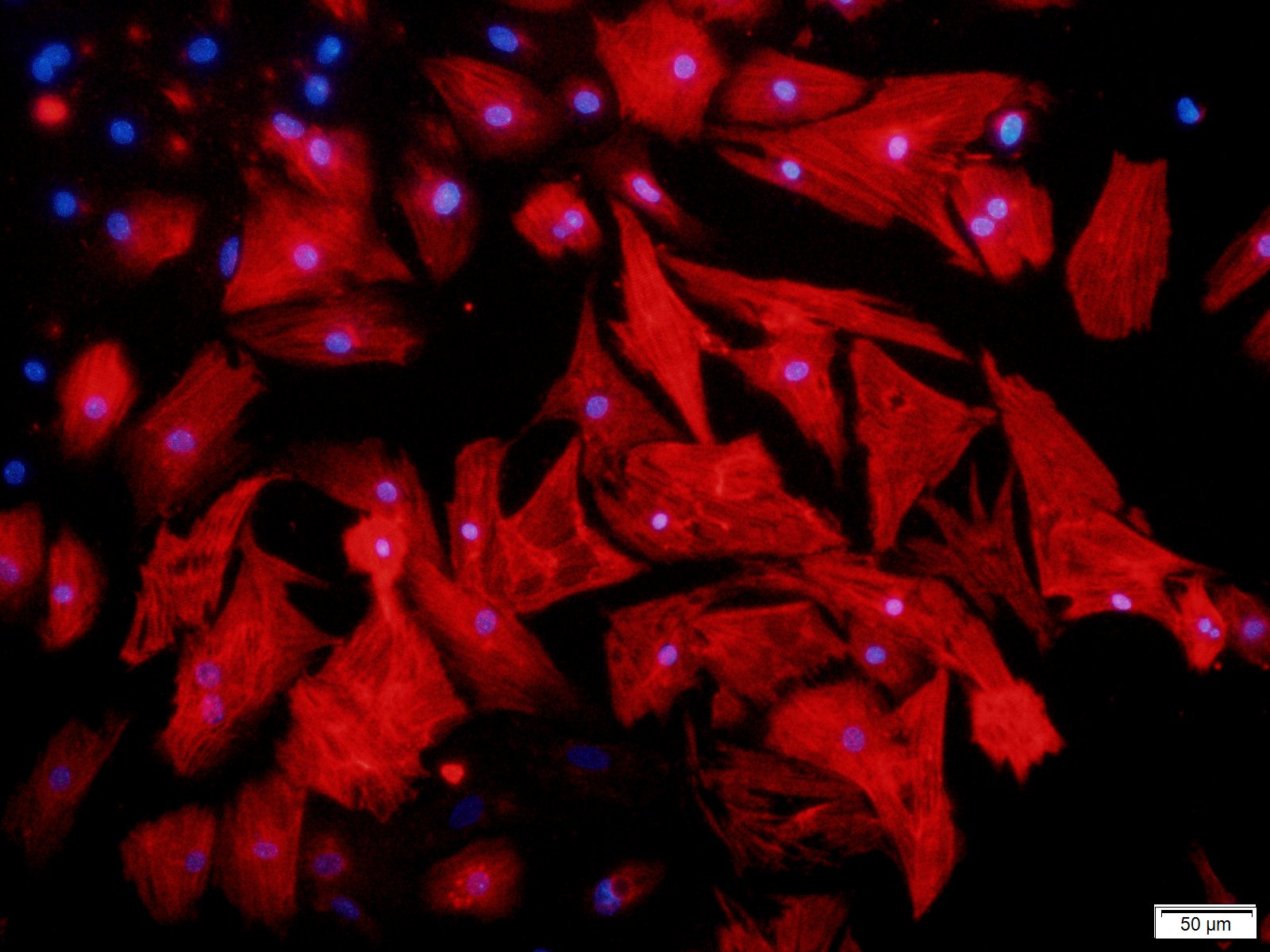

Supplement: Supplementary file 5 — Source data Fig. 2 [file 44321_2025_334_MOESM5_ESM.zip › Figure 2/2M/Ad-NC+Ang II.jpg]

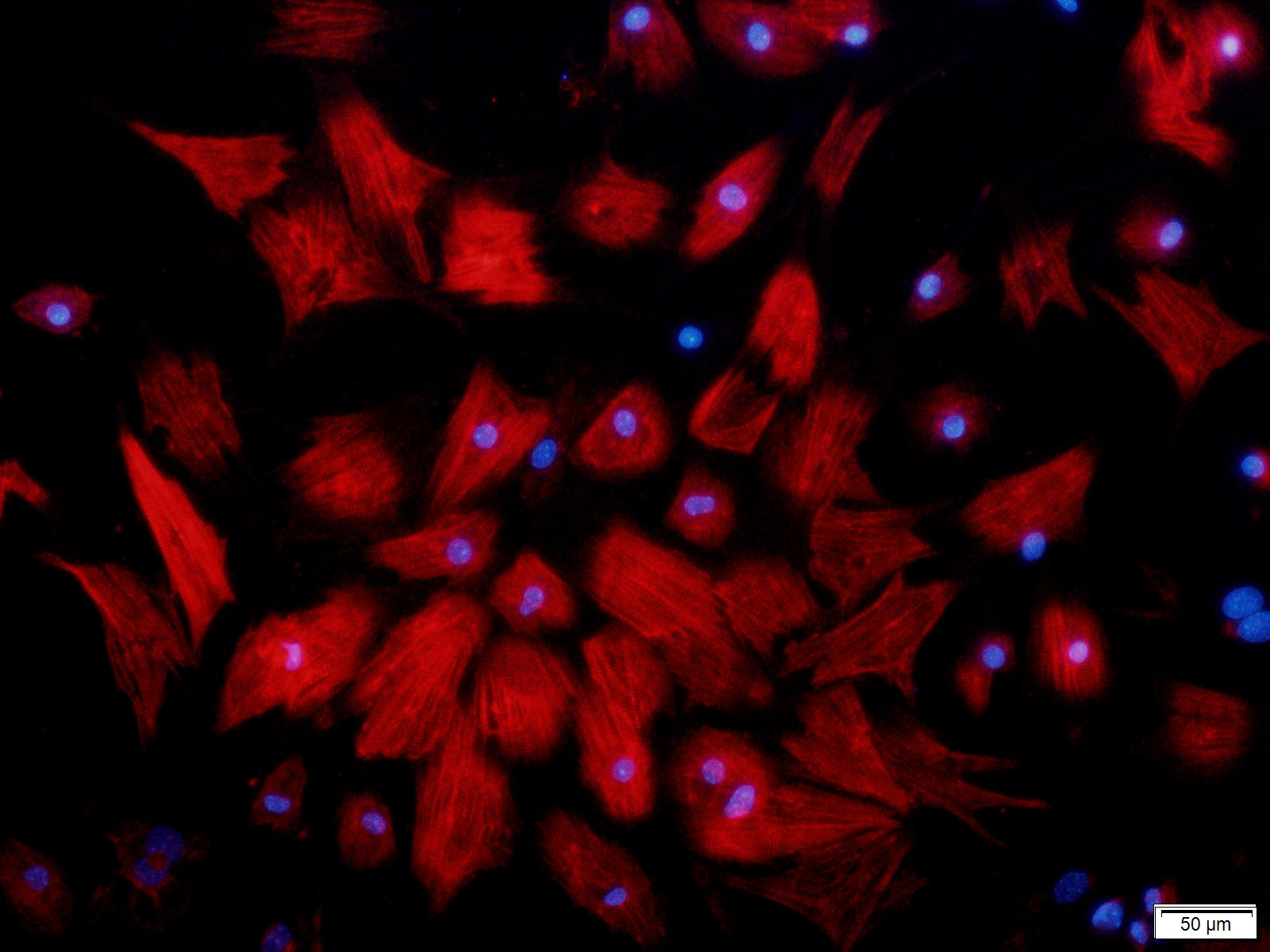

Supplement: Supplementary file 5 — Source data Fig. 2 [file 44321_2025_334_MOESM5_ESM.zip › Figure 2/2M/Ad-NC.jpg]

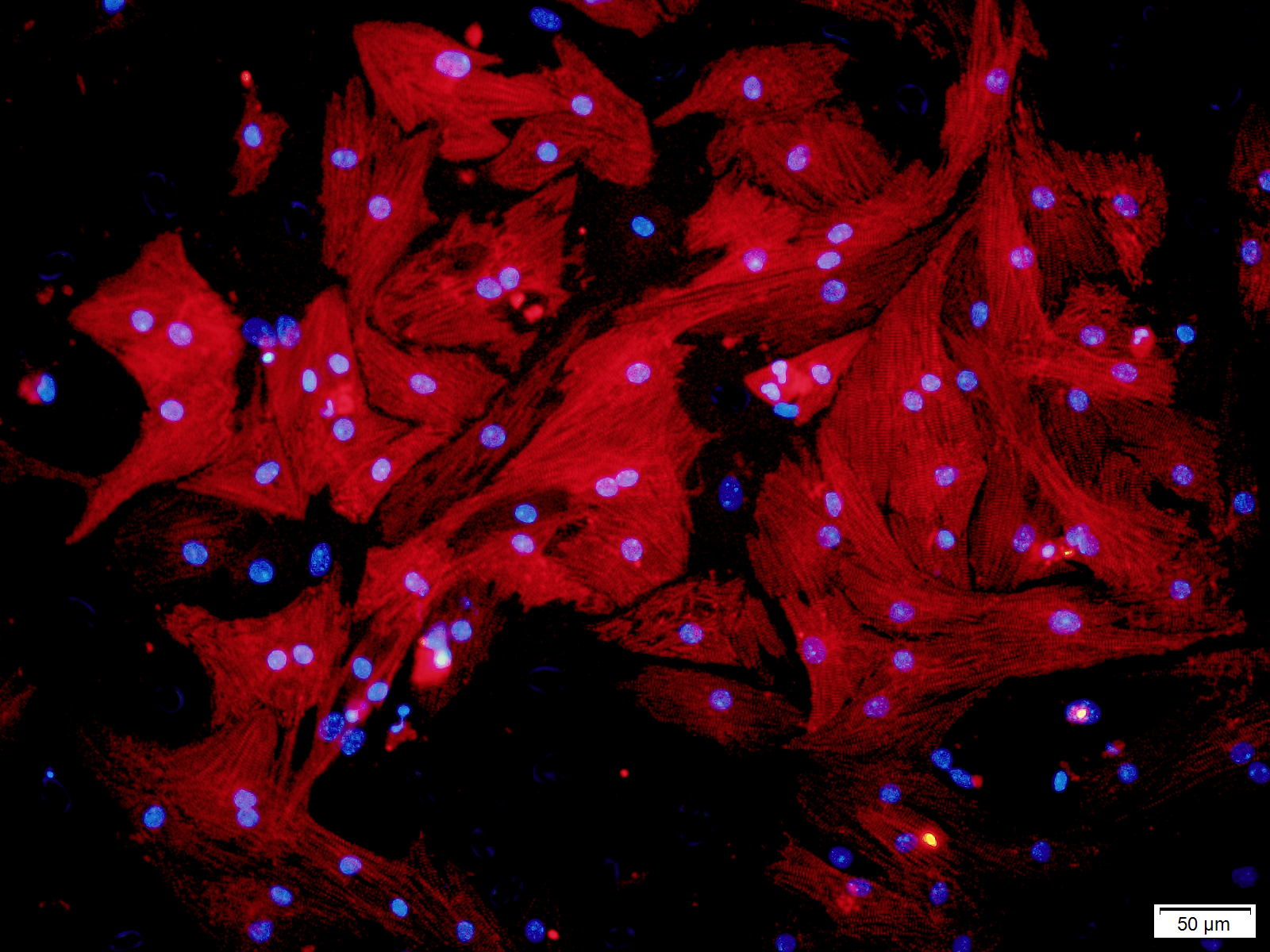

Supplement: Supplementary file 5 — Source data Fig. 2 [file 44321_2025_334_MOESM5_ESM.zip › Figure 2/2M/Ad-RBMS1+Ang II.tif]

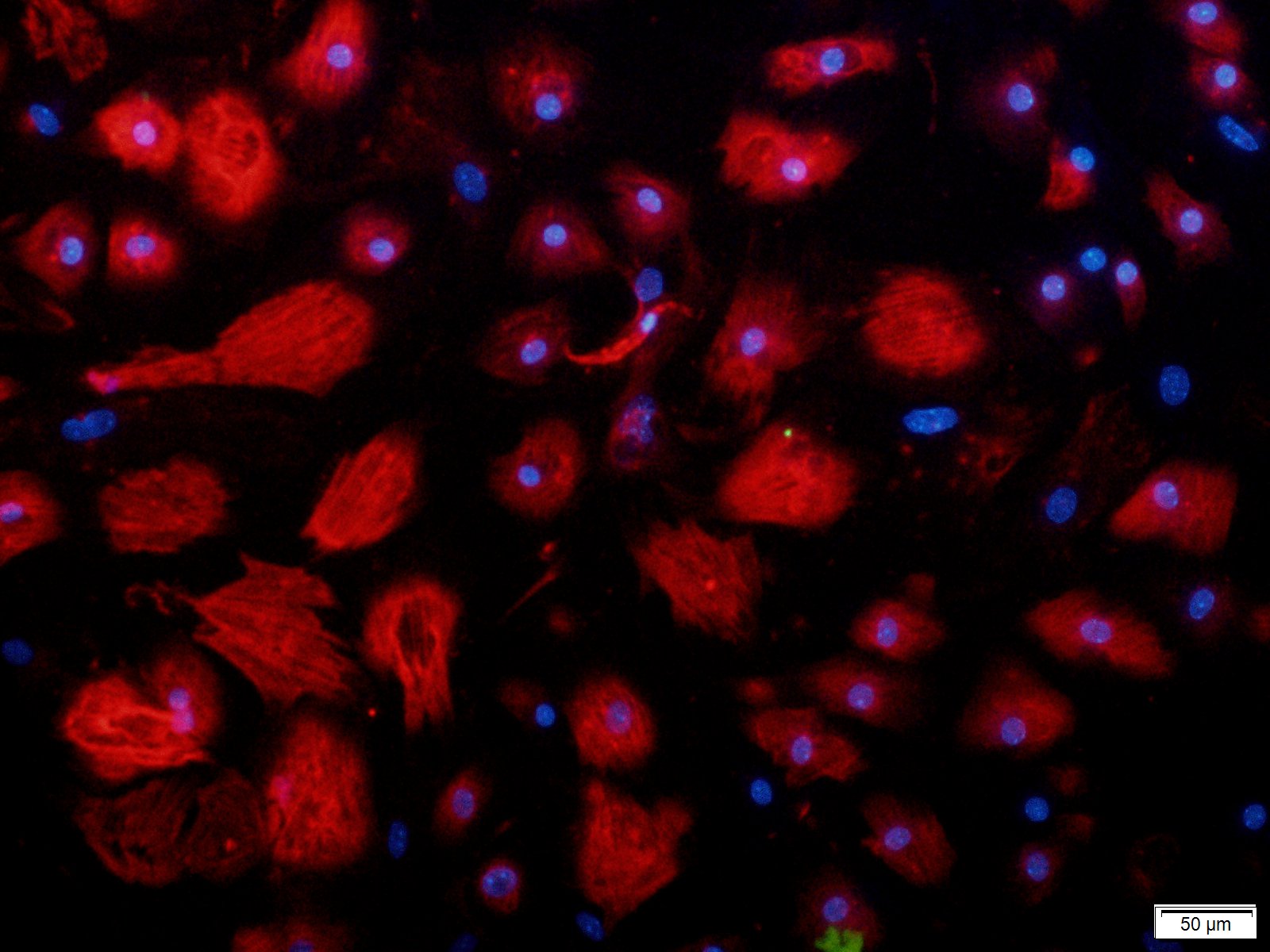

Supplement: Supplementary file 5 — Source data Fig. 2 [file 44321_2025_334_MOESM5_ESM.zip › Figure 2/2M/Ad-RBMS1.jpg]

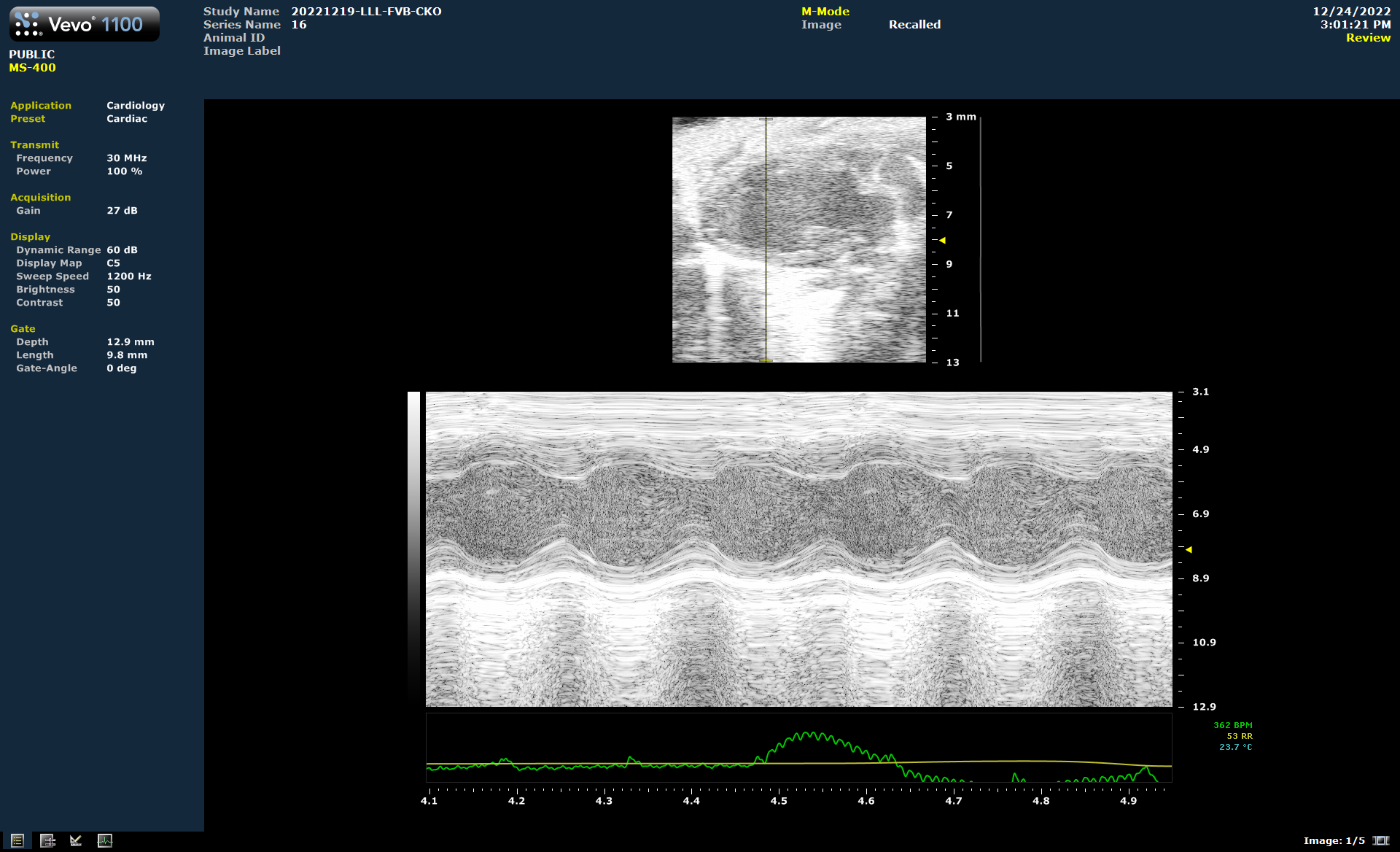

Supplement: Supplementary file 6 — Source data Fig. 3 [file 44321_2025_334_MOESM6_ESM.zip › Figure 3/3B/B Mode/RBMS1-cko+Sham.tif]

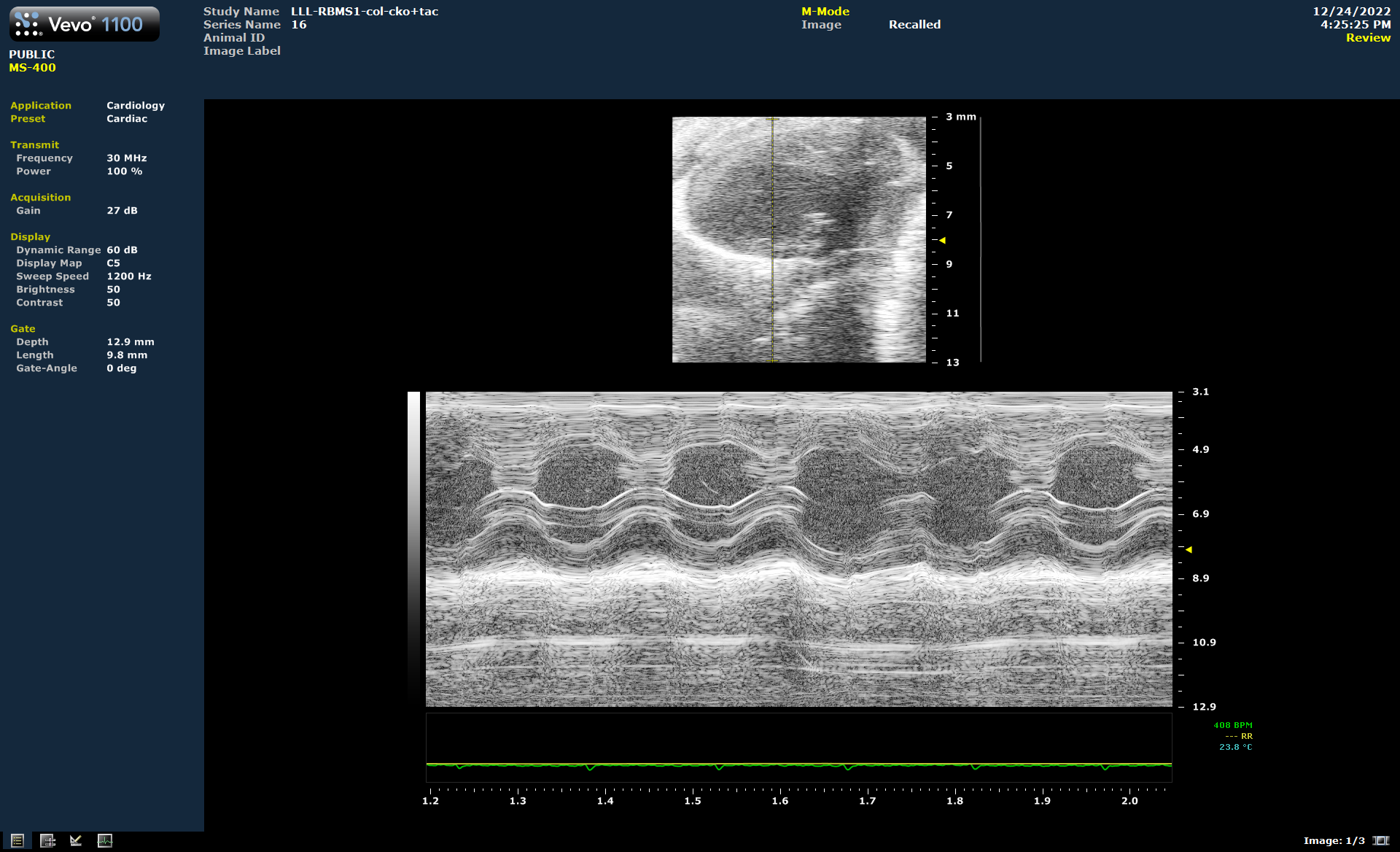

Supplement: Supplementary file 6 — Source data Fig. 3 [file 44321_2025_334_MOESM6_ESM.zip › Figure 3/3B/B Mode/RBMS1-cko+TAC.tif]

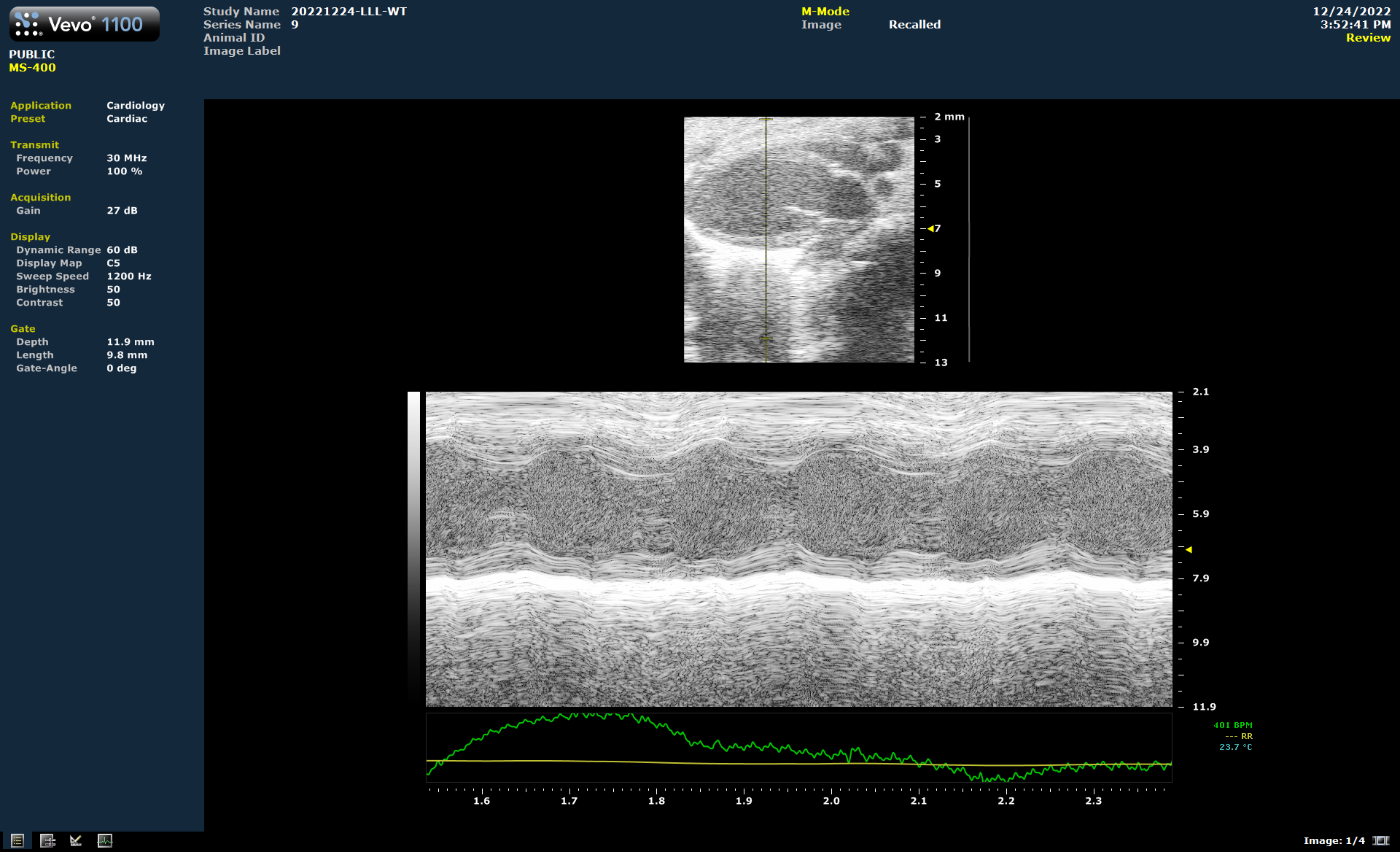

Supplement: Supplementary file 6 — Source data Fig. 3 [file 44321_2025_334_MOESM6_ESM.zip › Figure 3/3B/B Mode/RBMS1-flox+Sham.tif]

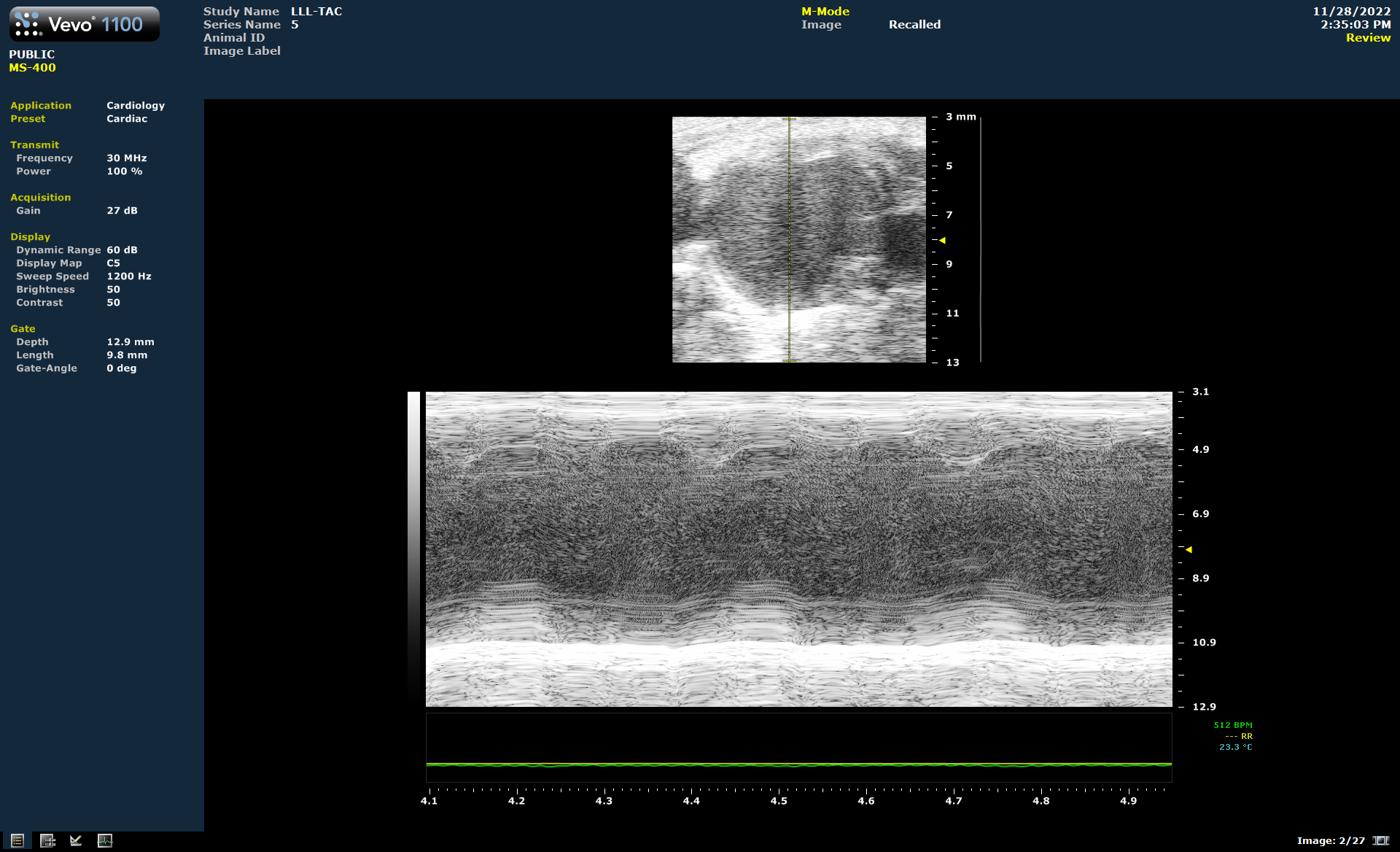

Supplement: Supplementary file 6 — Source data Fig. 3 [file 44321_2025_334_MOESM6_ESM.zip › Figure 3/3B/B Mode/RBMS1-flox+TAC.tif]

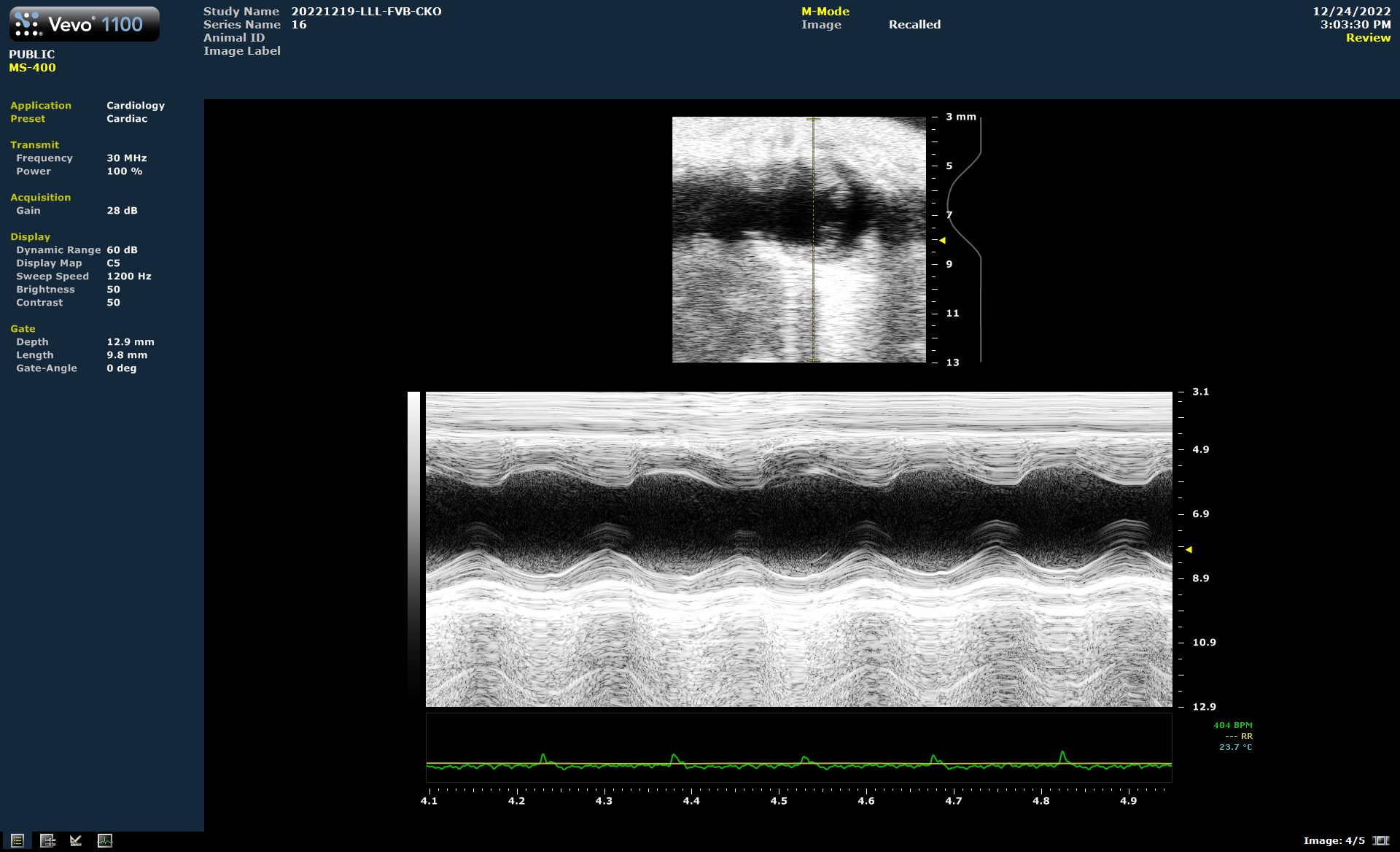

Supplement: Supplementary file 6 — Source data Fig. 3 [file 44321_2025_334_MOESM6_ESM.zip › Figure 3/3B/M Mode/RBMS1-cko+Sham.tif]

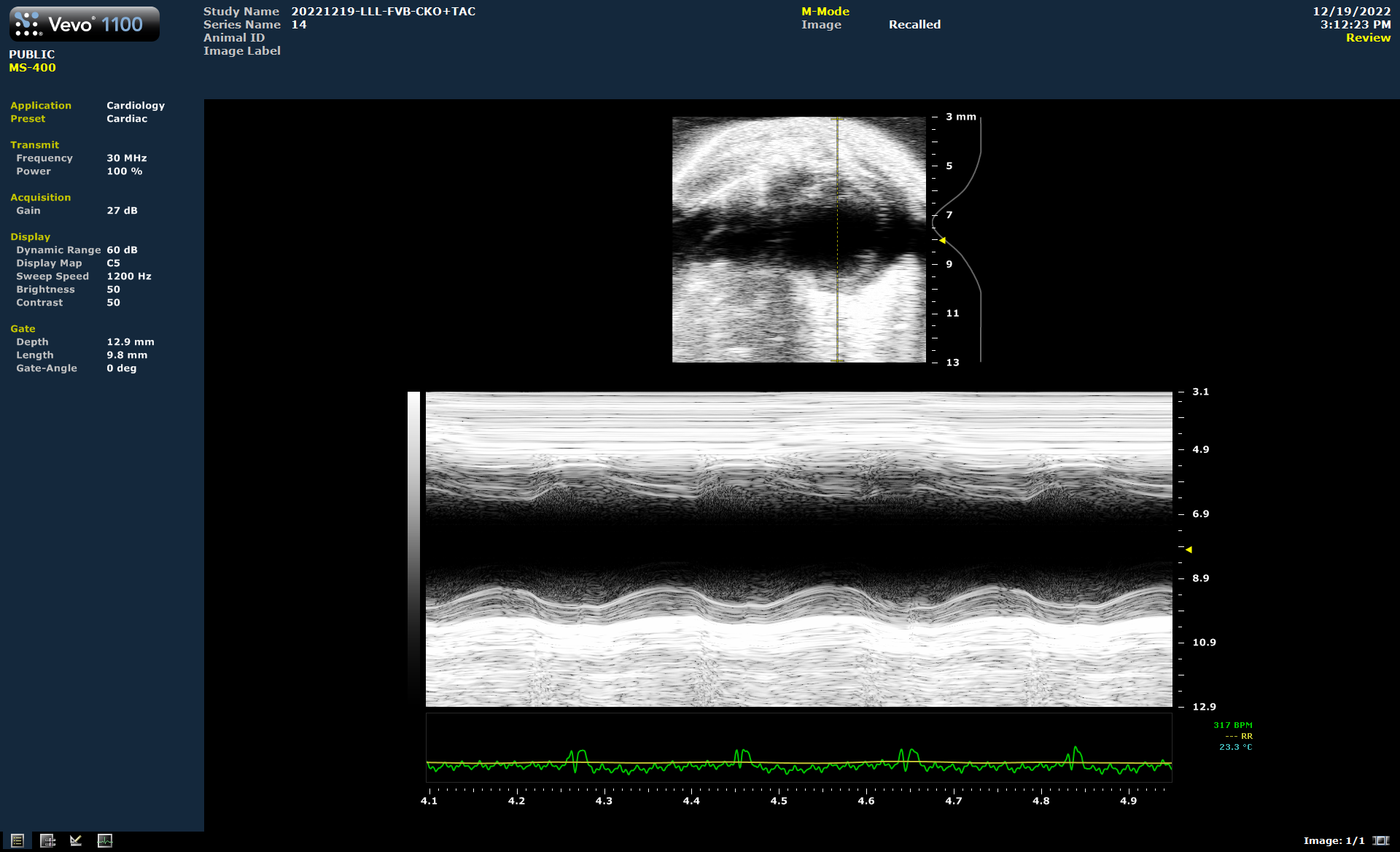

Supplement: Supplementary file 6 — Source data Fig. 3 [file 44321_2025_334_MOESM6_ESM.zip › Figure 3/3B/M Mode/RBMS1-cko+TAC.tif]

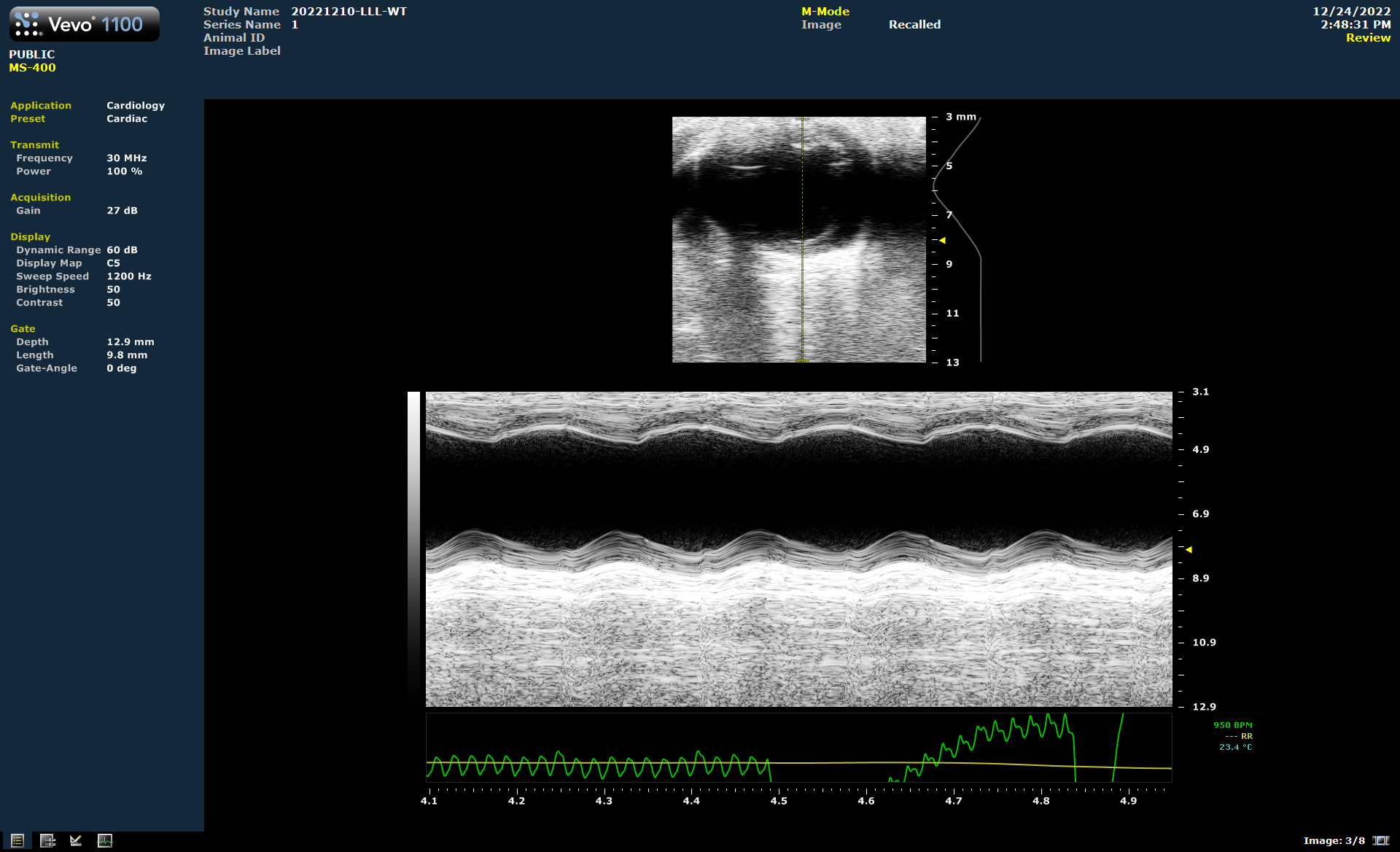

Supplement: Supplementary file 6 — Source data Fig. 3 [file 44321_2025_334_MOESM6_ESM.zip › Figure 3/3B/M Mode/RBMS1-flox+Sham.tif]

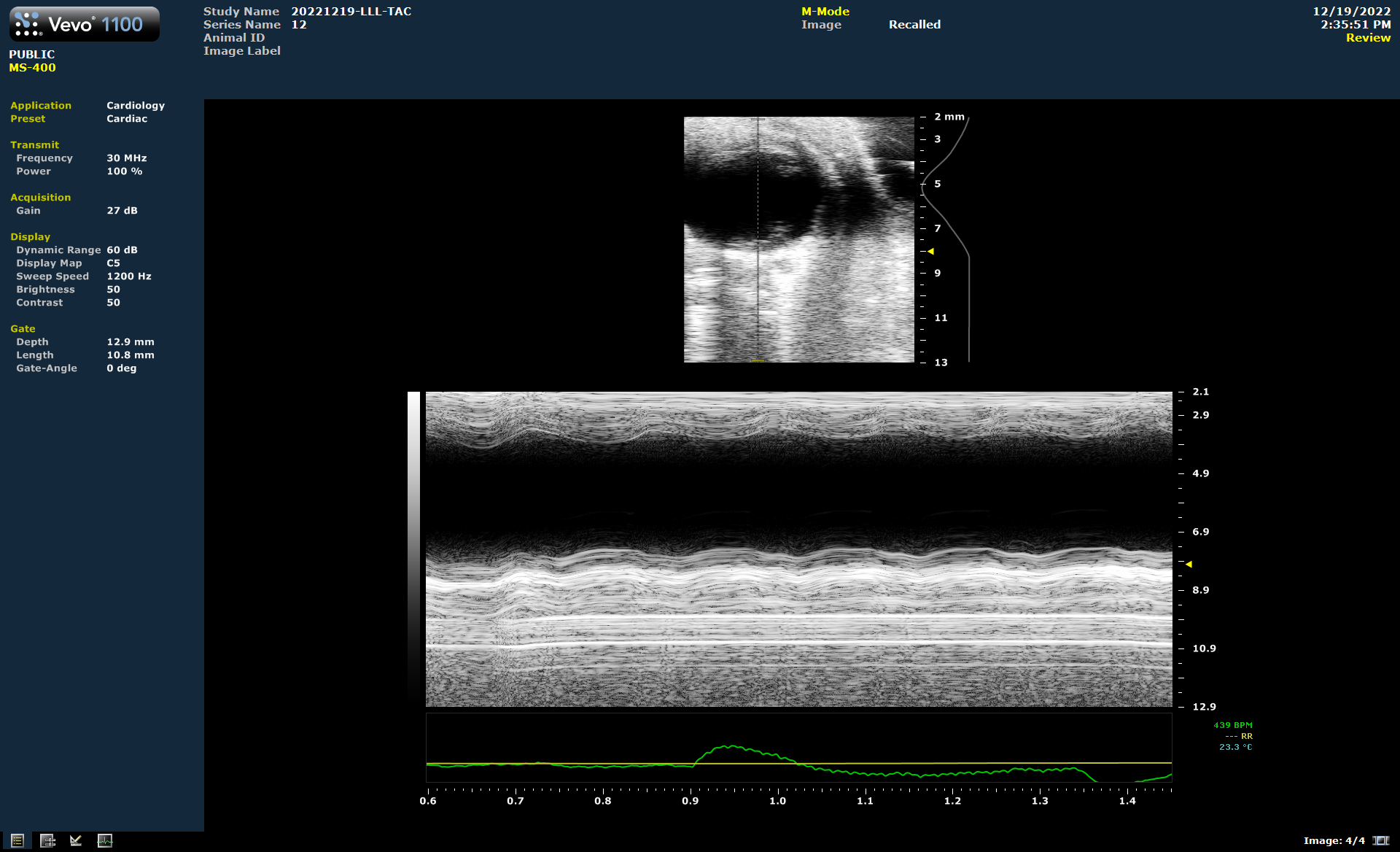

Supplement: Supplementary file 6 — Source data Fig. 3 [file 44321_2025_334_MOESM6_ESM.zip › Figure 3/3B/M Mode/RBMS1-flox+TAC.tif]

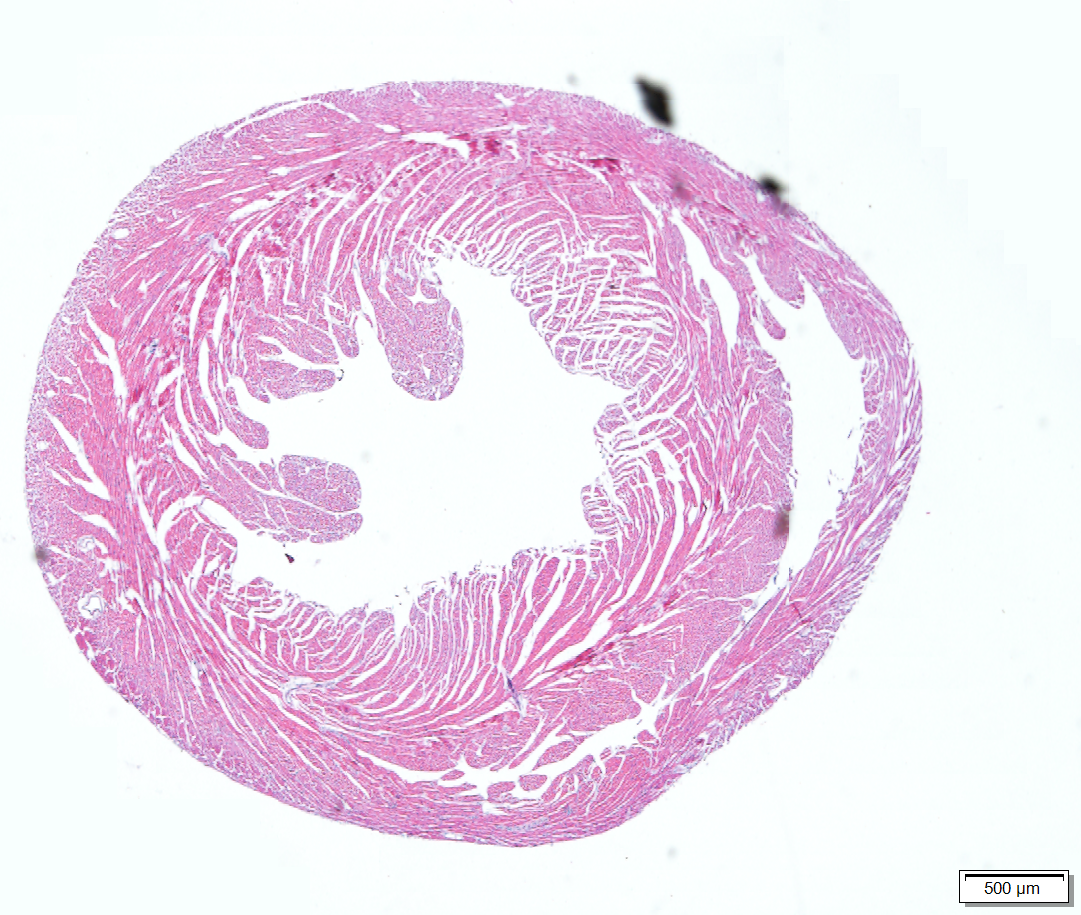

Supplement: Supplementary file 6 — Source data Fig. 3 [file 44321_2025_334_MOESM6_ESM.zip › Figure 3/3D/Cross/RBMS1-cko+Sham.tif]

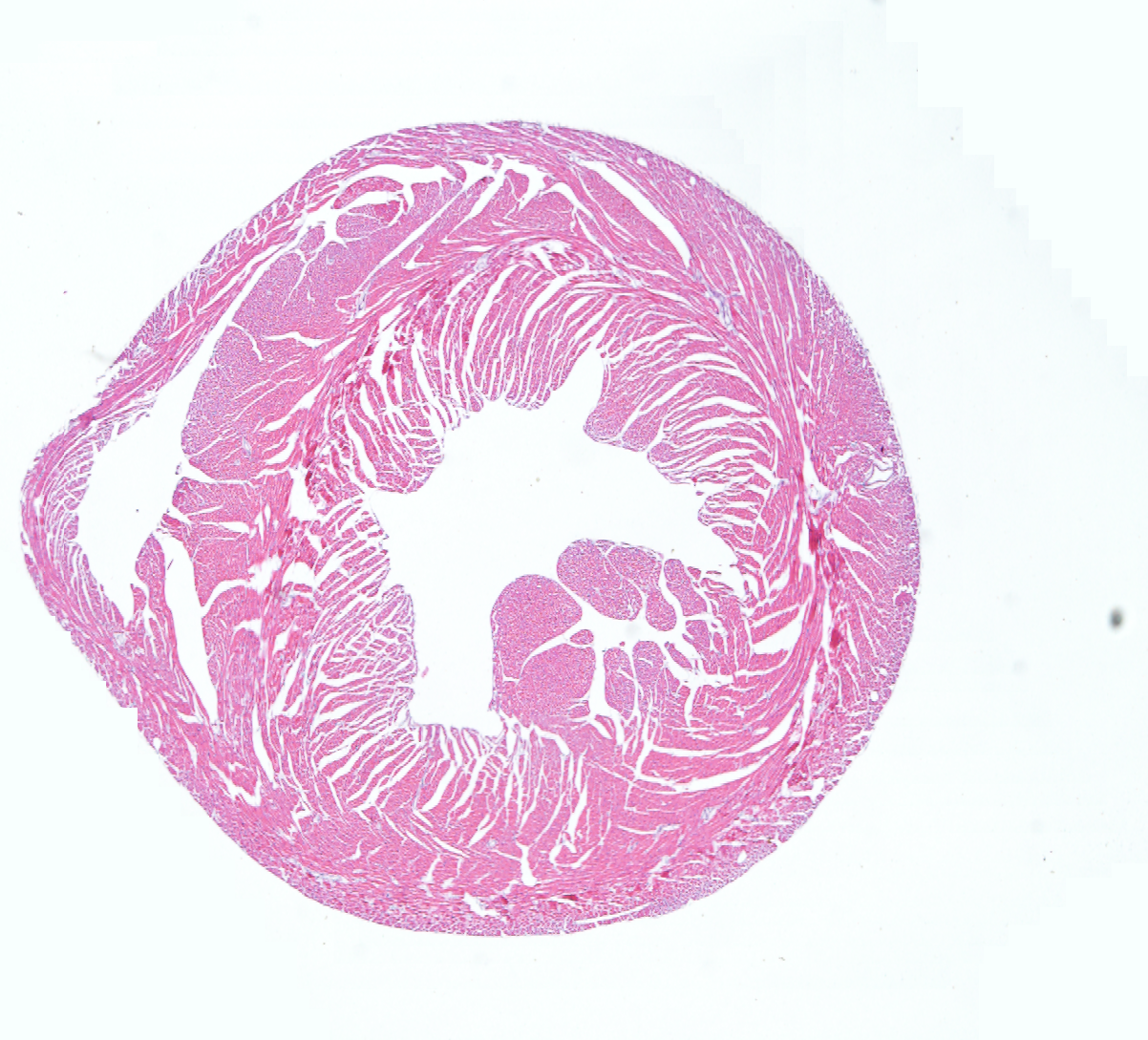

Supplement: Supplementary file 6 — Source data Fig. 3 [file 44321_2025_334_MOESM6_ESM.zip › Figure 3/3D/Cross/RBMS1-cko+TAC.tif]

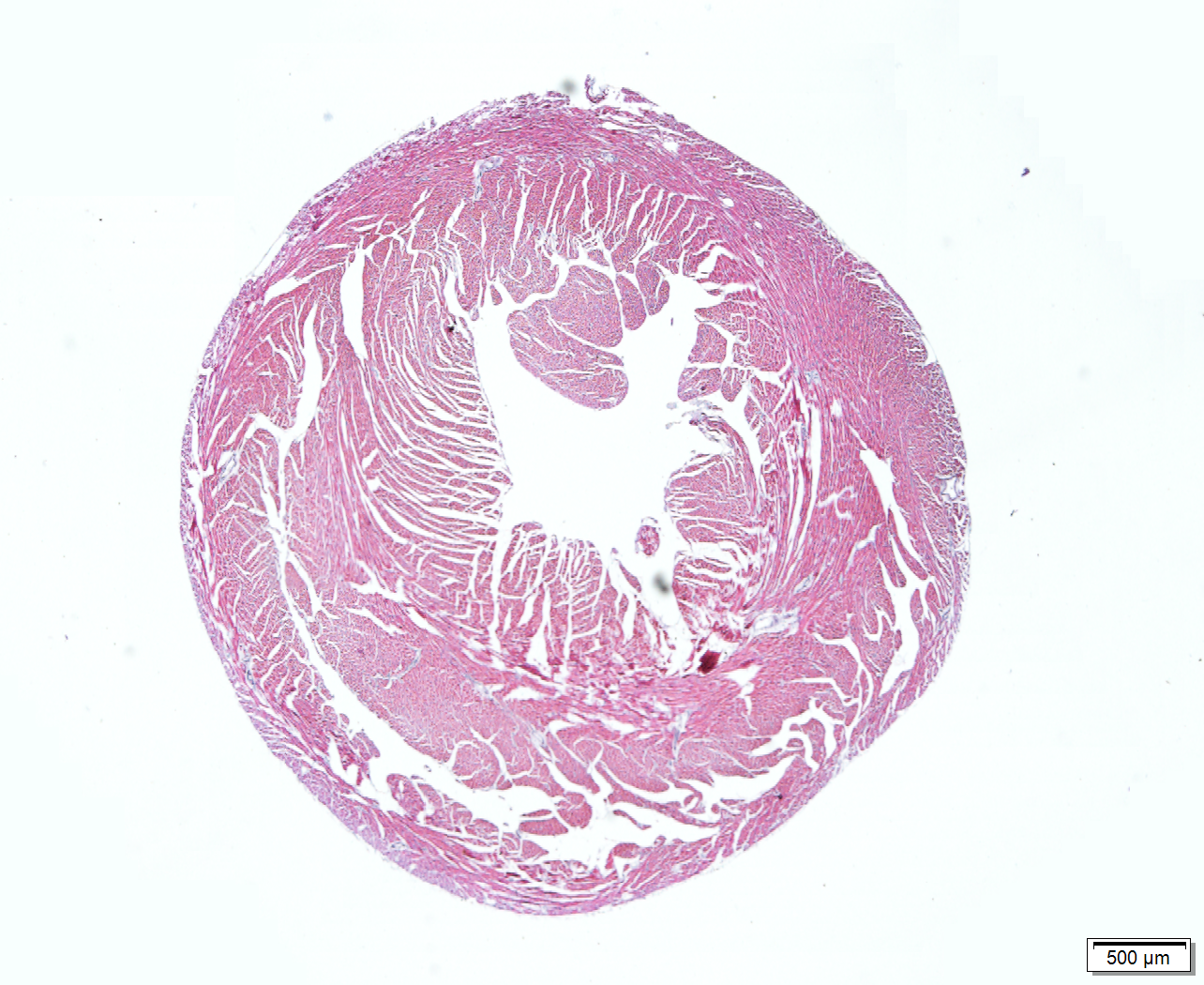

Supplement: Supplementary file 6 — Source data Fig. 3 [file 44321_2025_334_MOESM6_ESM.zip › Figure 3/3D/Cross/RBMS1-flox+Sham.tif]

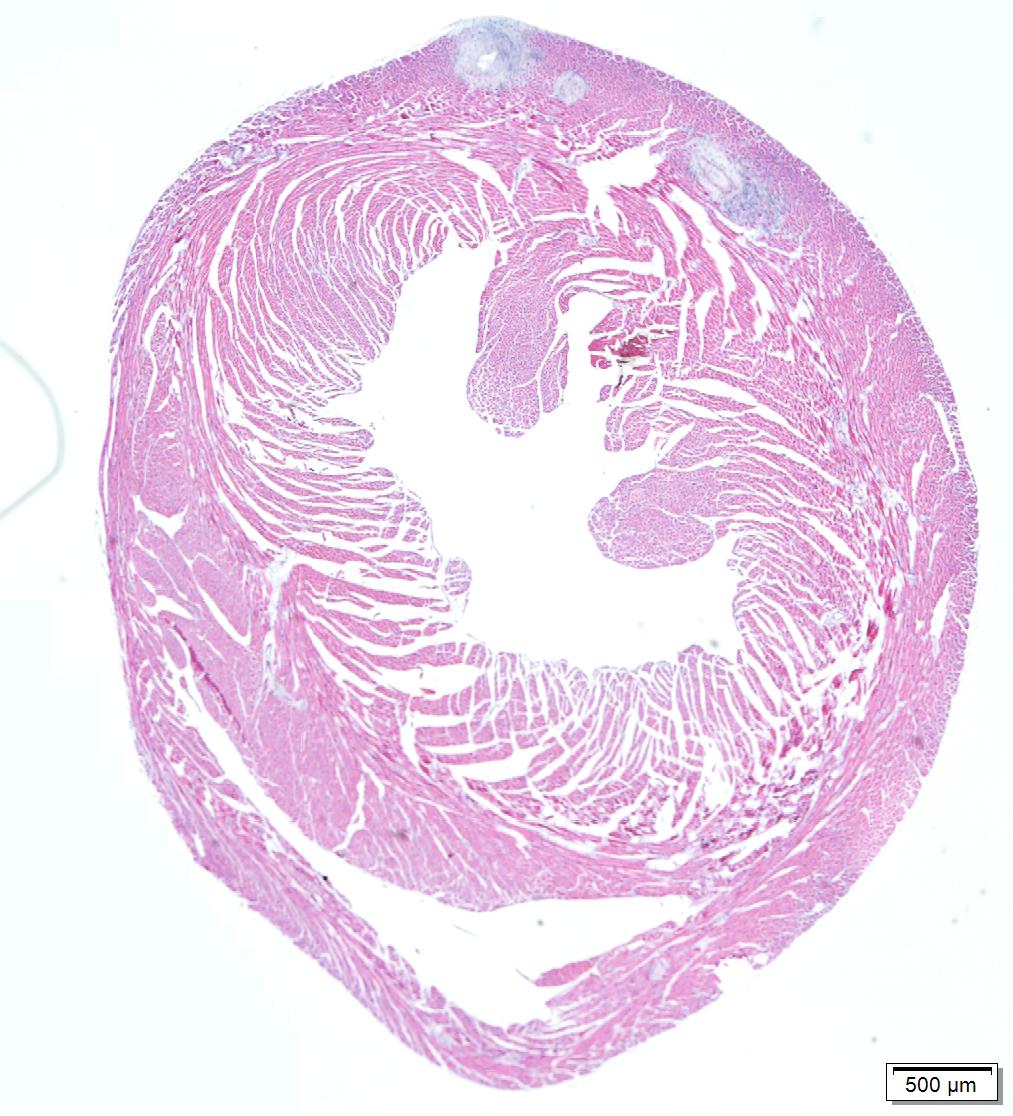

Supplement: Supplementary file 6 — Source data Fig. 3 [file 44321_2025_334_MOESM6_ESM.zip › Figure 3/3D/Cross/RBMS1-flox+TAC.tif]

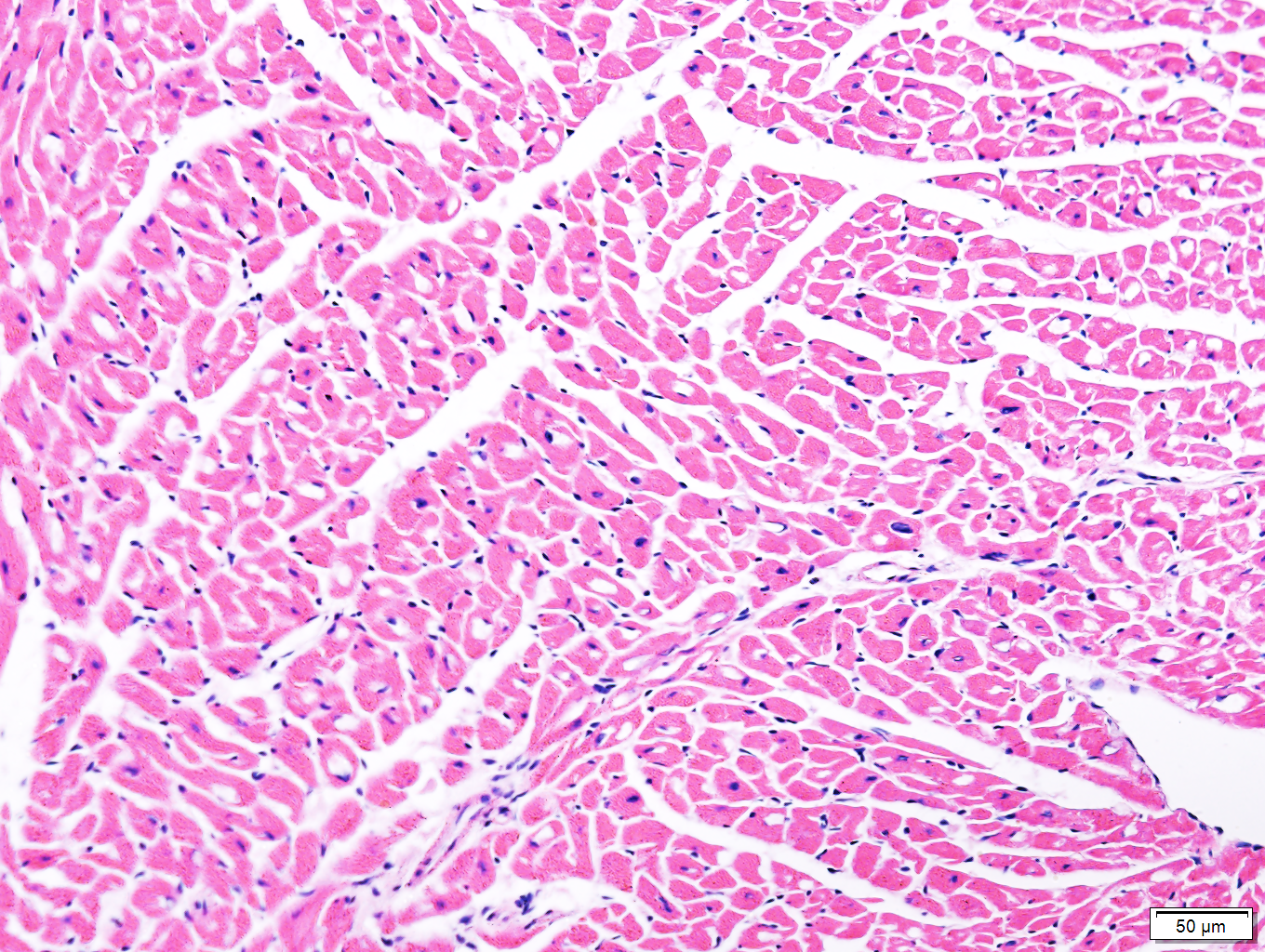

Supplement: Supplementary file 6 — Source data Fig. 3 [file 44321_2025_334_MOESM6_ESM.zip › Figure 3/3D/H&E/RBMS1-cko+Sham.tif]

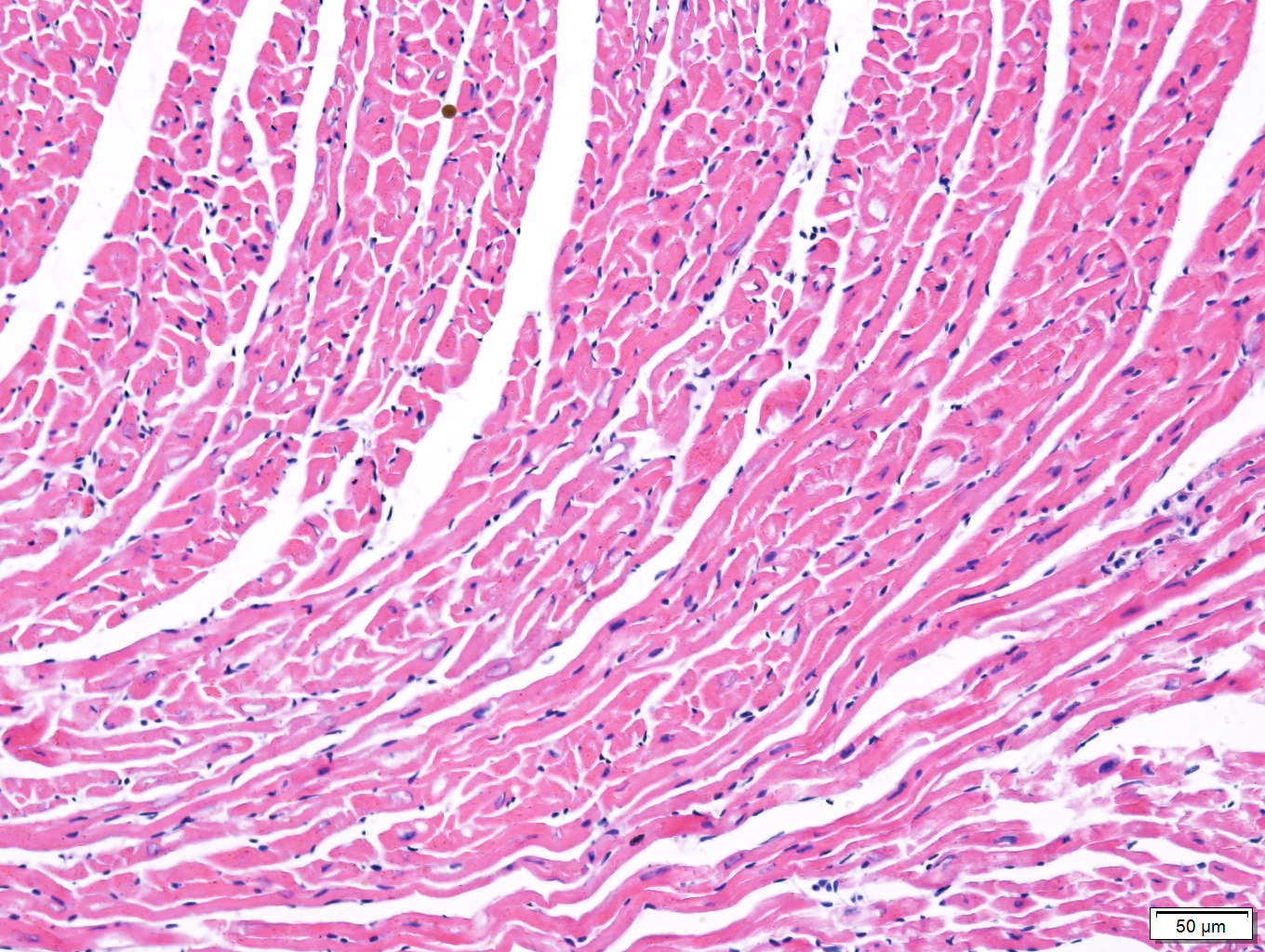

Supplement: Supplementary file 6 — Source data Fig. 3 [file 44321_2025_334_MOESM6_ESM.zip › Figure 3/3D/H&E/RBMS1-cko+TAC.tif]

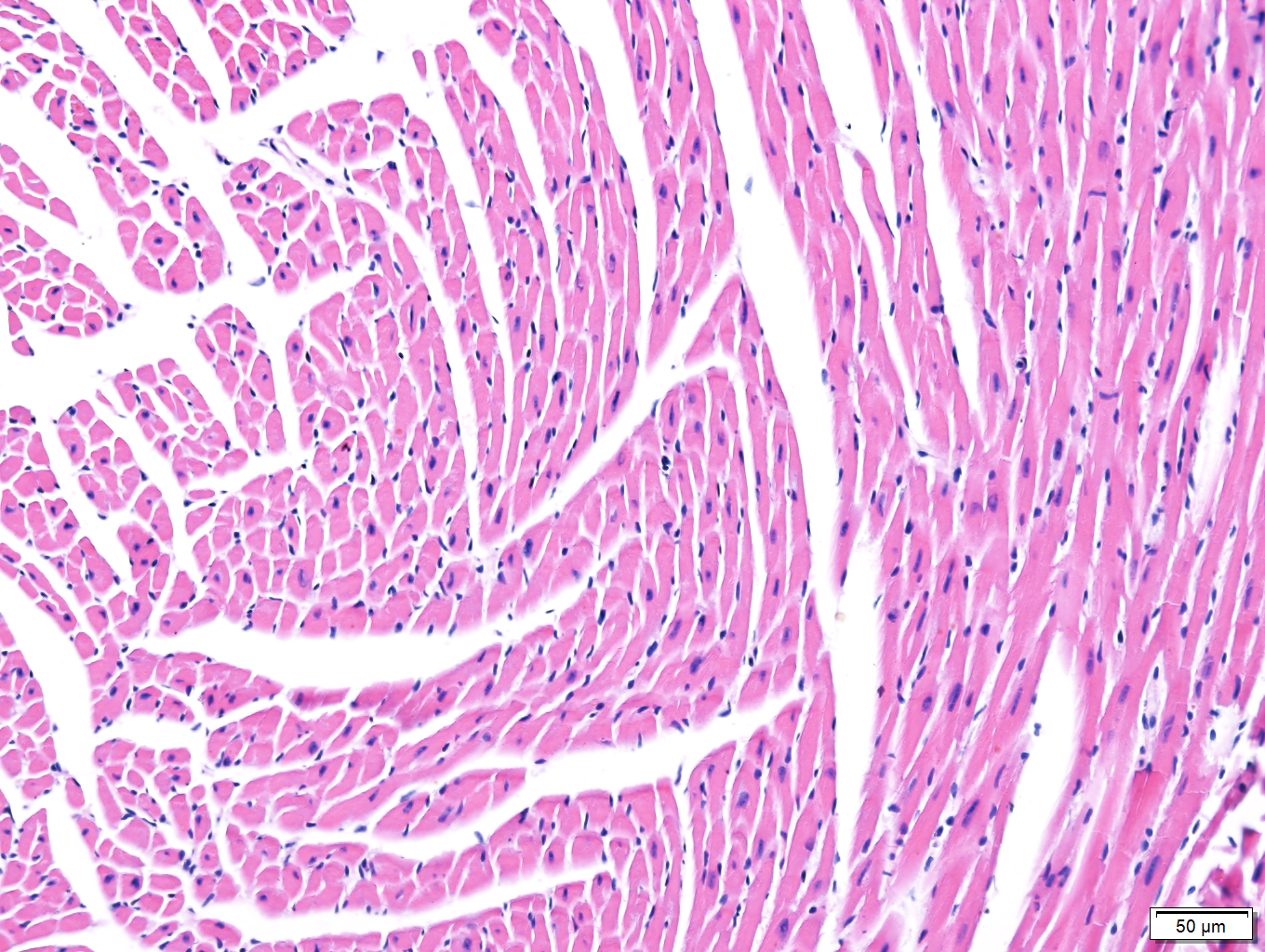

Supplement: Supplementary file 6 — Source data Fig. 3 [file 44321_2025_334_MOESM6_ESM.zip › Figure 3/3D/H&E/RBMS1-flox+Sham.tif]

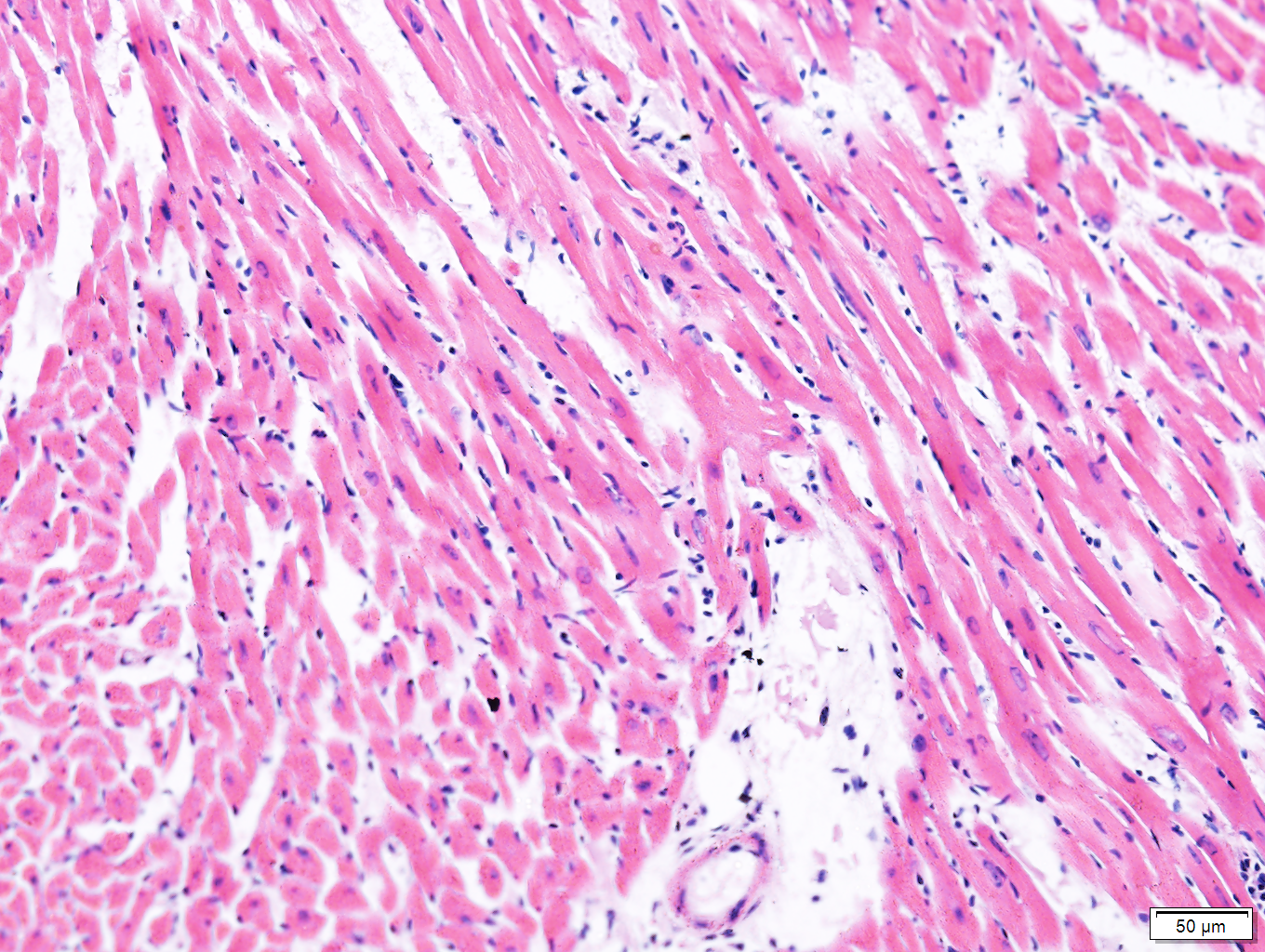

Supplement: Supplementary file 6 — Source data Fig. 3 [file 44321_2025_334_MOESM6_ESM.zip › Figure 3/3D/H&E/RBMS1-flox+TAC.tif]

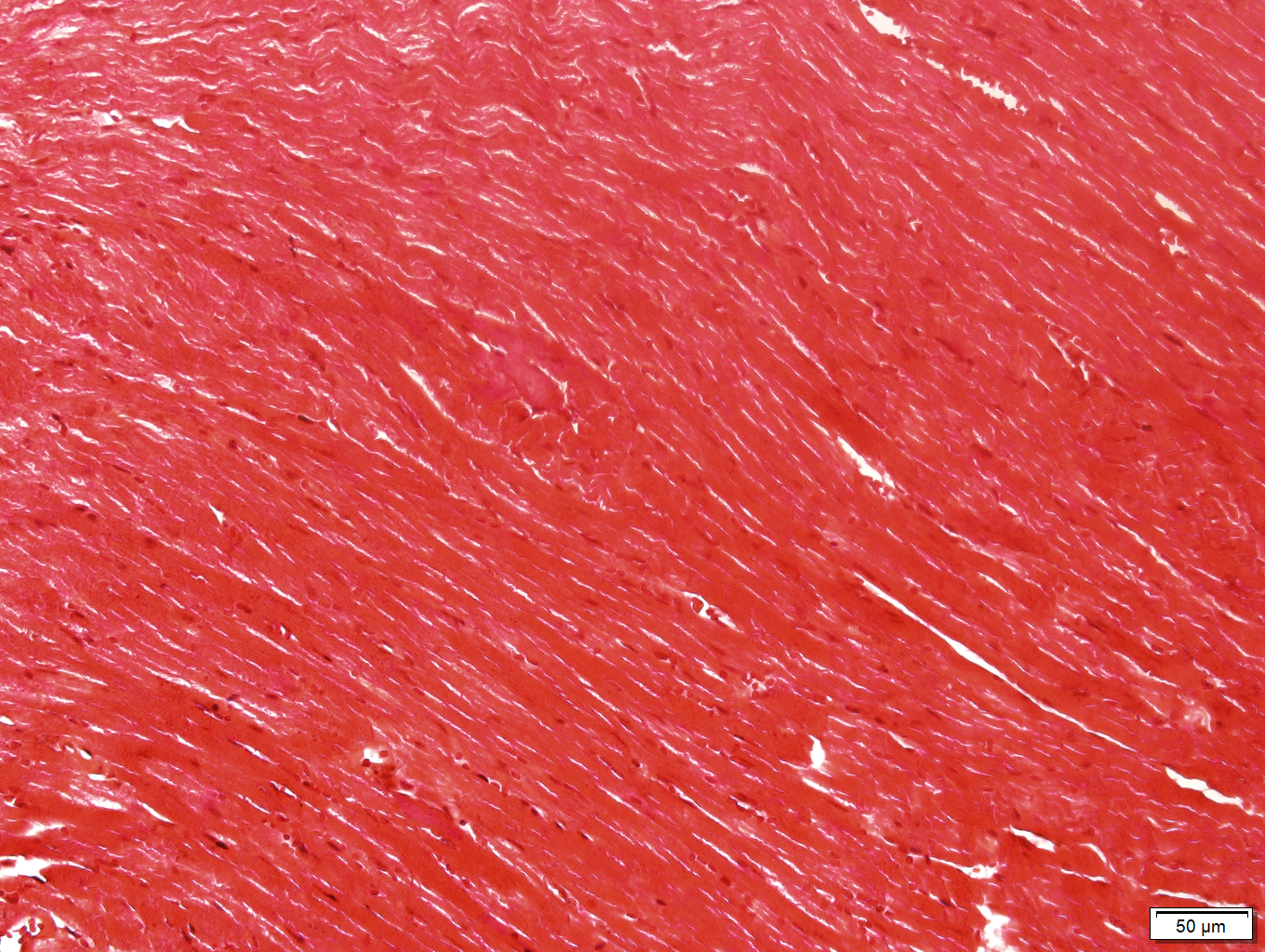

Supplement: Supplementary file 6 — Source data Fig. 3 [file 44321_2025_334_MOESM6_ESM.zip › Figure 3/3D/Interstital/RBMS1-cko+Sham.tif]

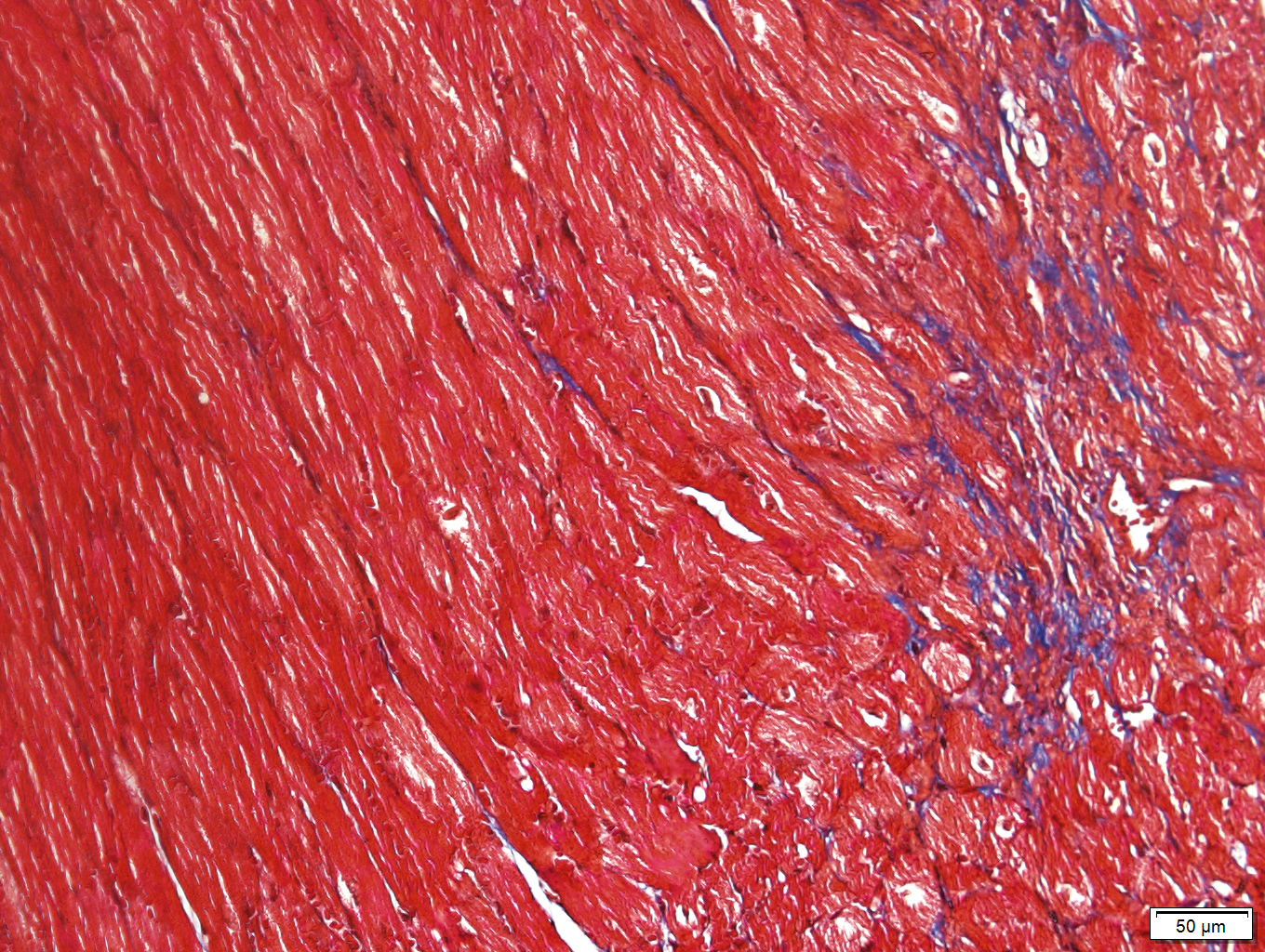

Supplement: Supplementary file 6 — Source data Fig. 3 [file 44321_2025_334_MOESM6_ESM.zip › Figure 3/3D/Interstital/RBMS1-cko+TAC.tif]

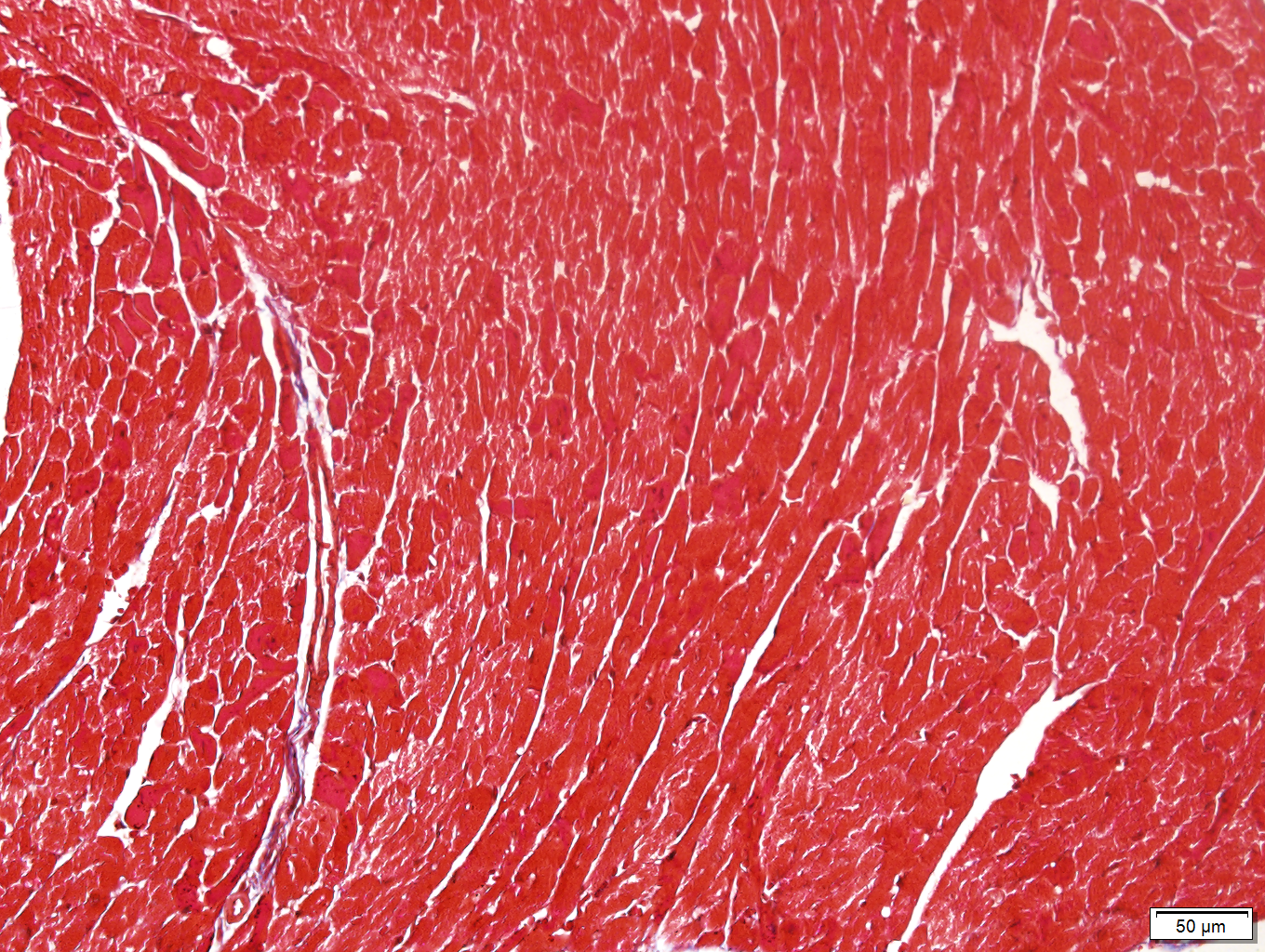

Supplement: Supplementary file 6 — Source data Fig. 3 [file 44321_2025_334_MOESM6_ESM.zip › Figure 3/3D/Interstital/RBMS1-flox+Sham.tif]

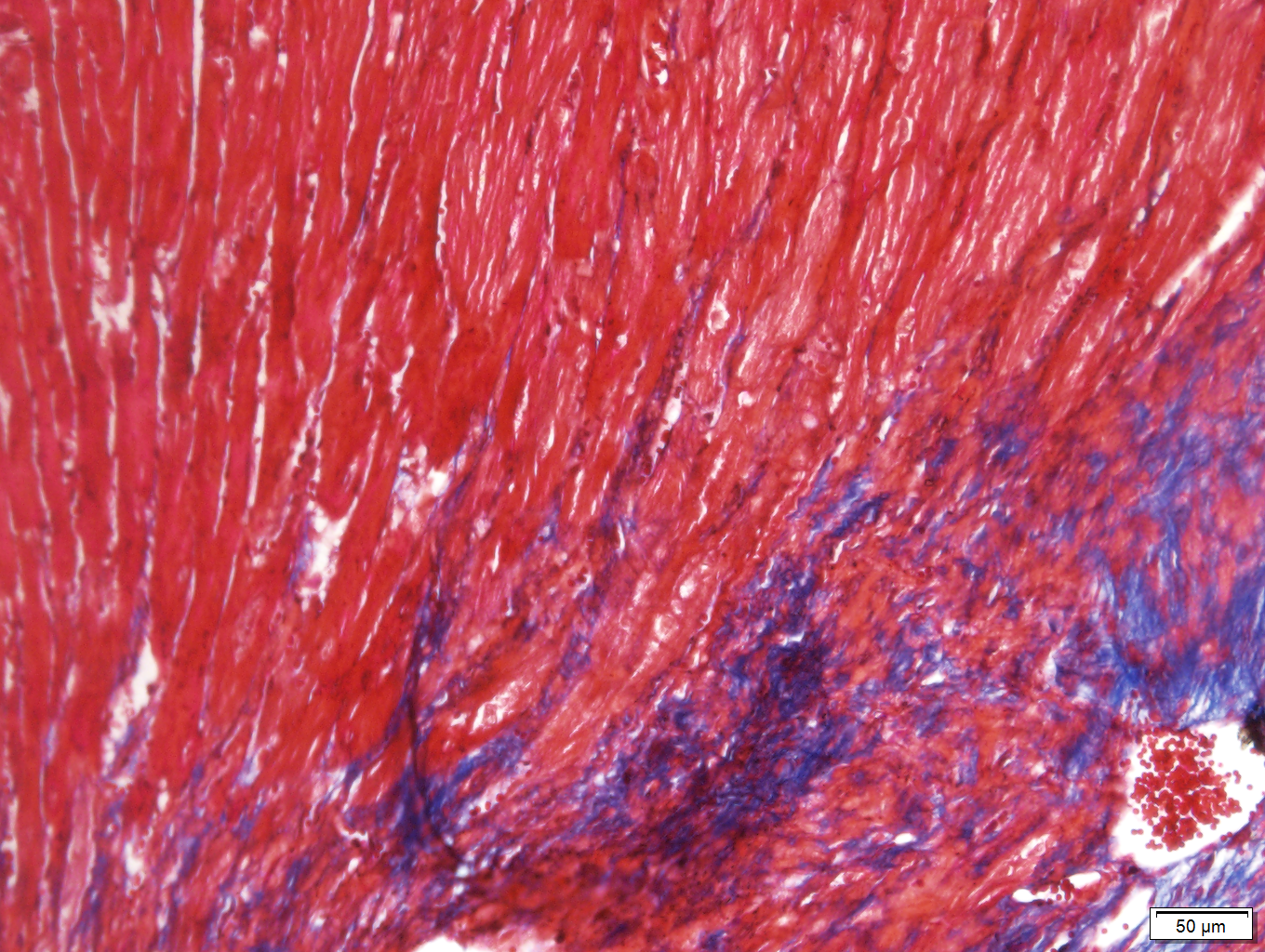

Supplement: Supplementary file 6 — Source data Fig. 3 [file 44321_2025_334_MOESM6_ESM.zip › Figure 3/3D/Interstital/RBMS1-flox+TAC.tif]

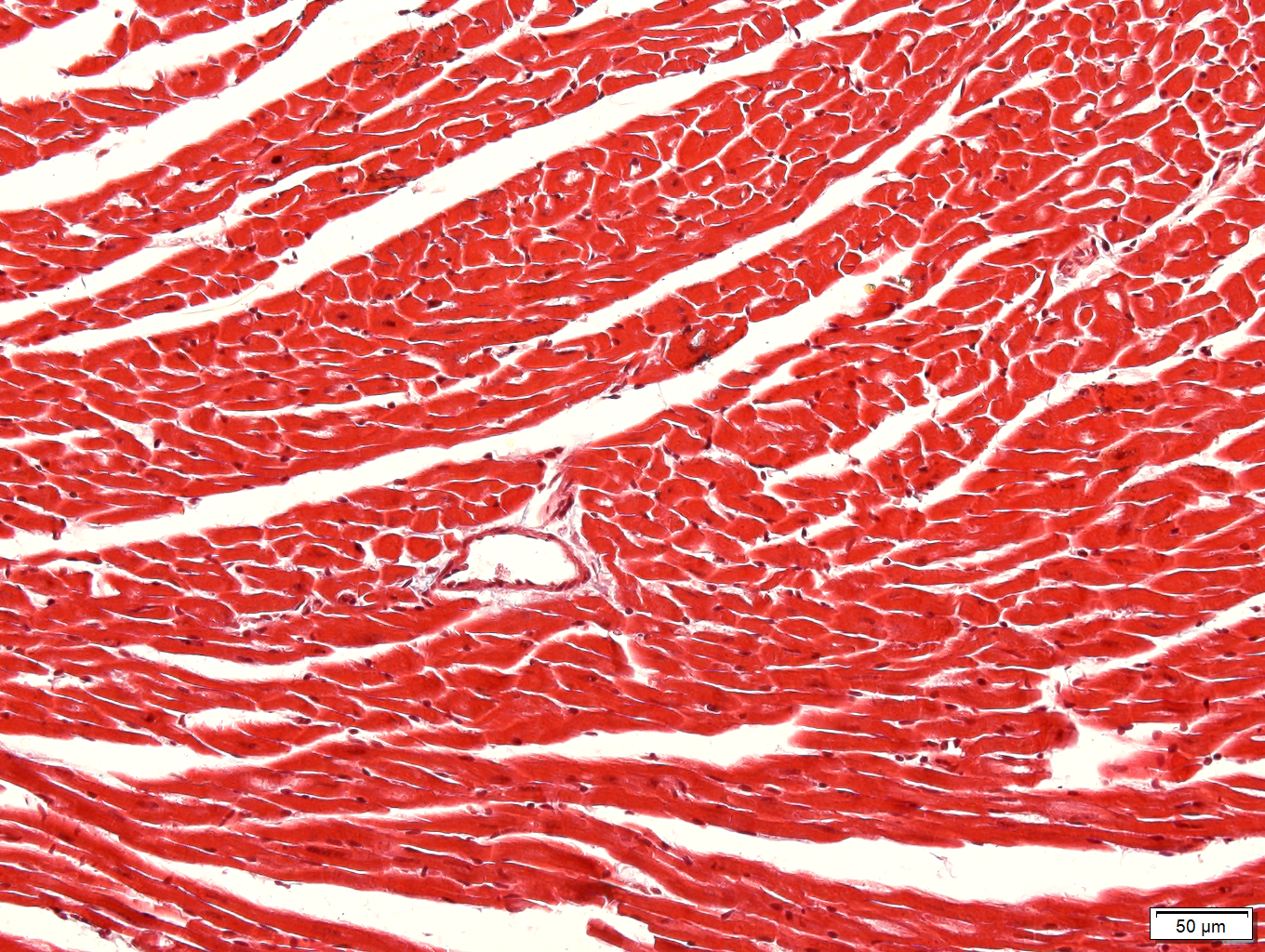

Supplement: Supplementary file 6 — Source data Fig. 3 [file 44321_2025_334_MOESM6_ESM.zip › Figure 3/3D/Perivascular/RBMS1-cko+Sham.tif]

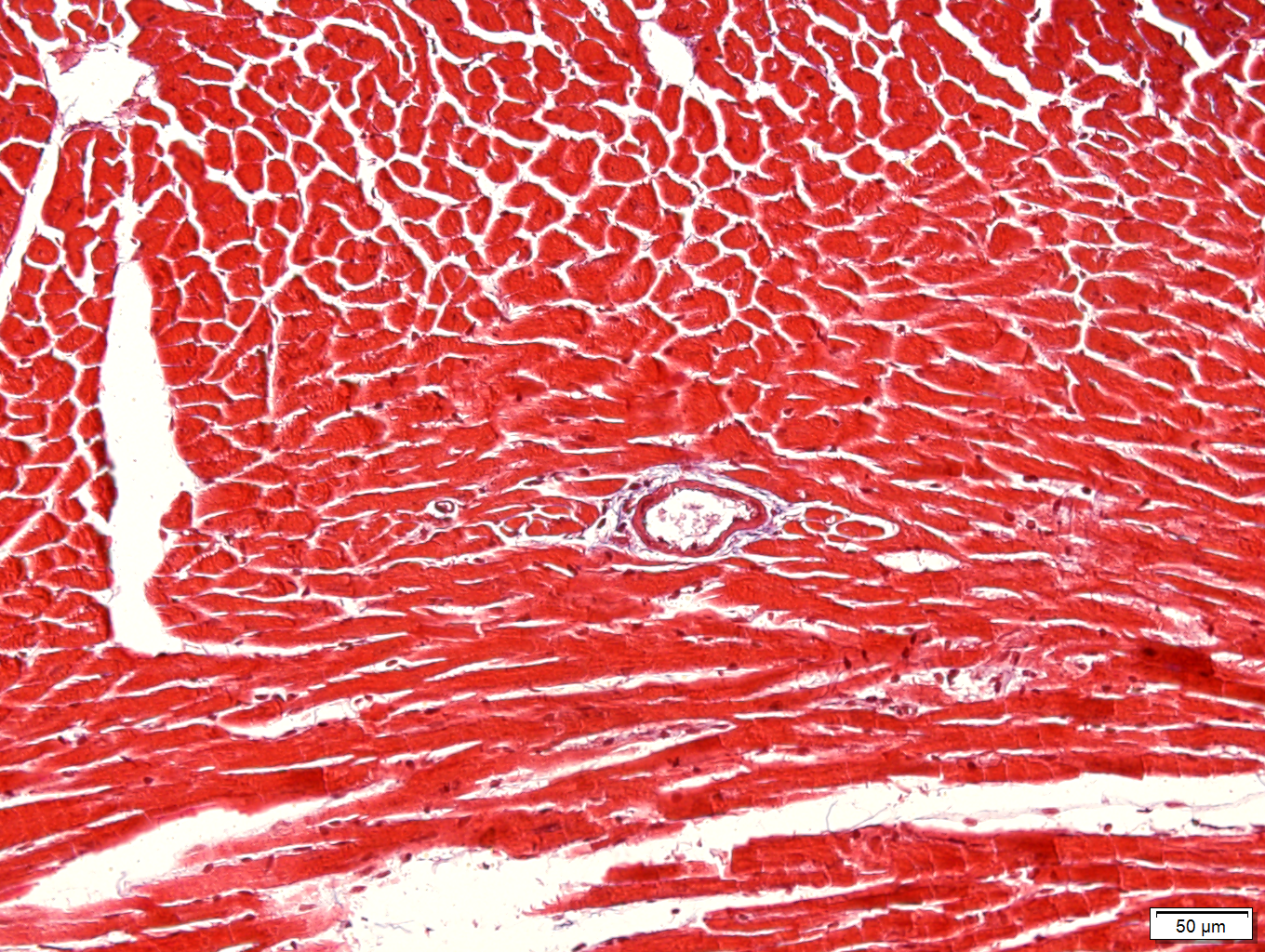

Supplement: Supplementary file 6 — Source data Fig. 3 [file 44321_2025_334_MOESM6_ESM.zip › Figure 3/3D/Perivascular/RBMS1-cko+TAC.tif]

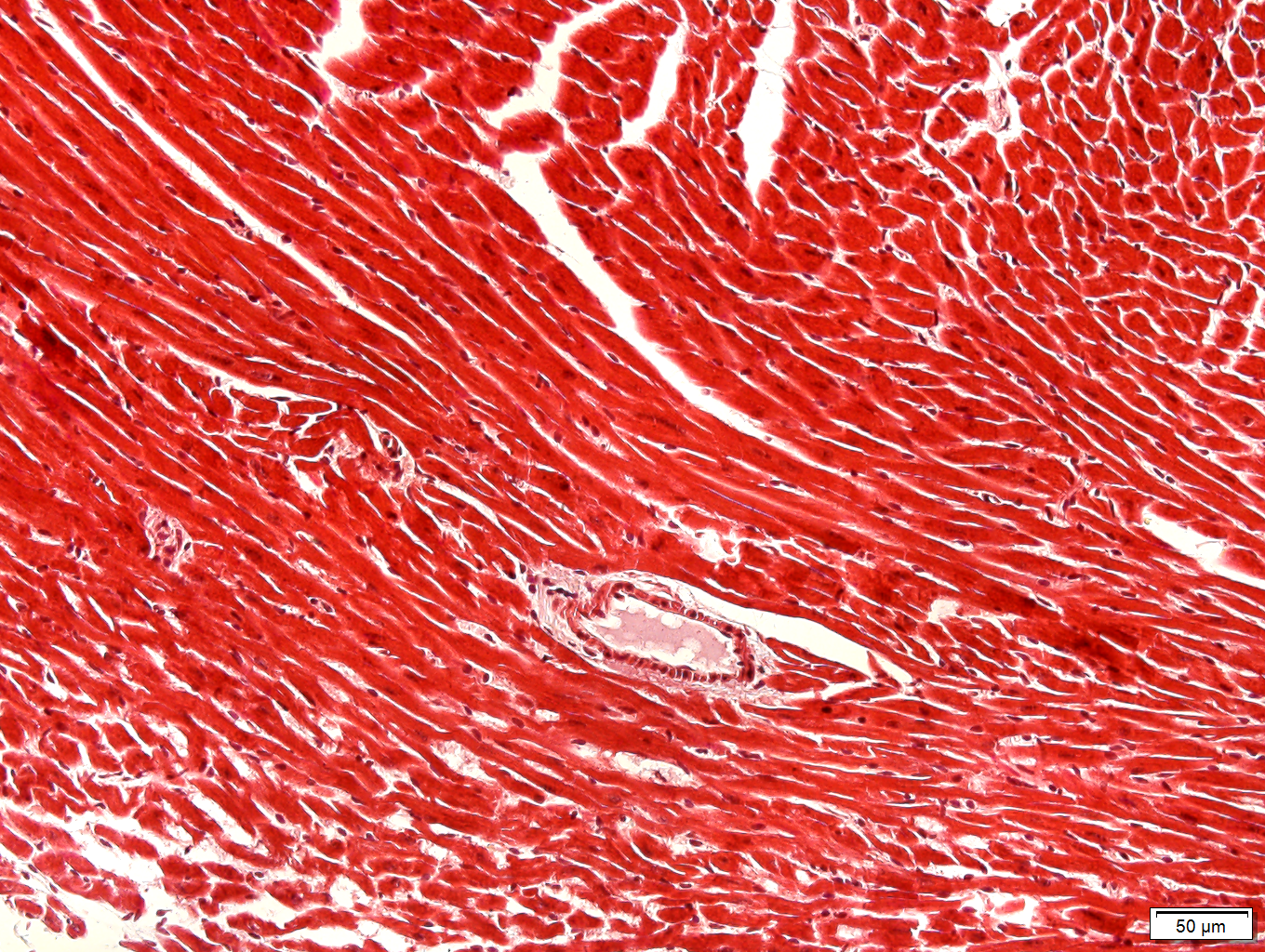

Supplement: Supplementary file 6 — Source data Fig. 3 [file 44321_2025_334_MOESM6_ESM.zip › Figure 3/3D/Perivascular/RBMS1-flox+Sham.tif]

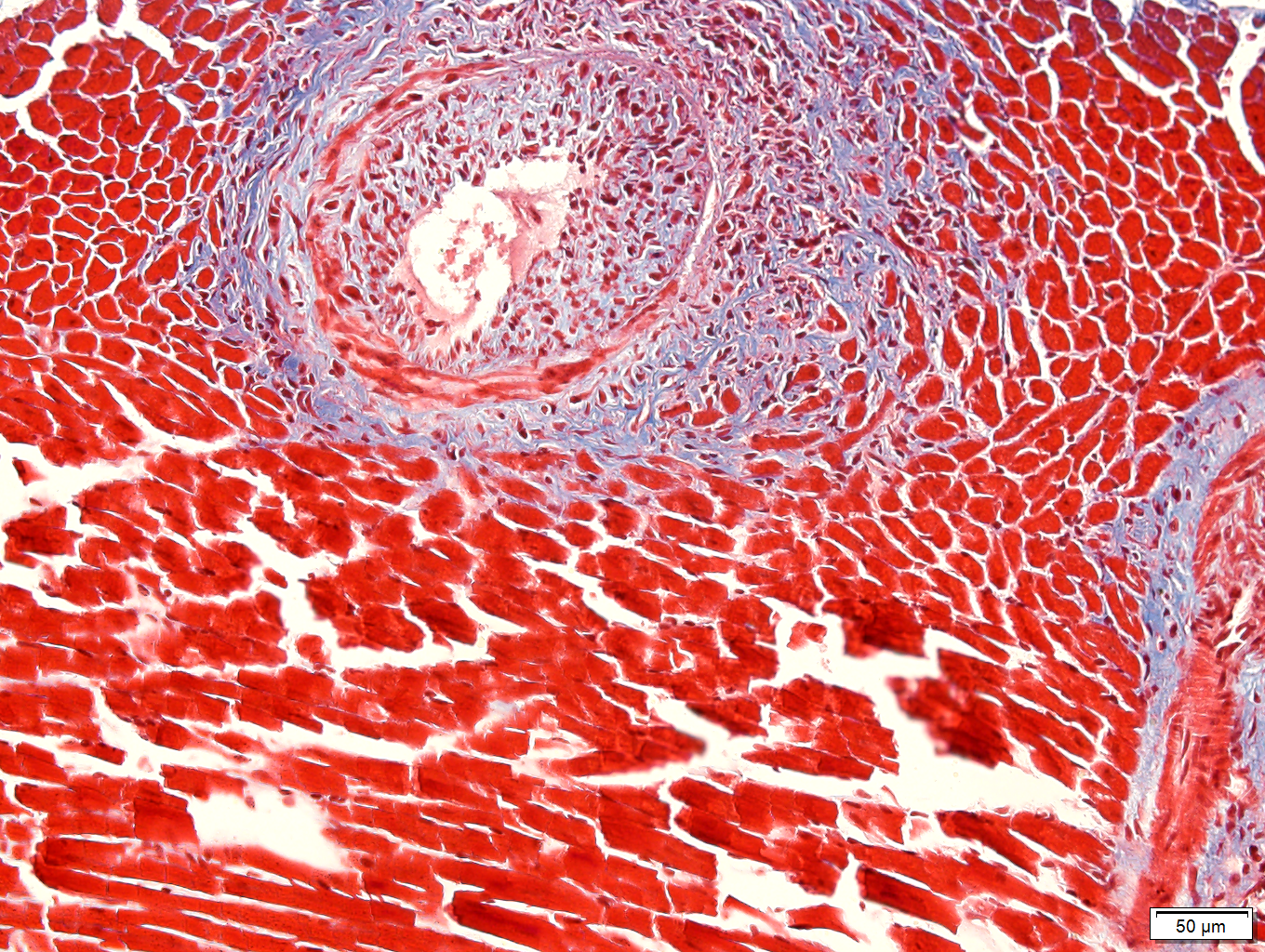

Supplement: Supplementary file 6 — Source data Fig. 3 [file 44321_2025_334_MOESM6_ESM.zip › Figure 3/3D/Perivascular/RBMS1-flox+TAC.tif]

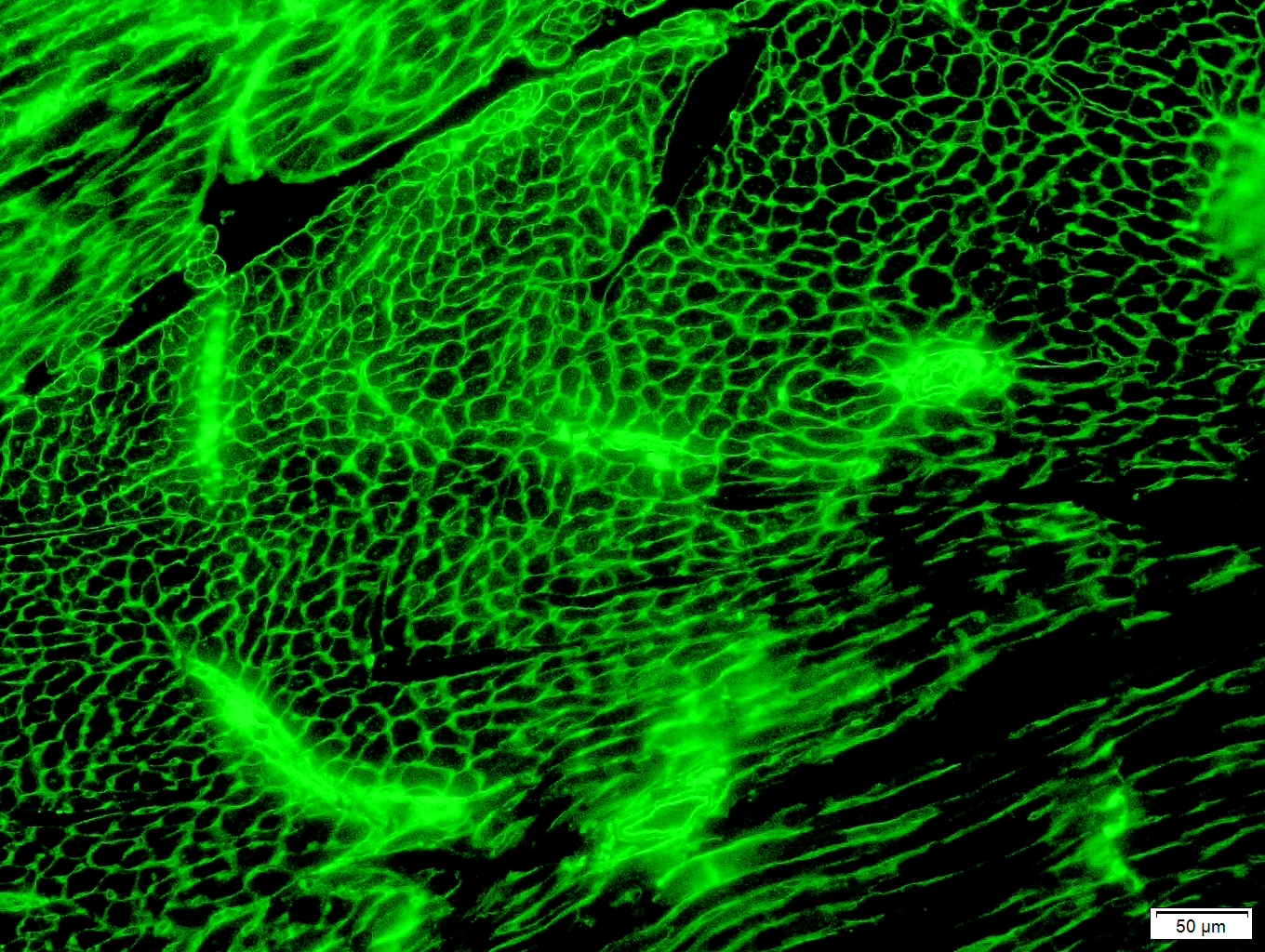

Supplement: Supplementary file 6 — Source data Fig. 3 [file 44321_2025_334_MOESM6_ESM.zip › Figure 3/3D/WGA/RBMS1-cko+Sham.tif]

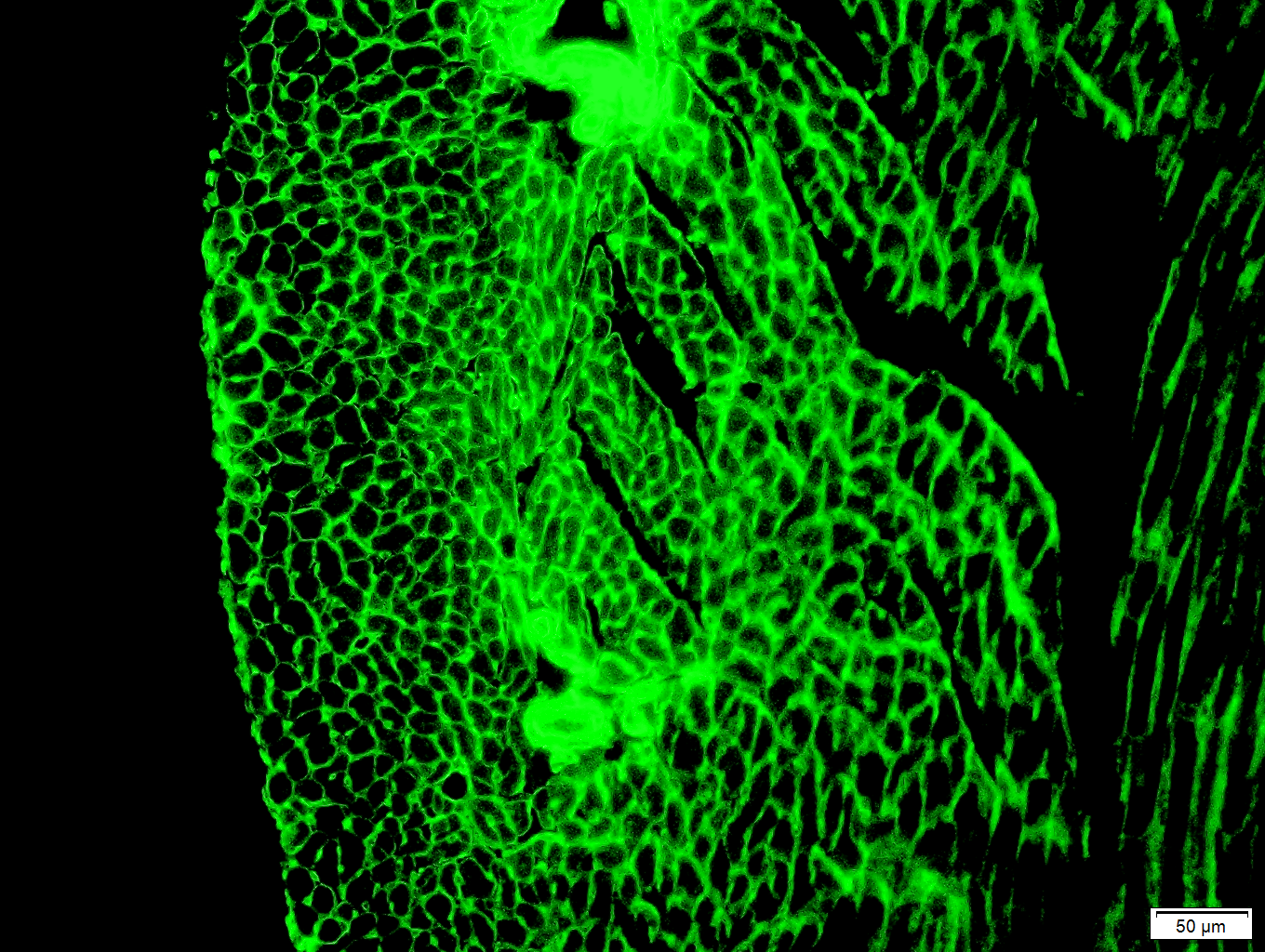

Supplement: Supplementary file 6 — Source data Fig. 3 [file 44321_2025_334_MOESM6_ESM.zip › Figure 3/3D/WGA/RBMS1-cko+TAC.tif]

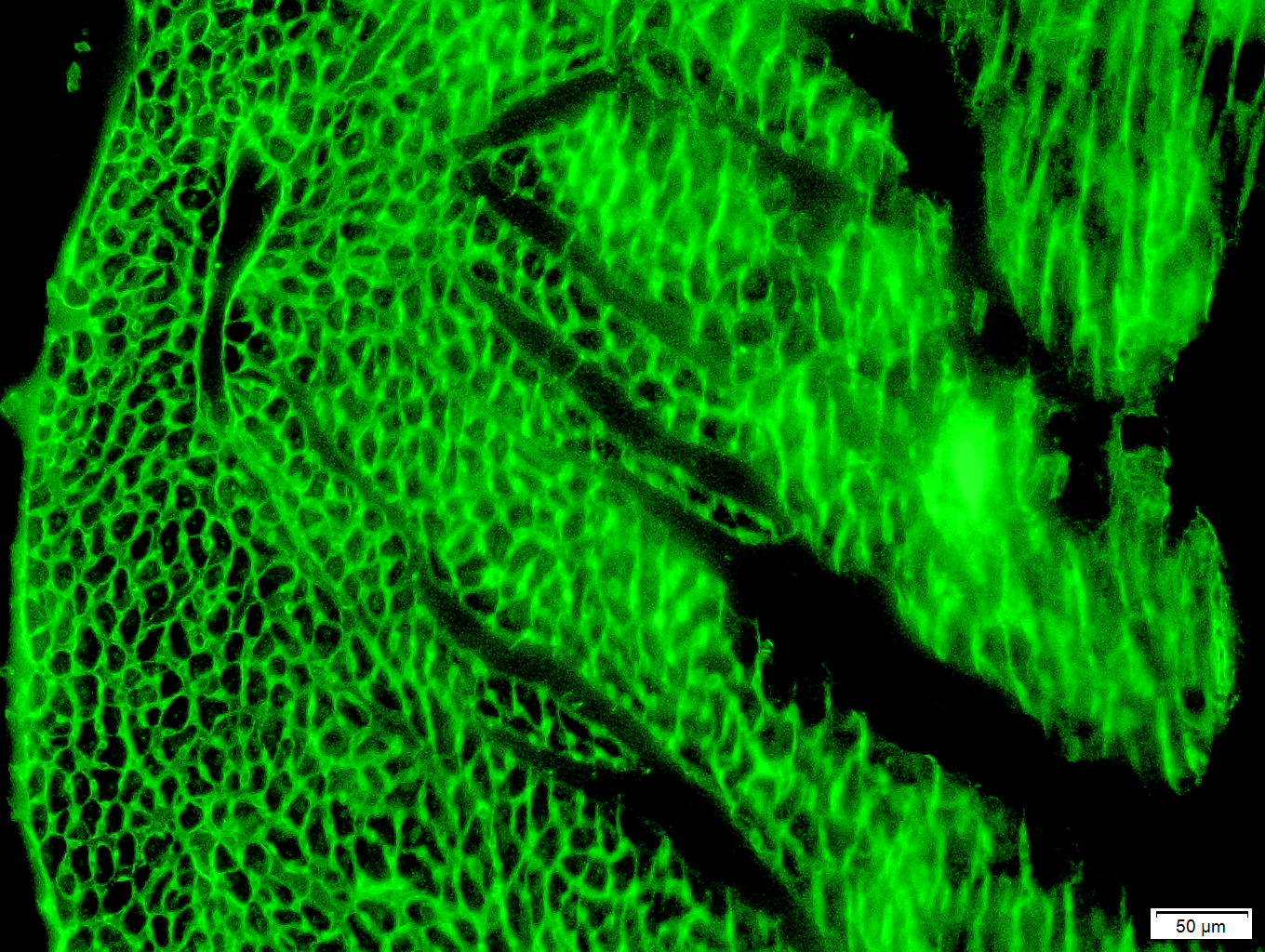

Supplement: Supplementary file 6 — Source data Fig. 3 [file 44321_2025_334_MOESM6_ESM.zip › Figure 3/3D/WGA/RBMS1-flox+Sham.tif]

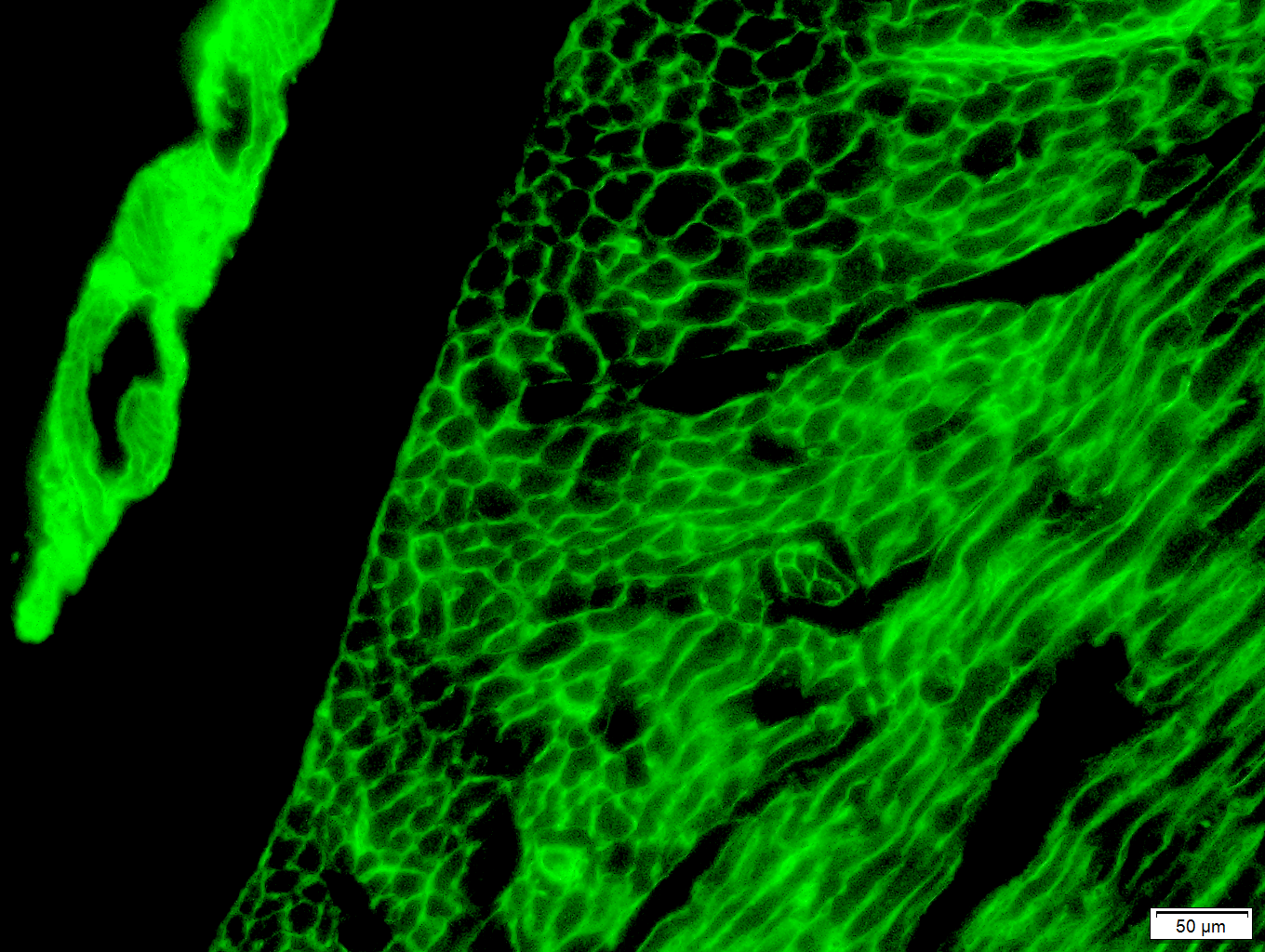

Supplement: Supplementary file 6 — Source data Fig. 3 [file 44321_2025_334_MOESM6_ESM.zip › Figure 3/3D/WGA/RBMS1-flox+TAC.tif]

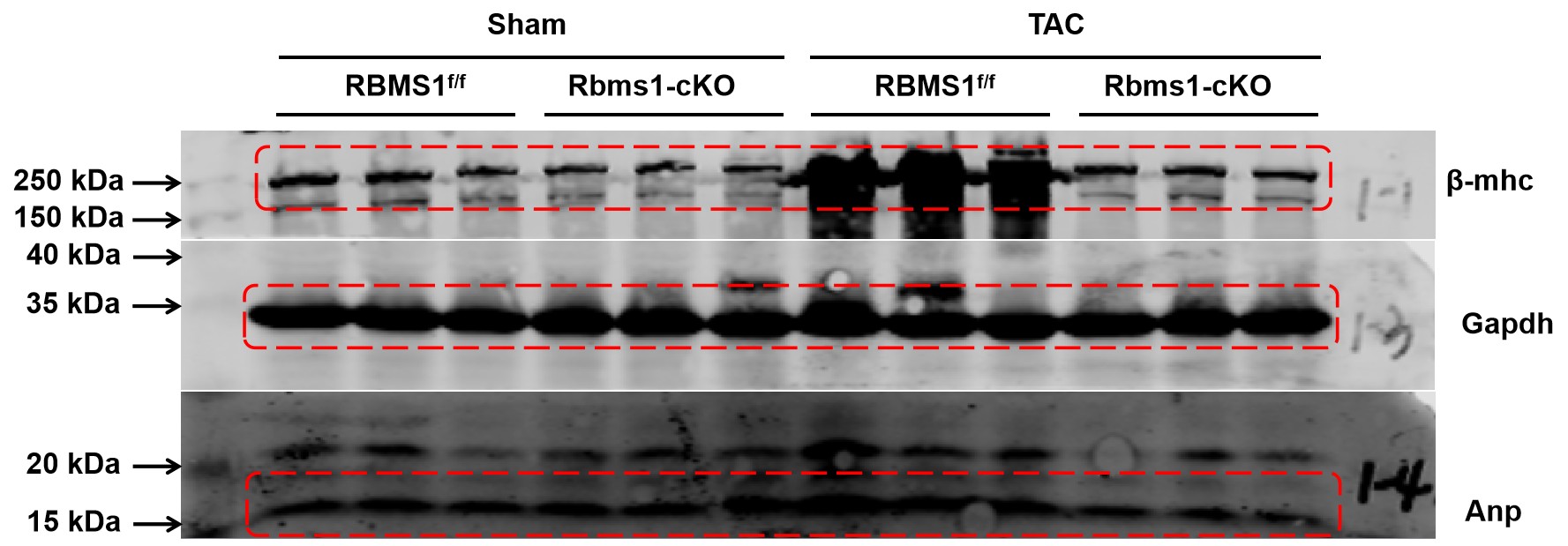

Supplement: Supplementary file 6 — Source data Fig. 3 [file 44321_2025_334_MOESM6_ESM.zip › Figure 3/3H/3H.jpg]

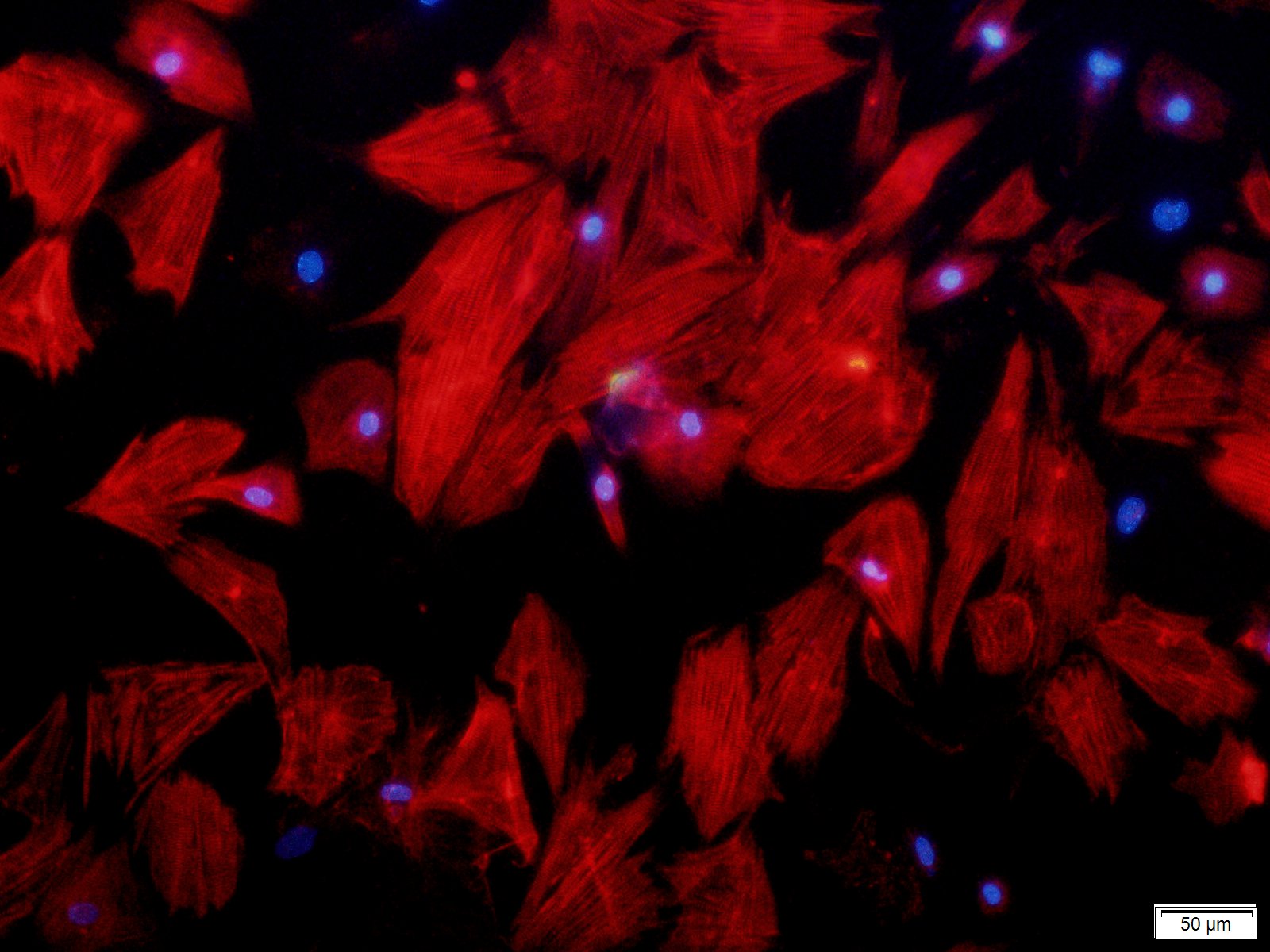

Supplement: Supplementary file 6 — Source data Fig. 3 [file 44321_2025_334_MOESM6_ESM.zip › Figure 3/3K/Ang II+si-NC.jpg]

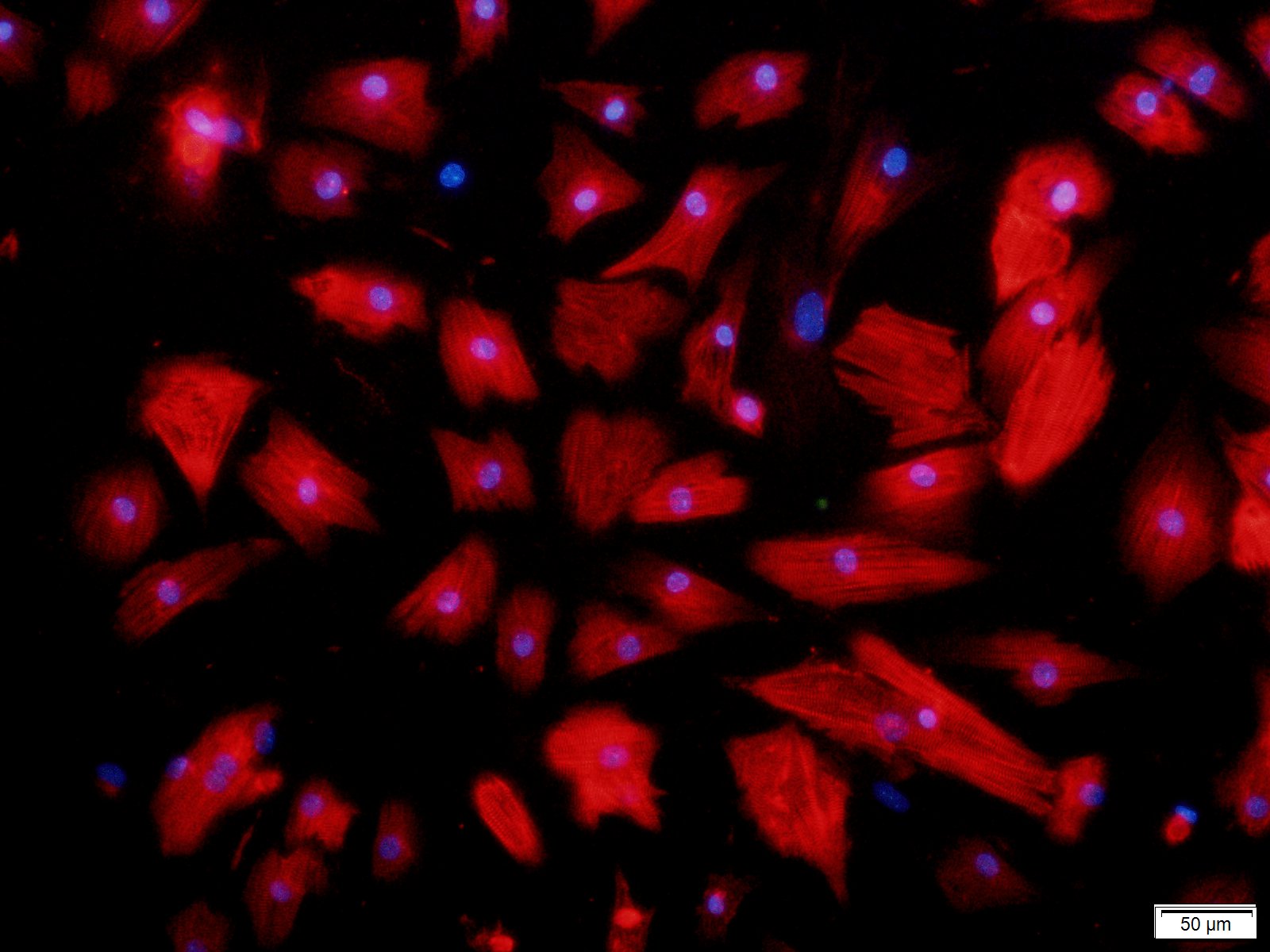

Supplement: Supplementary file 6 — Source data Fig. 3 [file 44321_2025_334_MOESM6_ESM.zip › Figure 3/3K/Ang II+si-RBMS1.jpg]

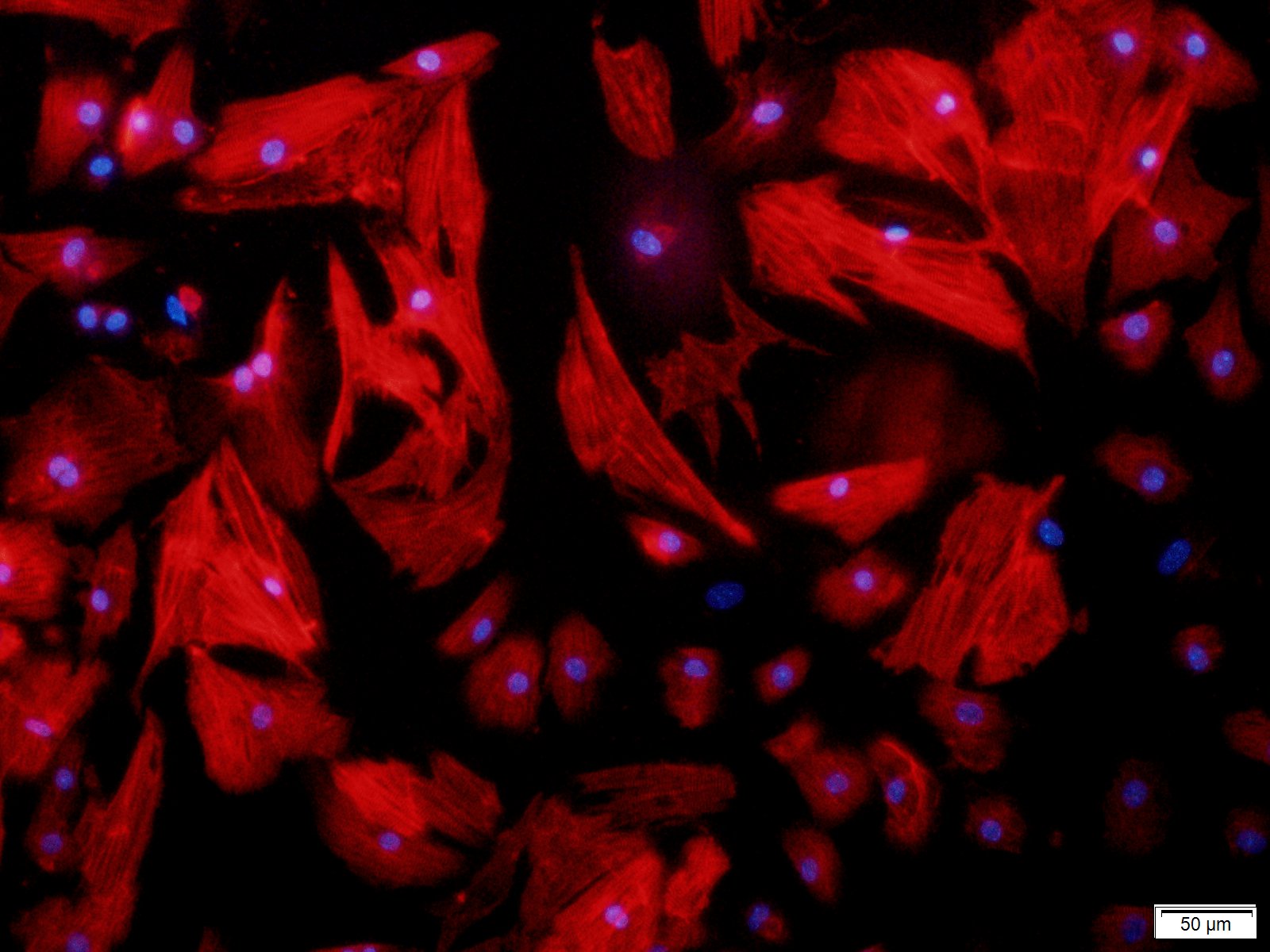

Supplement: Supplementary file 6 — Source data Fig. 3 [file 44321_2025_334_MOESM6_ESM.zip › Figure 3/3K/Ang II.jpg]

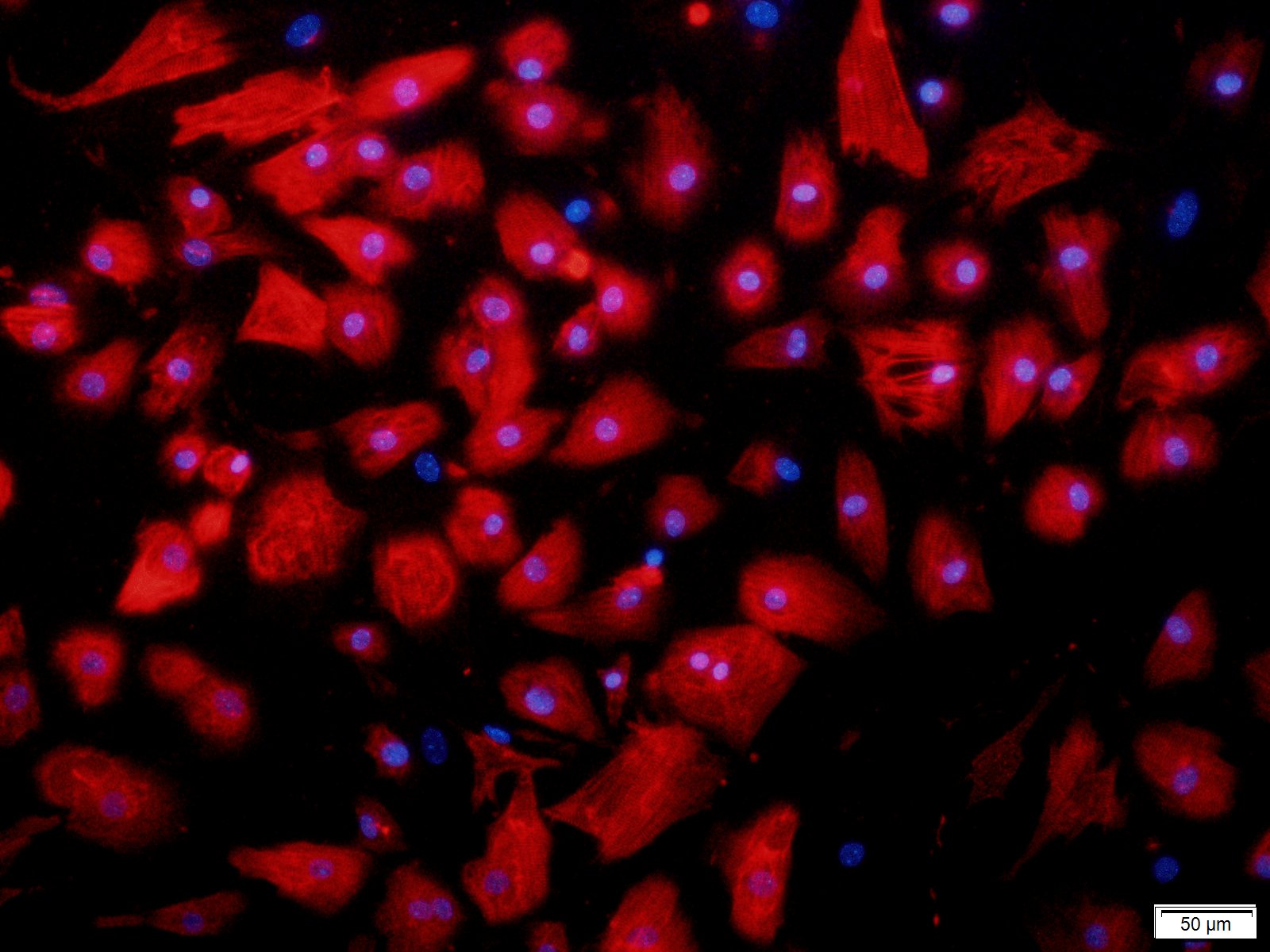

Supplement: Supplementary file 6 — Source data Fig. 3 [file 44321_2025_334_MOESM6_ESM.zip › Figure 3/3K/Control.jpg]

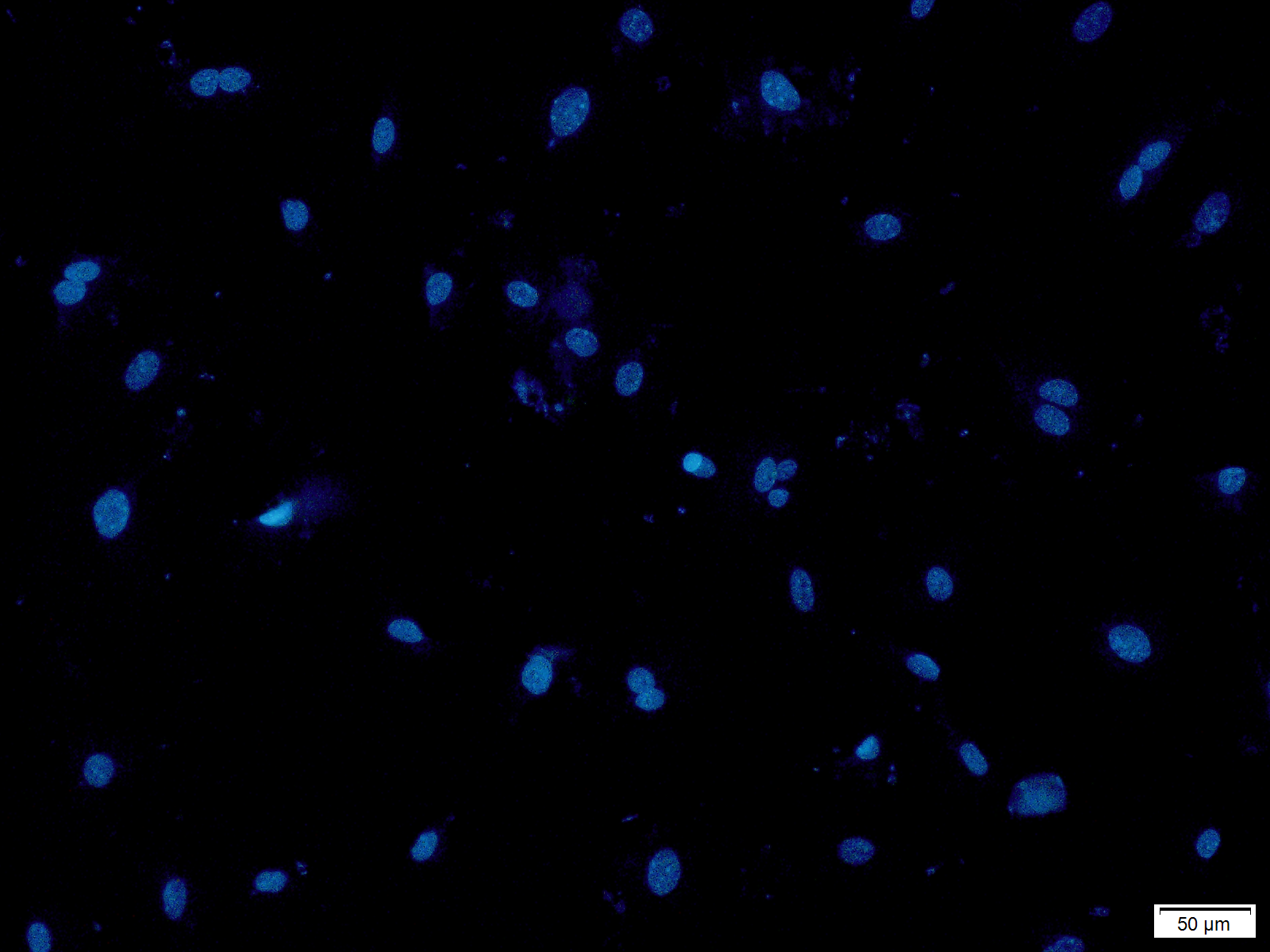

Supplement: Supplementary file 7 — Source data Fig. 4 [file 44321_2025_334_MOESM7_ESM.zip › Figure 4/4A/Ang II-DAPI.tif]

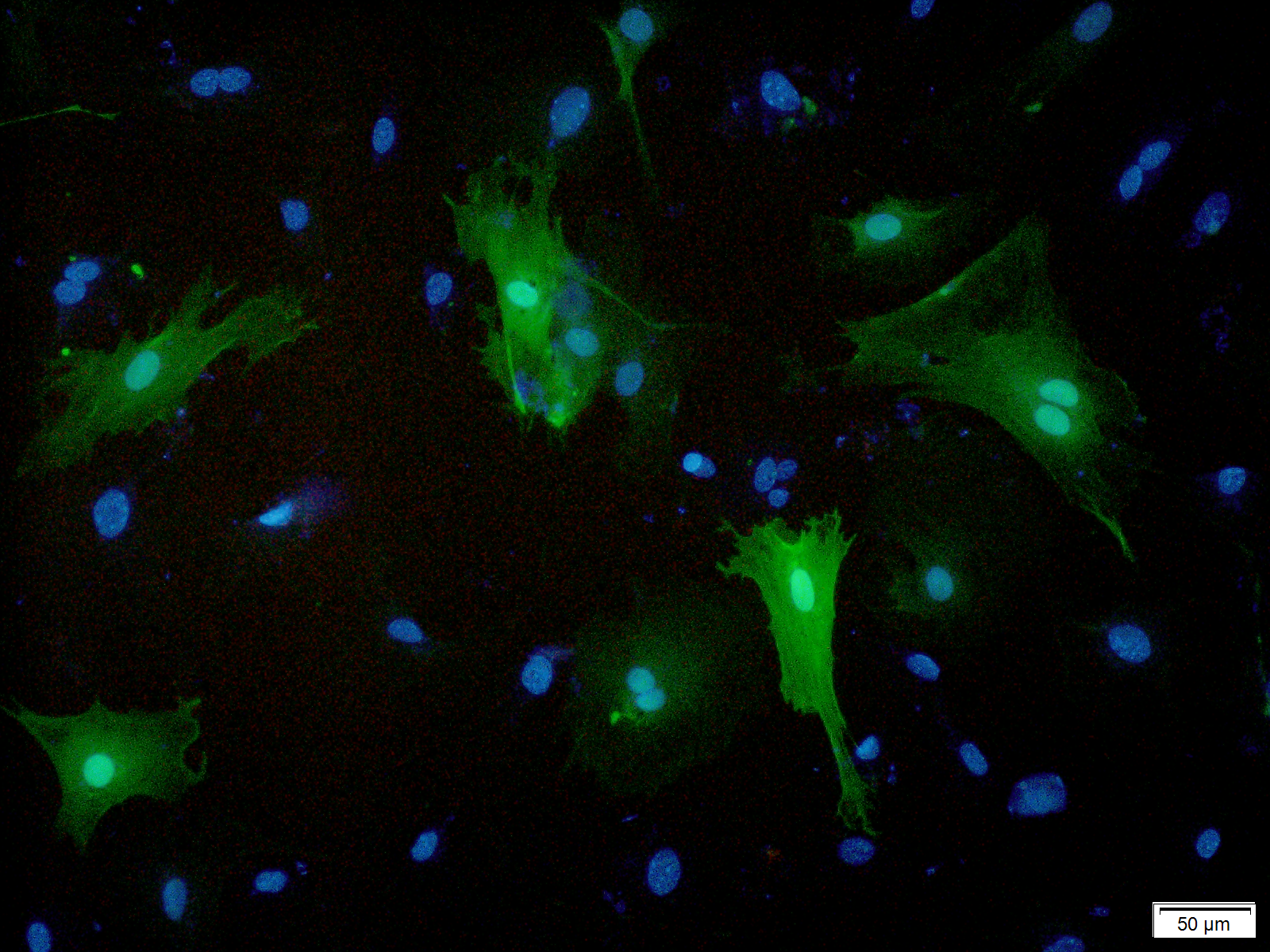

Supplement: Supplementary file 7 — Source data Fig. 4 [file 44321_2025_334_MOESM7_ESM.zip › Figure 4/4A/Ang II-Merge.tif]

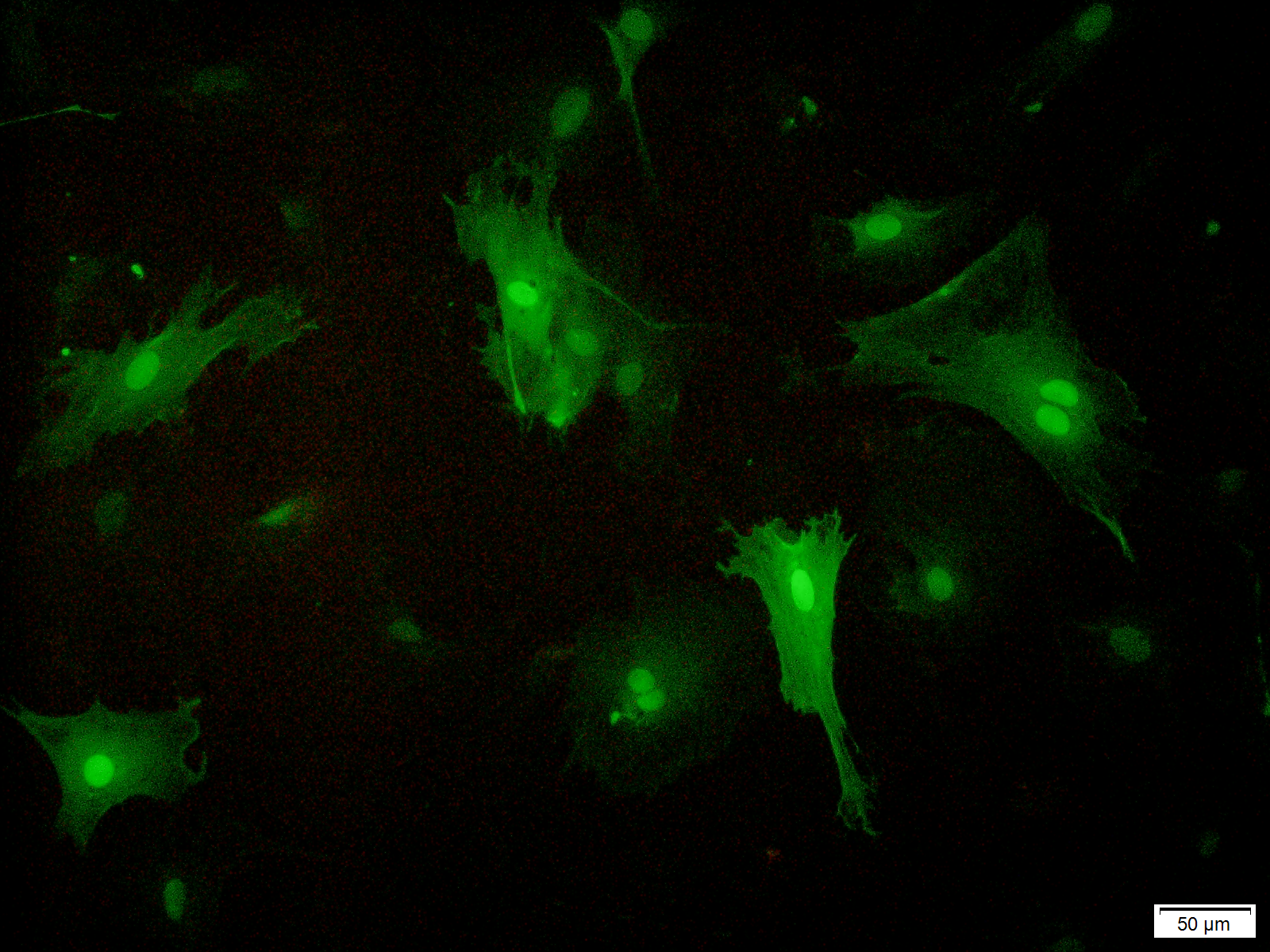

Supplement: Supplementary file 7 — Source data Fig. 4 [file 44321_2025_334_MOESM7_ESM.zip › Figure 4/4A/Ang II-RBMS1.tif]

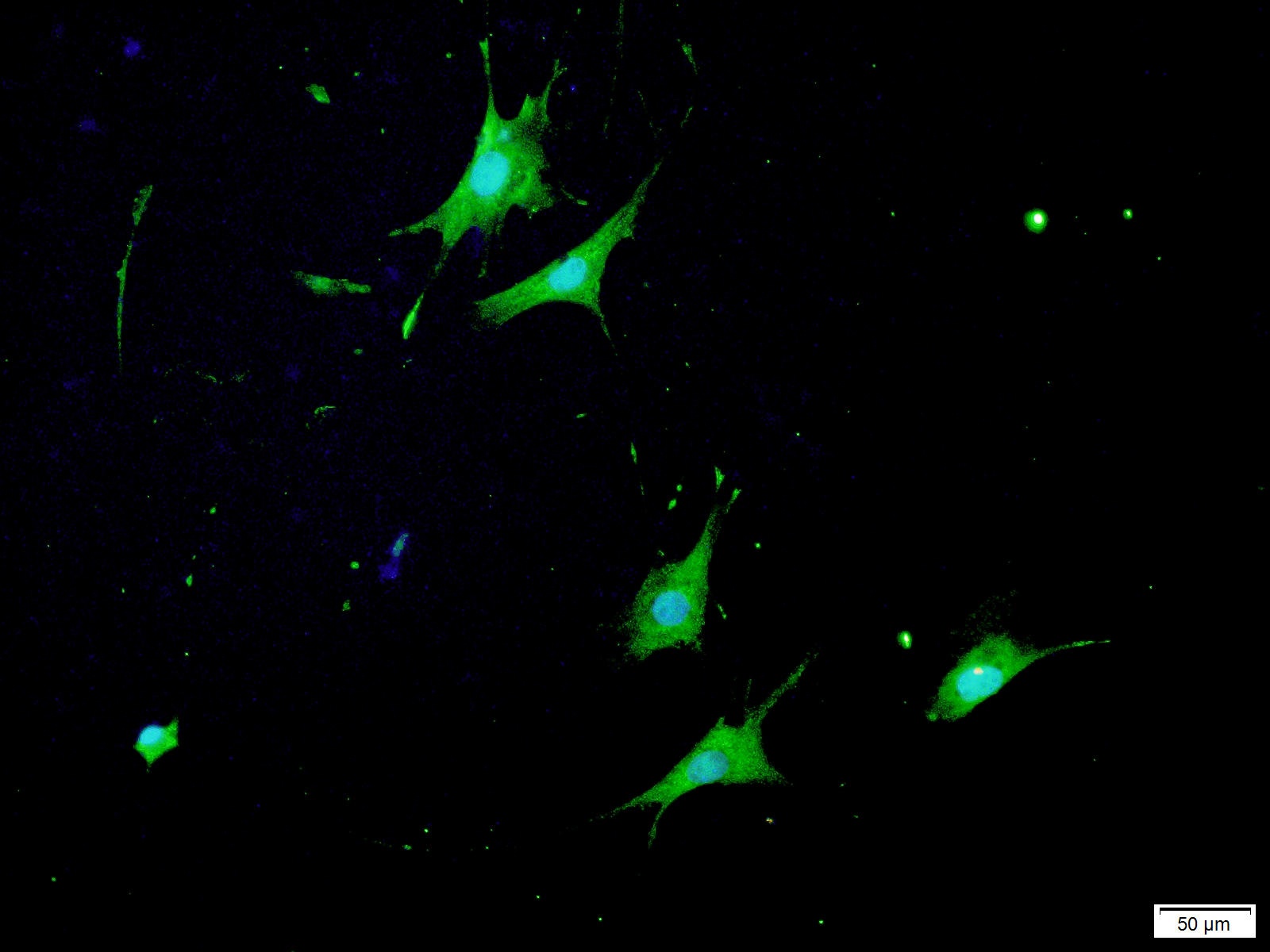

Supplement: Supplementary file 7 — Source data Fig. 4 [file 44321_2025_334_MOESM7_ESM.zip › Figure 4/4A/Contril-Merge.tif]

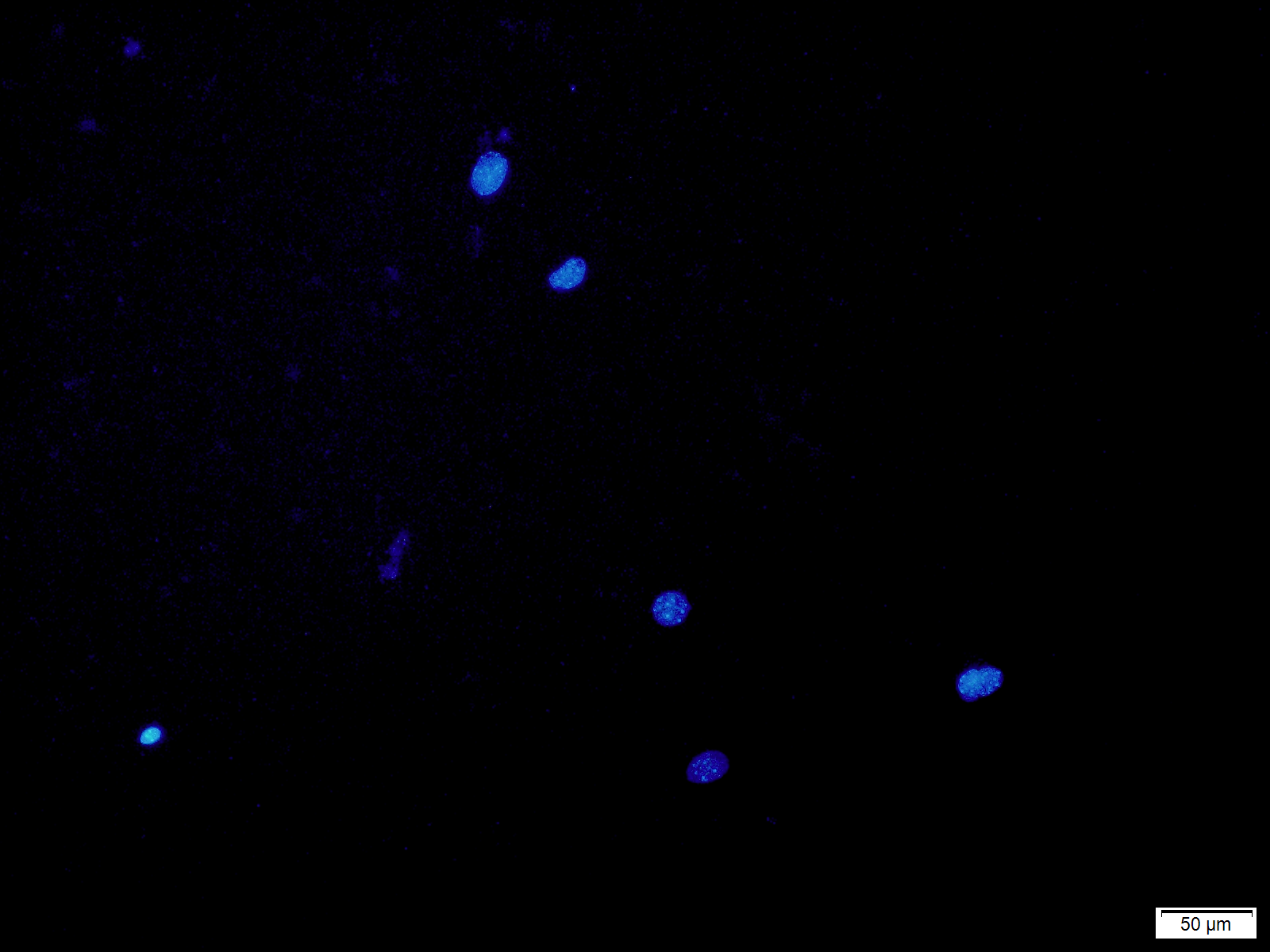

Supplement: Supplementary file 7 — Source data Fig. 4 [file 44321_2025_334_MOESM7_ESM.zip › Figure 4/4A/Control-DAPI.tif]

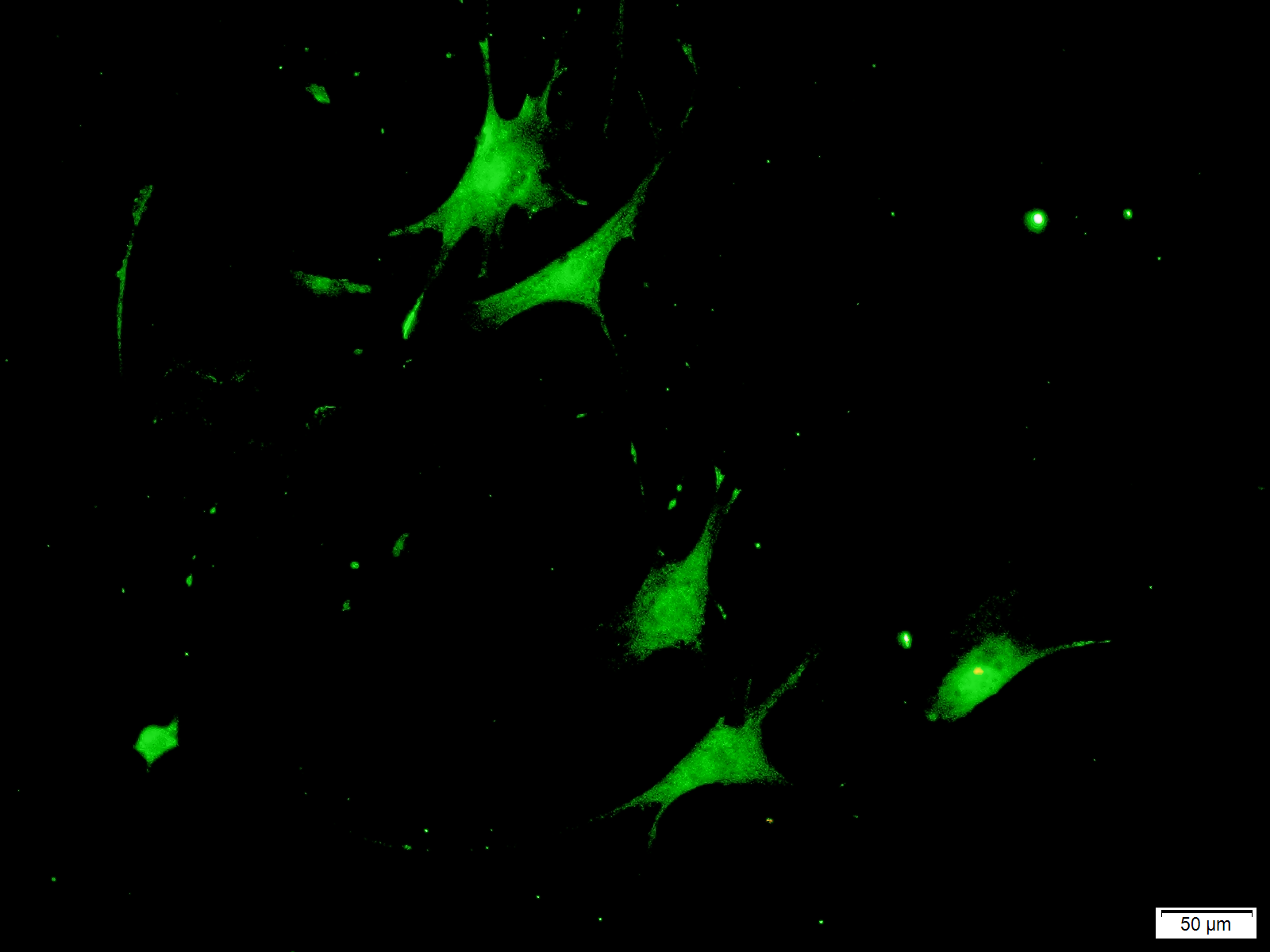

Supplement: Supplementary file 7 — Source data Fig. 4 [file 44321_2025_334_MOESM7_ESM.zip › Figure 4/4A/Control-RBMS1.tif]

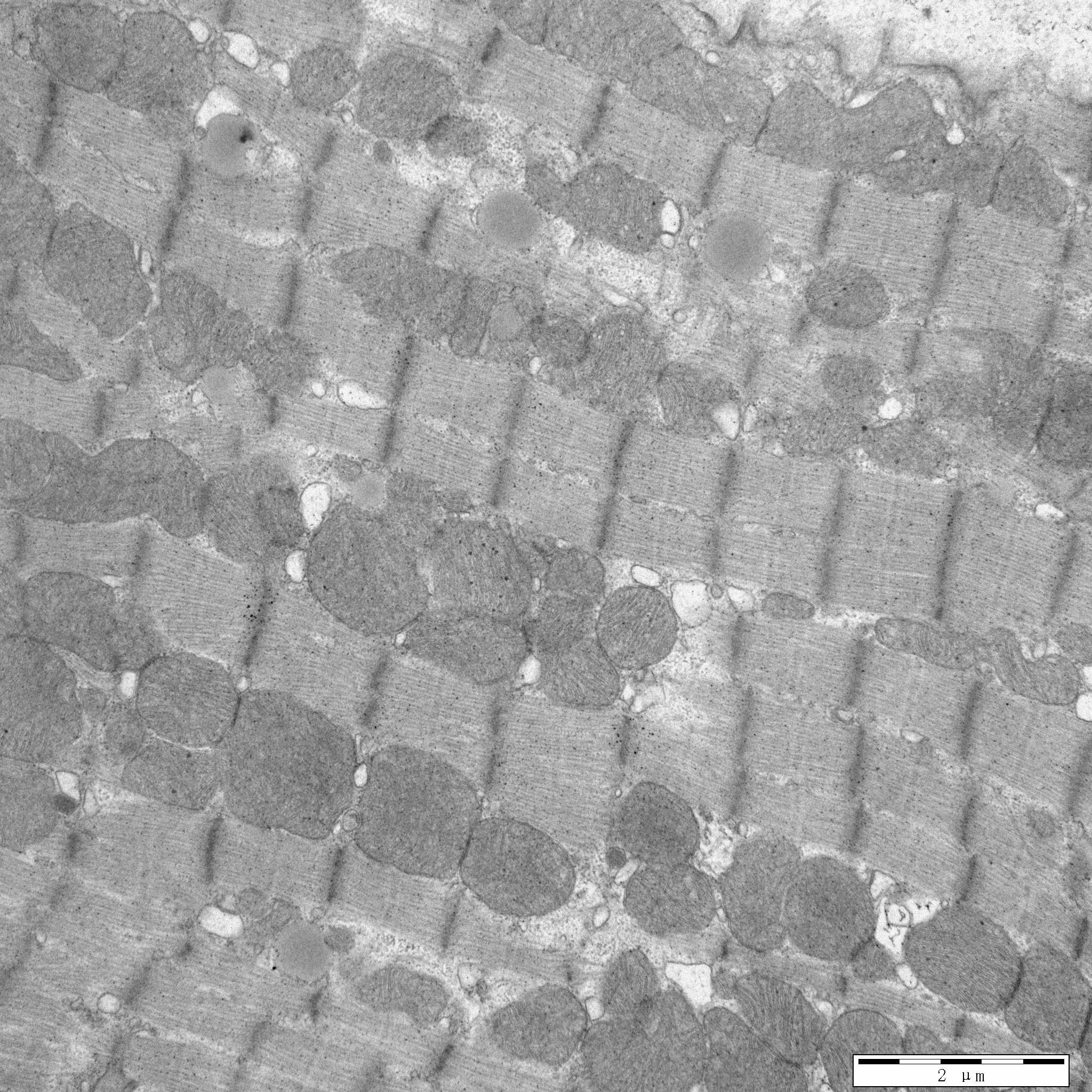

Supplement: Supplementary file 7 — Source data Fig. 4 [file 44321_2025_334_MOESM7_ESM.zip › Figure 4/4D/AAV9-RBMS1+Sham-1.JPG]

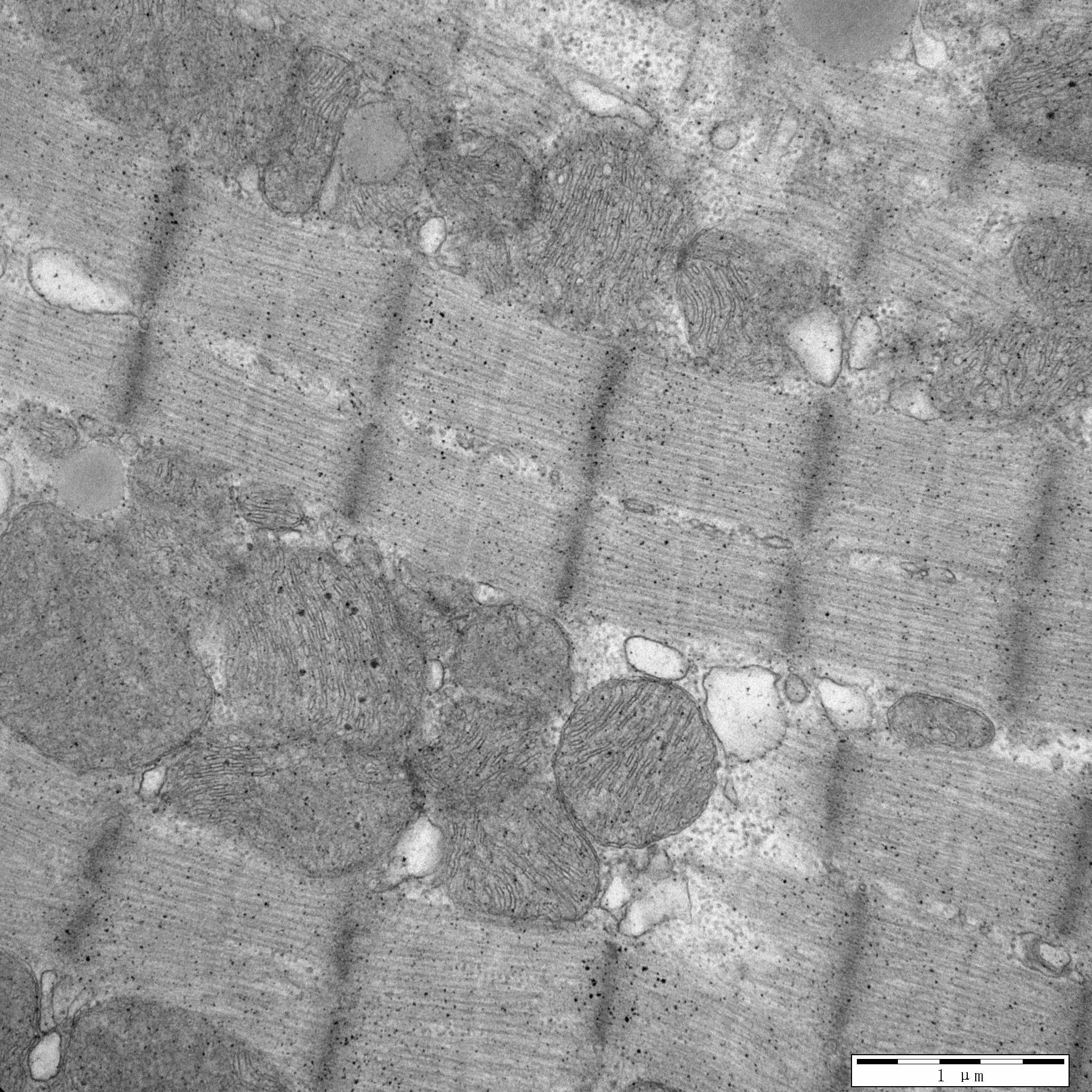

Supplement: Supplementary file 7 — Source data Fig. 4 [file 44321_2025_334_MOESM7_ESM.zip › Figure 4/4D/AAV9-RBMS1+Sham-2.JPG]

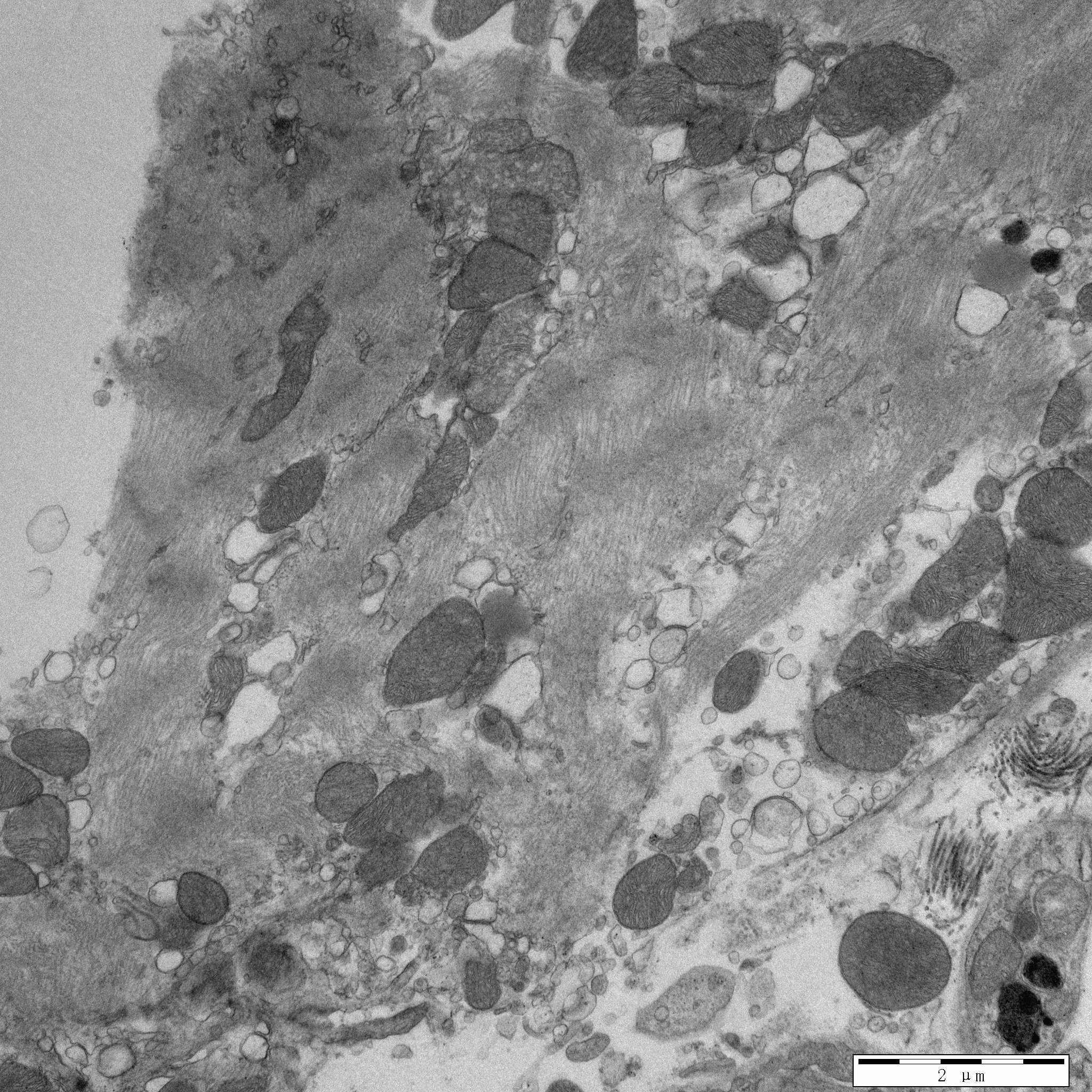

Supplement: Supplementary file 7 — Source data Fig. 4 [file 44321_2025_334_MOESM7_ESM.zip › Figure 4/4D/AAV9-RBMS1+TAC-1.JPG]

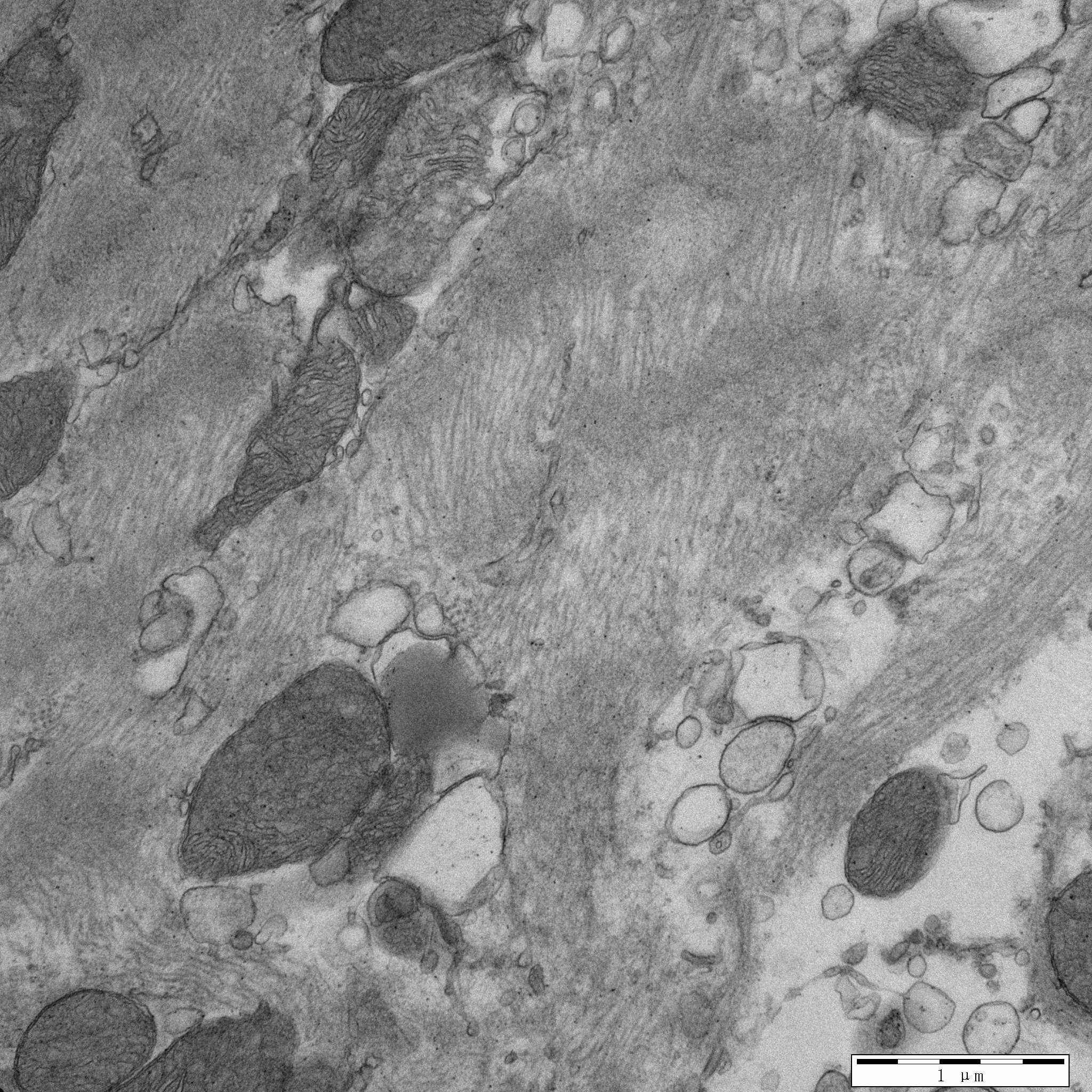

Supplement: Supplementary file 7 — Source data Fig. 4 [file 44321_2025_334_MOESM7_ESM.zip › Figure 4/4D/AAV9-RBMS1+TAC-2.JPG]

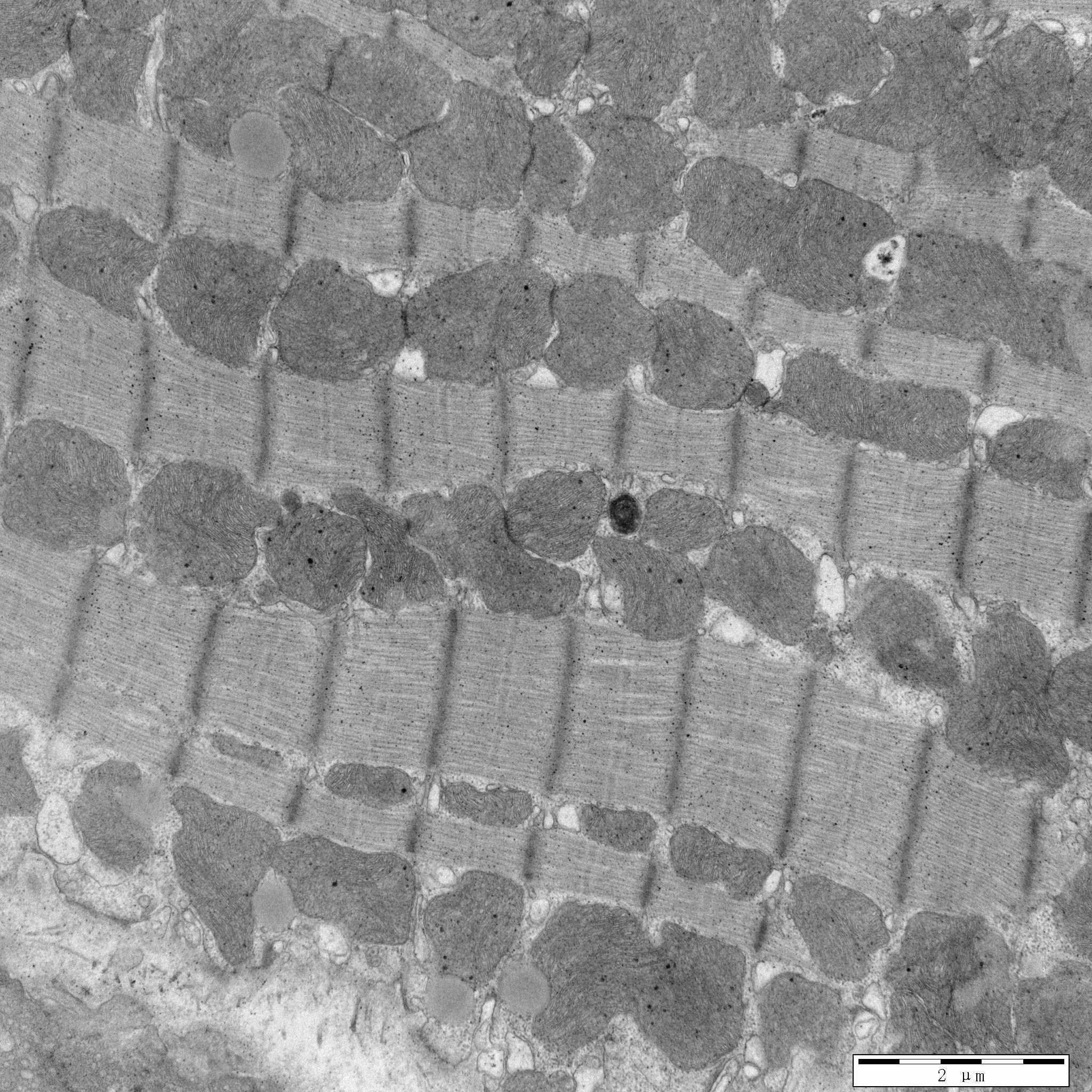

Supplement: Supplementary file 7 — Source data Fig. 4 [file 44321_2025_334_MOESM7_ESM.zip › Figure 4/4D/AAV9-Vector+Sham-1.JPG]

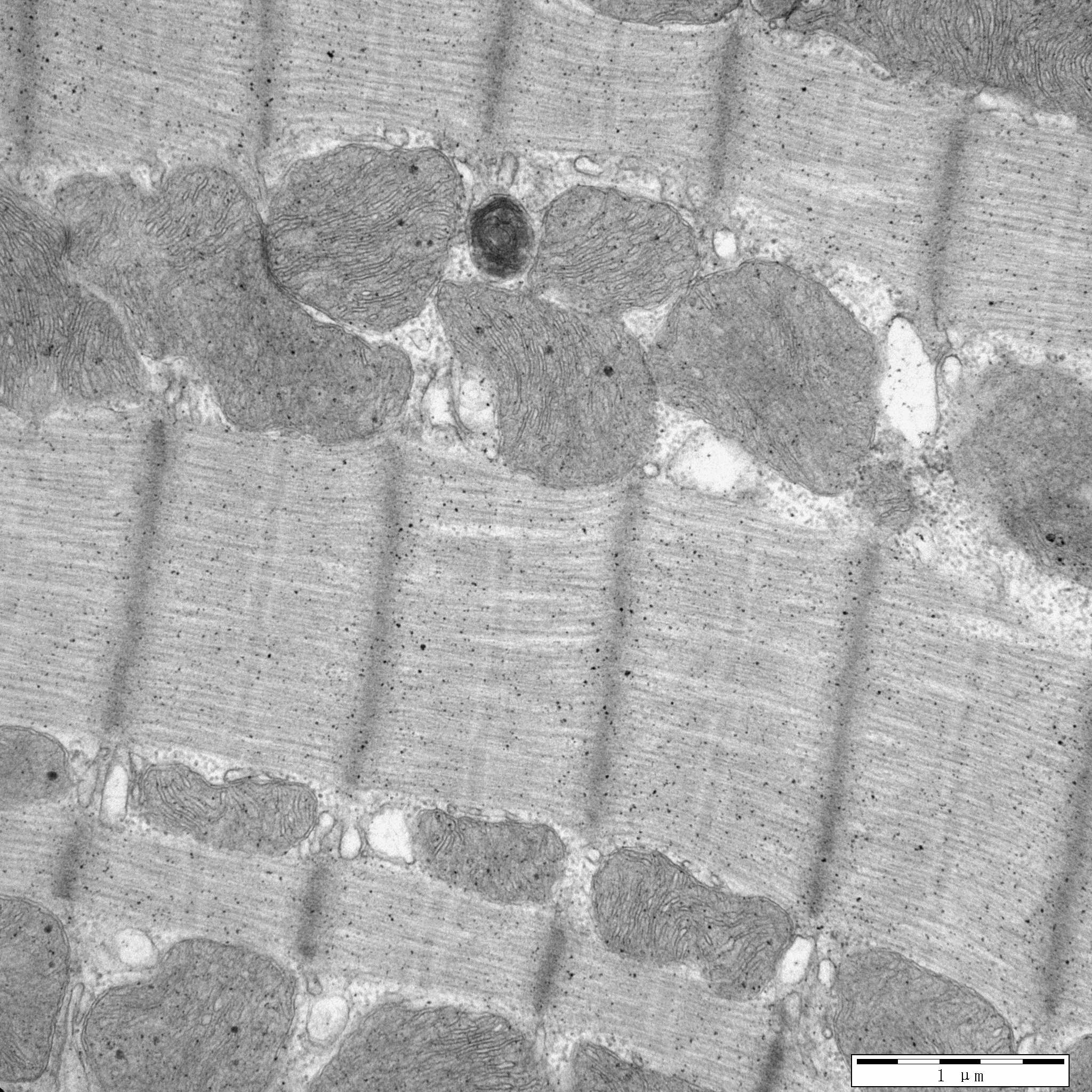

Supplement: Supplementary file 7 — Source data Fig. 4 [file 44321_2025_334_MOESM7_ESM.zip › Figure 4/4D/AAV9-Vector+Sham-2.JPG]

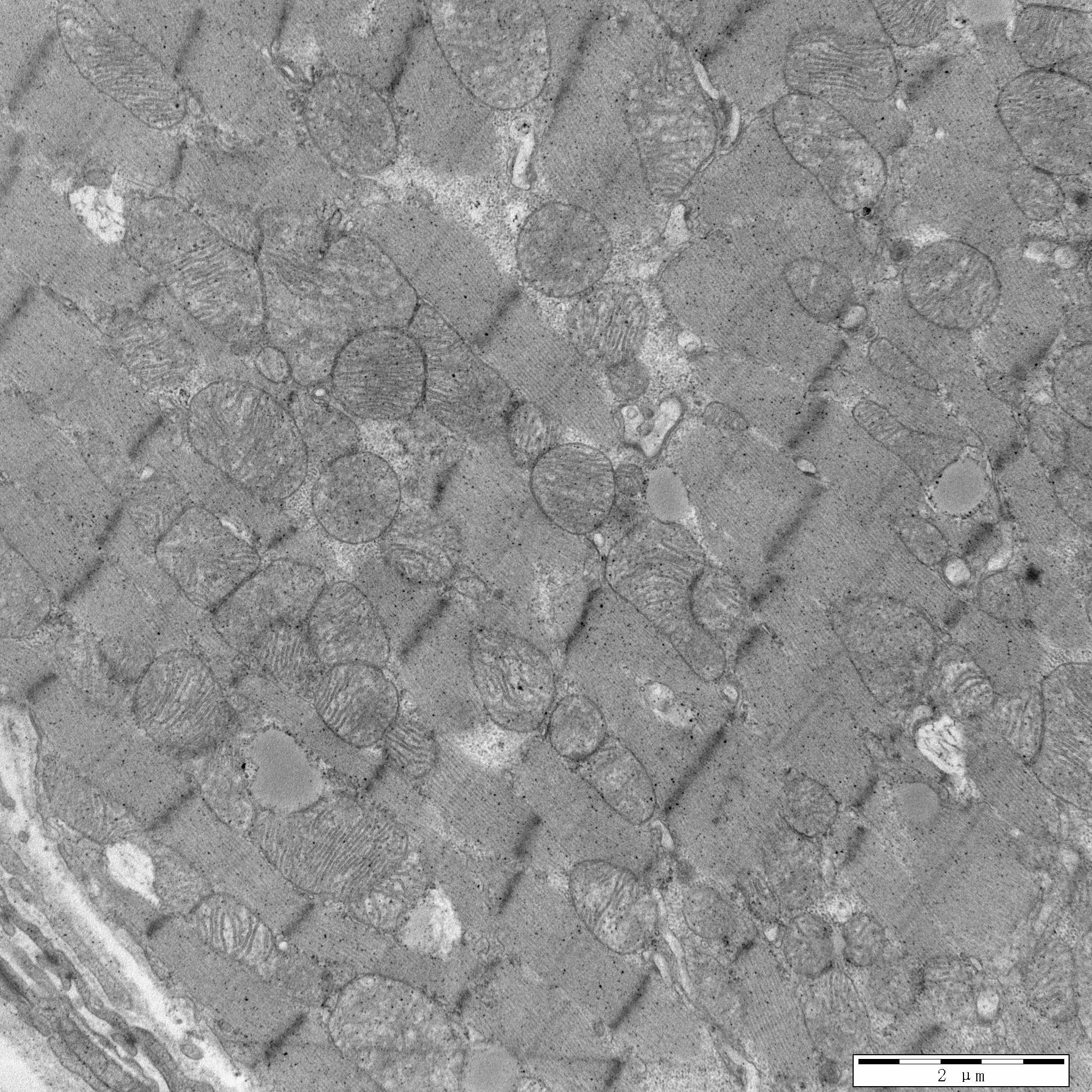

Supplement: Supplementary file 7 — Source data Fig. 4 [file 44321_2025_334_MOESM7_ESM.zip › Figure 4/4D/AAV9-Vector+TAC-1.JPG]

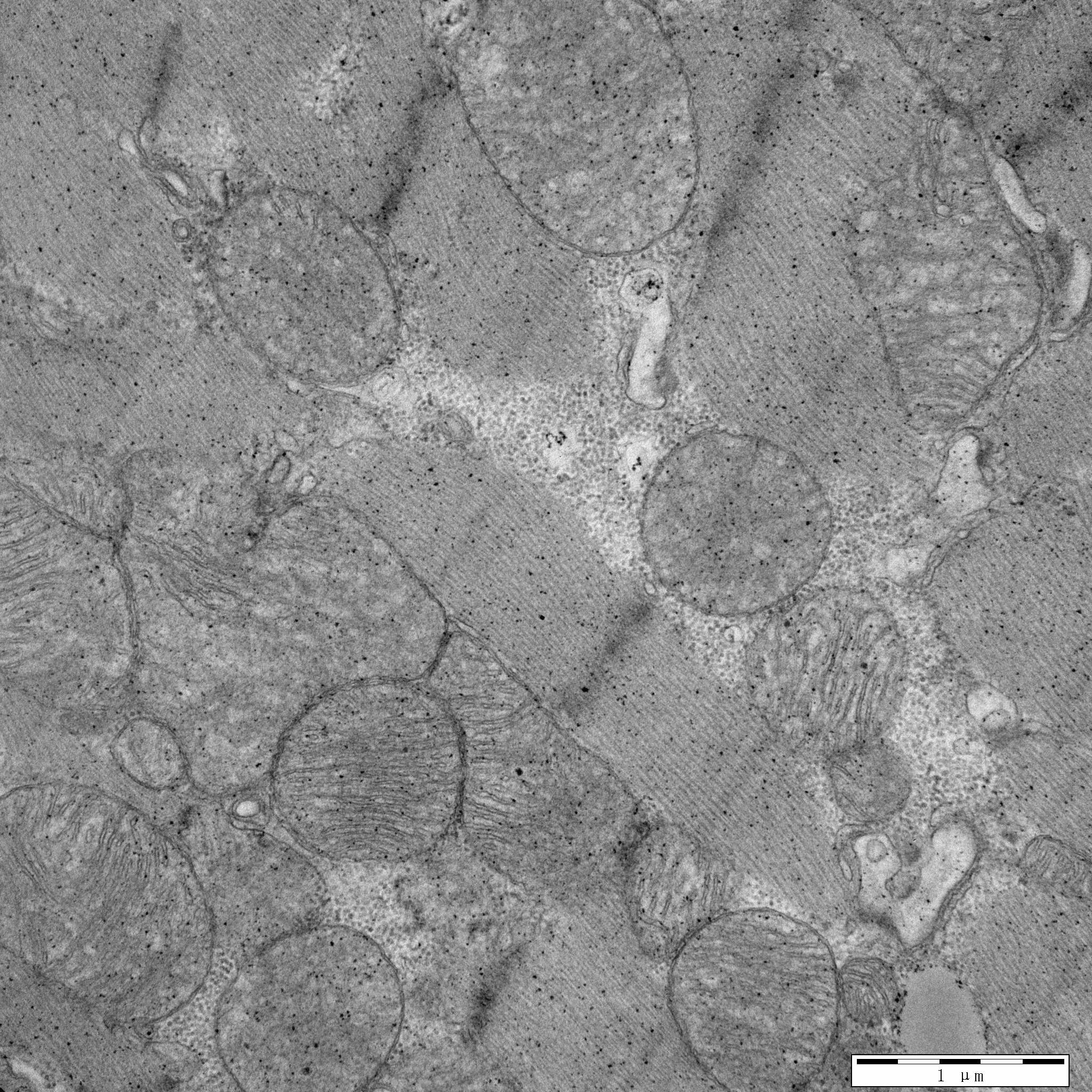

Supplement: Supplementary file 7 — Source data Fig. 4 [file 44321_2025_334_MOESM7_ESM.zip › Figure 4/4D/AAV9-Vector+TAC-2.JPG]

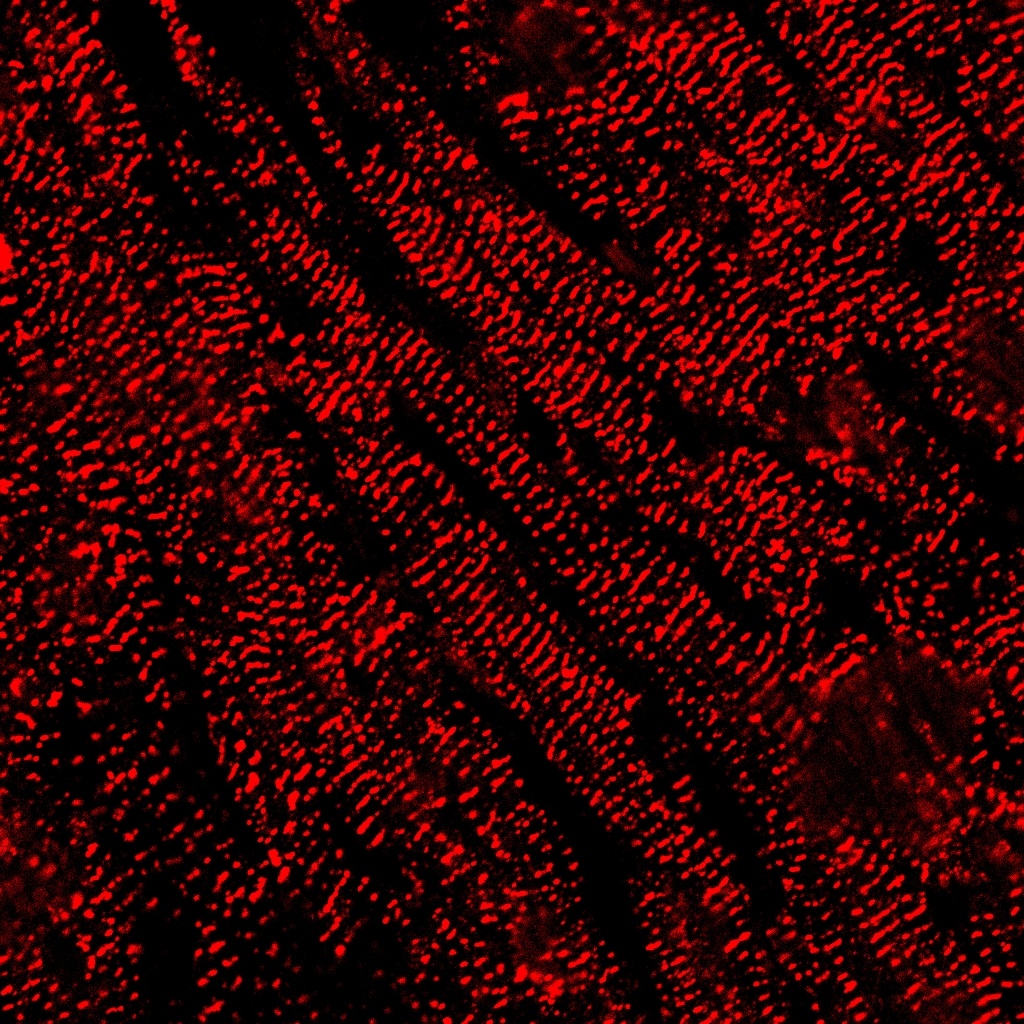

Supplement: Supplementary file 7 — Source data Fig. 4 [file 44321_2025_334_MOESM7_ESM.zip › Figure 4/4E/AAV9-RBMS1+Sham-ACTN2.jpeg]

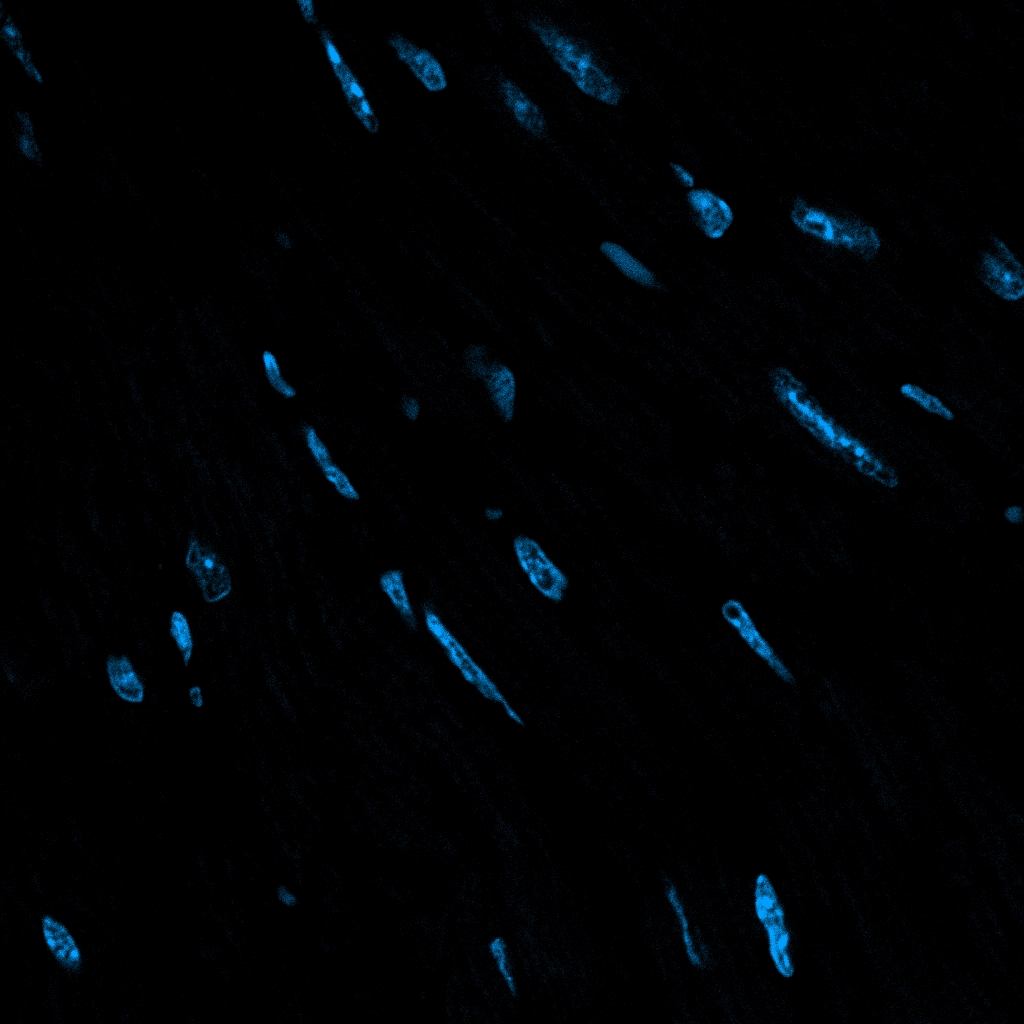

Supplement: Supplementary file 7 — Source data Fig. 4 [file 44321_2025_334_MOESM7_ESM.zip › Figure 4/4E/AAV9-RBMS1+Sham-DAPI.jpeg]

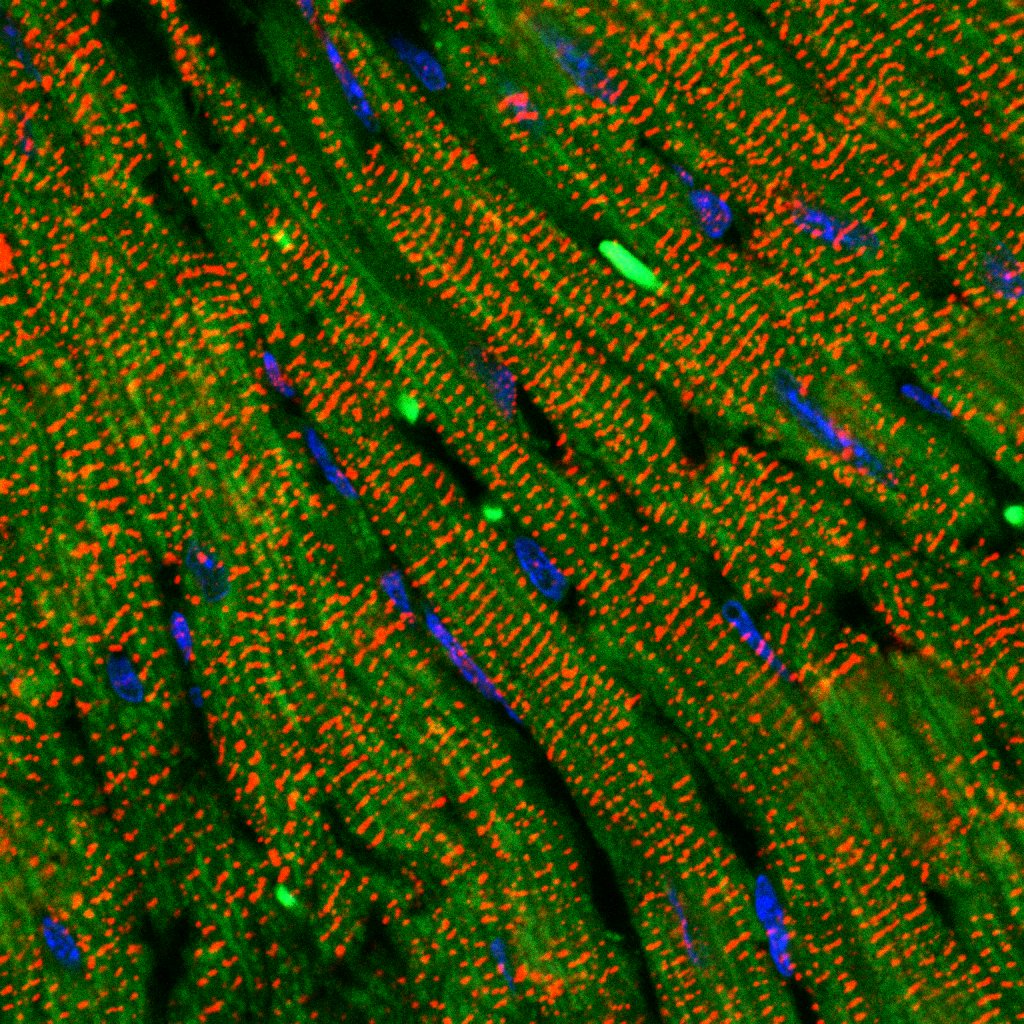

Supplement: Supplementary file 7 — Source data Fig. 4 [file 44321_2025_334_MOESM7_ESM.zip › Figure 4/4E/AAV9-RBMS1+Sham-Merge.jpg]

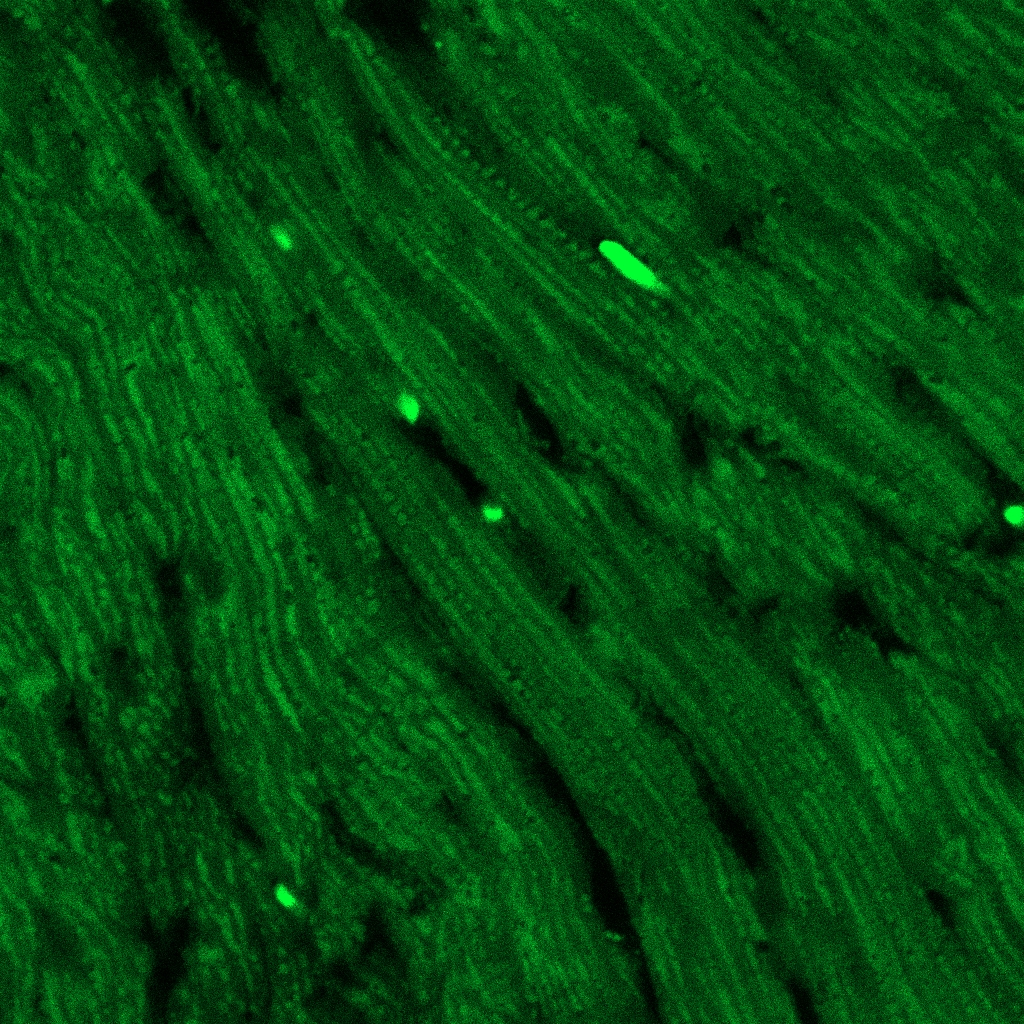

Supplement: Supplementary file 7 — Source data Fig. 4 [file 44321_2025_334_MOESM7_ESM.zip › Figure 4/4E/AAV9-RBMS1+Sham-α-ACTININ.jpeg]

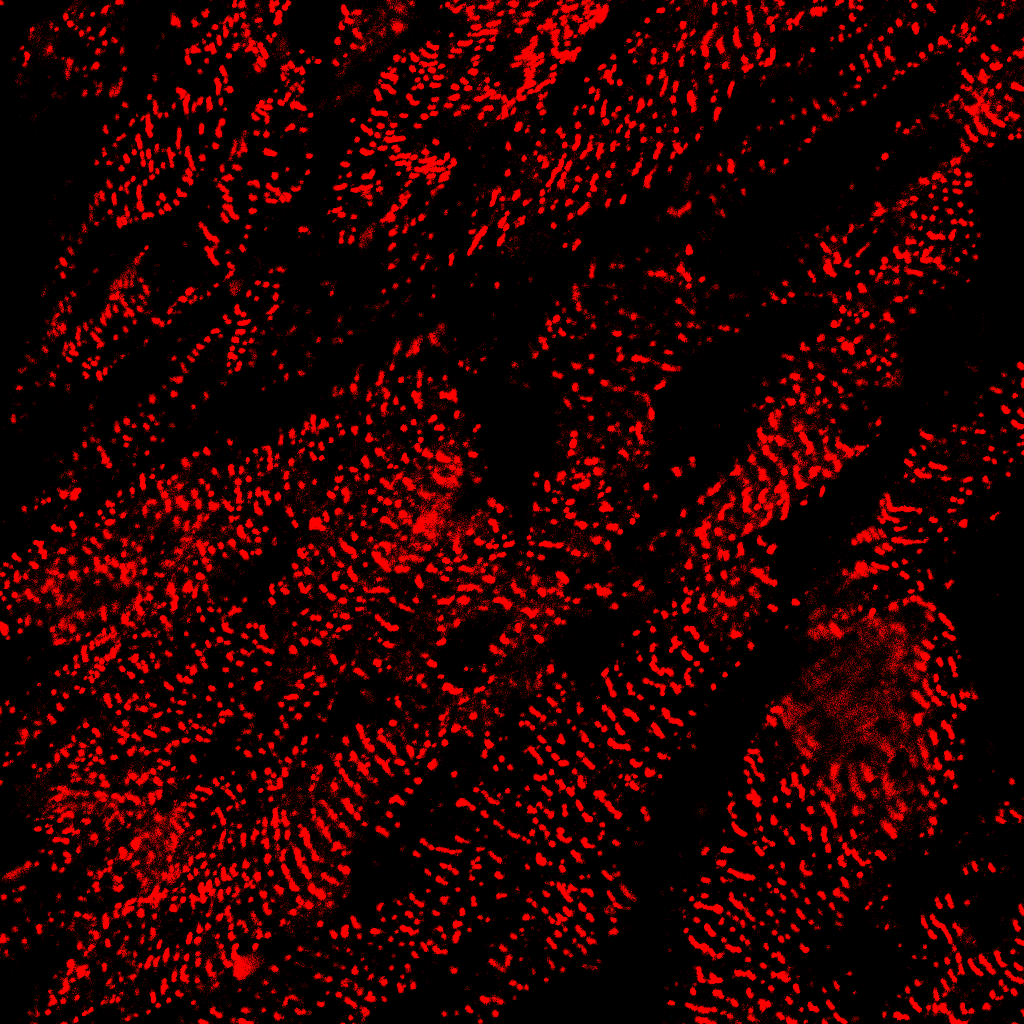

Supplement: Supplementary file 7 — Source data Fig. 4 [file 44321_2025_334_MOESM7_ESM.zip › Figure 4/4E/AAV9-RBMS1+TAC-ACTN2.tiff]

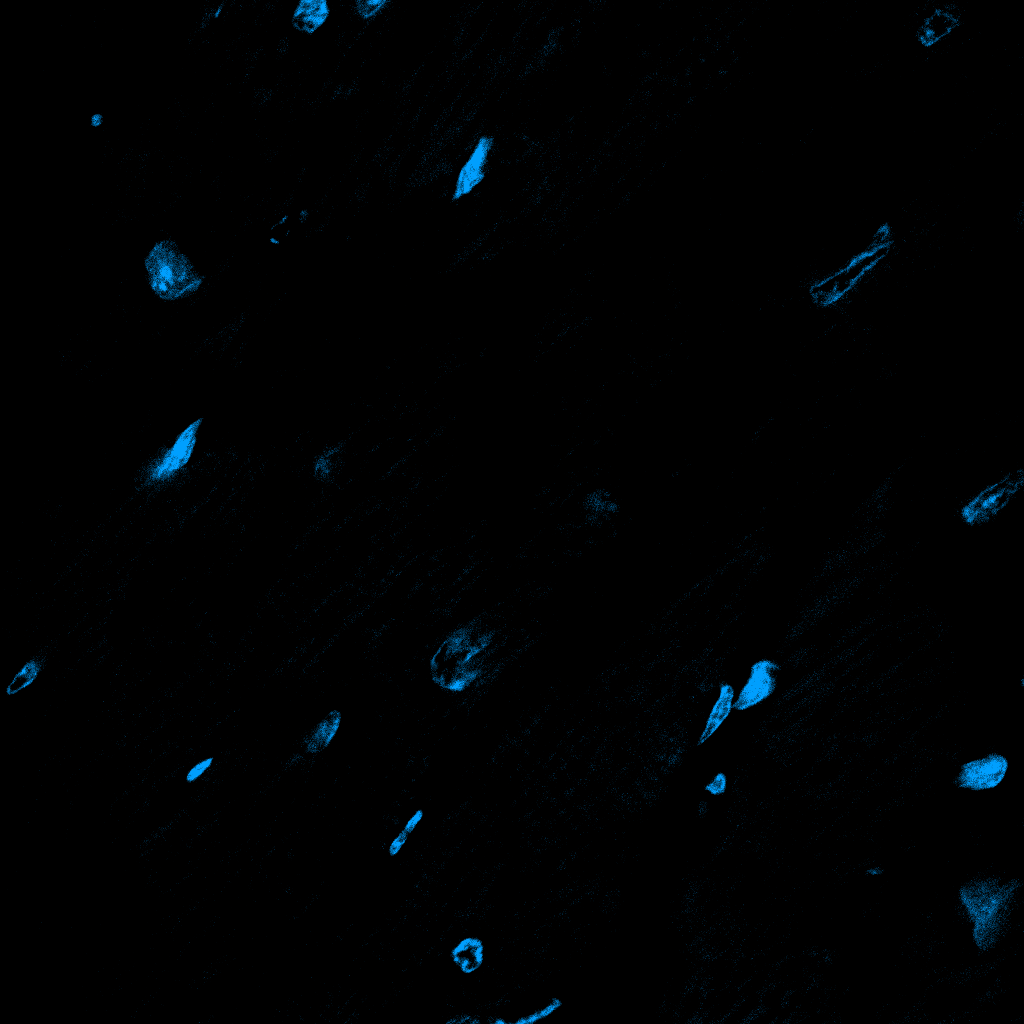

Supplement: Supplementary file 7 — Source data Fig. 4 [file 44321_2025_334_MOESM7_ESM.zip › Figure 4/4E/AAV9-RBMS1+TAC-DAPI.tiff]

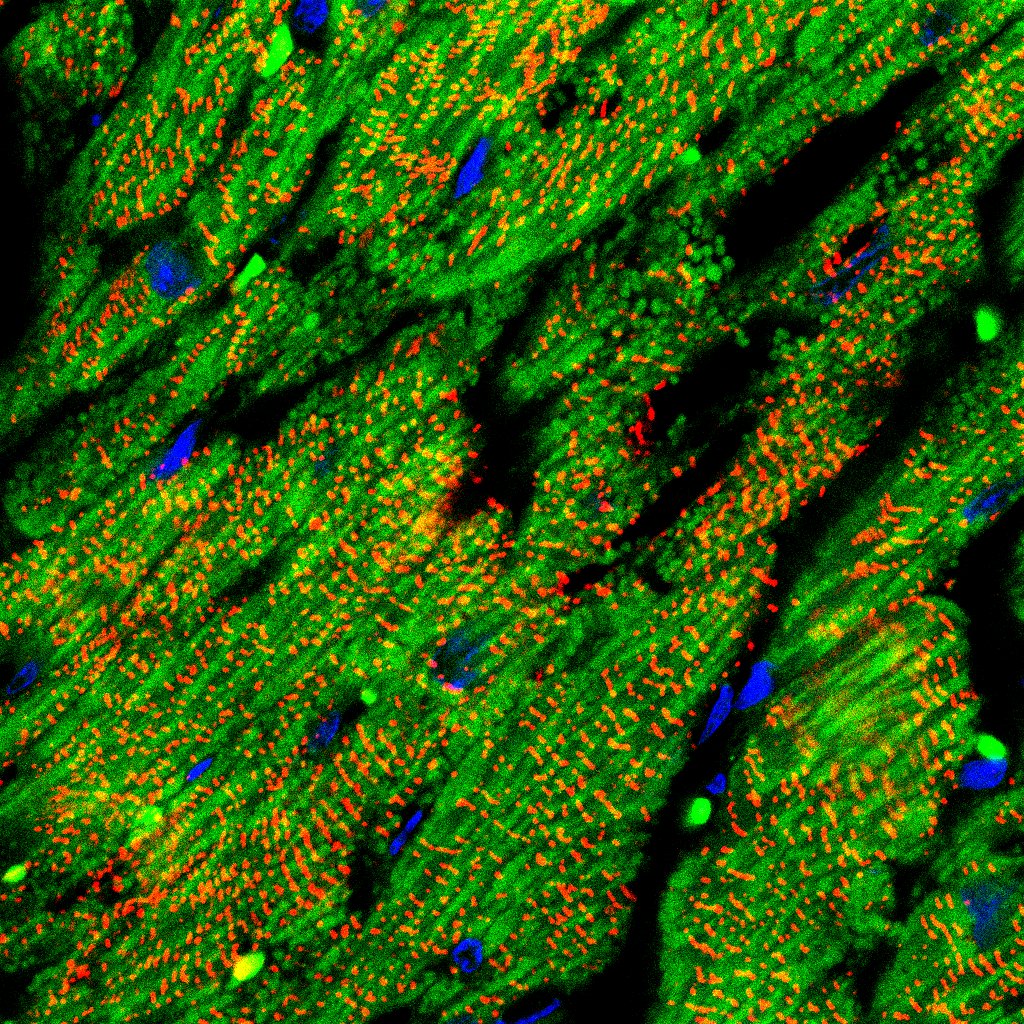

Supplement: Supplementary file 7 — Source data Fig. 4 [file 44321_2025_334_MOESM7_ESM.zip › Figure 4/4E/AAV9-RBMS1+TAC-Merge.jpg]

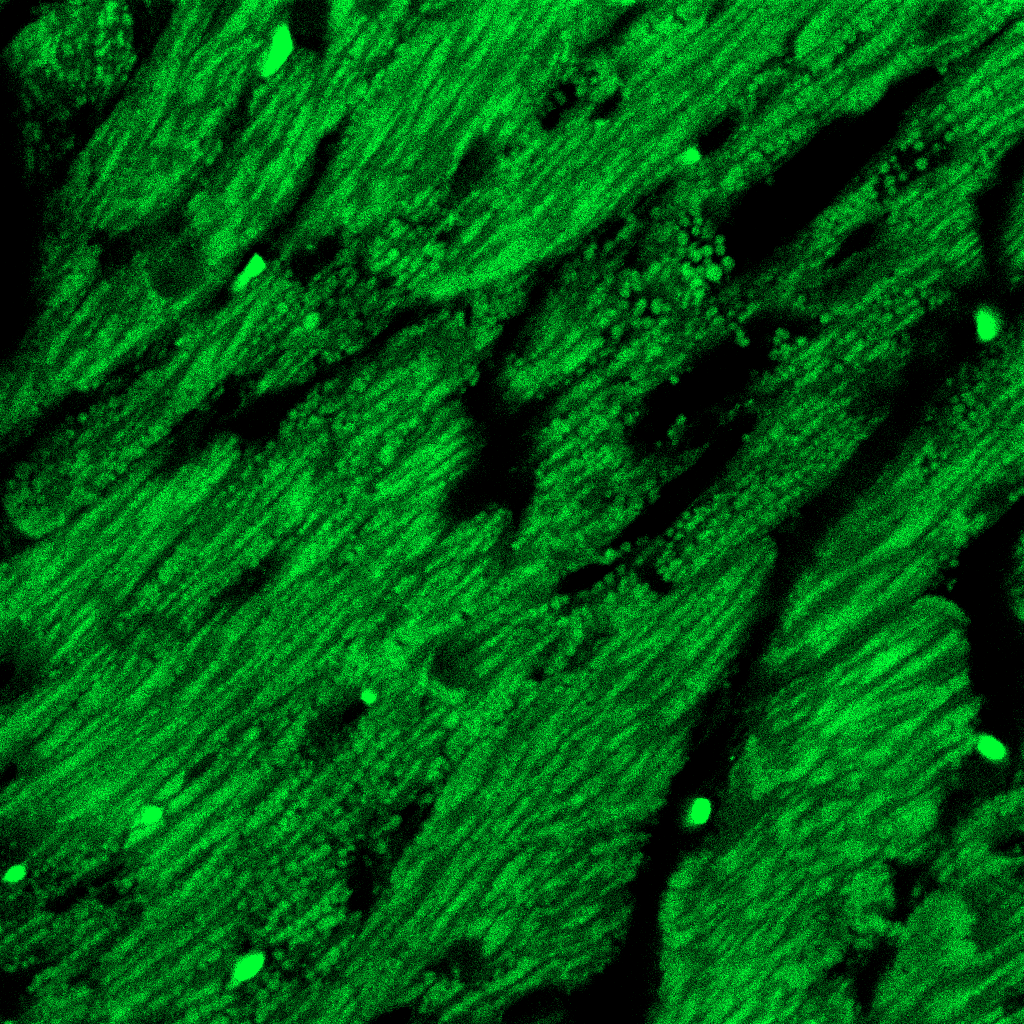

Supplement: Supplementary file 7 — Source data Fig. 4 [file 44321_2025_334_MOESM7_ESM.zip › Figure 4/4E/AAV9-RBMS1+TAC-α-ACTININ.tiff]

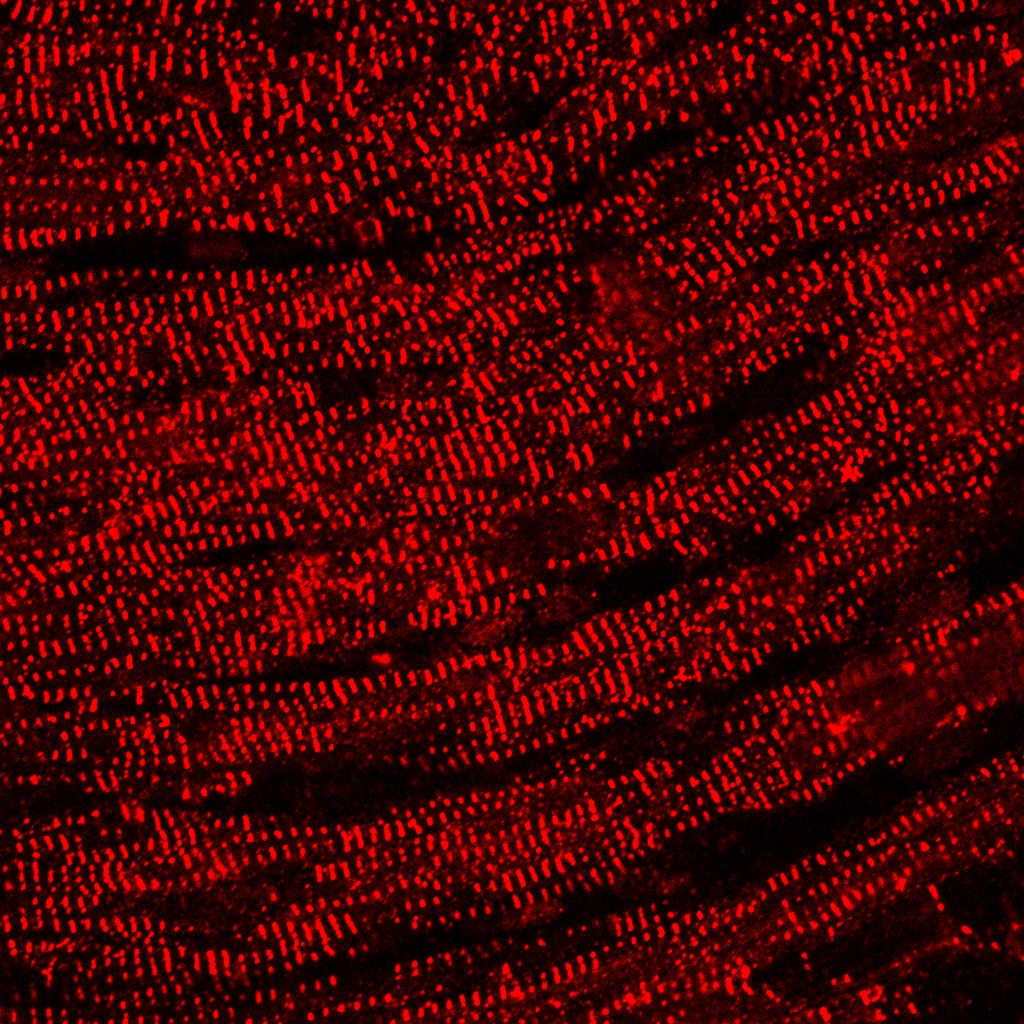

Supplement: Supplementary file 7 — Source data Fig. 4 [file 44321_2025_334_MOESM7_ESM.zip › Figure 4/4E/AAV9-Vector+Sham-ACTN2.jpeg]

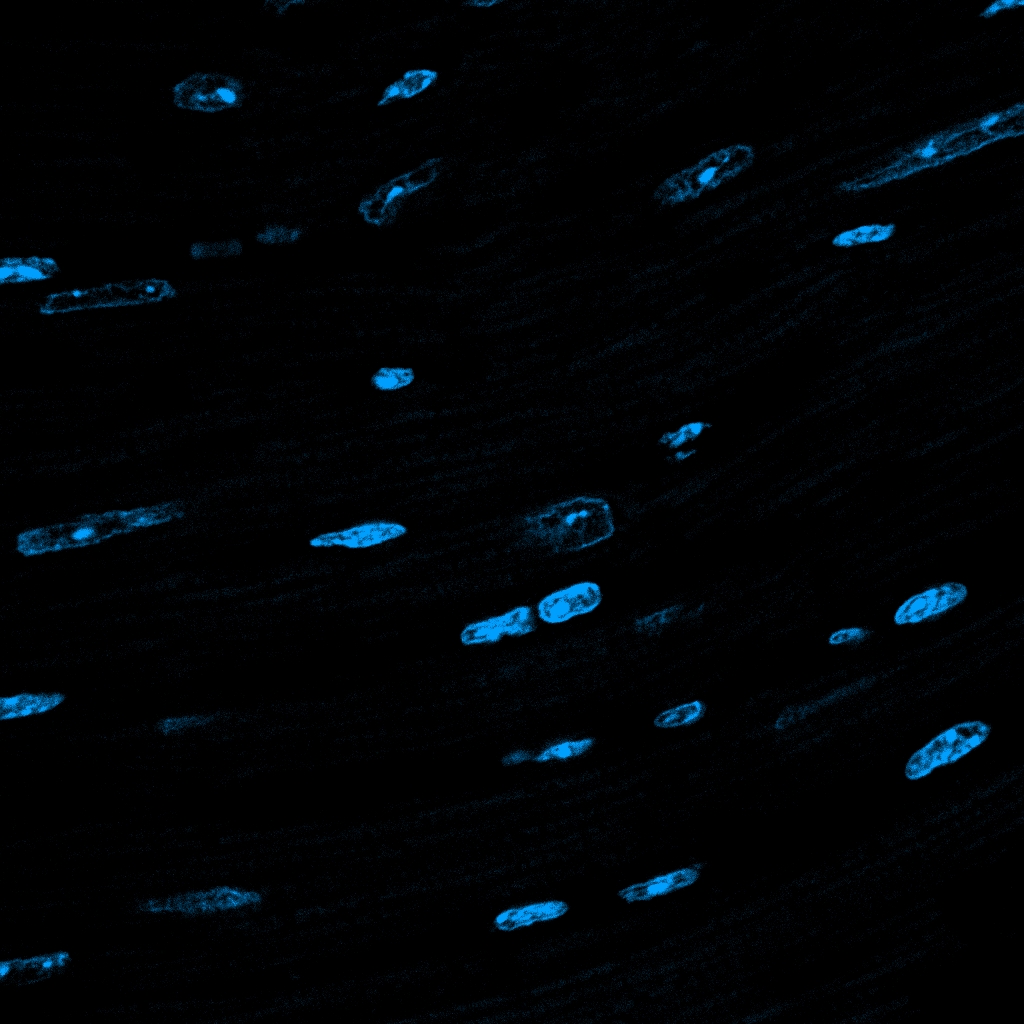

Supplement: Supplementary file 7 — Source data Fig. 4 [file 44321_2025_334_MOESM7_ESM.zip › Figure 4/4E/AAV9-Vector+Sham-DAPI.jpeg]

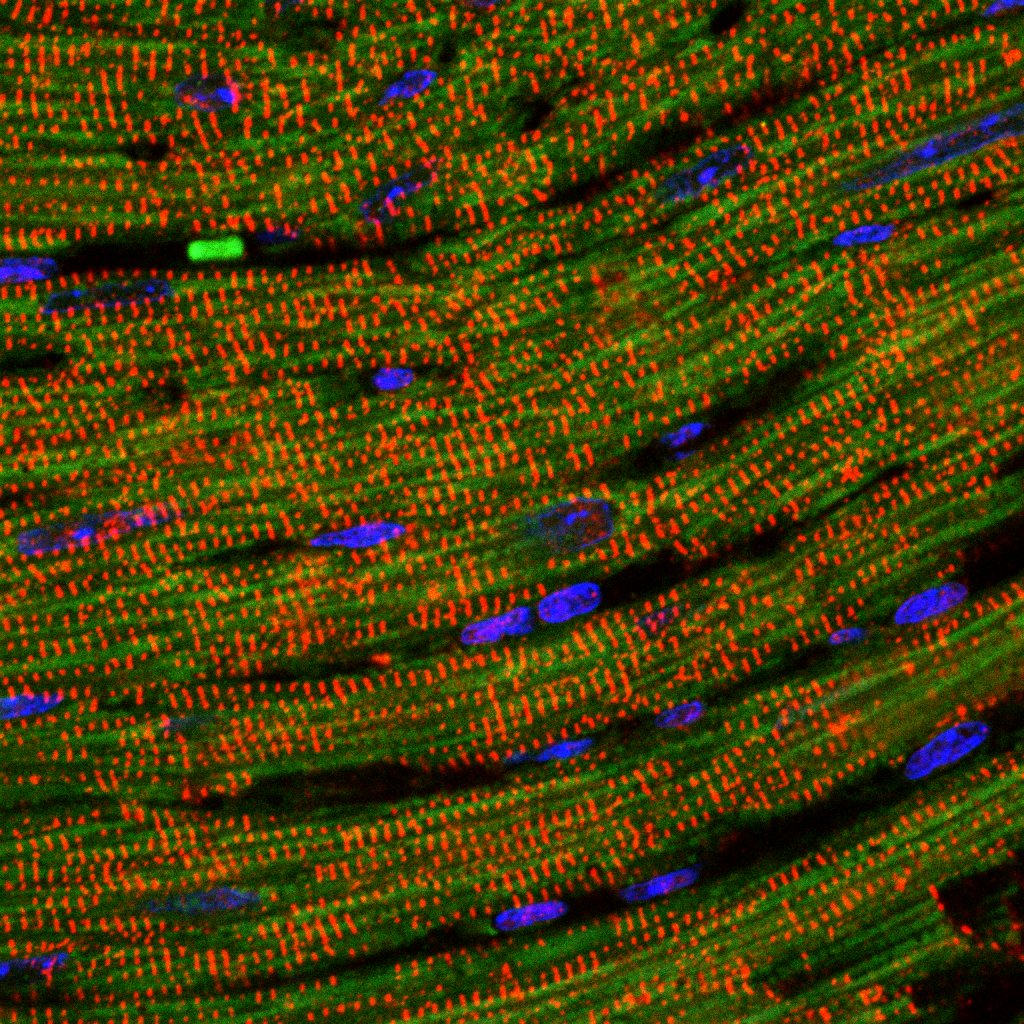

Supplement: Supplementary file 7 — Source data Fig. 4 [file 44321_2025_334_MOESM7_ESM.zip › Figure 4/4E/AAV9-Vector+Sham-Merge.jpg]

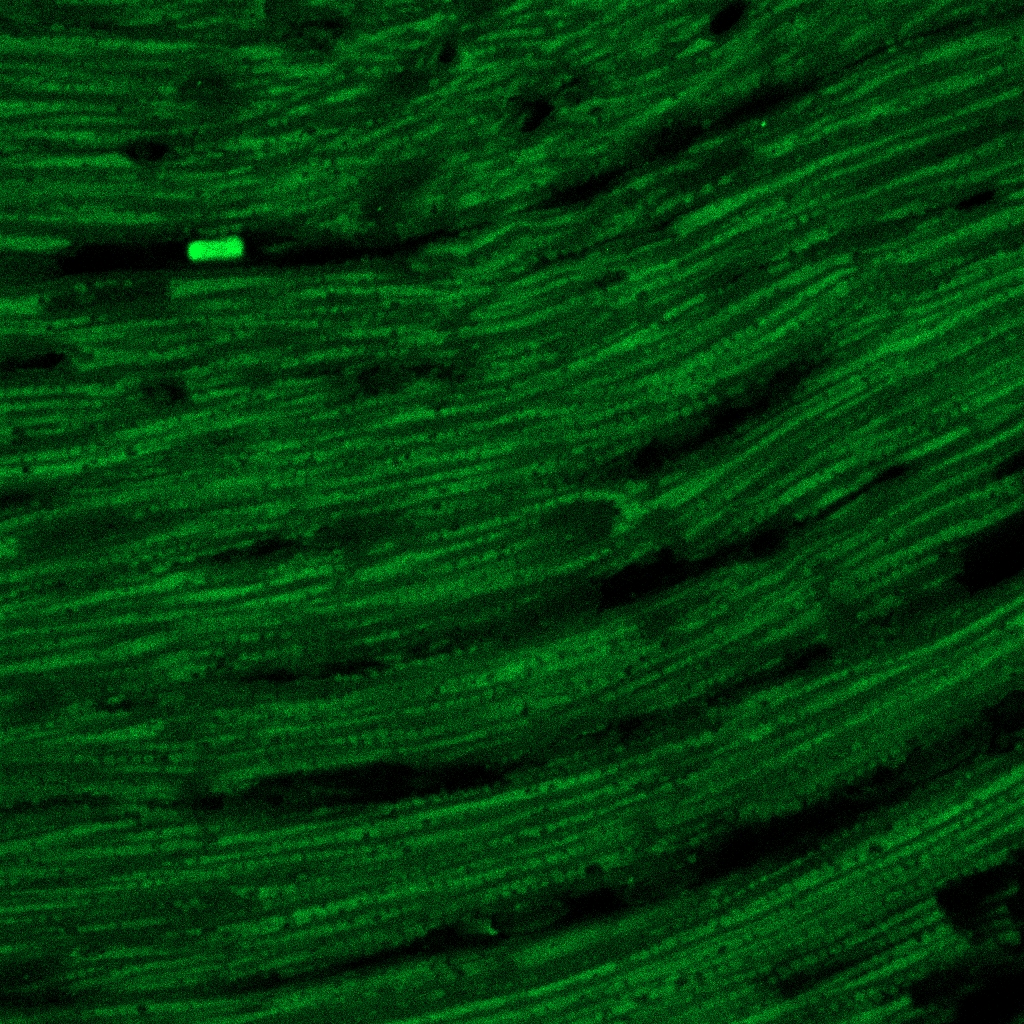

Supplement: Supplementary file 7 — Source data Fig. 4 [file 44321_2025_334_MOESM7_ESM.zip › Figure 4/4E/AAV9-Vector+Sham-α-ACTININ.jpeg]

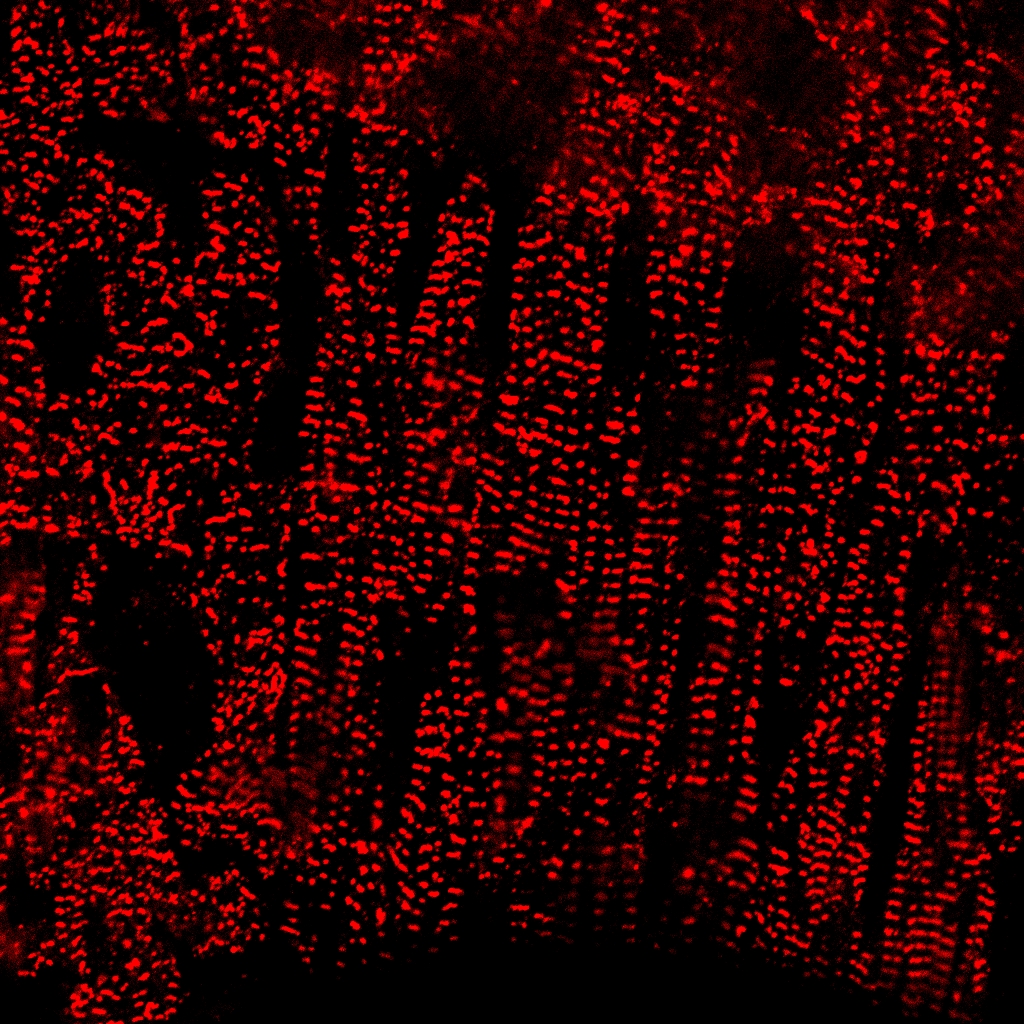

Supplement: Supplementary file 7 — Source data Fig. 4 [file 44321_2025_334_MOESM7_ESM.zip › Figure 4/4E/AAV9-Vector+TAC-ACTN2.jpeg]

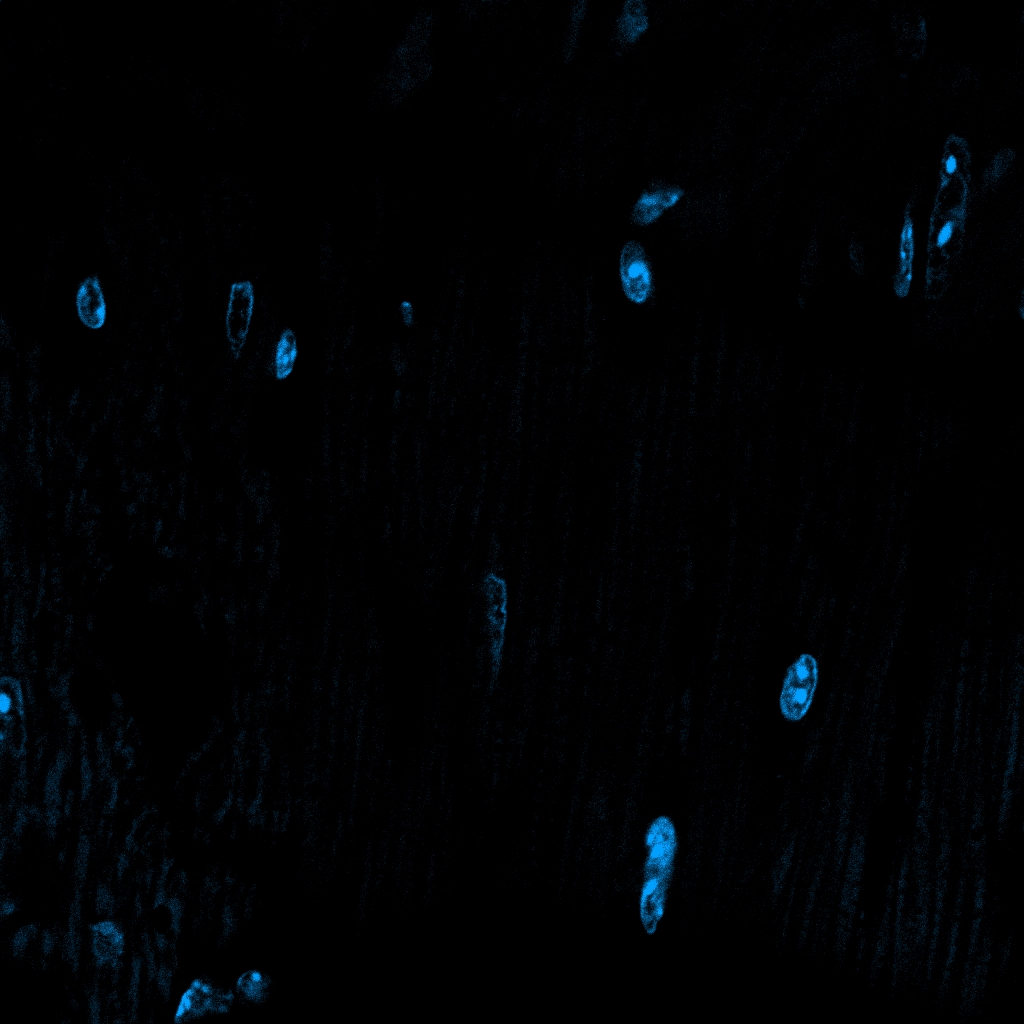

Supplement: Supplementary file 7 — Source data Fig. 4 [file 44321_2025_334_MOESM7_ESM.zip › Figure 4/4E/AAV9-Vector+TAC-DAPI.jpeg]
