# Supplementary figures and images for: RBMS1 orchestrates cardiac hypertrophy by facilitating CTTN splice-switching and sarcomere dynamics (part 2 of 4)
Source: EMBO Mol Med. 2025 Nov 10;17(12):3555–85. doi: 10.1038/s44321-025-00334-z (PMC12686484; doi:10.1038/s44321-025-00334-z)

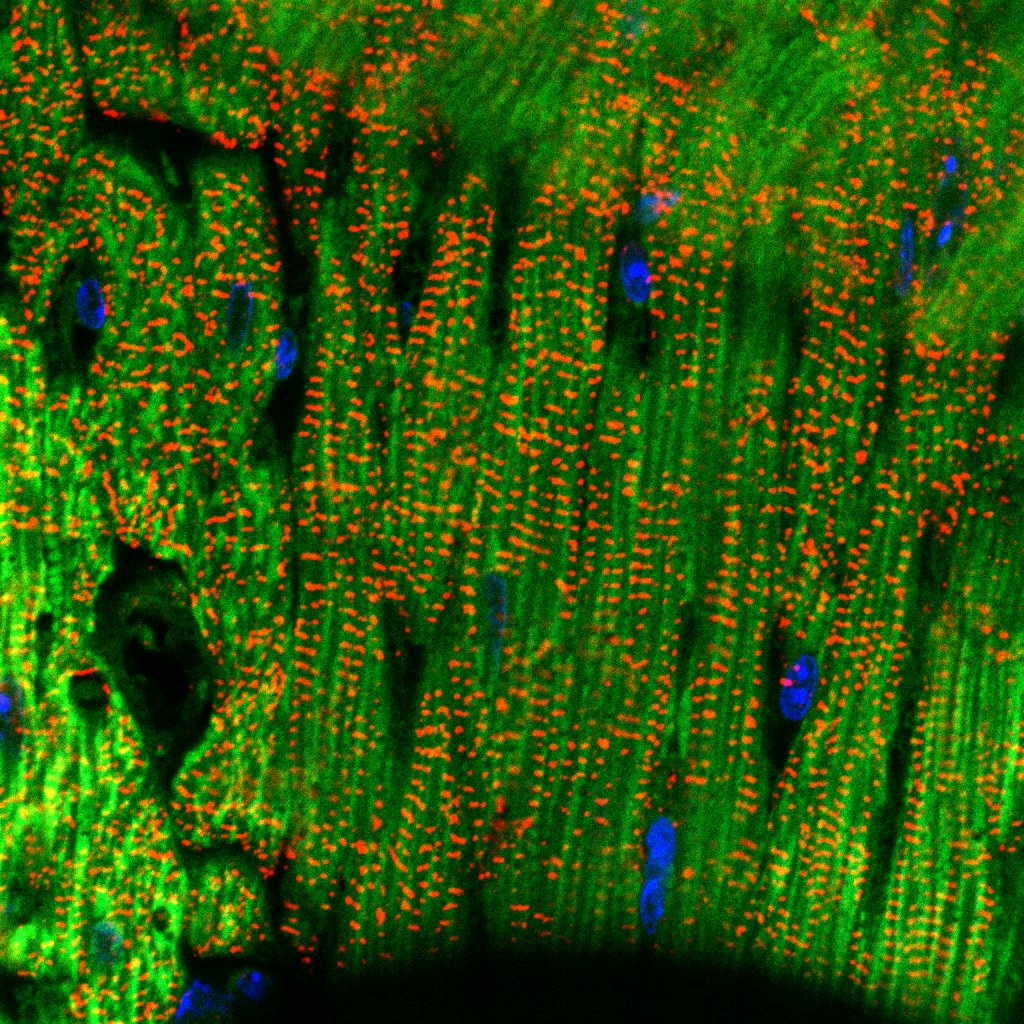

Supplement: Supplementary file 7 — Source data Fig. 4 [file 44321_2025_334_MOESM7_ESM.zip › Figure 4/4E/AAV9-Vector+TAC-Merge.jpg]

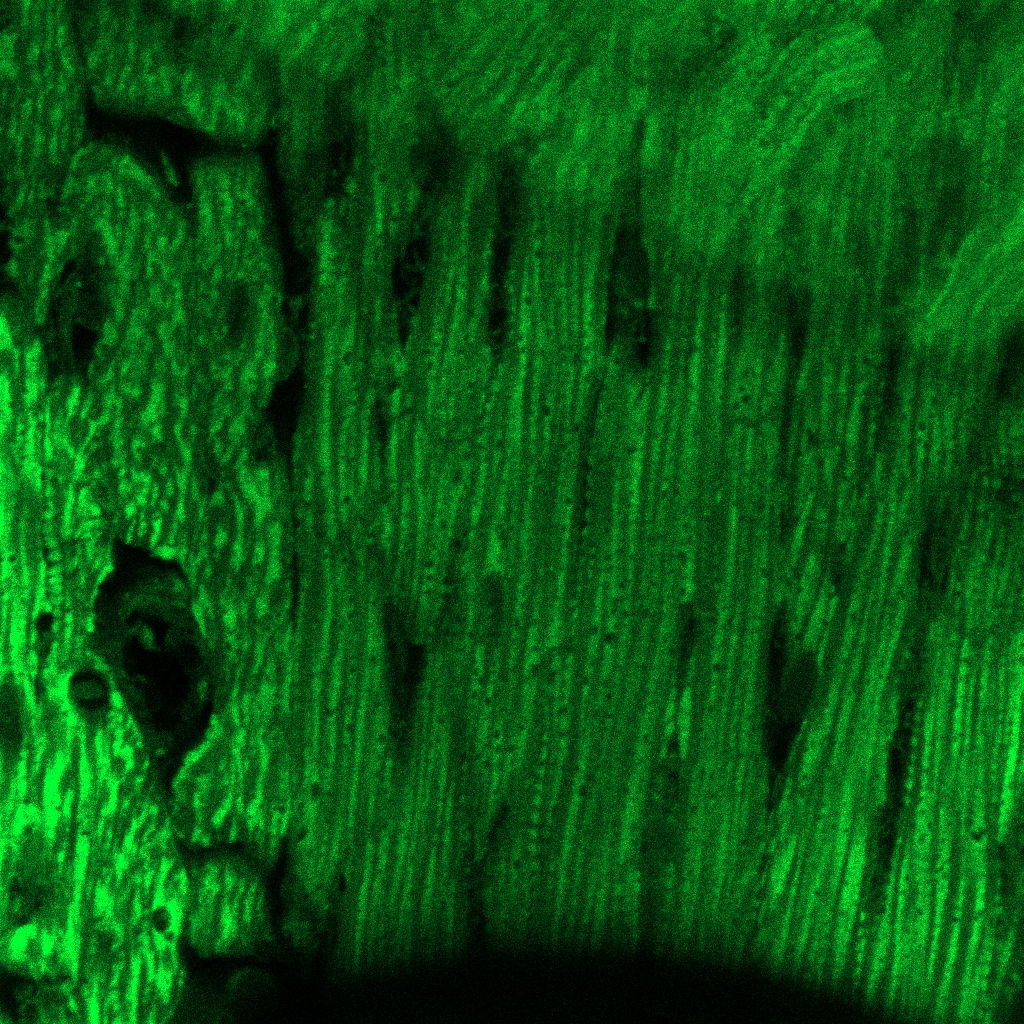

Supplement: Supplementary file 7 — Source data Fig. 4 [file 44321_2025_334_MOESM7_ESM.zip › Figure 4/4E/AAV9-Vector+TAC-α-ACTININ.jpeg]

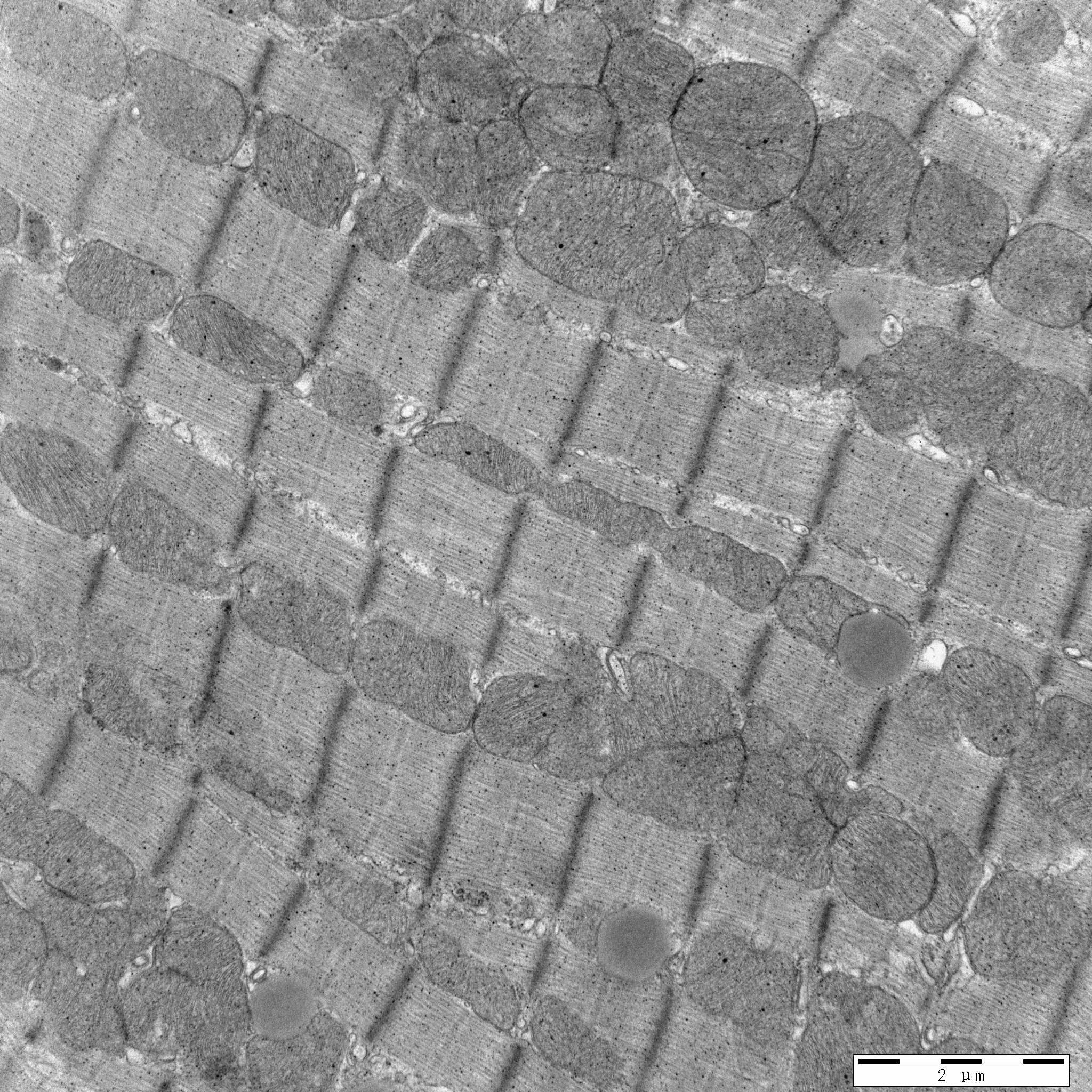

Supplement: Supplementary file 7 — Source data Fig. 4 [file 44321_2025_334_MOESM7_ESM.zip › Figure 4/4F/RBMS1-cko+Sham-1.JPG]

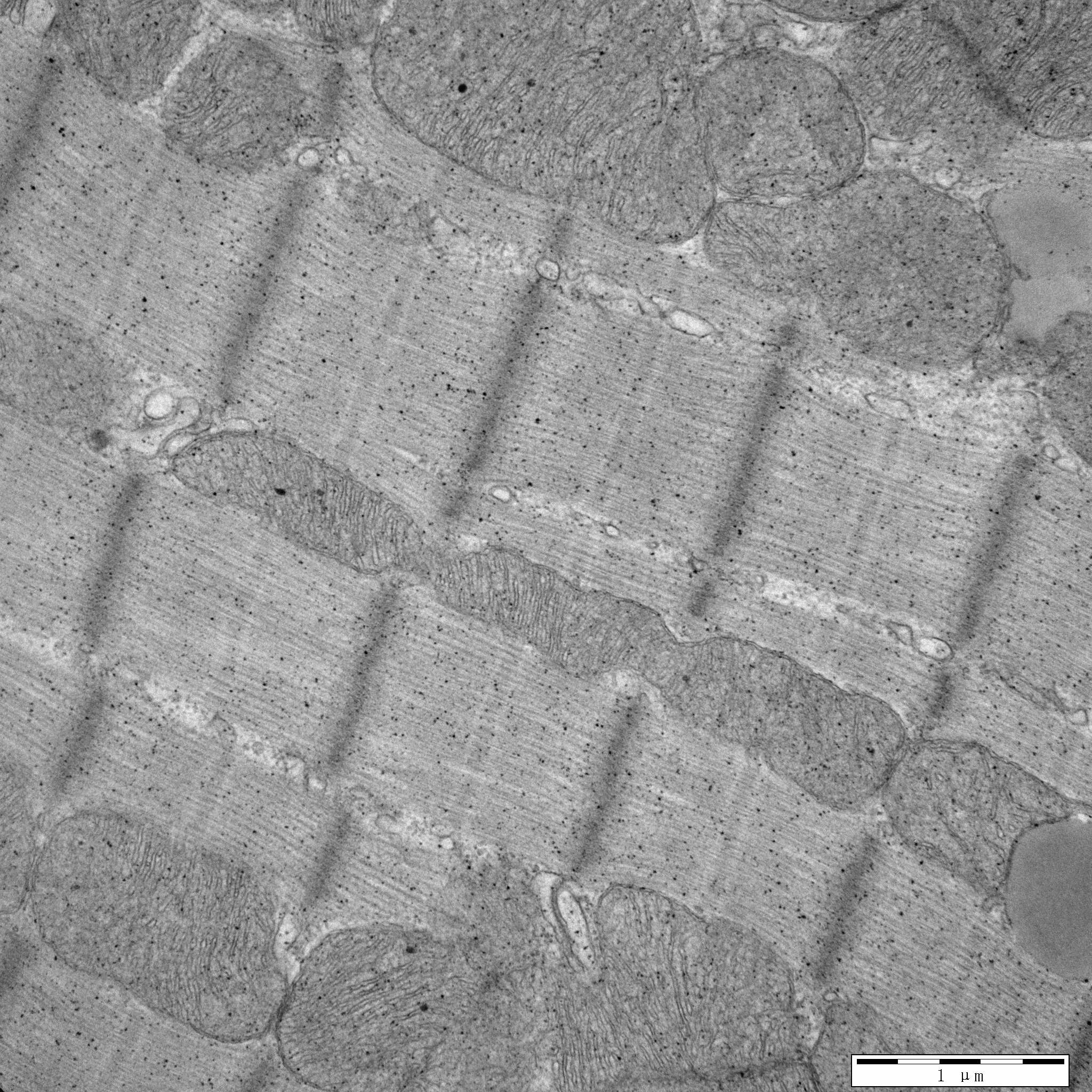

Supplement: Supplementary file 7 — Source data Fig. 4 [file 44321_2025_334_MOESM7_ESM.zip › Figure 4/4F/RBMS1-cko+Sham-2.JPG]

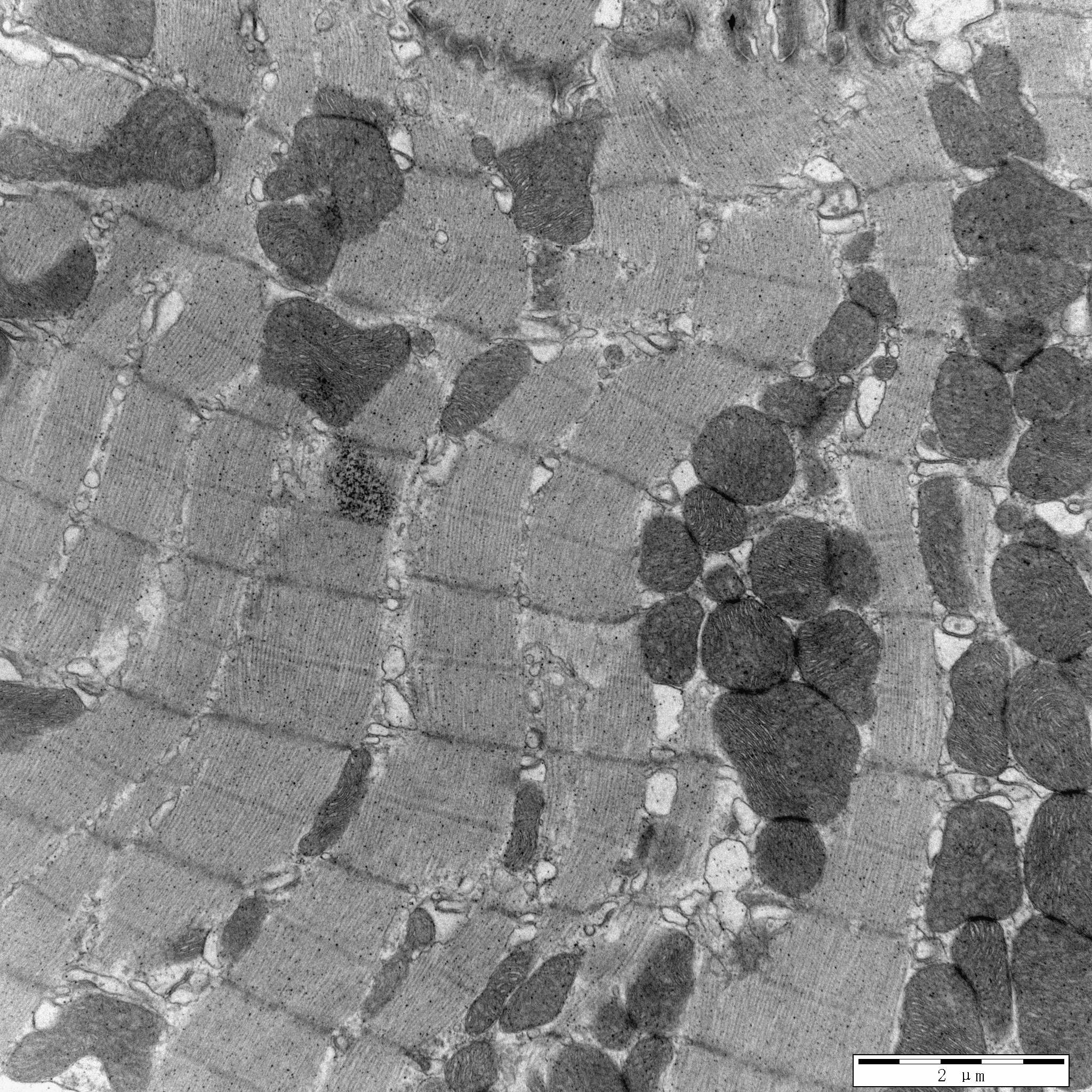

Supplement: Supplementary file 7 — Source data Fig. 4 [file 44321_2025_334_MOESM7_ESM.zip › Figure 4/4F/RBMS1-cko+TAC-1.JPG]

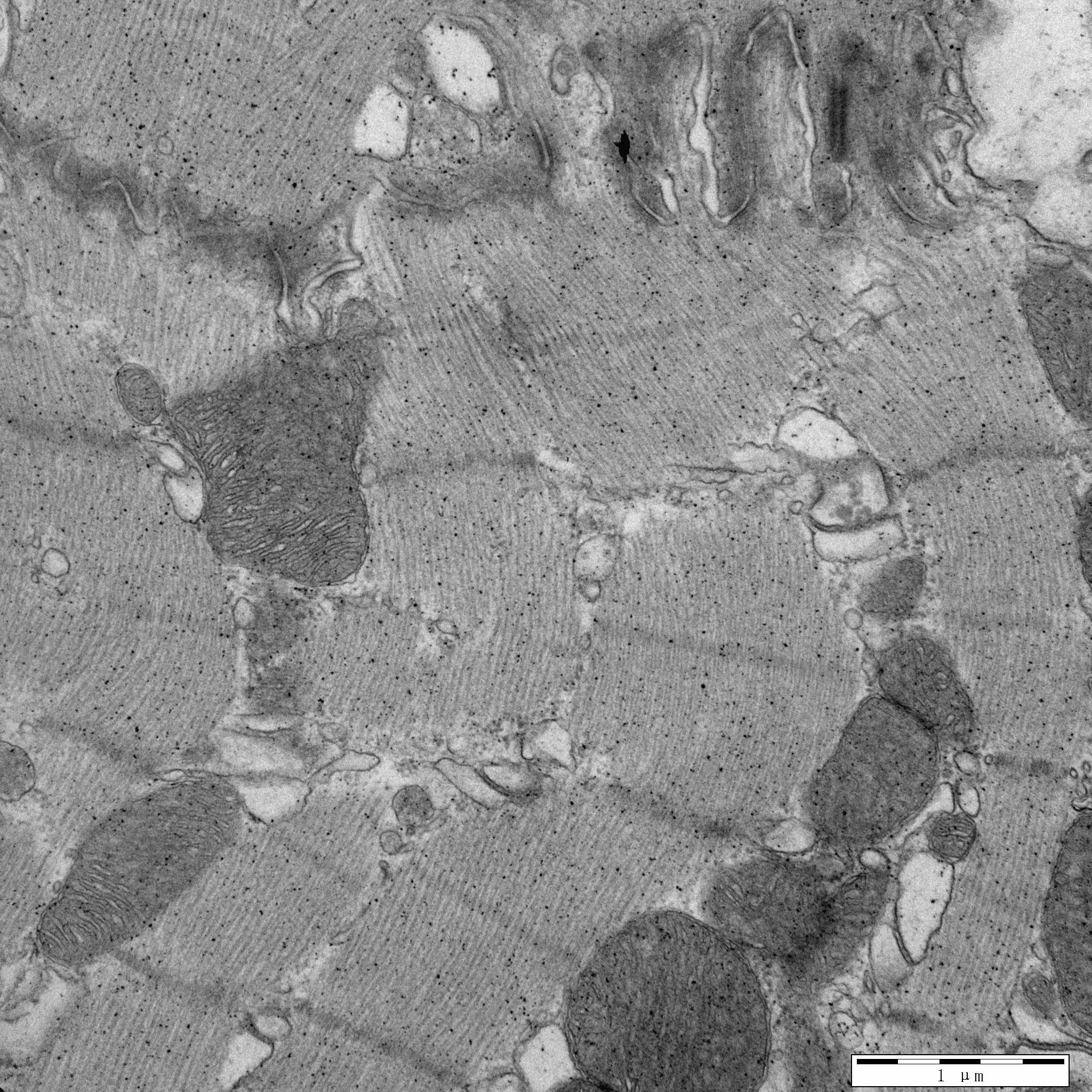

Supplement: Supplementary file 7 — Source data Fig. 4 [file 44321_2025_334_MOESM7_ESM.zip › Figure 4/4F/RBMS1-cko+TAC-2.JPG]

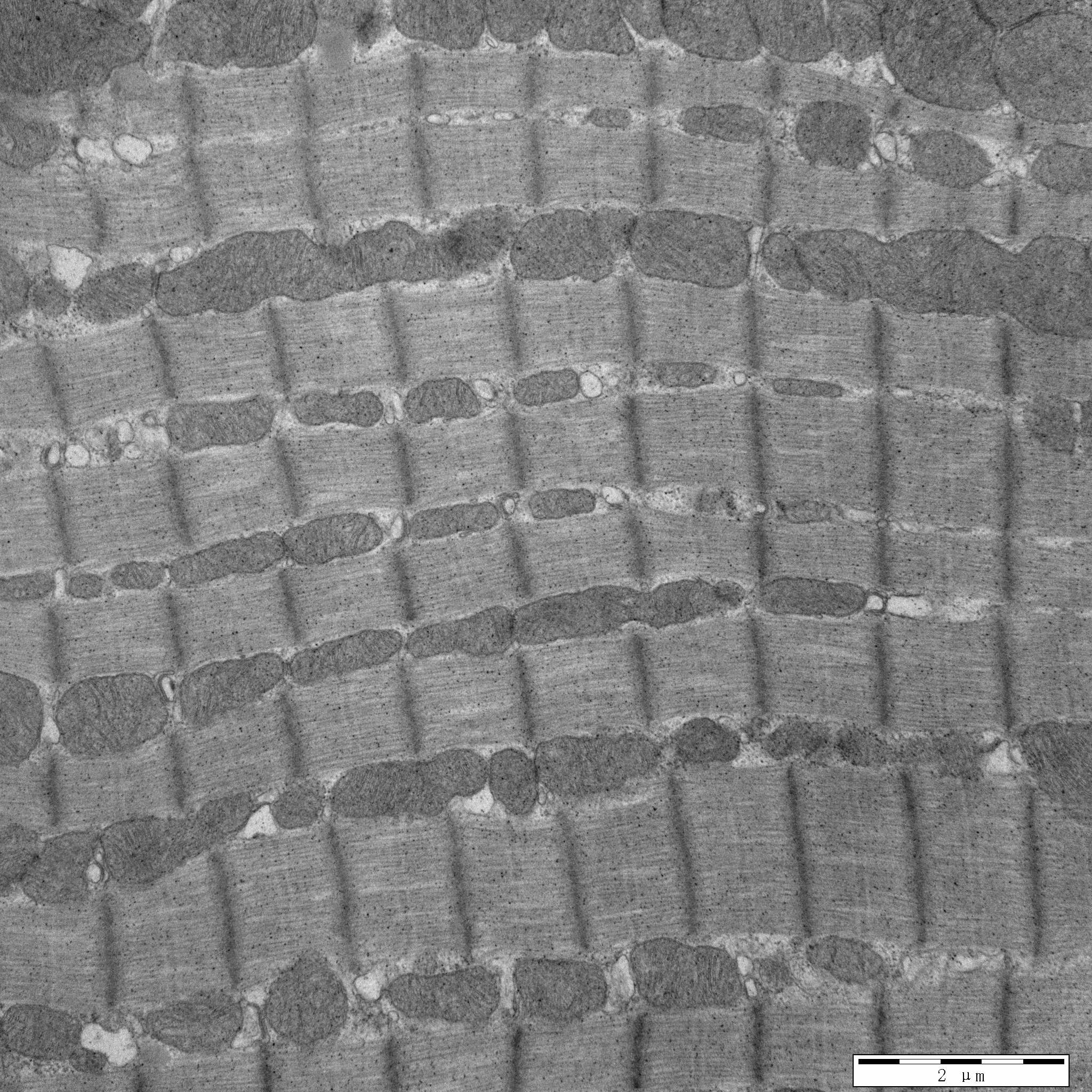

Supplement: Supplementary file 7 — Source data Fig. 4 [file 44321_2025_334_MOESM7_ESM.zip › Figure 4/4F/RBMS1-flox+Sham-1.JPG]

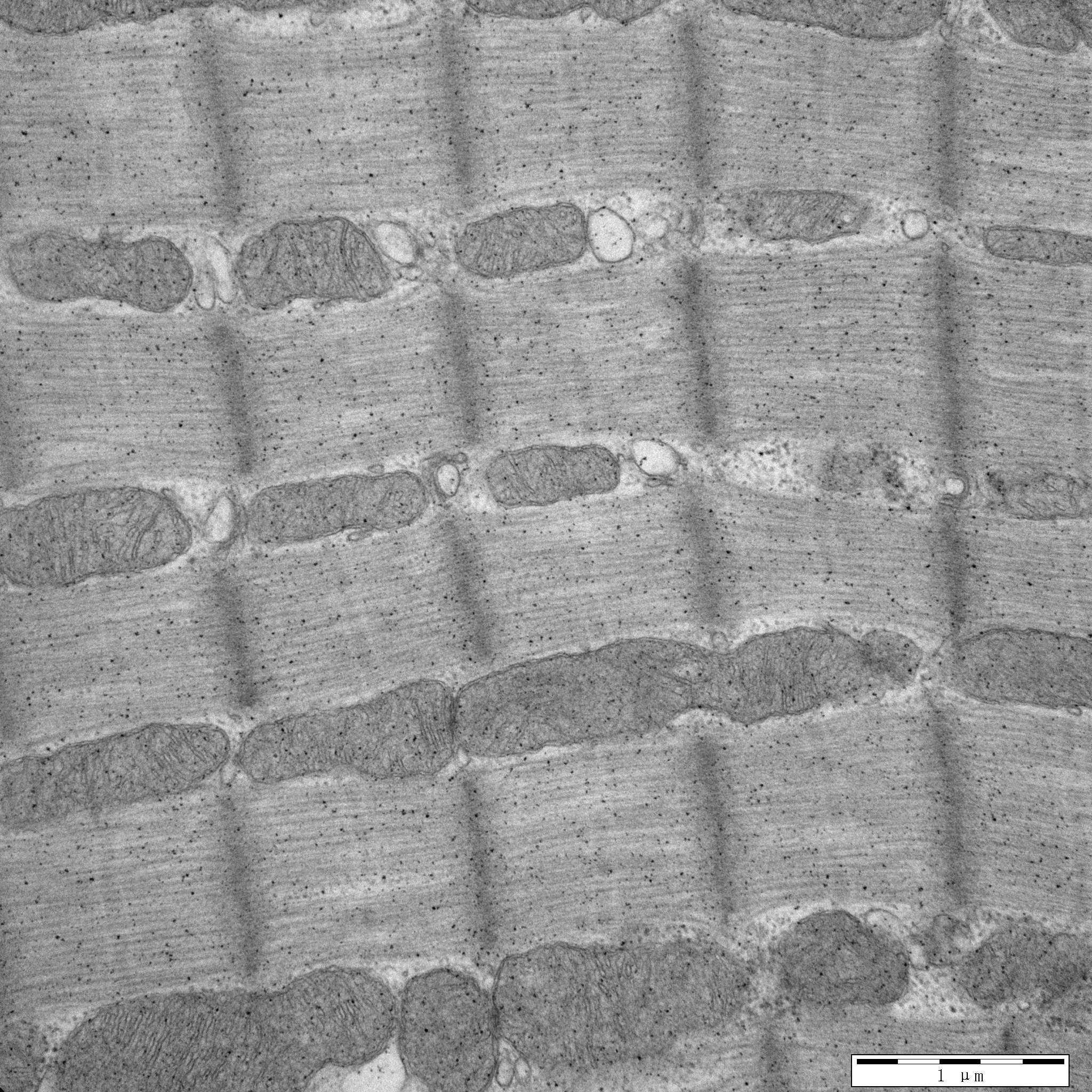

Supplement: Supplementary file 7 — Source data Fig. 4 [file 44321_2025_334_MOESM7_ESM.zip › Figure 4/4F/RBMS1-flox+Sham-2.JPG]

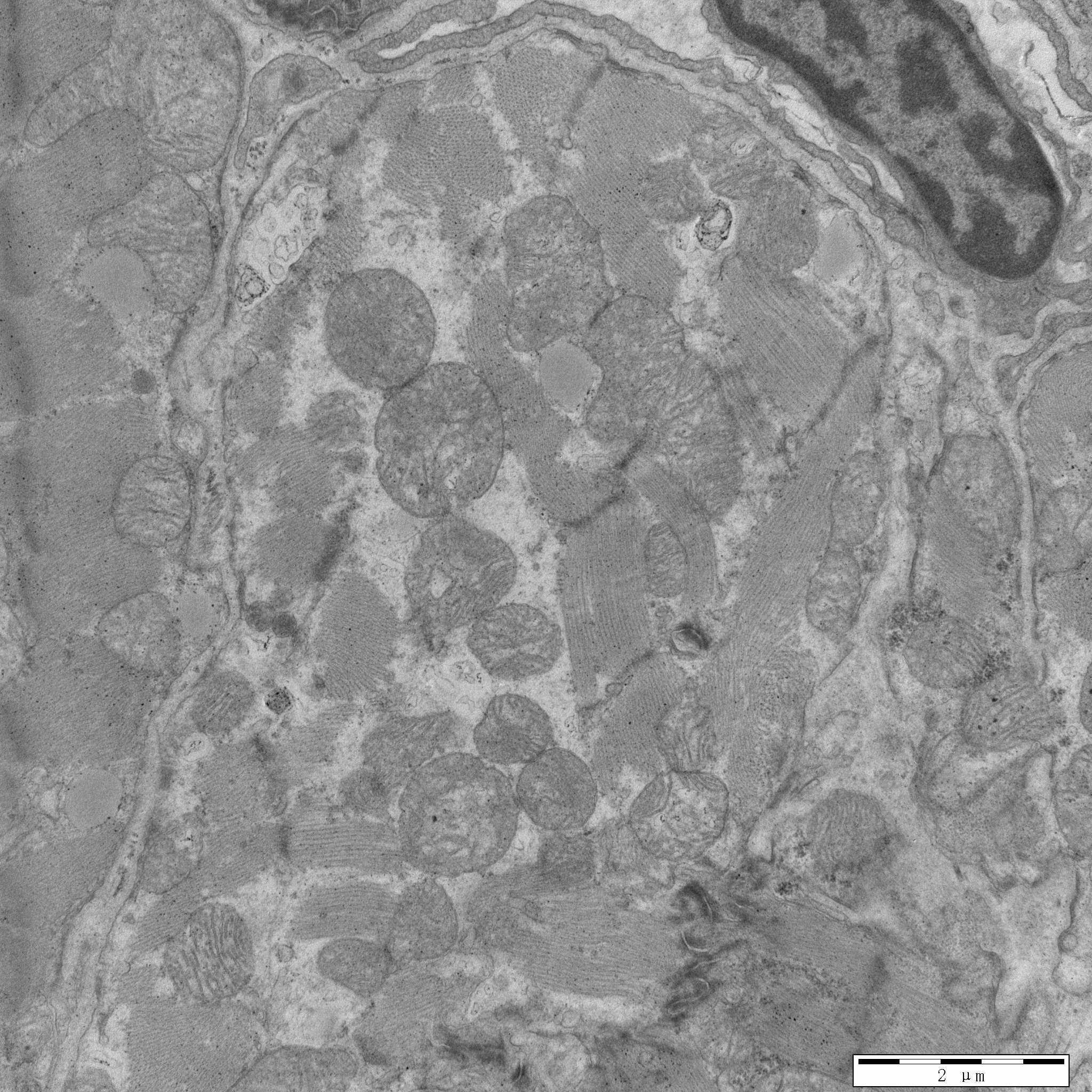

Supplement: Supplementary file 7 — Source data Fig. 4 [file 44321_2025_334_MOESM7_ESM.zip › Figure 4/4F/RBMS1-flox+TAC-1.JPG]

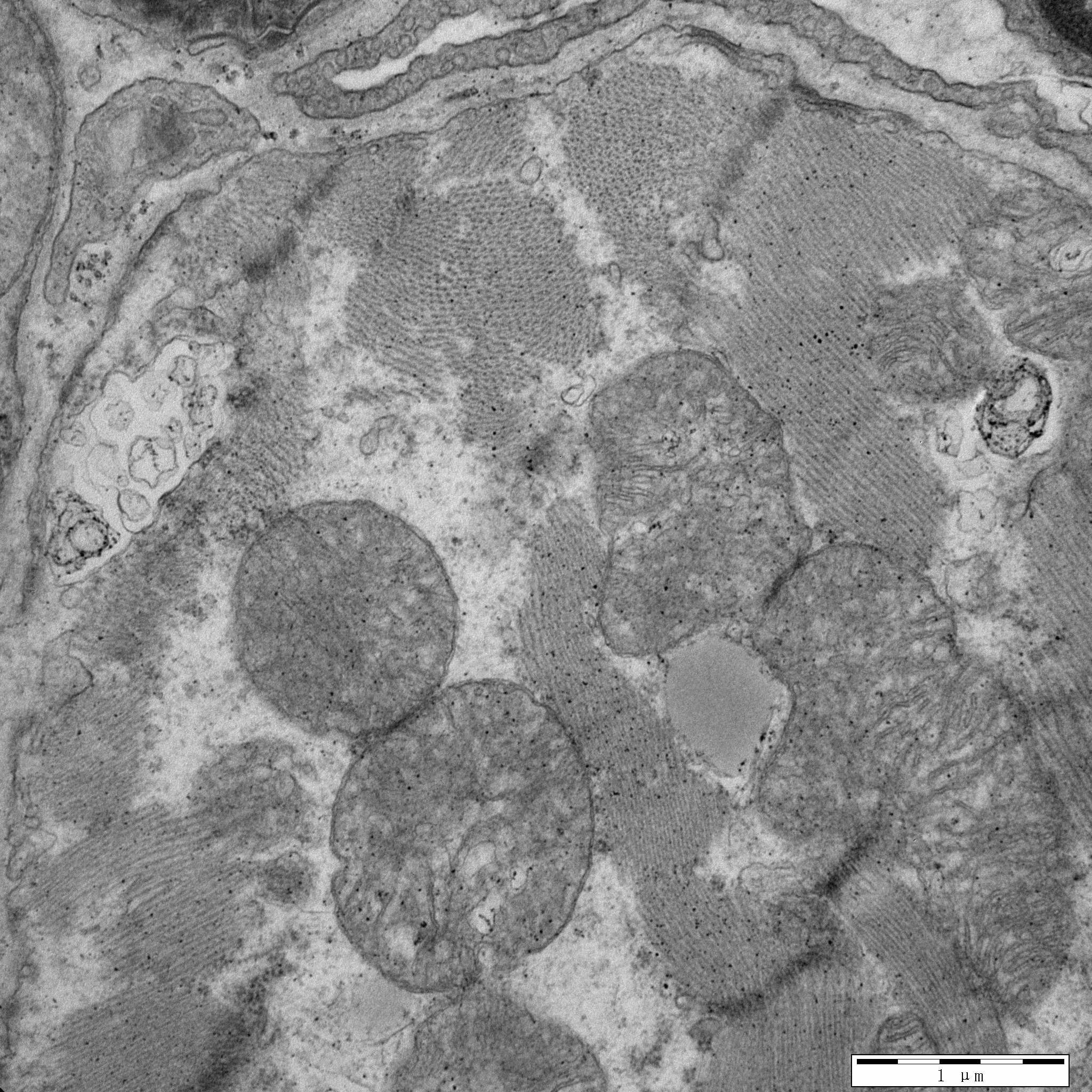

Supplement: Supplementary file 7 — Source data Fig. 4 [file 44321_2025_334_MOESM7_ESM.zip › Figure 4/4F/RBMS1-flox+TAC-2.JPG]

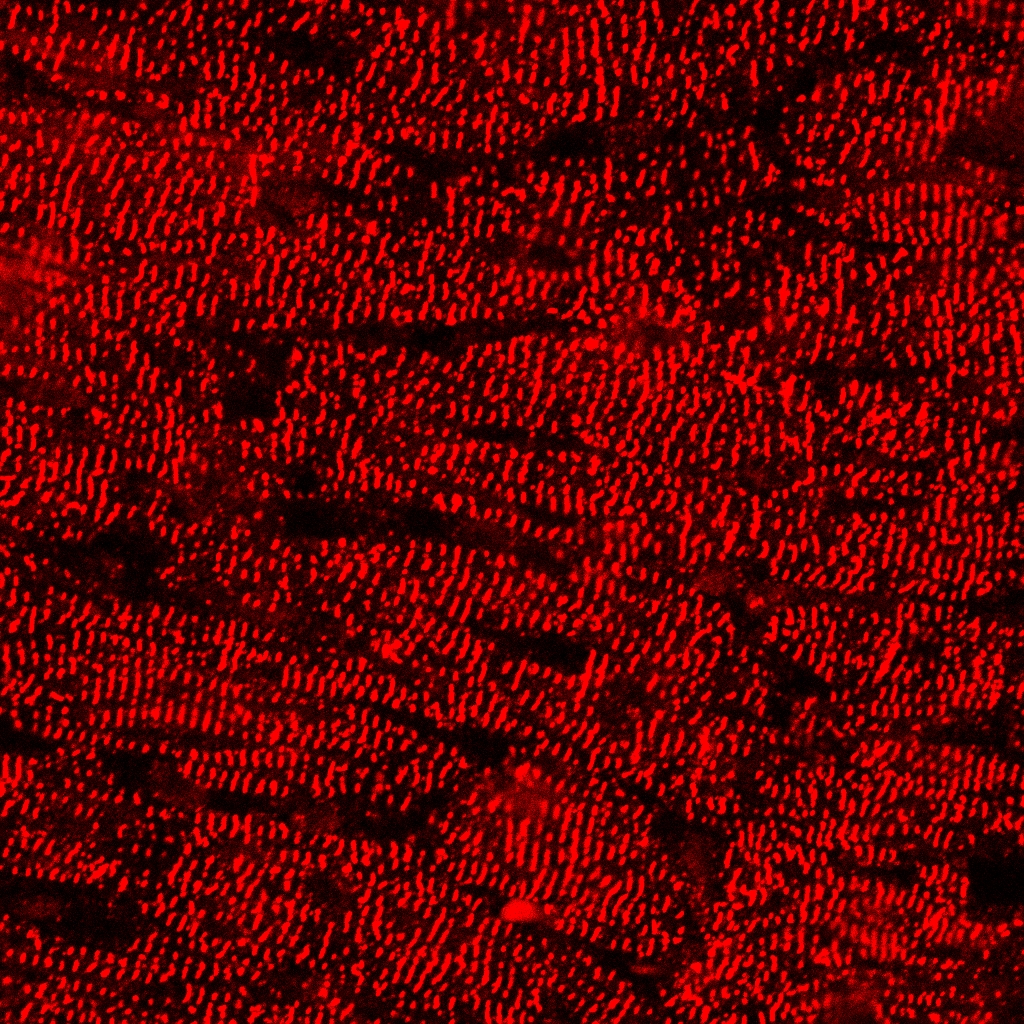

Supplement: Supplementary file 7 — Source data Fig. 4 [file 44321_2025_334_MOESM7_ESM.zip › Figure 4/4G/RBMS1-cko+Sham-ACTN2.jpeg]

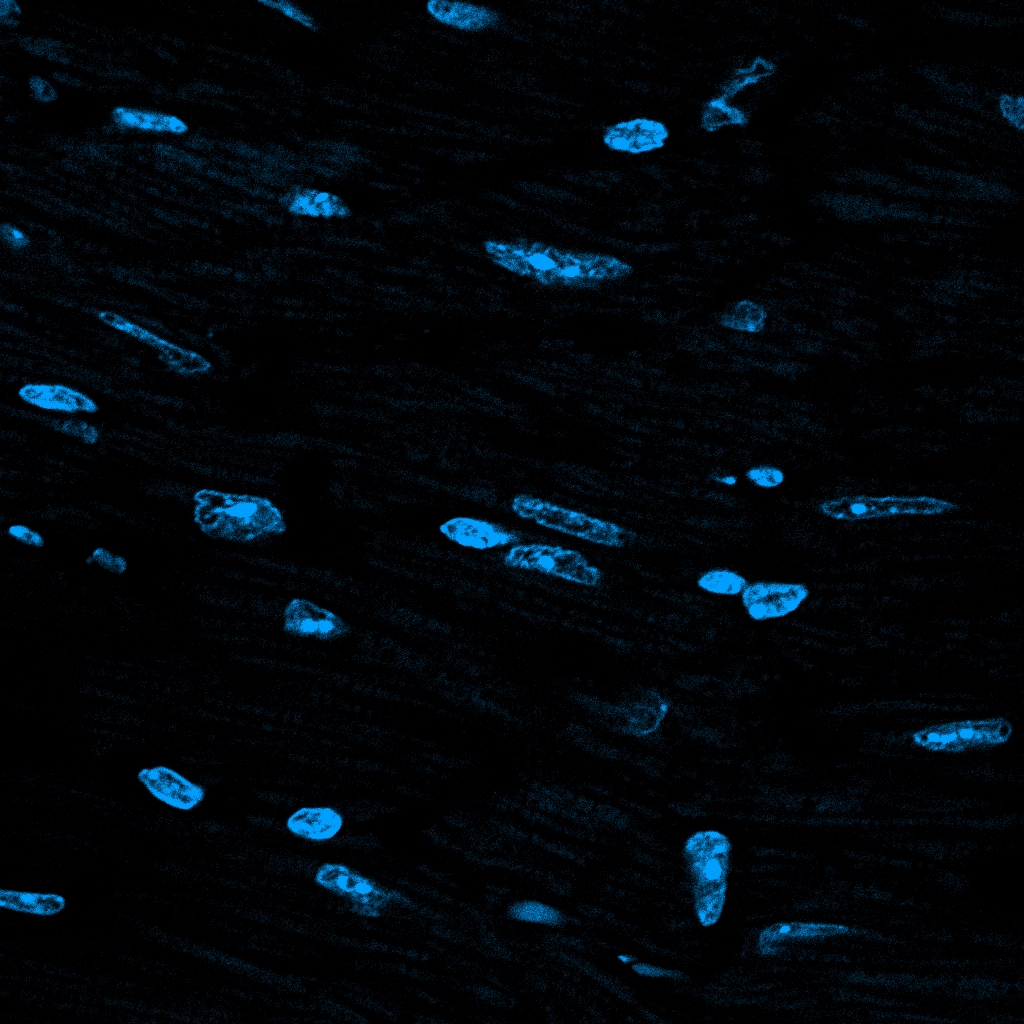

Supplement: Supplementary file 7 — Source data Fig. 4 [file 44321_2025_334_MOESM7_ESM.zip › Figure 4/4G/RBMS1-cko+Sham-DAPI.jpeg]

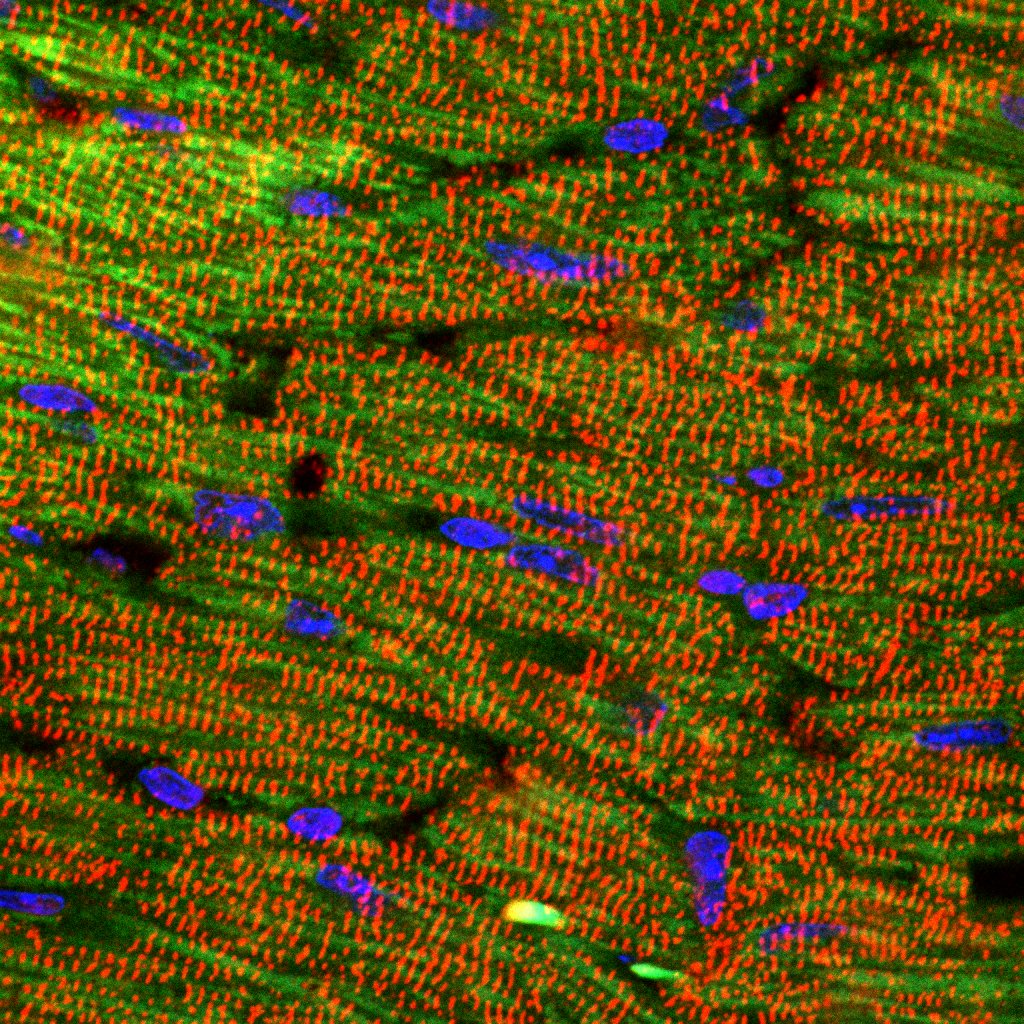

Supplement: Supplementary file 7 — Source data Fig. 4 [file 44321_2025_334_MOESM7_ESM.zip › Figure 4/4G/RBMS1-cko+Sham-Merge.jpg]

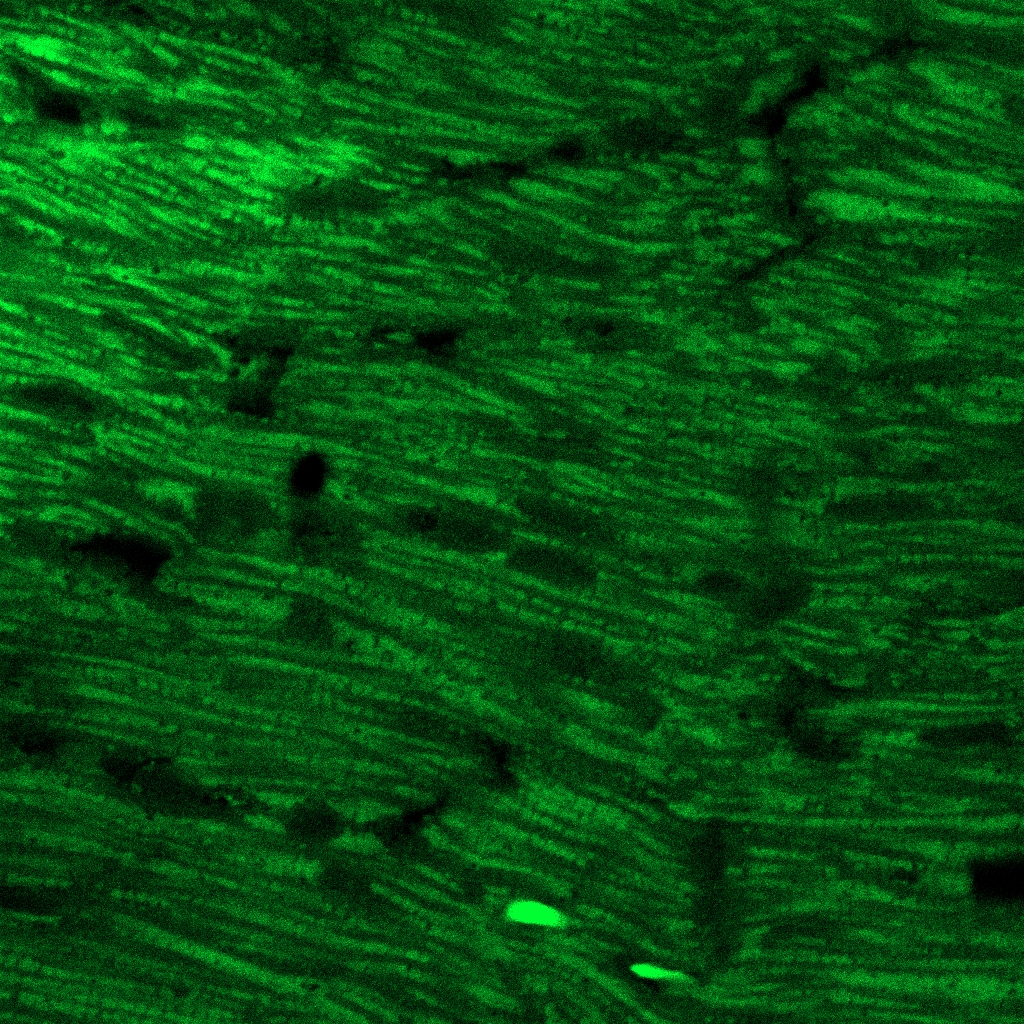

Supplement: Supplementary file 7 — Source data Fig. 4 [file 44321_2025_334_MOESM7_ESM.zip › Figure 4/4G/RBMS1-cko+Sham-α-ACTININ.jpeg]

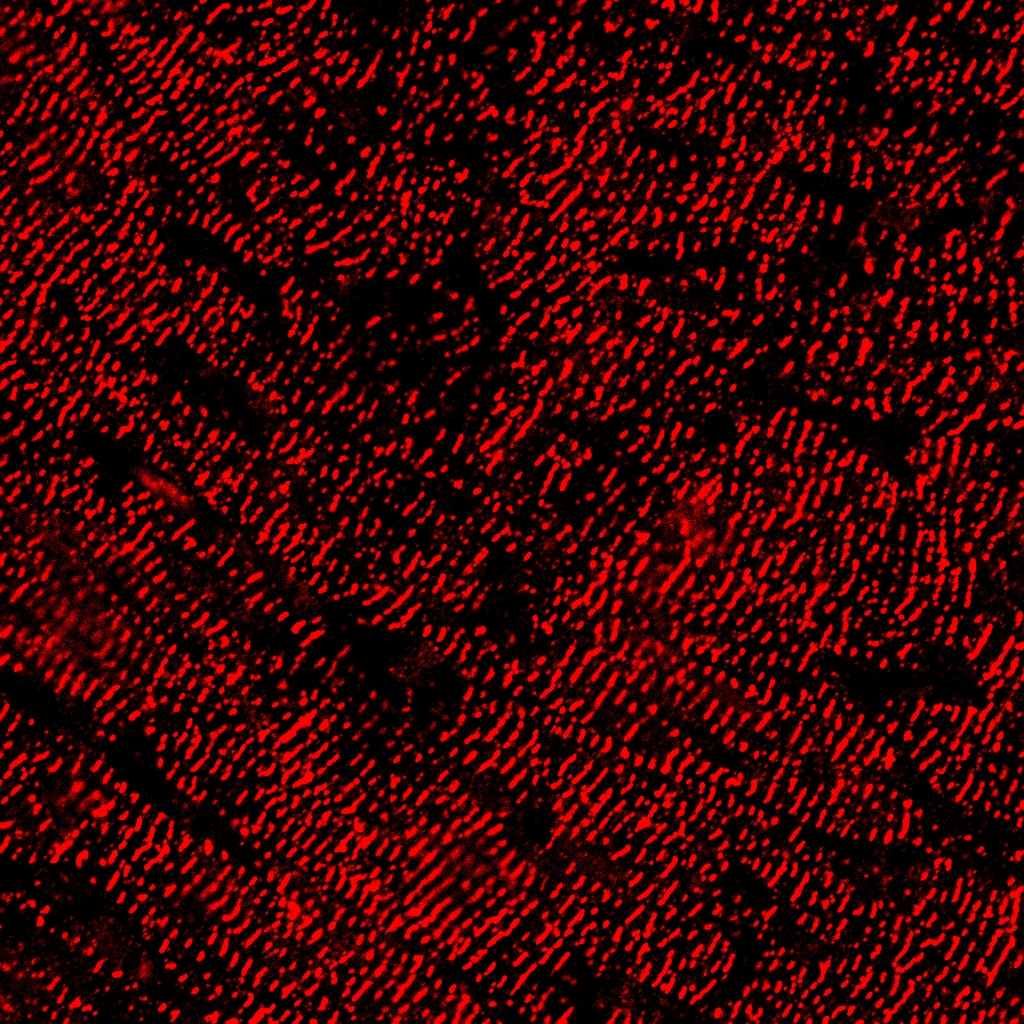

Supplement: Supplementary file 7 — Source data Fig. 4 [file 44321_2025_334_MOESM7_ESM.zip › Figure 4/4G/RBMS1-cko+TAC-ACTN2.jpeg]

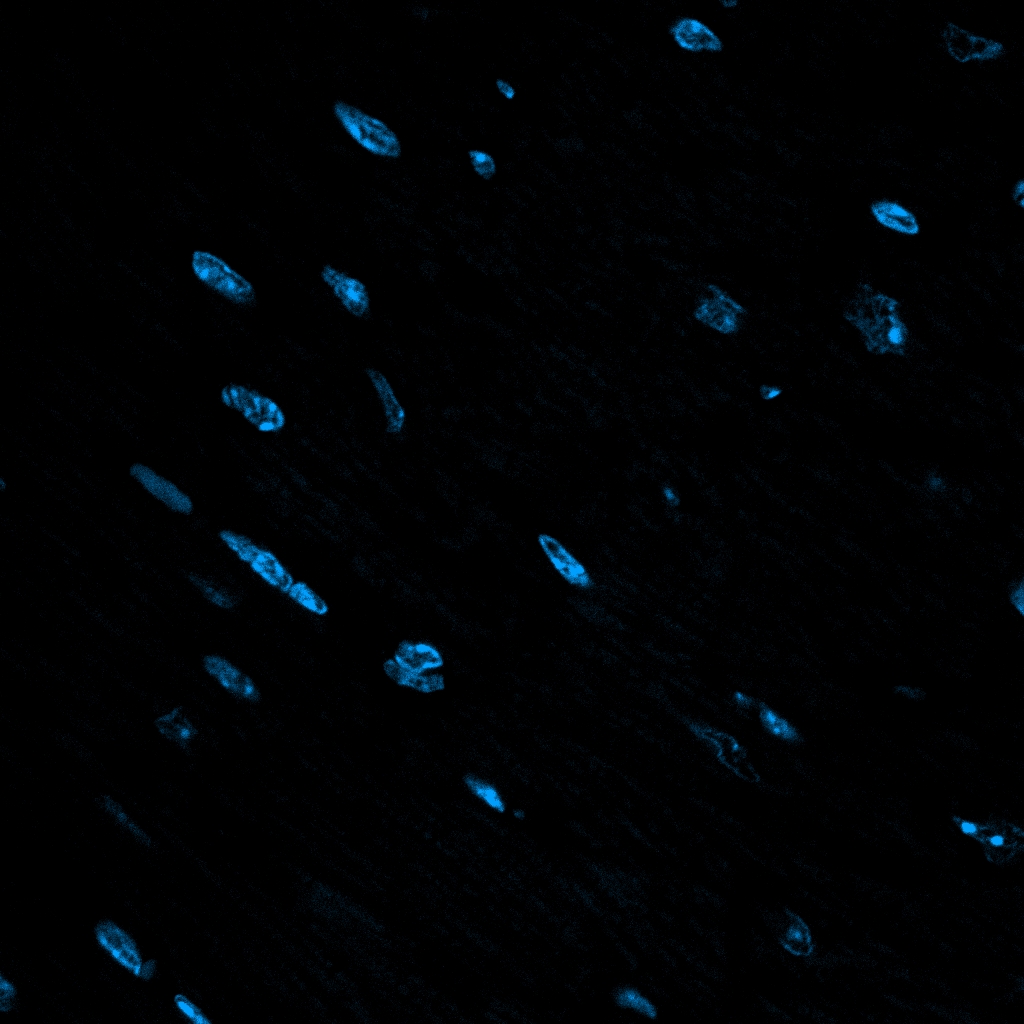

Supplement: Supplementary file 7 — Source data Fig. 4 [file 44321_2025_334_MOESM7_ESM.zip › Figure 4/4G/RBMS1-cko+TAC-DAPI.jpeg]

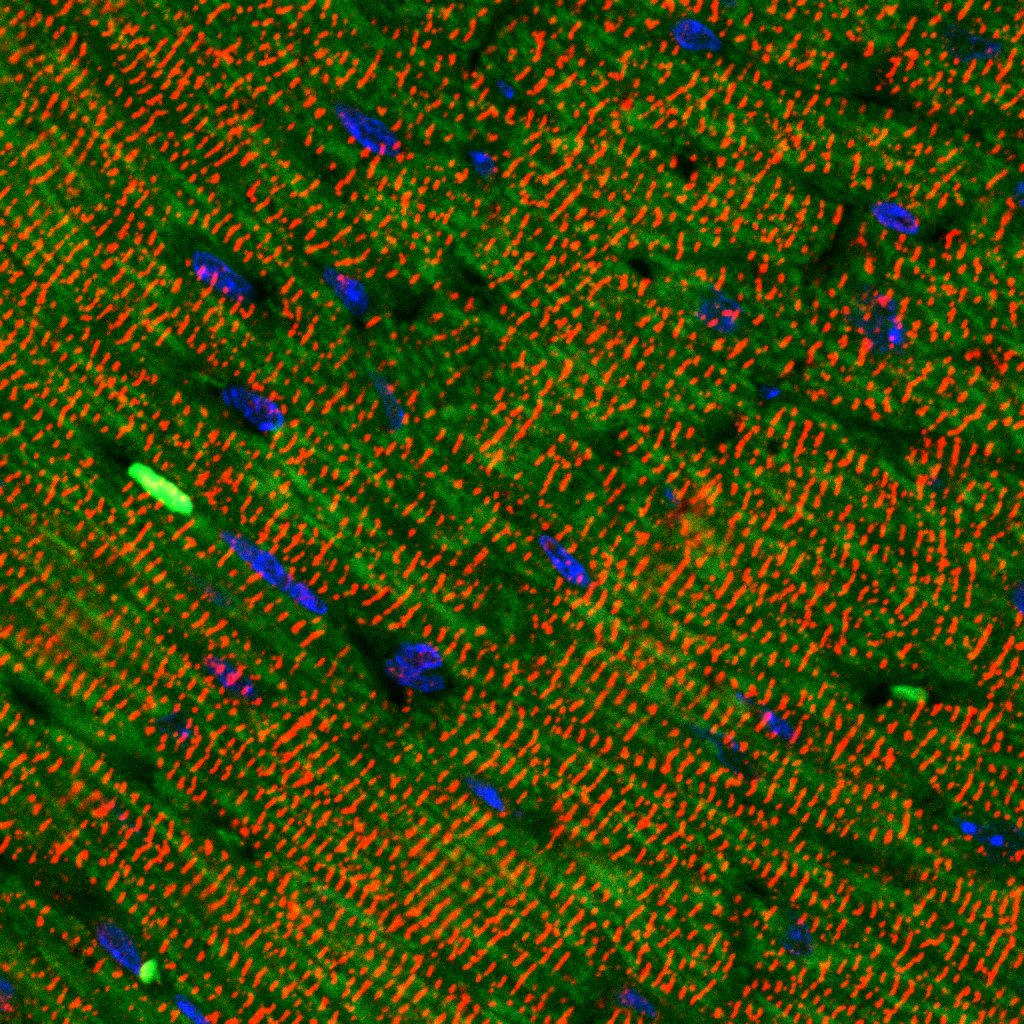

Supplement: Supplementary file 7 — Source data Fig. 4 [file 44321_2025_334_MOESM7_ESM.zip › Figure 4/4G/RBMS1-cko+TAC-Merge.jpg]

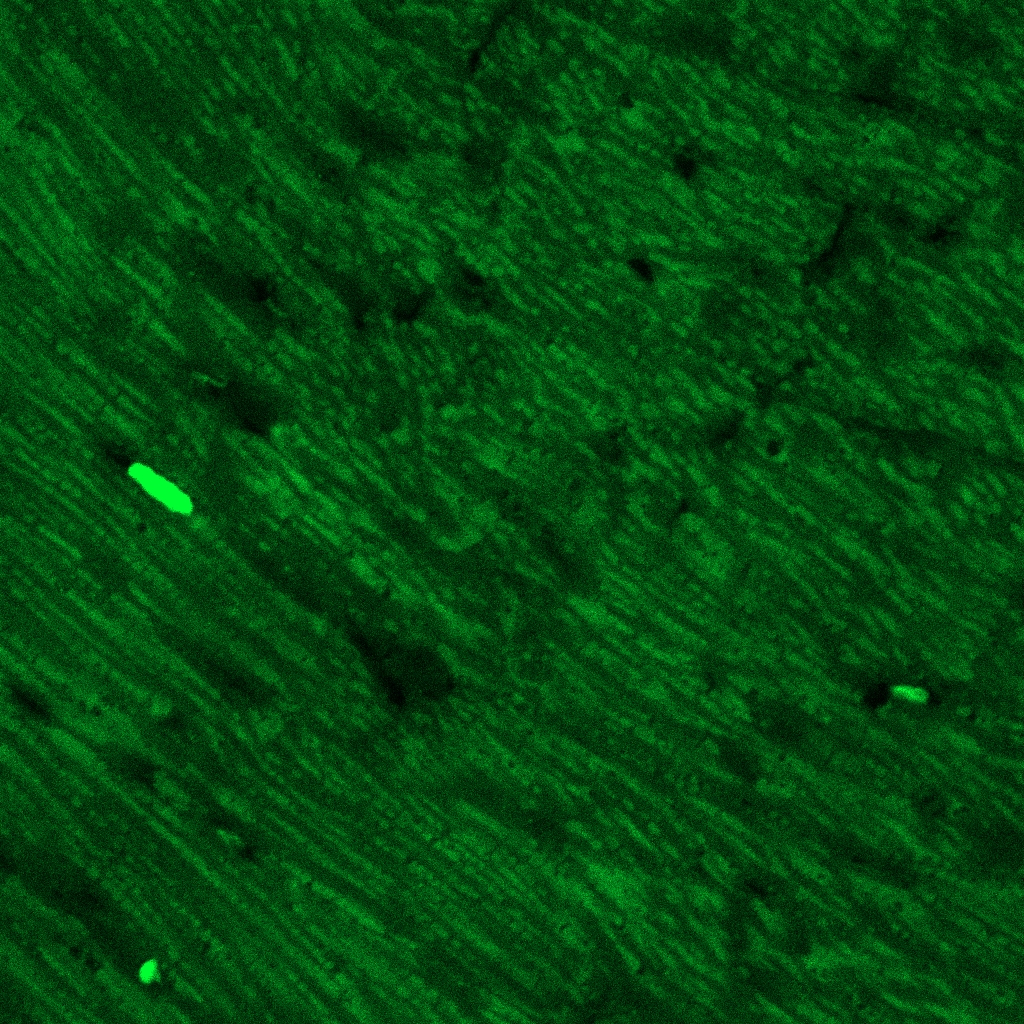

Supplement: Supplementary file 7 — Source data Fig. 4 [file 44321_2025_334_MOESM7_ESM.zip › Figure 4/4G/RBMS1-cko+TAC-α-ACTININ.jpeg]

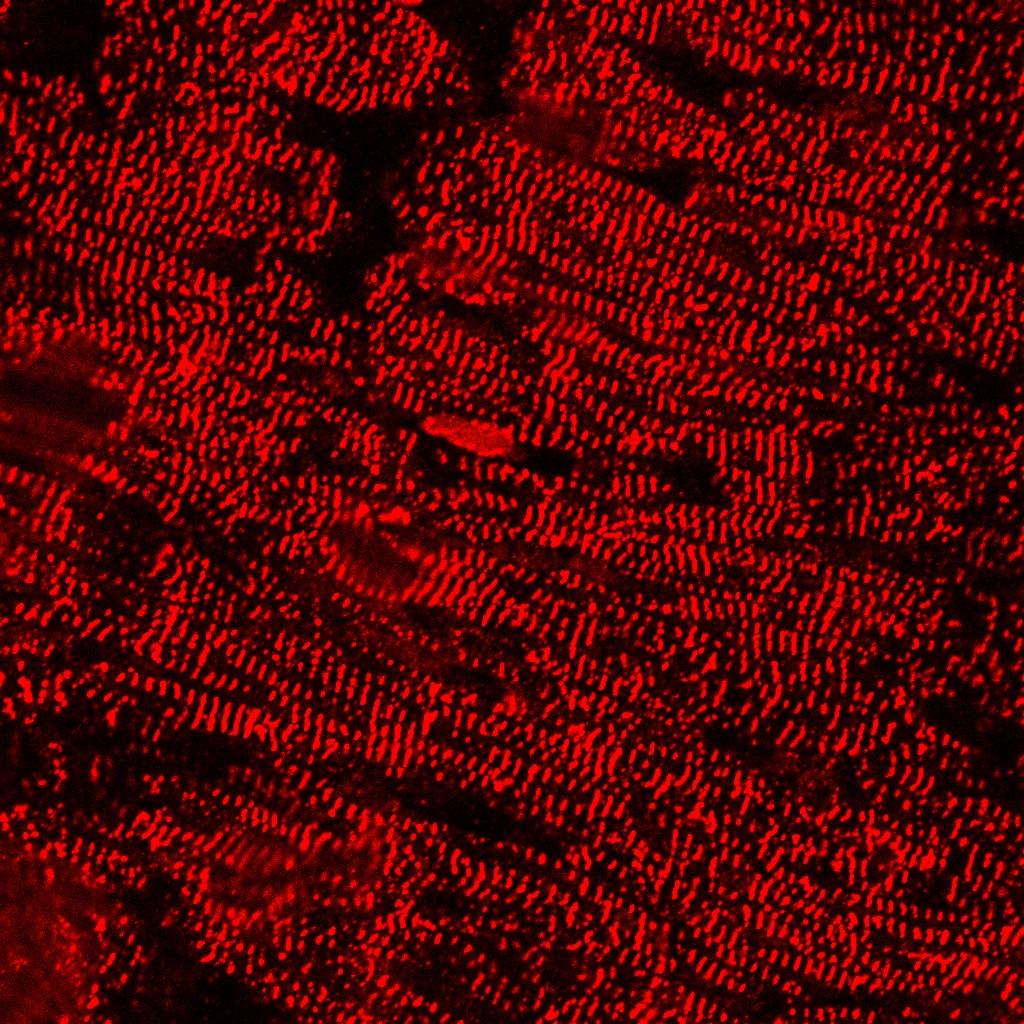

Supplement: Supplementary file 7 — Source data Fig. 4 [file 44321_2025_334_MOESM7_ESM.zip › Figure 4/4G/RBMS1-flox+Sham-ACTN2.jpeg]

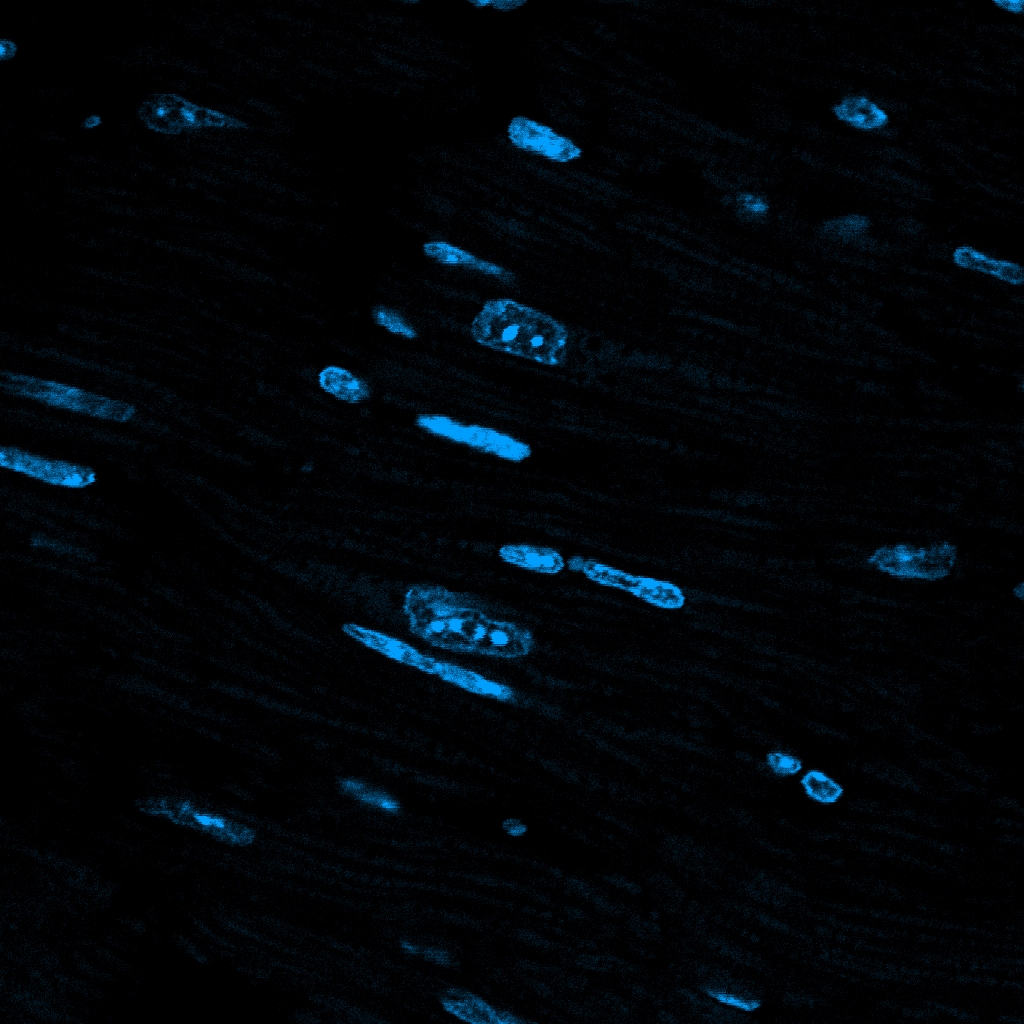

Supplement: Supplementary file 7 — Source data Fig. 4 [file 44321_2025_334_MOESM7_ESM.zip › Figure 4/4G/RBMS1-flox+Sham-DAPI.jpeg]

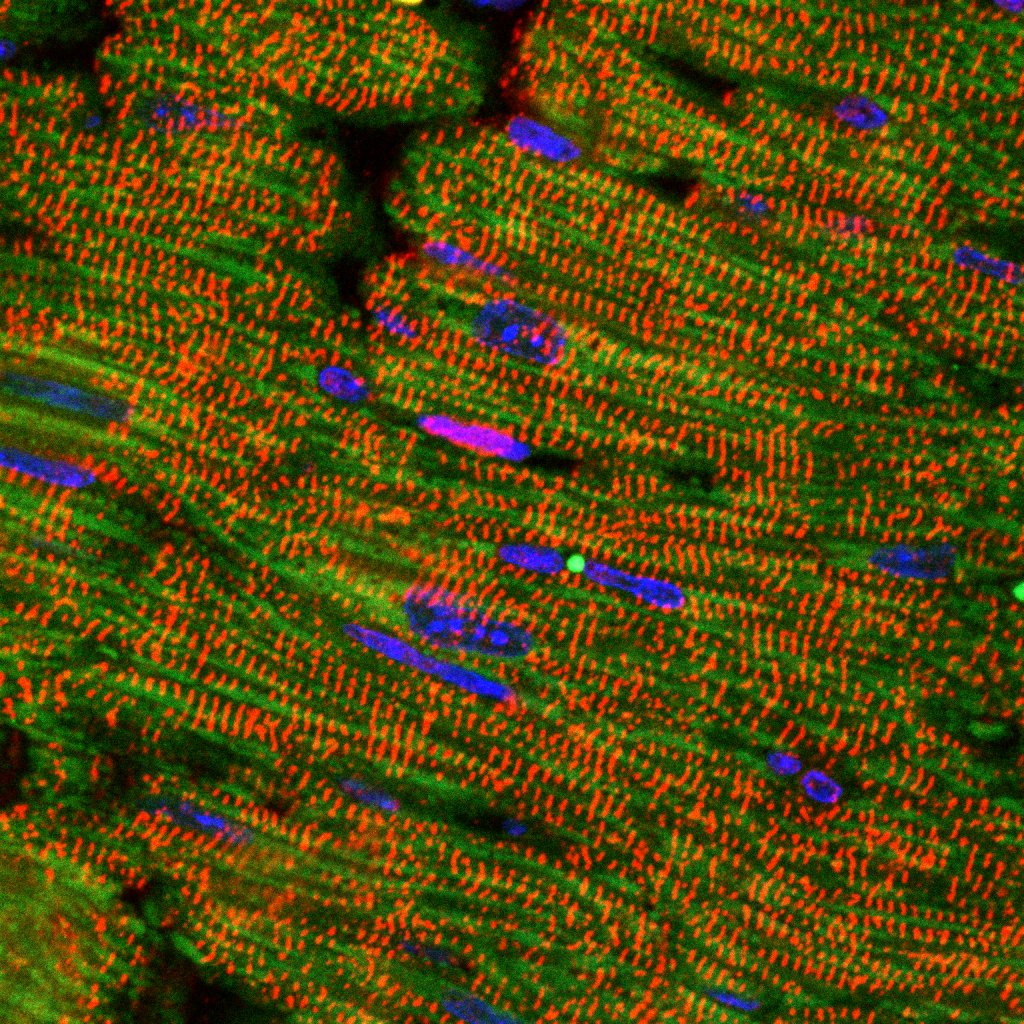

Supplement: Supplementary file 7 — Source data Fig. 4 [file 44321_2025_334_MOESM7_ESM.zip › Figure 4/4G/RBMS1-flox+Sham-Merge.jpg]

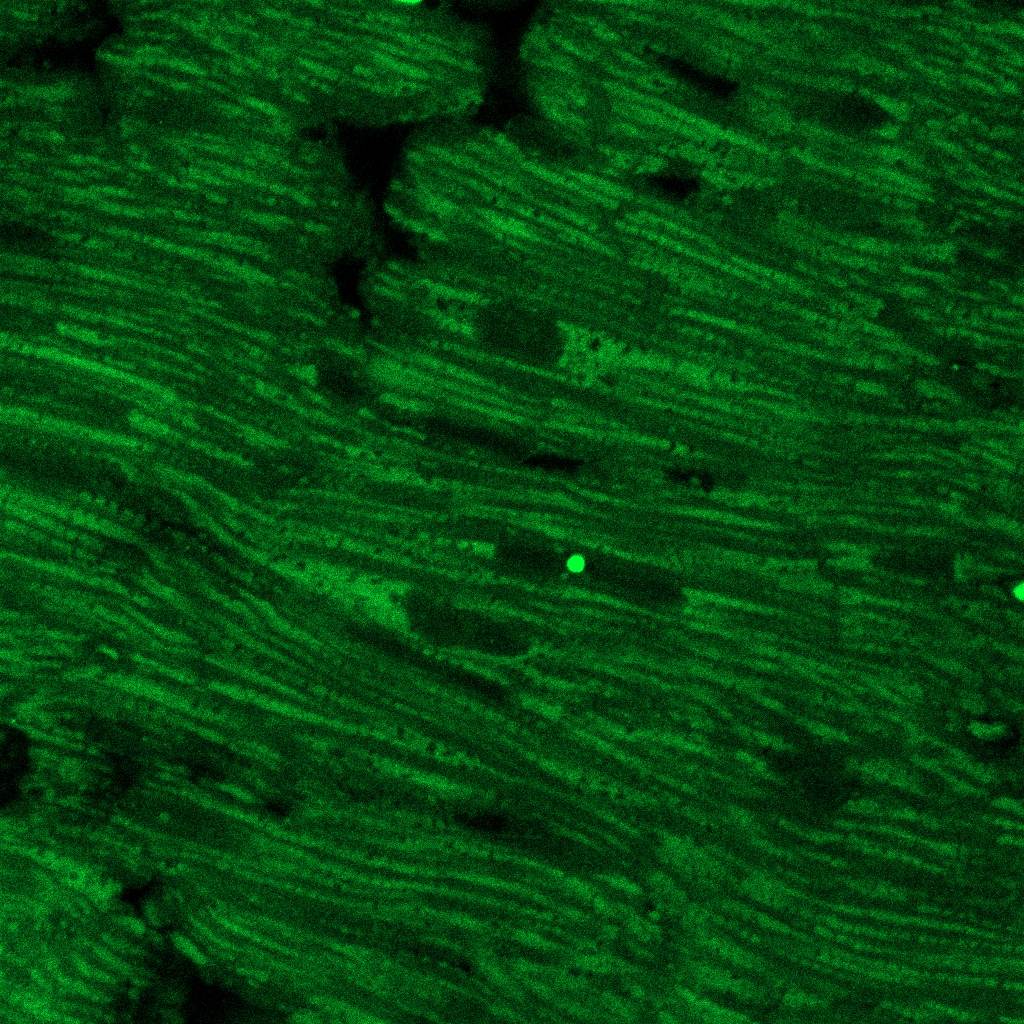

Supplement: Supplementary file 7 — Source data Fig. 4 [file 44321_2025_334_MOESM7_ESM.zip › Figure 4/4G/RBMS1-flox+Sham-α-ACTININ.jpeg]

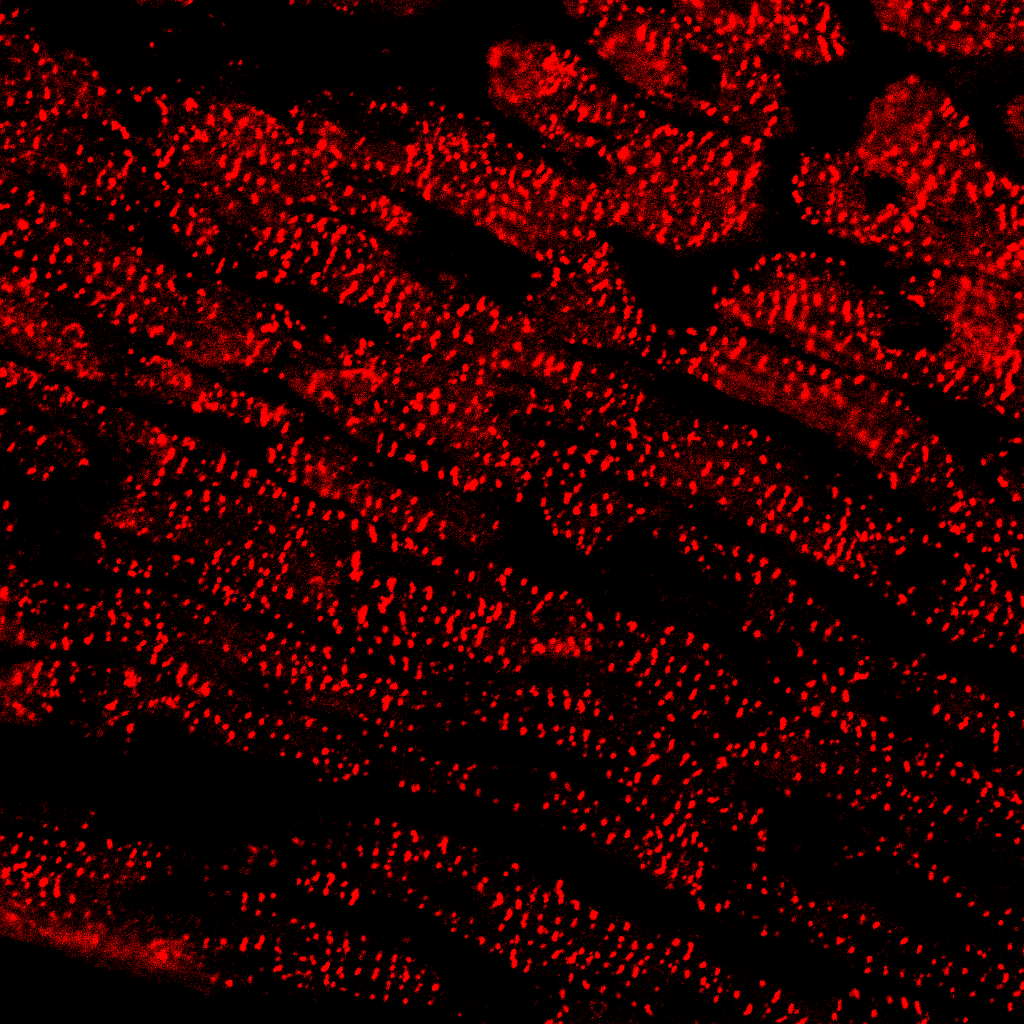

Supplement: Supplementary file 7 — Source data Fig. 4 [file 44321_2025_334_MOESM7_ESM.zip › Figure 4/4G/RBMS1-flox+TAC-ACTN2.tiff]

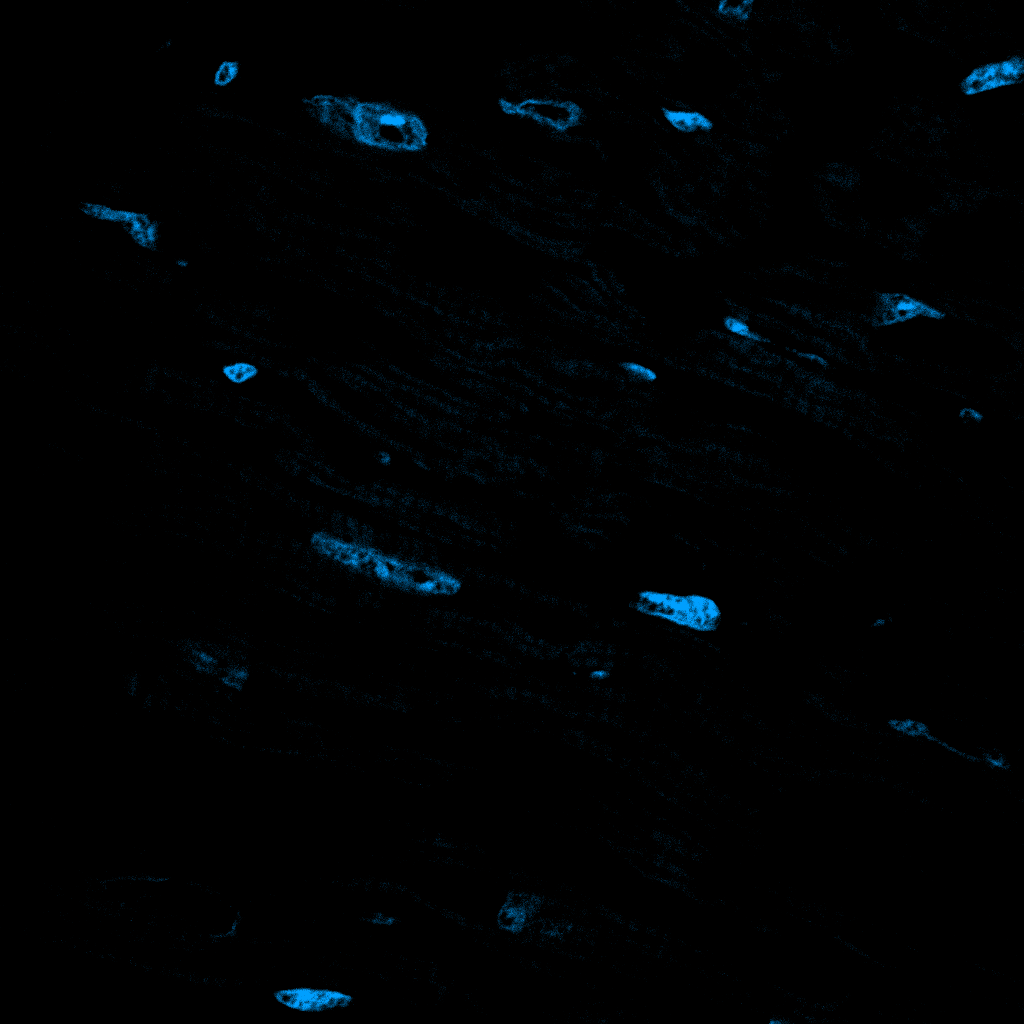

Supplement: Supplementary file 7 — Source data Fig. 4 [file 44321_2025_334_MOESM7_ESM.zip › Figure 4/4G/RBMS1-flox+TAC-DAPI.tiff]

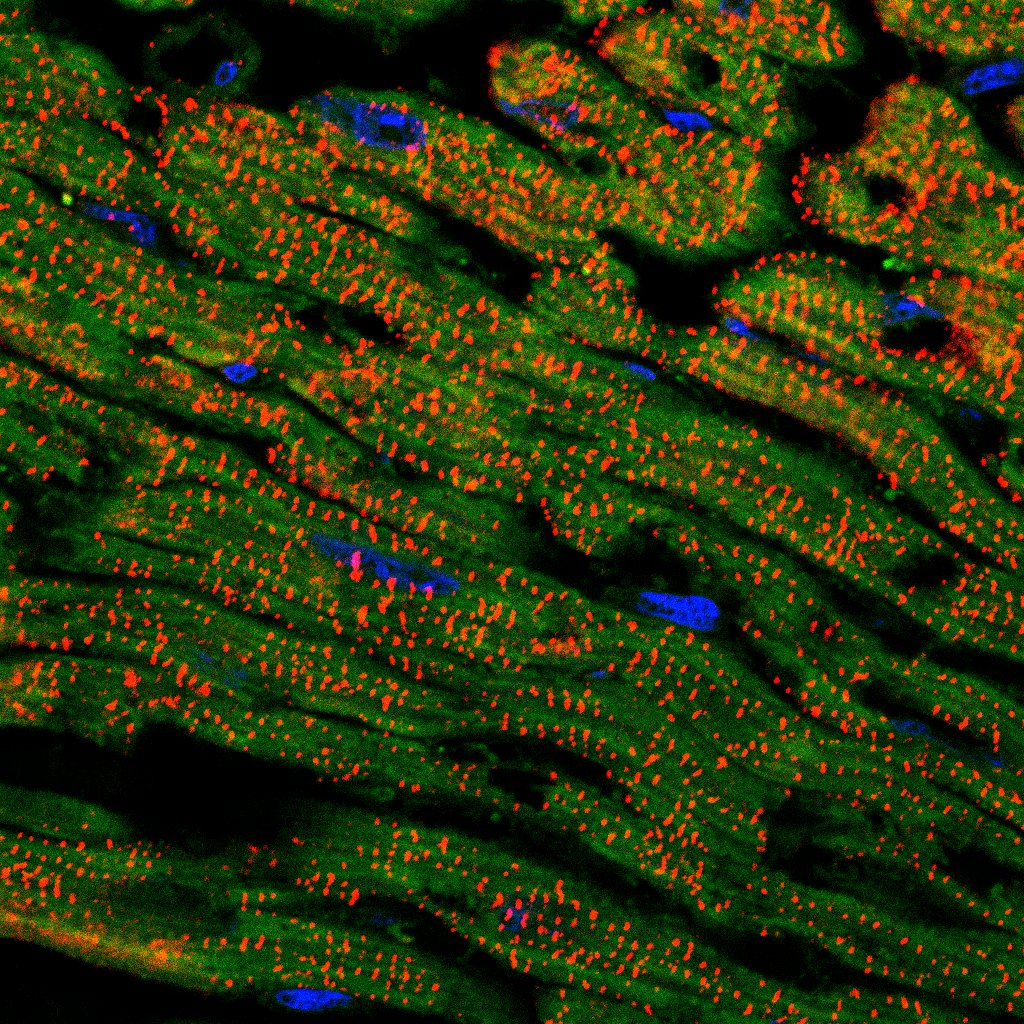

Supplement: Supplementary file 7 — Source data Fig. 4 [file 44321_2025_334_MOESM7_ESM.zip › Figure 4/4G/RBMS1-flox+TAC-Merge.jpg]

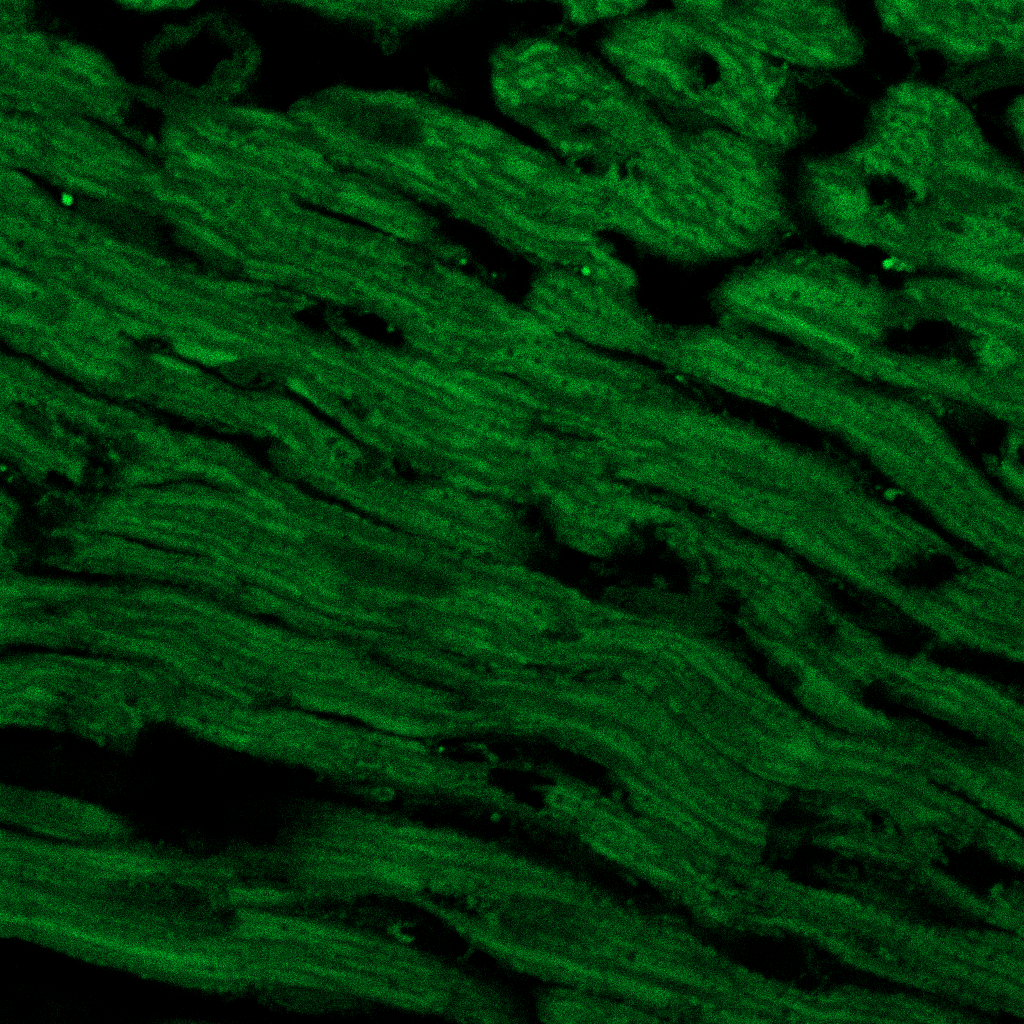

Supplement: Supplementary file 7 — Source data Fig. 4 [file 44321_2025_334_MOESM7_ESM.zip › Figure 4/4G/RBMS1-flox+TAC-α-ACTININ.tiff]

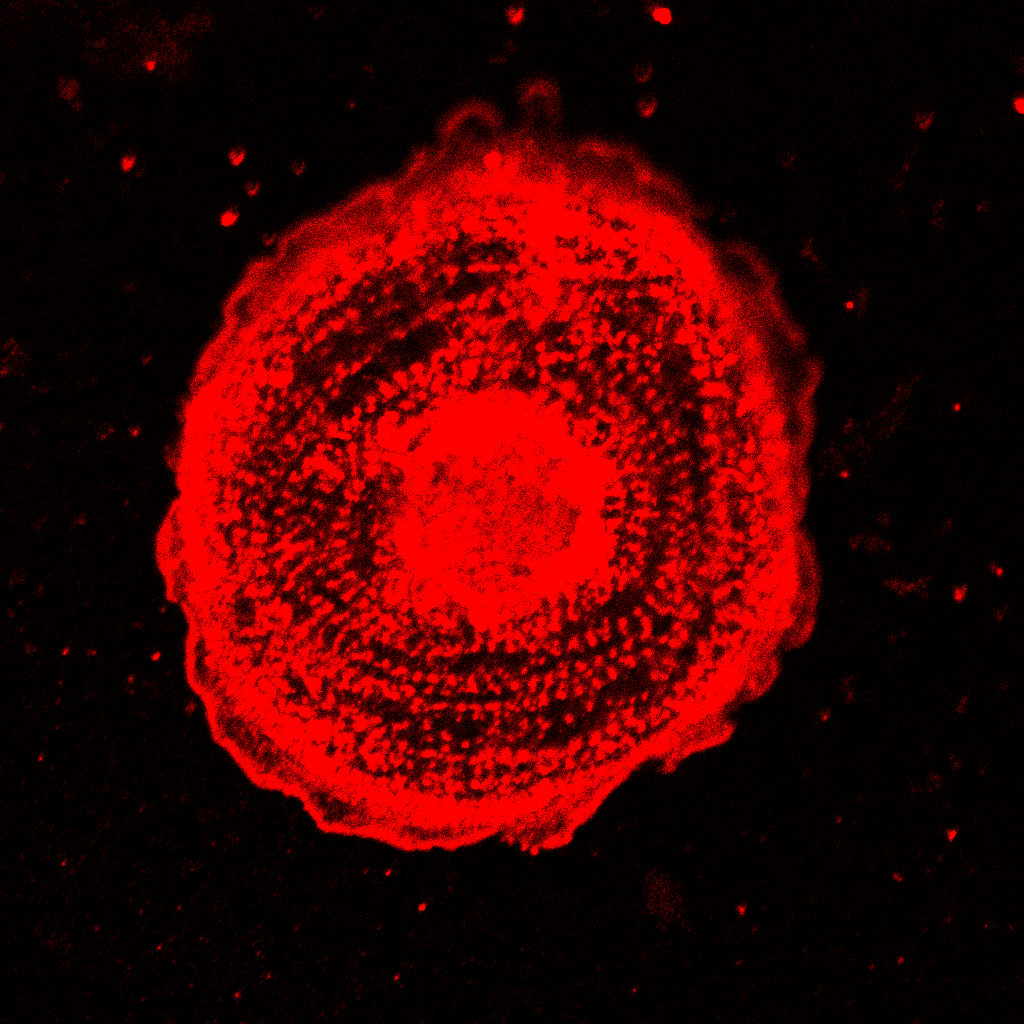

Supplement: Supplementary file 7 — Source data Fig. 4 [file 44321_2025_334_MOESM7_ESM.zip › Figure 4/4H/pcDNA3.1+Ang II-ACTN2.tiff]

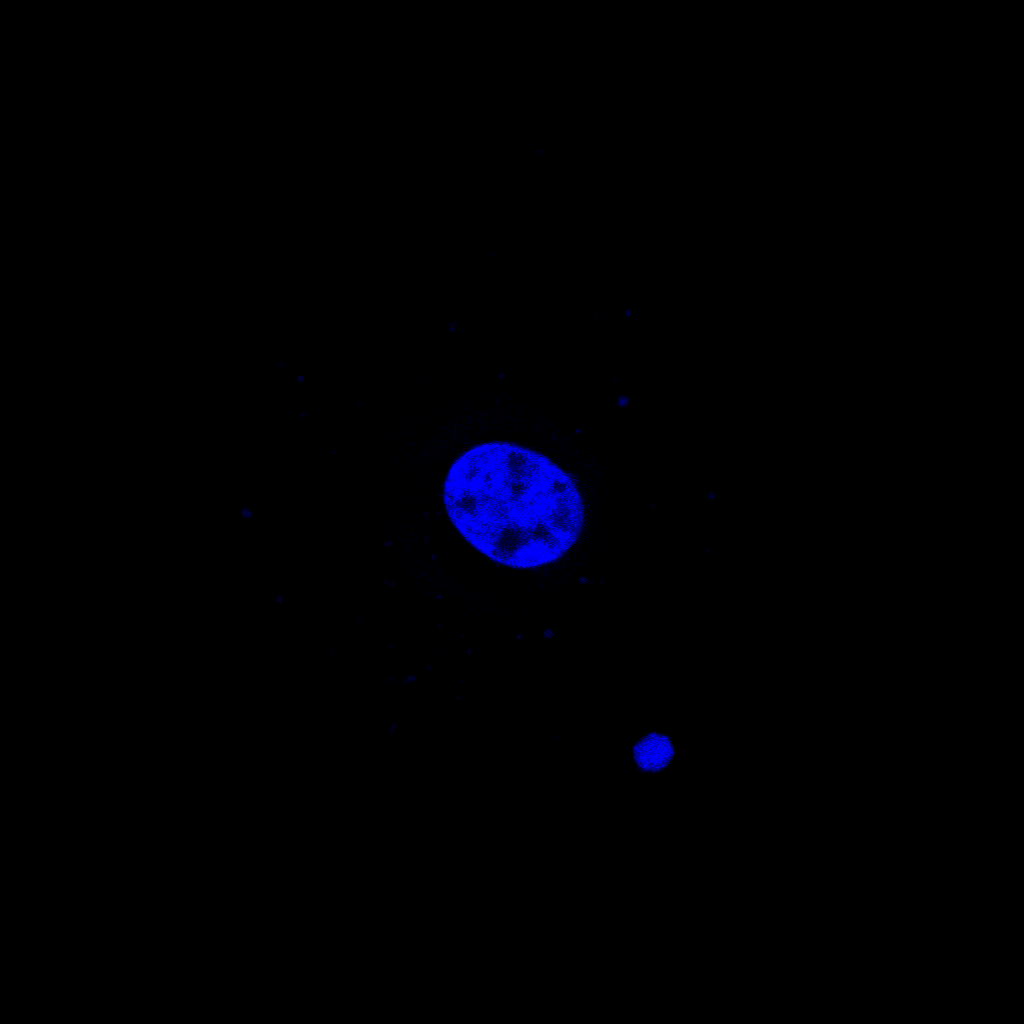

Supplement: Supplementary file 7 — Source data Fig. 4 [file 44321_2025_334_MOESM7_ESM.zip › Figure 4/4H/pcDNA3.1+Ang II-DAPI.tiff]

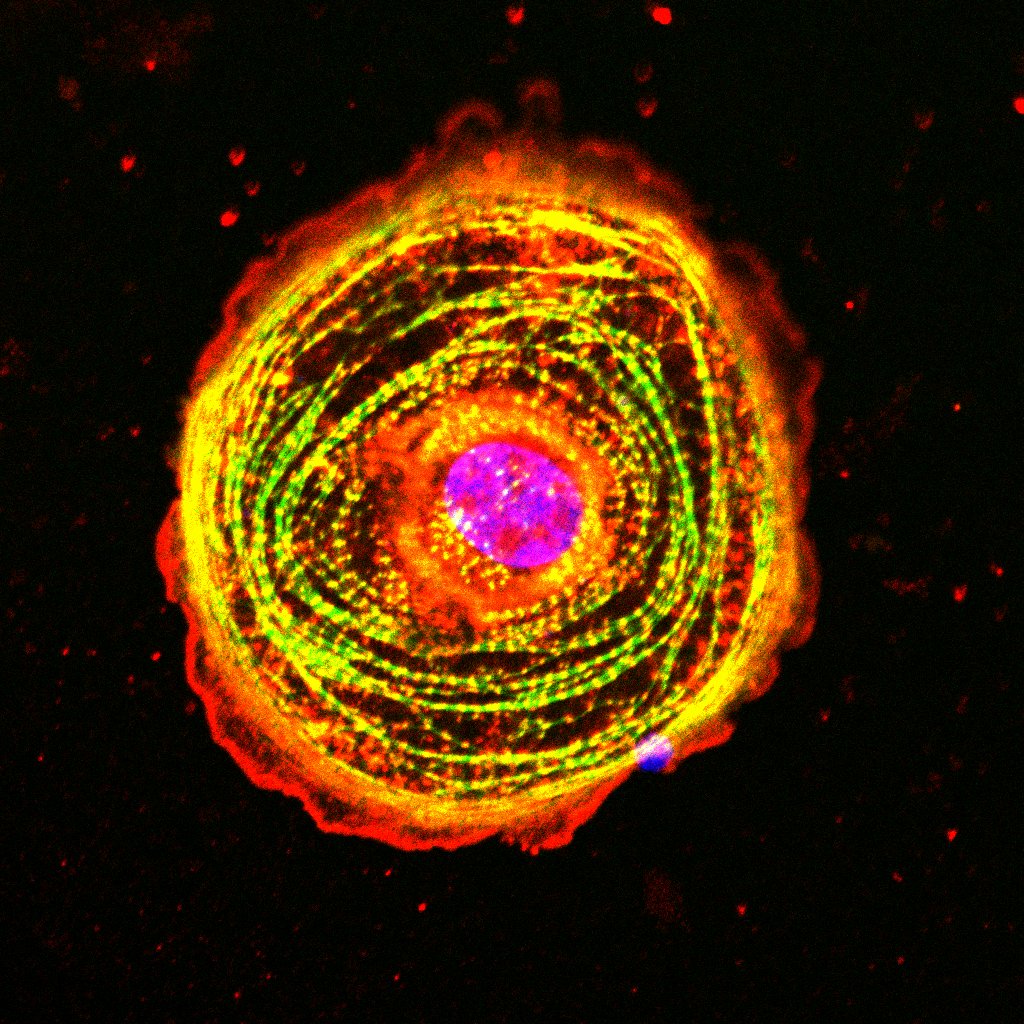

Supplement: Supplementary file 7 — Source data Fig. 4 [file 44321_2025_334_MOESM7_ESM.zip › Figure 4/4H/pcDNA3.1+Ang II-Merge.jpg]

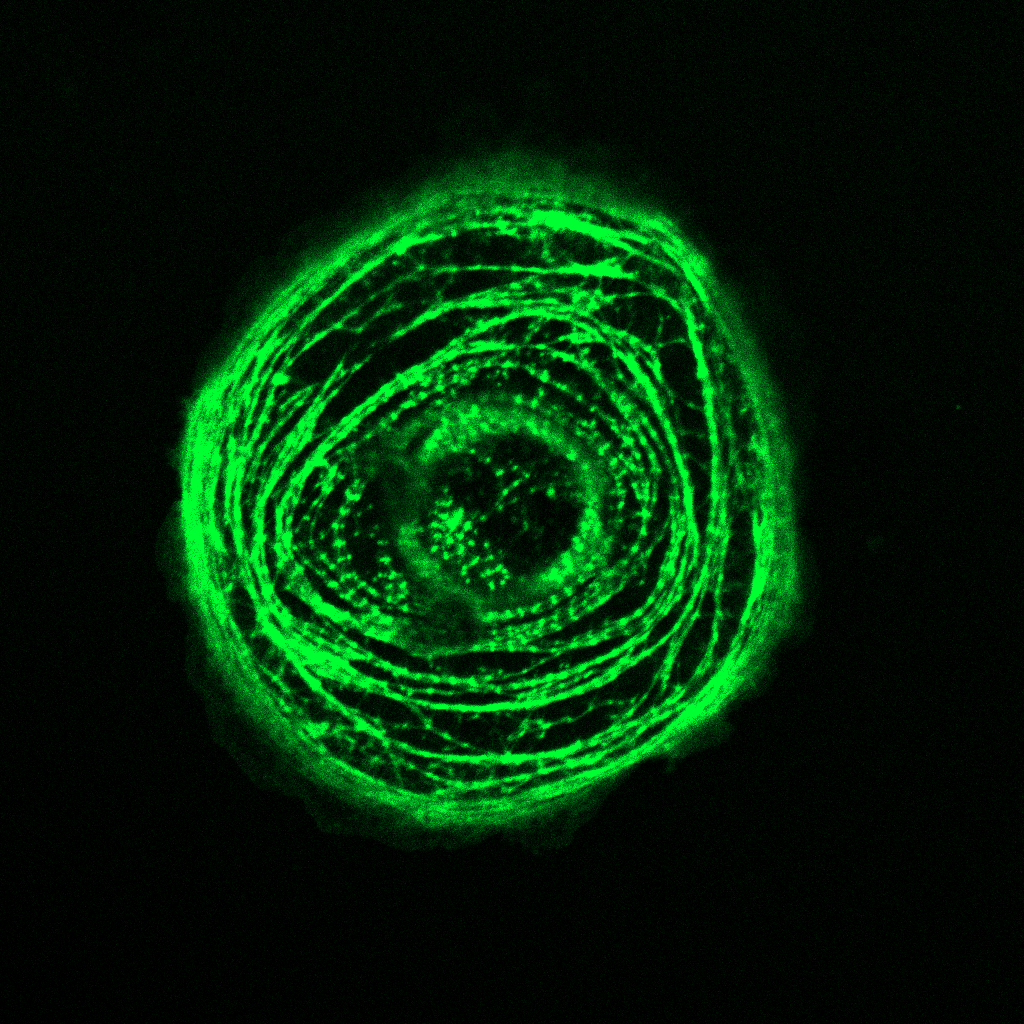

Supplement: Supplementary file 7 — Source data Fig. 4 [file 44321_2025_334_MOESM7_ESM.zip › Figure 4/4H/pcDNA3.1+Ang II-Phalloidine.tiff]

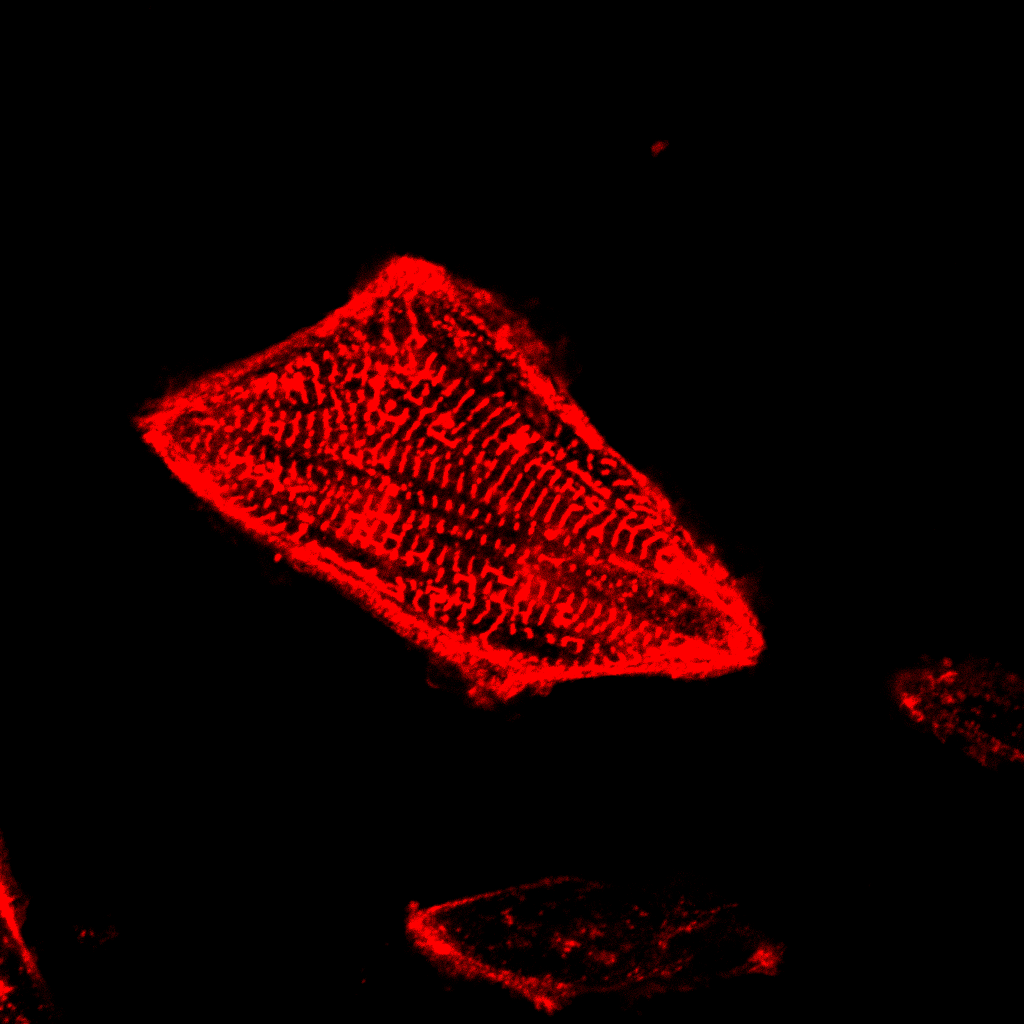

Supplement: Supplementary file 7 — Source data Fig. 4 [file 44321_2025_334_MOESM7_ESM.zip › Figure 4/4H/pcDNA3.1-ACTN2.tiff]

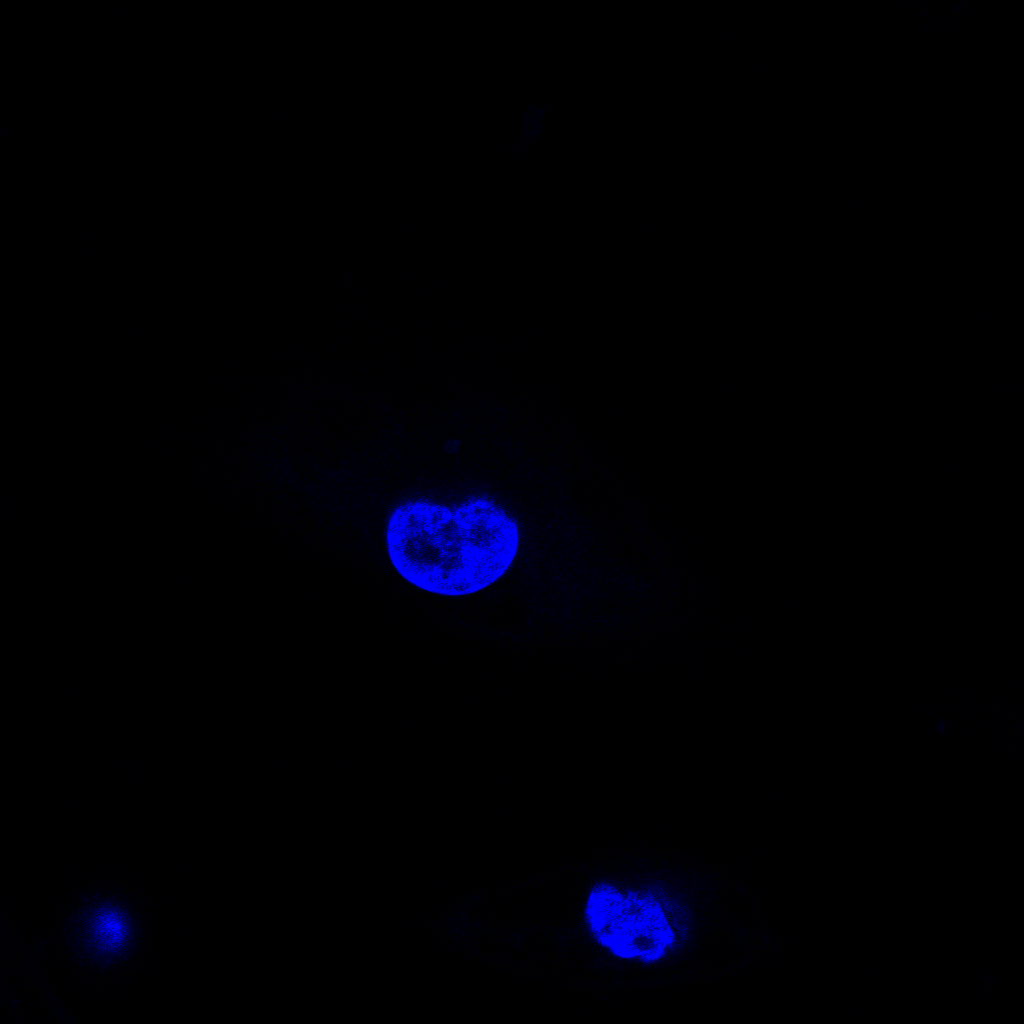

Supplement: Supplementary file 7 — Source data Fig. 4 [file 44321_2025_334_MOESM7_ESM.zip › Figure 4/4H/pcDNA3.1-DAPI.tiff]

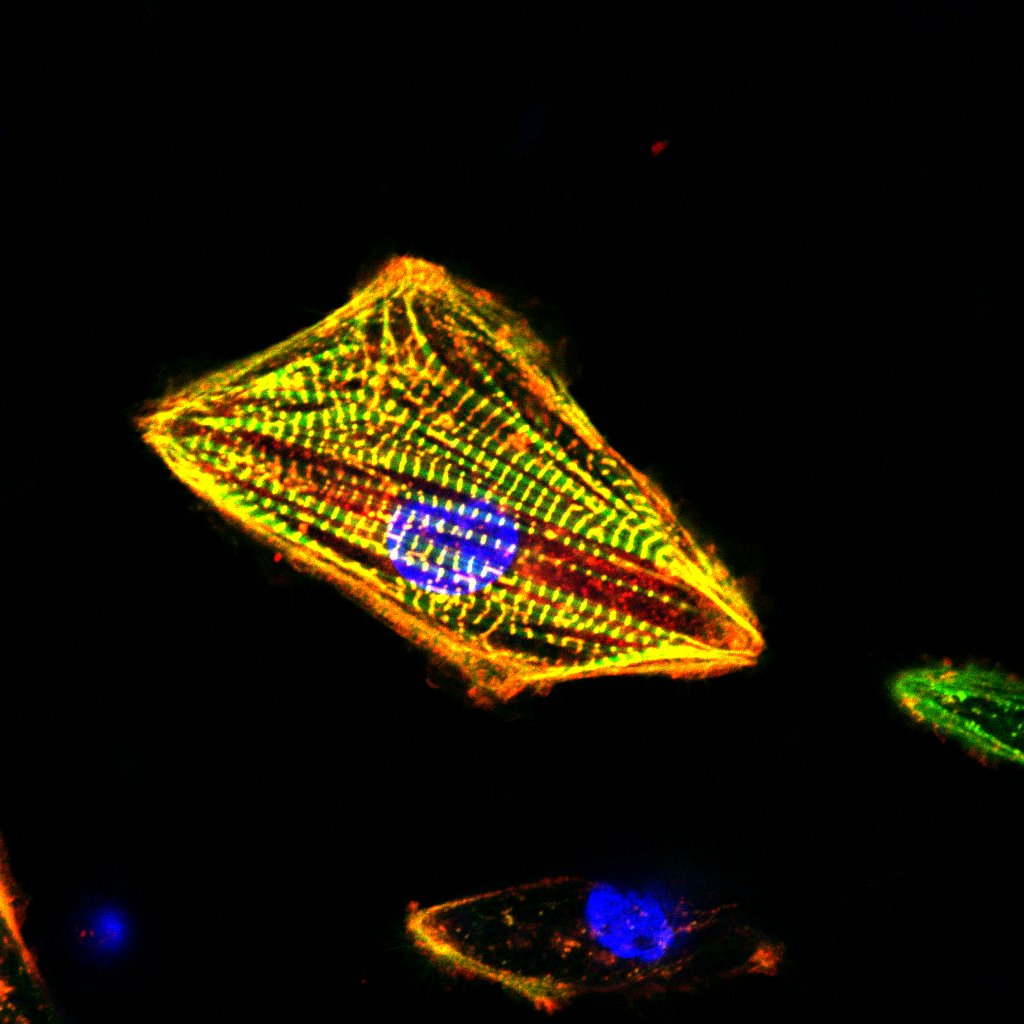

Supplement: Supplementary file 7 — Source data Fig. 4 [file 44321_2025_334_MOESM7_ESM.zip › Figure 4/4H/pcDNA3.1-Merge.jpg]

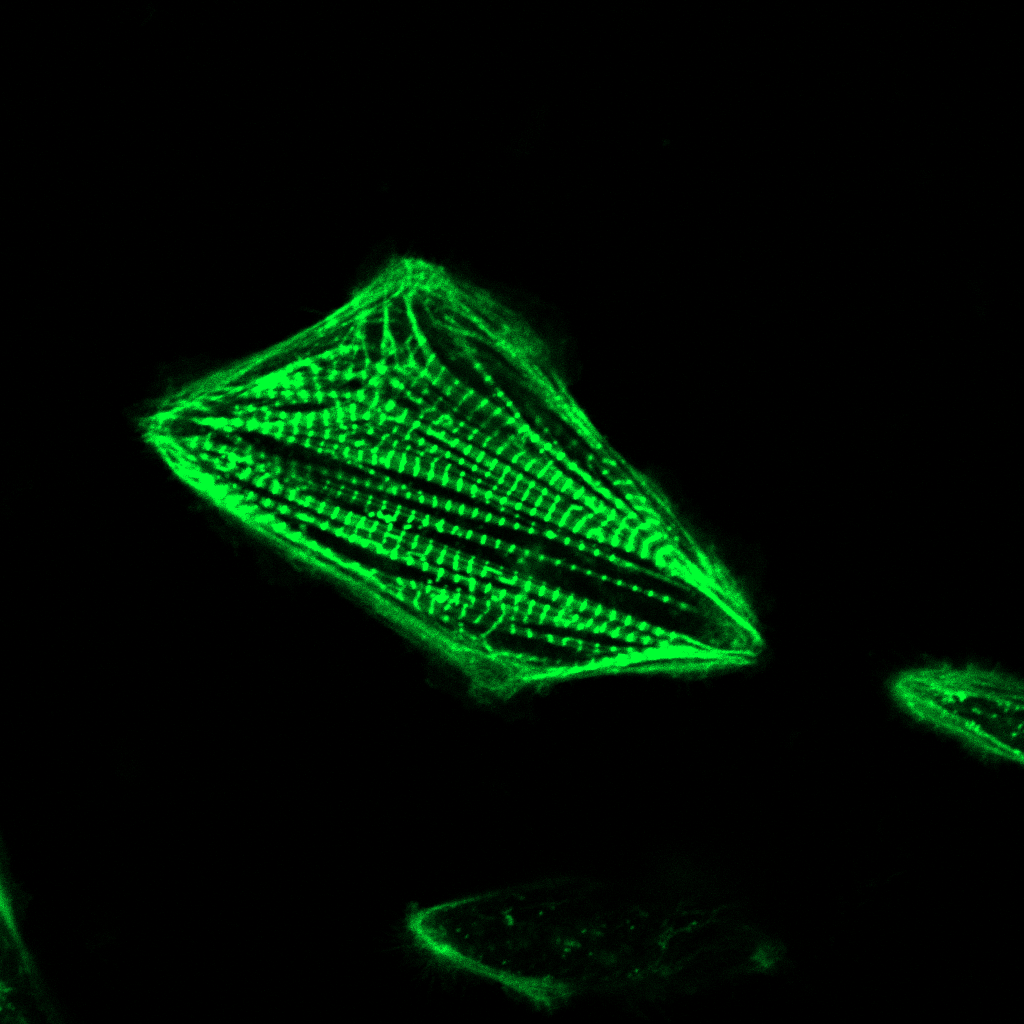

Supplement: Supplementary file 7 — Source data Fig. 4 [file 44321_2025_334_MOESM7_ESM.zip › Figure 4/4H/pcDNA3.1-Phalloidine.tiff]

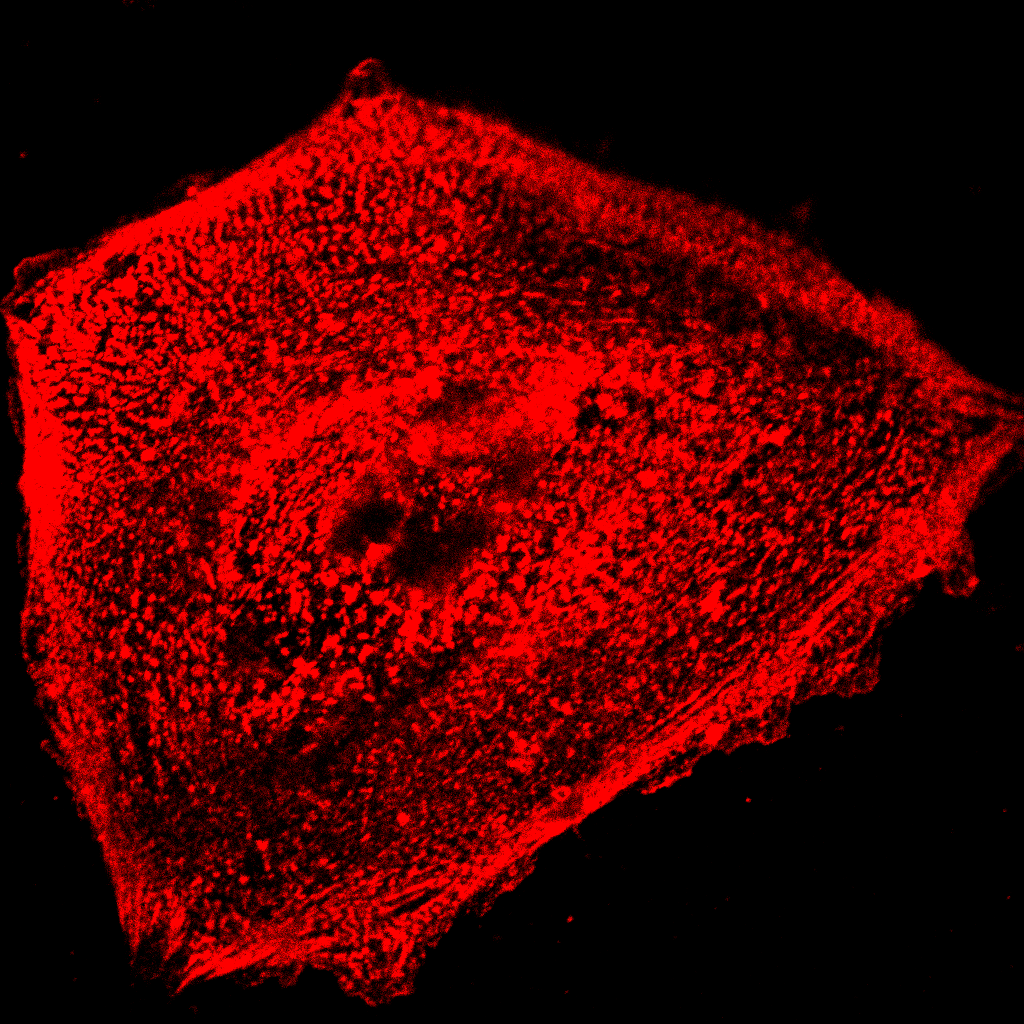

Supplement: Supplementary file 7 — Source data Fig. 4 [file 44321_2025_334_MOESM7_ESM.zip › Figure 4/4H/RBMS1+Ang II-ACTN2.tiff]

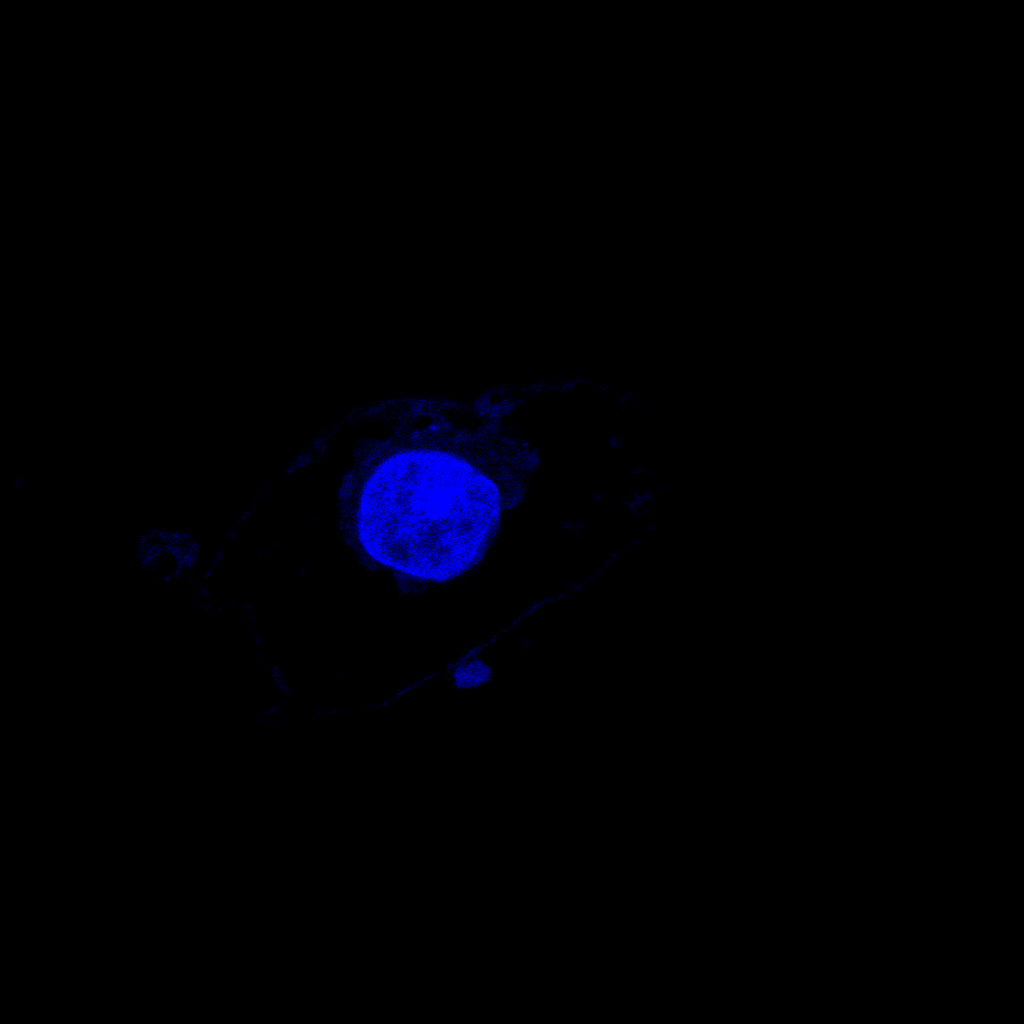

Supplement: Supplementary file 7 — Source data Fig. 4 [file 44321_2025_334_MOESM7_ESM.zip › Figure 4/4H/RBMS1+Ang II-DAPI.tiff]

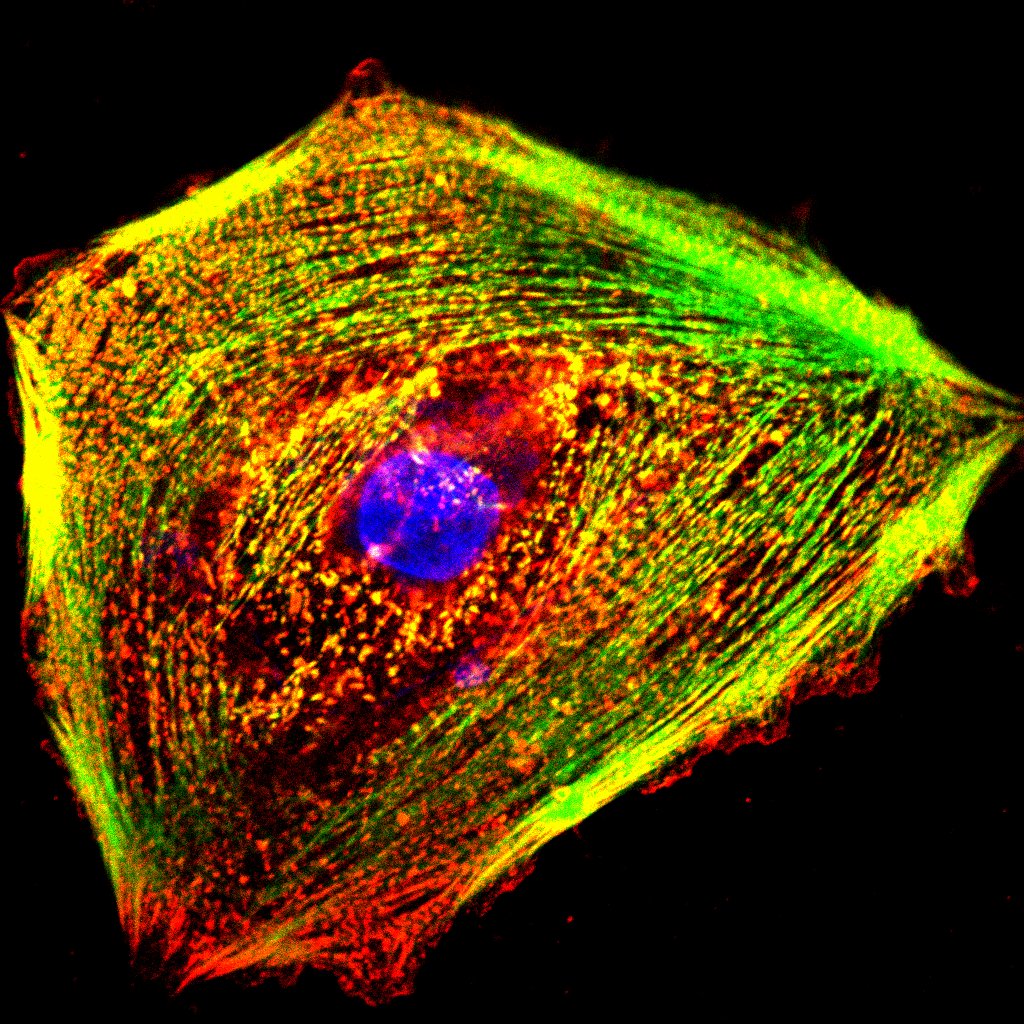

Supplement: Supplementary file 7 — Source data Fig. 4 [file 44321_2025_334_MOESM7_ESM.zip › Figure 4/4H/RBMS1+Ang II-Merge.jpg]

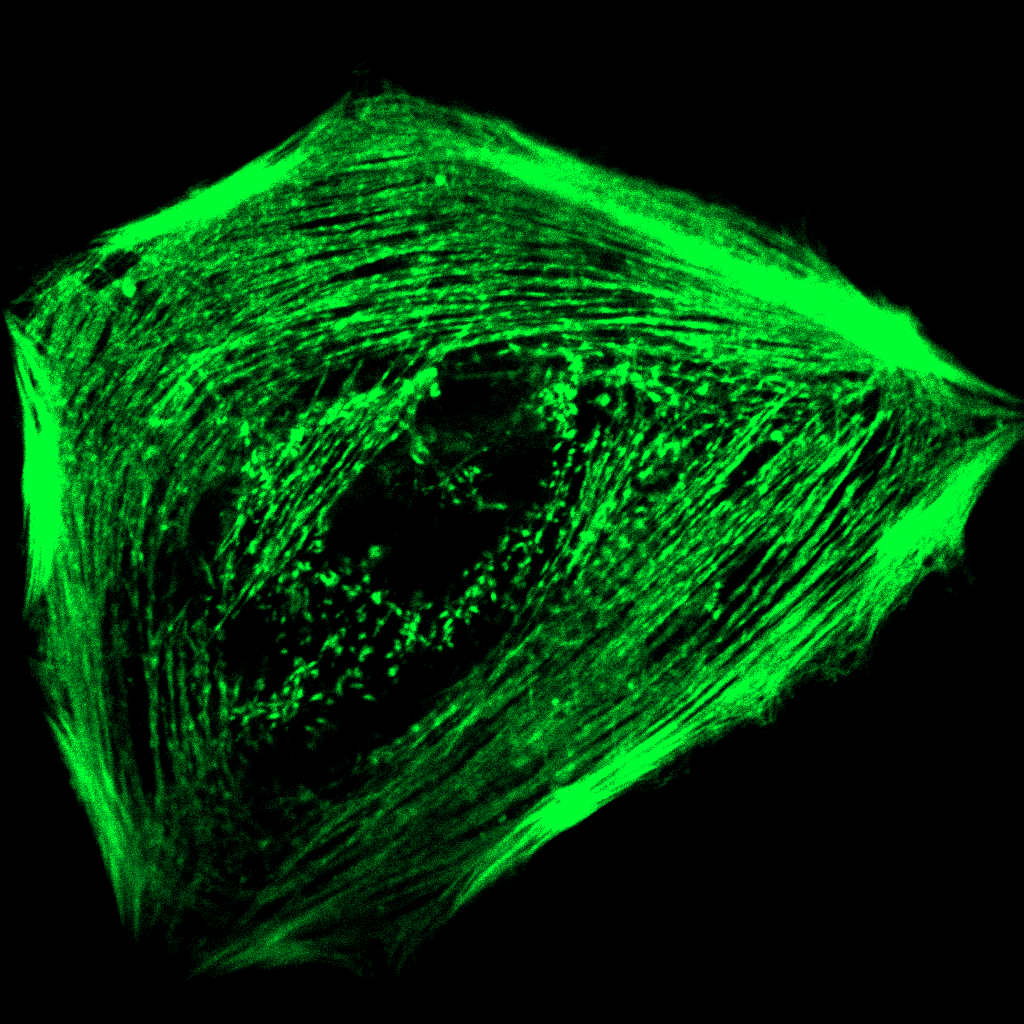

Supplement: Supplementary file 7 — Source data Fig. 4 [file 44321_2025_334_MOESM7_ESM.zip › Figure 4/4H/RBMS1+Ang II-Phalloidine.tiff]

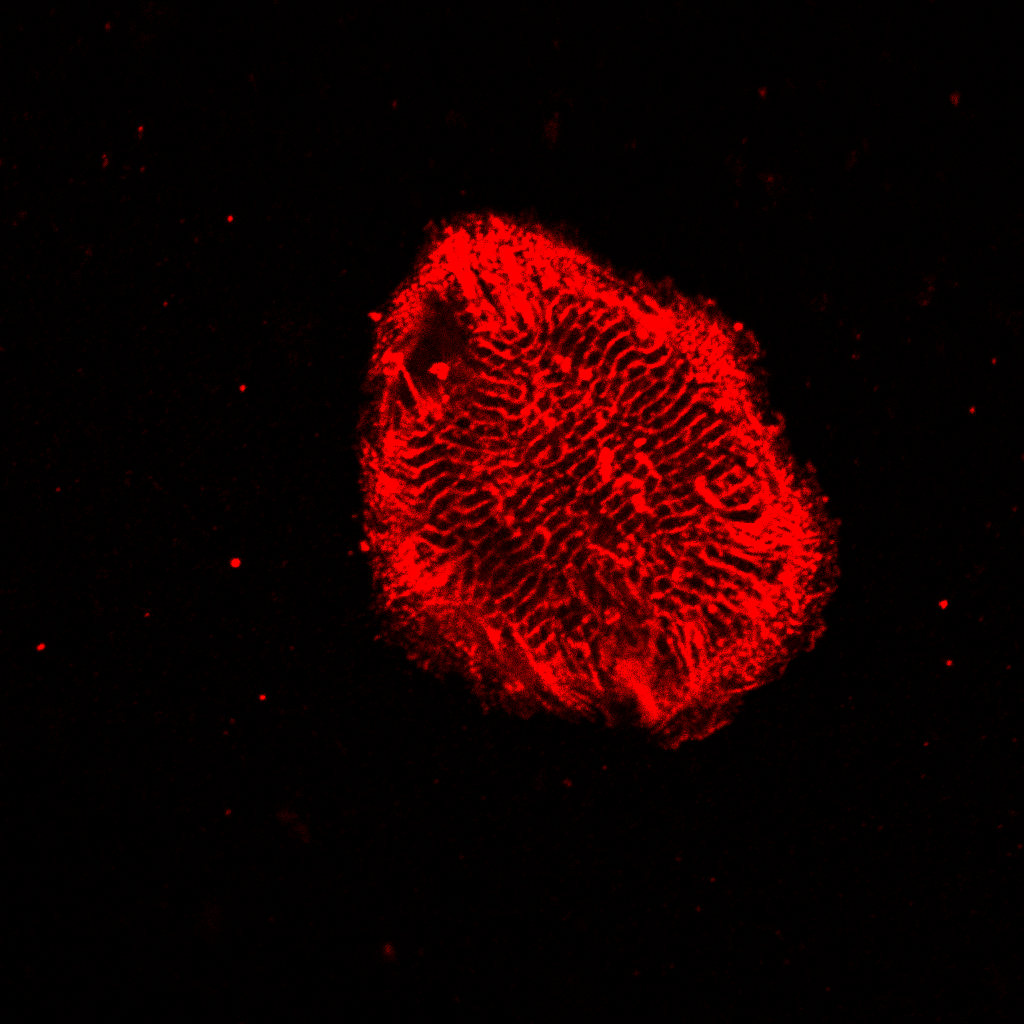

Supplement: Supplementary file 7 — Source data Fig. 4 [file 44321_2025_334_MOESM7_ESM.zip › Figure 4/4H/RBMS1-ACTN2.tiff]

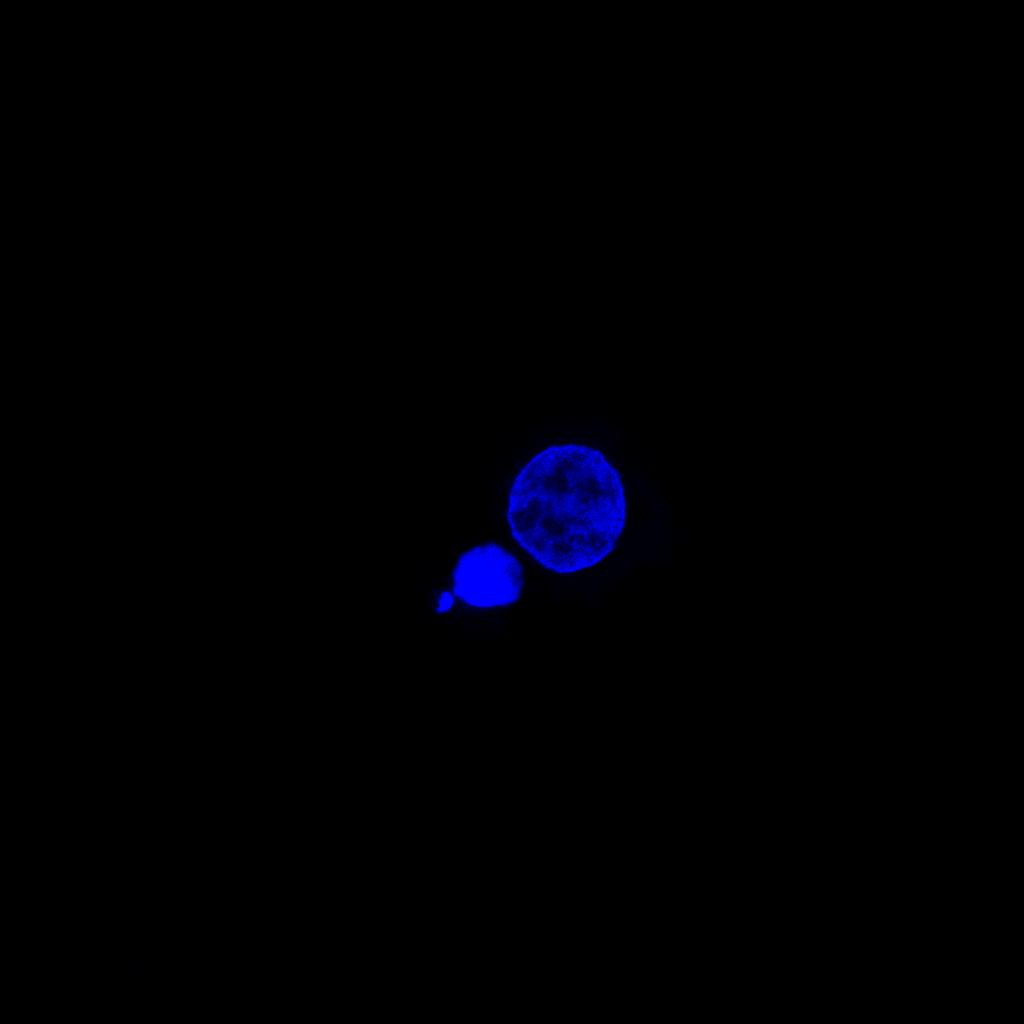

Supplement: Supplementary file 7 — Source data Fig. 4 [file 44321_2025_334_MOESM7_ESM.zip › Figure 4/4H/RBMS1-DAPI.tiff]

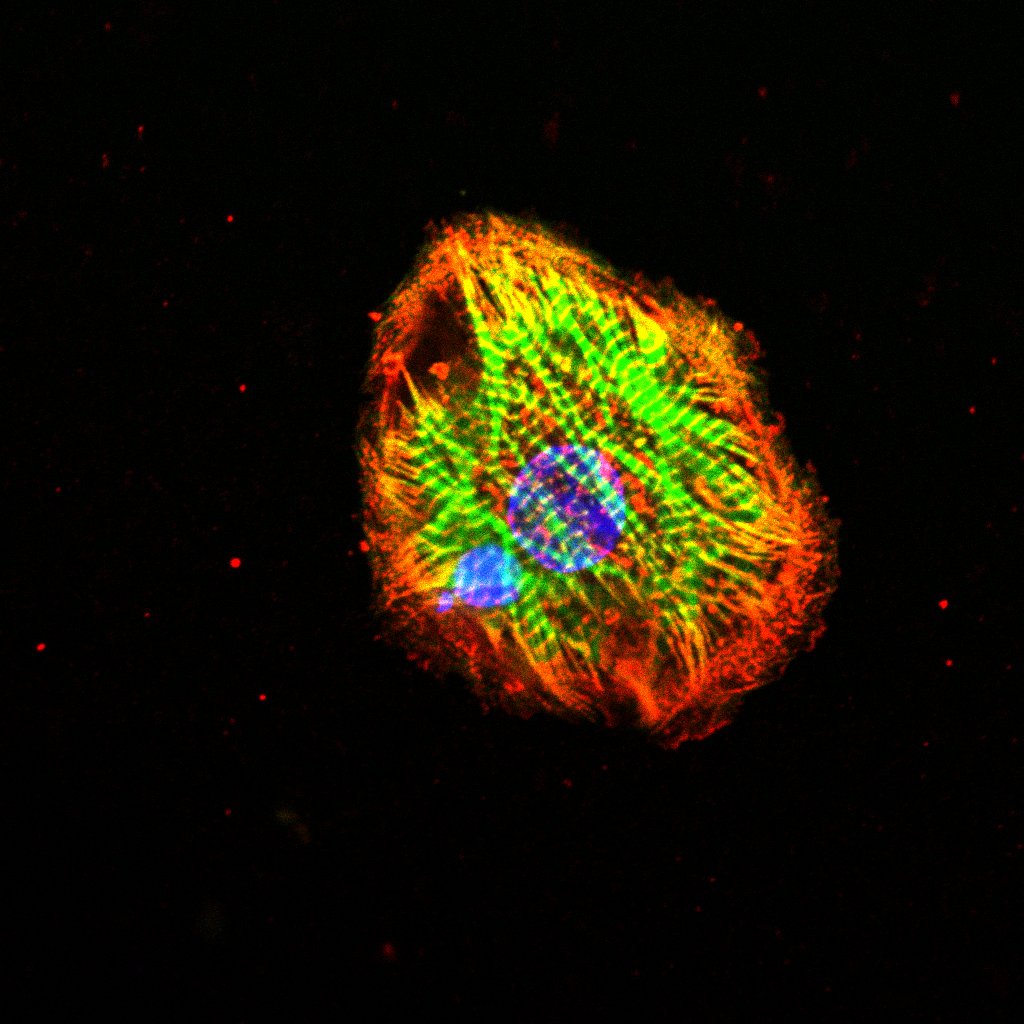

Supplement: Supplementary file 7 — Source data Fig. 4 [file 44321_2025_334_MOESM7_ESM.zip › Figure 4/4H/RBMS1-Merge.jpg]

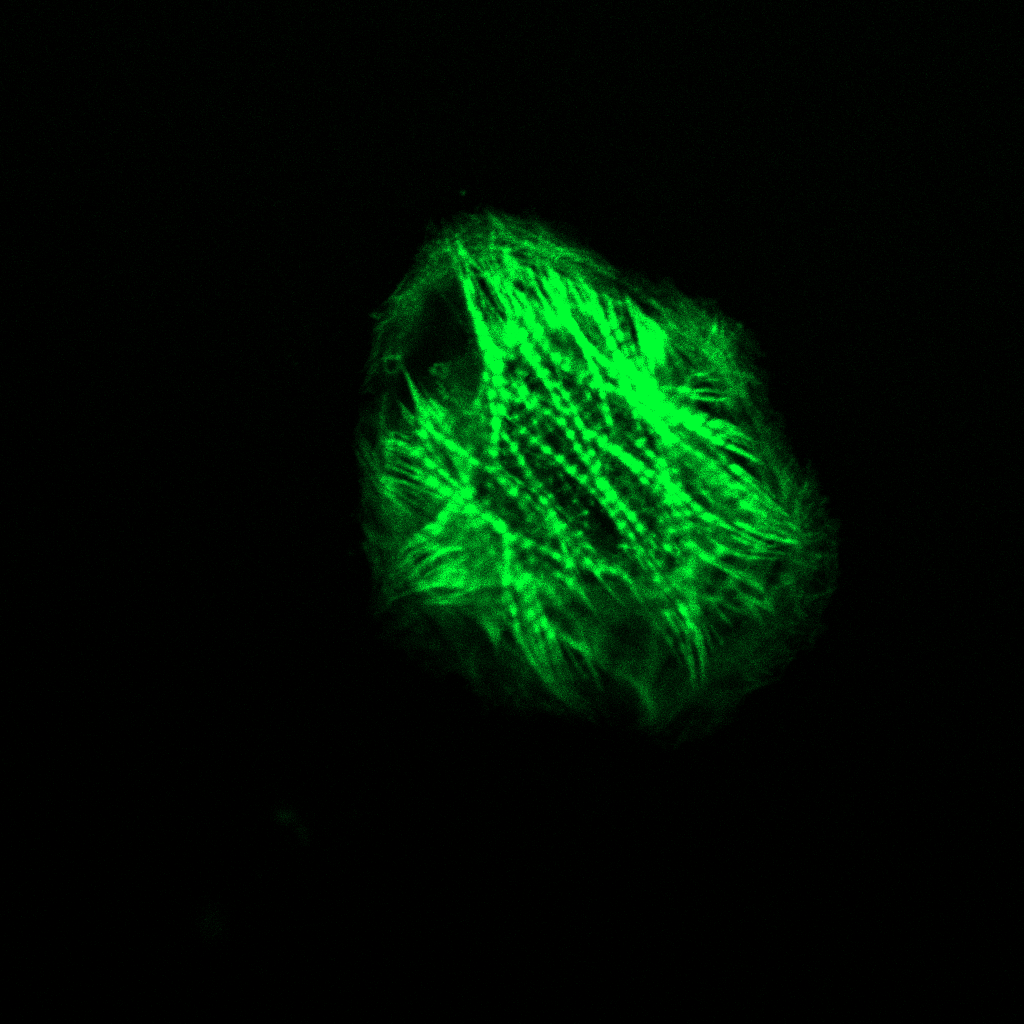

Supplement: Supplementary file 7 — Source data Fig. 4 [file 44321_2025_334_MOESM7_ESM.zip › Figure 4/4H/RBMS1-Phalloidine.tiff]

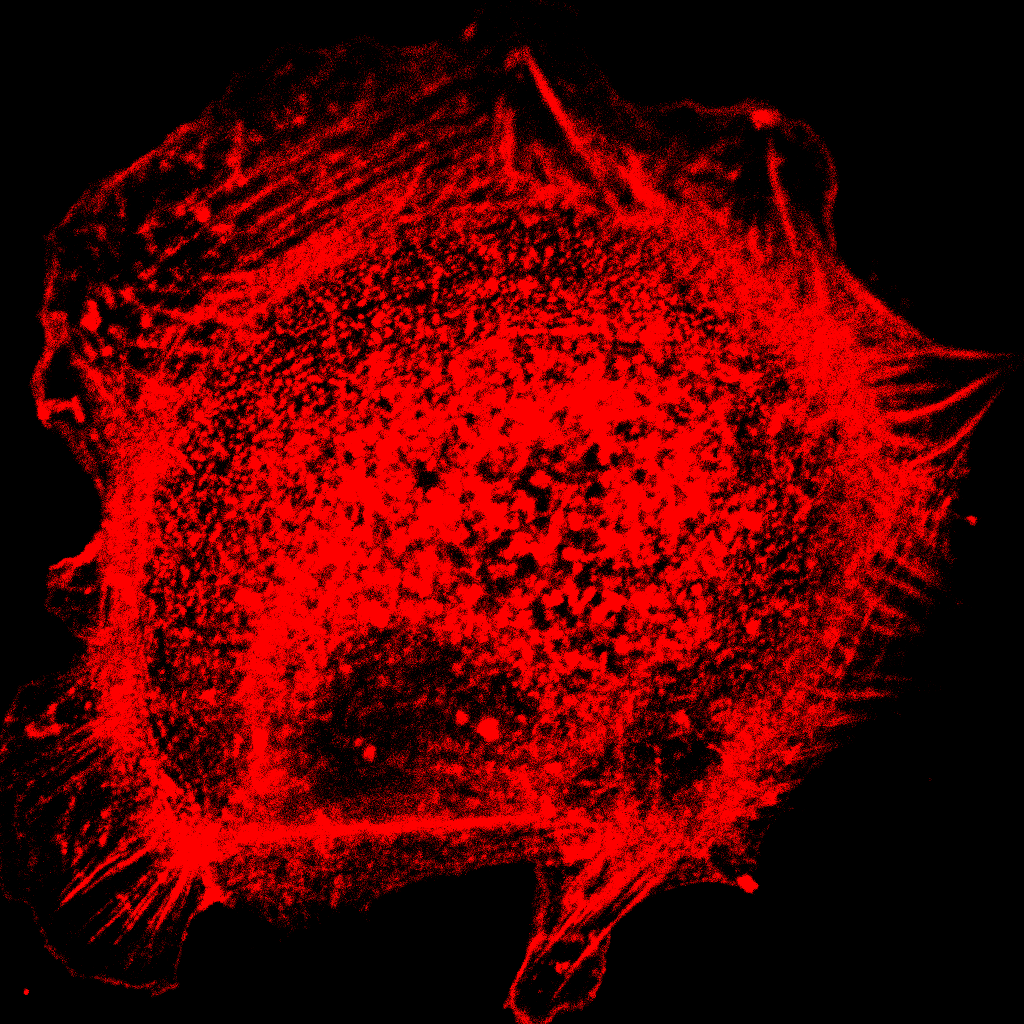

Supplement: Supplementary file 7 — Source data Fig. 4 [file 44321_2025_334_MOESM7_ESM.zip › Figure 4/4I/Ang II+si-NC-ACTN2.tiff]

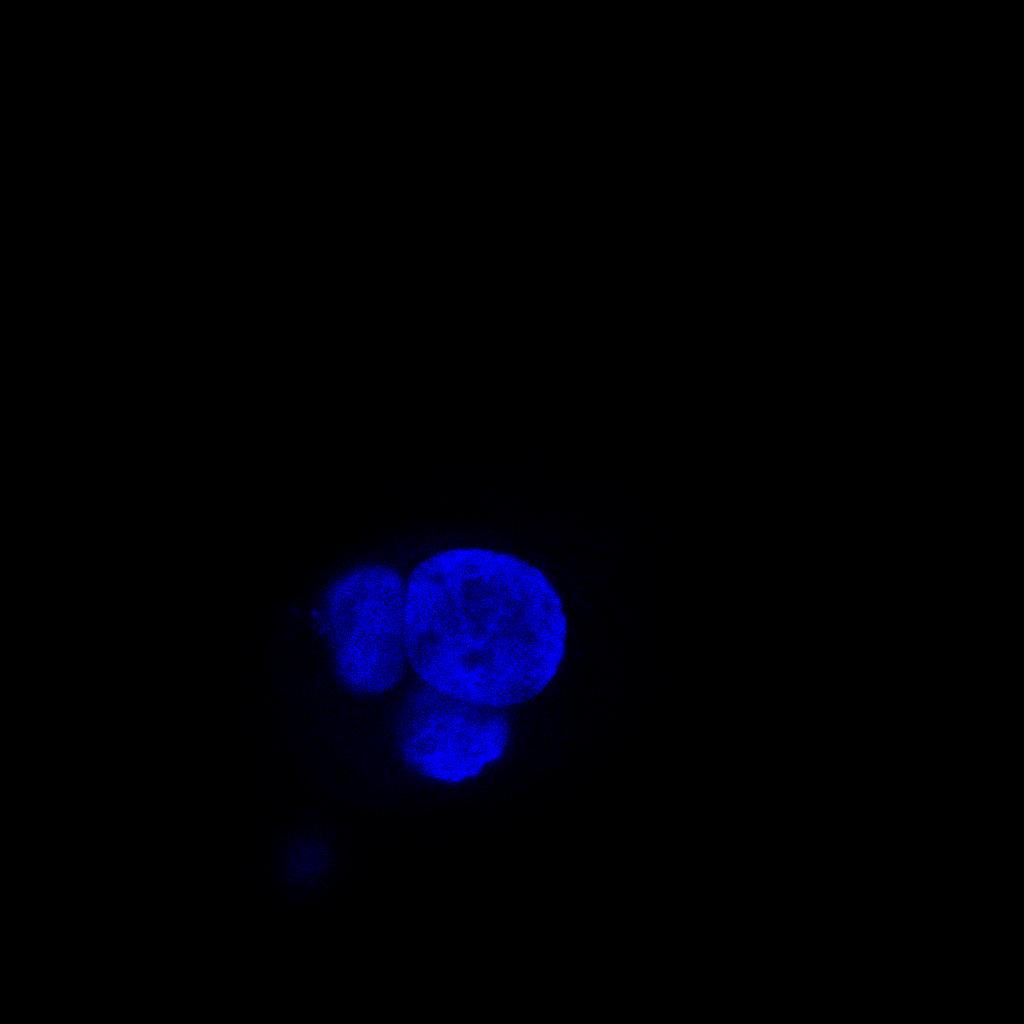

Supplement: Supplementary file 7 — Source data Fig. 4 [file 44321_2025_334_MOESM7_ESM.zip › Figure 4/4I/Ang II+si-NC-DAPI.tiff]

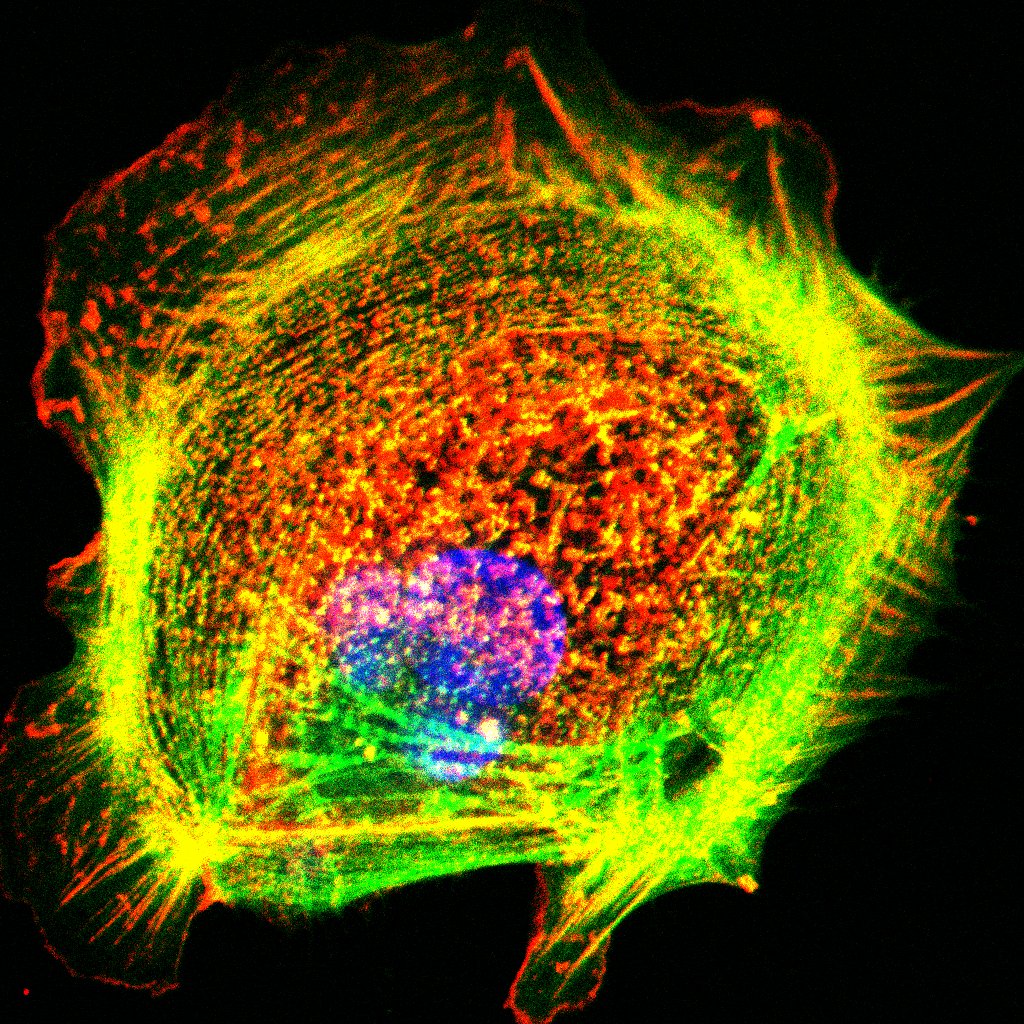

Supplement: Supplementary file 7 — Source data Fig. 4 [file 44321_2025_334_MOESM7_ESM.zip › Figure 4/4I/Ang II+si-NC-Merge.jpg]

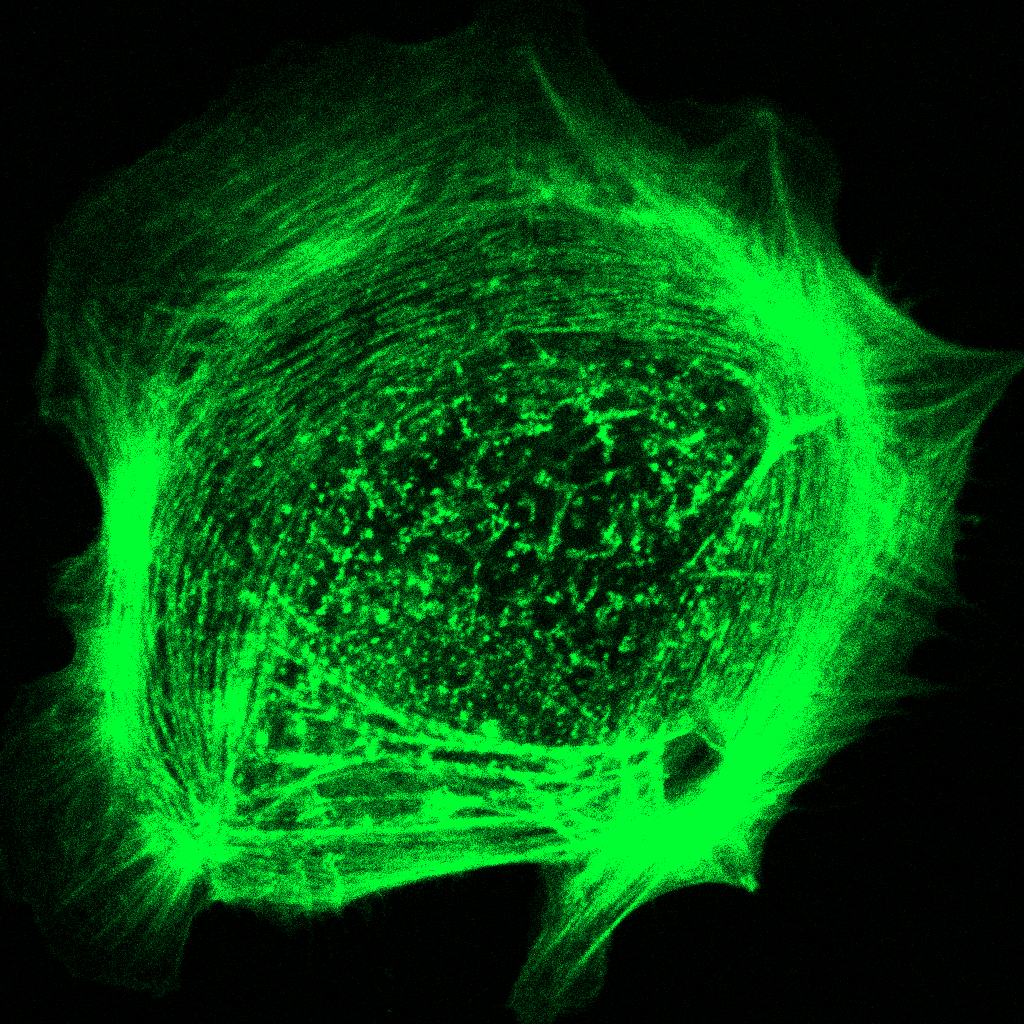

Supplement: Supplementary file 7 — Source data Fig. 4 [file 44321_2025_334_MOESM7_ESM.zip › Figure 4/4I/Ang II+si-NC-Phalloidine.tiff]

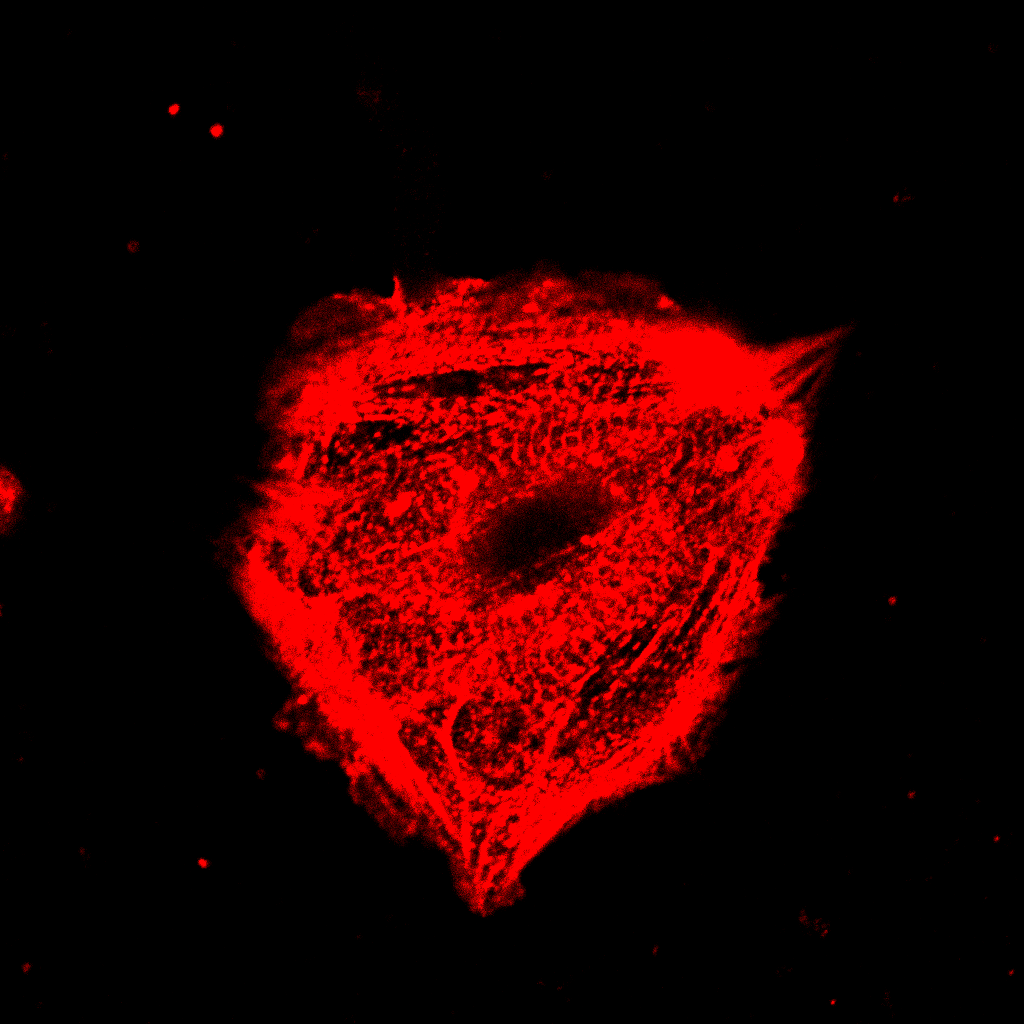

Supplement: Supplementary file 7 — Source data Fig. 4 [file 44321_2025_334_MOESM7_ESM.zip › Figure 4/4I/Ang II+si-RBMS1-ACTN2.tiff]

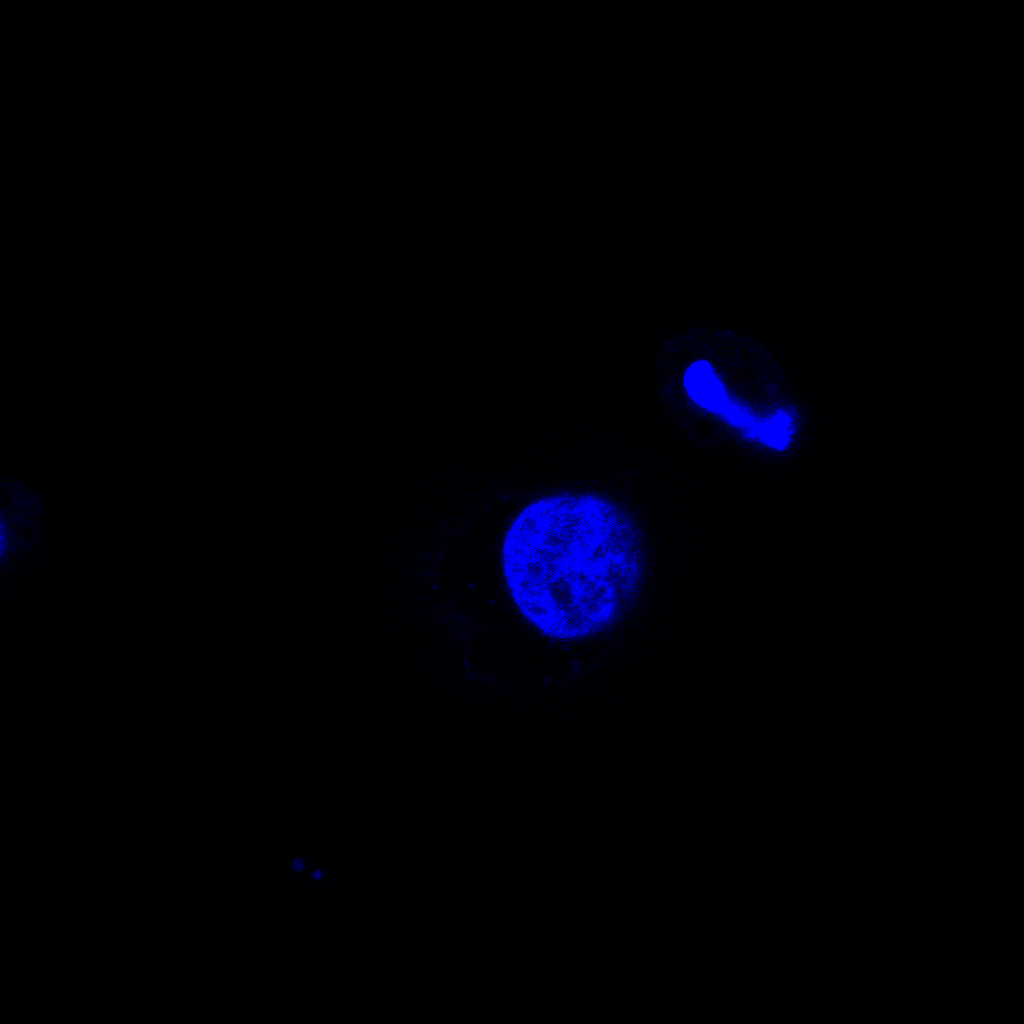

Supplement: Supplementary file 7 — Source data Fig. 4 [file 44321_2025_334_MOESM7_ESM.zip › Figure 4/4I/Ang II+si-RBMS1-DAPI.tiff]

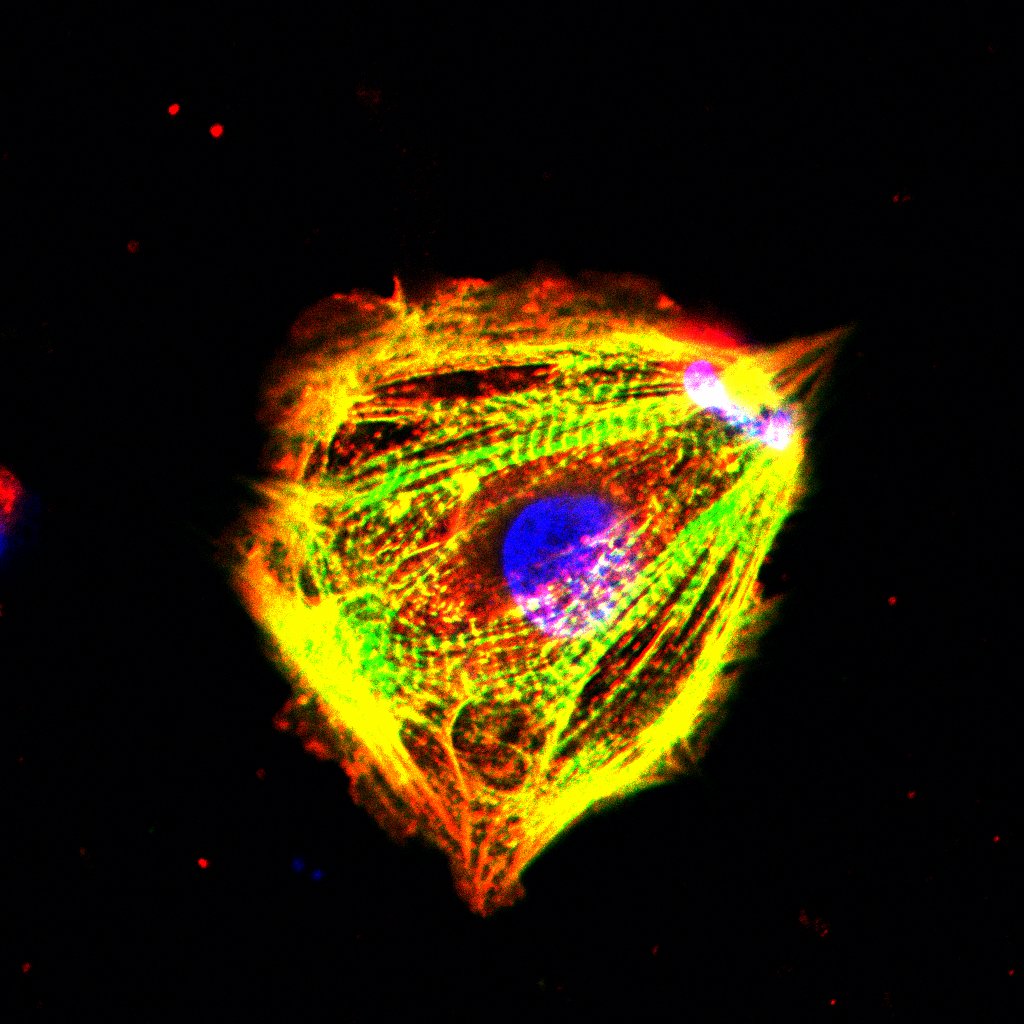

Supplement: Supplementary file 7 — Source data Fig. 4 [file 44321_2025_334_MOESM7_ESM.zip › Figure 4/4I/Ang II+si-RBMS1-Merge.jpg]

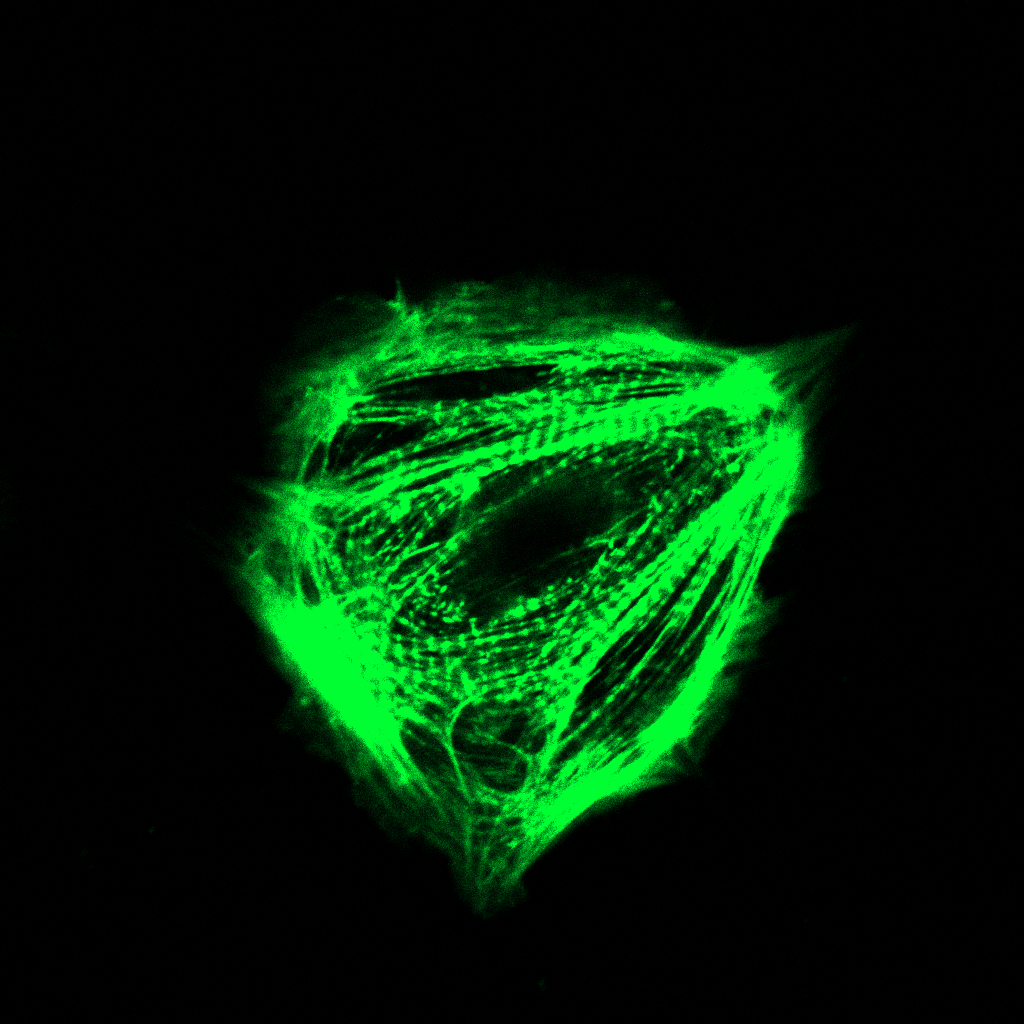

Supplement: Supplementary file 7 — Source data Fig. 4 [file 44321_2025_334_MOESM7_ESM.zip › Figure 4/4I/Ang II+si-RBMS1-Phalloidine.tiff]

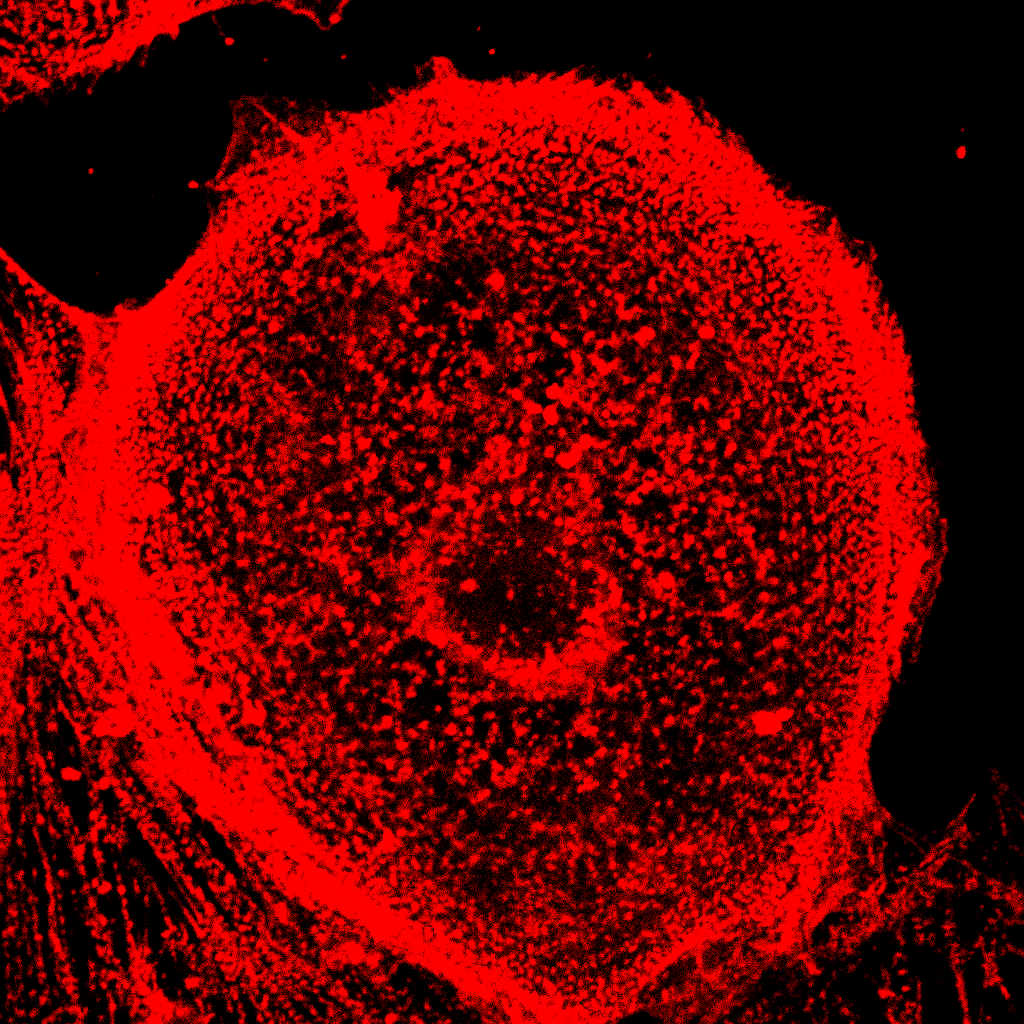

Supplement: Supplementary file 7 — Source data Fig. 4 [file 44321_2025_334_MOESM7_ESM.zip › Figure 4/4I/Ang II-ACTN2.tiff]

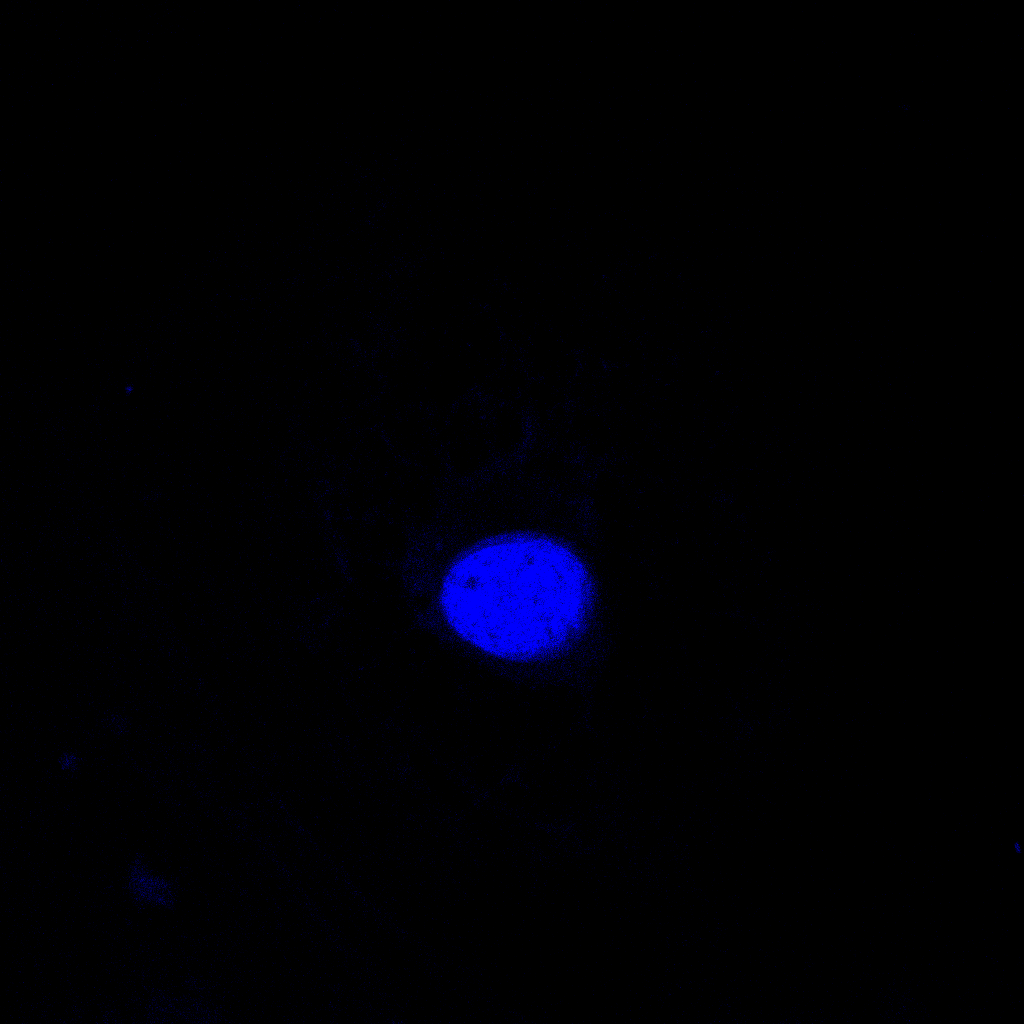

Supplement: Supplementary file 7 — Source data Fig. 4 [file 44321_2025_334_MOESM7_ESM.zip › Figure 4/4I/Ang II-DAPI.tiff]

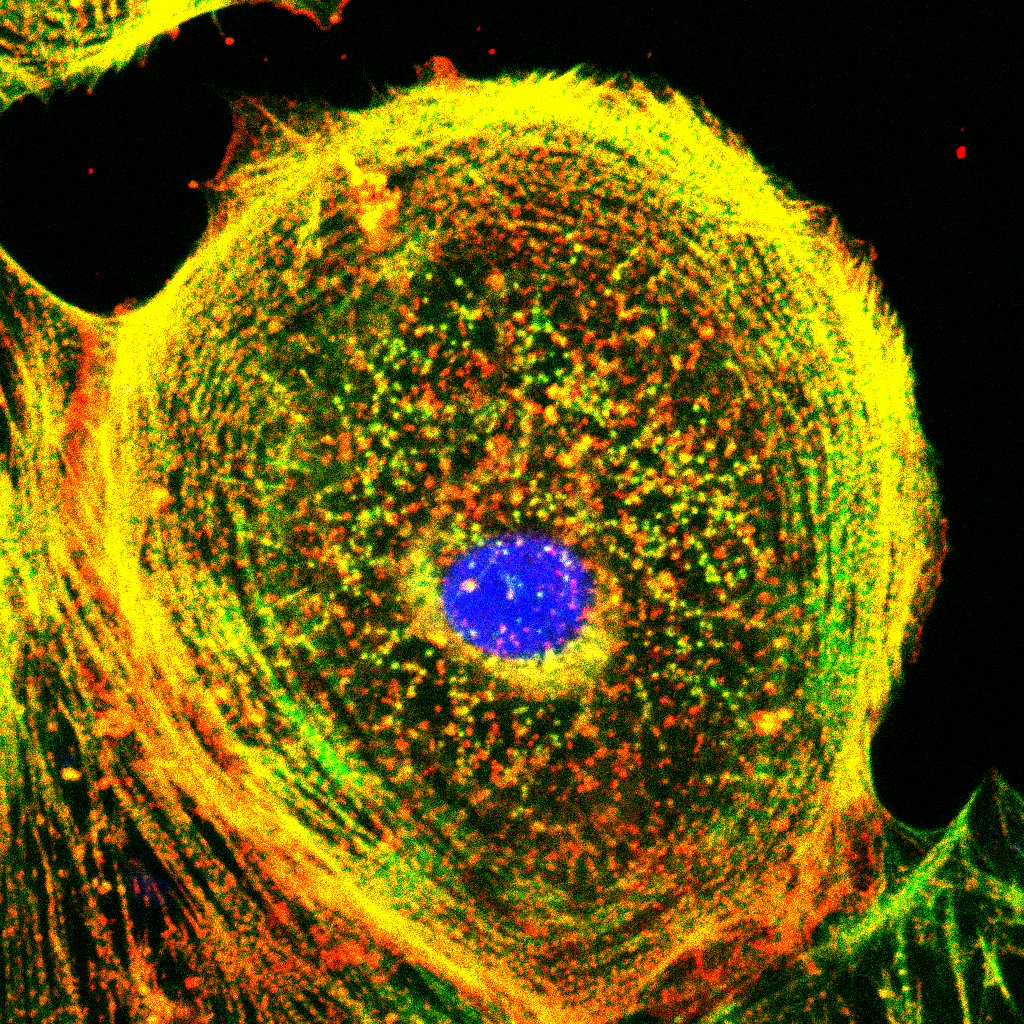

Supplement: Supplementary file 7 — Source data Fig. 4 [file 44321_2025_334_MOESM7_ESM.zip › Figure 4/4I/Ang II-Merge.jpg]

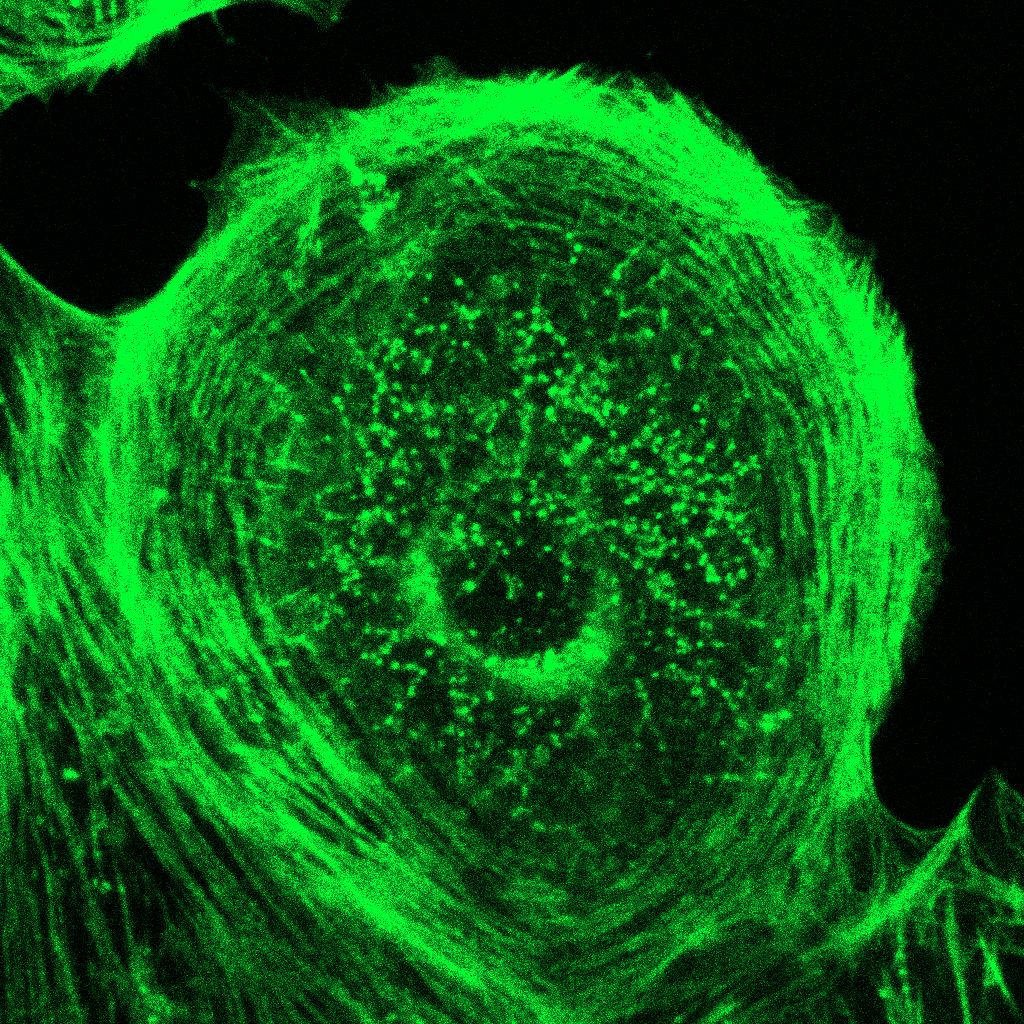

Supplement: Supplementary file 7 — Source data Fig. 4 [file 44321_2025_334_MOESM7_ESM.zip › Figure 4/4I/Ang II-Phalloidine.tiff]

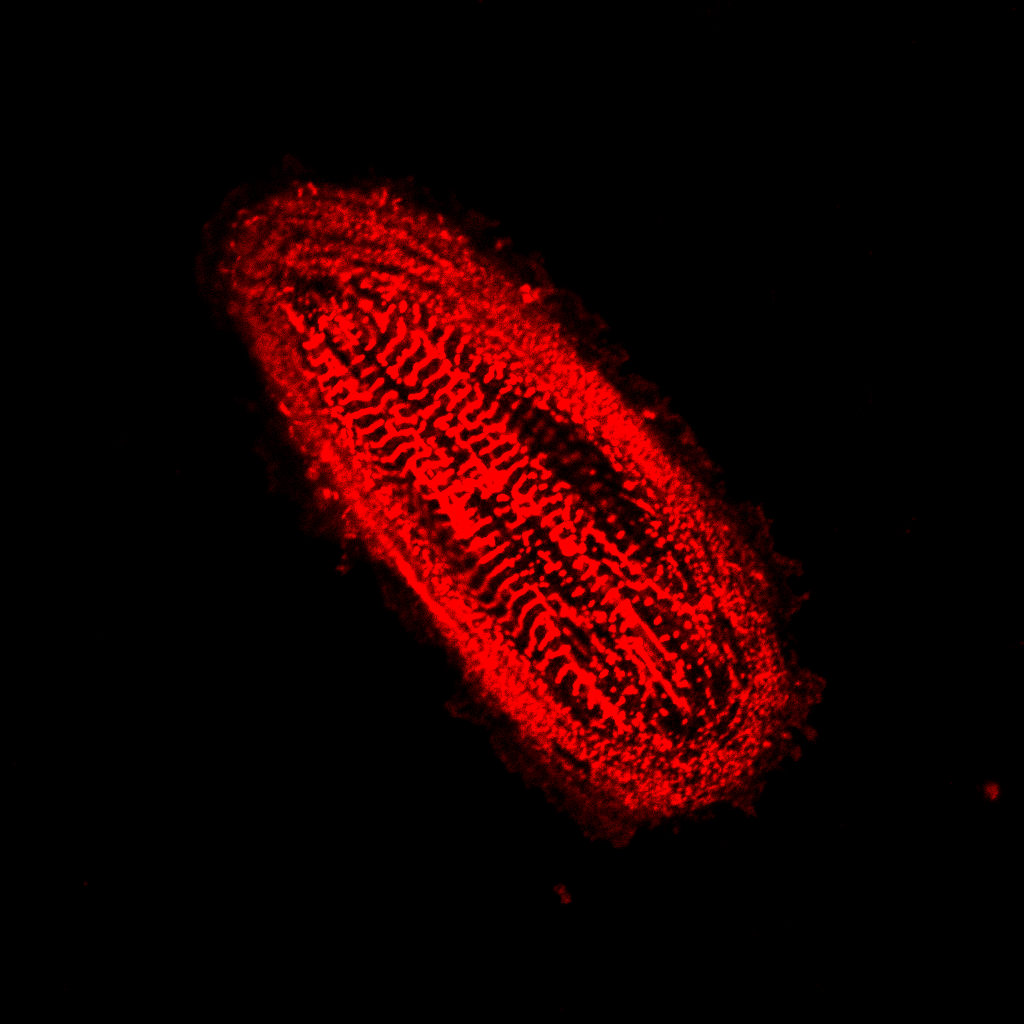

Supplement: Supplementary file 7 — Source data Fig. 4 [file 44321_2025_334_MOESM7_ESM.zip › Figure 4/4I/Control-ACTN2.tiff]

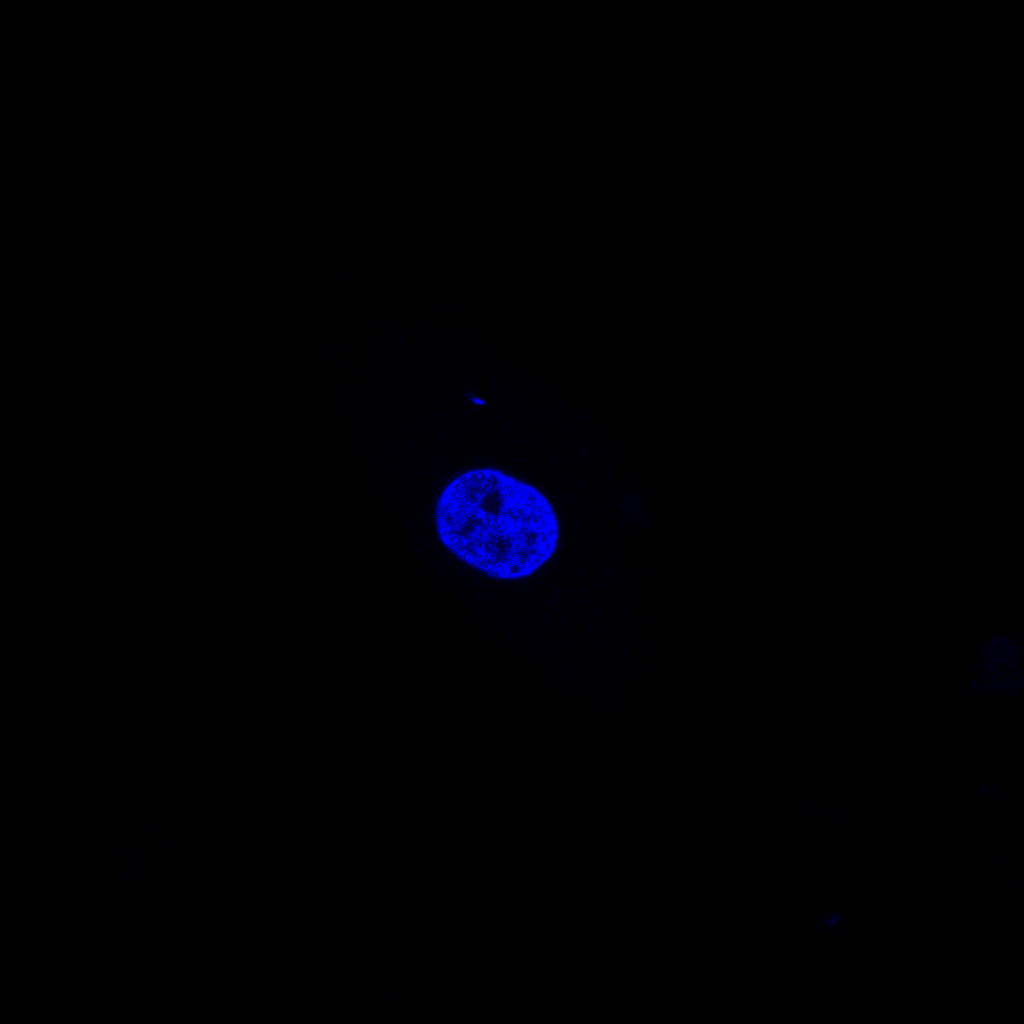

Supplement: Supplementary file 7 — Source data Fig. 4 [file 44321_2025_334_MOESM7_ESM.zip › Figure 4/4I/Control-DAPI.tiff]

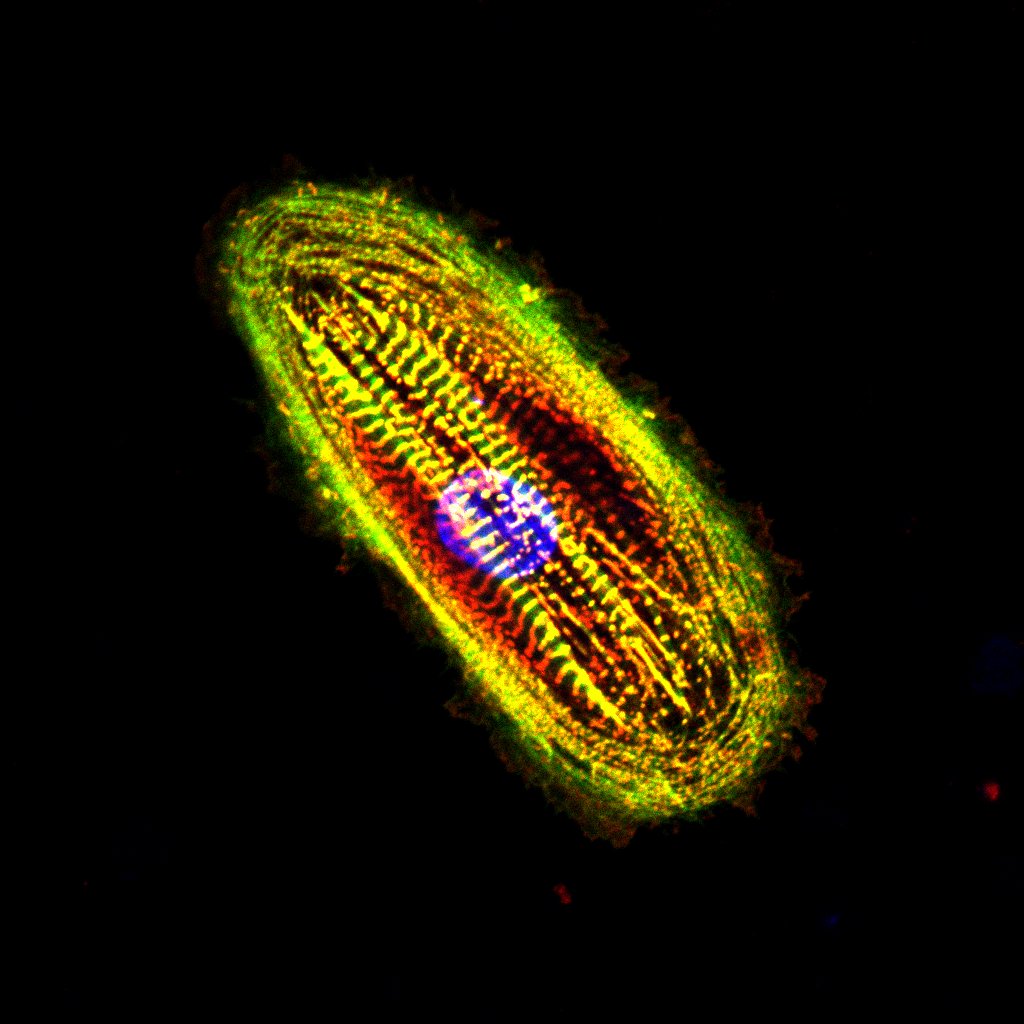

Supplement: Supplementary file 7 — Source data Fig. 4 [file 44321_2025_334_MOESM7_ESM.zip › Figure 4/4I/Control-Merge.jpg]

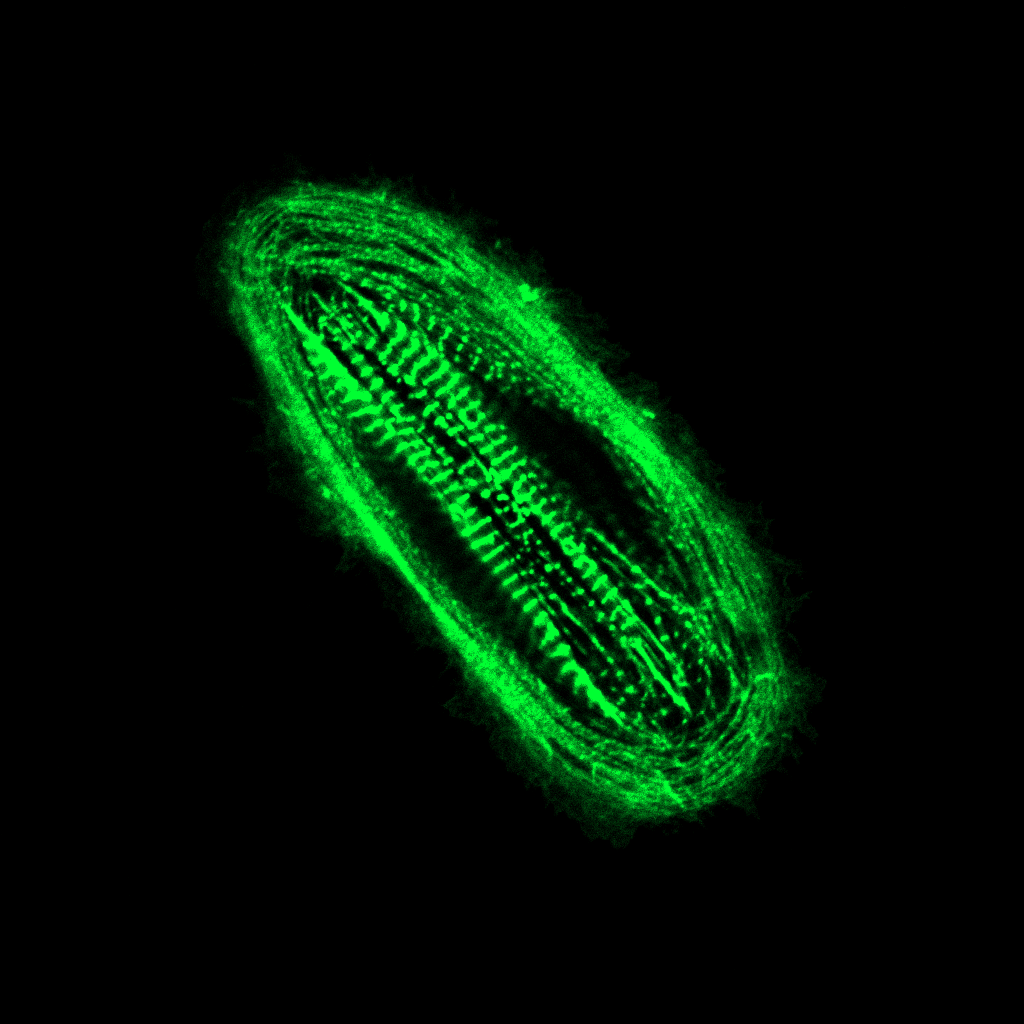

Supplement: Supplementary file 7 — Source data Fig. 4 [file 44321_2025_334_MOESM7_ESM.zip › Figure 4/4I/Control-Phalloidine.tiff]

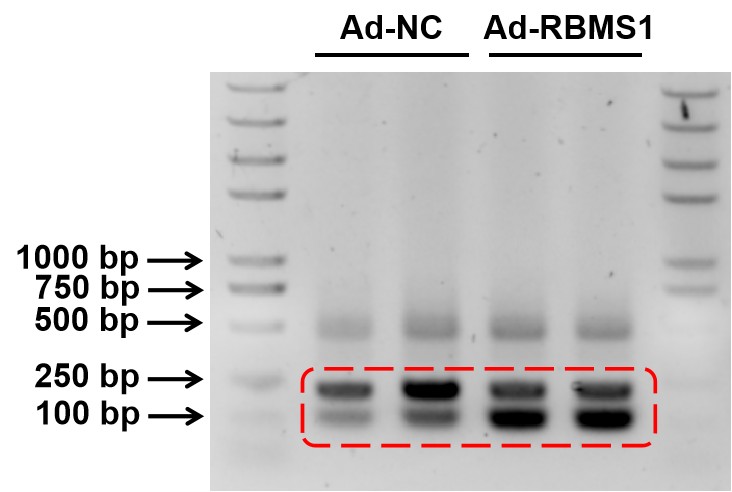

Supplement: Supplementary file 8 — Source data Fig. 5 [file 44321_2025_334_MOESM8_ESM.zip › Figure 5/5C/5C.jpg]

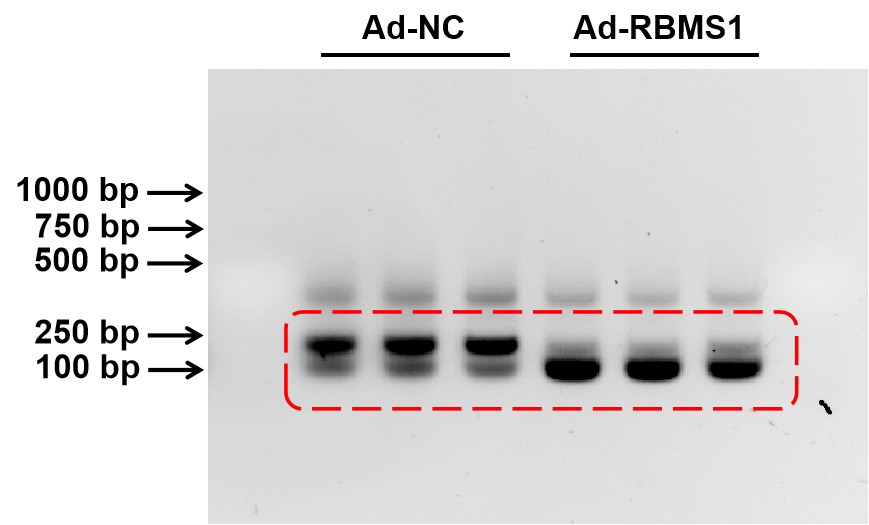

Supplement: Supplementary file 8 — Source data Fig. 5 [file 44321_2025_334_MOESM8_ESM.zip › Figure 5/5E/5E.jpg]

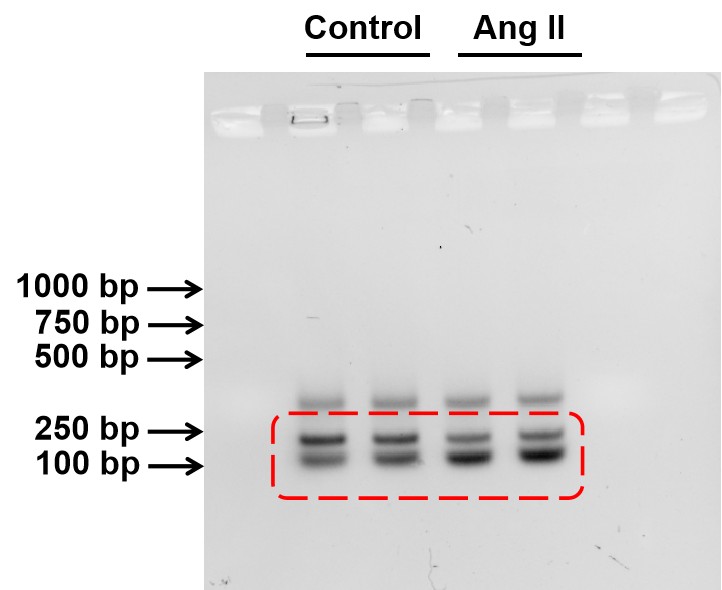

Supplement: Supplementary file 8 — Source data Fig. 5 [file 44321_2025_334_MOESM8_ESM.zip › Figure 5/5F/5F.jpg]

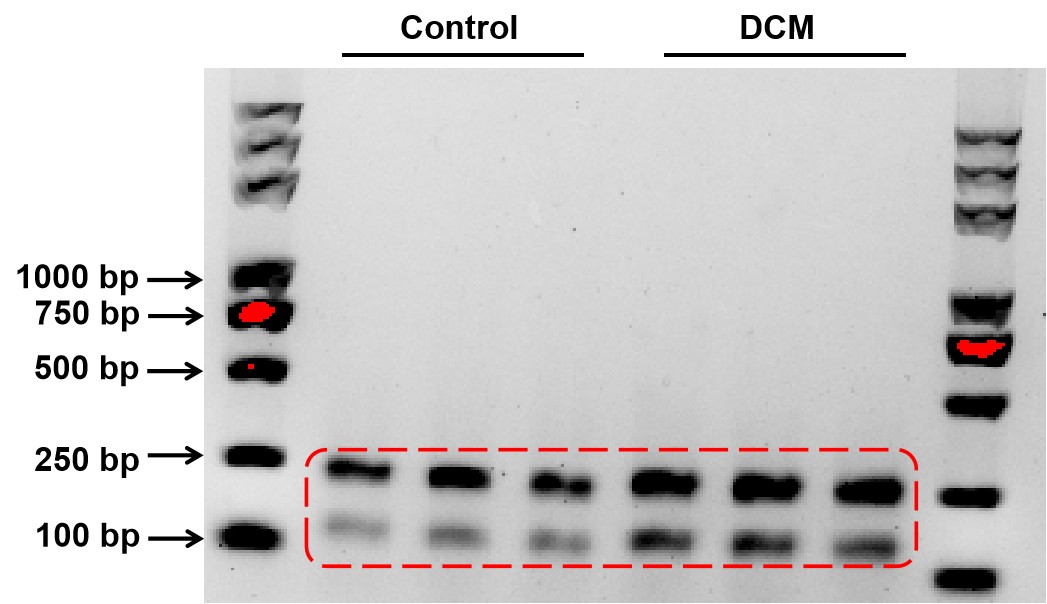

Supplement: Supplementary file 8 — Source data Fig. 5 [file 44321_2025_334_MOESM8_ESM.zip › Figure 5/5G/5G.jpg]

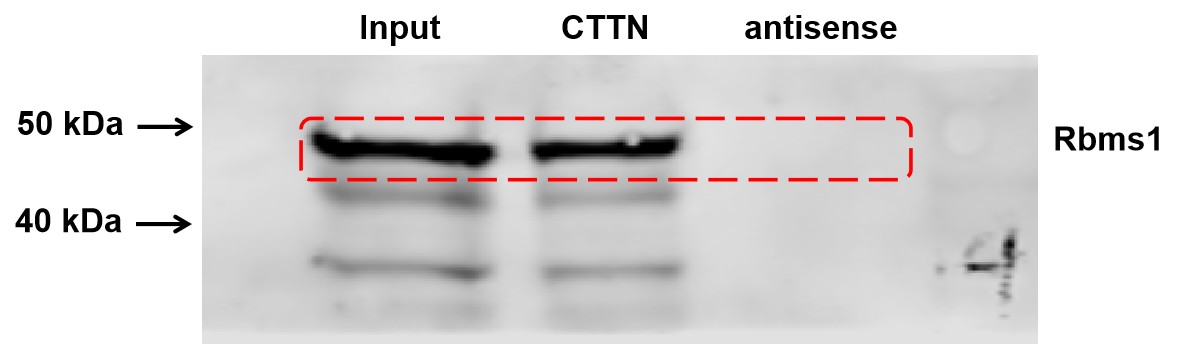

Supplement: Supplementary file 8 — Source data Fig. 5 [file 44321_2025_334_MOESM8_ESM.zip › Figure 5/5I/5I.jpg]

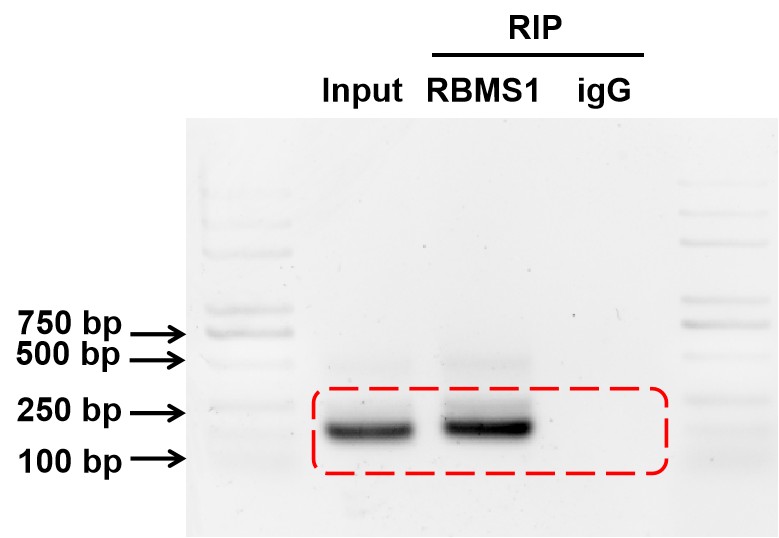

Supplement: Supplementary file 8 — Source data Fig. 5 [file 44321_2025_334_MOESM8_ESM.zip › Figure 5/5J/5J.jpg]

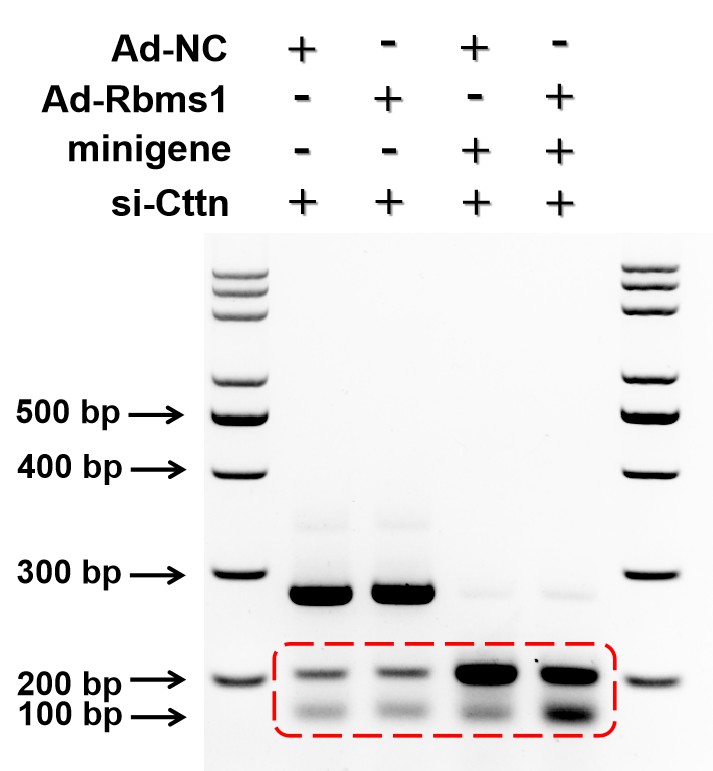

Supplement: Supplementary file 8 — Source data Fig. 5 [file 44321_2025_334_MOESM8_ESM.zip › Figure 5/5K/5K.jpg]

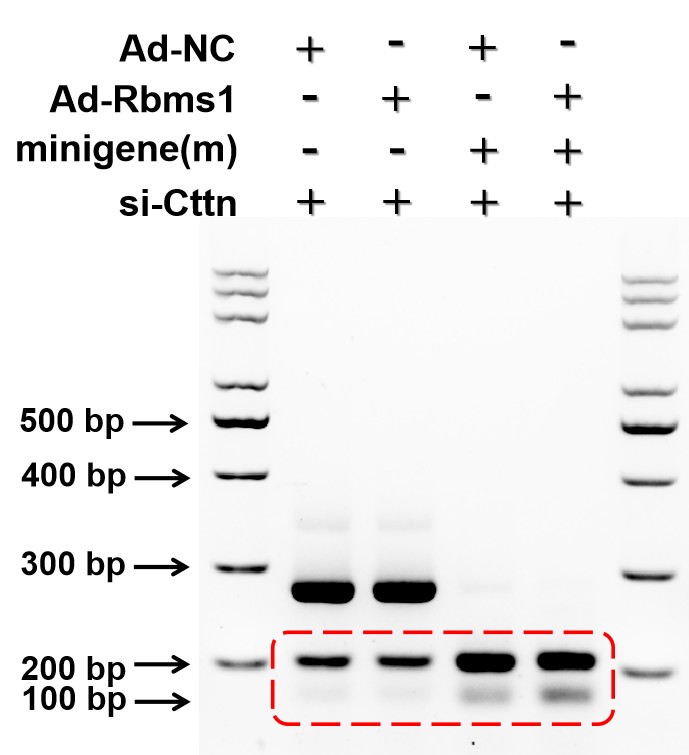

Supplement: Supplementary file 8 — Source data Fig. 5 [file 44321_2025_334_MOESM8_ESM.zip › Figure 5/5L/5L.jpg]

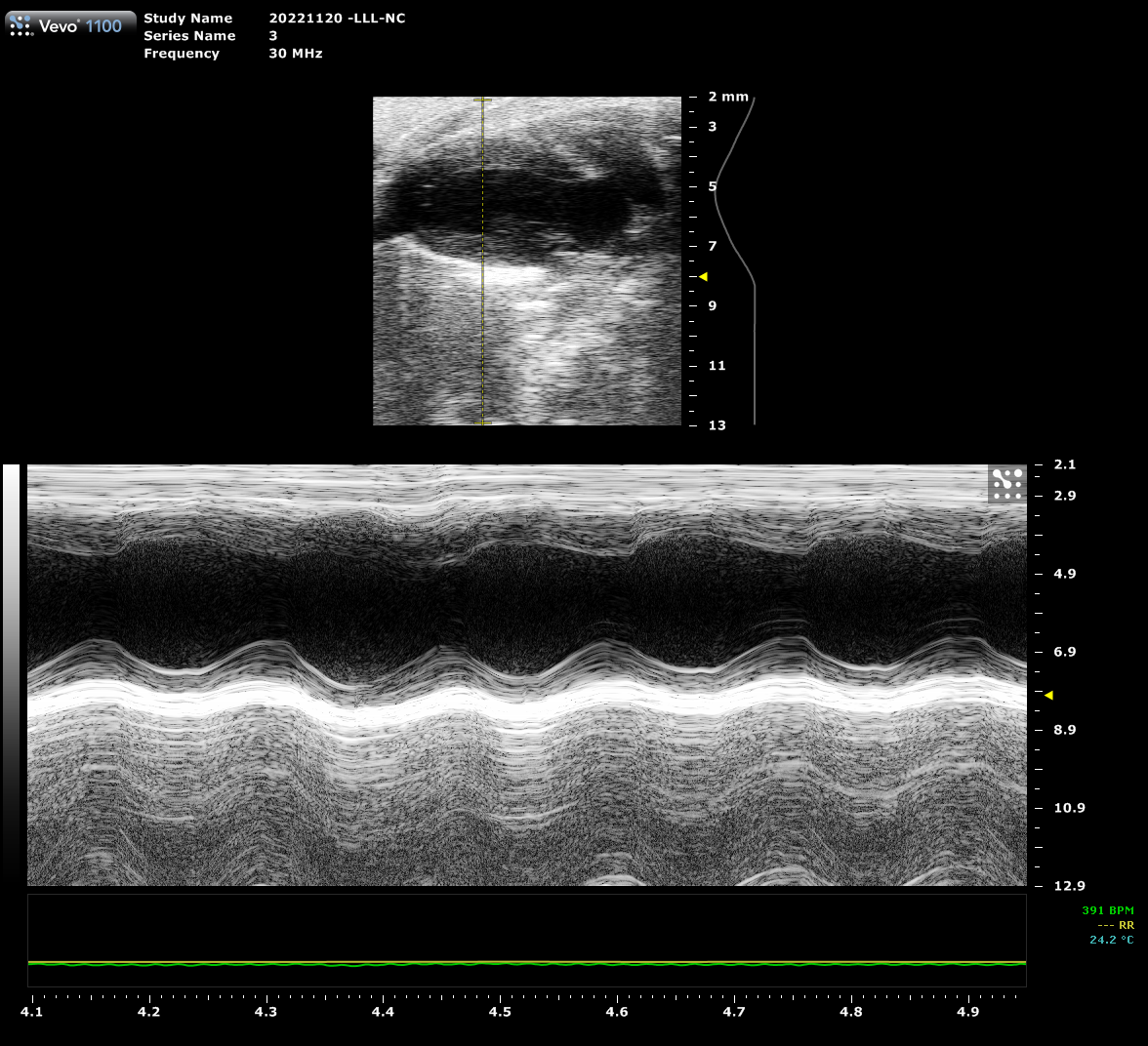

Supplement: Supplementary file 9 — Source data Fig. 6 [file 44321_2025_334_MOESM9_ESM.zip › Figure 6/6B/B Mode/sh-Vector+Sham.tif]

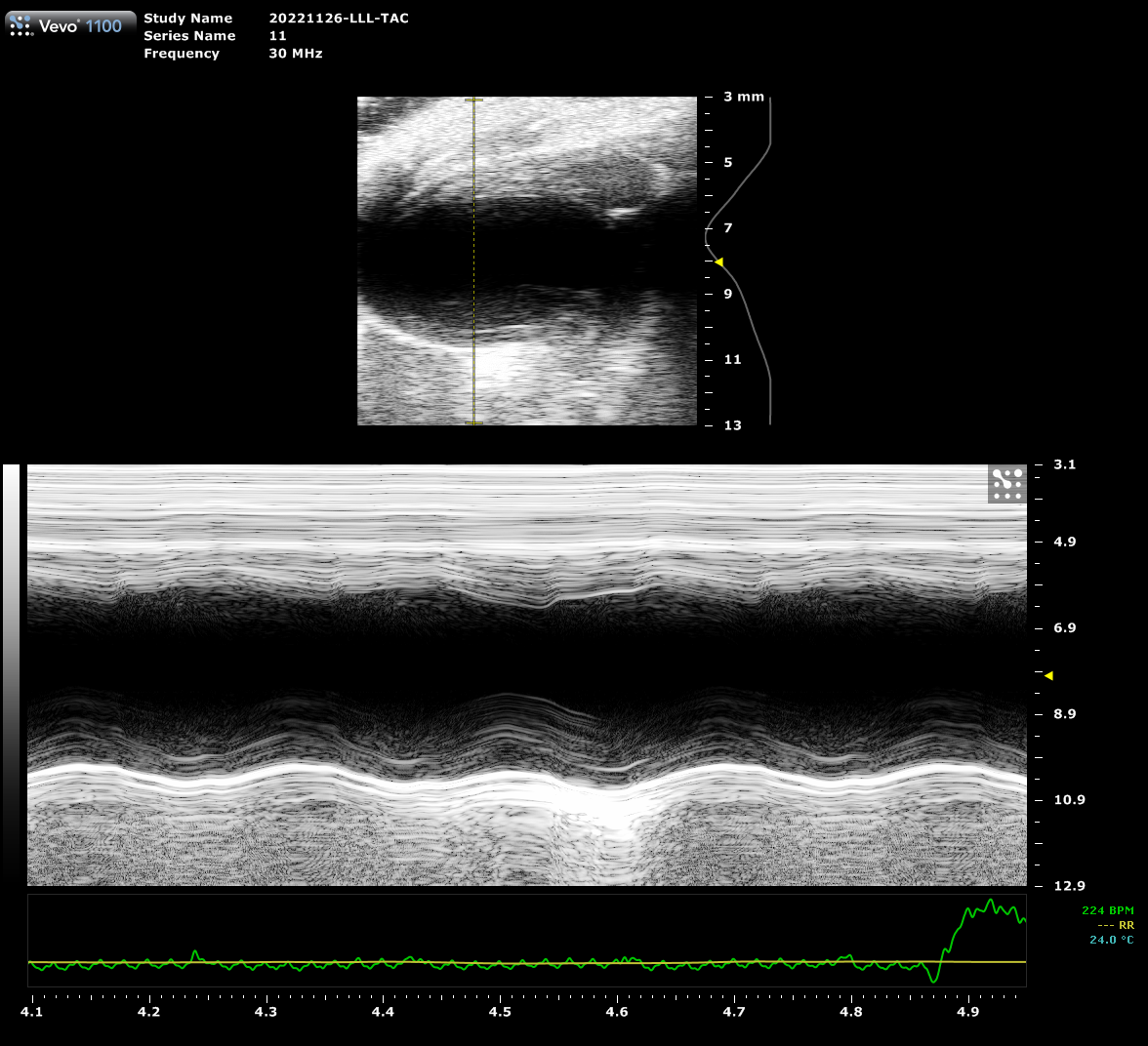

Supplement: Supplementary file 9 — Source data Fig. 6 [file 44321_2025_334_MOESM9_ESM.zip › Figure 6/6B/B Mode/sh-Vector+TAC.tif]

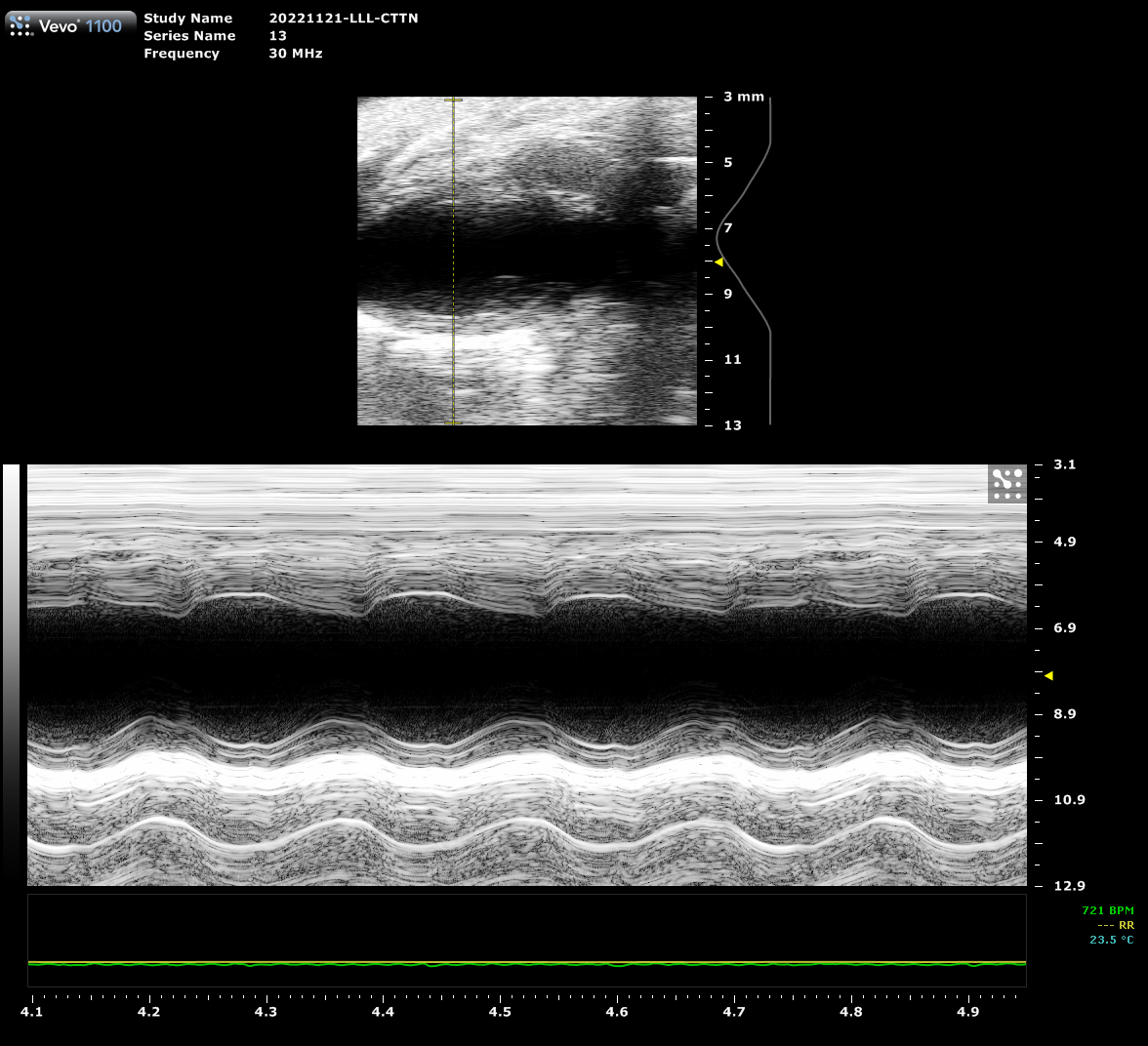

Supplement: Supplementary file 9 — Source data Fig. 6 [file 44321_2025_334_MOESM9_ESM.zip › Figure 6/6B/B Mode/sh-Δe11+Sham.tif]

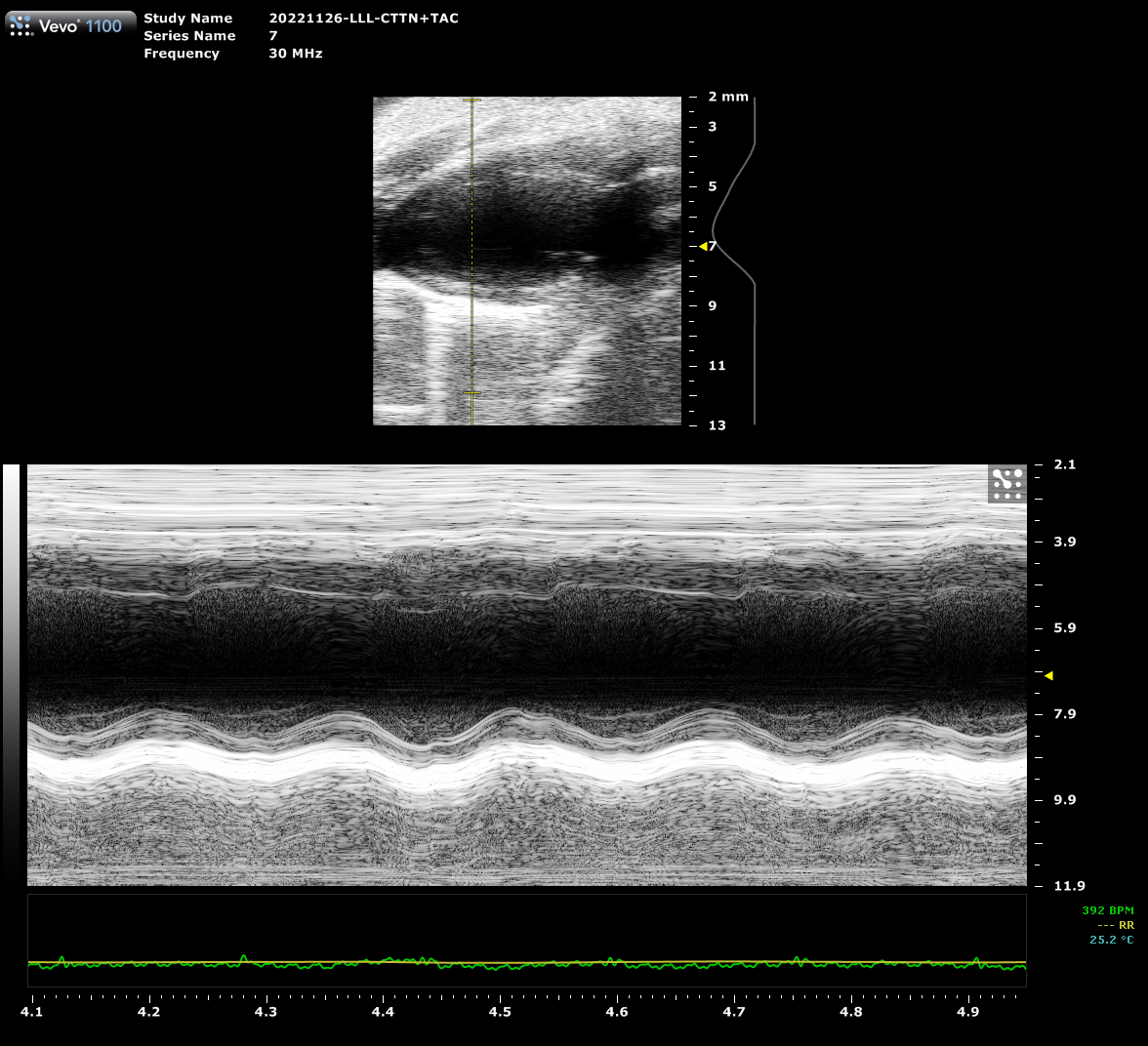

Supplement: Supplementary file 9 — Source data Fig. 6 [file 44321_2025_334_MOESM9_ESM.zip › Figure 6/6B/B Mode/sh-Δe11+TAC.tif]

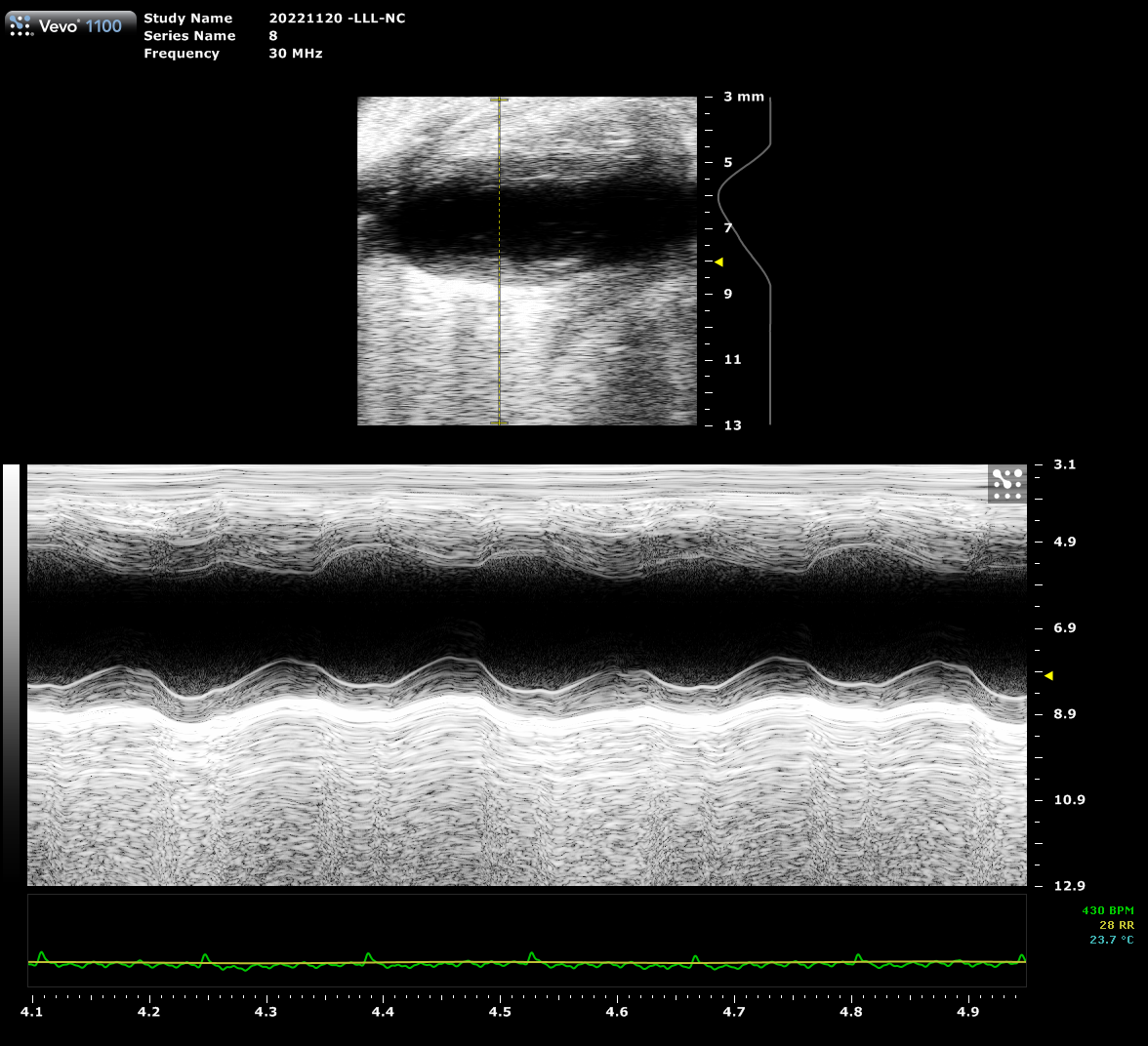

Supplement: Supplementary file 9 — Source data Fig. 6 [file 44321_2025_334_MOESM9_ESM.zip › Figure 6/6B/M Mode/sh-Vector+Sham.tif]

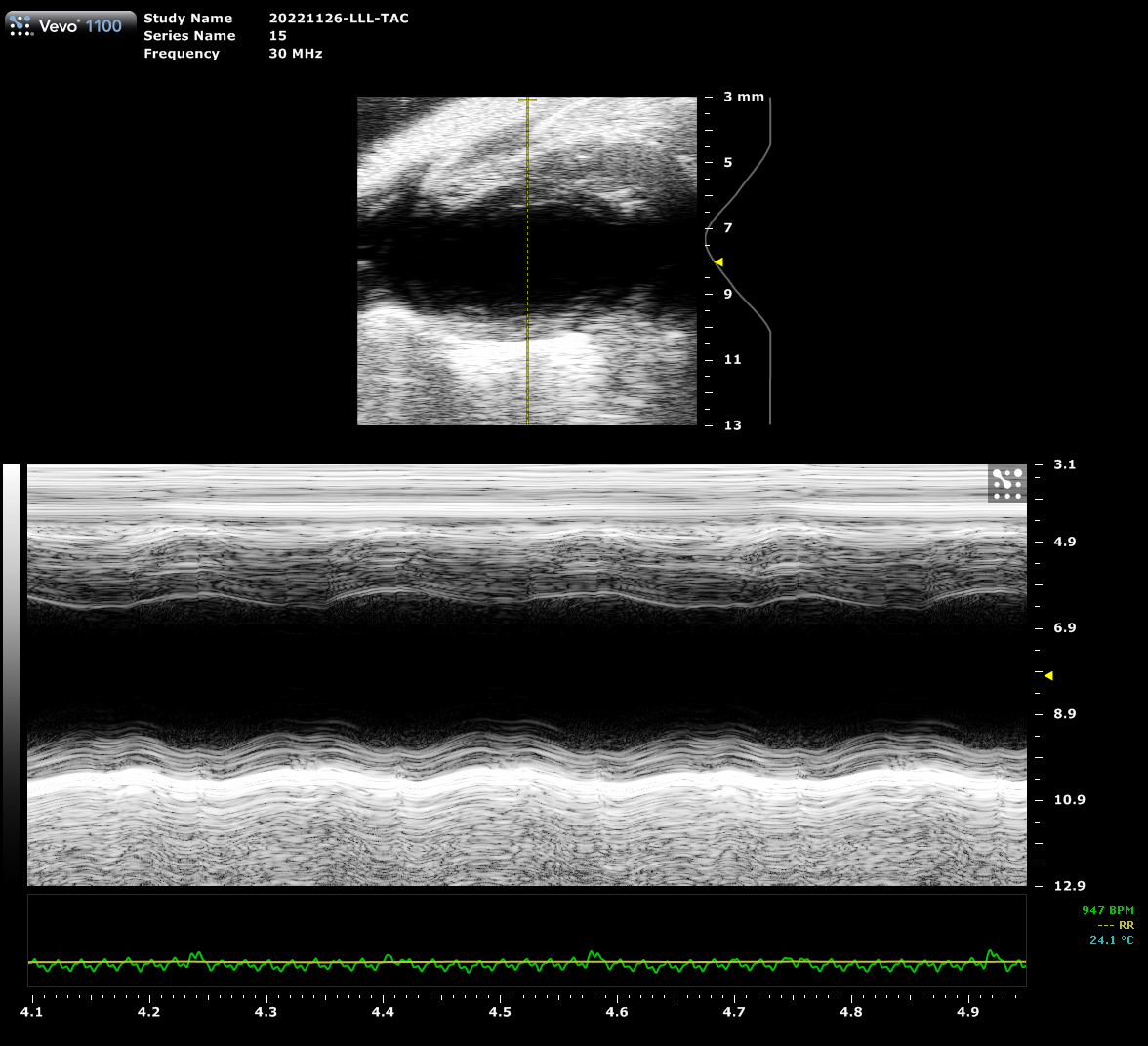

Supplement: Supplementary file 9 — Source data Fig. 6 [file 44321_2025_334_MOESM9_ESM.zip › Figure 6/6B/M Mode/sh-Vector+TAC.tif]

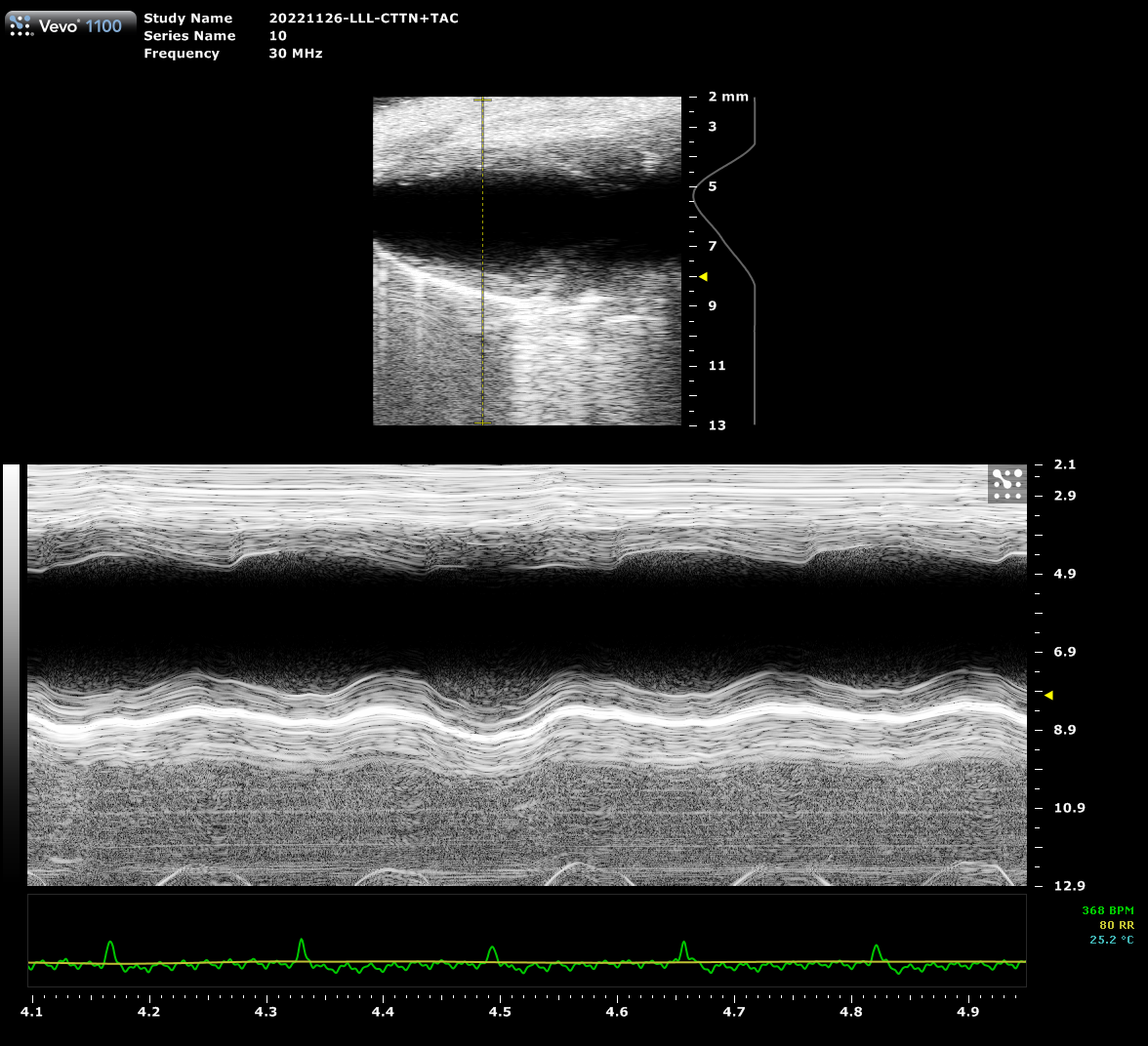

Supplement: Supplementary file 9 — Source data Fig. 6 [file 44321_2025_334_MOESM9_ESM.zip › Figure 6/6B/M Mode/sh-Δe11+TAC.tif]

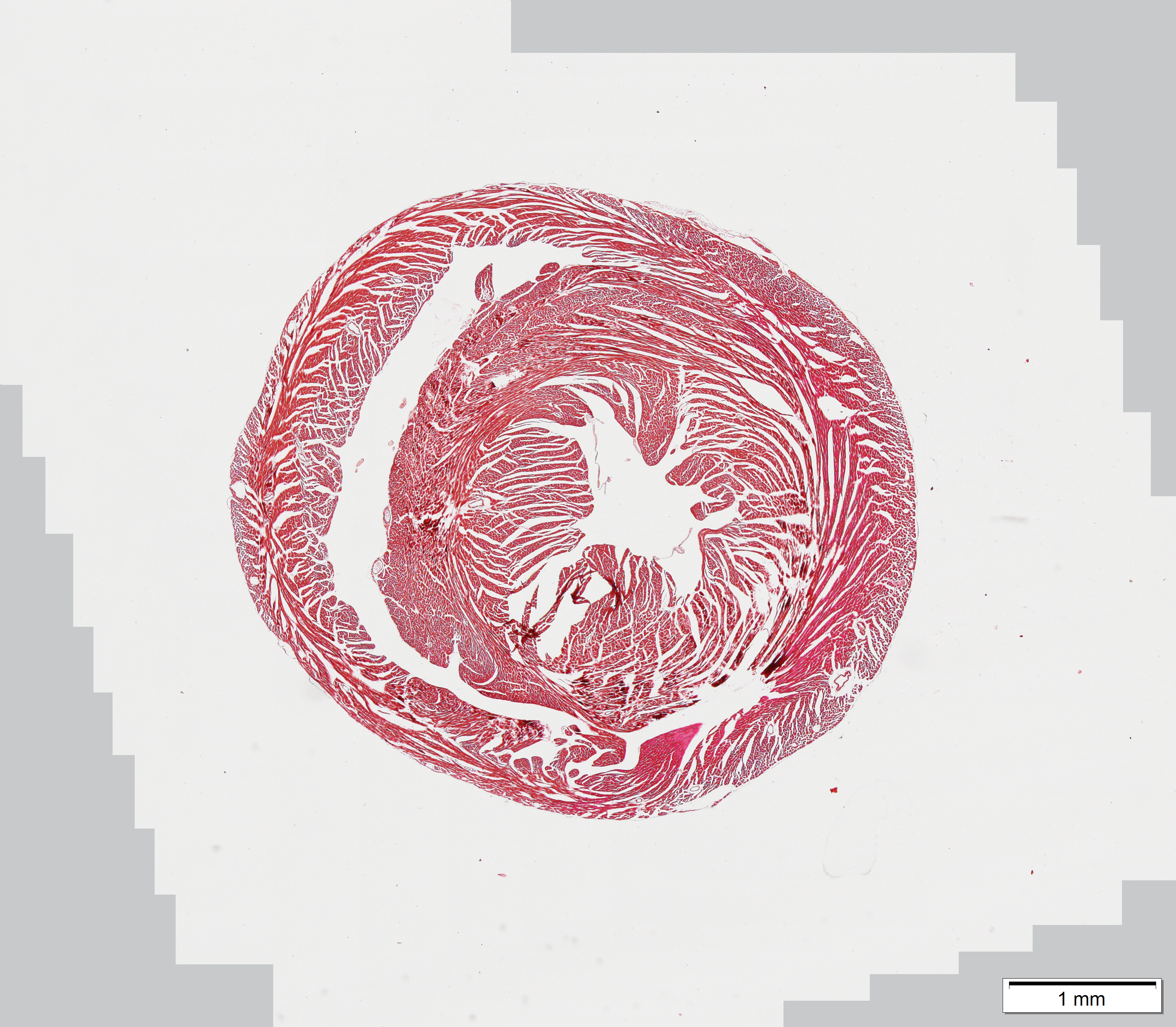

Supplement: Supplementary file 9 — Source data Fig. 6 [file 44321_2025_334_MOESM9_ESM.zip › Figure 6/6D/Cross/sh-Vector+Sham.tif]

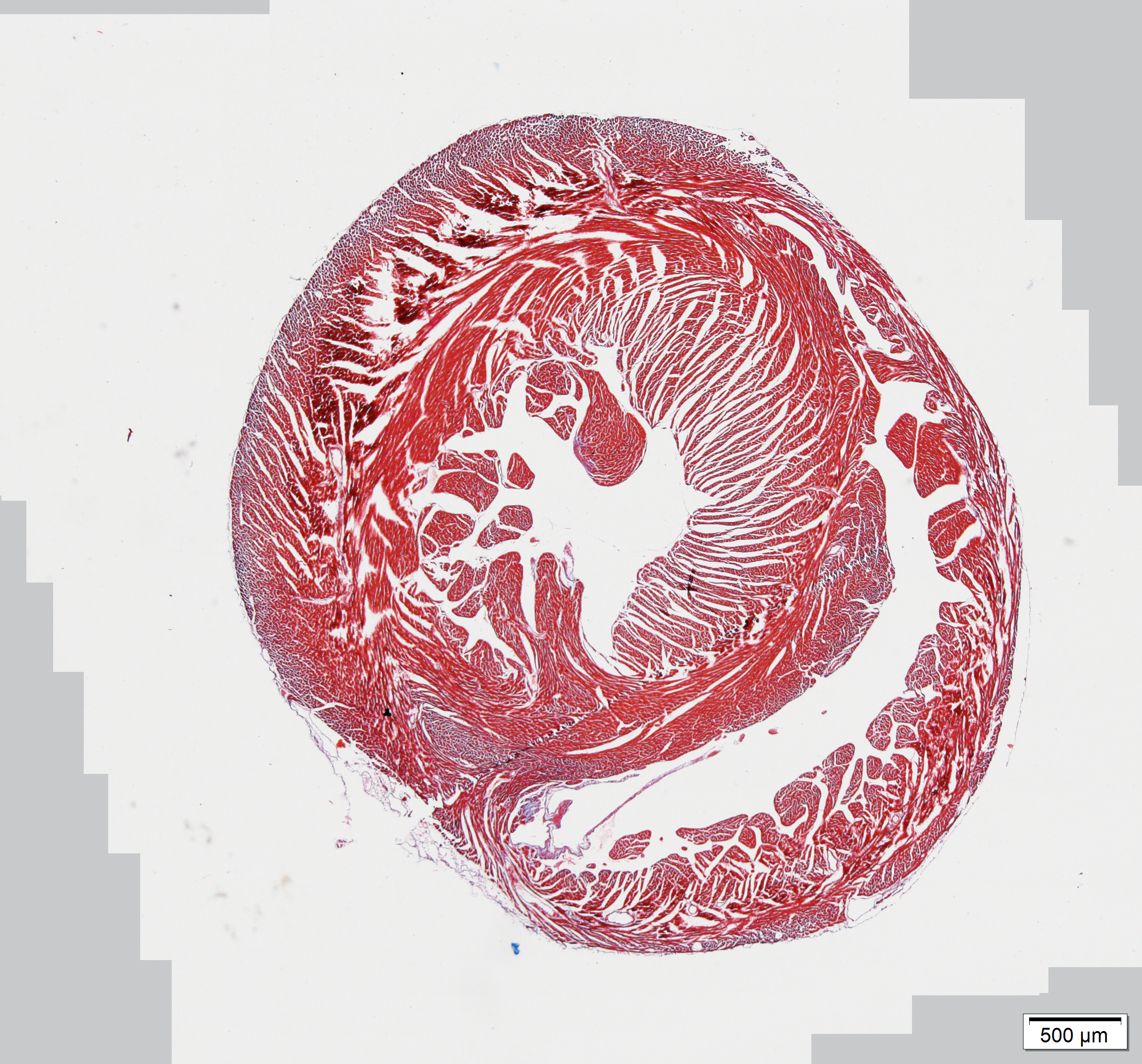

Supplement: Supplementary file 9 — Source data Fig. 6 [file 44321_2025_334_MOESM9_ESM.zip › Figure 6/6D/Cross/sh-Vector+TAC.tif]

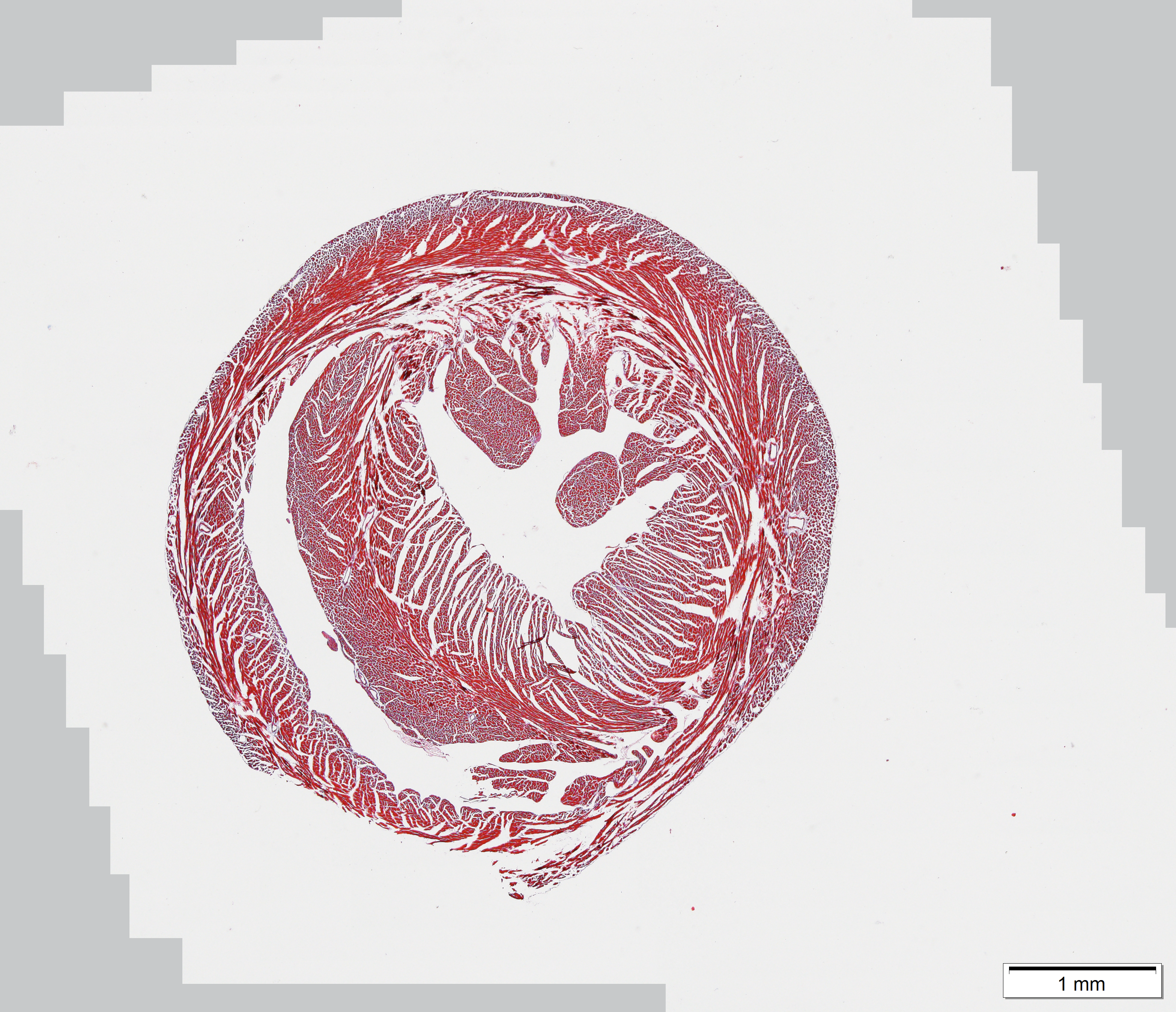

Supplement: Supplementary file 9 — Source data Fig. 6 [file 44321_2025_334_MOESM9_ESM.zip › Figure 6/6D/Cross/sh-Δe11+Sham.tif]

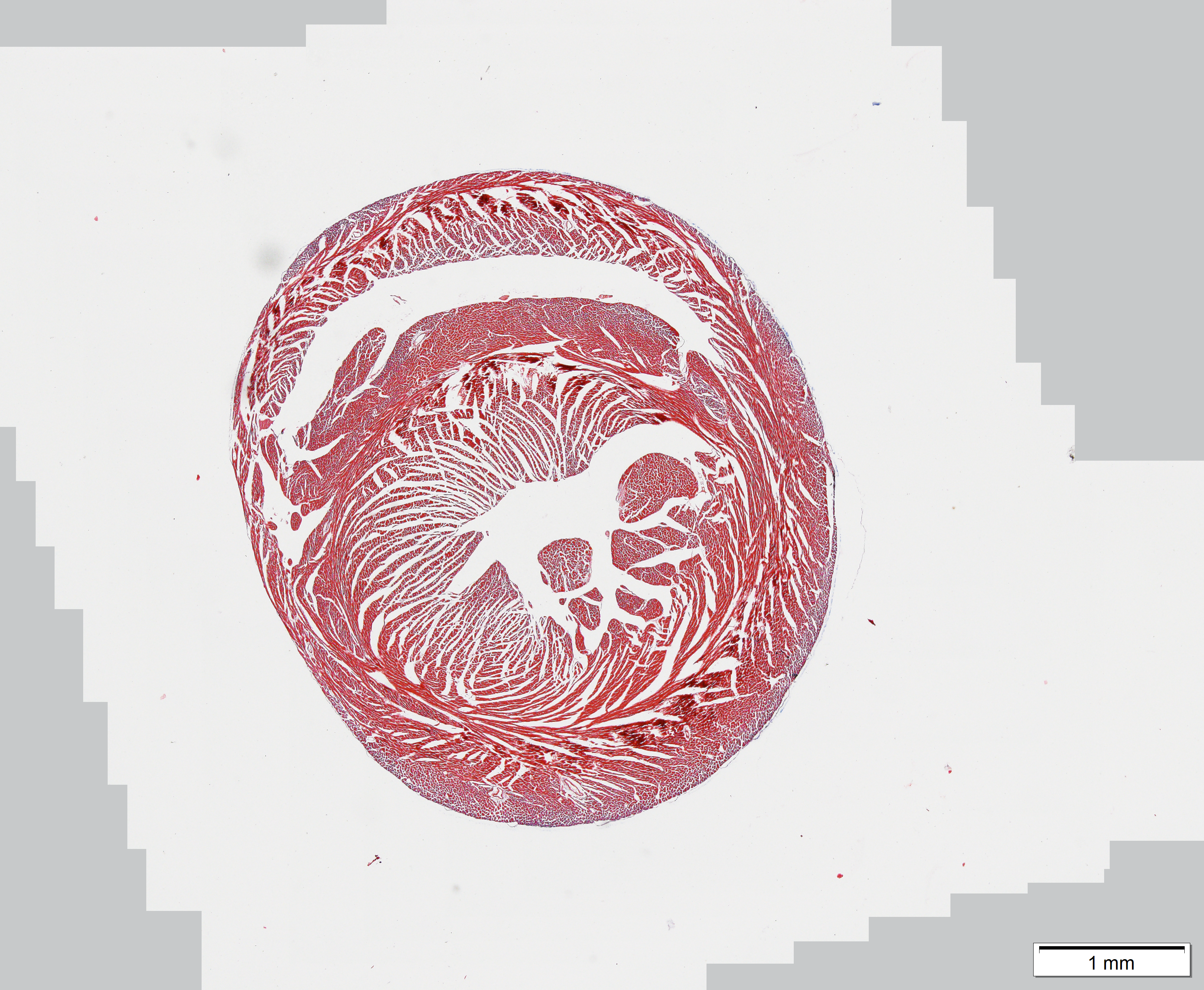

Supplement: Supplementary file 9 — Source data Fig. 6 [file 44321_2025_334_MOESM9_ESM.zip › Figure 6/6D/Cross/sh-Δe11+TAC.tif]

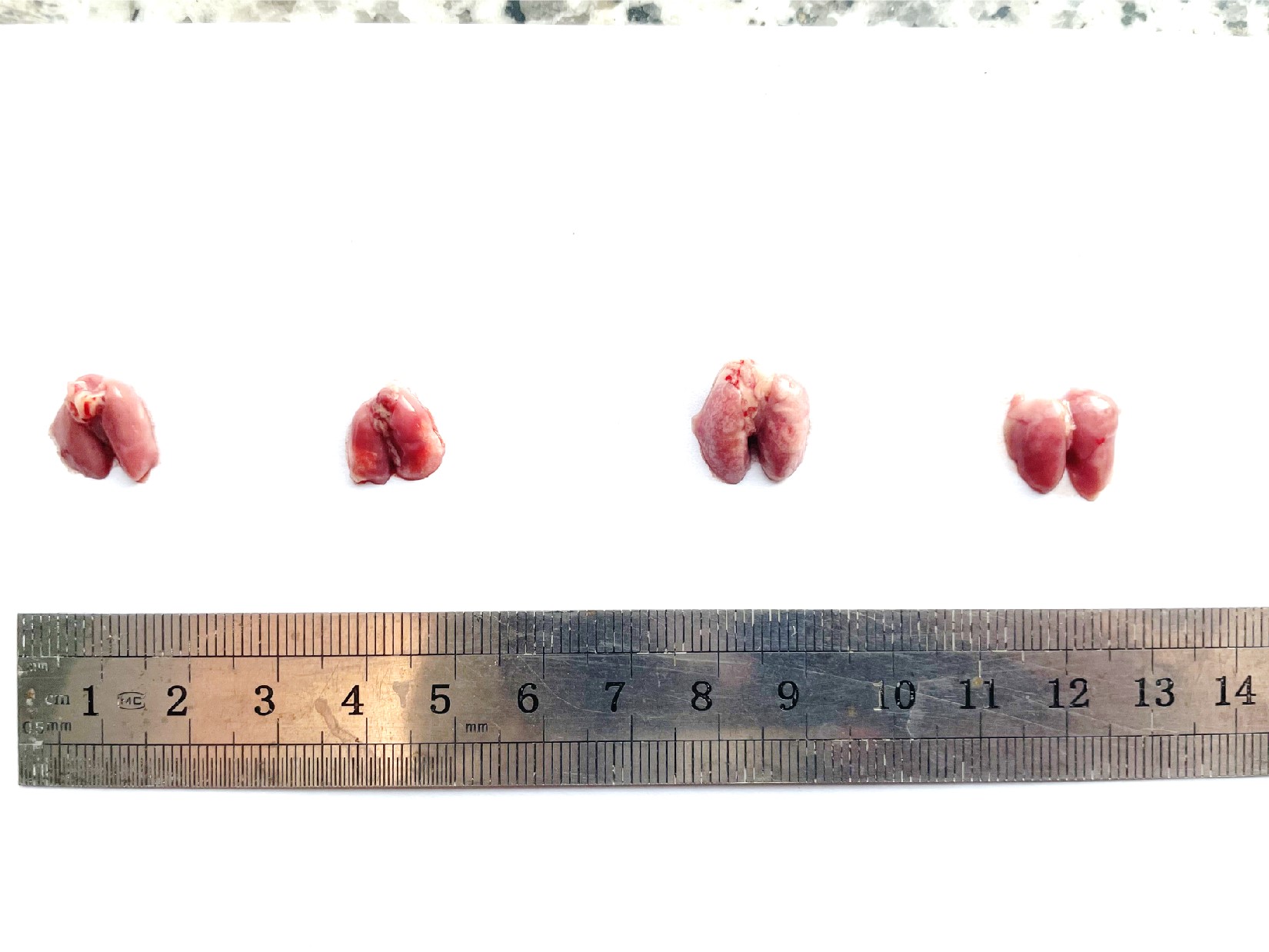

Supplement: Supplementary file 9 — Source data Fig. 6 [file 44321_2025_334_MOESM9_ESM.zip › Figure 6/6D/Heart size/6D-1.jpg]

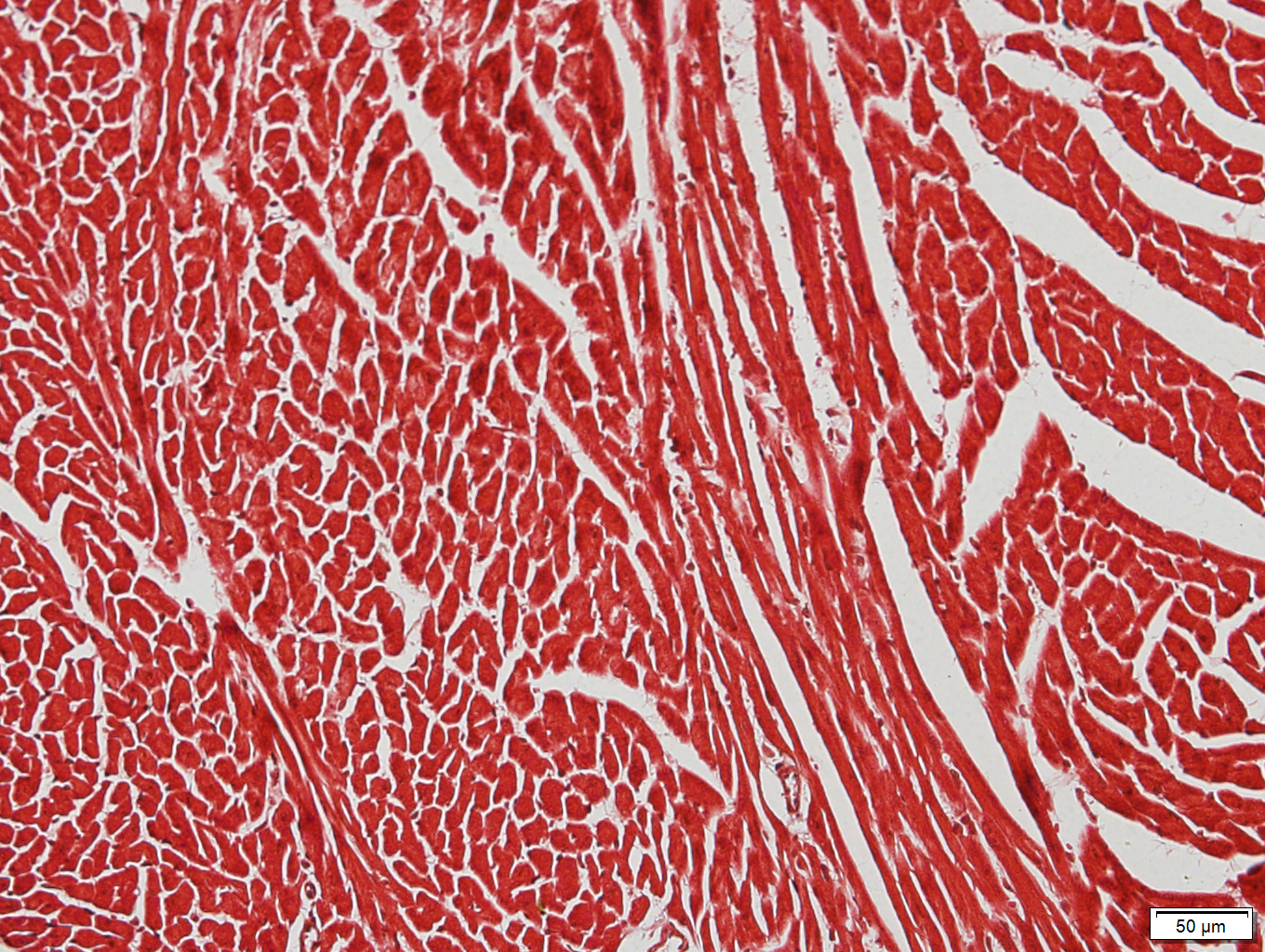

Supplement: Supplementary file 9 — Source data Fig. 6 [file 44321_2025_334_MOESM9_ESM.zip › Figure 6/6D/Interstital/sh-Vector+Sham.tif]

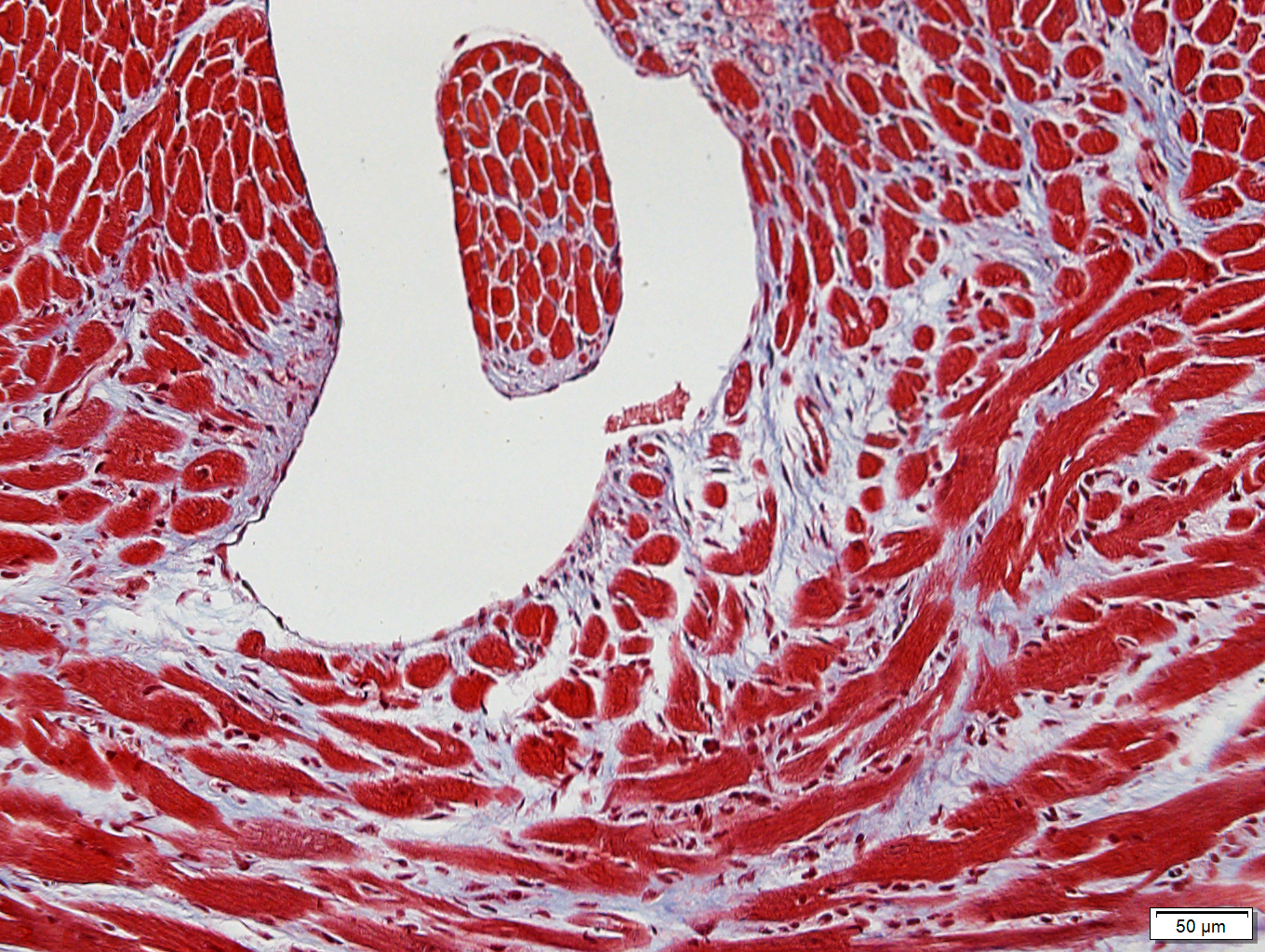

Supplement: Supplementary file 9 — Source data Fig. 6 [file 44321_2025_334_MOESM9_ESM.zip › Figure 6/6D/Interstital/sh-Vector+TAC.tif]

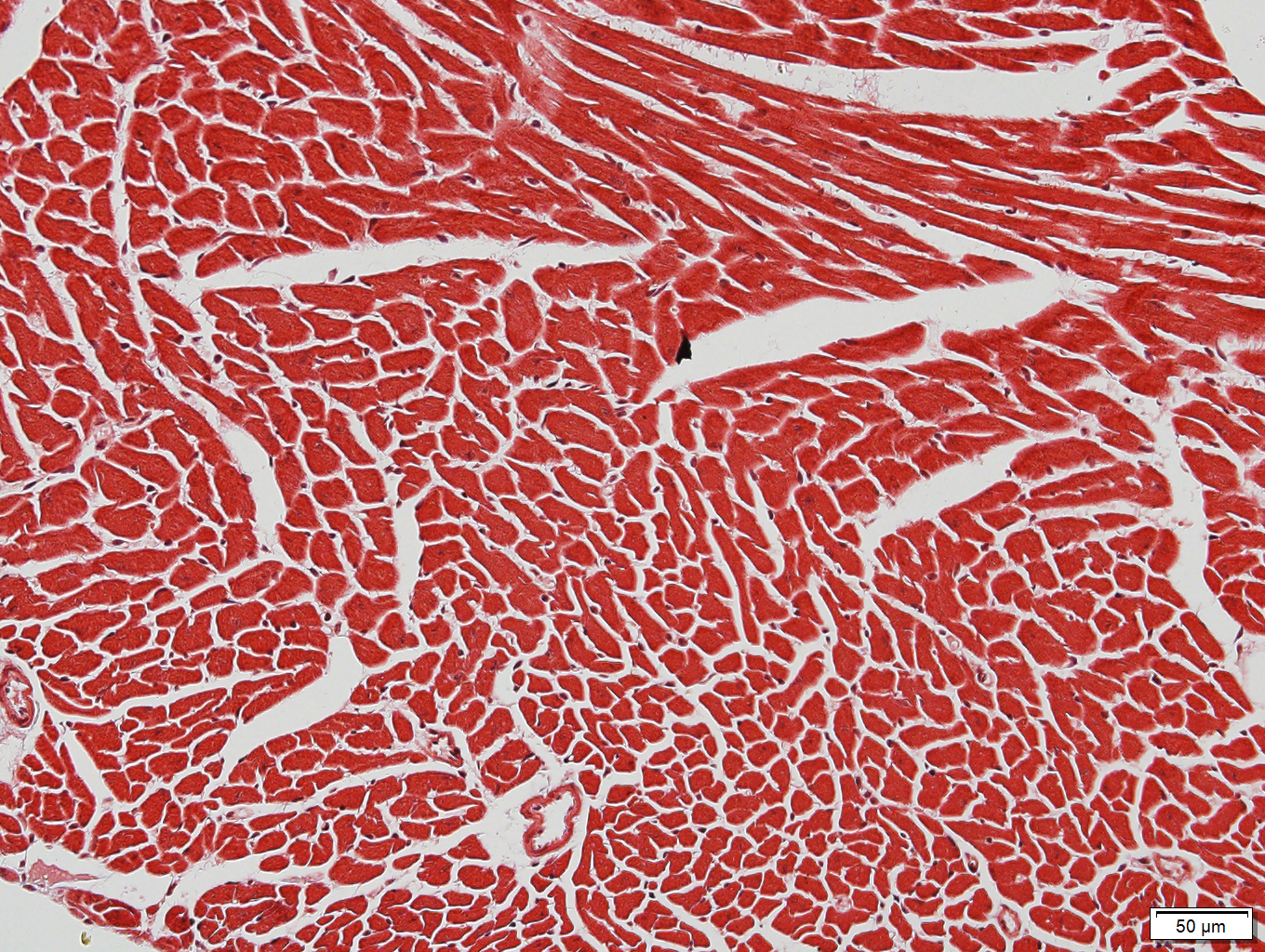

Supplement: Supplementary file 9 — Source data Fig. 6 [file 44321_2025_334_MOESM9_ESM.zip › Figure 6/6D/Interstital/sh-Δe11+Sham.tif]

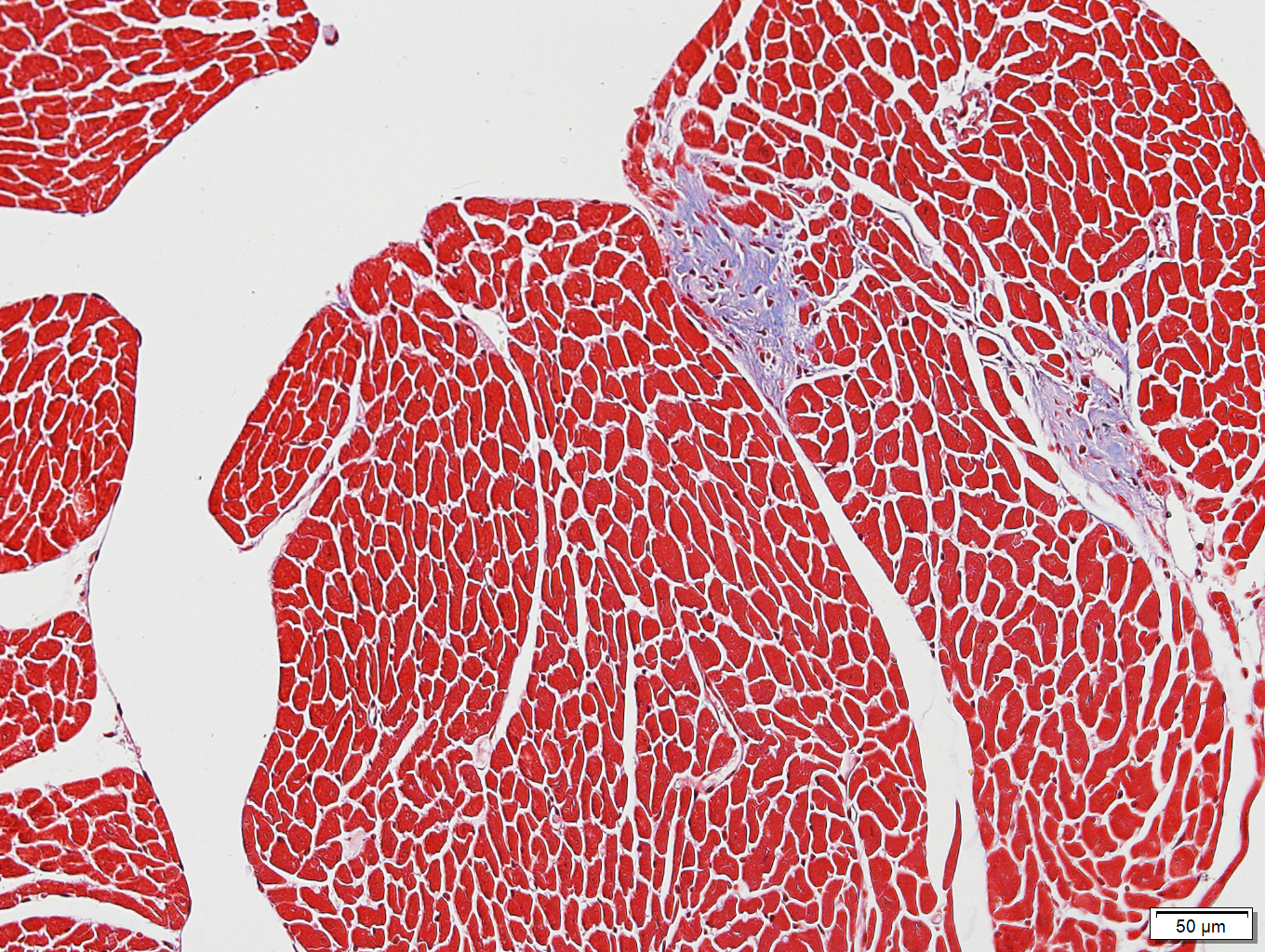

Supplement: Supplementary file 9 — Source data Fig. 6 [file 44321_2025_334_MOESM9_ESM.zip › Figure 6/6D/Interstital/sh-Δe11+TAC.tif]

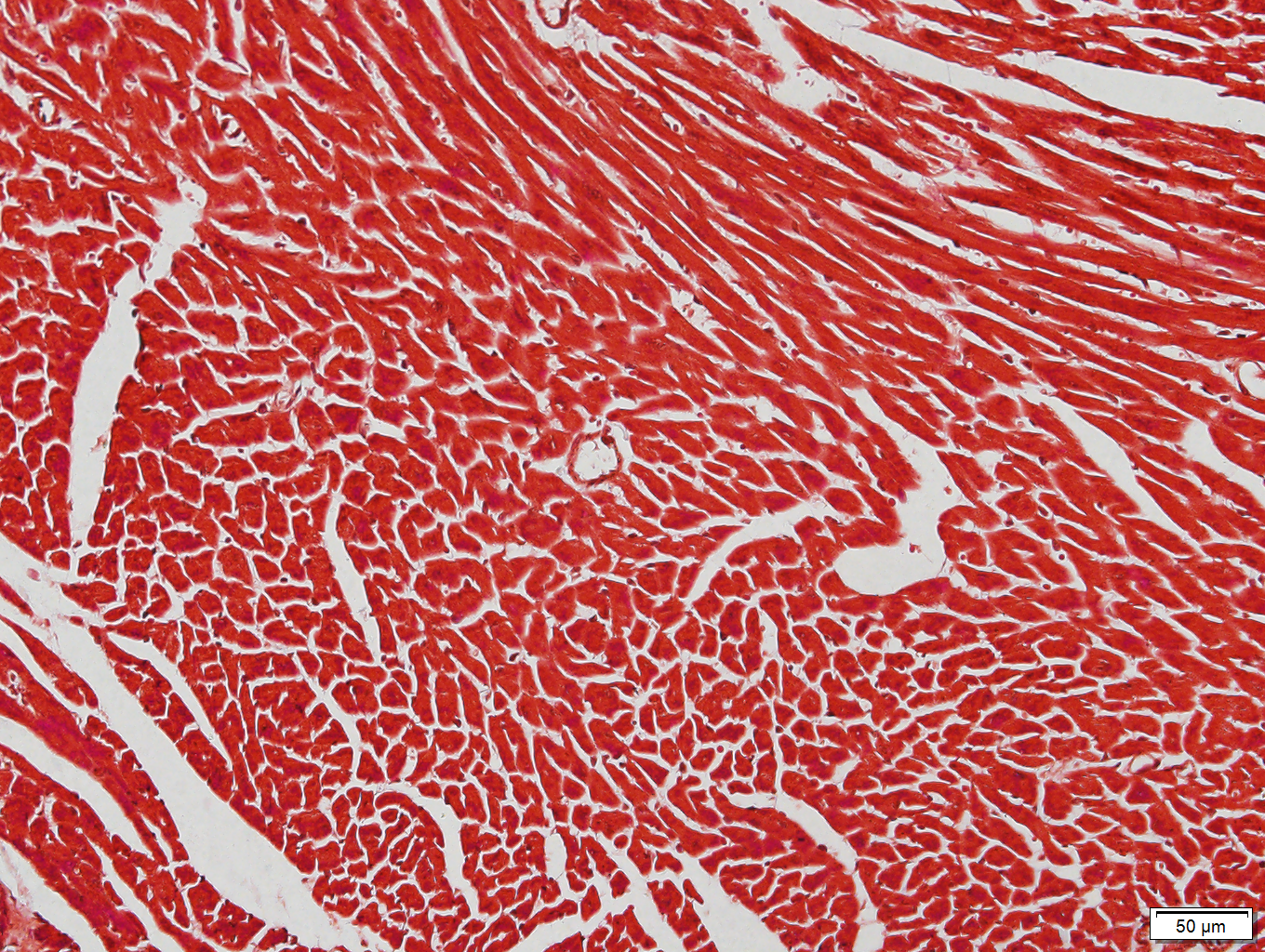

Supplement: Supplementary file 9 — Source data Fig. 6 [file 44321_2025_334_MOESM9_ESM.zip › Figure 6/6D/Perivascular/sh-Vector+Sham.tif]

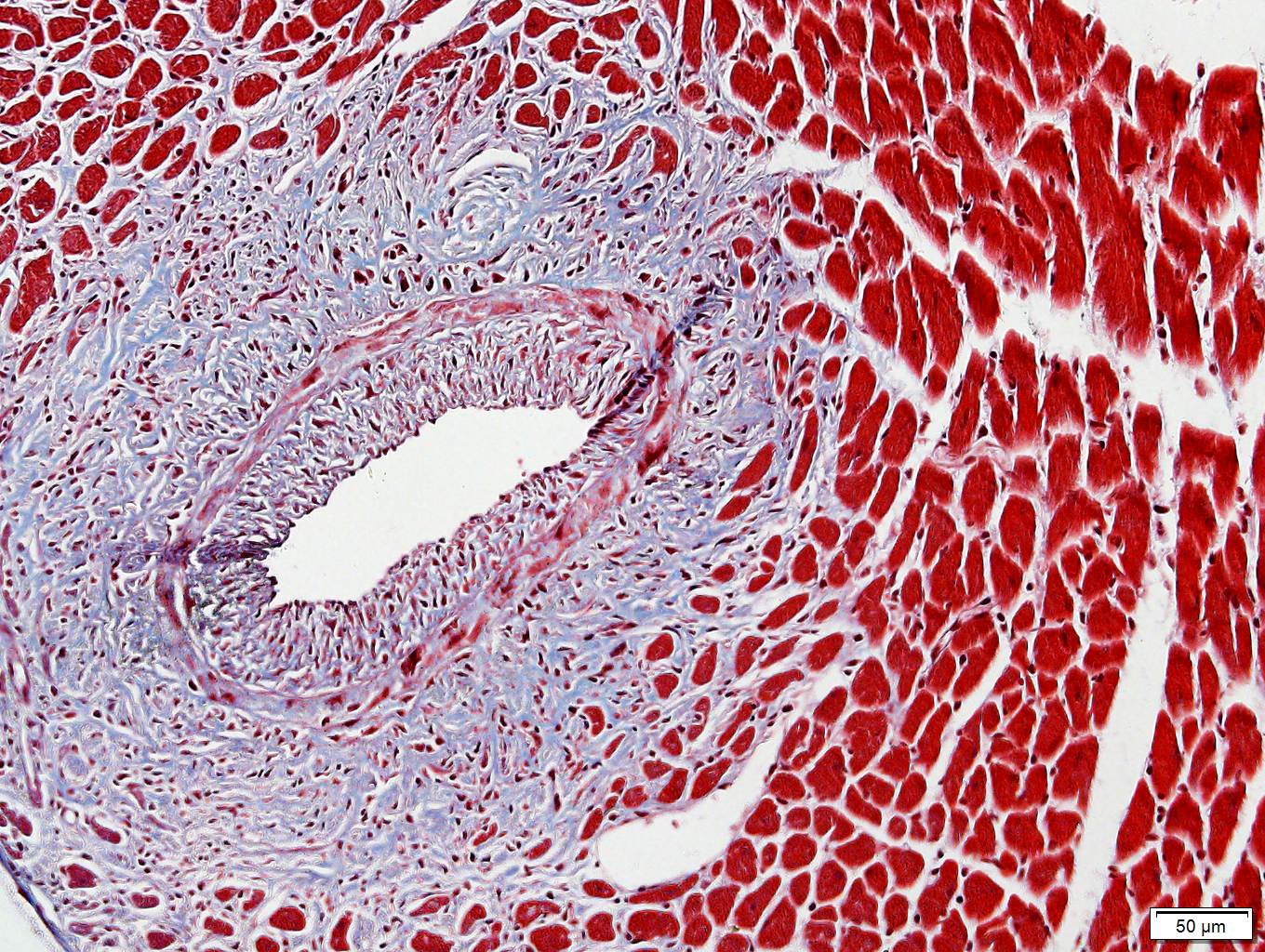

Supplement: Supplementary file 9 — Source data Fig. 6 [file 44321_2025_334_MOESM9_ESM.zip › Figure 6/6D/Perivascular/sh-Vector+TAC.tif]

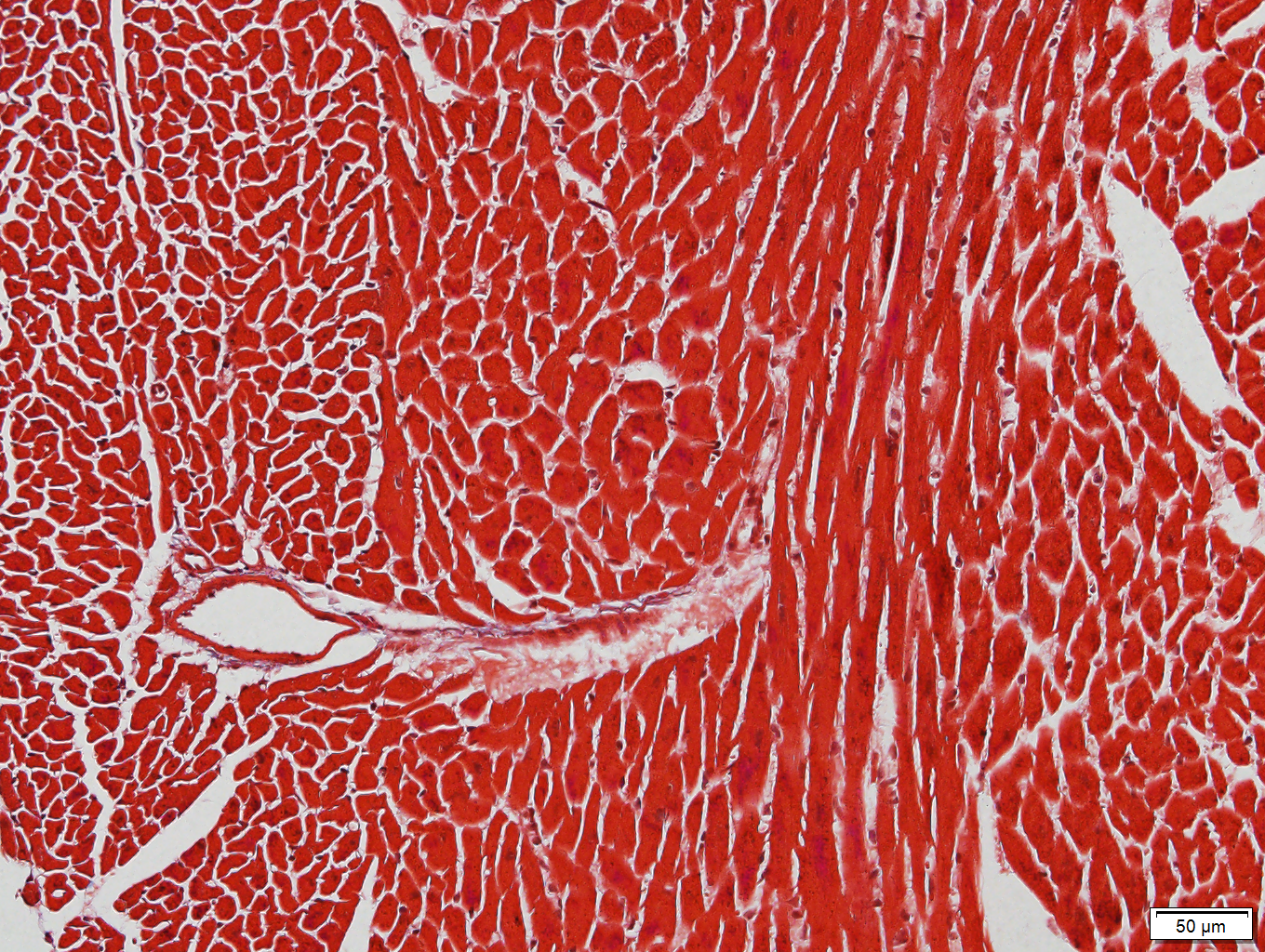

Supplement: Supplementary file 9 — Source data Fig. 6 [file 44321_2025_334_MOESM9_ESM.zip › Figure 6/6D/Perivascular/sh-Δe11+Sham.tif]

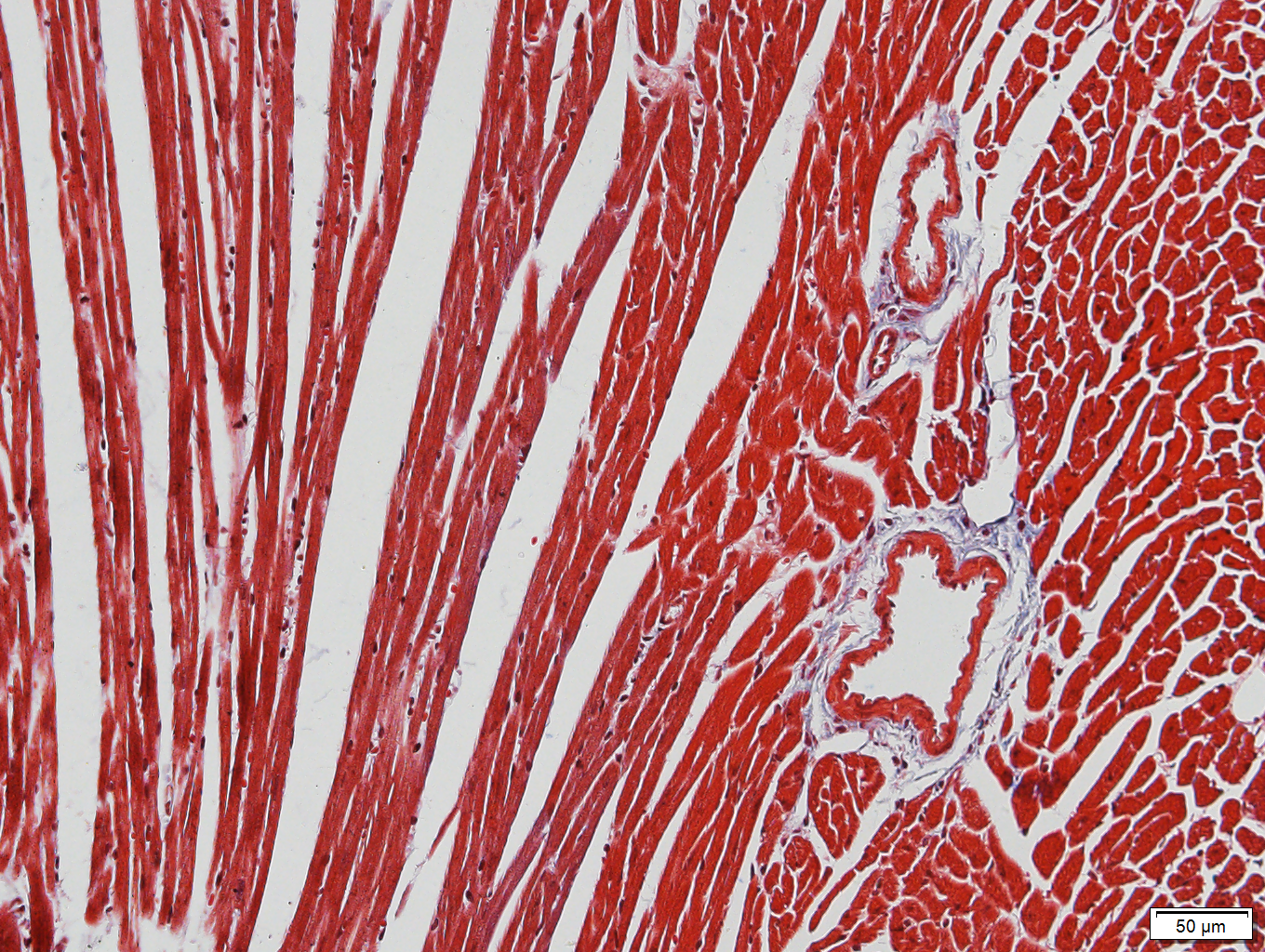

Supplement: Supplementary file 9 — Source data Fig. 6 [file 44321_2025_334_MOESM9_ESM.zip › Figure 6/6D/Perivascular/sh-Δe11+TAC.tif]

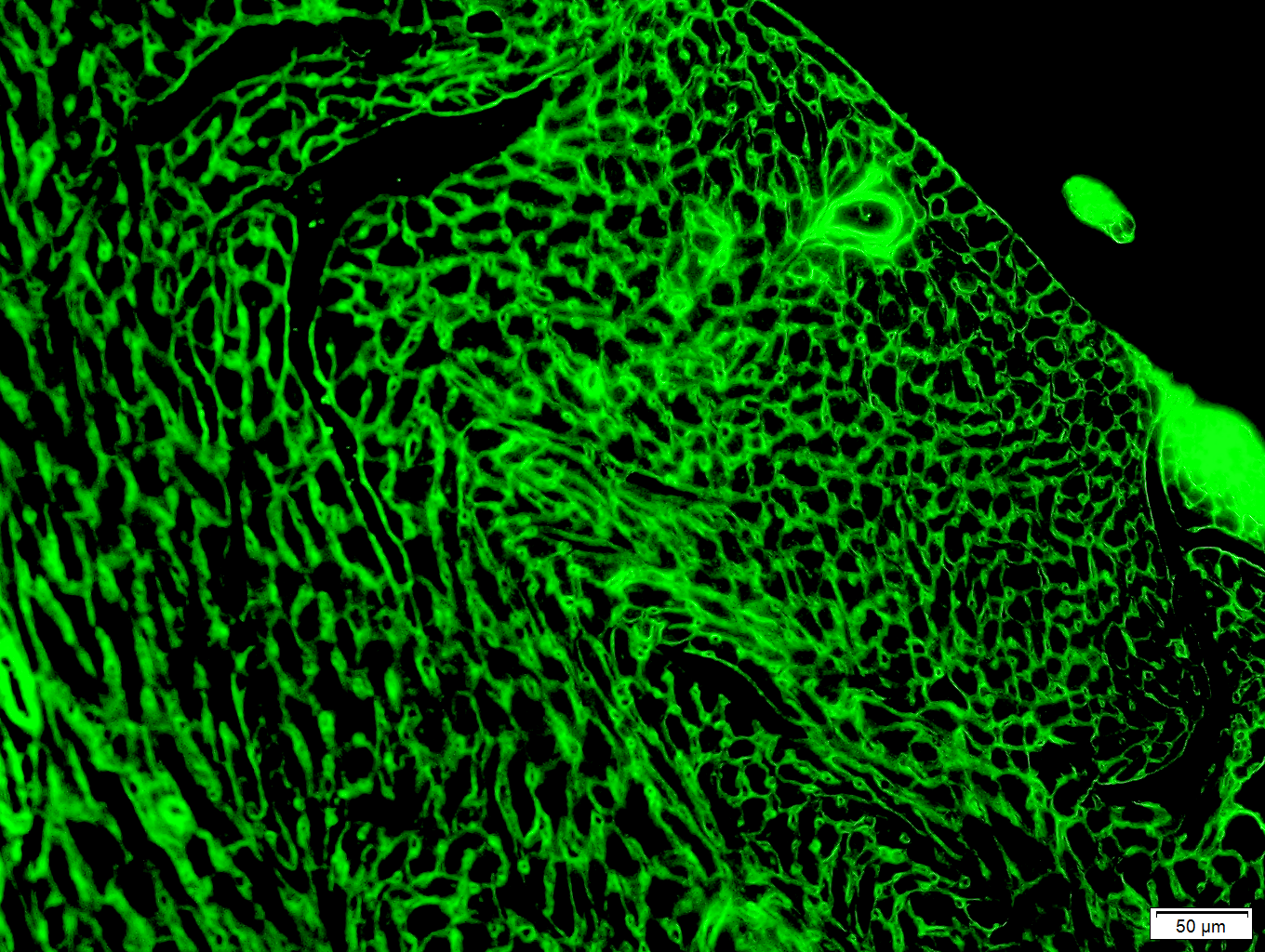

Supplement: Supplementary file 9 — Source data Fig. 6 [file 44321_2025_334_MOESM9_ESM.zip › Figure 6/6D/WGA/sh-Vector+Sham.tif]

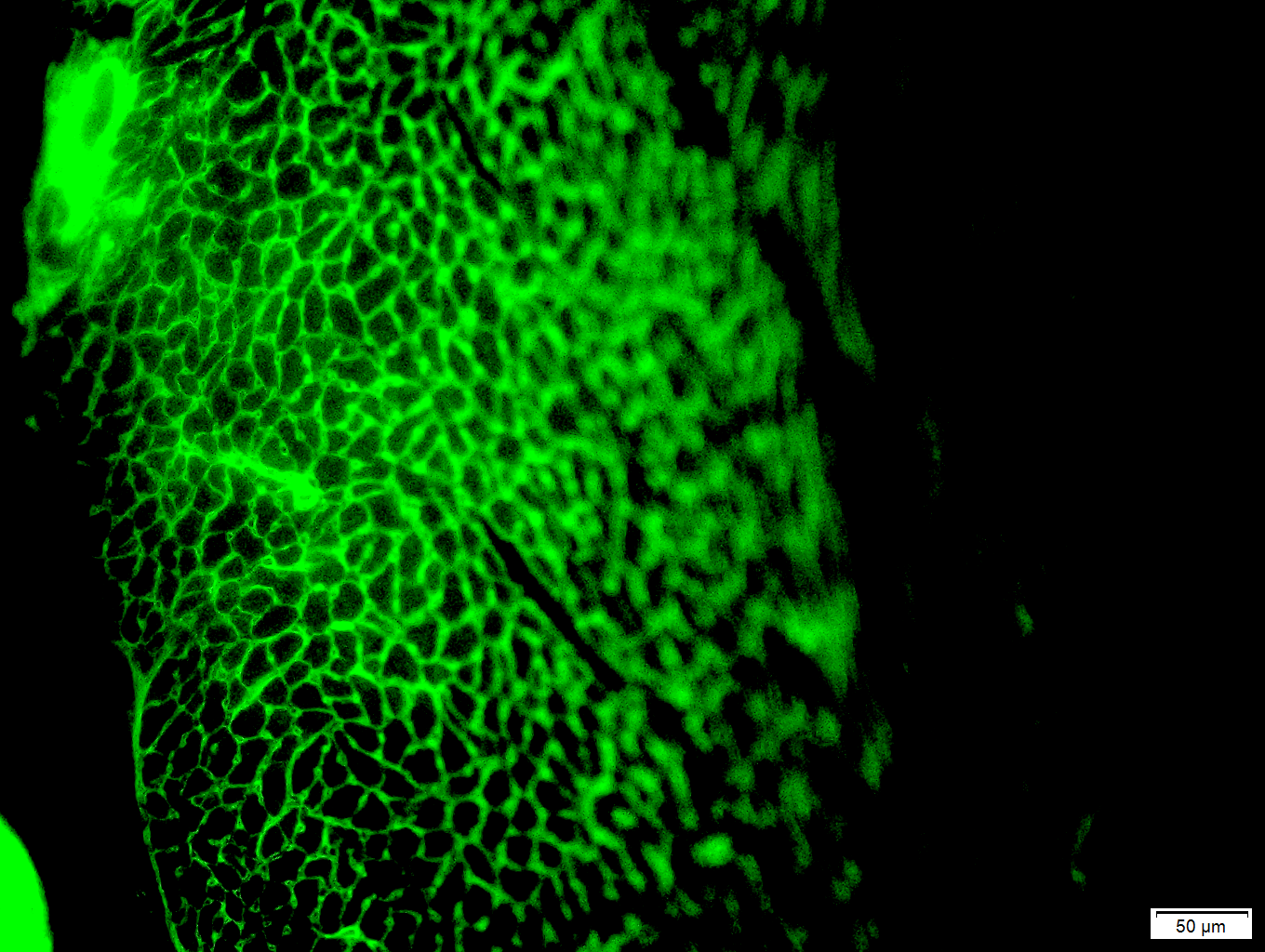

Supplement: Supplementary file 9 — Source data Fig. 6 [file 44321_2025_334_MOESM9_ESM.zip › Figure 6/6D/WGA/sh-Vector+TAC.tif]

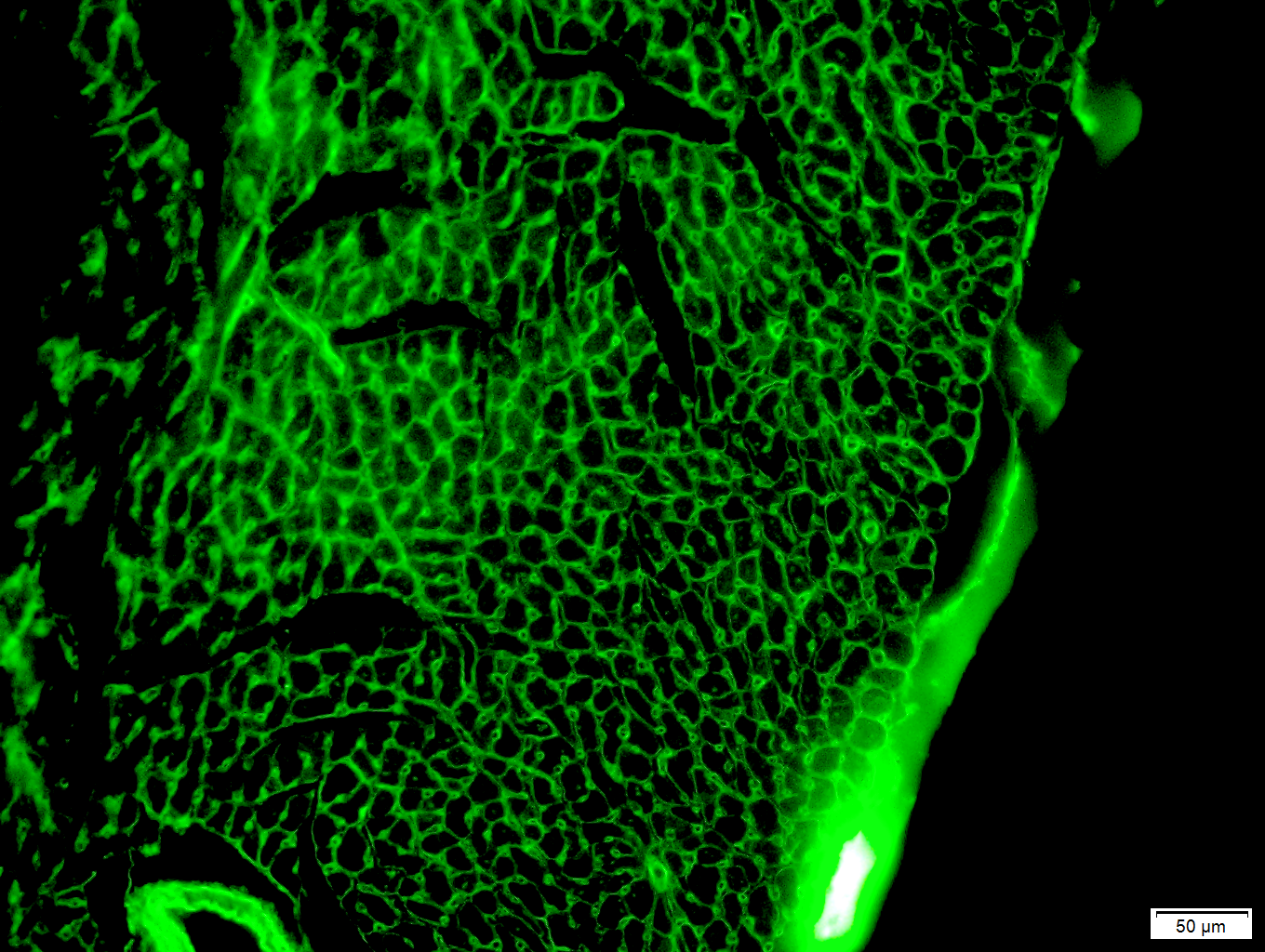

Supplement: Supplementary file 9 — Source data Fig. 6 [file 44321_2025_334_MOESM9_ESM.zip › Figure 6/6D/WGA/sh-Δe11+Sham.tif]

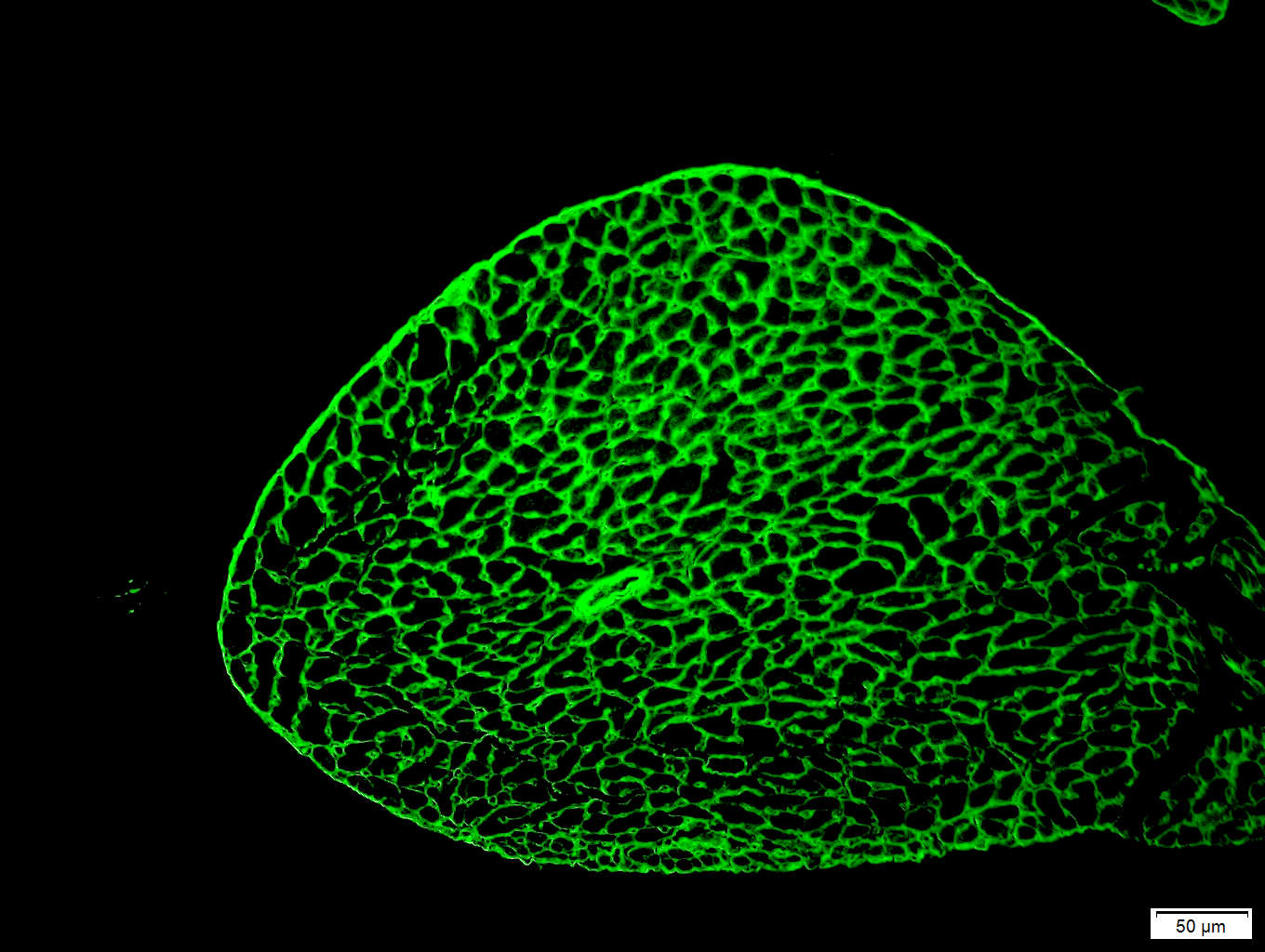

Supplement: Supplementary file 9 — Source data Fig. 6 [file 44321_2025_334_MOESM9_ESM.zip › Figure 6/6D/WGA/sh-Δe11+TAC.tif]

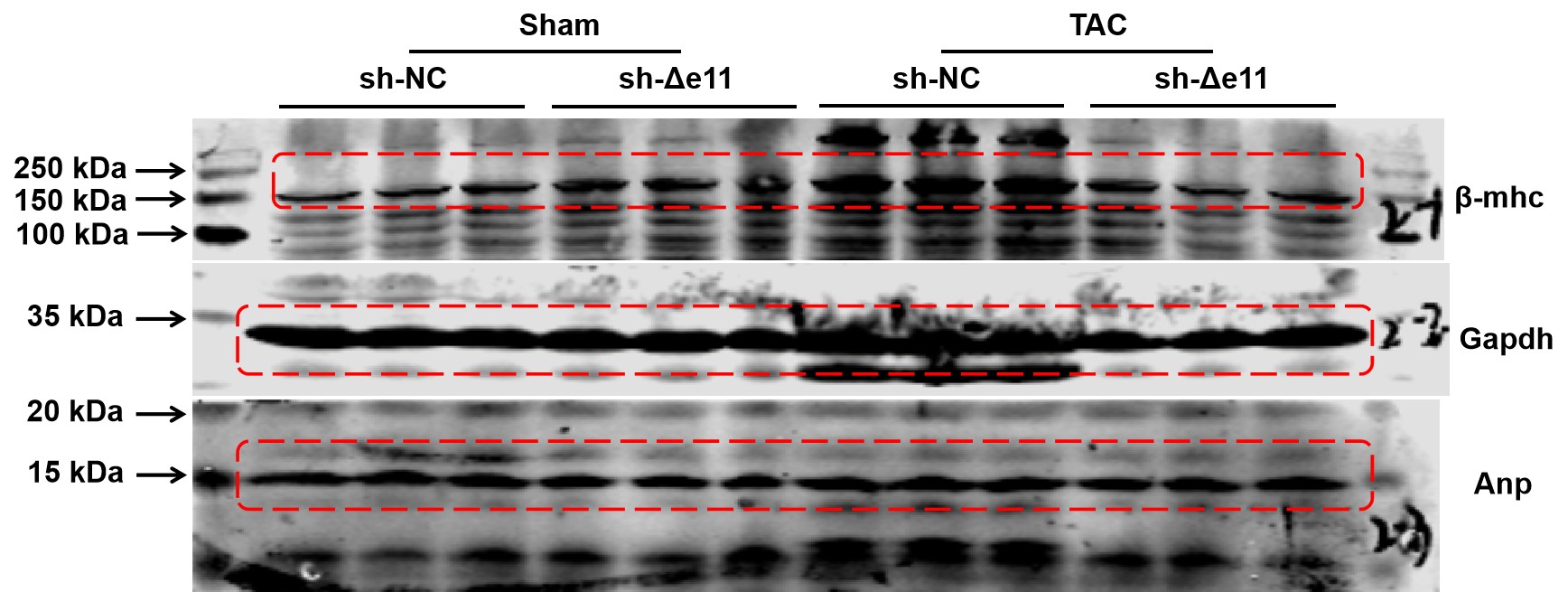

Supplement: Supplementary file 9 — Source data Fig. 6 [file 44321_2025_334_MOESM9_ESM.zip › Figure 6/6H/6H.jpg]

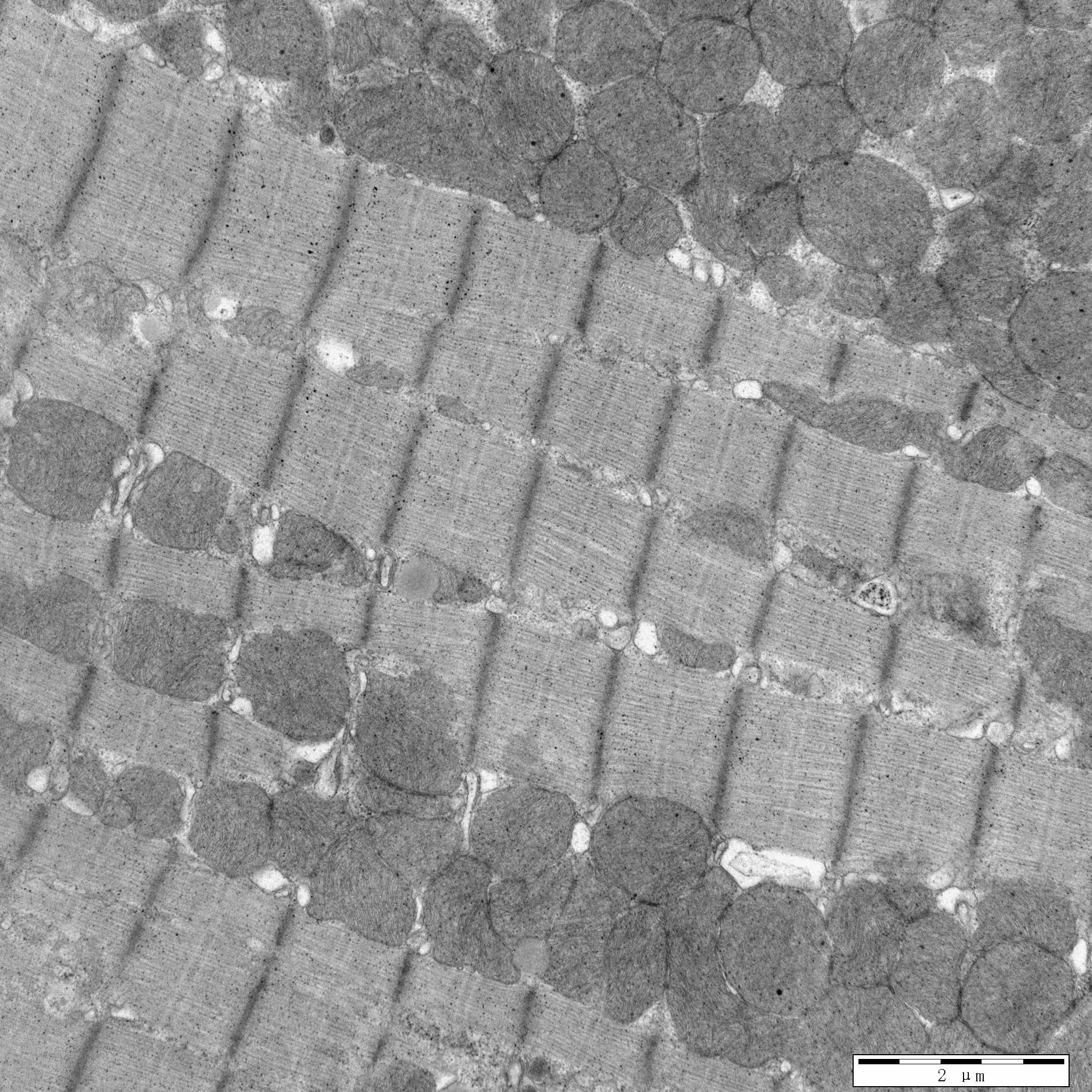

Supplement: Supplementary file 9 — Source data Fig. 6 [file 44321_2025_334_MOESM9_ESM.zip › Figure 6/6J/sh-Vector+Sham-1.JPG]

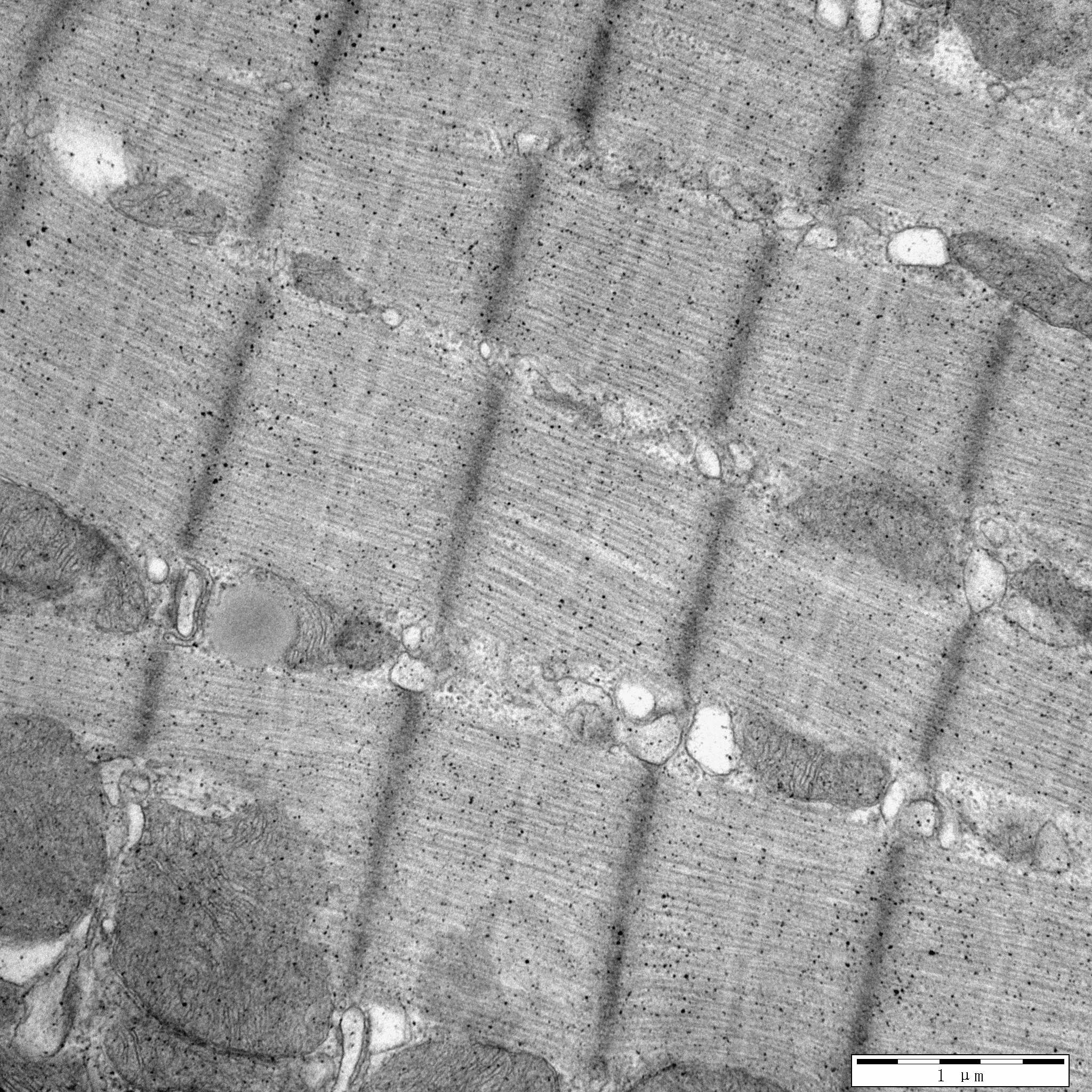

Supplement: Supplementary file 9 — Source data Fig. 6 [file 44321_2025_334_MOESM9_ESM.zip › Figure 6/6J/sh-Vector+Sham-2.JPG]

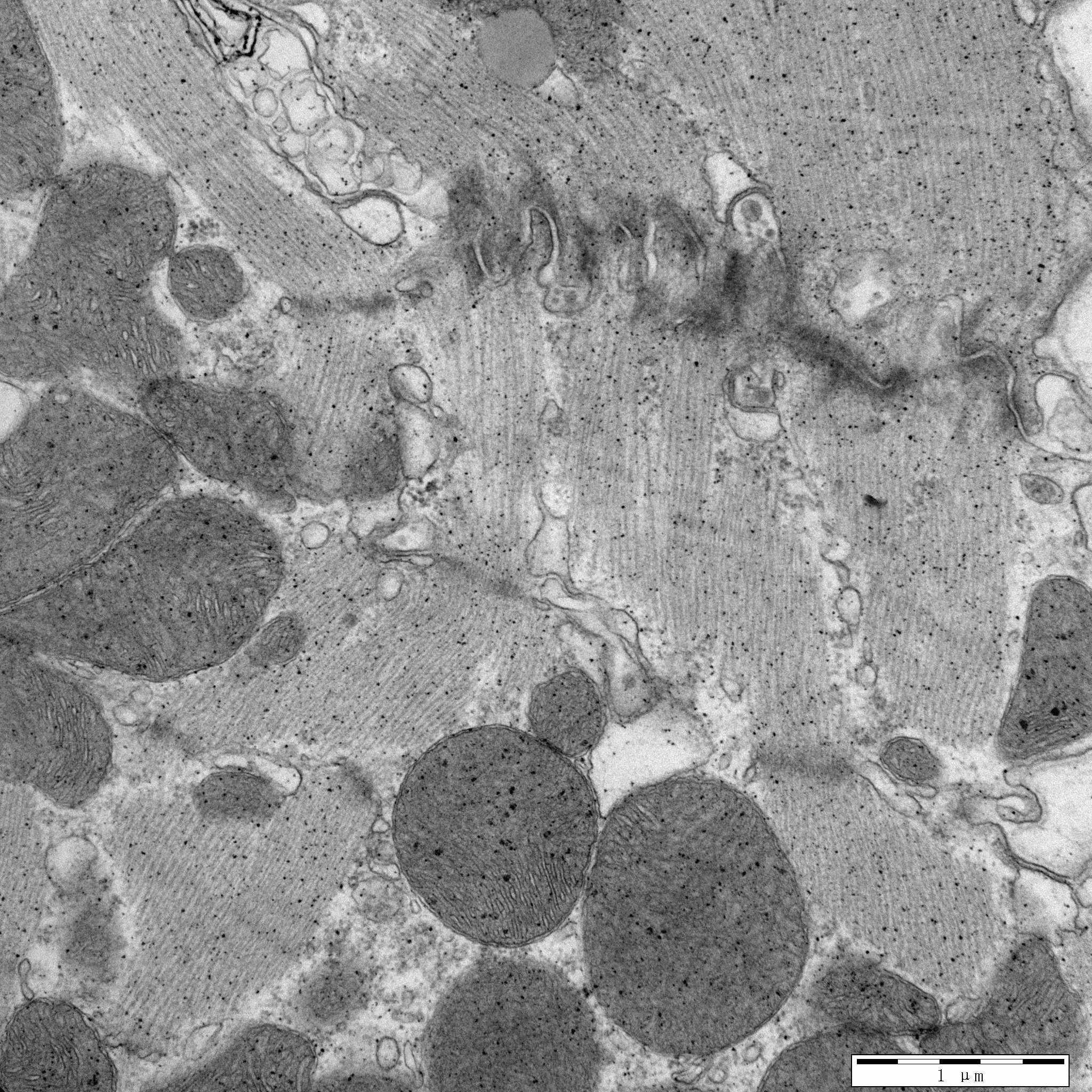

Supplement: Supplementary file 9 — Source data Fig. 6 [file 44321_2025_334_MOESM9_ESM.zip › Figure 6/6J/sh-Vector+TAC-1.JPG]

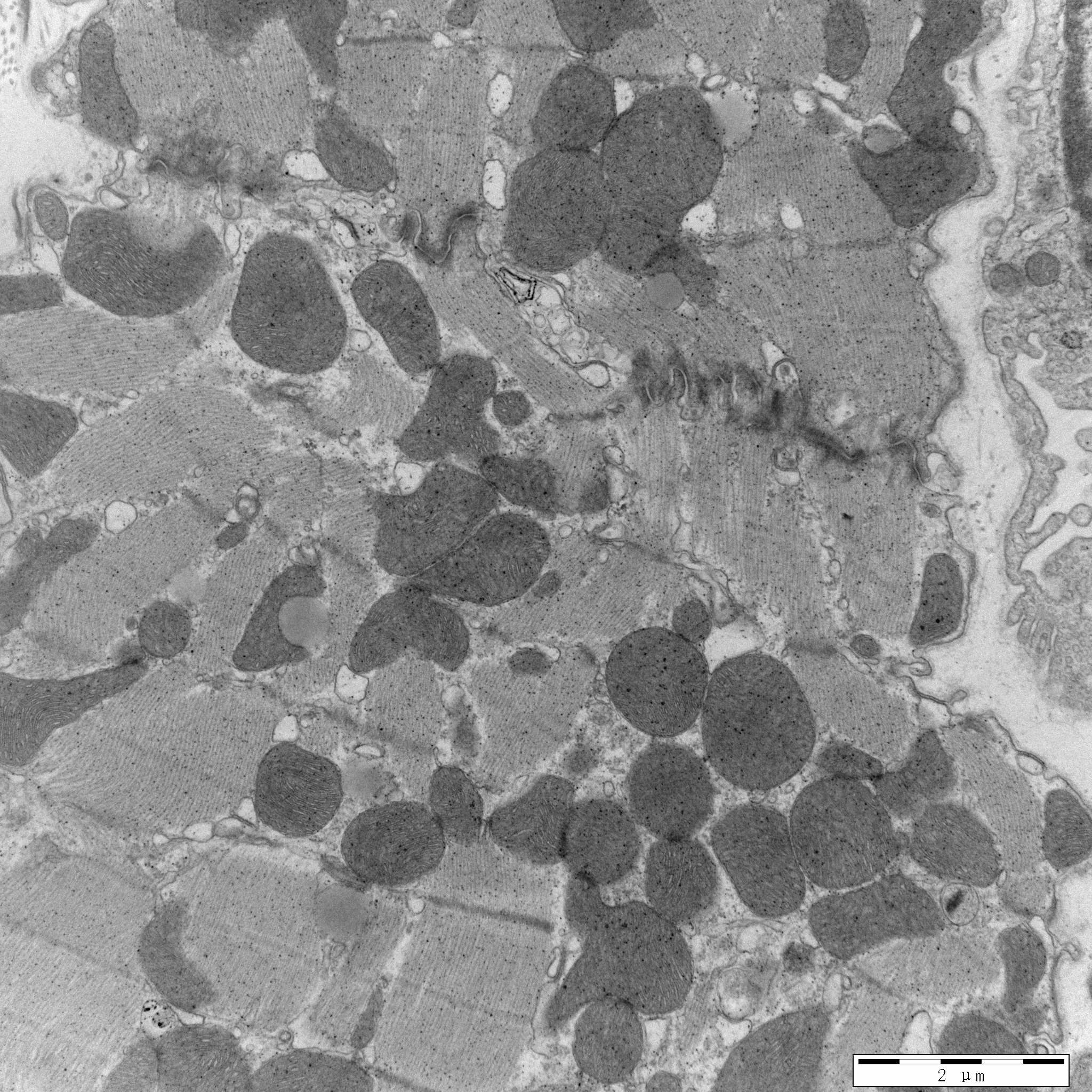

Supplement: Supplementary file 9 — Source data Fig. 6 [file 44321_2025_334_MOESM9_ESM.zip › Figure 6/6J/sh-Vector+TAC-2.JPG]

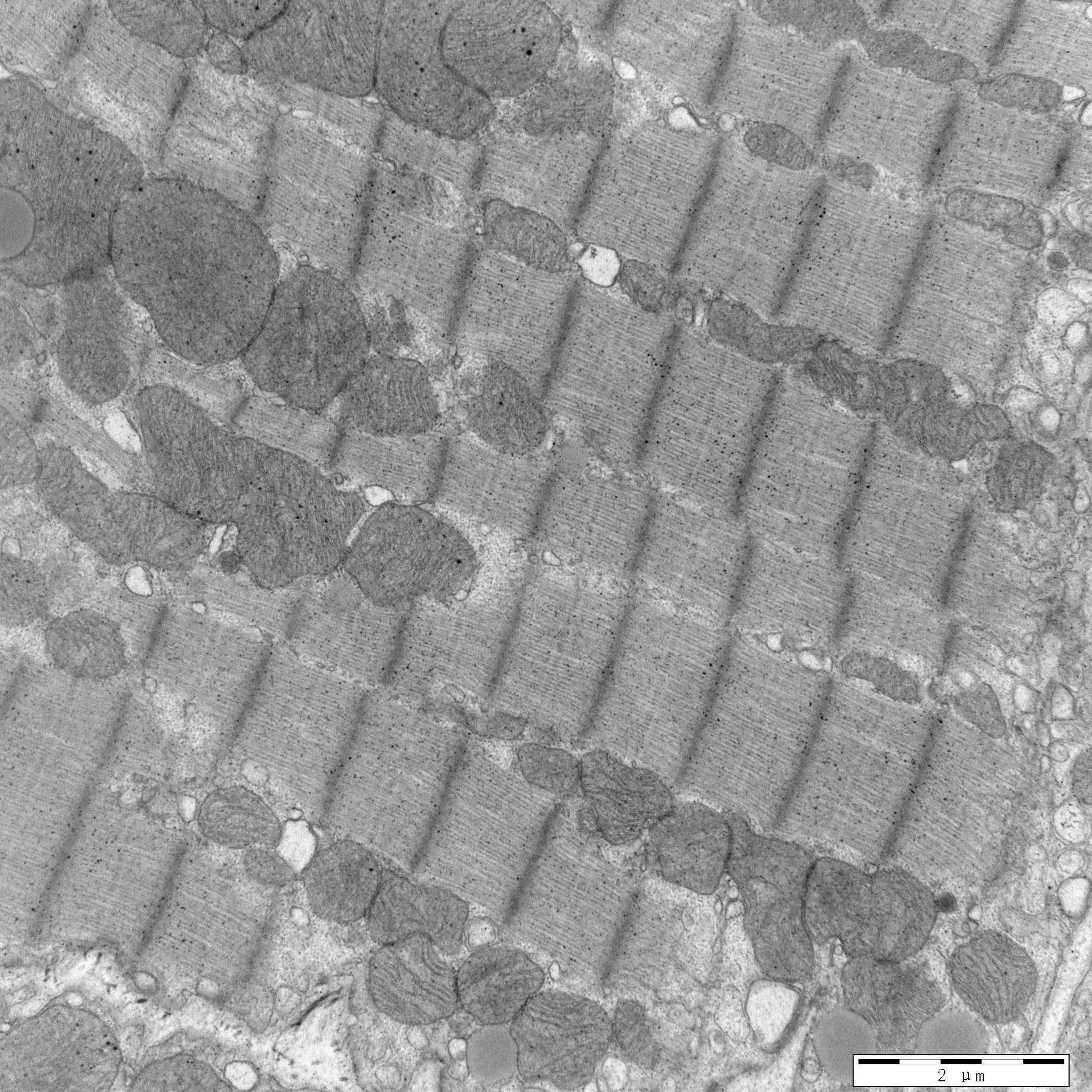

Supplement: Supplementary file 9 — Source data Fig. 6 [file 44321_2025_334_MOESM9_ESM.zip › Figure 6/6J/sh-Δe11+Sham-1.JPG]

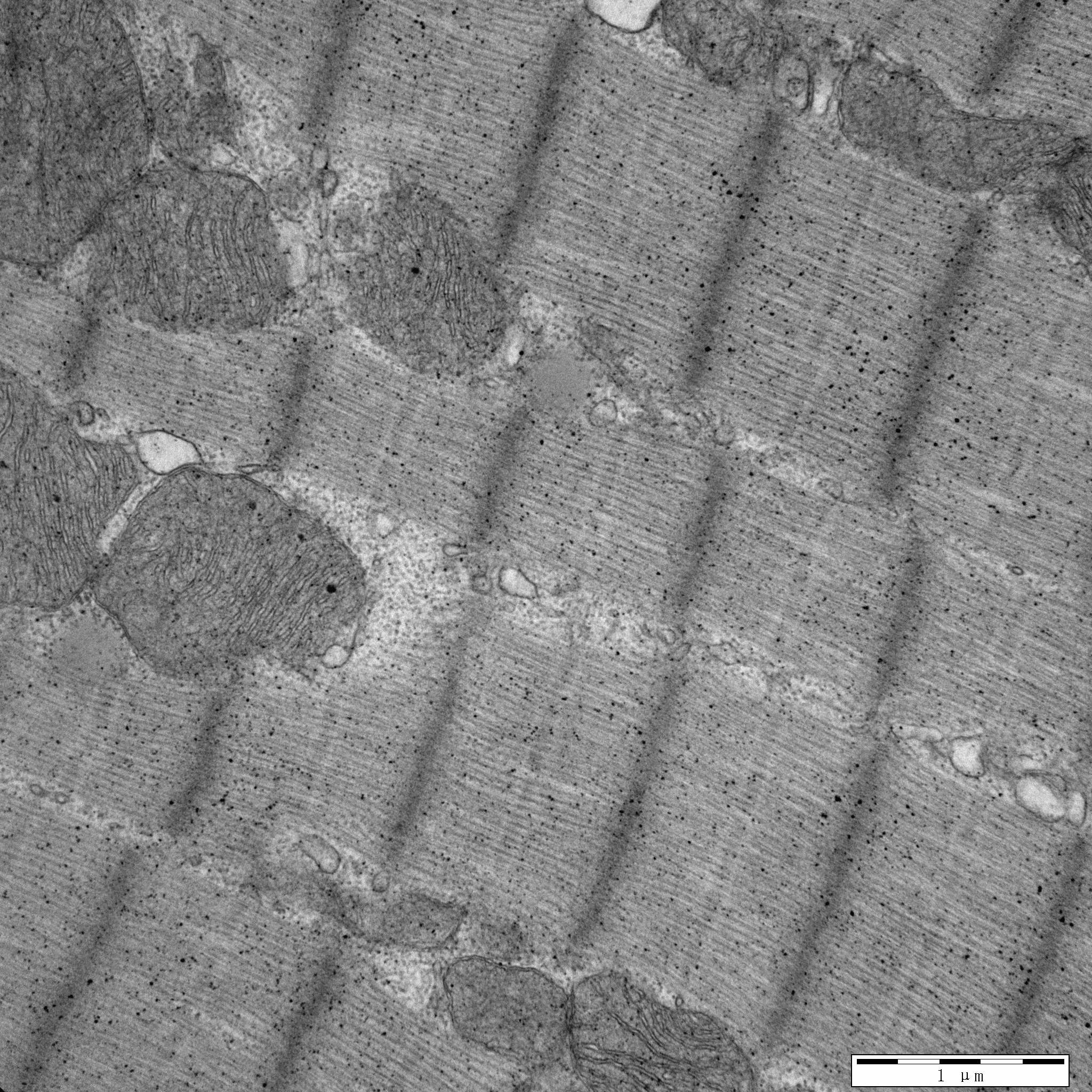

Supplement: Supplementary file 9 — Source data Fig. 6 [file 44321_2025_334_MOESM9_ESM.zip › Figure 6/6J/sh-Δe11+Sham-2.JPG]

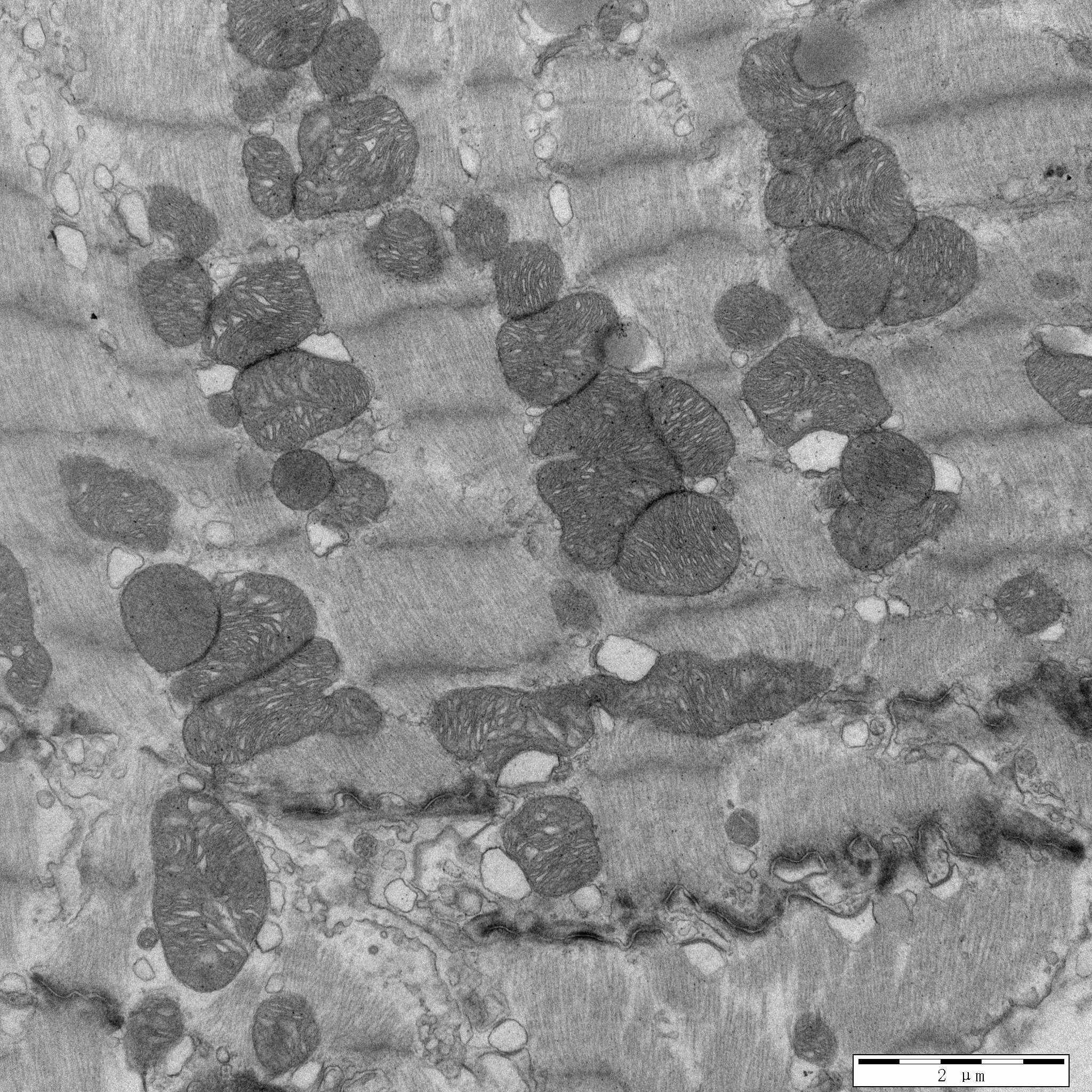

Supplement: Supplementary file 9 — Source data Fig. 6 [file 44321_2025_334_MOESM9_ESM.zip › Figure 6/6J/sh-Δe11+TAC-1.JPG]

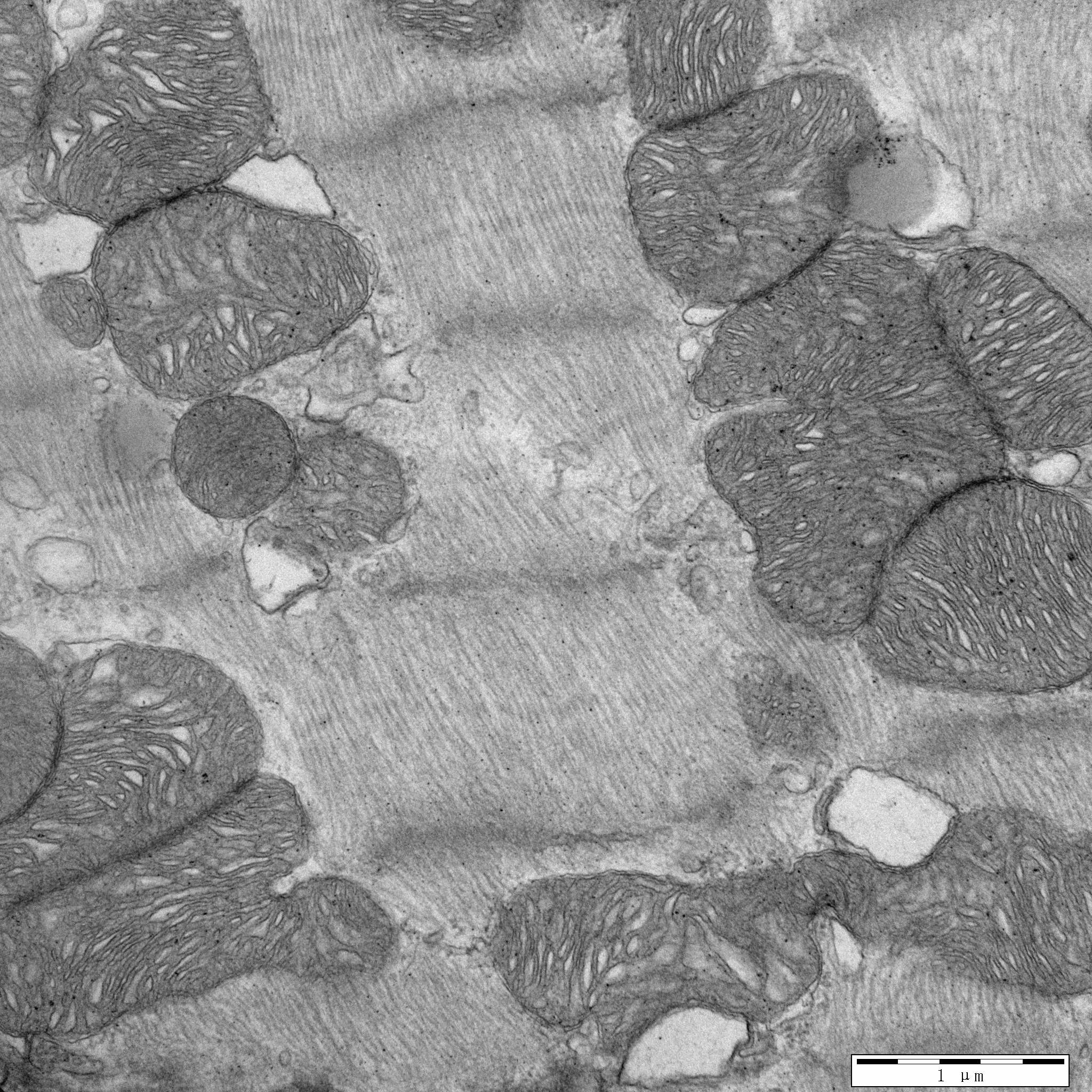

Supplement: Supplementary file 9 — Source data Fig. 6 [file 44321_2025_334_MOESM9_ESM.zip › Figure 6/6J/sh-Δe11+TAC-2.JPG]

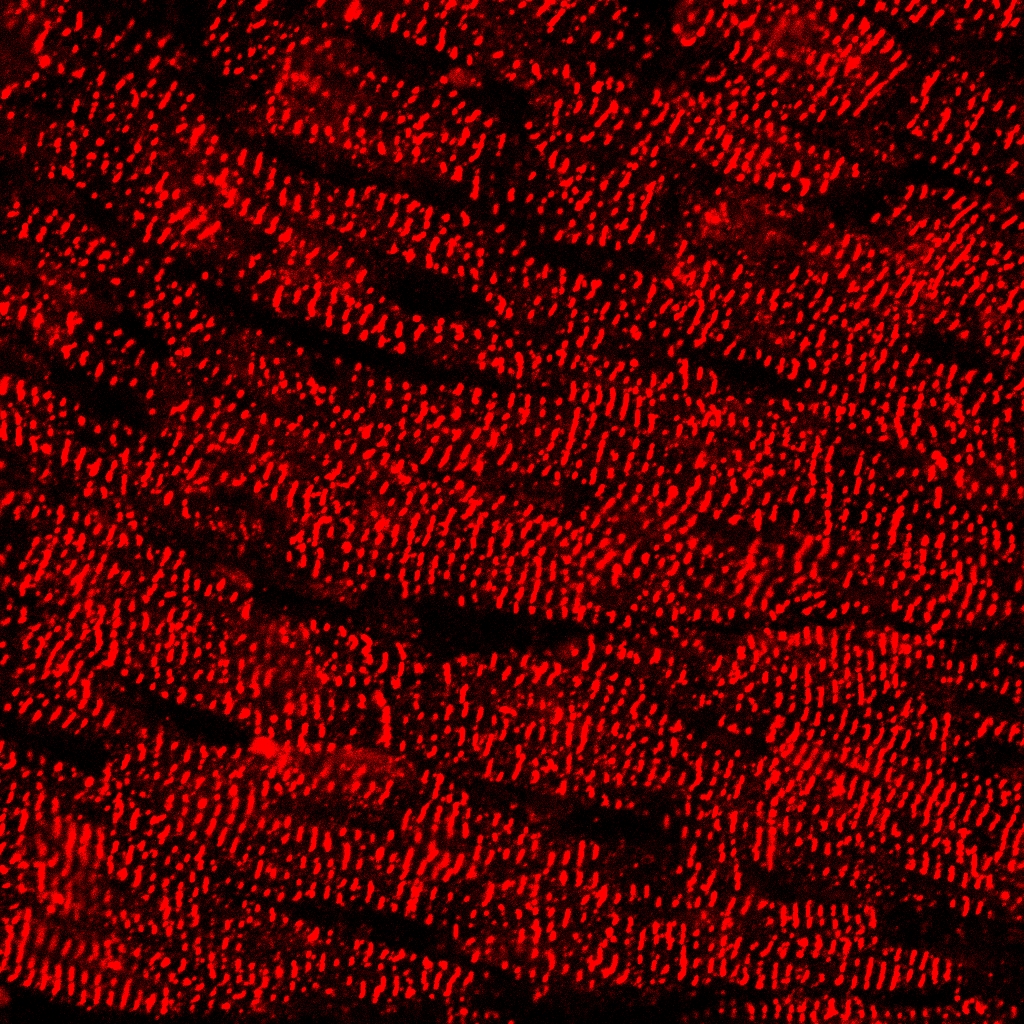

Supplement: Supplementary file 9 — Source data Fig. 6 [file 44321_2025_334_MOESM9_ESM.zip › Figure 6/6K/sh-Vector+Sham-ACTN2.jpeg]
