# Supplementary figures and images for: RBMS1 orchestrates cardiac hypertrophy by facilitating CTTN splice-switching and sarcomere dynamics (part 3 of 4)
Source: EMBO Mol Med. 2025 Nov 10;17(12):3555–85. doi: 10.1038/s44321-025-00334-z (PMC12686484; doi:10.1038/s44321-025-00334-z)

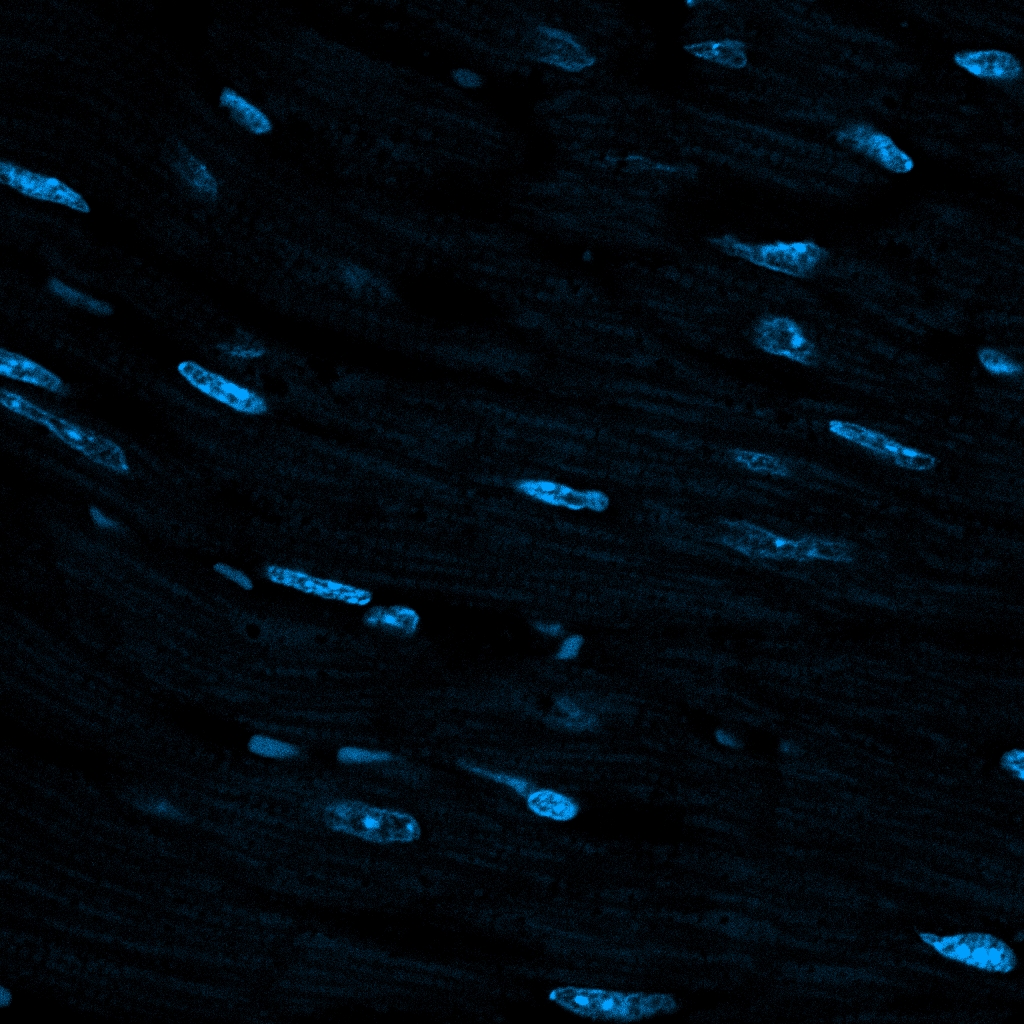

Supplement: Supplementary file 9 — Source data Fig. 6 [file 44321_2025_334_MOESM9_ESM.zip › Figure 6/6K/sh-Vector+Sham-DAPI.jpeg]

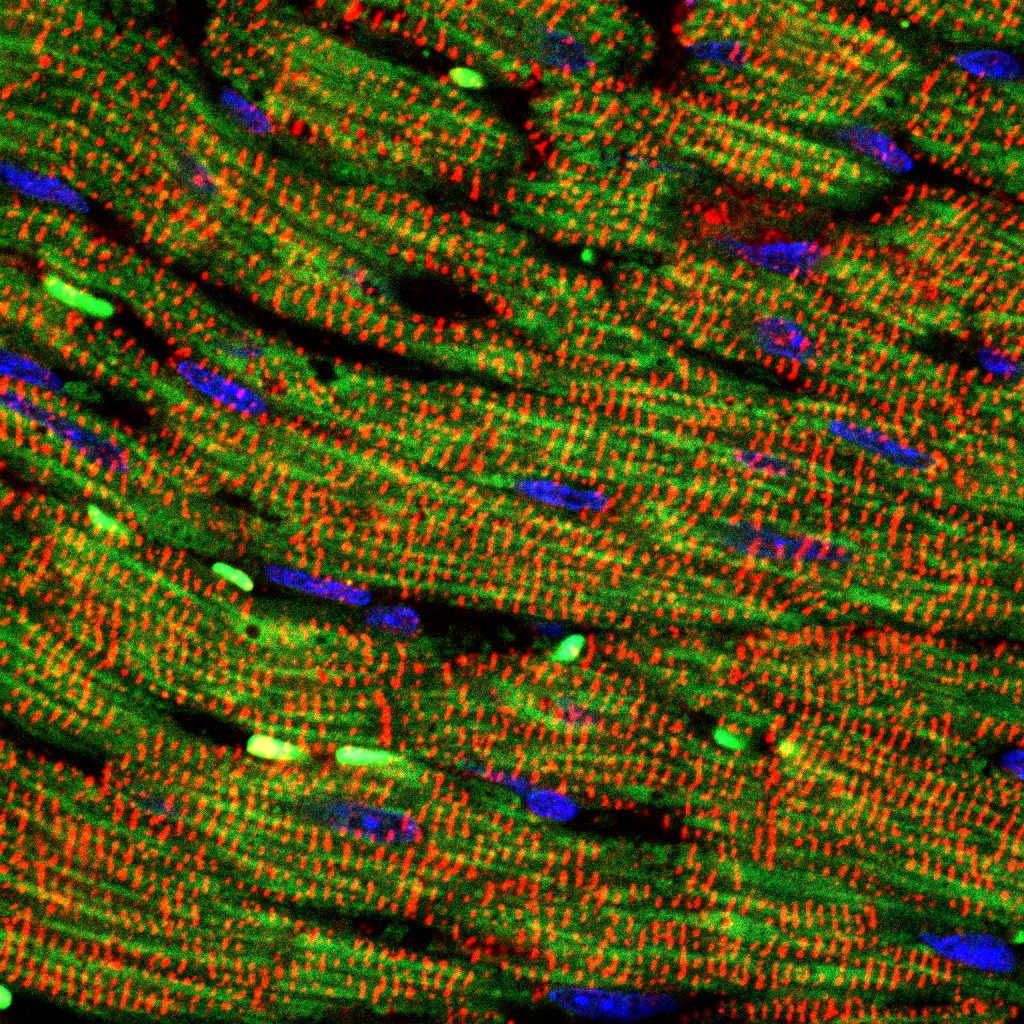

Supplement: Supplementary file 9 — Source data Fig. 6 [file 44321_2025_334_MOESM9_ESM.zip › Figure 6/6K/sh-Vector+Sham-Merge.jpg]

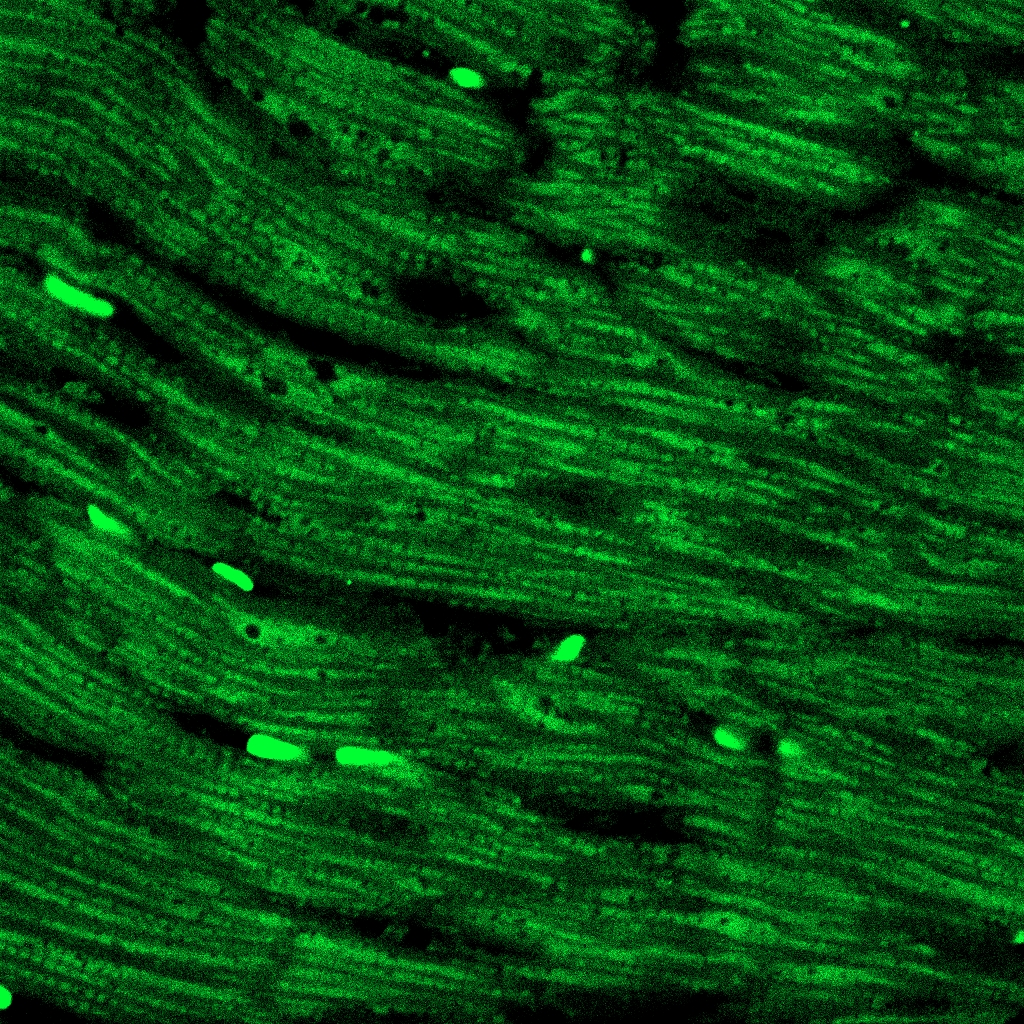

Supplement: Supplementary file 9 — Source data Fig. 6 [file 44321_2025_334_MOESM9_ESM.zip › Figure 6/6K/sh-Vector+Sham-α-ACTININ.jpeg]

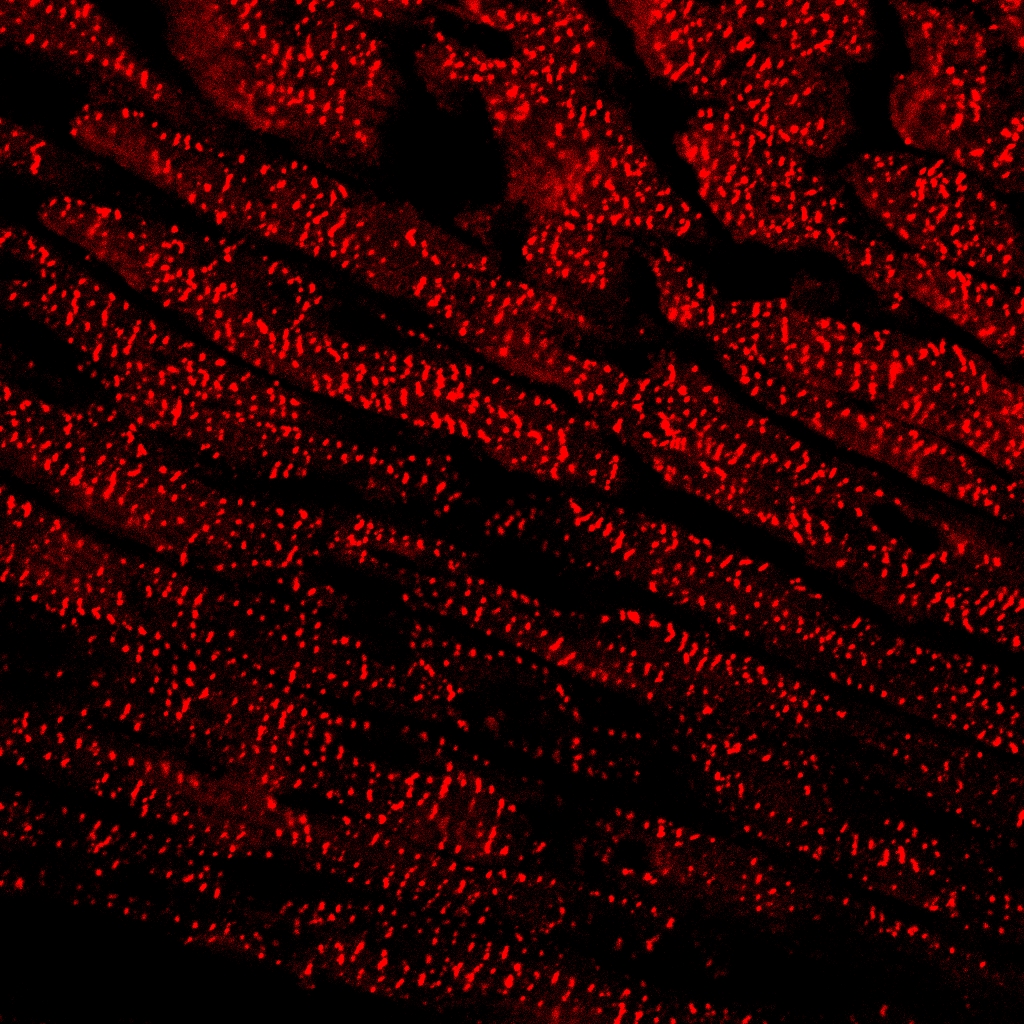

Supplement: Supplementary file 9 — Source data Fig. 6 [file 44321_2025_334_MOESM9_ESM.zip › Figure 6/6K/sh-Vector+TAC-ACTN2.jpeg]

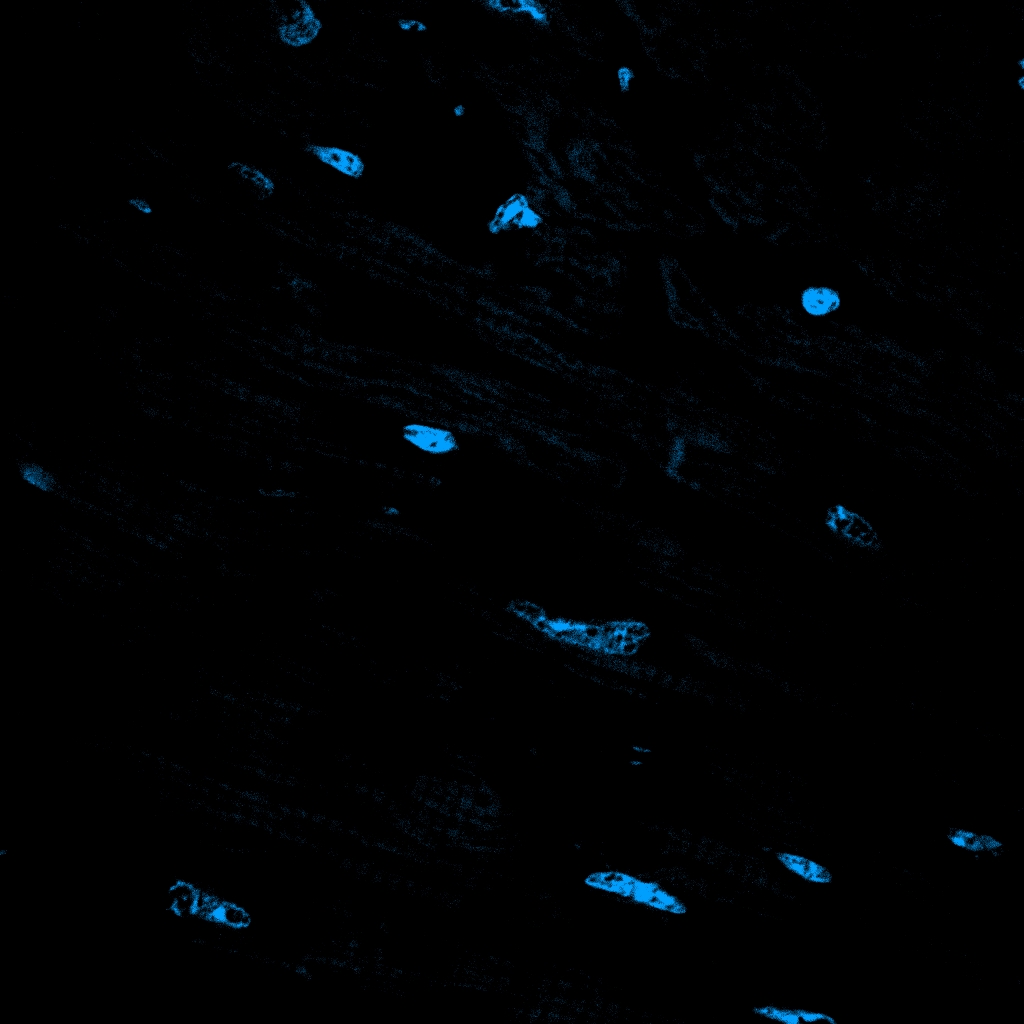

Supplement: Supplementary file 9 — Source data Fig. 6 [file 44321_2025_334_MOESM9_ESM.zip › Figure 6/6K/sh-Vector+TAC-DAPI.jpeg]

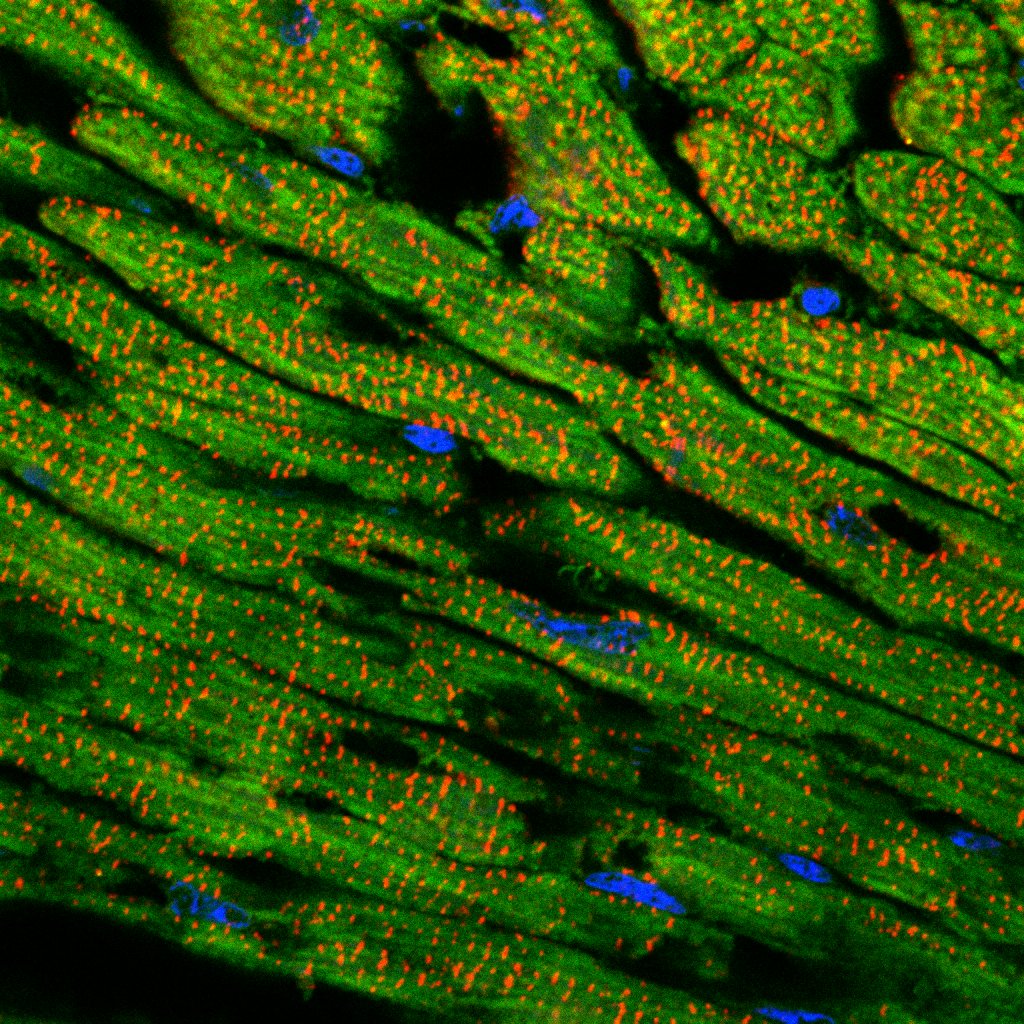

Supplement: Supplementary file 9 — Source data Fig. 6 [file 44321_2025_334_MOESM9_ESM.zip › Figure 6/6K/sh-Vector+TAC-Merge.jpg]

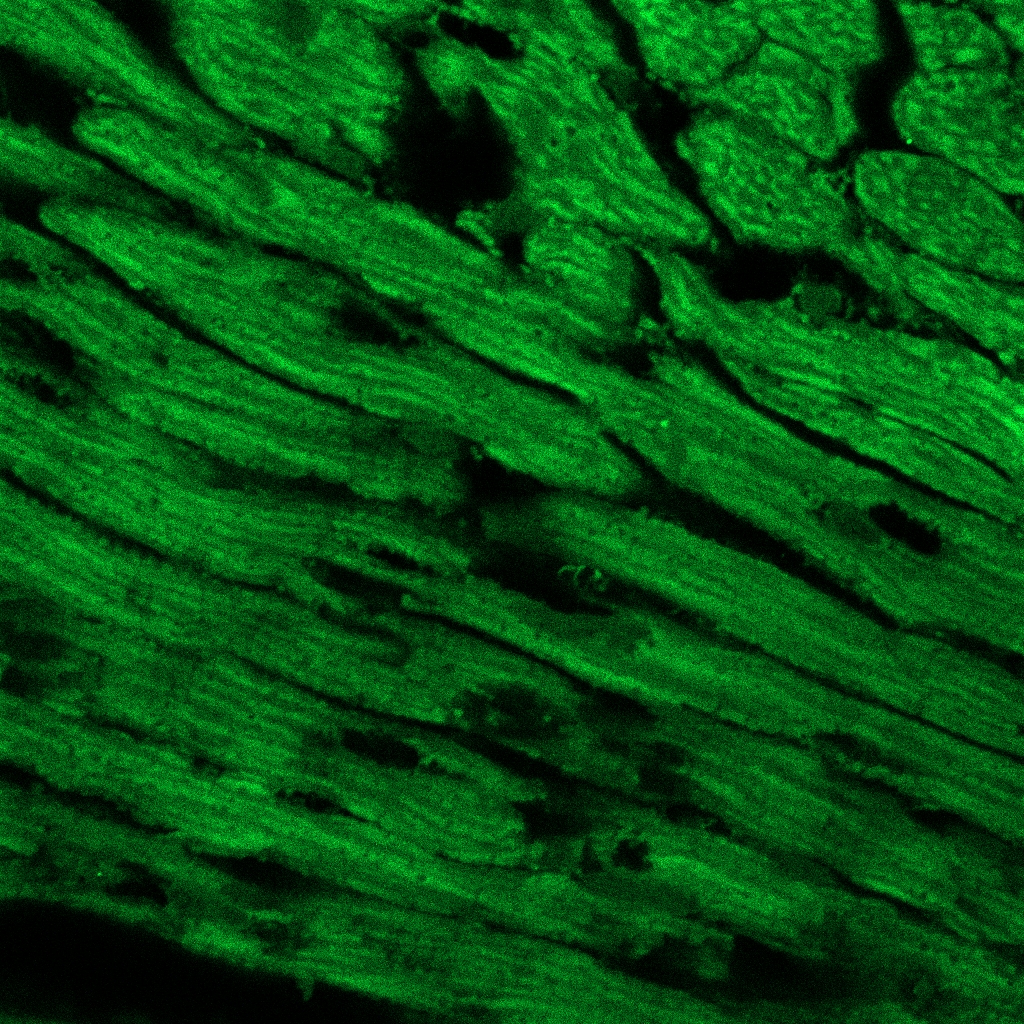

Supplement: Supplementary file 9 — Source data Fig. 6 [file 44321_2025_334_MOESM9_ESM.zip › Figure 6/6K/sh-Vector+TAC-α-ACTININ.jpeg]

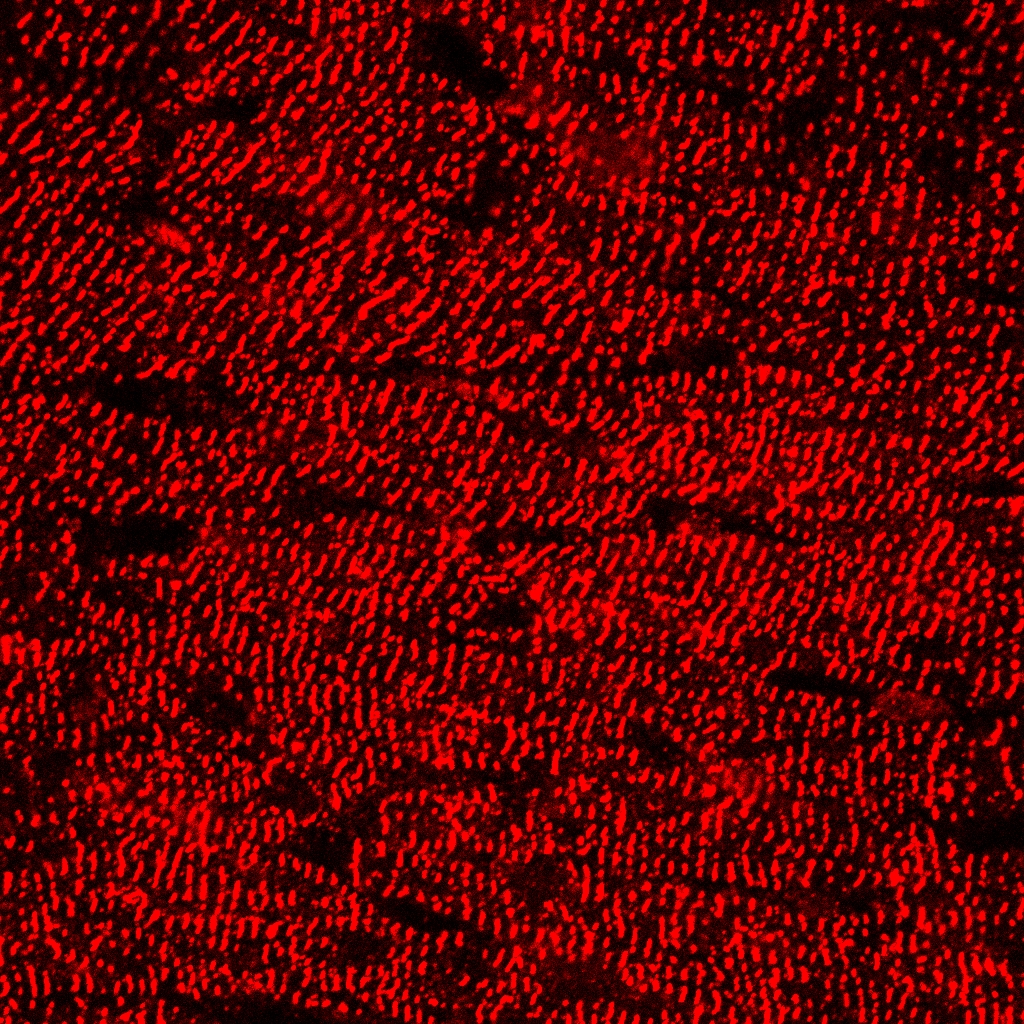

Supplement: Supplementary file 9 — Source data Fig. 6 [file 44321_2025_334_MOESM9_ESM.zip › Figure 6/6K/sh-Δe11+Sham-ACTN2.jpeg]

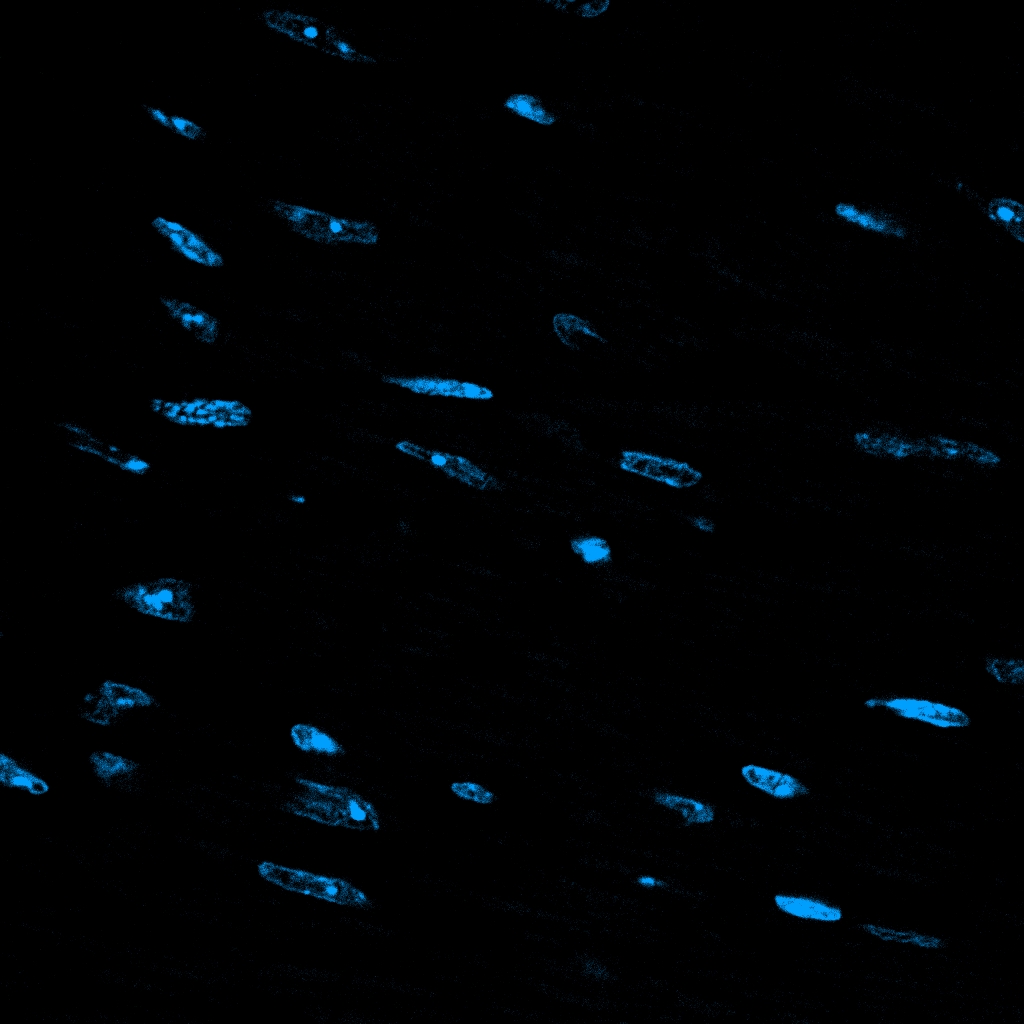

Supplement: Supplementary file 9 — Source data Fig. 6 [file 44321_2025_334_MOESM9_ESM.zip › Figure 6/6K/sh-Δe11+Sham-DAPI.jpeg]

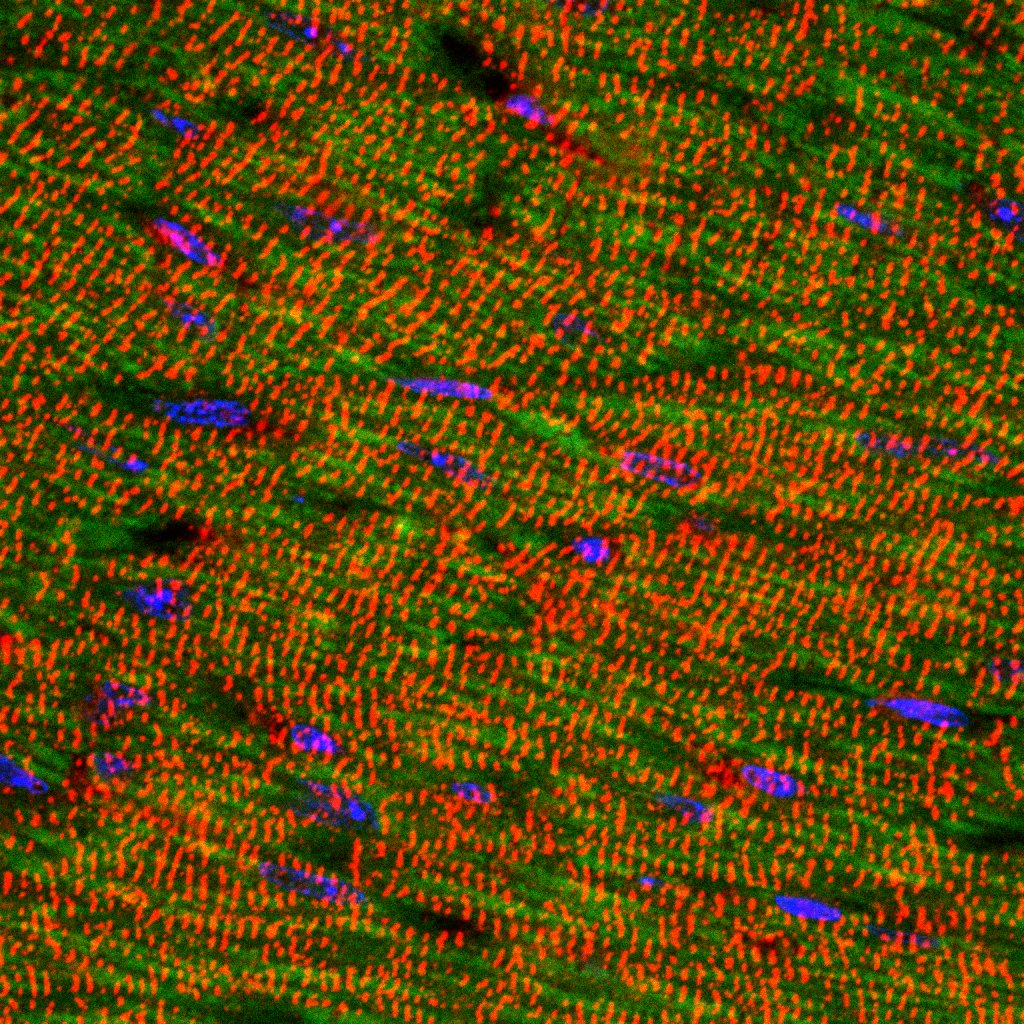

Supplement: Supplementary file 9 — Source data Fig. 6 [file 44321_2025_334_MOESM9_ESM.zip › Figure 6/6K/sh-Δe11+Sham-Merge.jpg]

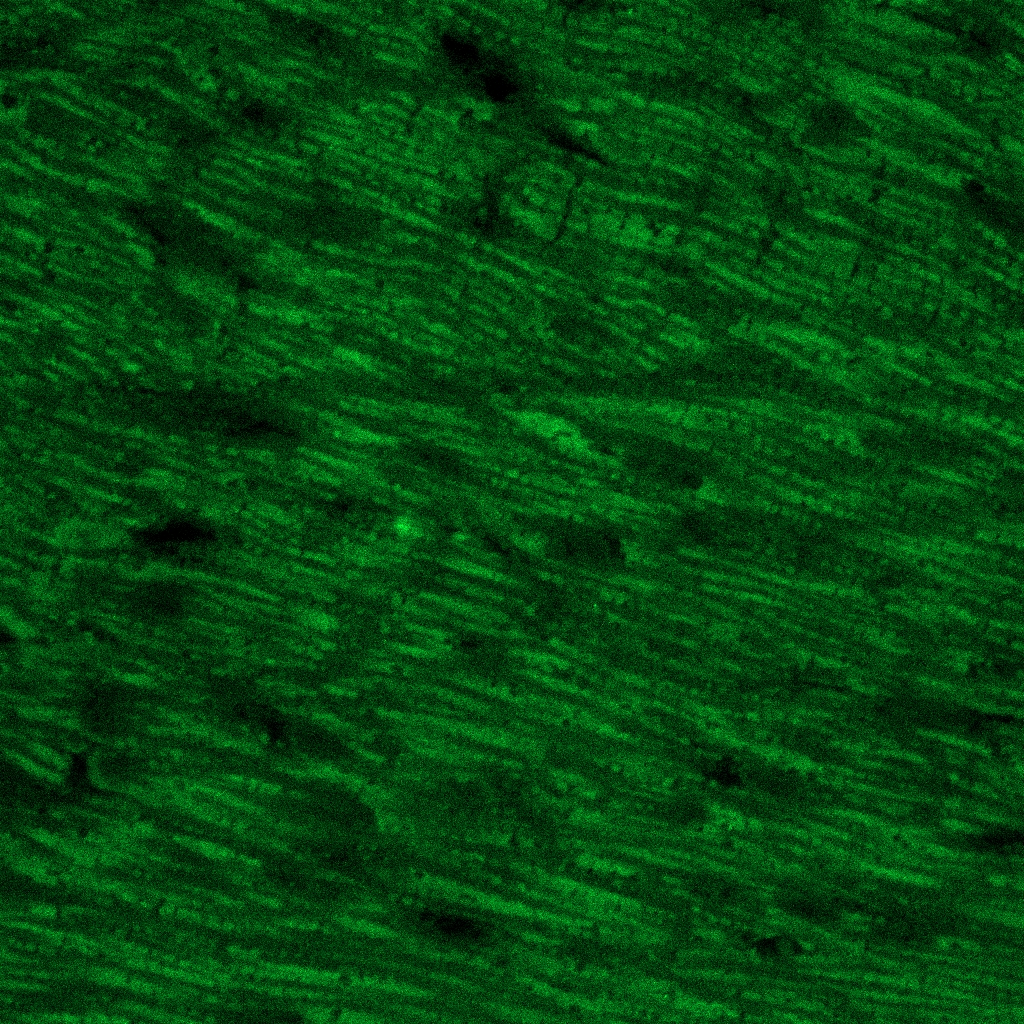

Supplement: Supplementary file 9 — Source data Fig. 6 [file 44321_2025_334_MOESM9_ESM.zip › Figure 6/6K/sh-Δe11+Sham-α-ACTININ.jpeg]

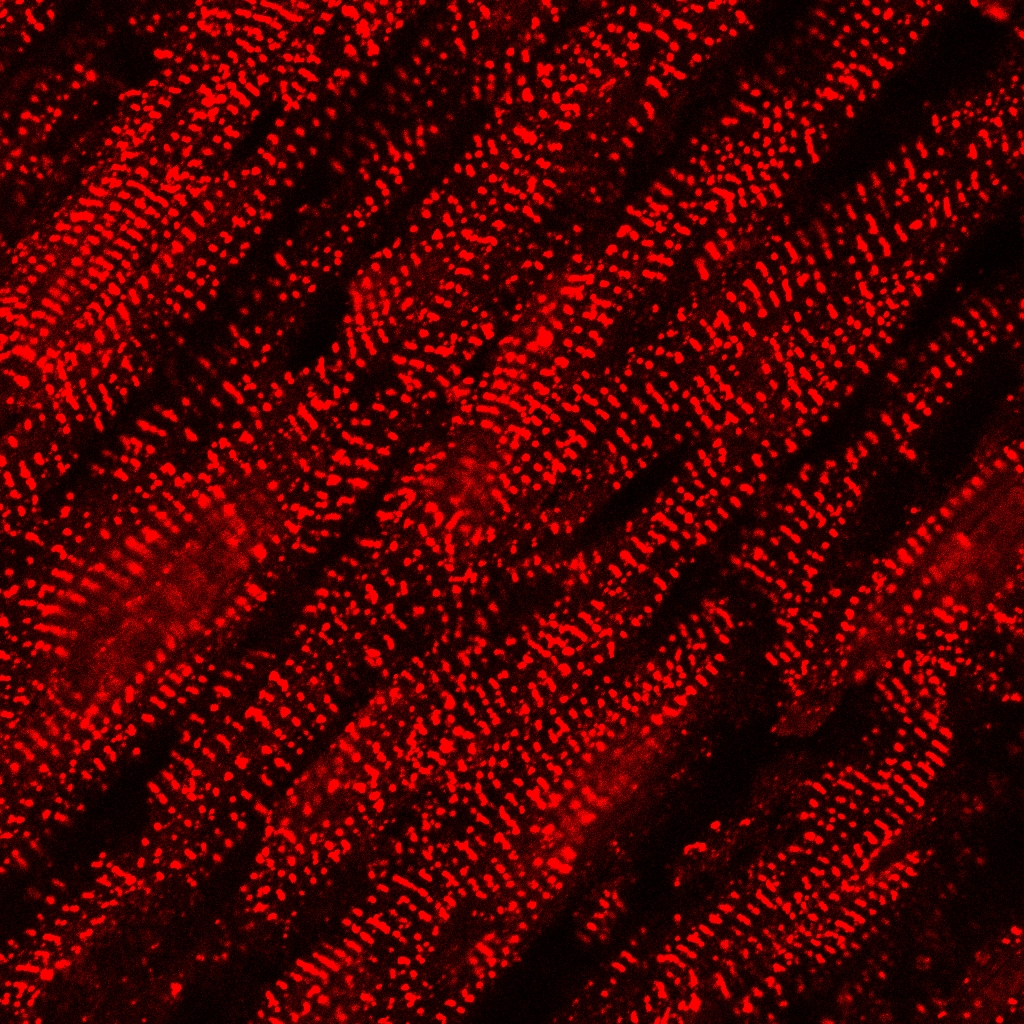

Supplement: Supplementary file 9 — Source data Fig. 6 [file 44321_2025_334_MOESM9_ESM.zip › Figure 6/6K/sh-Δe11+TAC-ACTN2.jpeg]

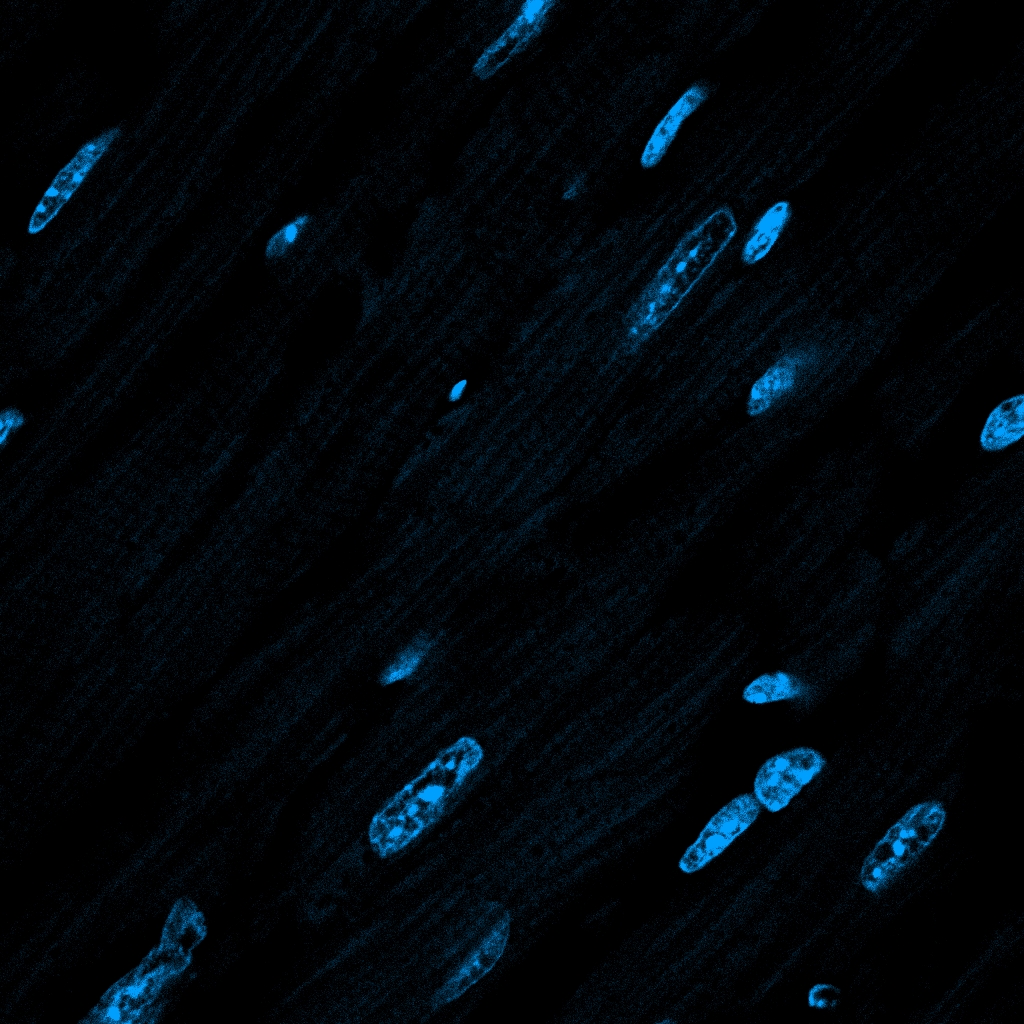

Supplement: Supplementary file 9 — Source data Fig. 6 [file 44321_2025_334_MOESM9_ESM.zip › Figure 6/6K/sh-Δe11+TAC-DAPI.jpeg]

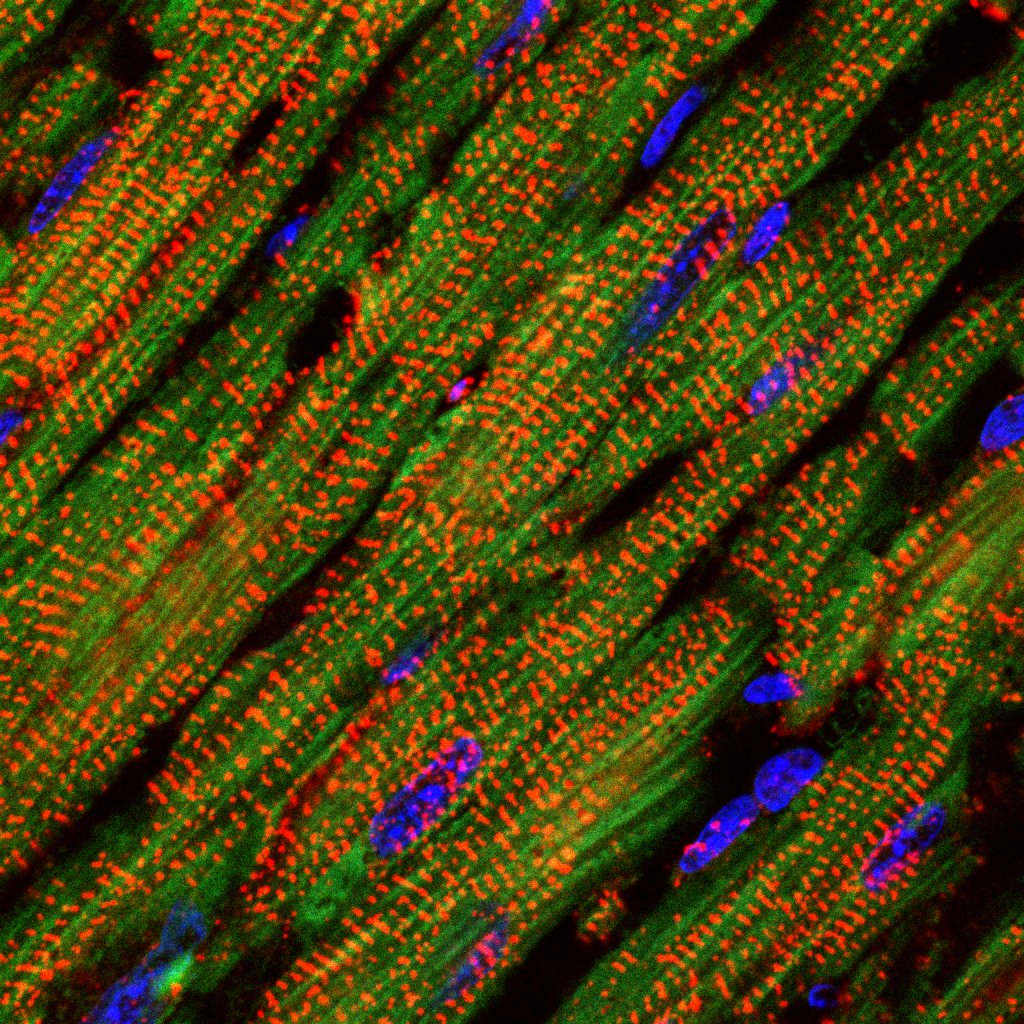

Supplement: Supplementary file 9 — Source data Fig. 6 [file 44321_2025_334_MOESM9_ESM.zip › Figure 6/6K/sh-Δe11+TAC-Merge.jpg]

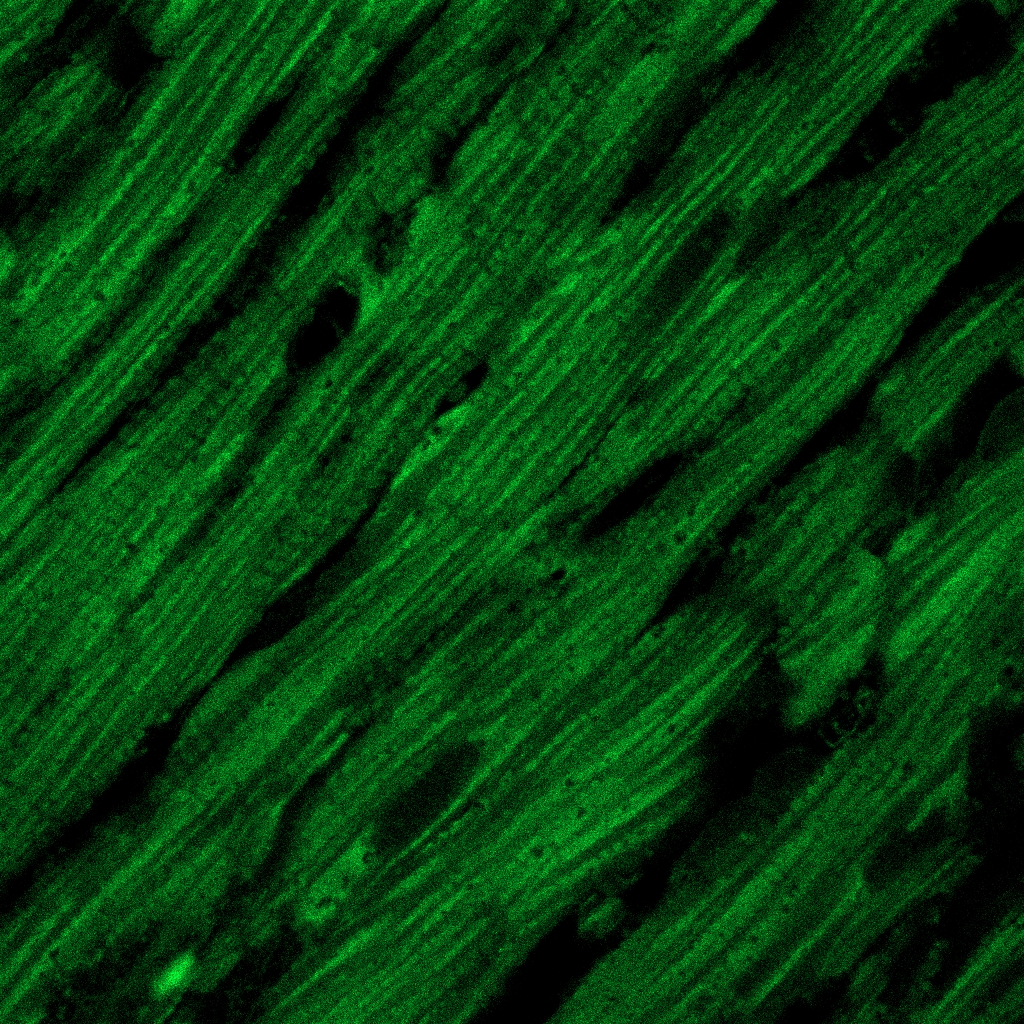

Supplement: Supplementary file 9 — Source data Fig. 6 [file 44321_2025_334_MOESM9_ESM.zip › Figure 6/6K/sh-Δe11+TAC-α-ACTININ.jpeg]

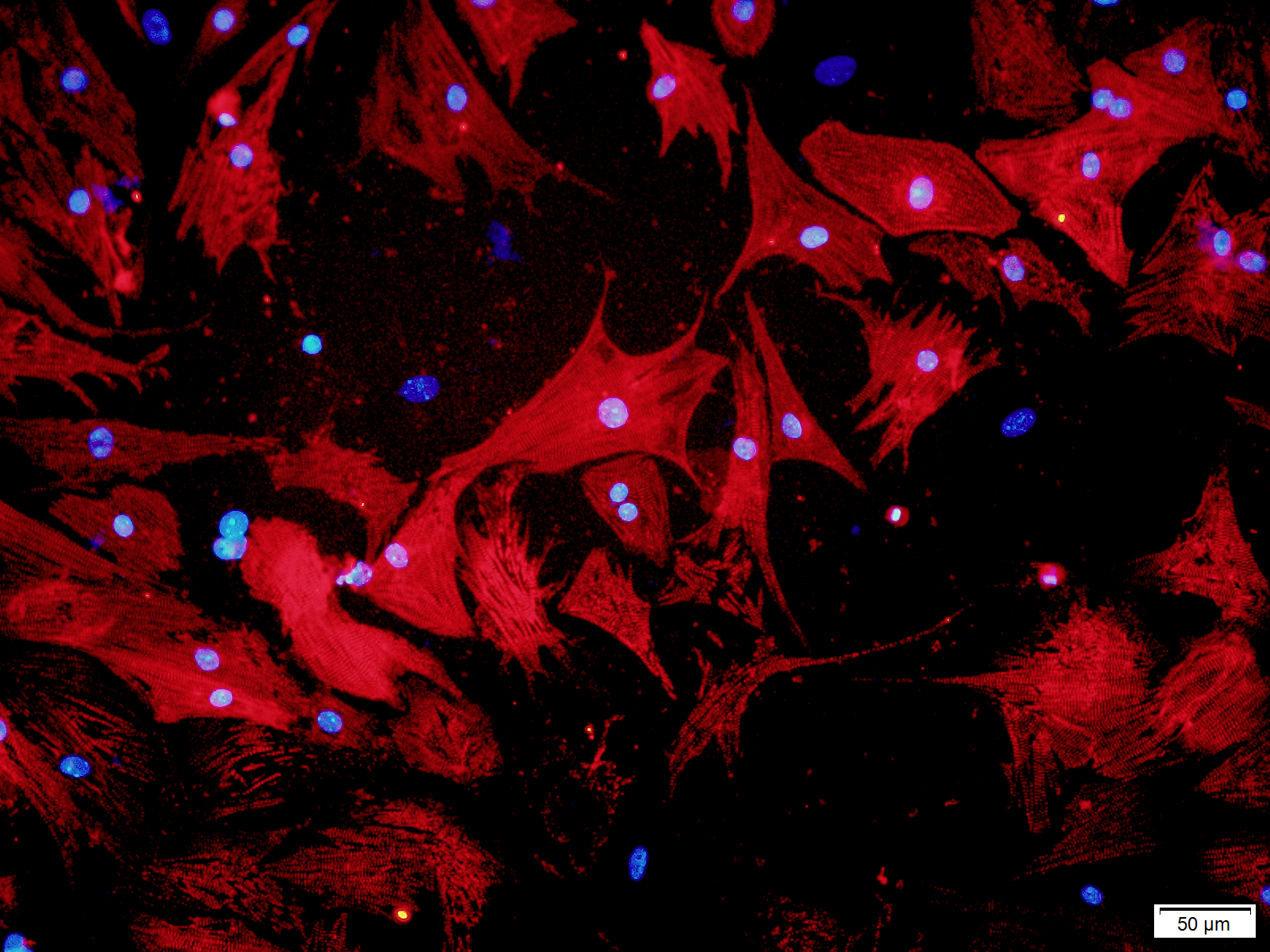

Supplement: Supplementary file 9 — Source data Fig. 6 [file 44321_2025_334_MOESM9_ESM.zip › Figure 6/6L/Ang II+si-NC.tif]

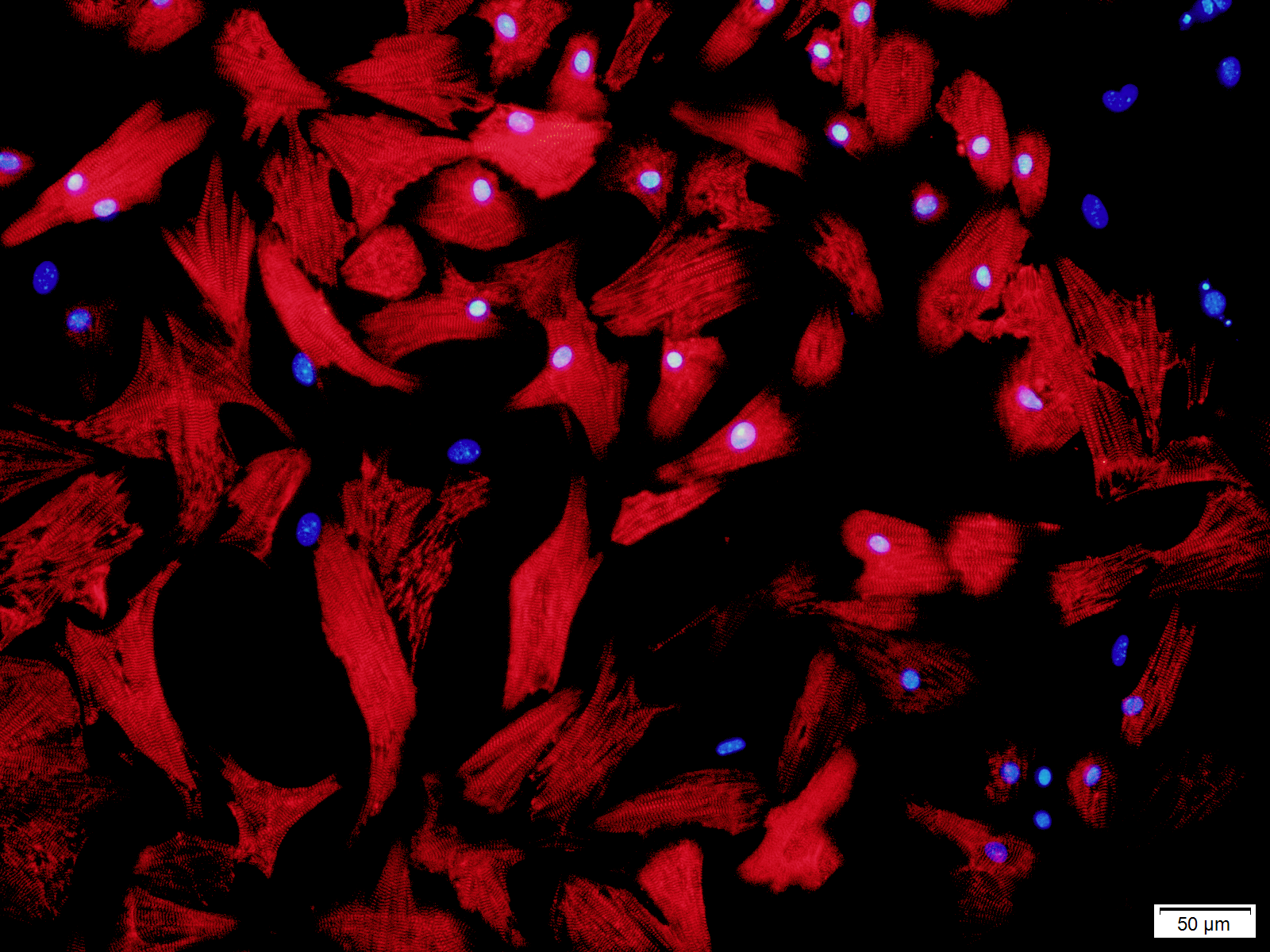

Supplement: Supplementary file 9 — Source data Fig. 6 [file 44321_2025_334_MOESM9_ESM.zip › Figure 6/6L/Ang II+si-Δe11-1.tif]

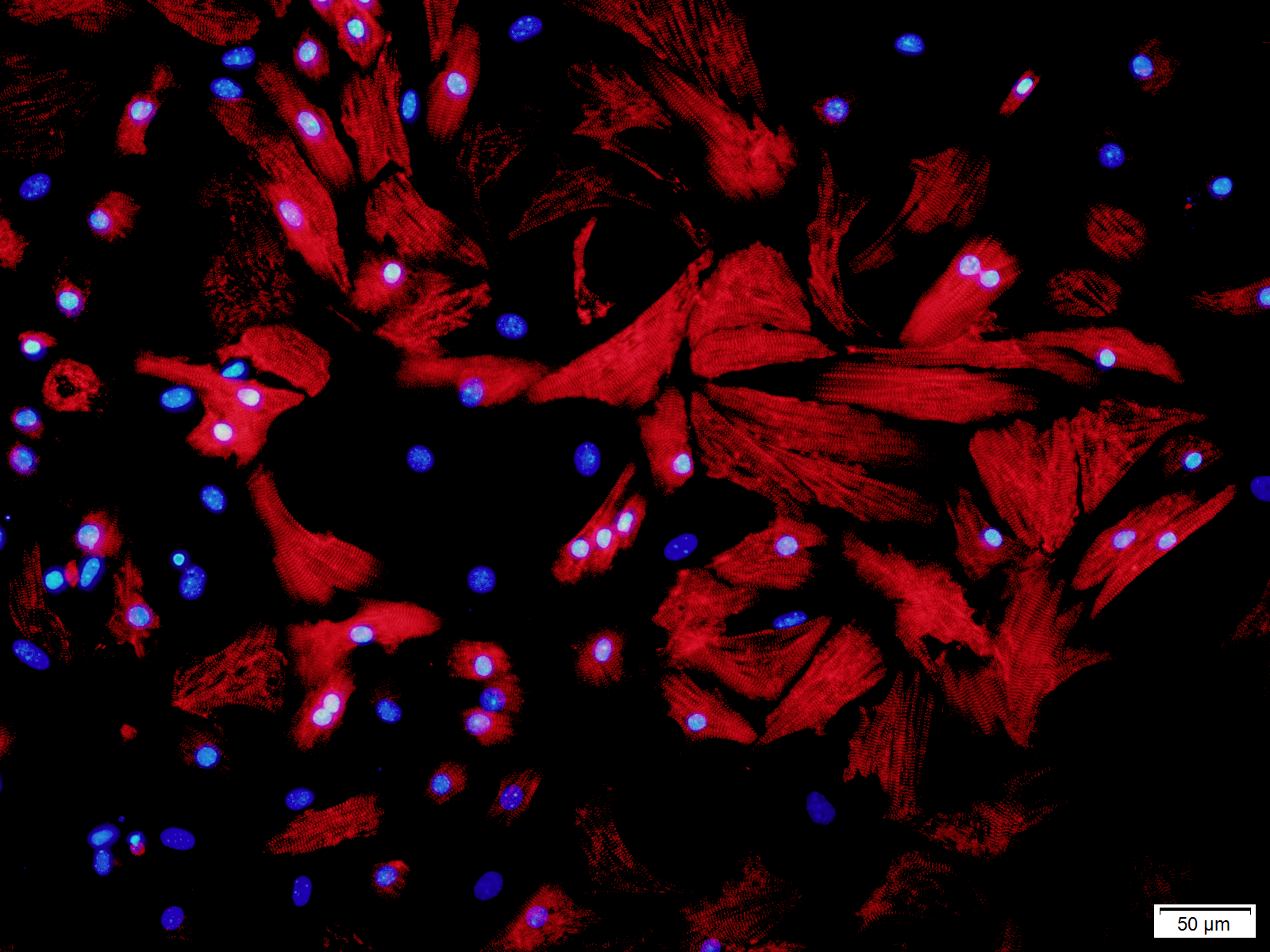

Supplement: Supplementary file 9 — Source data Fig. 6 [file 44321_2025_334_MOESM9_ESM.zip › Figure 6/6L/Ang II+si-Δe11-3.tif]

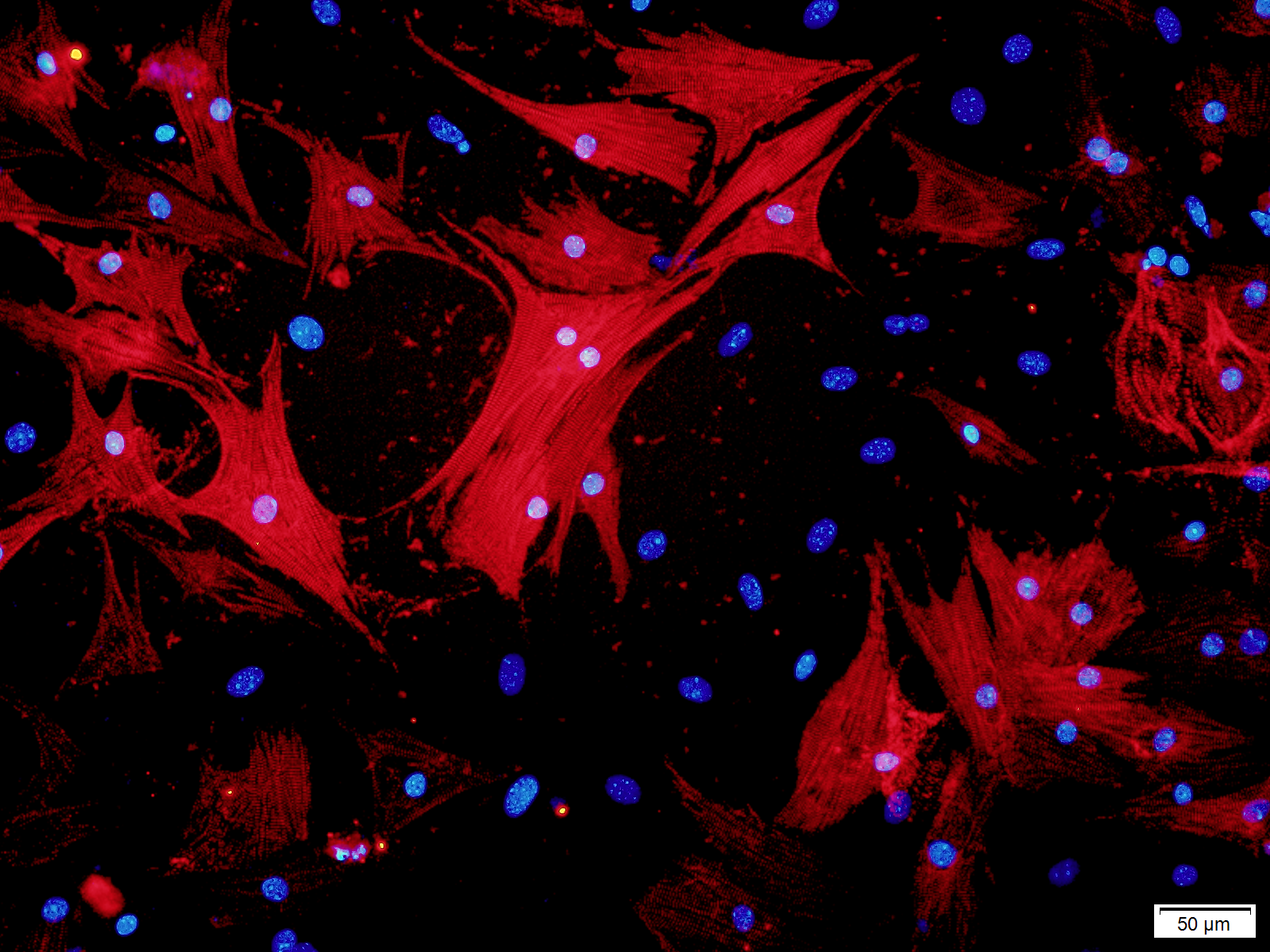

Supplement: Supplementary file 9 — Source data Fig. 6 [file 44321_2025_334_MOESM9_ESM.zip › Figure 6/6L/Ang II.tif]

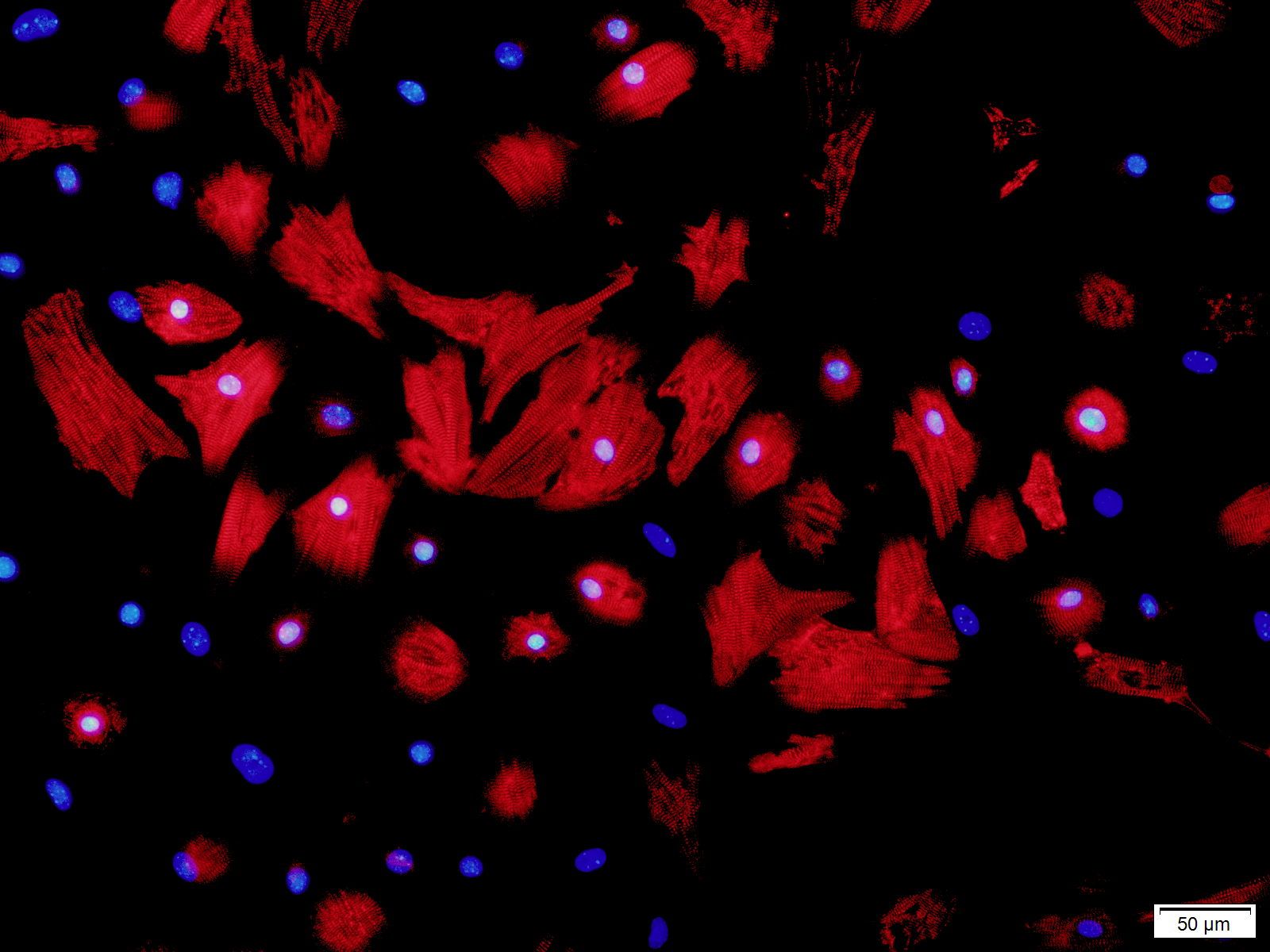

Supplement: Supplementary file 9 — Source data Fig. 6 [file 44321_2025_334_MOESM9_ESM.zip › Figure 6/6L/Control.tif]

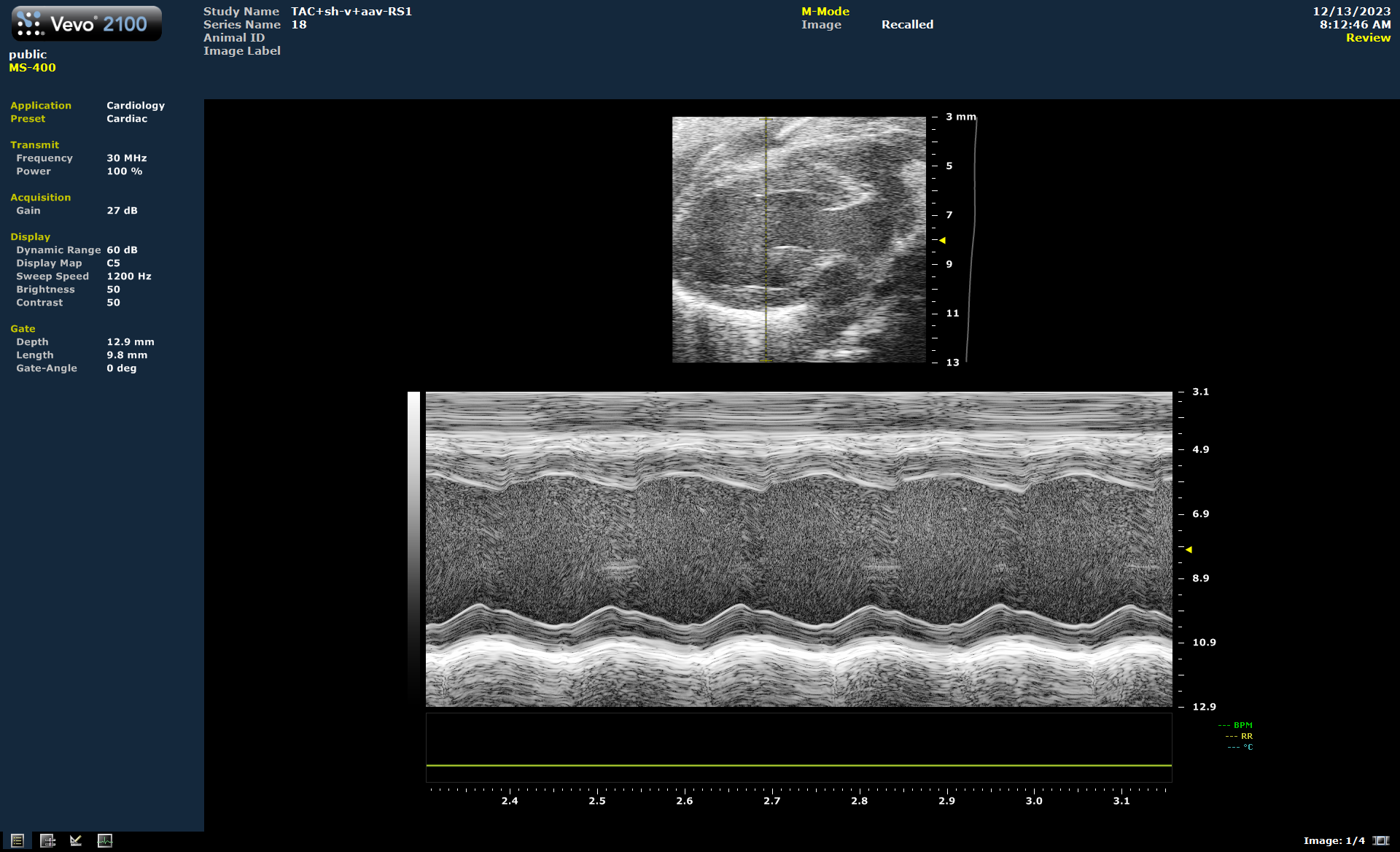

Supplement: Supplementary file 10 — Source data Fig. 7 [file 44321_2025_334_MOESM10_ESM.zip › Figure 7/7B/B Mode/TAC+AAV9-sh-Vector+AAV9-RBMS1.tif]

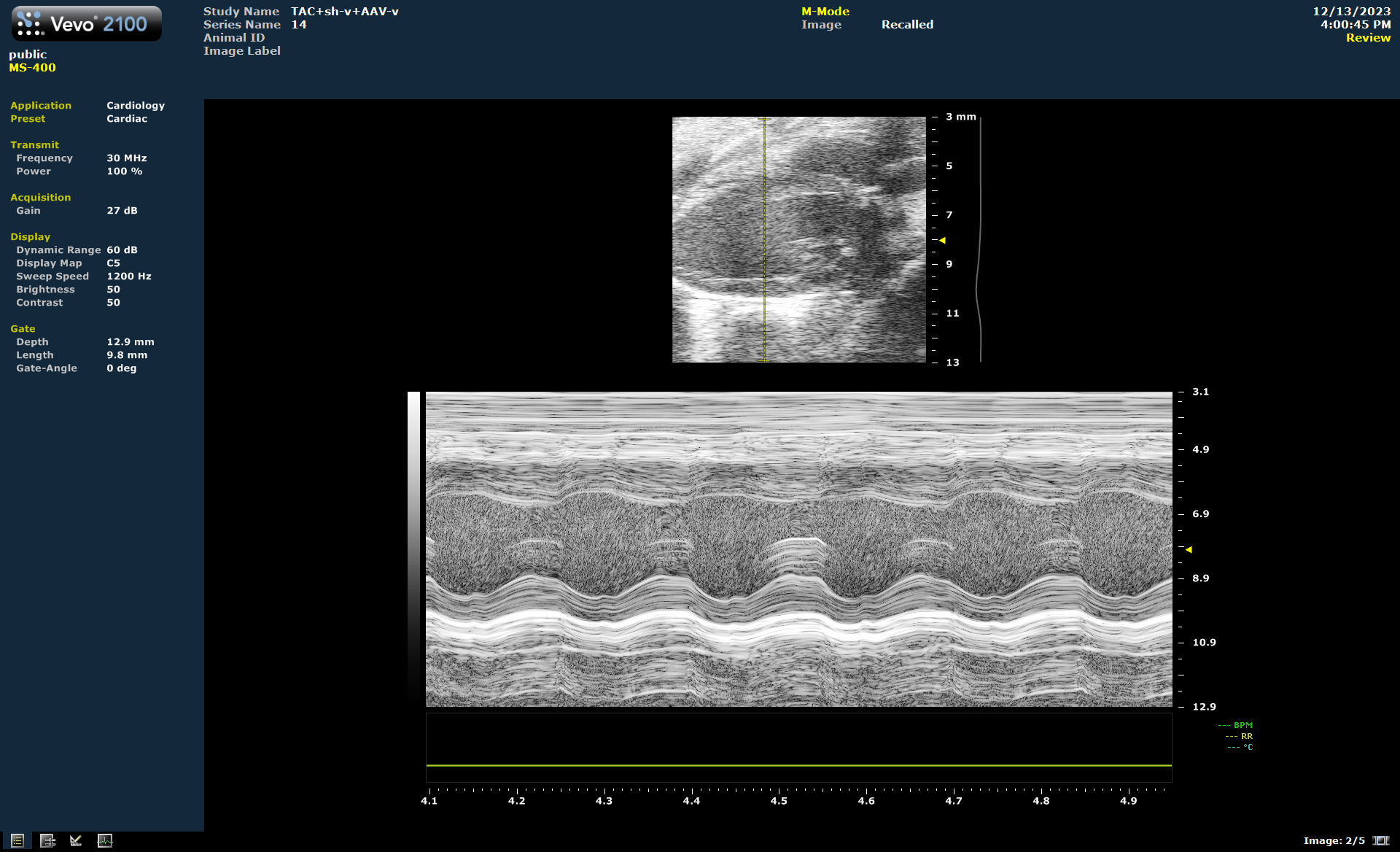

Supplement: Supplementary file 10 — Source data Fig. 7 [file 44321_2025_334_MOESM10_ESM.zip › Figure 7/7B/B Mode/TAC+AAV9-sh-Vector+AAV9-Vector.tif]

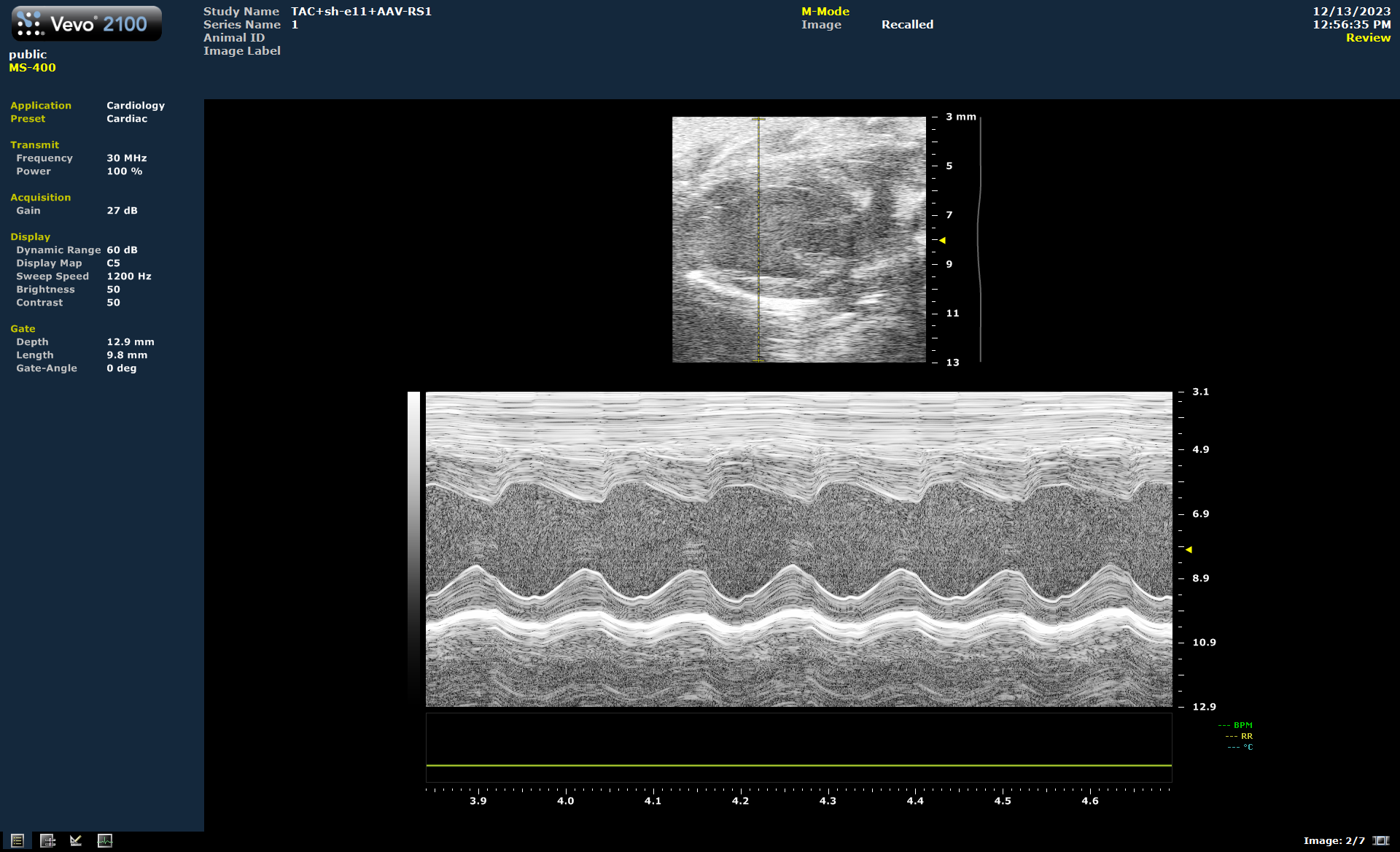

Supplement: Supplementary file 10 — Source data Fig. 7 [file 44321_2025_334_MOESM10_ESM.zip › Figure 7/7B/B Mode/TAC+AAV9-sh-Δe11+AAV9-RBMS1.tif]

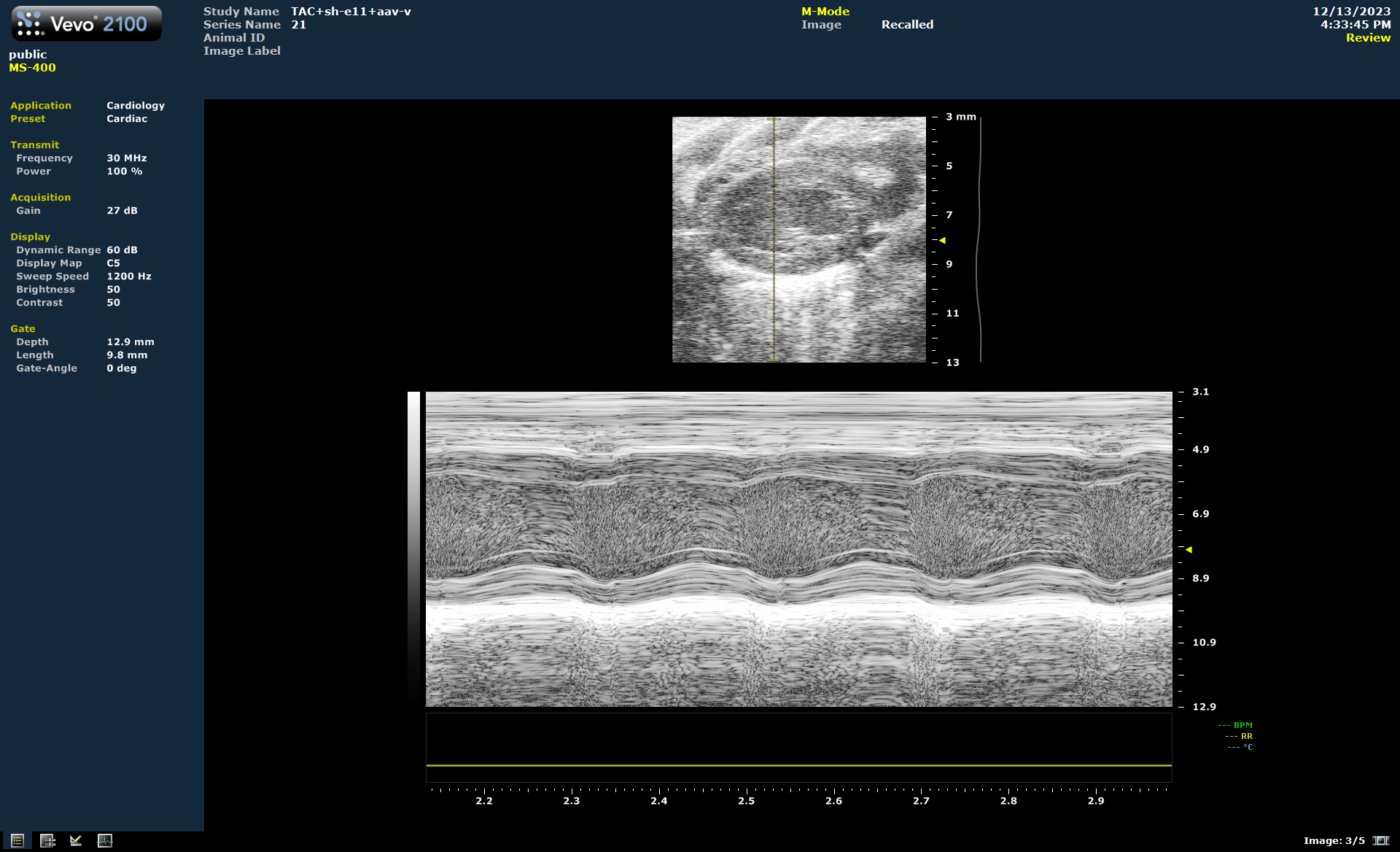

Supplement: Supplementary file 10 — Source data Fig. 7 [file 44321_2025_334_MOESM10_ESM.zip › Figure 7/7B/B Mode/TAC+AAV9-sh-Δe11+AAV9-Vector.tif]

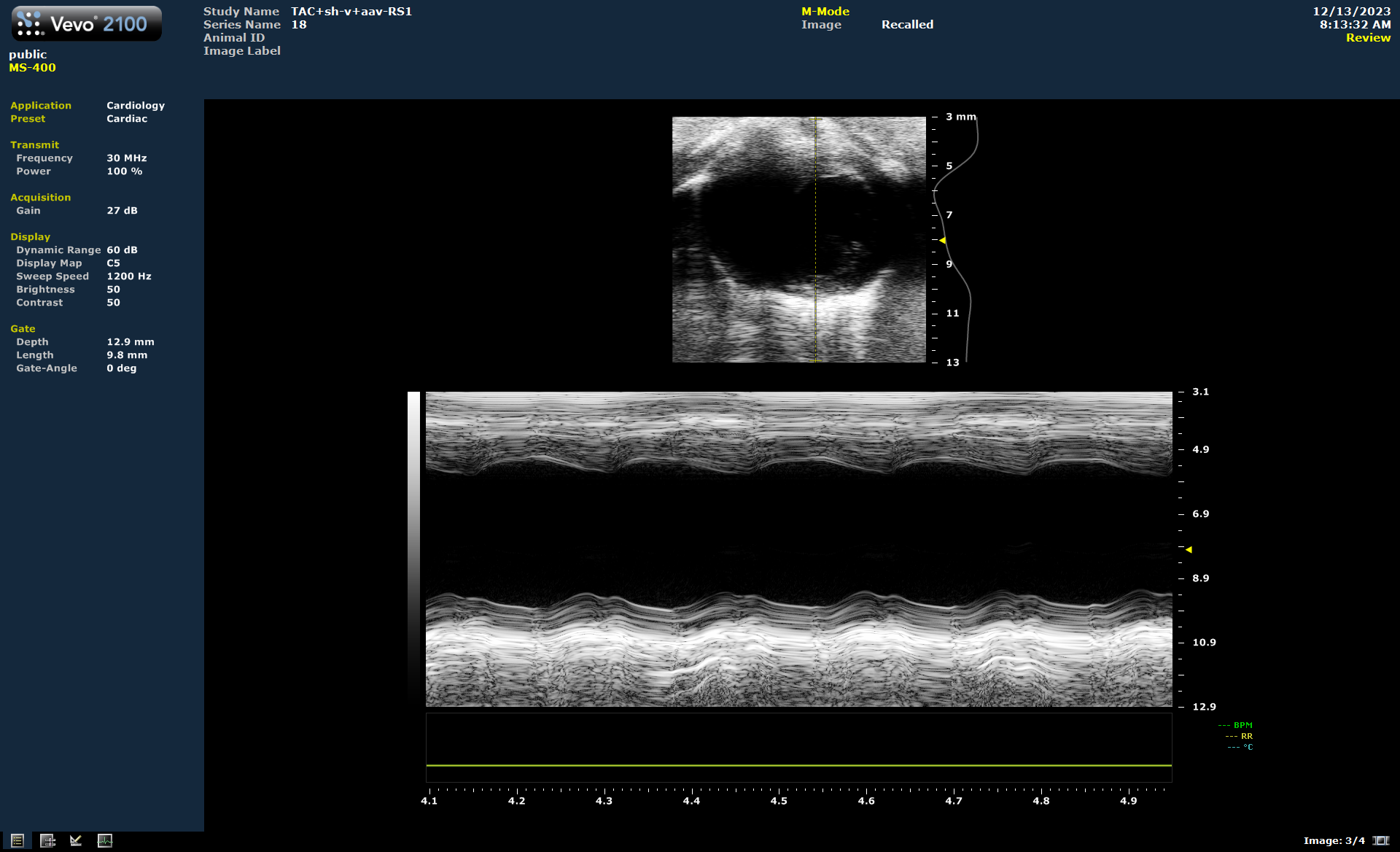

Supplement: Supplementary file 10 — Source data Fig. 7 [file 44321_2025_334_MOESM10_ESM.zip › Figure 7/7B/M Mode/TAC+AAV9-sh-Vector+AAV9-RBMS1.tif]

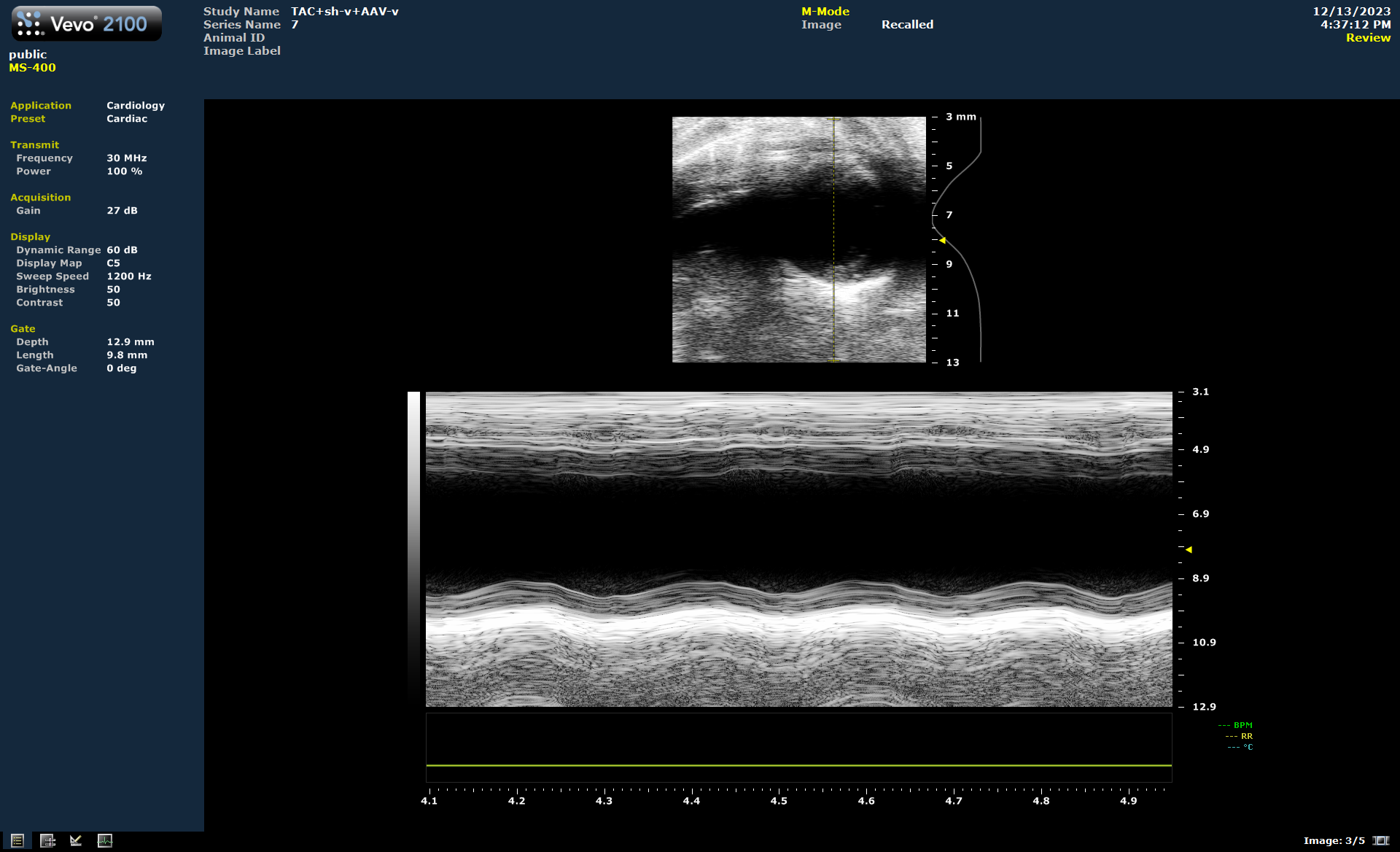

Supplement: Supplementary file 10 — Source data Fig. 7 [file 44321_2025_334_MOESM10_ESM.zip › Figure 7/7B/M Mode/TAC+AAV9-sh-Vector+AAV9-Vector.tif]

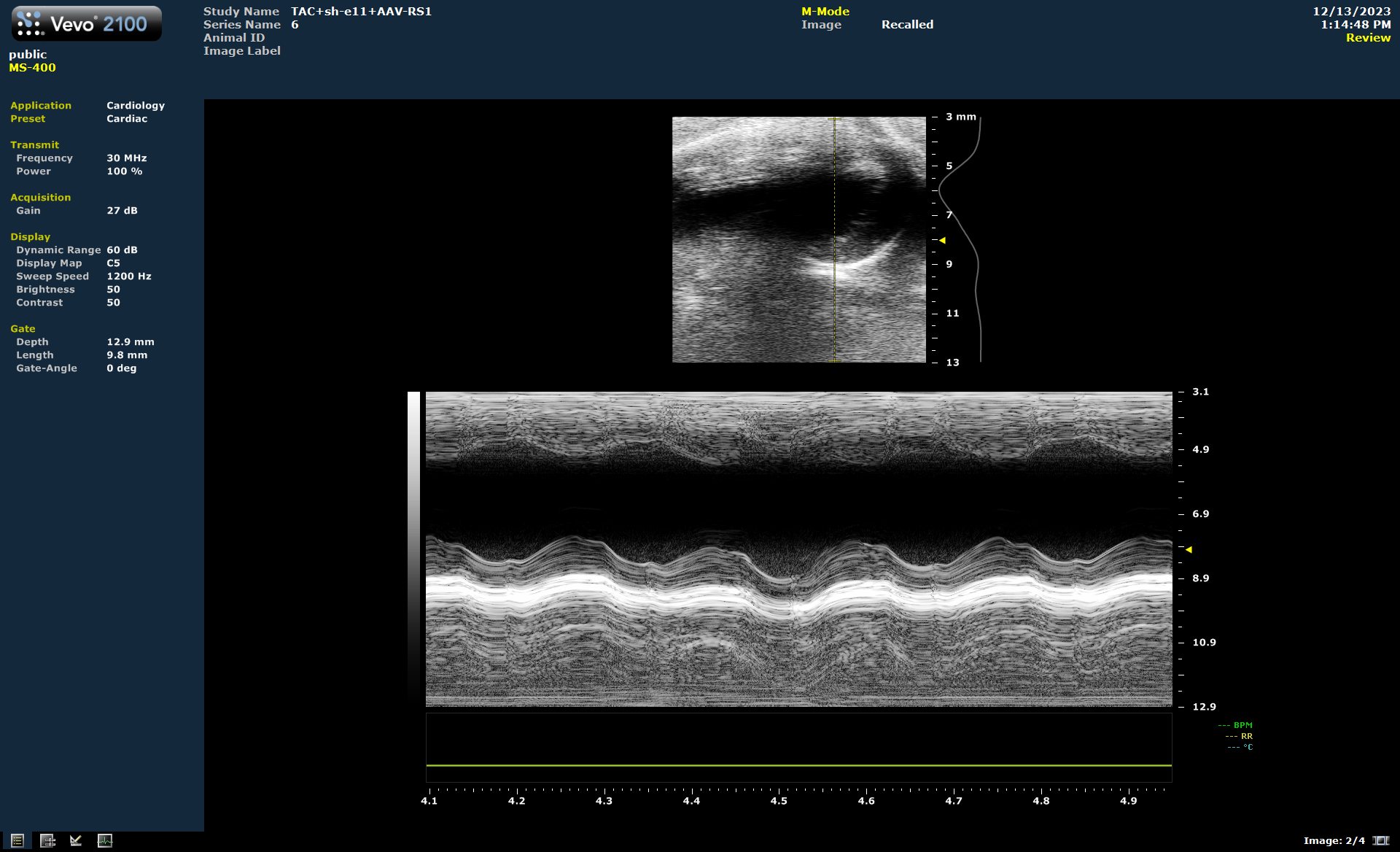

Supplement: Supplementary file 10 — Source data Fig. 7 [file 44321_2025_334_MOESM10_ESM.zip › Figure 7/7B/M Mode/TAC+AAV9-sh-Δe11+AAV9-RBMS1.tif]

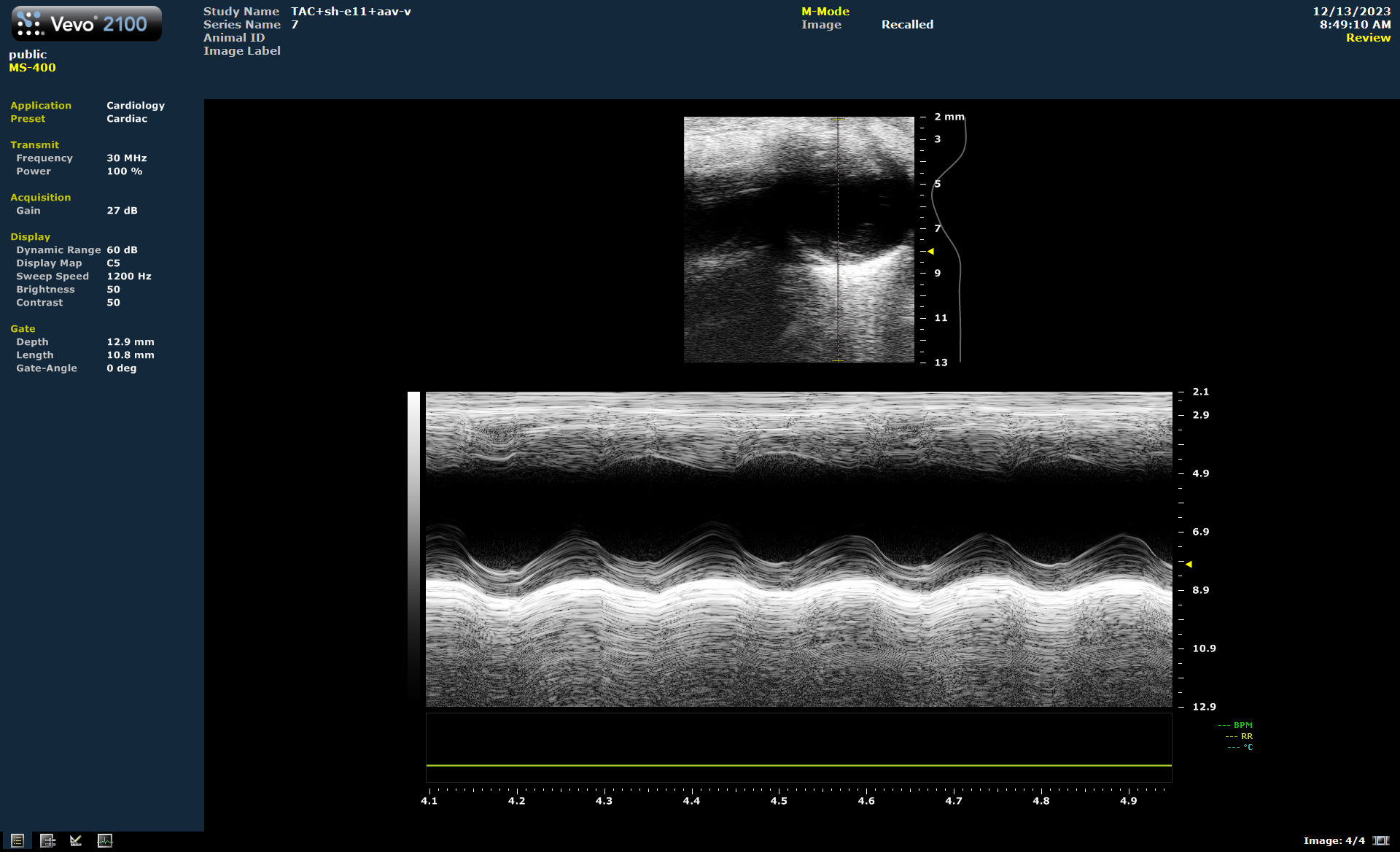

Supplement: Supplementary file 10 — Source data Fig. 7 [file 44321_2025_334_MOESM10_ESM.zip › Figure 7/7B/M Mode/TAC+AAV9-sh-Δe11+AAV9-Vector.tif]

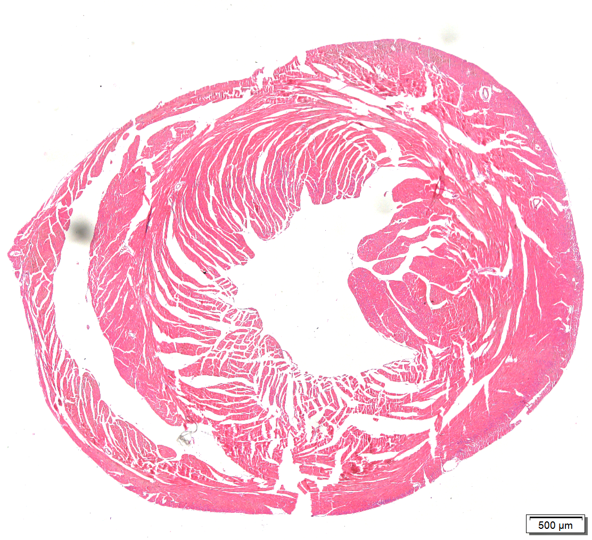

Supplement: Supplementary file 10 — Source data Fig. 7 [file 44321_2025_334_MOESM10_ESM.zip › Figure 7/7D/Cross/TAC+AAV9-sh-Vector+AAV9-RBMS1.tif]

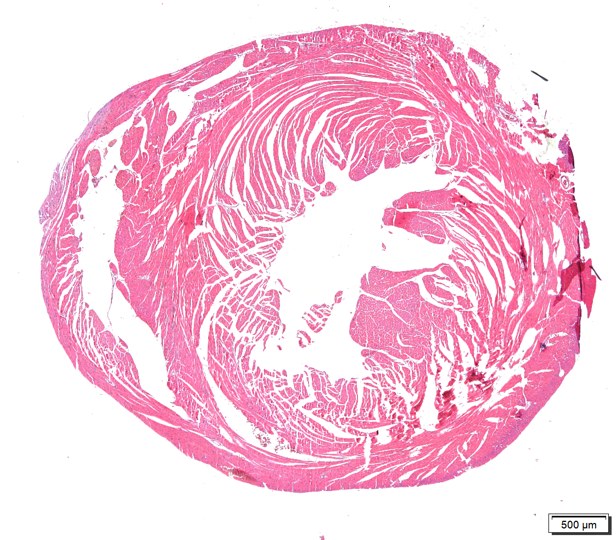

Supplement: Supplementary file 10 — Source data Fig. 7 [file 44321_2025_334_MOESM10_ESM.zip › Figure 7/7D/Cross/TAC+AAV9-sh-Vector+AAV9-Vector.tif]

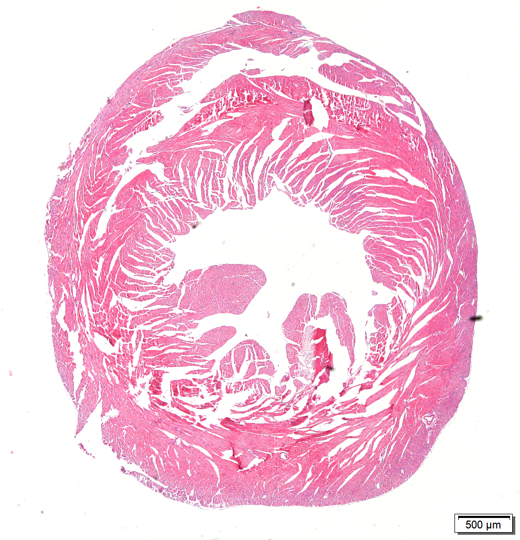

Supplement: Supplementary file 10 — Source data Fig. 7 [file 44321_2025_334_MOESM10_ESM.zip › Figure 7/7D/Cross/TAC+AAV9-sh-Δe11+AAV9-RBMS1.tif]

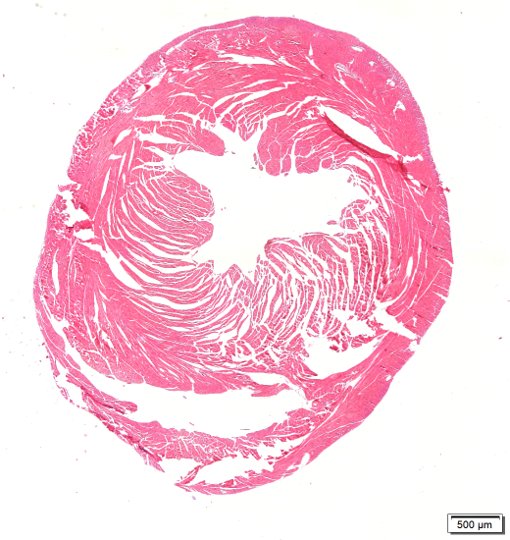

Supplement: Supplementary file 10 — Source data Fig. 7 [file 44321_2025_334_MOESM10_ESM.zip › Figure 7/7D/Cross/TAC+AAV9-sh-Δe11+AAV9-Vector.tif]

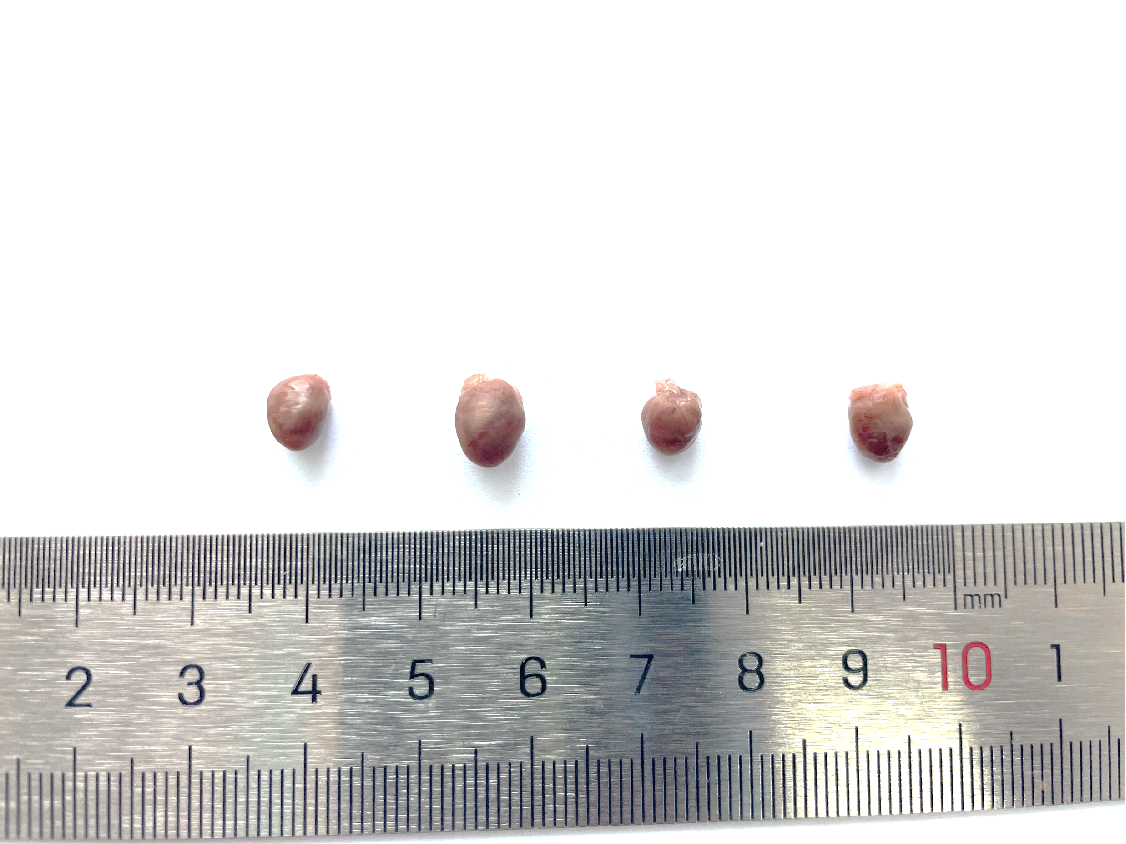

Supplement: Supplementary file 10 — Source data Fig. 7 [file 44321_2025_334_MOESM10_ESM.zip › Figure 7/7D/Heart size/7D.png]

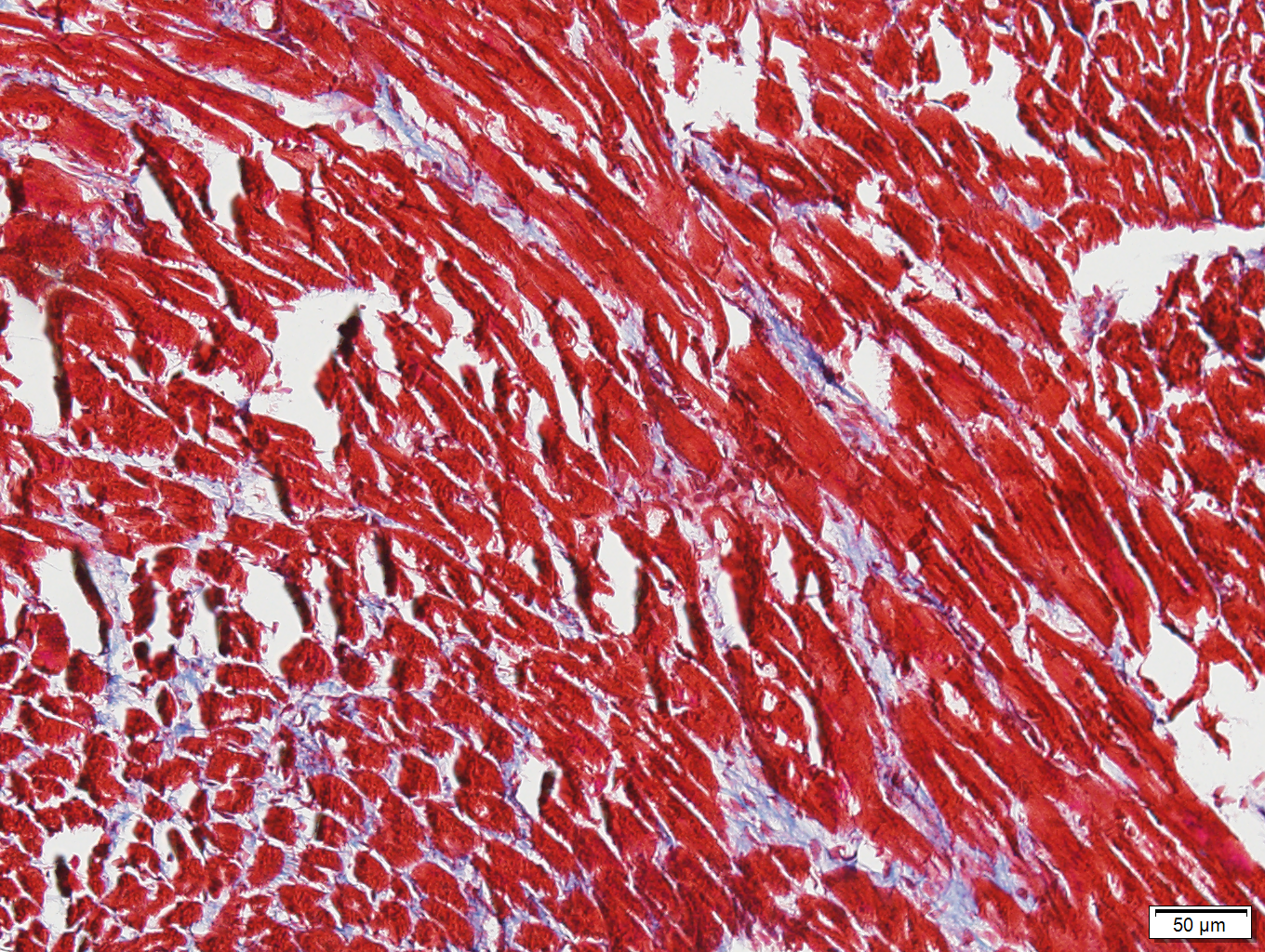

Supplement: Supplementary file 10 — Source data Fig. 7 [file 44321_2025_334_MOESM10_ESM.zip › Figure 7/7D/Interstital/TAC+AAV9-sh-Vector+AAV9-RBMS1.tif]

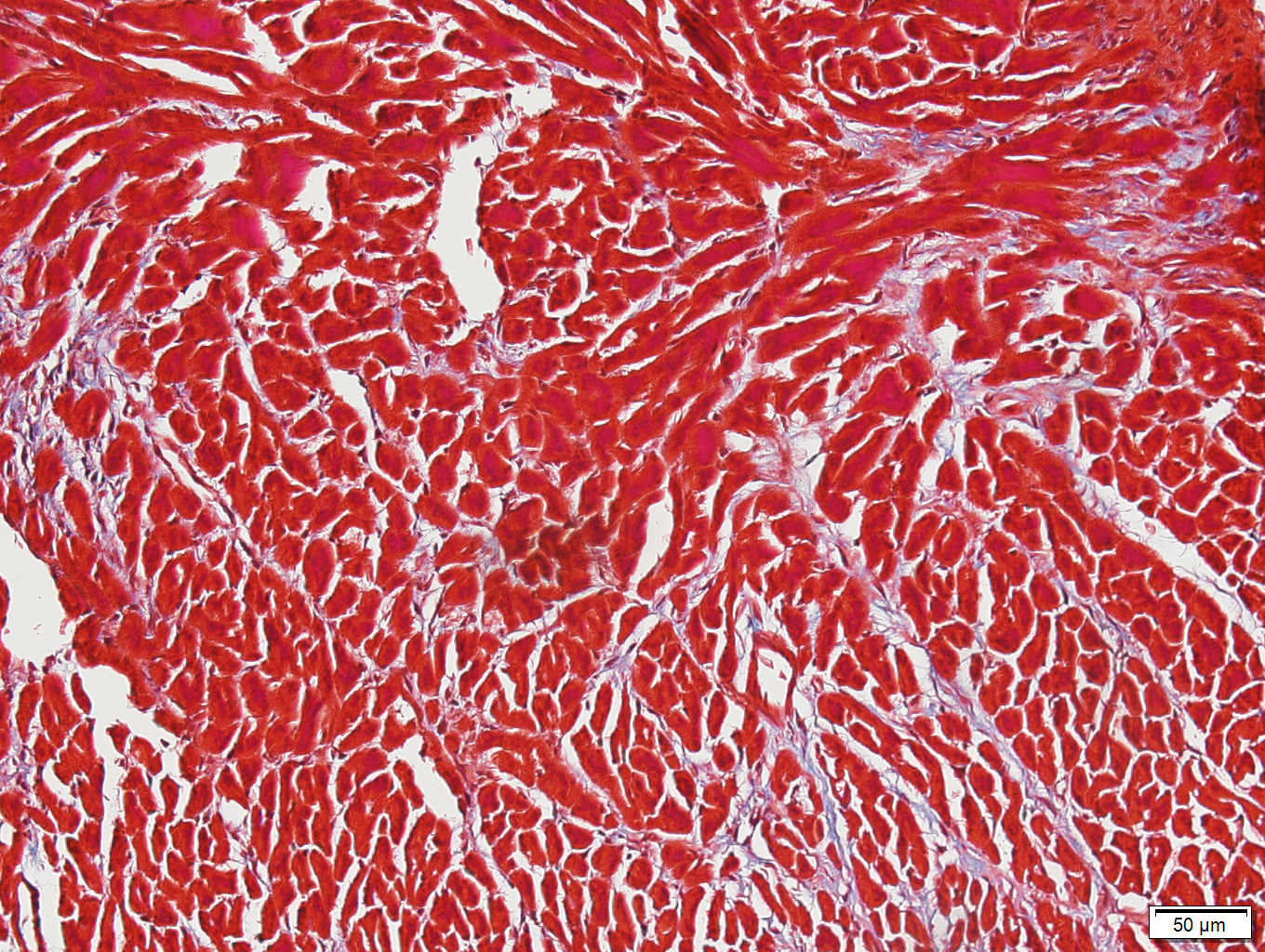

Supplement: Supplementary file 10 — Source data Fig. 7 [file 44321_2025_334_MOESM10_ESM.zip › Figure 7/7D/Interstital/TAC+AAV9-sh-Vector+AAV9-Vector.tif]

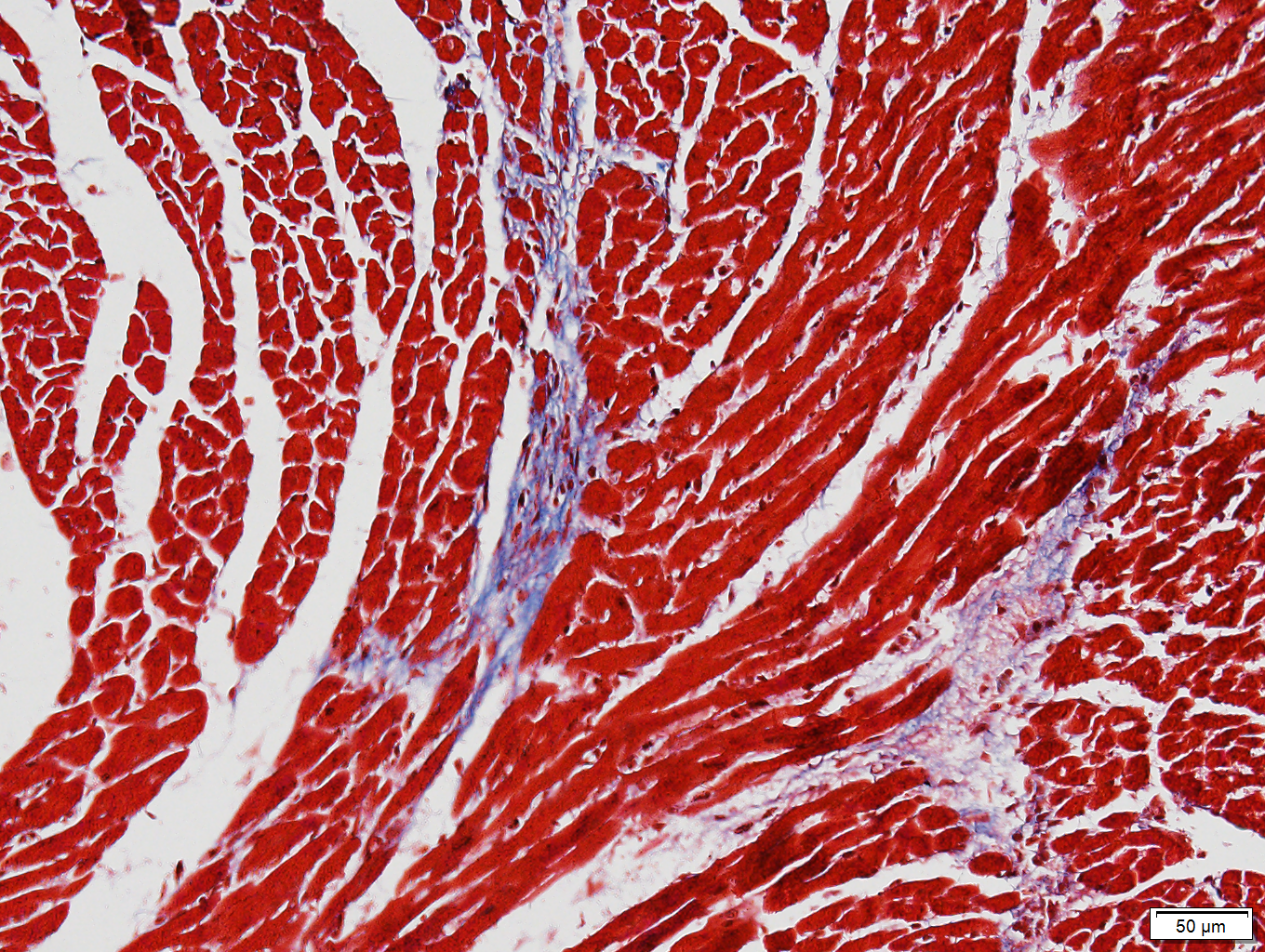

Supplement: Supplementary file 10 — Source data Fig. 7 [file 44321_2025_334_MOESM10_ESM.zip › Figure 7/7D/Interstital/TAC+AAV9-sh-Δe11+AAV9-RBMS1.tif]

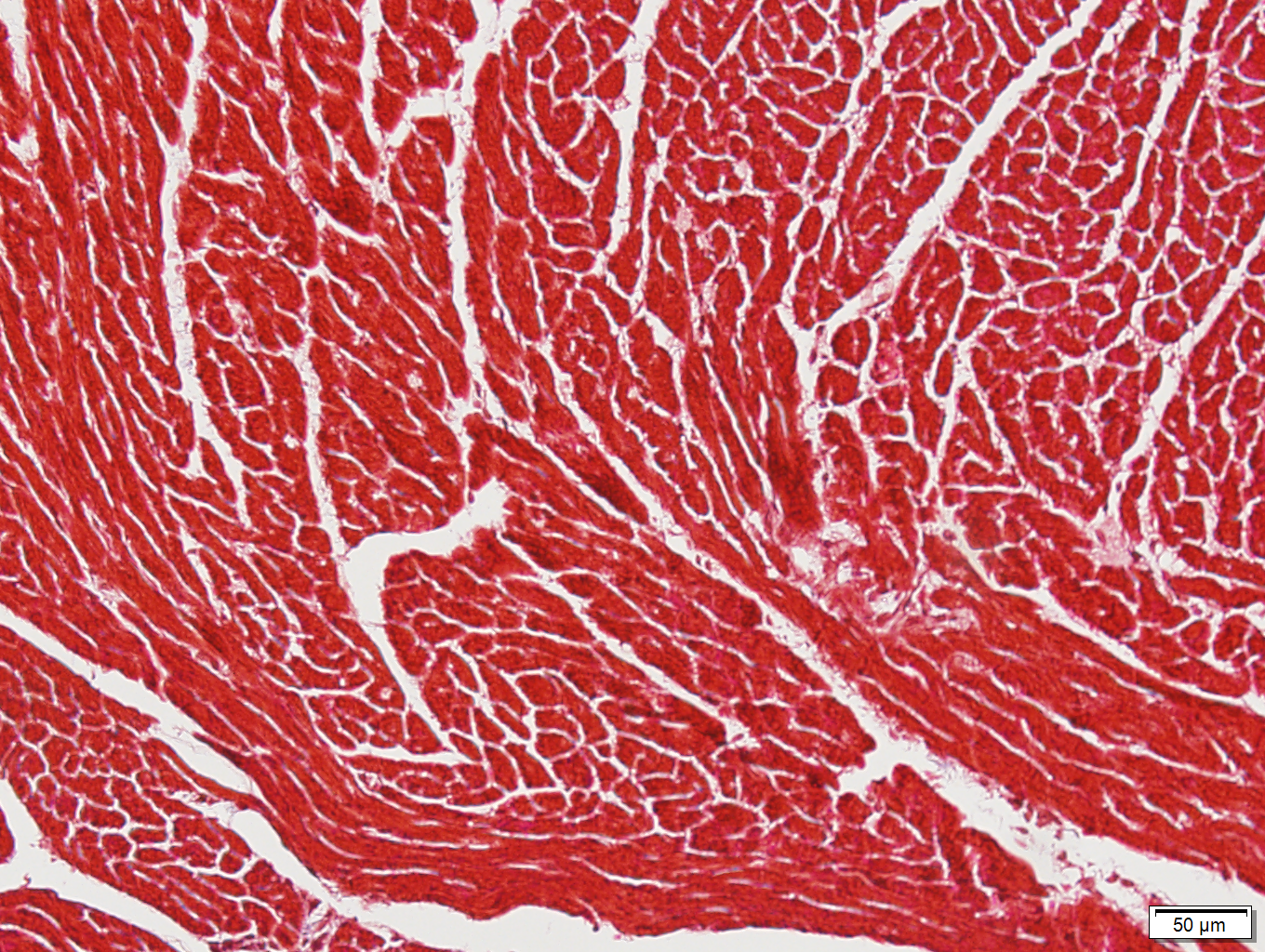

Supplement: Supplementary file 10 — Source data Fig. 7 [file 44321_2025_334_MOESM10_ESM.zip › Figure 7/7D/Interstital/TAC+AAV9-sh-Δe11+AAV9-Vector.tif]

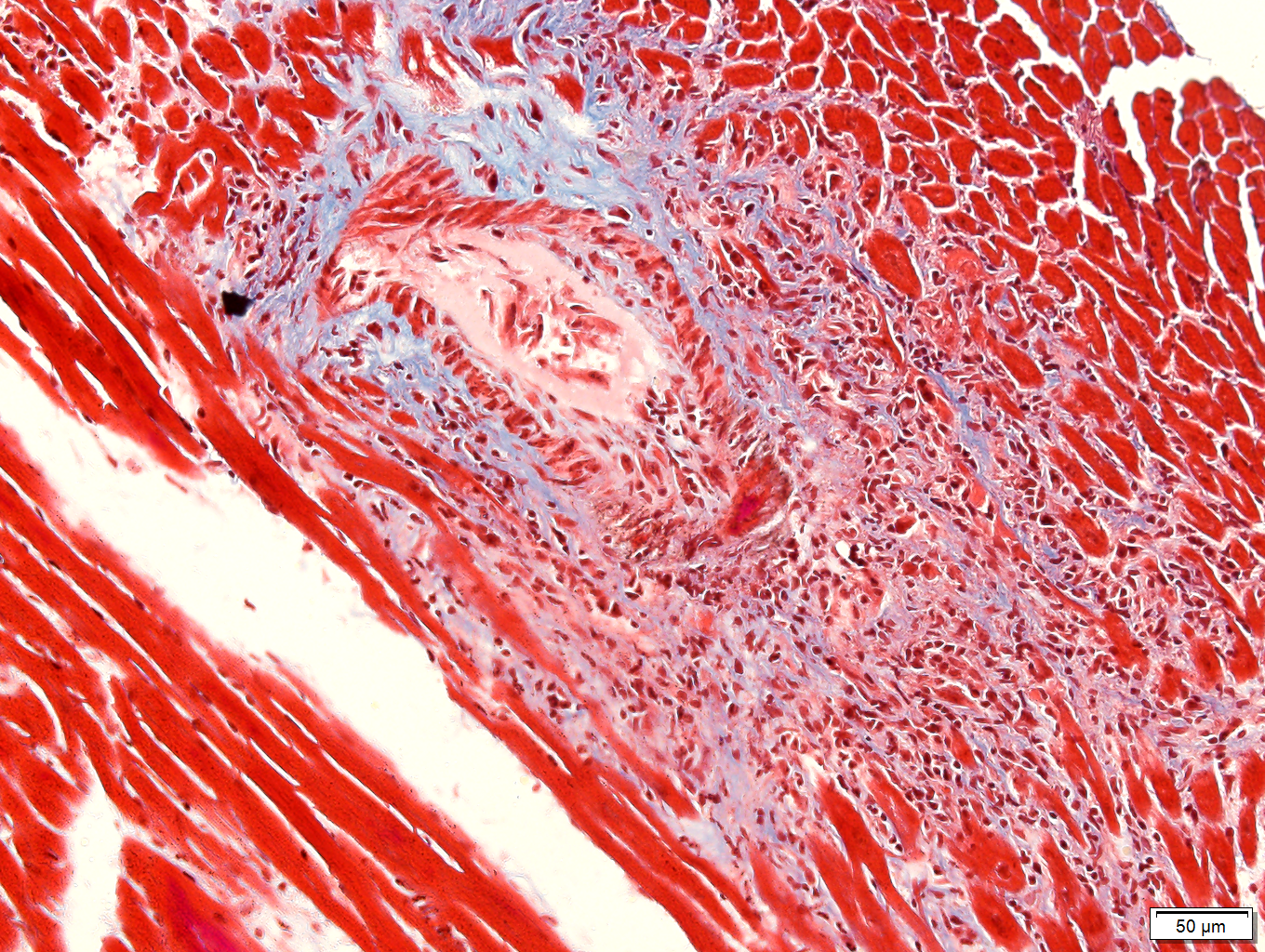

Supplement: Supplementary file 10 — Source data Fig. 7 [file 44321_2025_334_MOESM10_ESM.zip › Figure 7/7D/Perivascular/TAC+AAV9-sh-Vector+AAV9-RBMS1.tif]

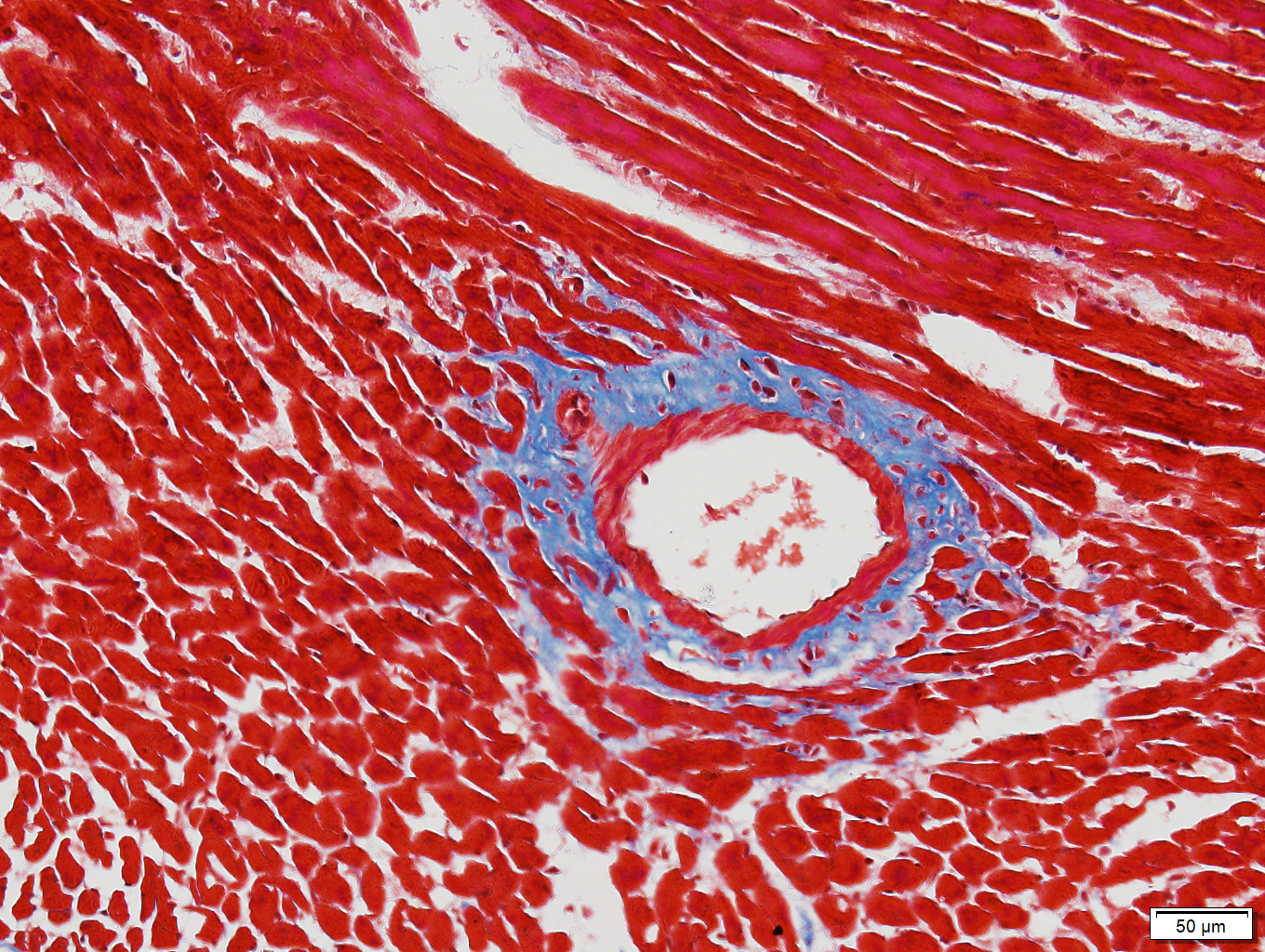

Supplement: Supplementary file 10 — Source data Fig. 7 [file 44321_2025_334_MOESM10_ESM.zip › Figure 7/7D/Perivascular/TAC+AAV9-sh-Vector+AAV9-Vector.tif]

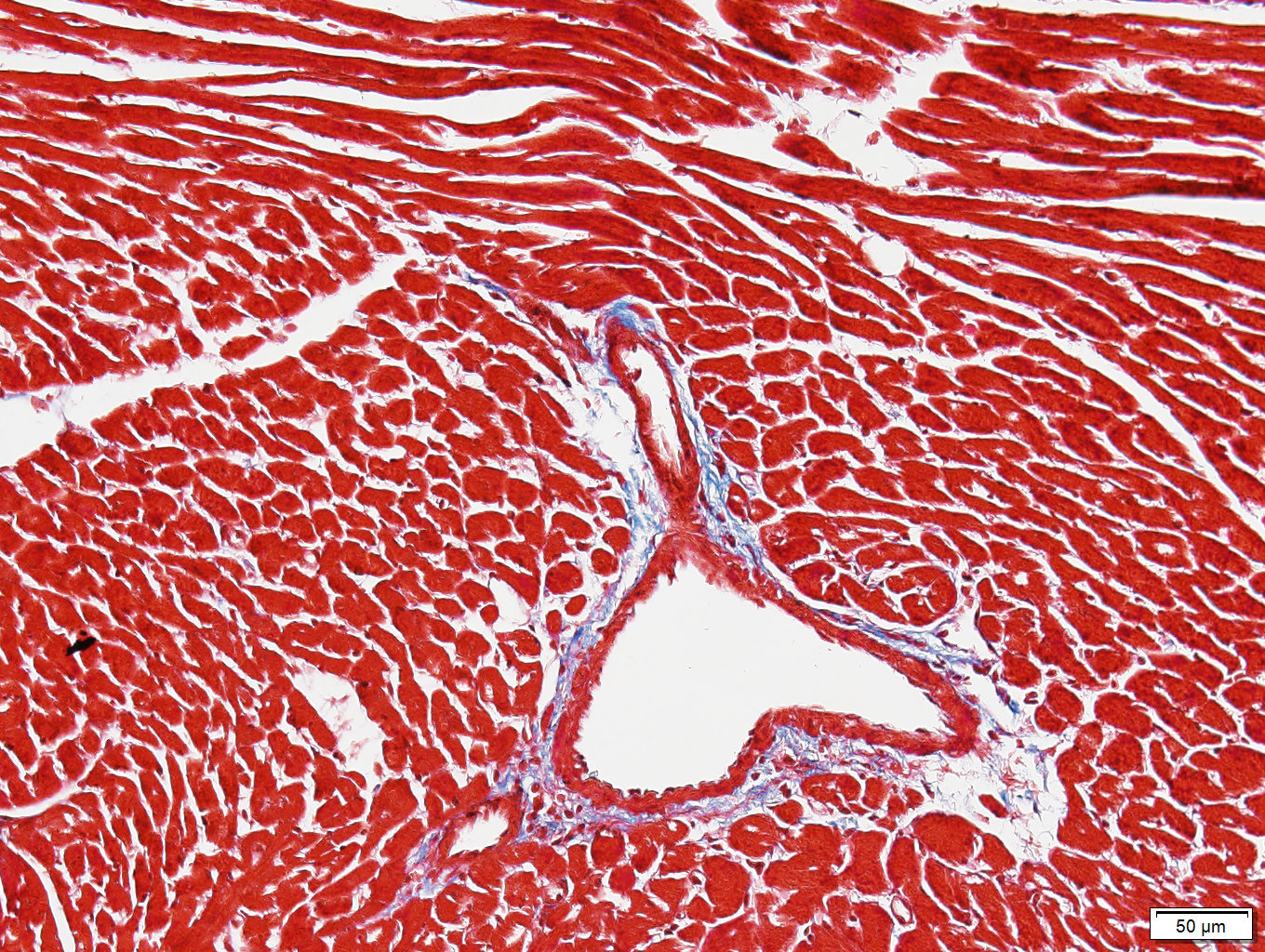

Supplement: Supplementary file 10 — Source data Fig. 7 [file 44321_2025_334_MOESM10_ESM.zip › Figure 7/7D/Perivascular/TAC+AAV9-sh-Δe11+AAV9-RBMS1.tif]

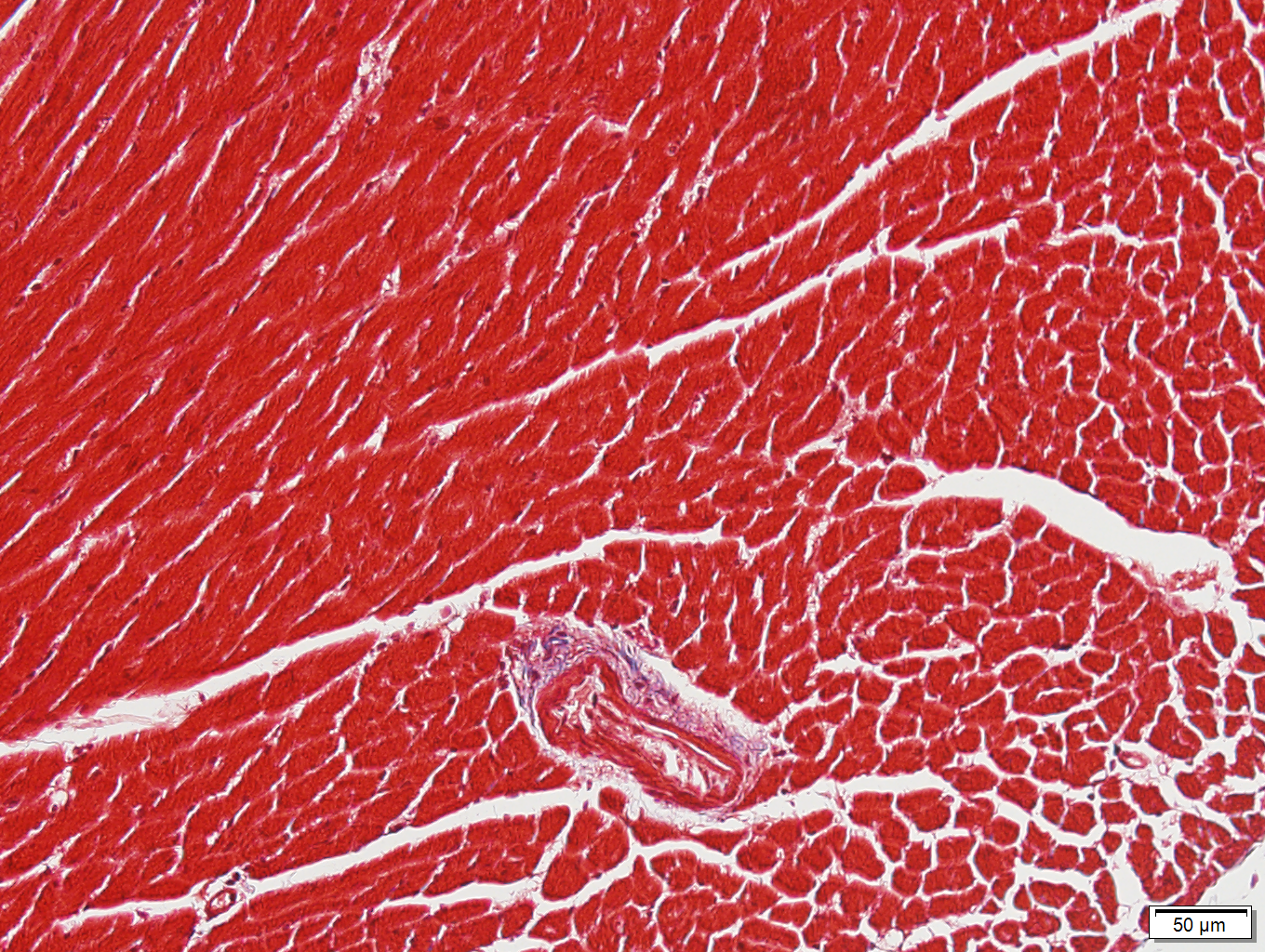

Supplement: Supplementary file 10 — Source data Fig. 7 [file 44321_2025_334_MOESM10_ESM.zip › Figure 7/7D/Perivascular/TAC+AAV9-sh-Δe11+AAV9-Vector.tif]

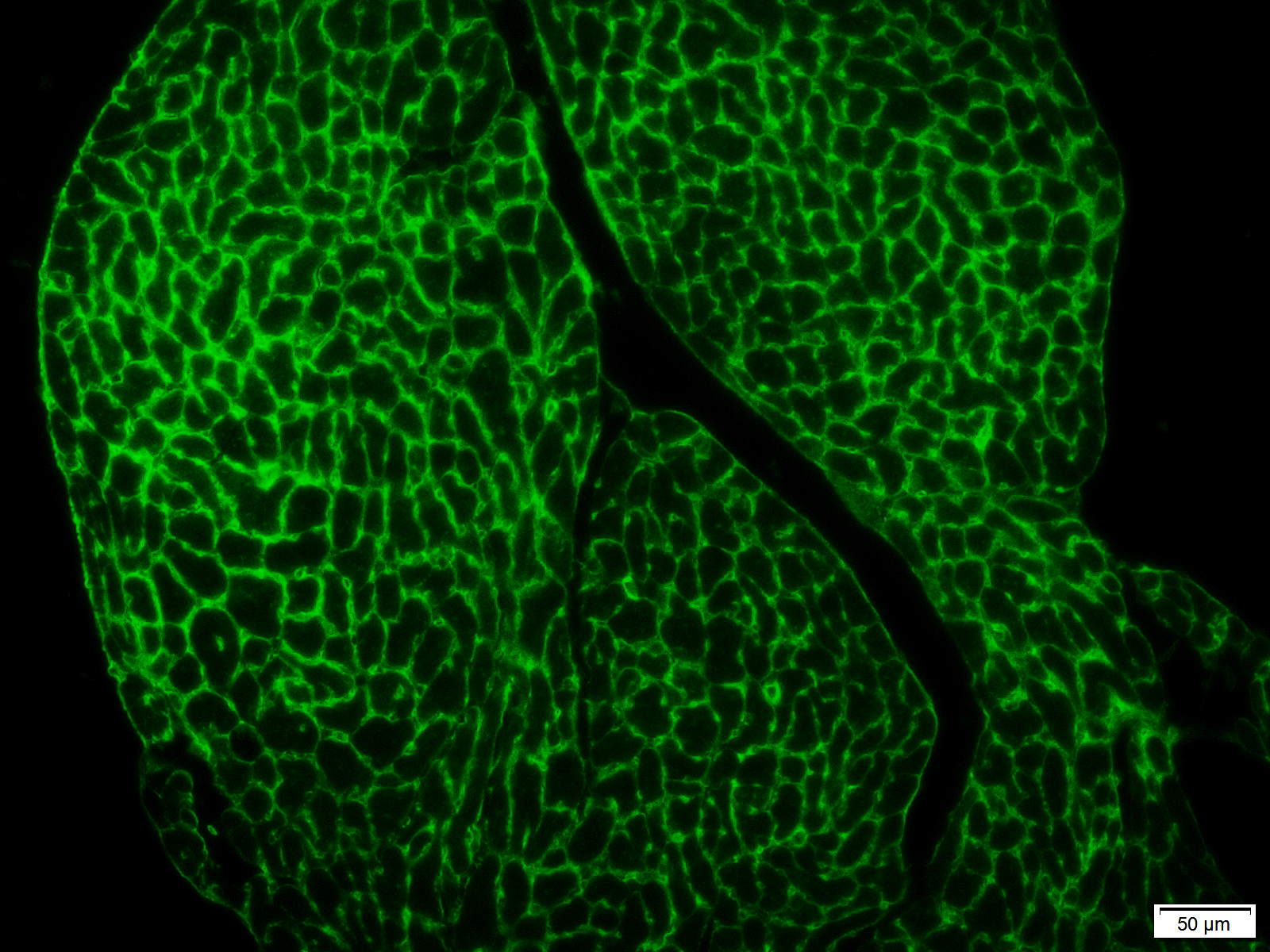

Supplement: Supplementary file 10 — Source data Fig. 7 [file 44321_2025_334_MOESM10_ESM.zip › Figure 7/7D/WGA/TAC+AAV9-sh-Vector+AAV9-RBMS1.tif]

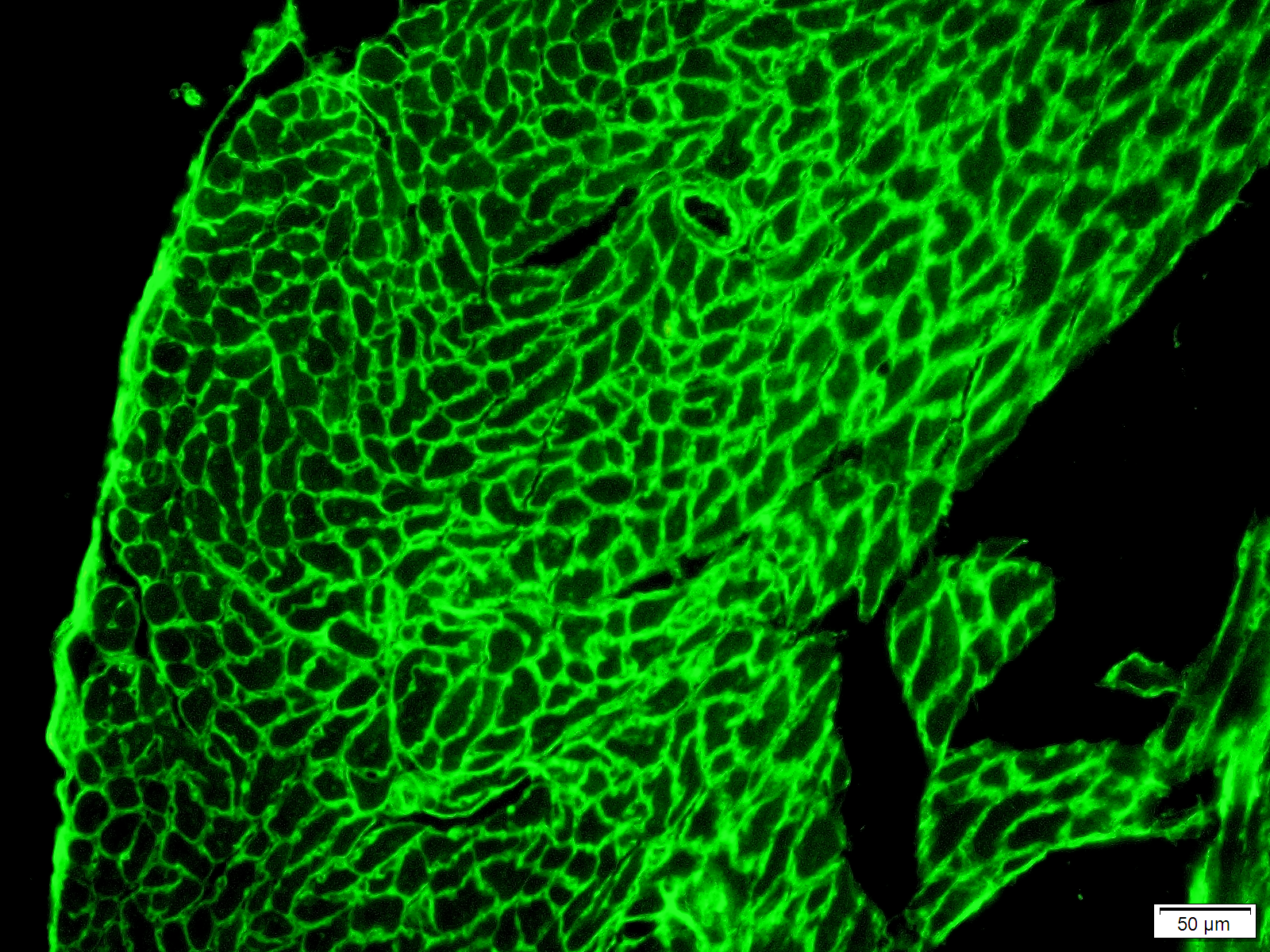

Supplement: Supplementary file 10 — Source data Fig. 7 [file 44321_2025_334_MOESM10_ESM.zip › Figure 7/7D/WGA/TAC+AAV9-sh-Vector+AAV9-Vector.tif]

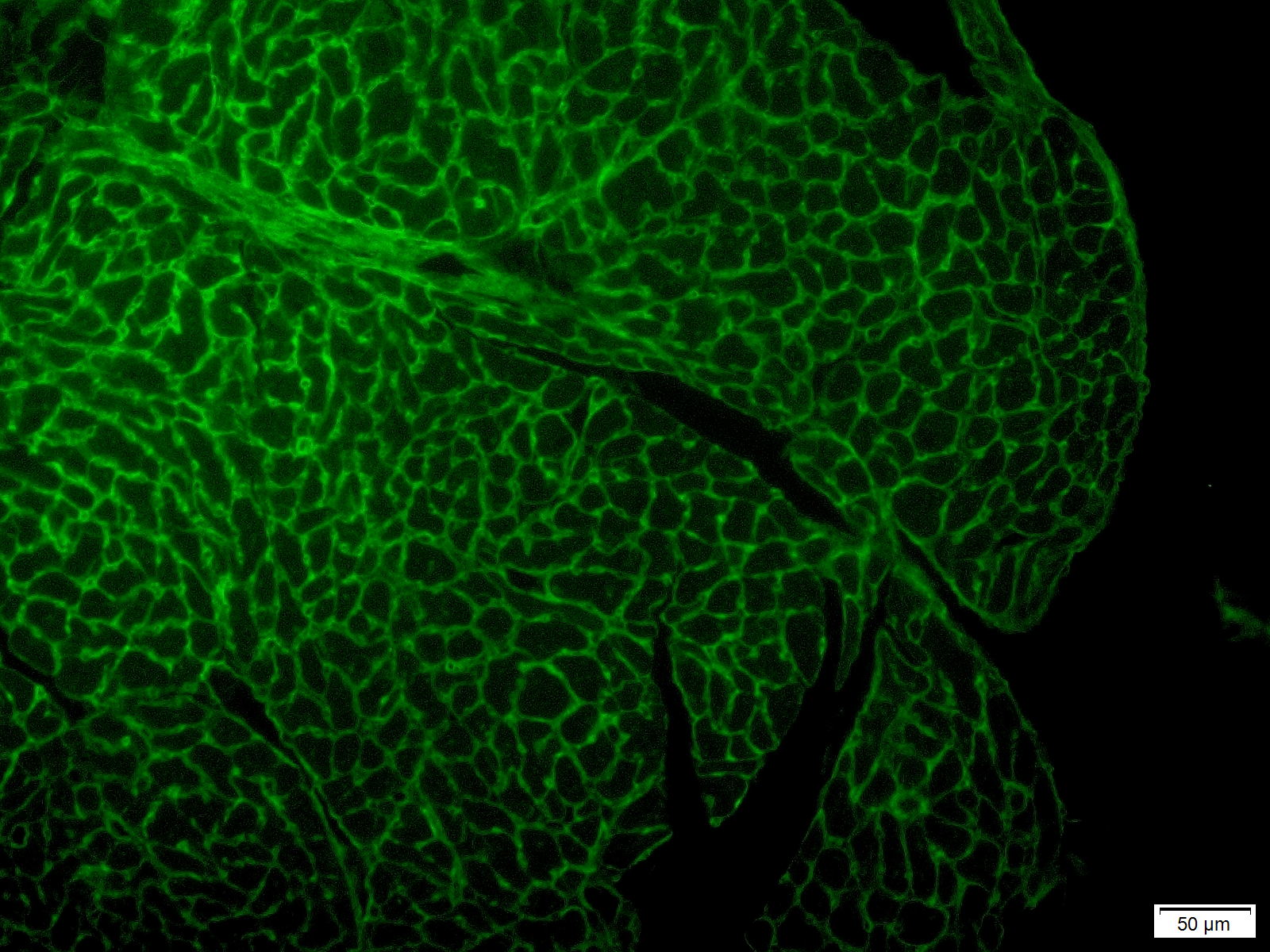

Supplement: Supplementary file 10 — Source data Fig. 7 [file 44321_2025_334_MOESM10_ESM.zip › Figure 7/7D/WGA/TAC+AAV9-sh-Δe11+AAV9-RBMS1.tif]

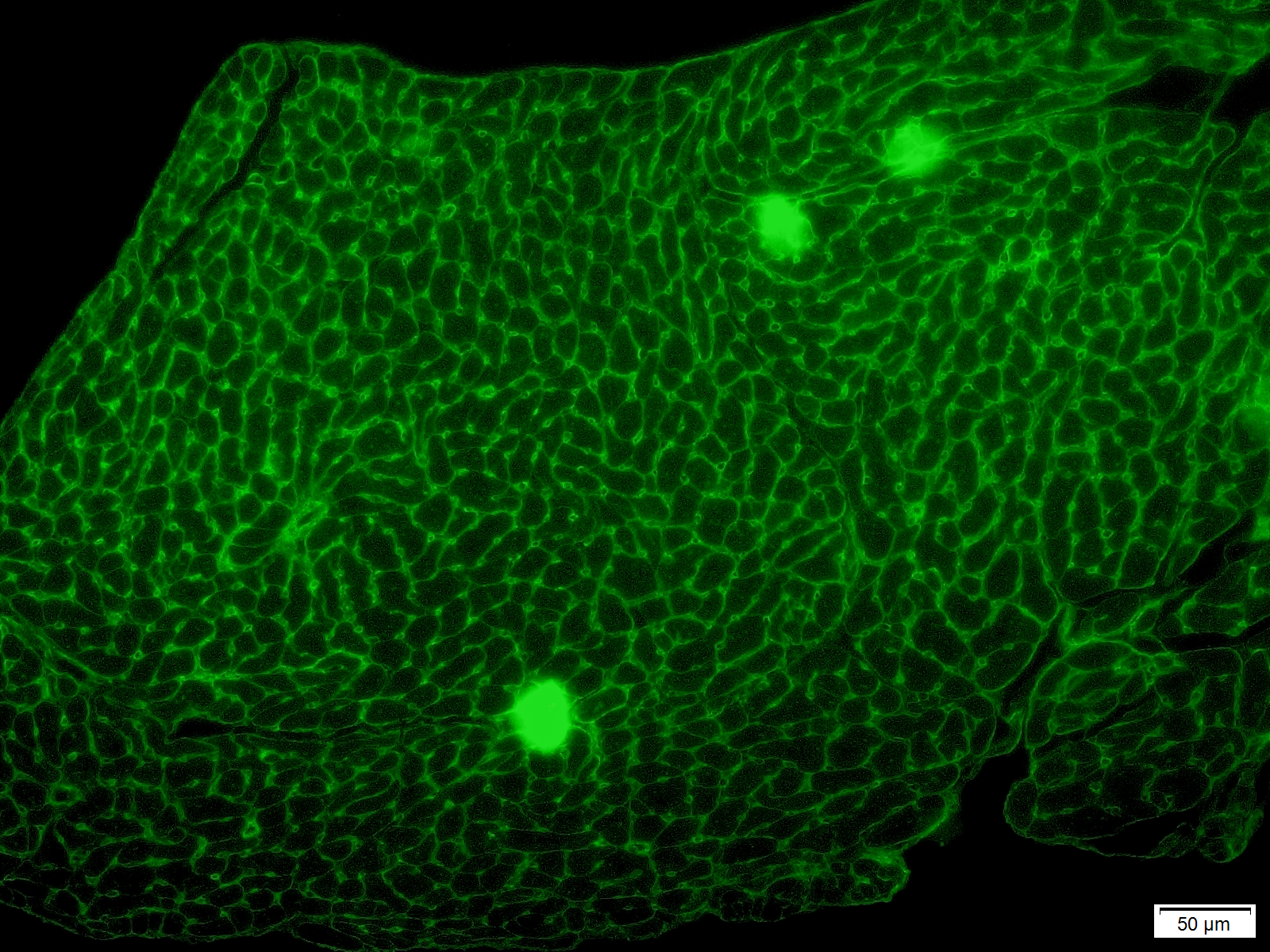

Supplement: Supplementary file 10 — Source data Fig. 7 [file 44321_2025_334_MOESM10_ESM.zip › Figure 7/7D/WGA/TAC+AAV9-sh-Δe11+AAV9-Vector.tif]

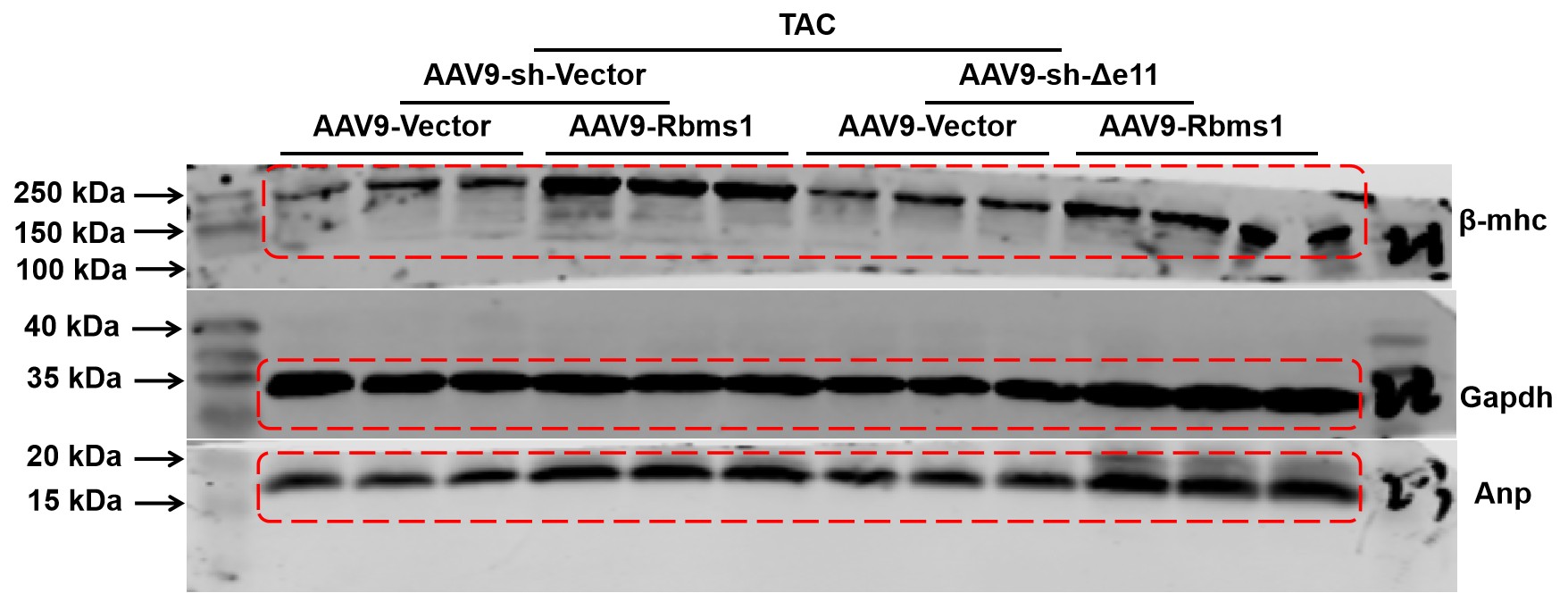

Supplement: Supplementary file 10 — Source data Fig. 7 [file 44321_2025_334_MOESM10_ESM.zip › Figure 7/7H/7H.jpg]

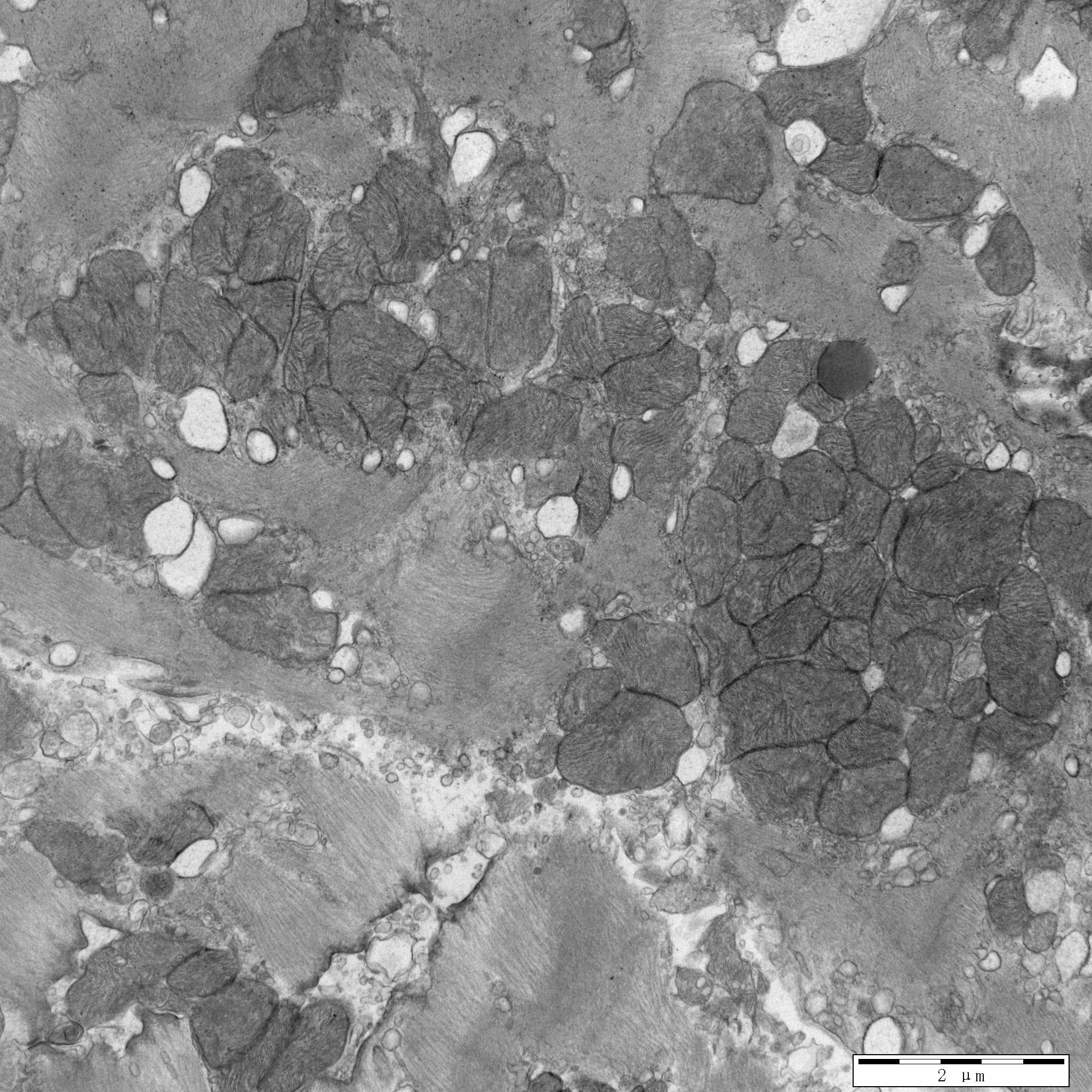

Supplement: Supplementary file 10 — Source data Fig. 7 [file 44321_2025_334_MOESM10_ESM.zip › Figure 7/7J/TAC+AAV9-sh-Vector+AAV9-RBMS1-1.JPG]

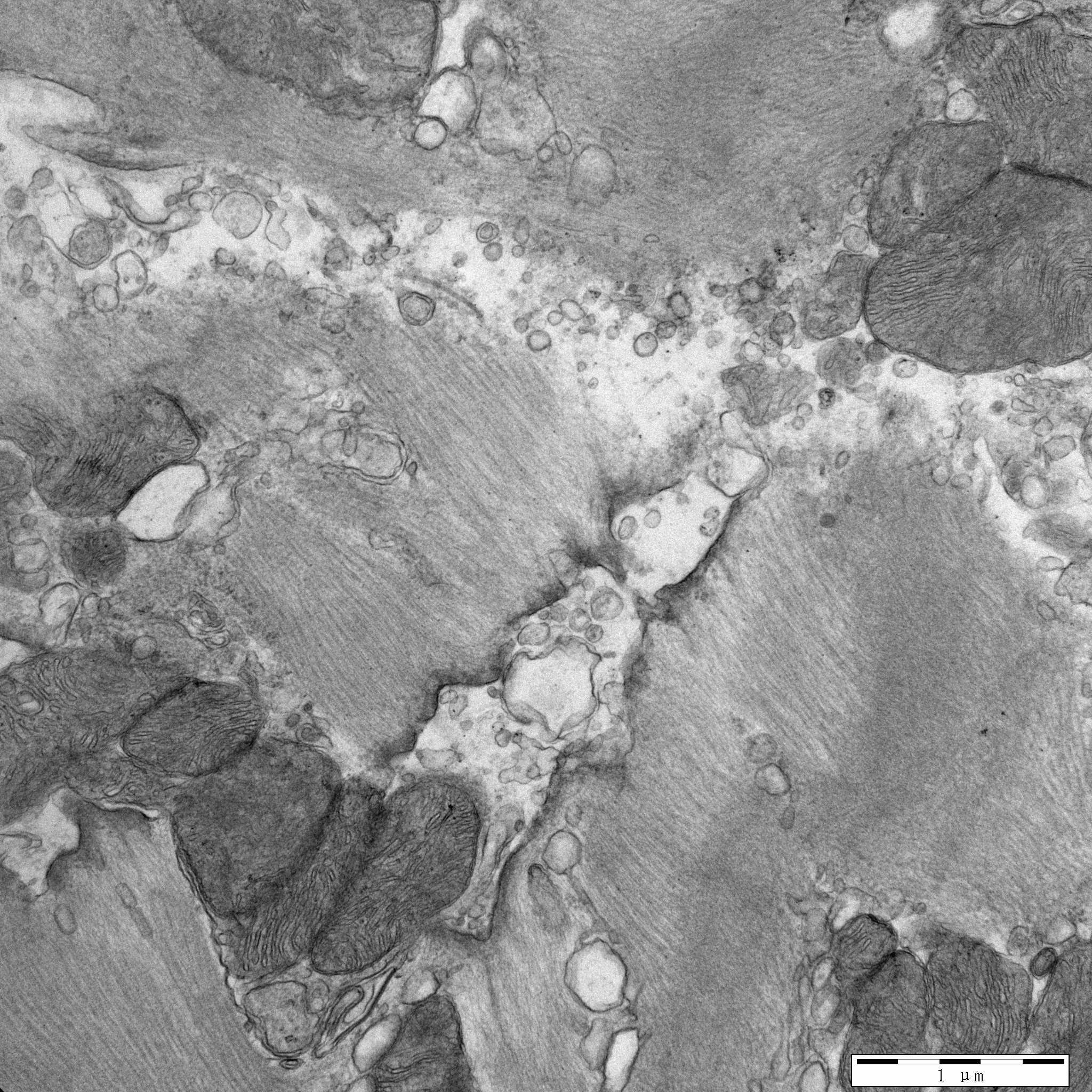

Supplement: Supplementary file 10 — Source data Fig. 7 [file 44321_2025_334_MOESM10_ESM.zip › Figure 7/7J/TAC+AAV9-sh-Vector+AAV9-RBMS1-2.JPG]

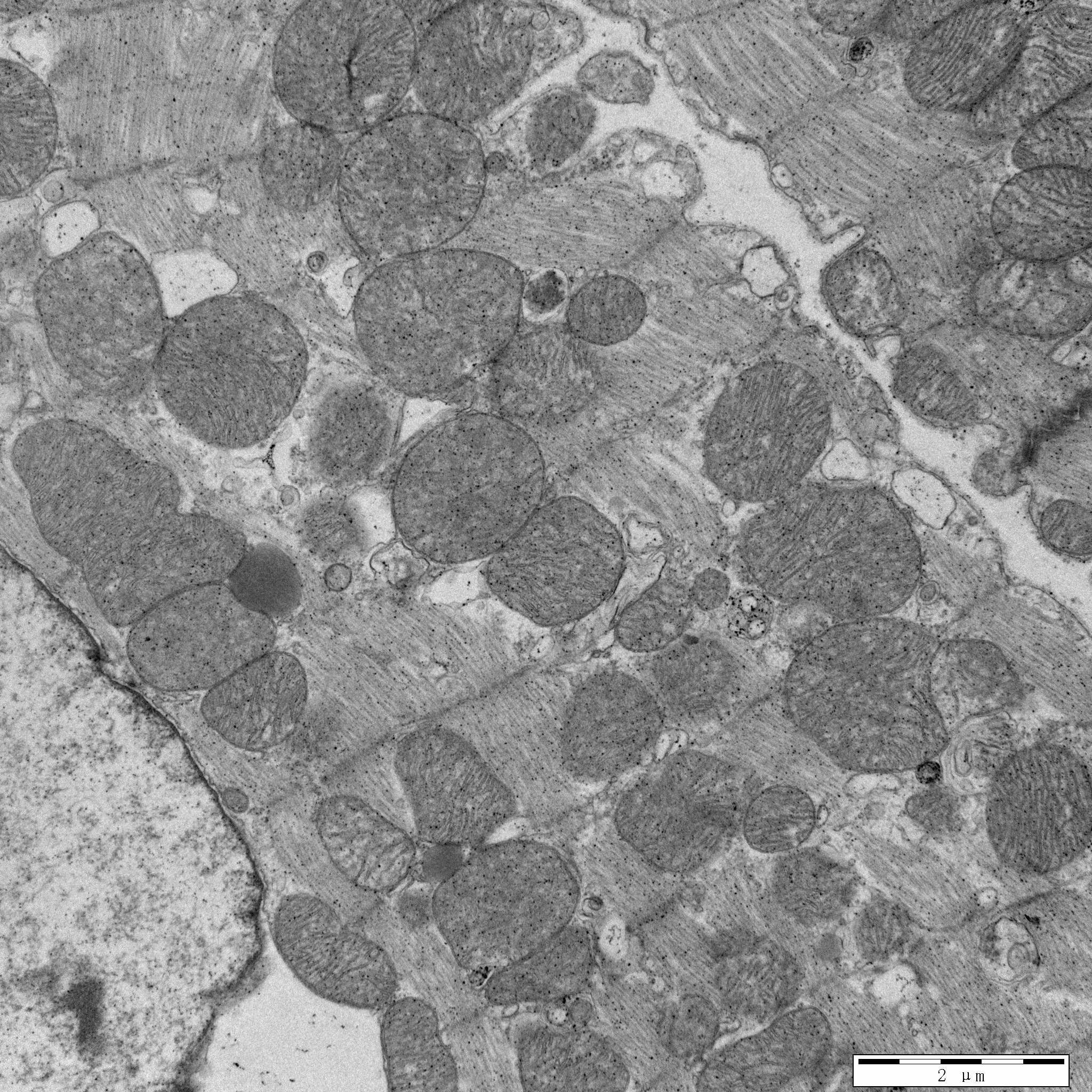

Supplement: Supplementary file 10 — Source data Fig. 7 [file 44321_2025_334_MOESM10_ESM.zip › Figure 7/7J/TAC+AAV9-sh-Vector+AAV9-Vector-1.JPG]

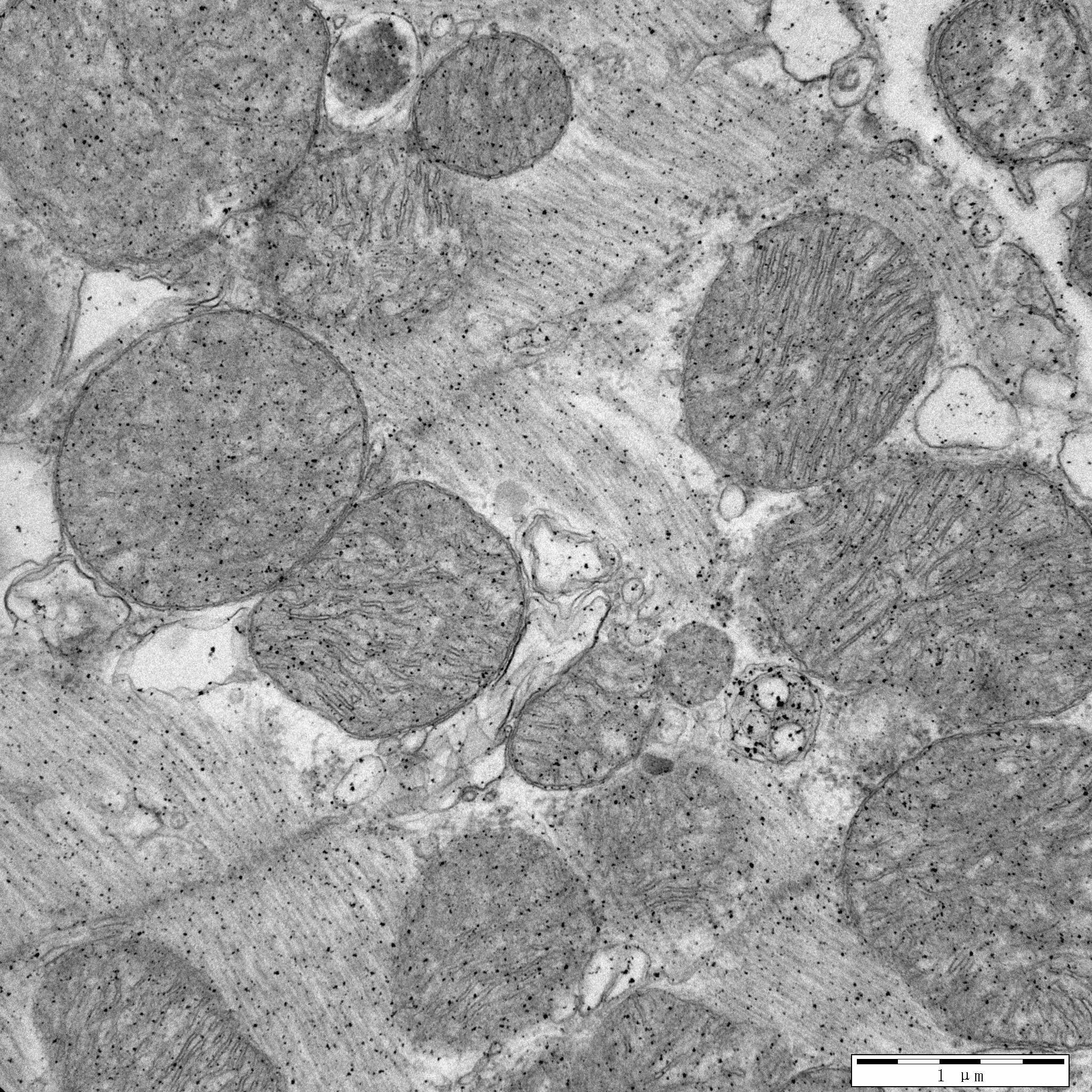

Supplement: Supplementary file 10 — Source data Fig. 7 [file 44321_2025_334_MOESM10_ESM.zip › Figure 7/7J/TAC+AAV9-sh-Vector+AAV9-Vector-2.JPG]

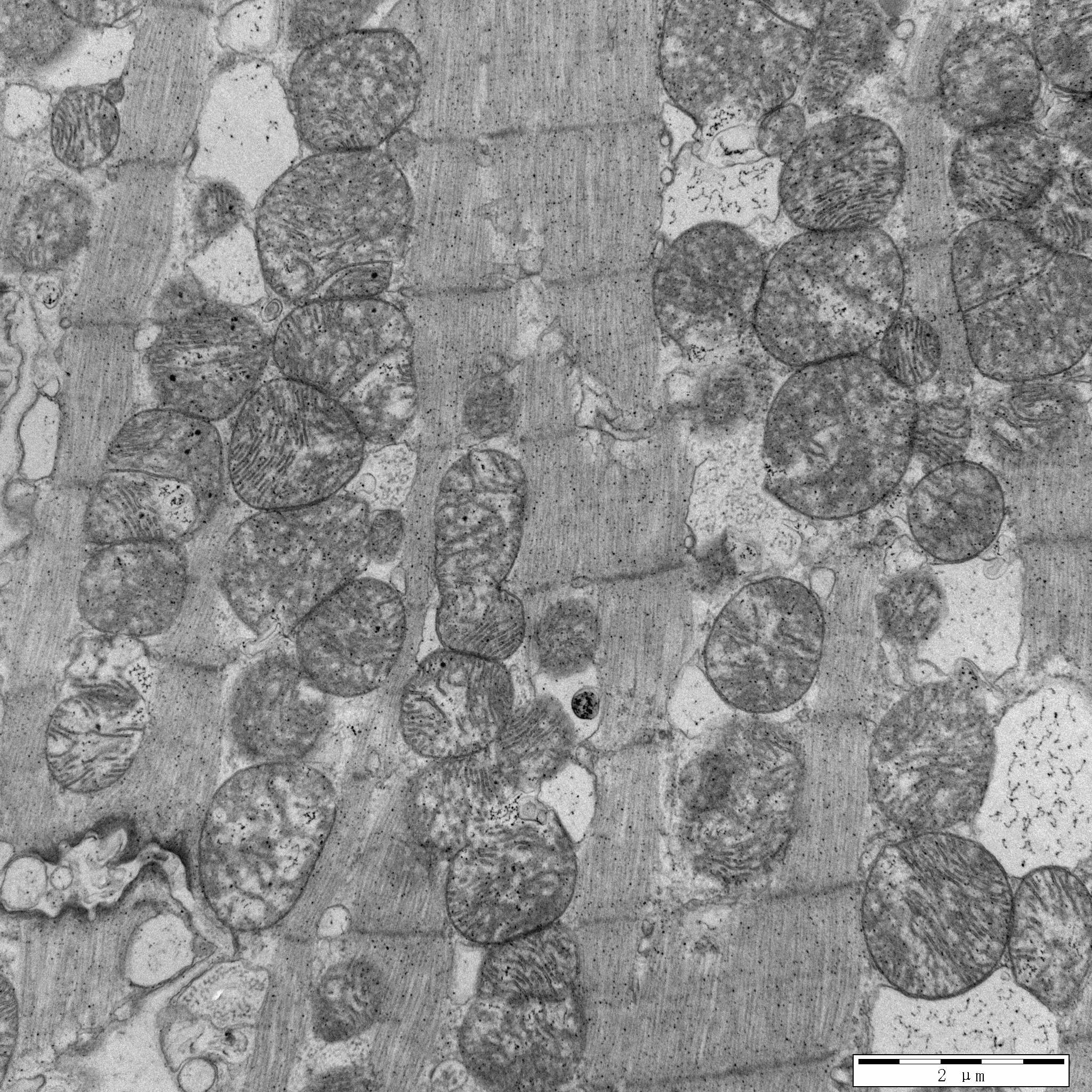

Supplement: Supplementary file 10 — Source data Fig. 7 [file 44321_2025_334_MOESM10_ESM.zip › Figure 7/7J/TAC+AAV9-sh-Δe11+AAV9-RBMS1-1.JPG]

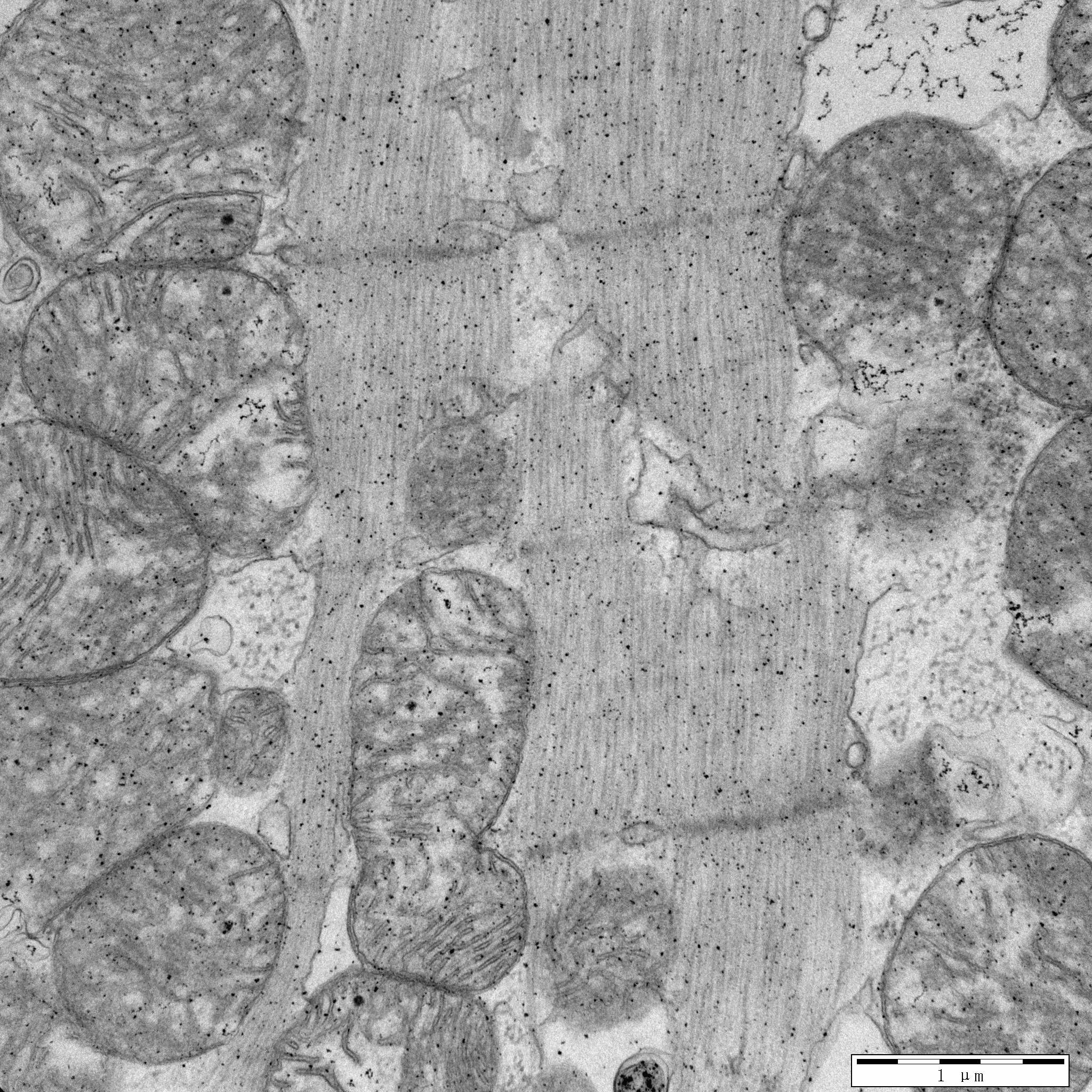

Supplement: Supplementary file 10 — Source data Fig. 7 [file 44321_2025_334_MOESM10_ESM.zip › Figure 7/7J/TAC+AAV9-sh-Δe11+AAV9-RBMS1-2.JPG]

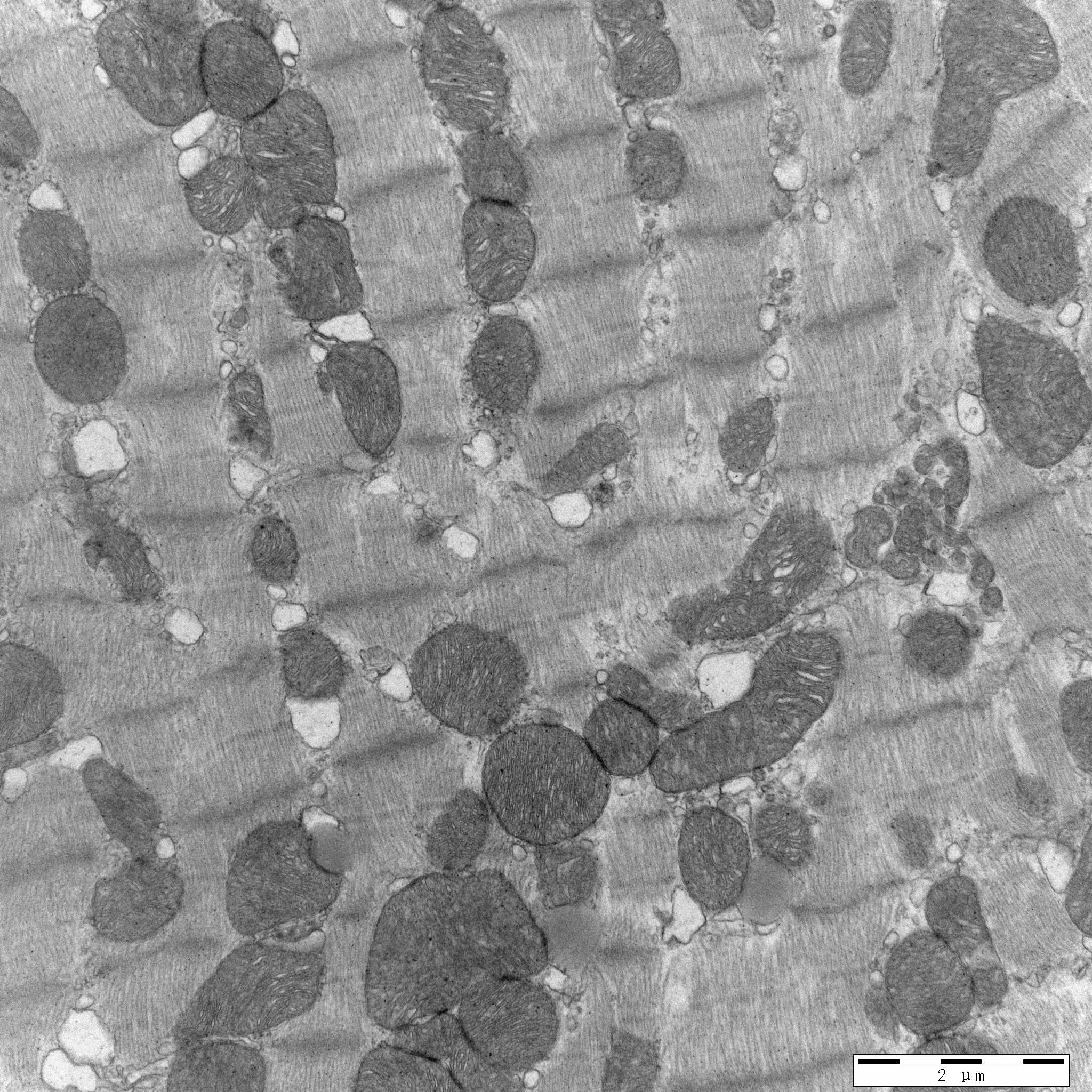

Supplement: Supplementary file 10 — Source data Fig. 7 [file 44321_2025_334_MOESM10_ESM.zip › Figure 7/7J/TAC+AAV9-sh-Δe11+AAV9-Vector-1.JPG]

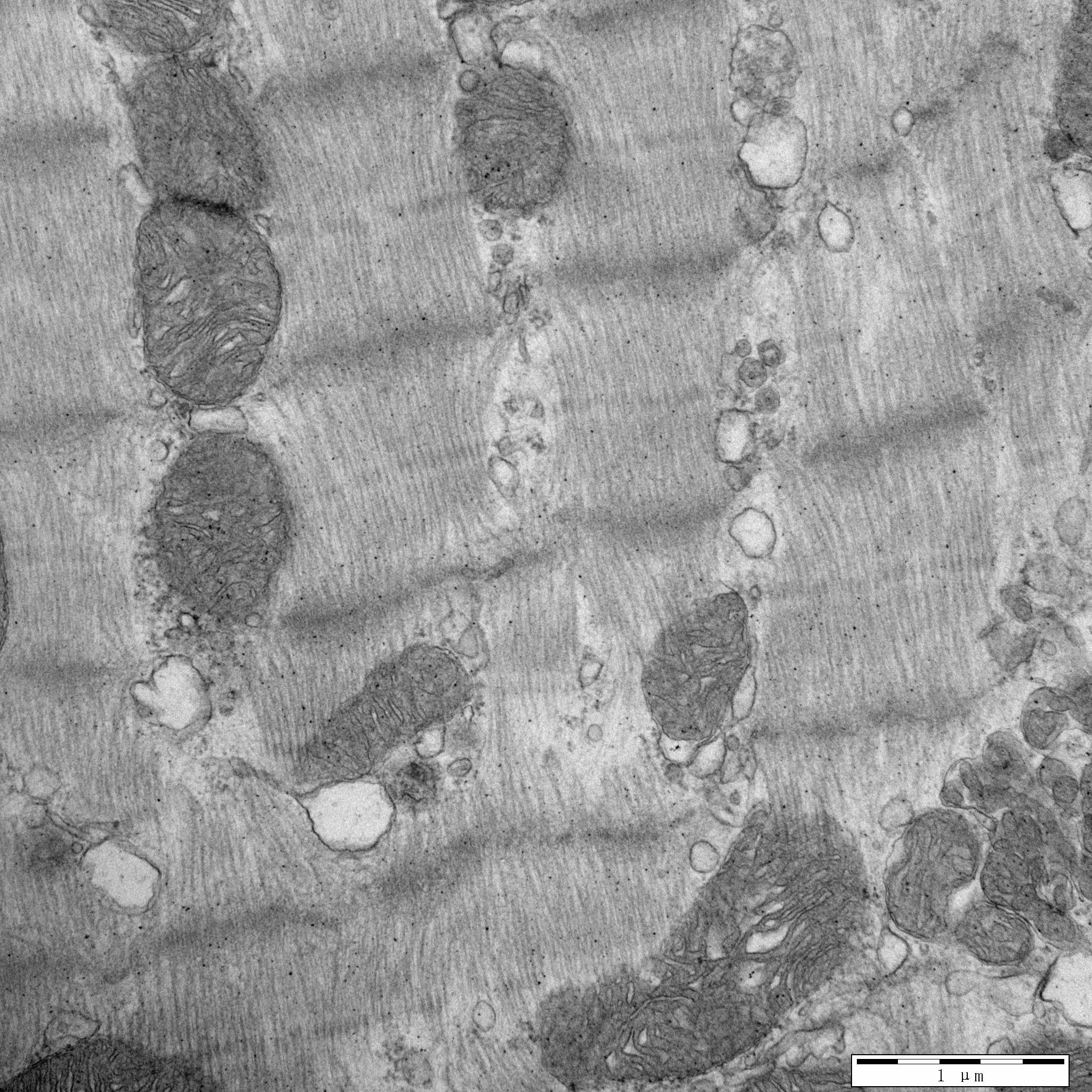

Supplement: Supplementary file 10 — Source data Fig. 7 [file 44321_2025_334_MOESM10_ESM.zip › Figure 7/7J/TAC+AAV9-sh-Δe11+AAV9-Vector-2.JPG]

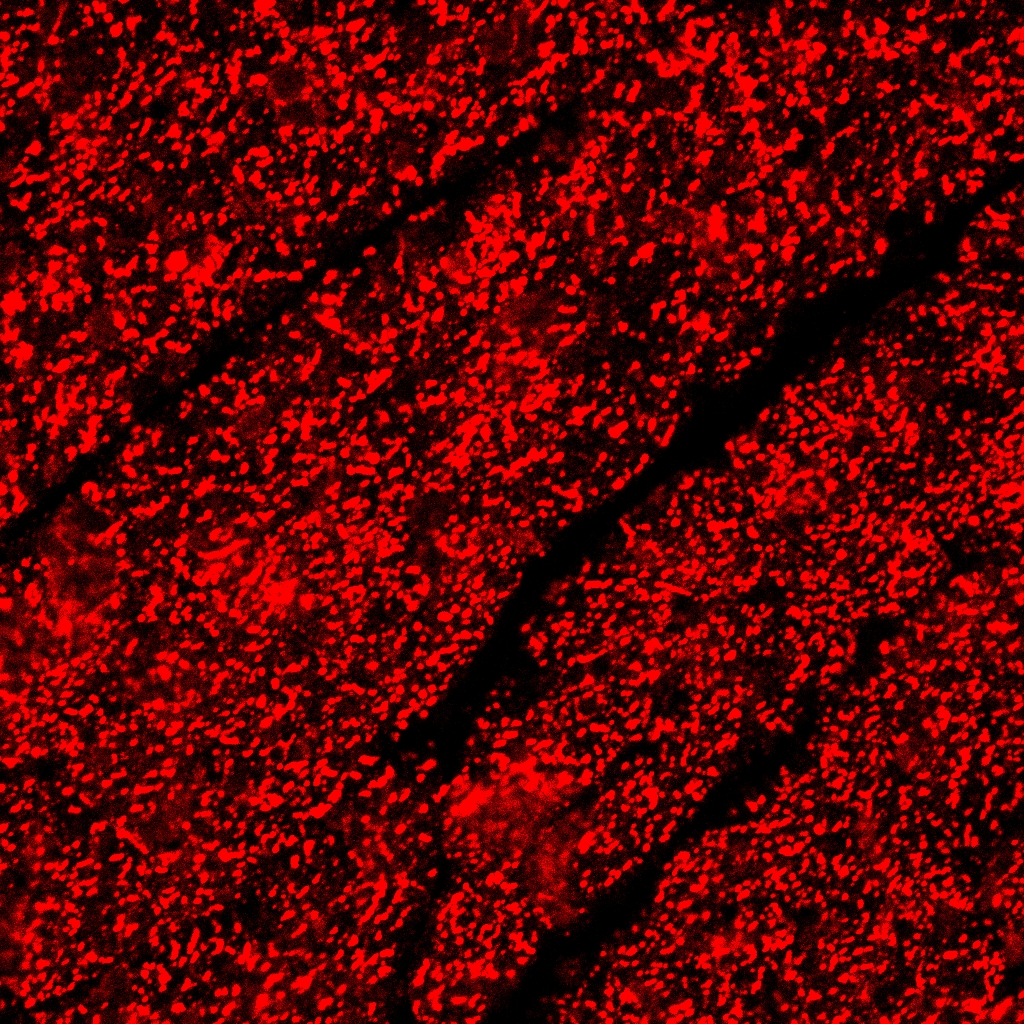

Supplement: Supplementary file 10 — Source data Fig. 7 [file 44321_2025_334_MOESM10_ESM.zip › Figure 7/7K/TAC+AAV9-sh-Vector+AAV9-RBMS1-ACTN2.jpeg]

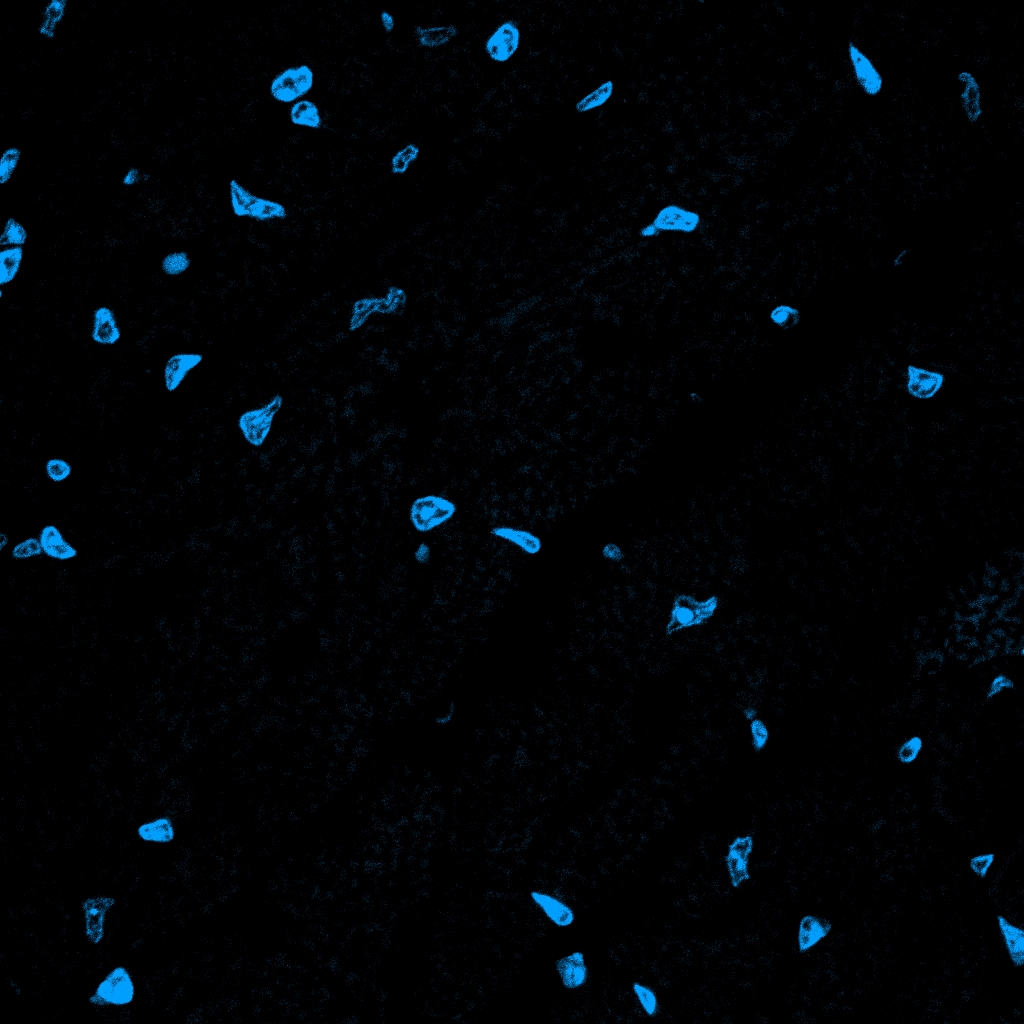

Supplement: Supplementary file 10 — Source data Fig. 7 [file 44321_2025_334_MOESM10_ESM.zip › Figure 7/7K/TAC+AAV9-sh-Vector+AAV9-RBMS1-DAPI.jpeg]

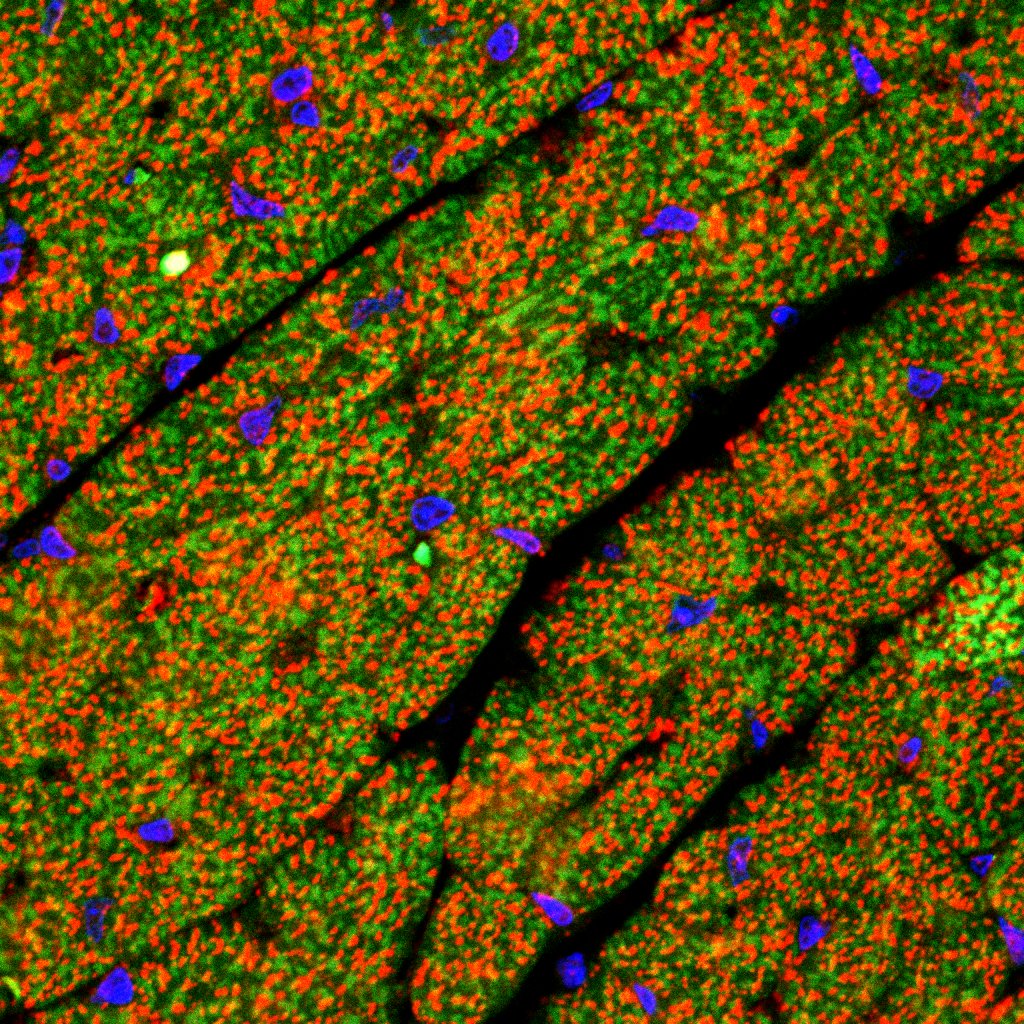

Supplement: Supplementary file 10 — Source data Fig. 7 [file 44321_2025_334_MOESM10_ESM.zip › Figure 7/7K/TAC+AAV9-sh-Vector+AAV9-RBMS1-Merge.jpg]

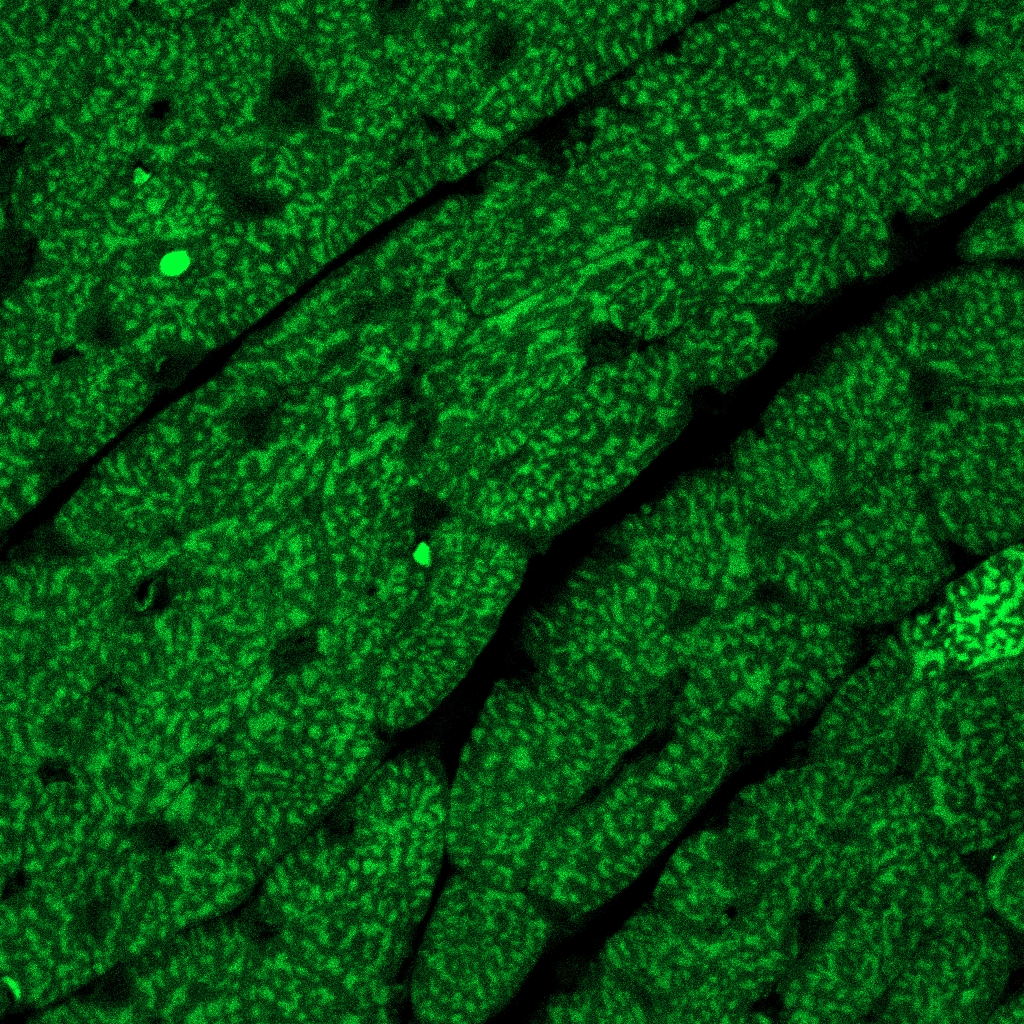

Supplement: Supplementary file 10 — Source data Fig. 7 [file 44321_2025_334_MOESM10_ESM.zip › Figure 7/7K/TAC+AAV9-sh-Vector+AAV9-RBMS1-α-ACTININ.jpeg]

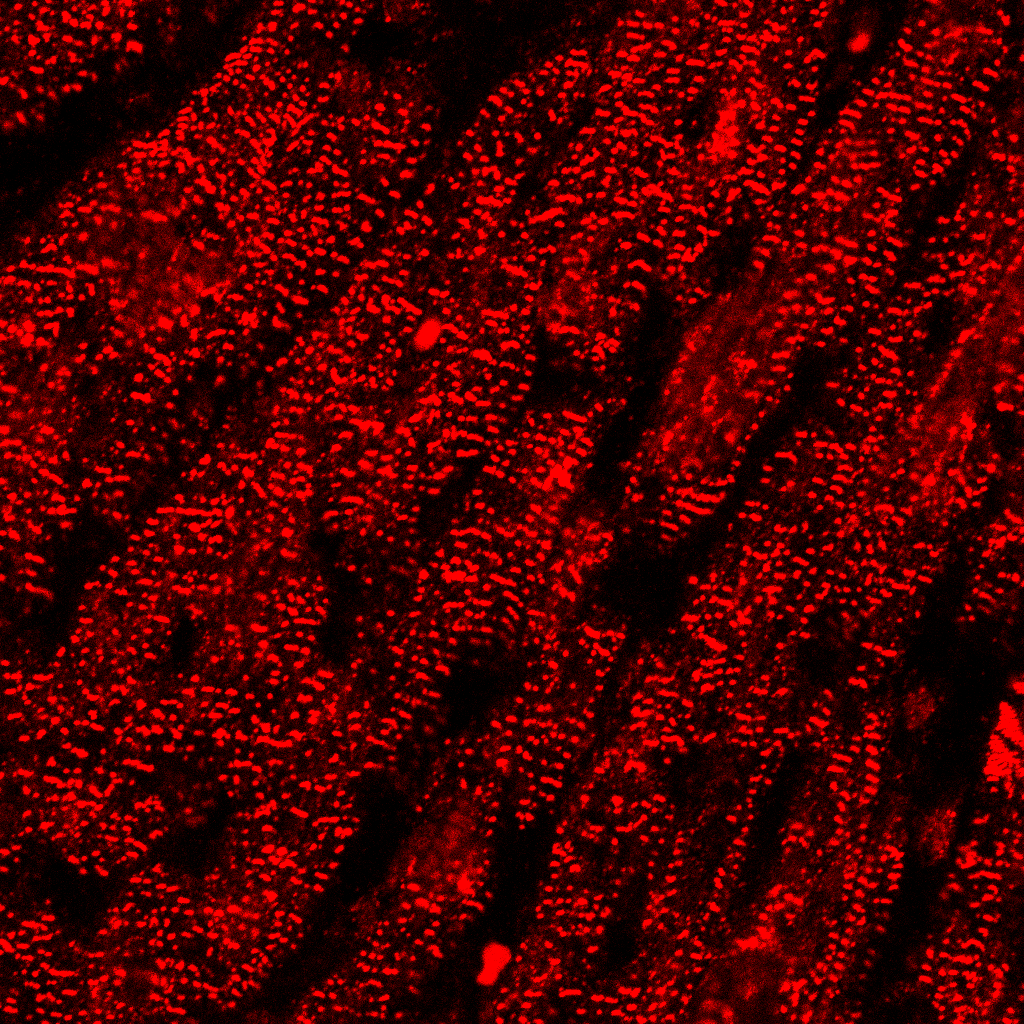

Supplement: Supplementary file 10 — Source data Fig. 7 [file 44321_2025_334_MOESM10_ESM.zip › Figure 7/7K/TAC+AAV9-sh-Vector+AAV9-Vector-ACTN2.tiff]

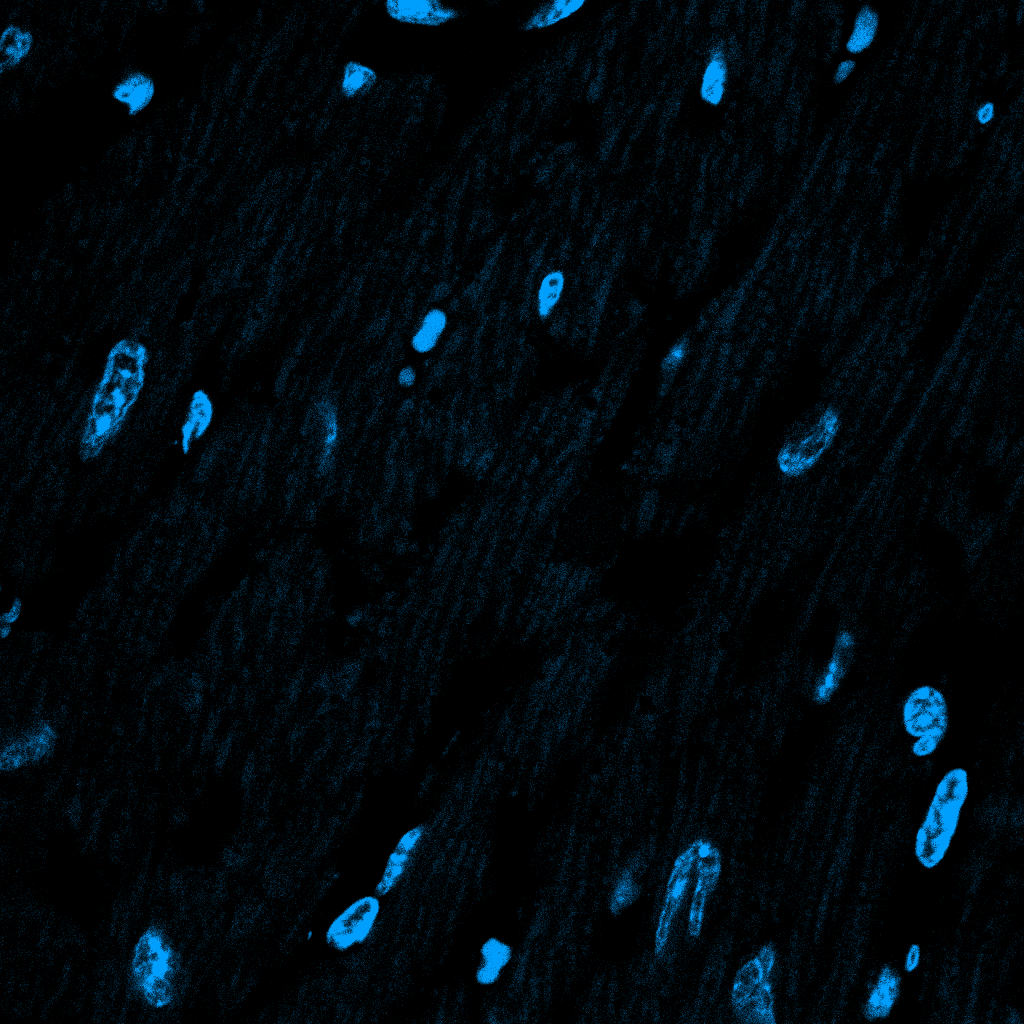

Supplement: Supplementary file 10 — Source data Fig. 7 [file 44321_2025_334_MOESM10_ESM.zip › Figure 7/7K/TAC+AAV9-sh-Vector+AAV9-Vector-DAPI.tiff]

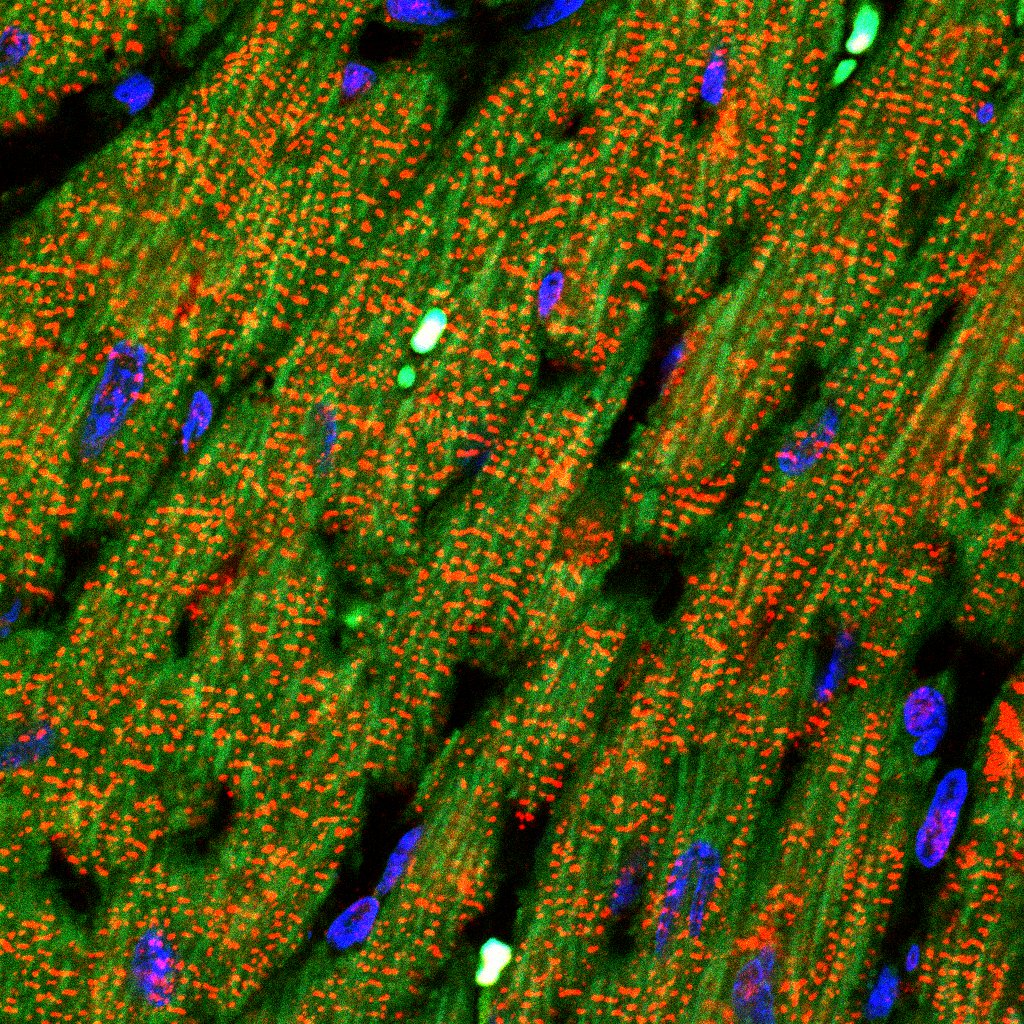

Supplement: Supplementary file 10 — Source data Fig. 7 [file 44321_2025_334_MOESM10_ESM.zip › Figure 7/7K/TAC+AAV9-sh-Vector+AAV9-Vector-Merge.jpg]

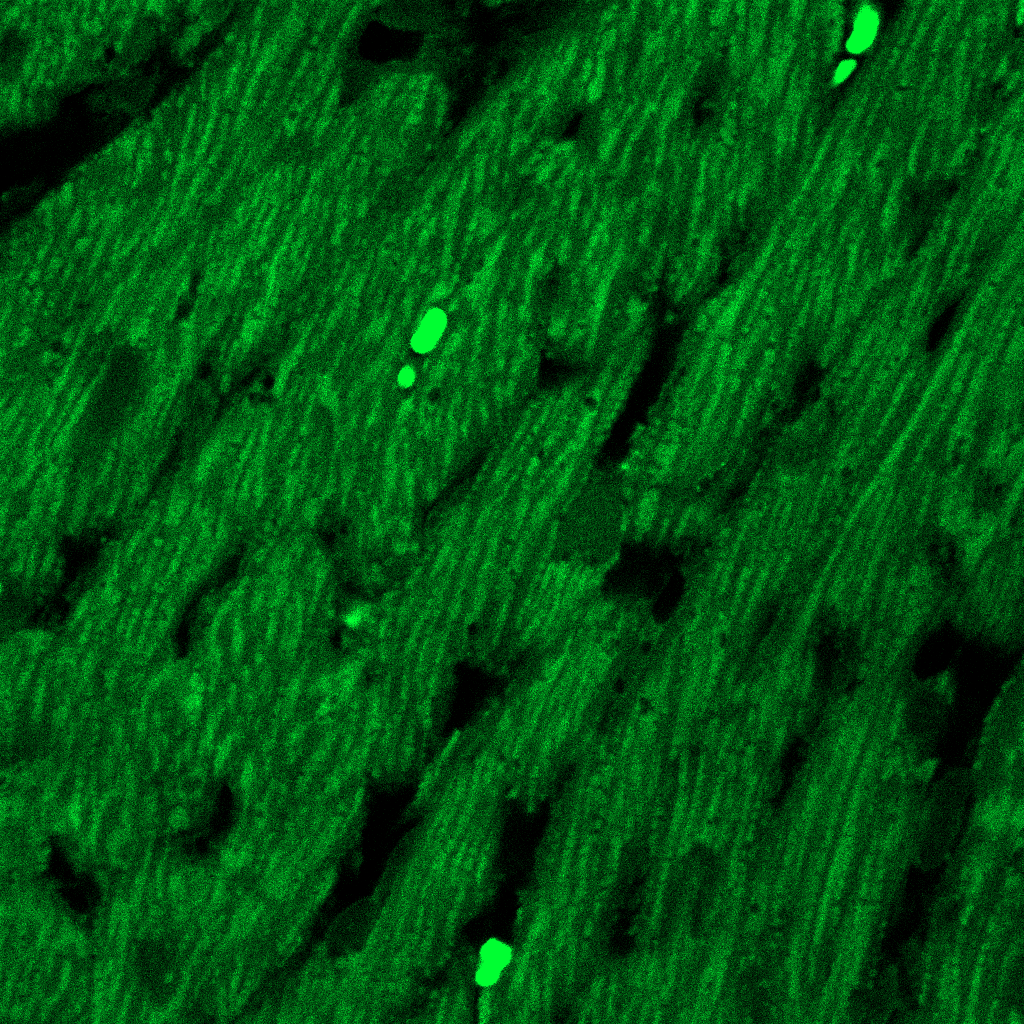

Supplement: Supplementary file 10 — Source data Fig. 7 [file 44321_2025_334_MOESM10_ESM.zip › Figure 7/7K/TAC+AAV9-sh-Vector+AAV9-Vector-α-ACTININ.tiff]

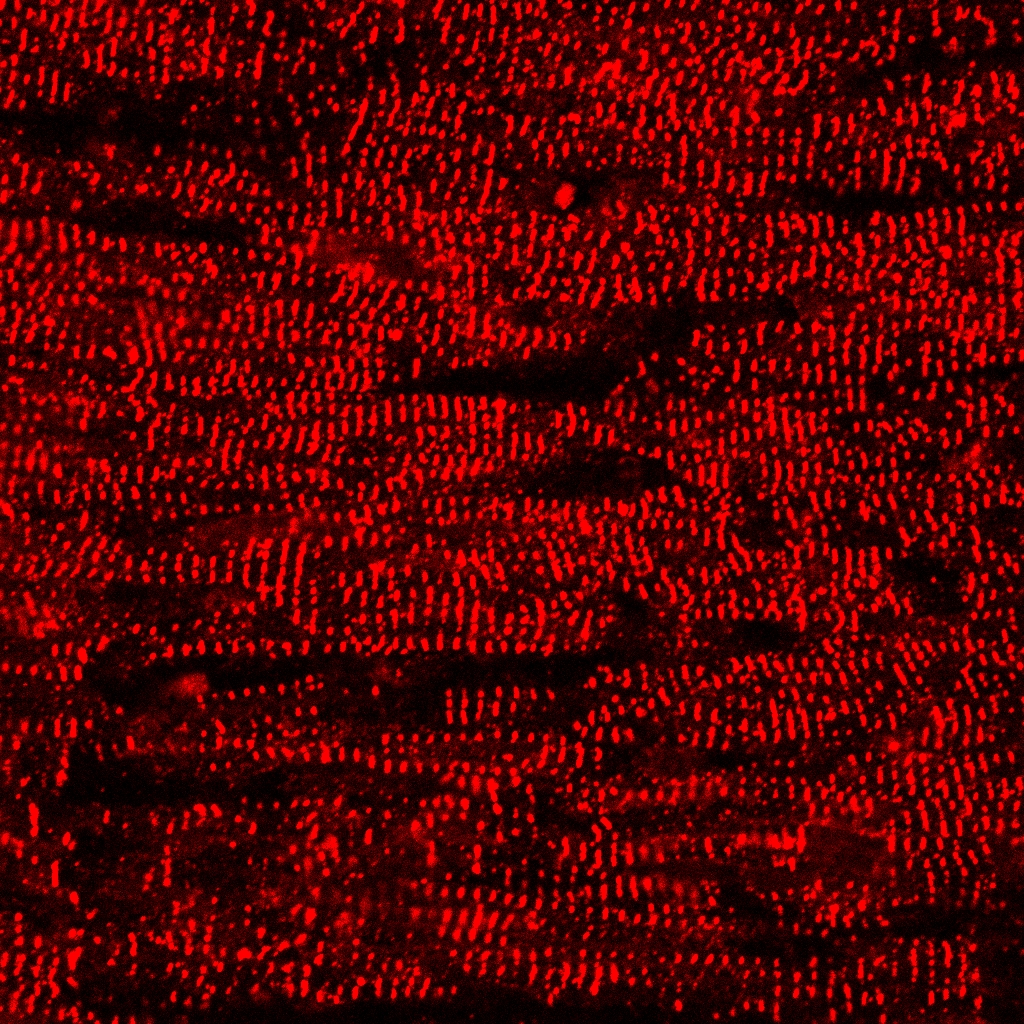

Supplement: Supplementary file 10 — Source data Fig. 7 [file 44321_2025_334_MOESM10_ESM.zip › Figure 7/7K/TAC+AAV9-sh-Δe11+AAV9-RBMS1-ACTN2.jpeg]

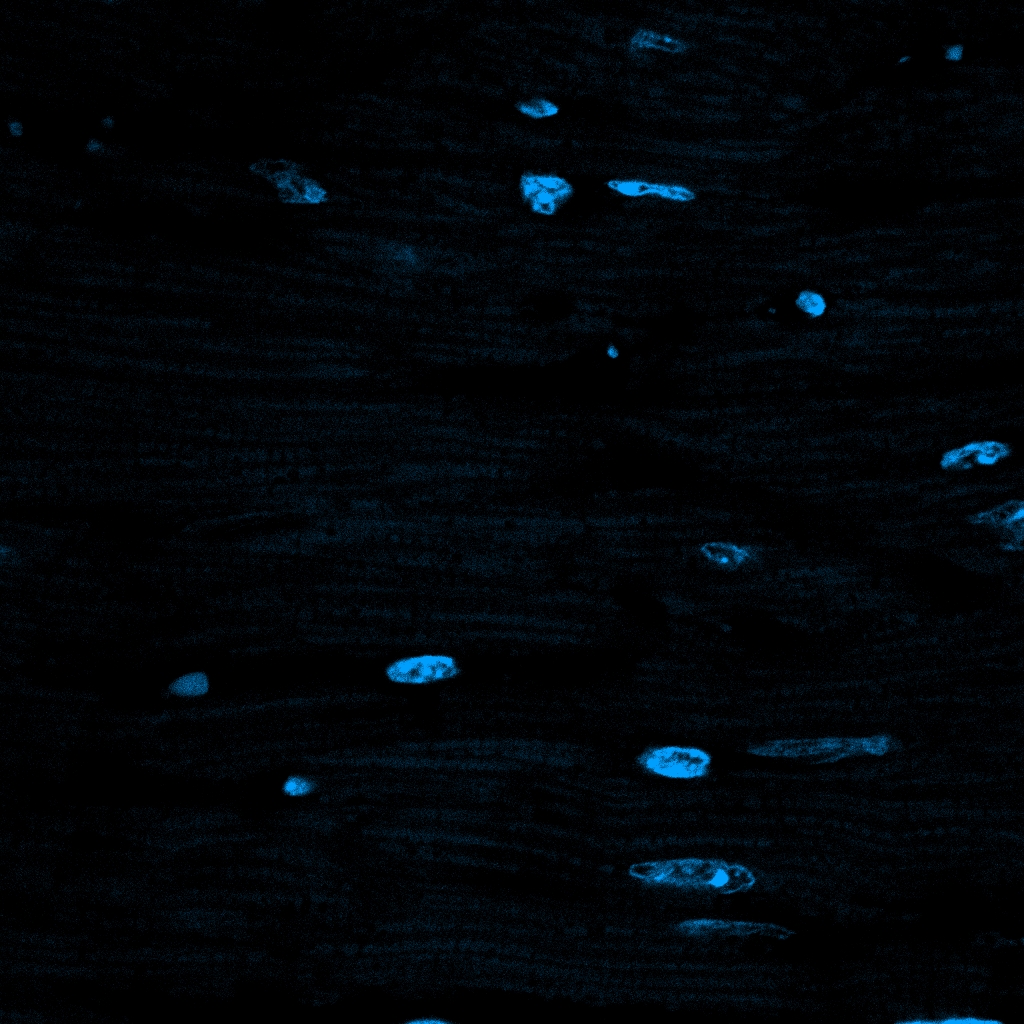

Supplement: Supplementary file 10 — Source data Fig. 7 [file 44321_2025_334_MOESM10_ESM.zip › Figure 7/7K/TAC+AAV9-sh-Δe11+AAV9-RBMS1-DAPI.jpeg]

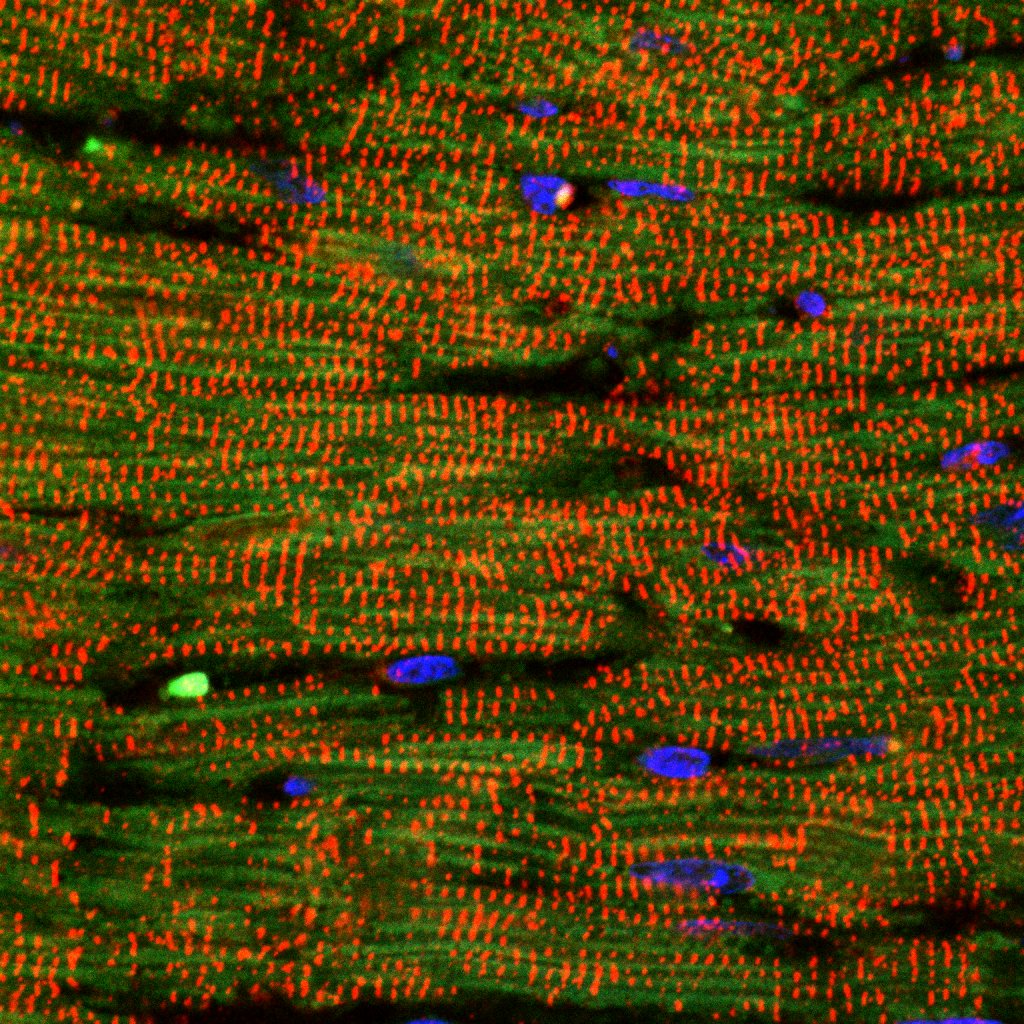

Supplement: Supplementary file 10 — Source data Fig. 7 [file 44321_2025_334_MOESM10_ESM.zip › Figure 7/7K/TAC+AAV9-sh-Δe11+AAV9-RBMS1-Merge.jpg]

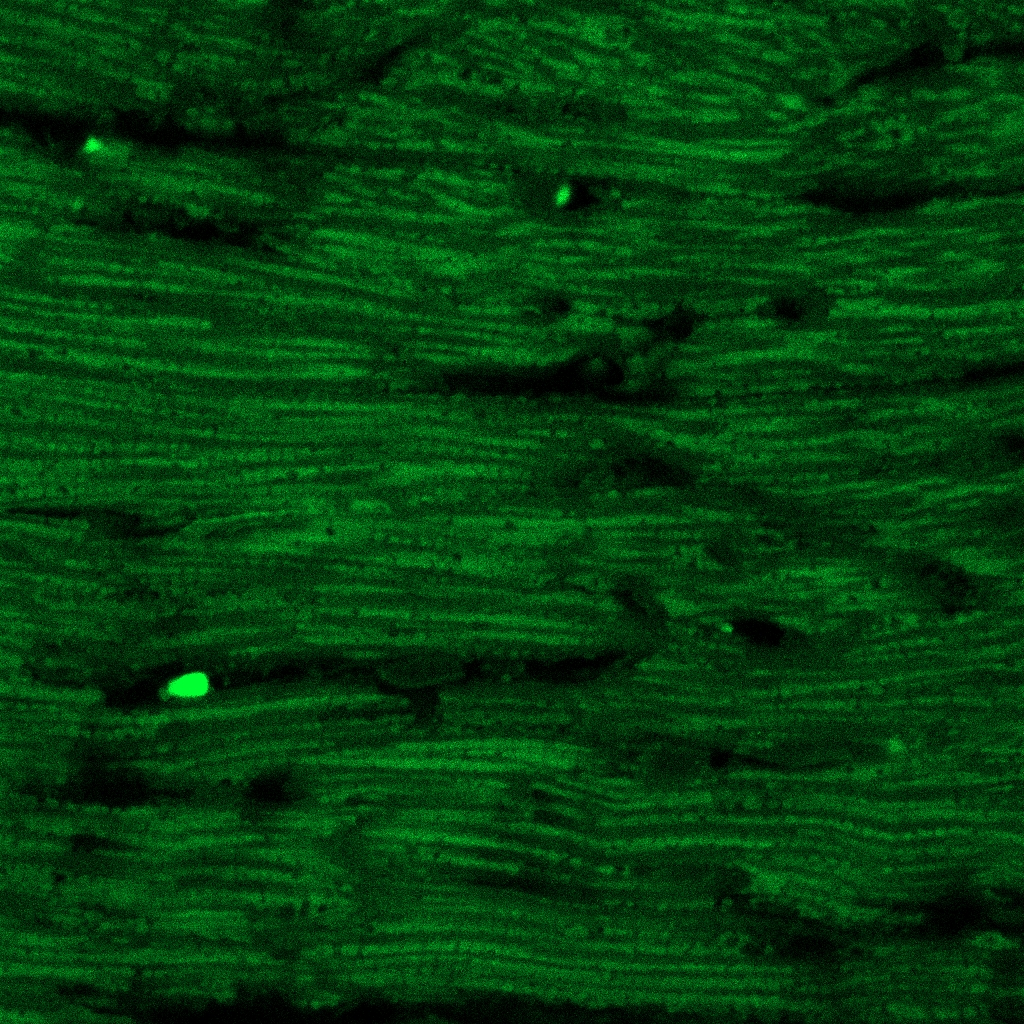

Supplement: Supplementary file 10 — Source data Fig. 7 [file 44321_2025_334_MOESM10_ESM.zip › Figure 7/7K/TAC+AAV9-sh-Δe11+AAV9-RBMS1-α-ACTININ.jpeg]

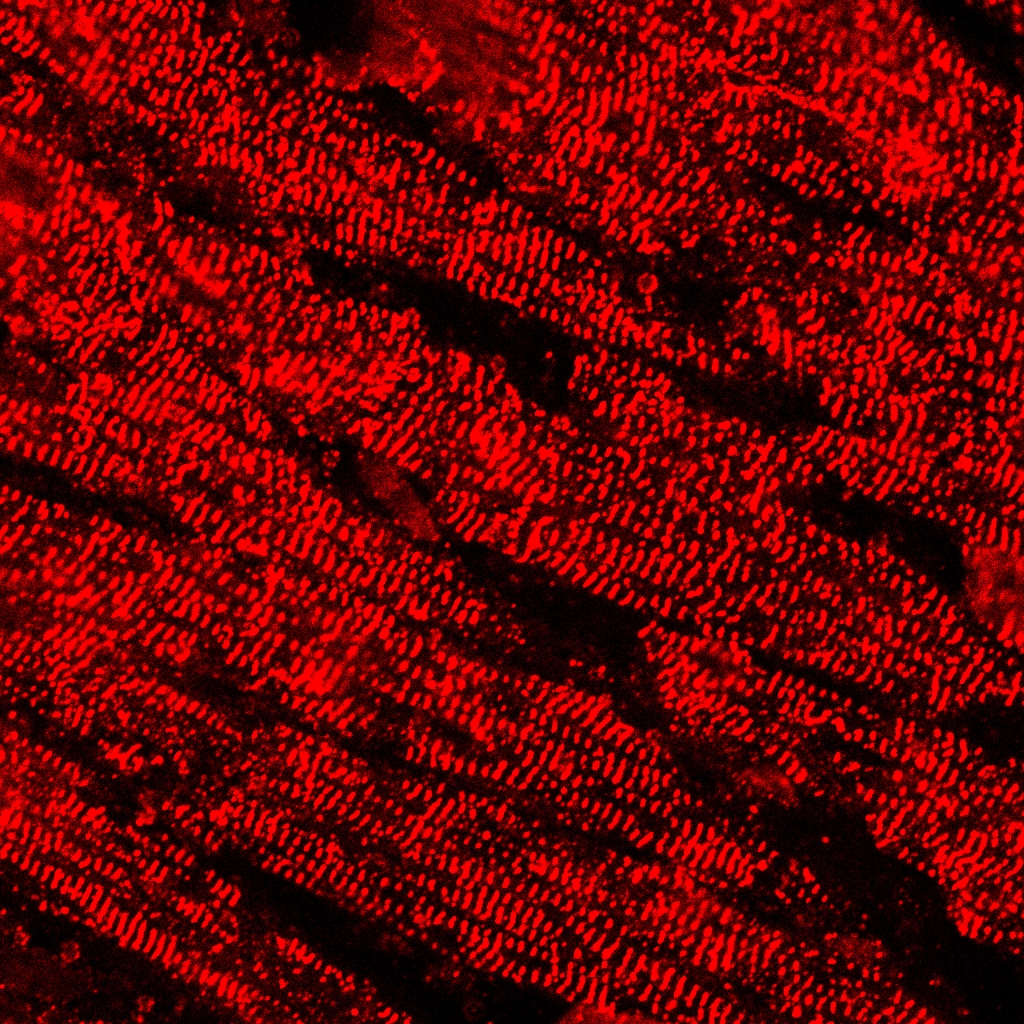

Supplement: Supplementary file 10 — Source data Fig. 7 [file 44321_2025_334_MOESM10_ESM.zip › Figure 7/7K/TAC+AAV9-sh-Δe11+AAV9-Vector-ACTN2.jpeg]

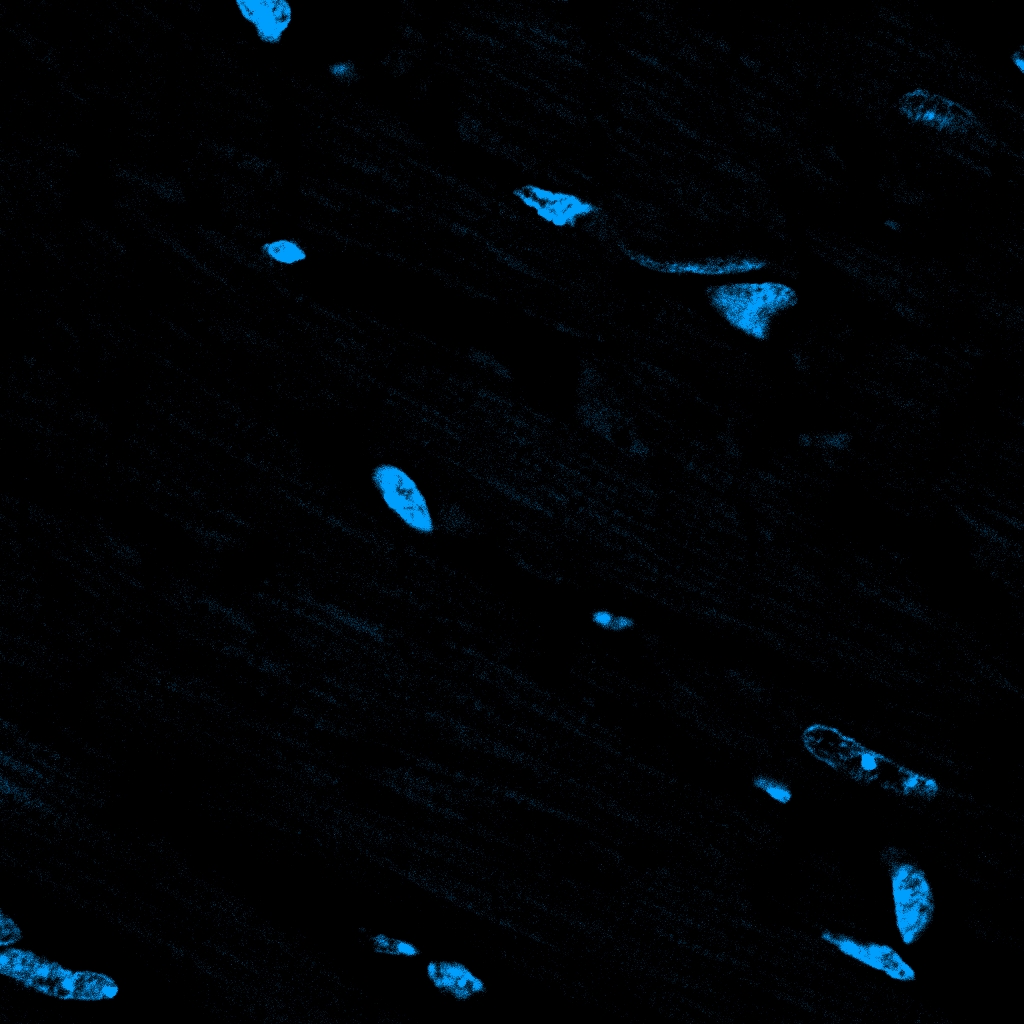

Supplement: Supplementary file 10 — Source data Fig. 7 [file 44321_2025_334_MOESM10_ESM.zip › Figure 7/7K/TAC+AAV9-sh-Δe11+AAV9-Vector-DAPI.jpeg]

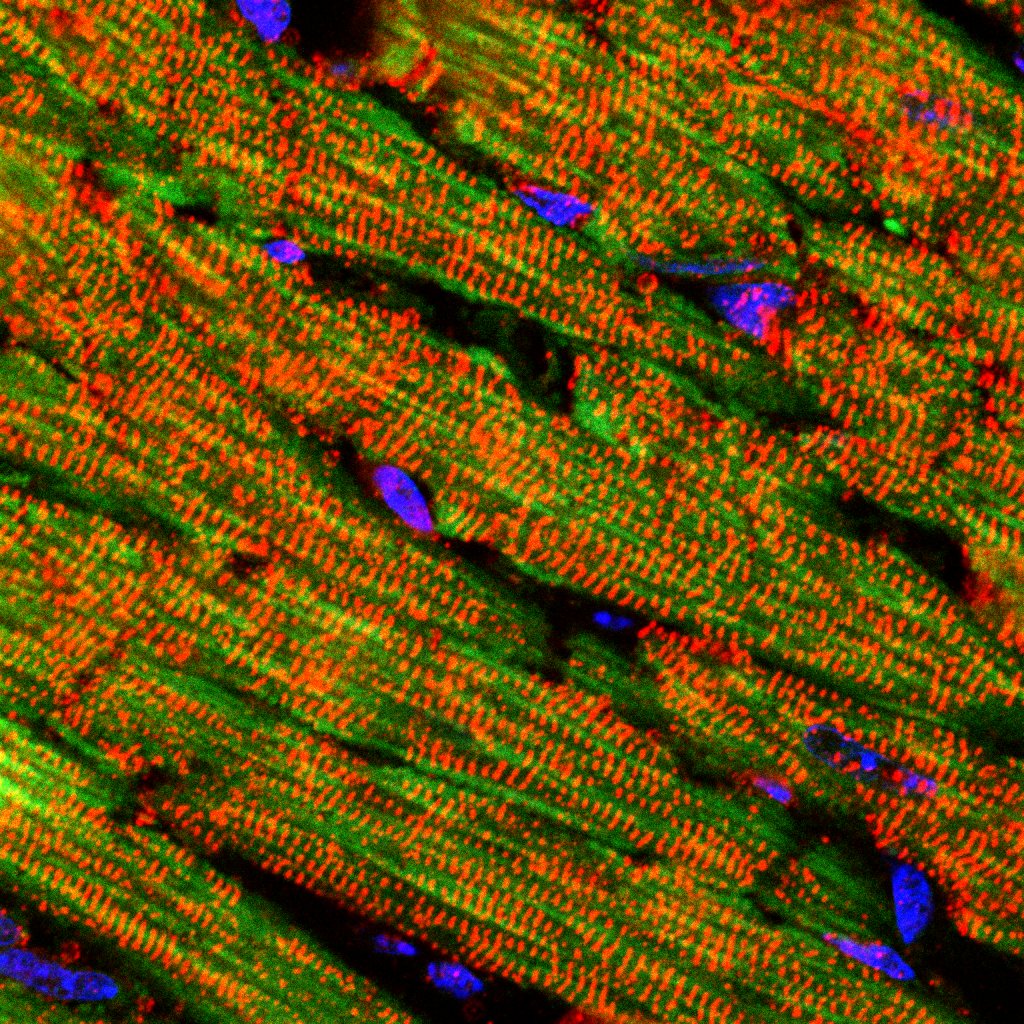

Supplement: Supplementary file 10 — Source data Fig. 7 [file 44321_2025_334_MOESM10_ESM.zip › Figure 7/7K/TAC+AAV9-sh-Δe11+AAV9-Vector-Merge.jpg]

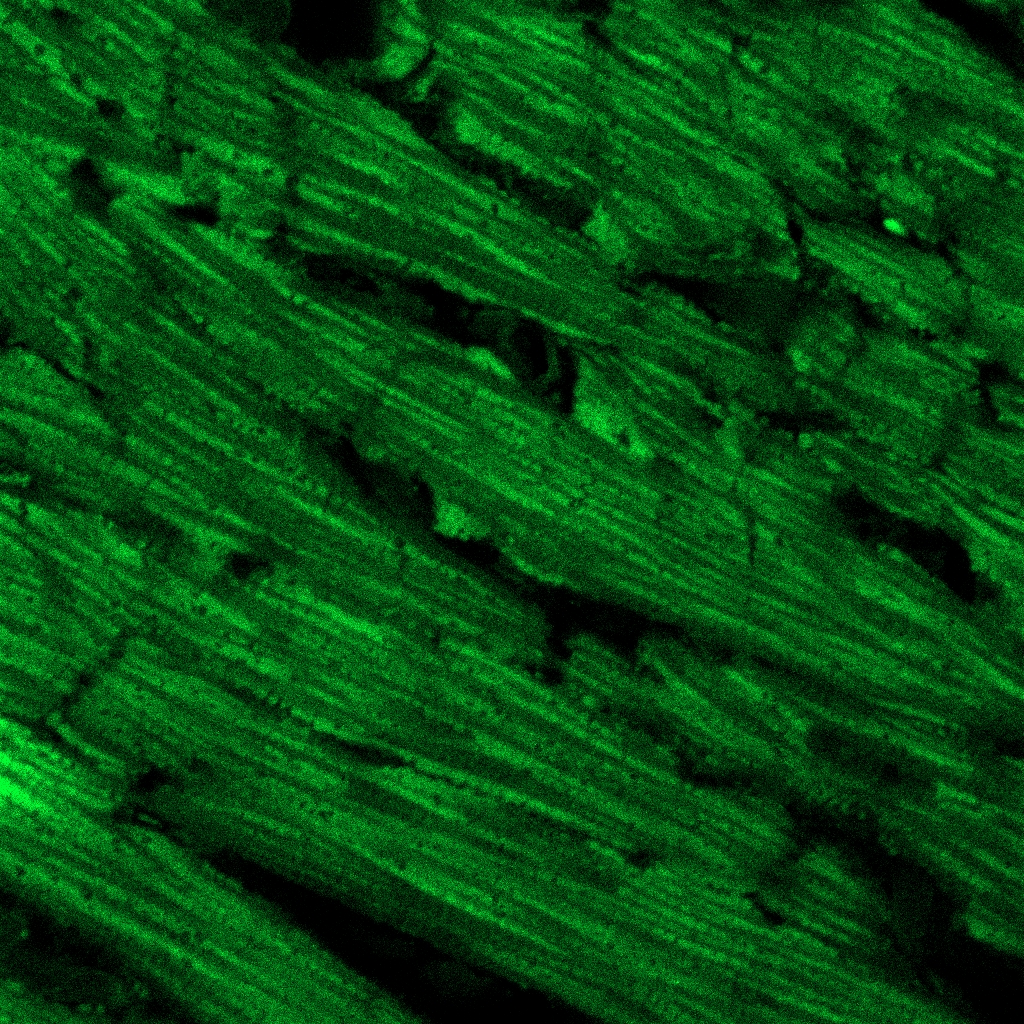

Supplement: Supplementary file 10 — Source data Fig. 7 [file 44321_2025_334_MOESM10_ESM.zip › Figure 7/7K/TAC+AAV9-sh-Δe11+AAV9-Vector-α-ACTININ.jpeg]

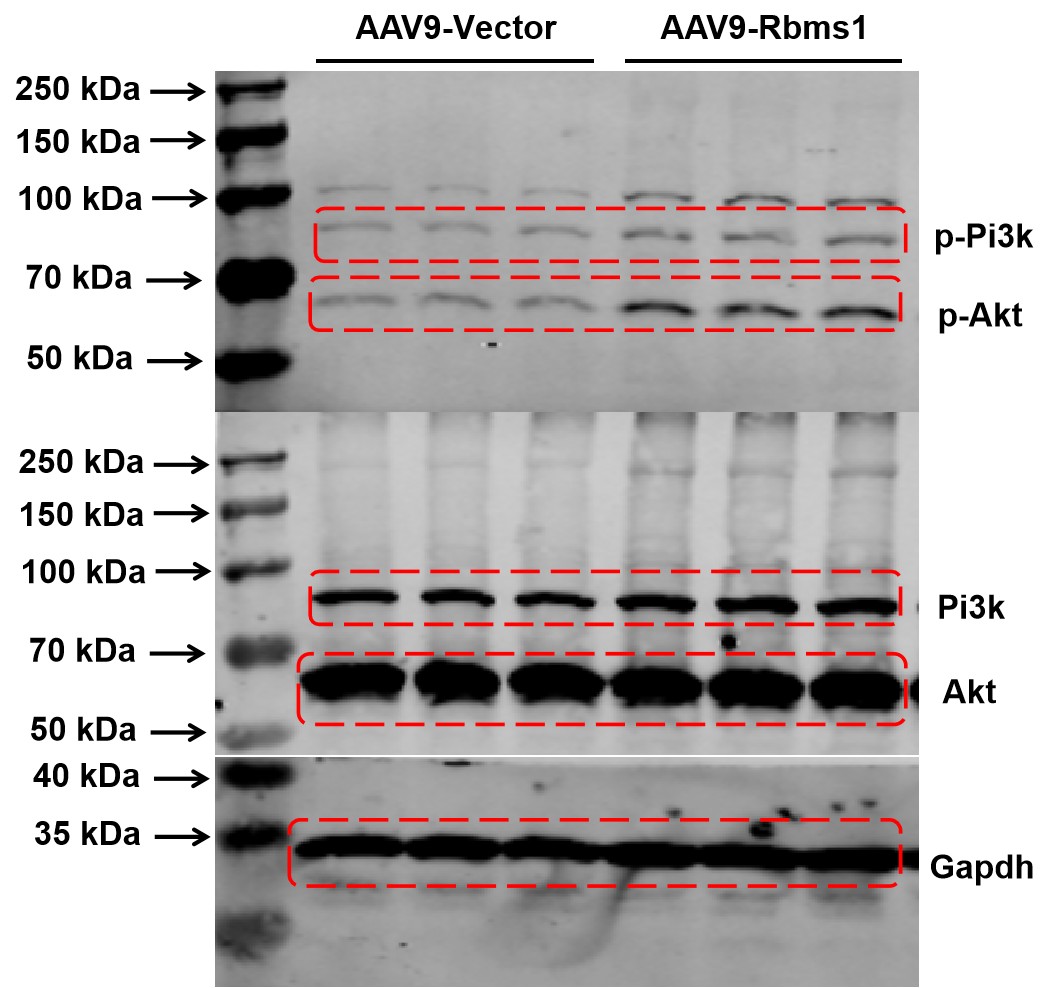

Supplement: Supplementary file 11 — Source data Fig. 8 [file 44321_2025_334_MOESM11_ESM.zip › Figure 8/8D/8D.jpg]

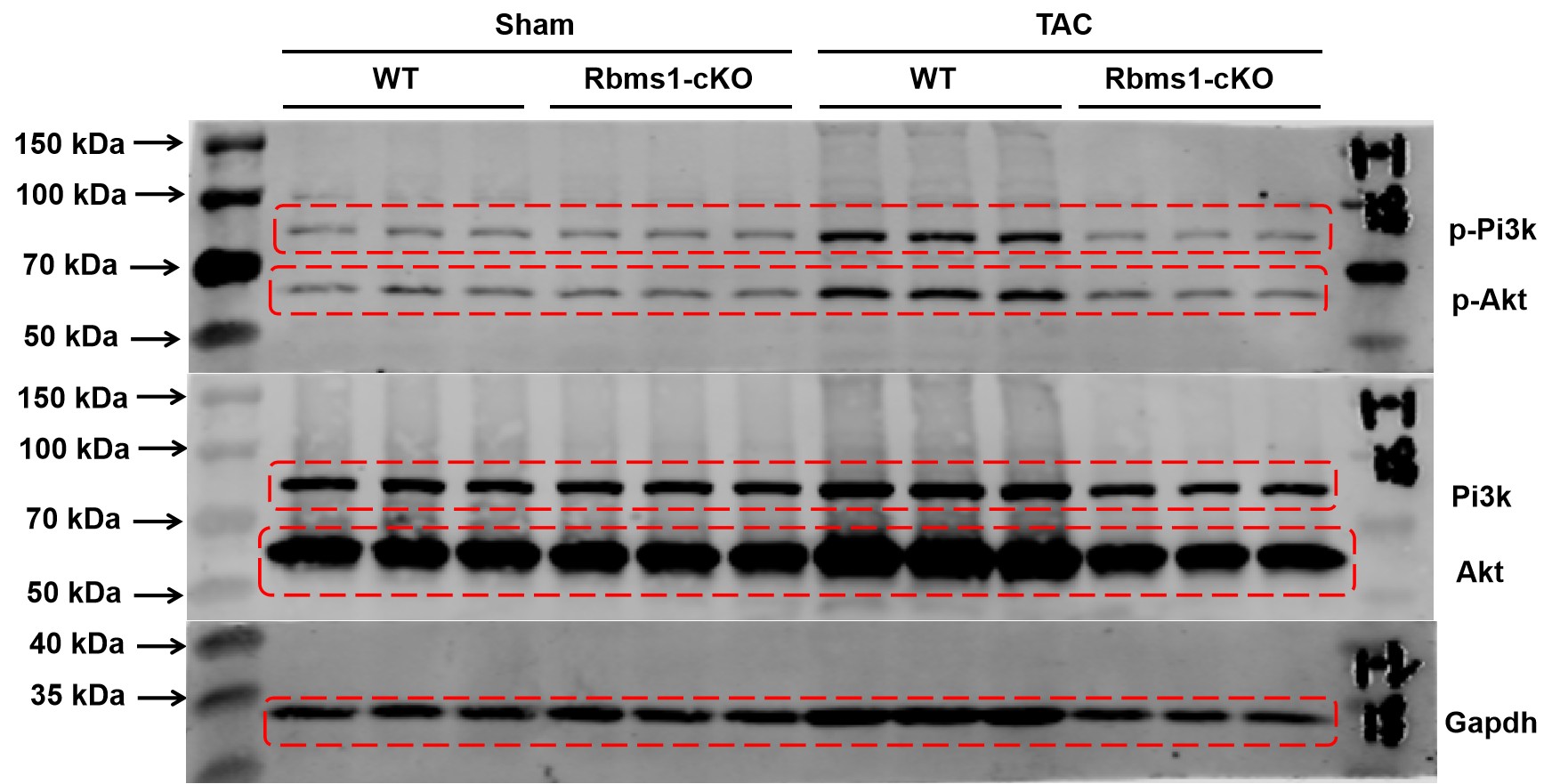

Supplement: Supplementary file 11 — Source data Fig. 8 [file 44321_2025_334_MOESM11_ESM.zip › Figure 8/8E/8E.jpg]

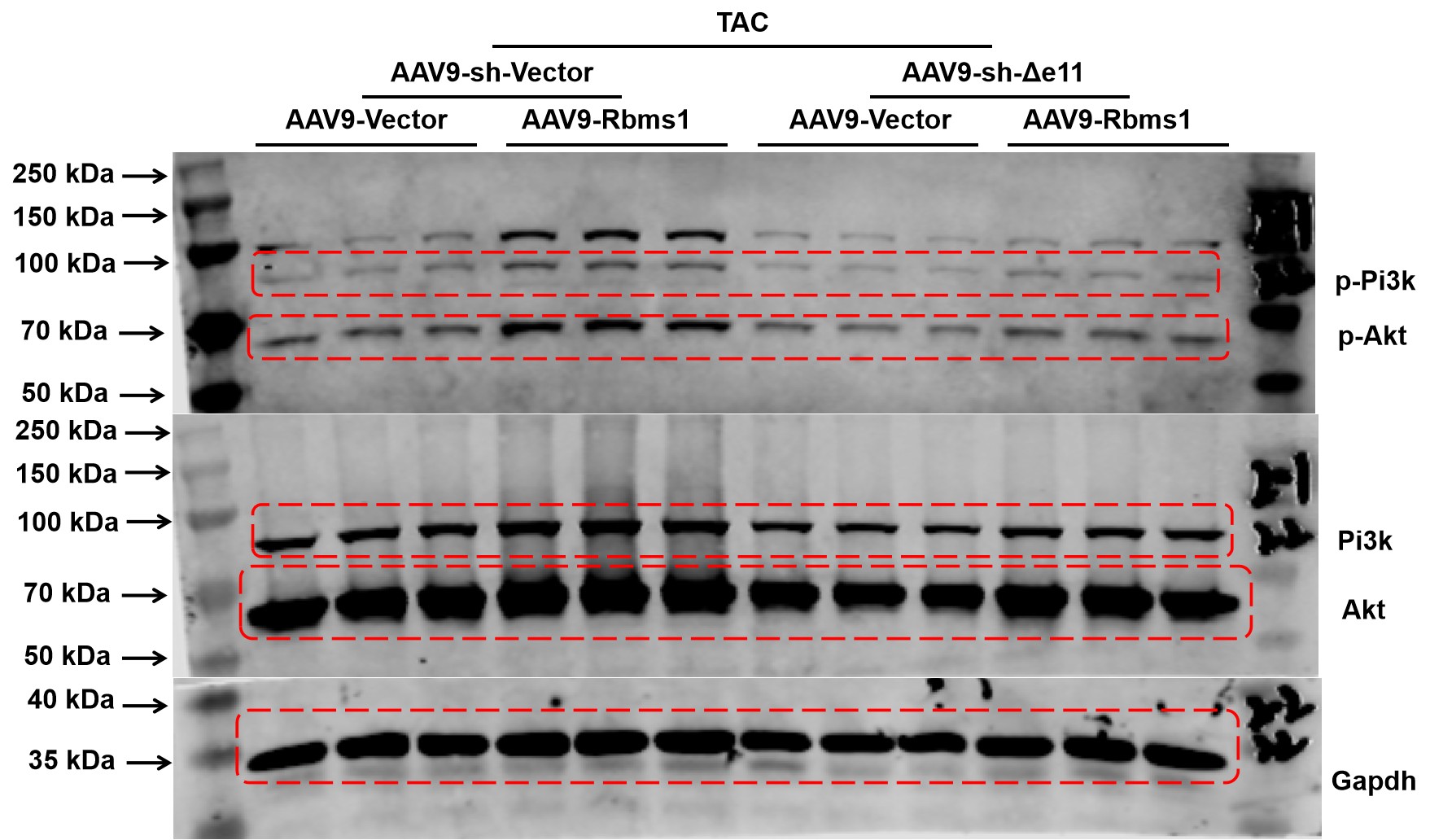

Supplement: Supplementary file 11 — Source data Fig. 8 [file 44321_2025_334_MOESM11_ESM.zip › Figure 8/8G/8G.jpg]

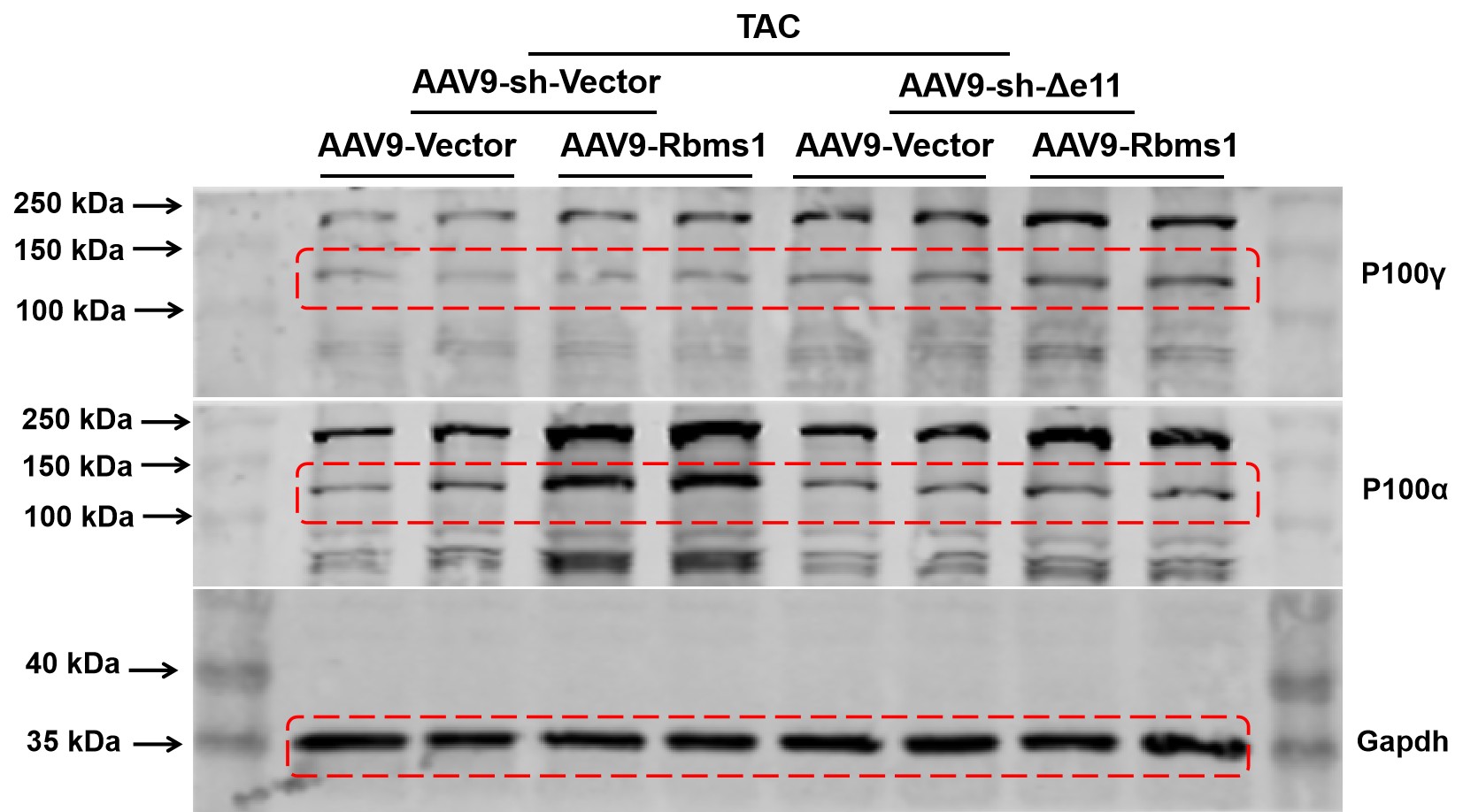

Supplement: Supplementary file 11 — Source data Fig. 8 [file 44321_2025_334_MOESM11_ESM.zip › Figure 8/8H/8H.jpg]

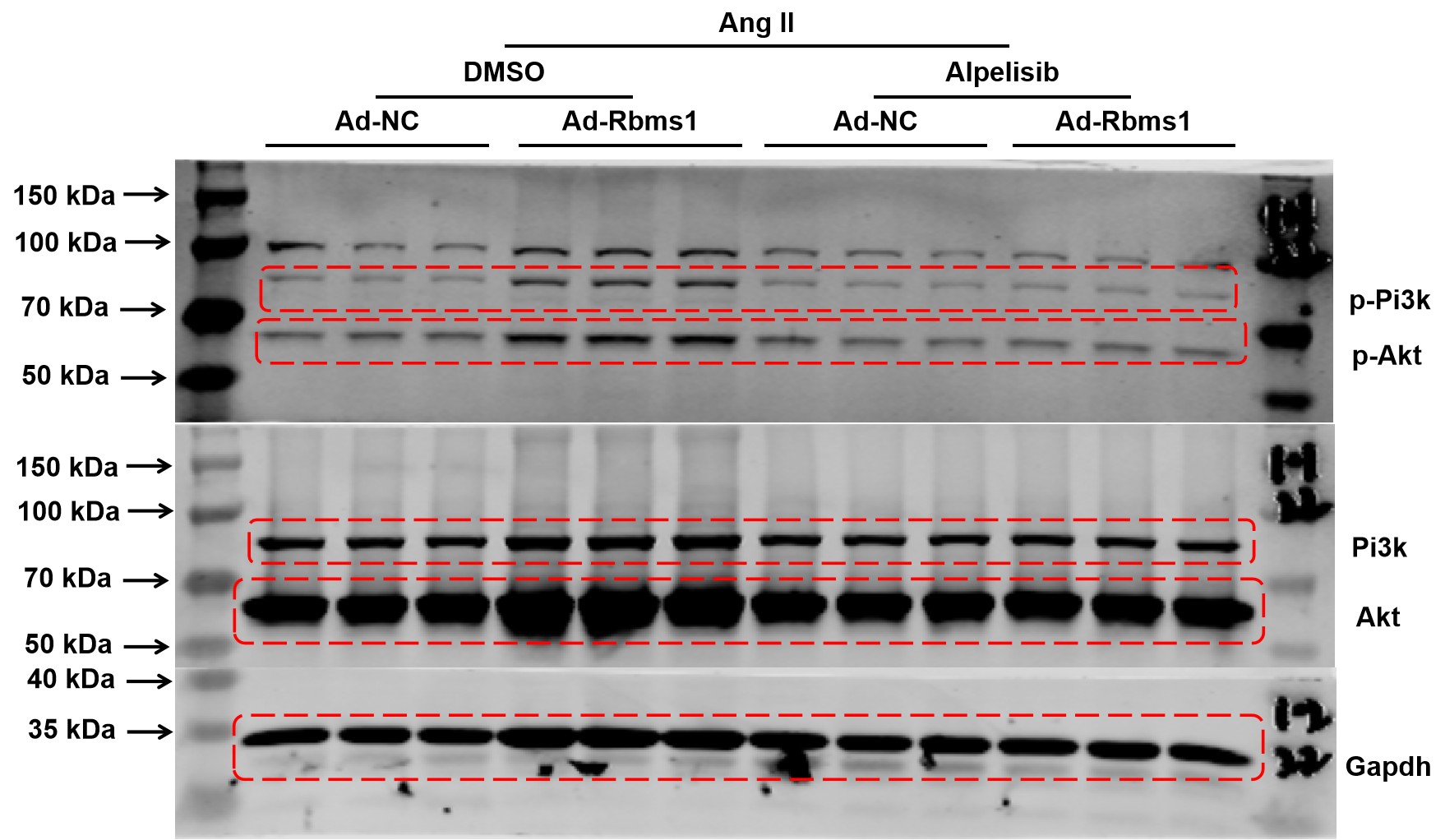

Supplement: Supplementary file 11 — Source data Fig. 8 [file 44321_2025_334_MOESM11_ESM.zip › Figure 8/8I/8I.jpg]

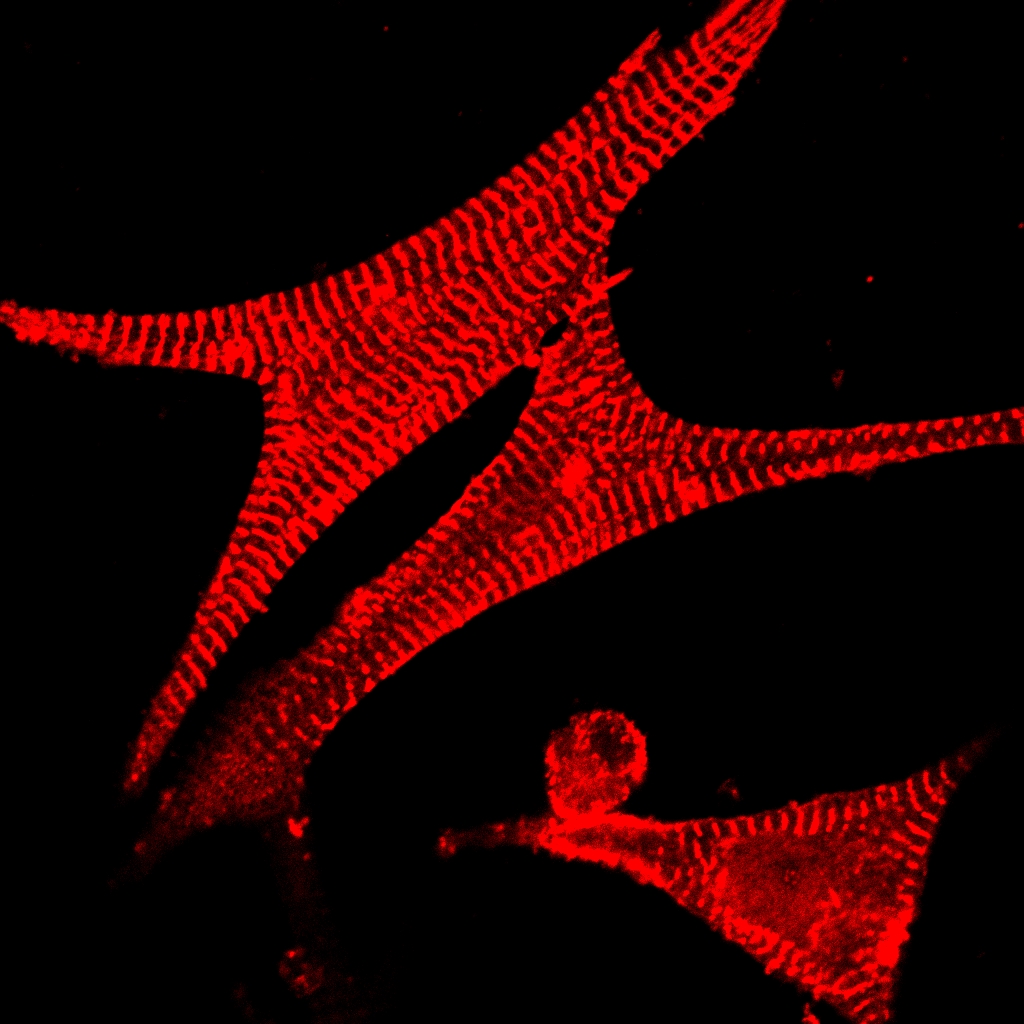

Supplement: Supplementary file 11 — Source data Fig. 8 [file 44321_2025_334_MOESM11_ESM.zip › Figure 8/8J/Ang II+Alpelisib+Ad-NC-ACTN2.jpeg]

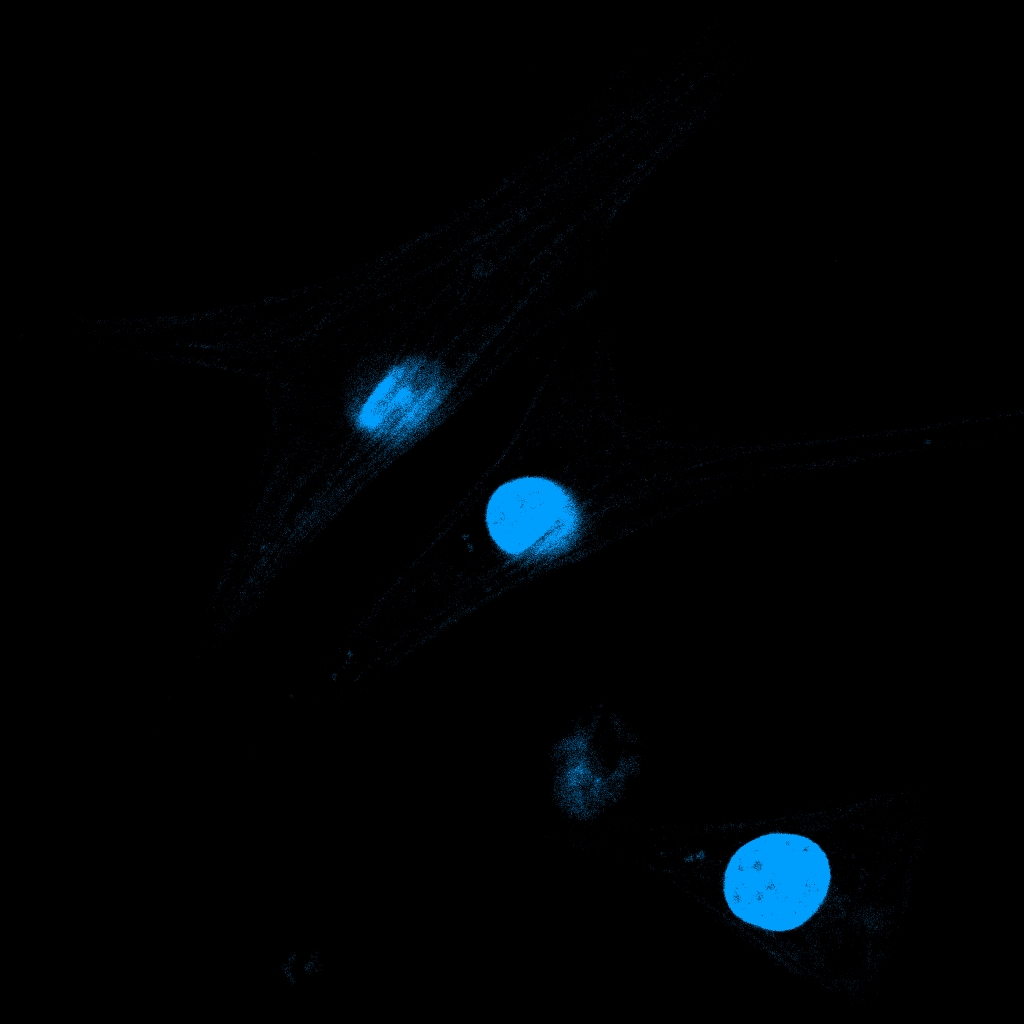

Supplement: Supplementary file 11 — Source data Fig. 8 [file 44321_2025_334_MOESM11_ESM.zip › Figure 8/8J/Ang II+Alpelisib+Ad-NC-DAPI.jpeg]

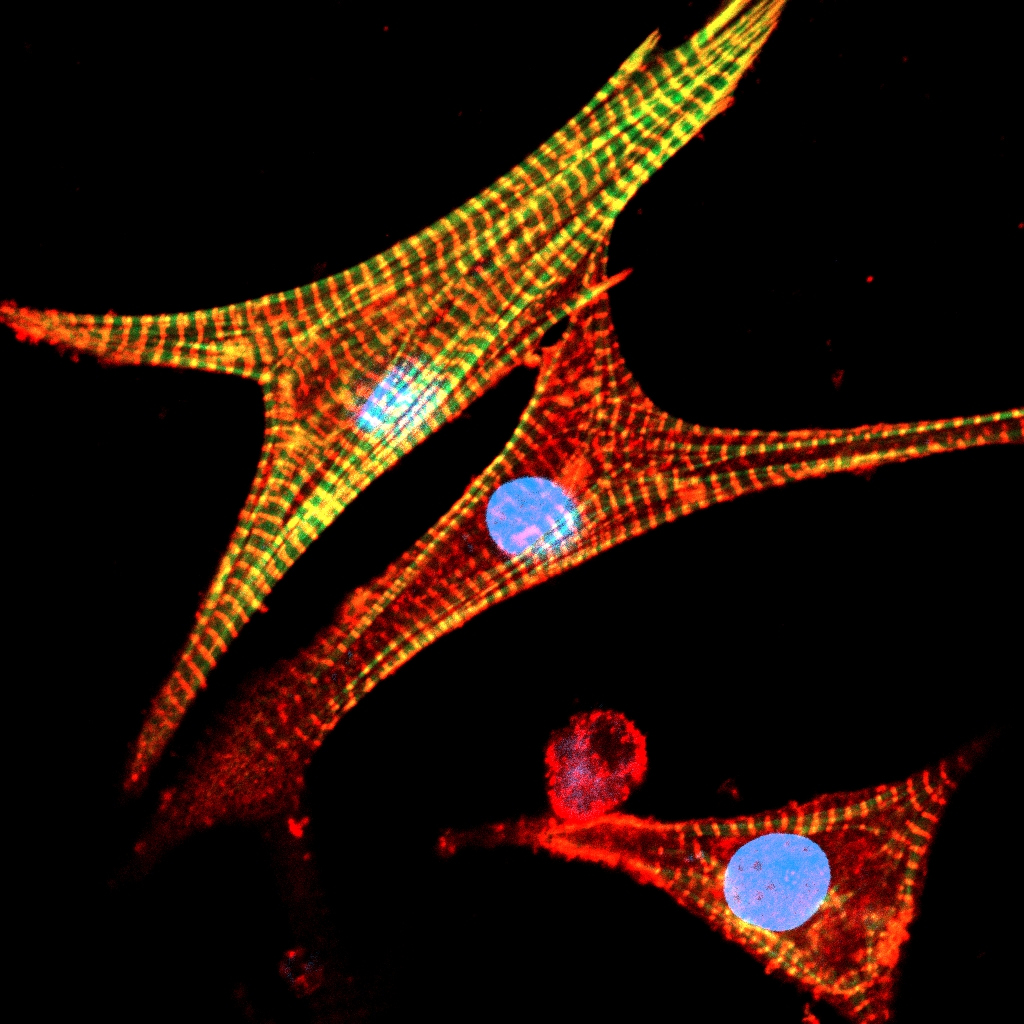

Supplement: Supplementary file 11 — Source data Fig. 8 [file 44321_2025_334_MOESM11_ESM.zip › Figure 8/8J/Ang II+Alpelisib+Ad-NC-Merge.jpeg]

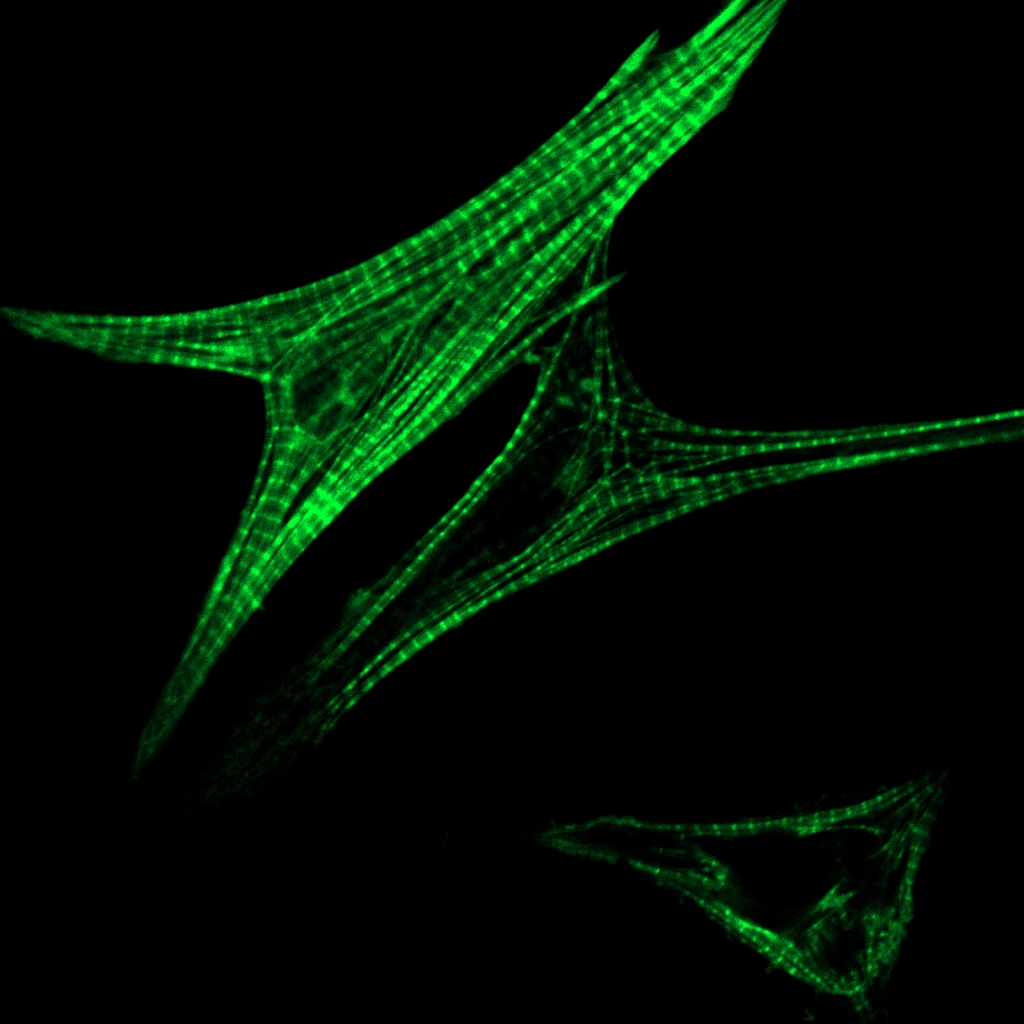

Supplement: Supplementary file 11 — Source data Fig. 8 [file 44321_2025_334_MOESM11_ESM.zip › Figure 8/8J/Ang II+Alpelisib+Ad-NC-Phalloidine.jpeg]

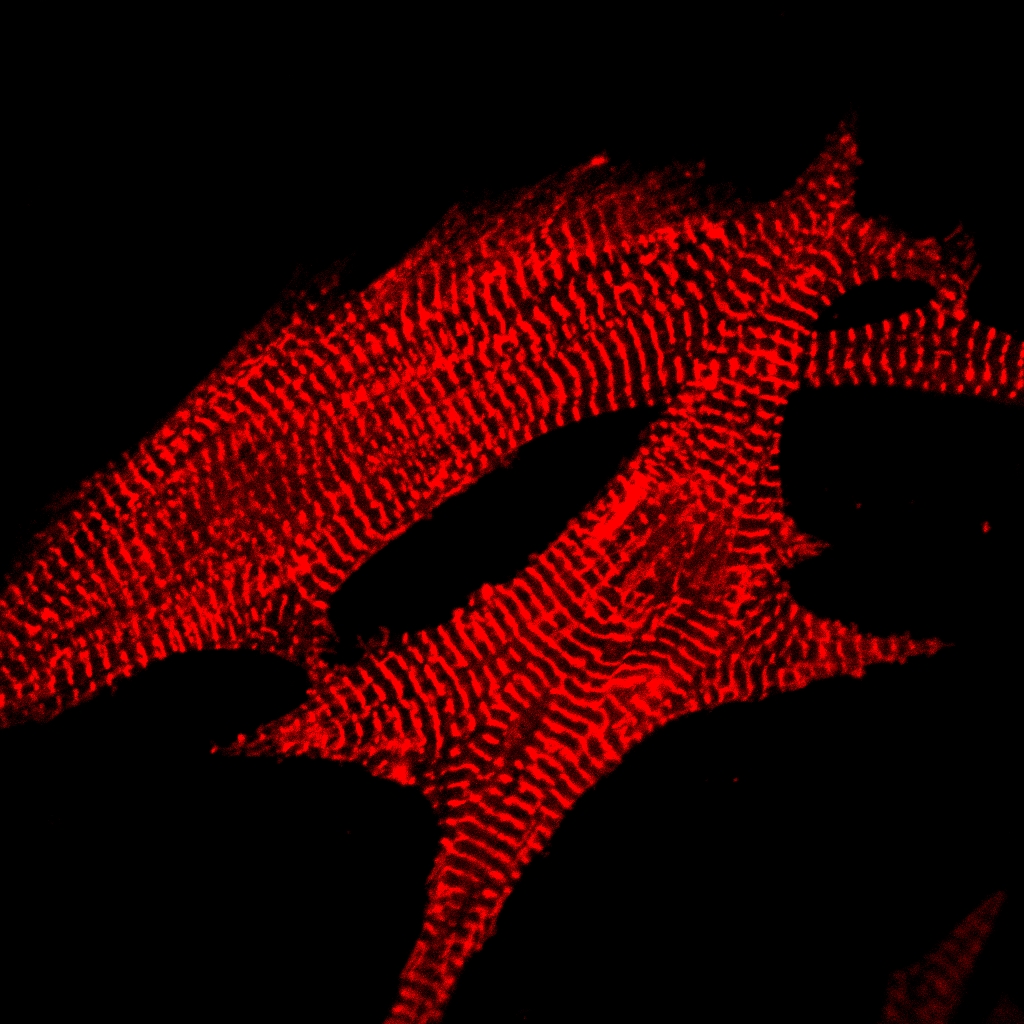

Supplement: Supplementary file 11 — Source data Fig. 8 [file 44321_2025_334_MOESM11_ESM.zip › Figure 8/8J/Ang II+Alpelisib+Ad-RBMS1-ACTN2.jpeg]

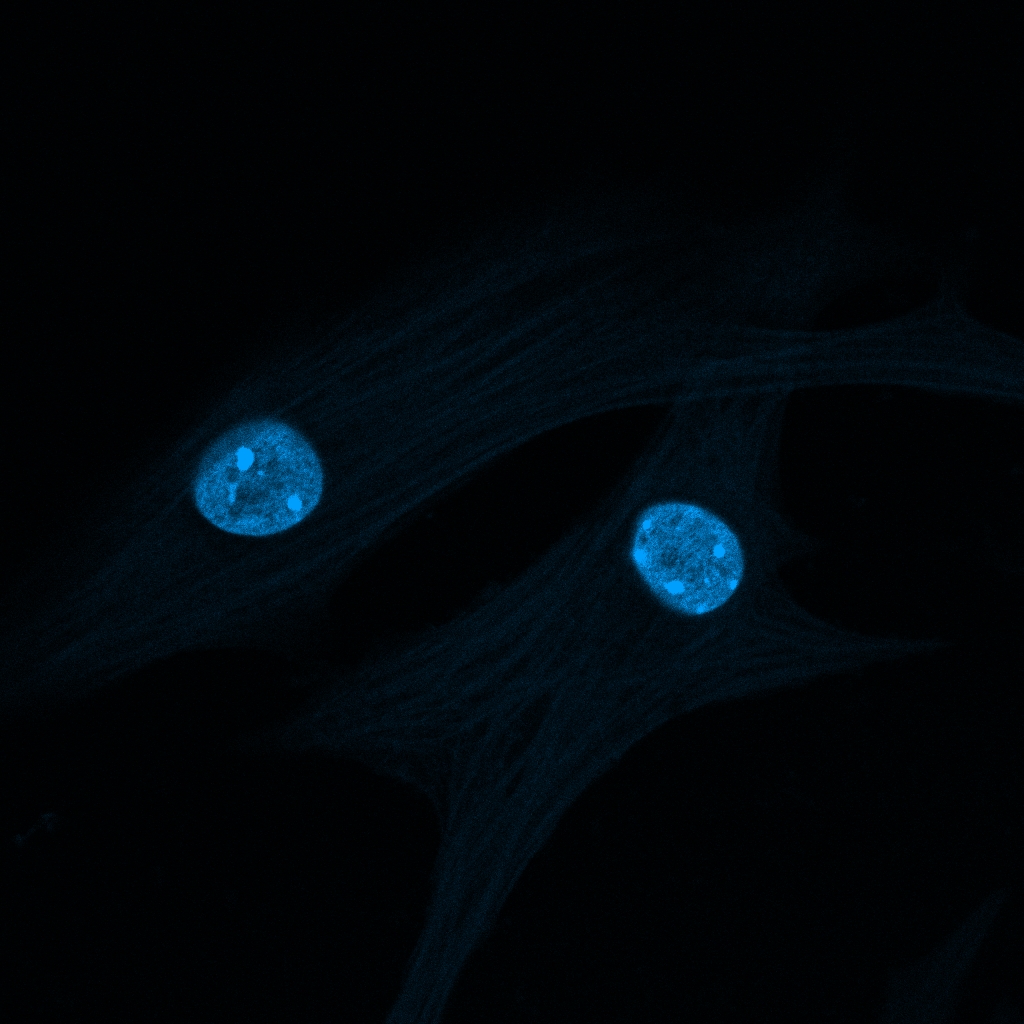

Supplement: Supplementary file 11 — Source data Fig. 8 [file 44321_2025_334_MOESM11_ESM.zip › Figure 8/8J/Ang II+Alpelisib+Ad-RBMS1-DAPI.jpeg]

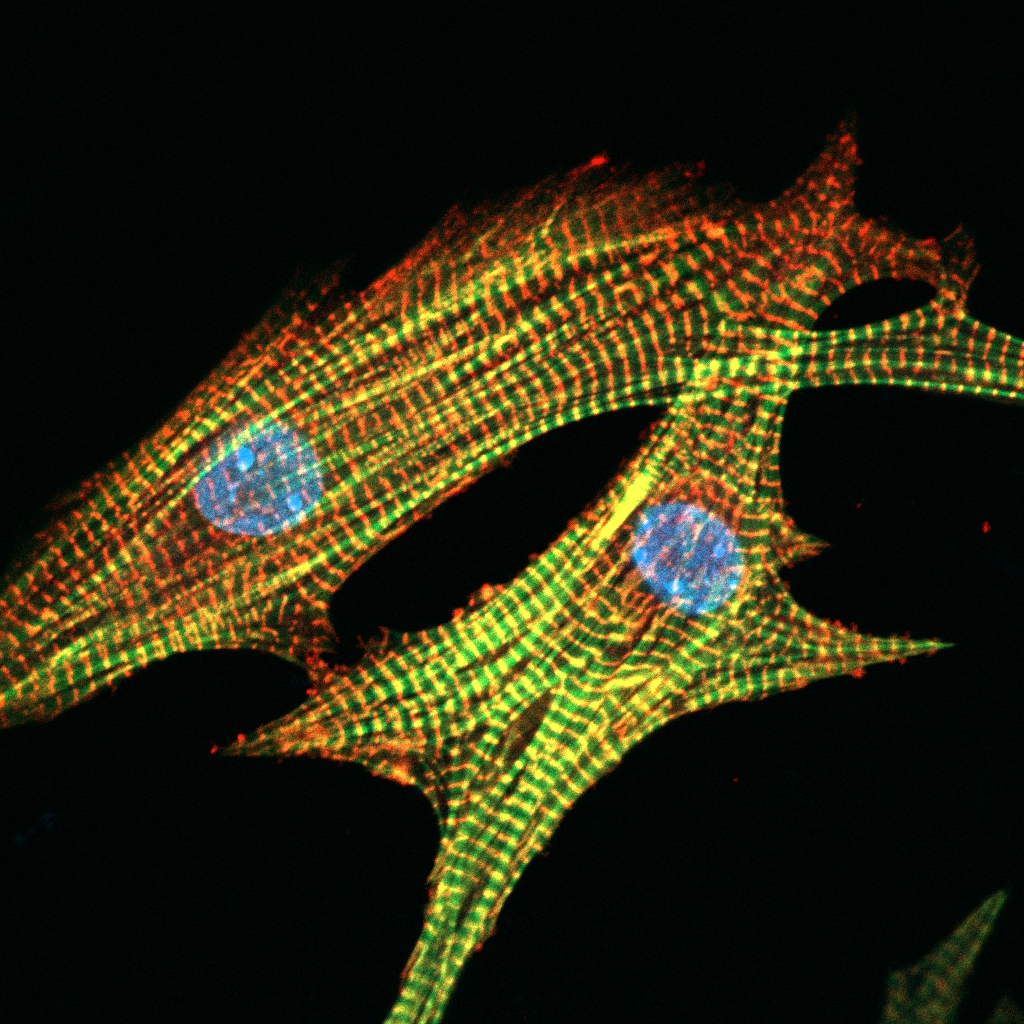

Supplement: Supplementary file 11 — Source data Fig. 8 [file 44321_2025_334_MOESM11_ESM.zip › Figure 8/8J/Ang II+Alpelisib+Ad-RBMS1-Merge.jpeg]

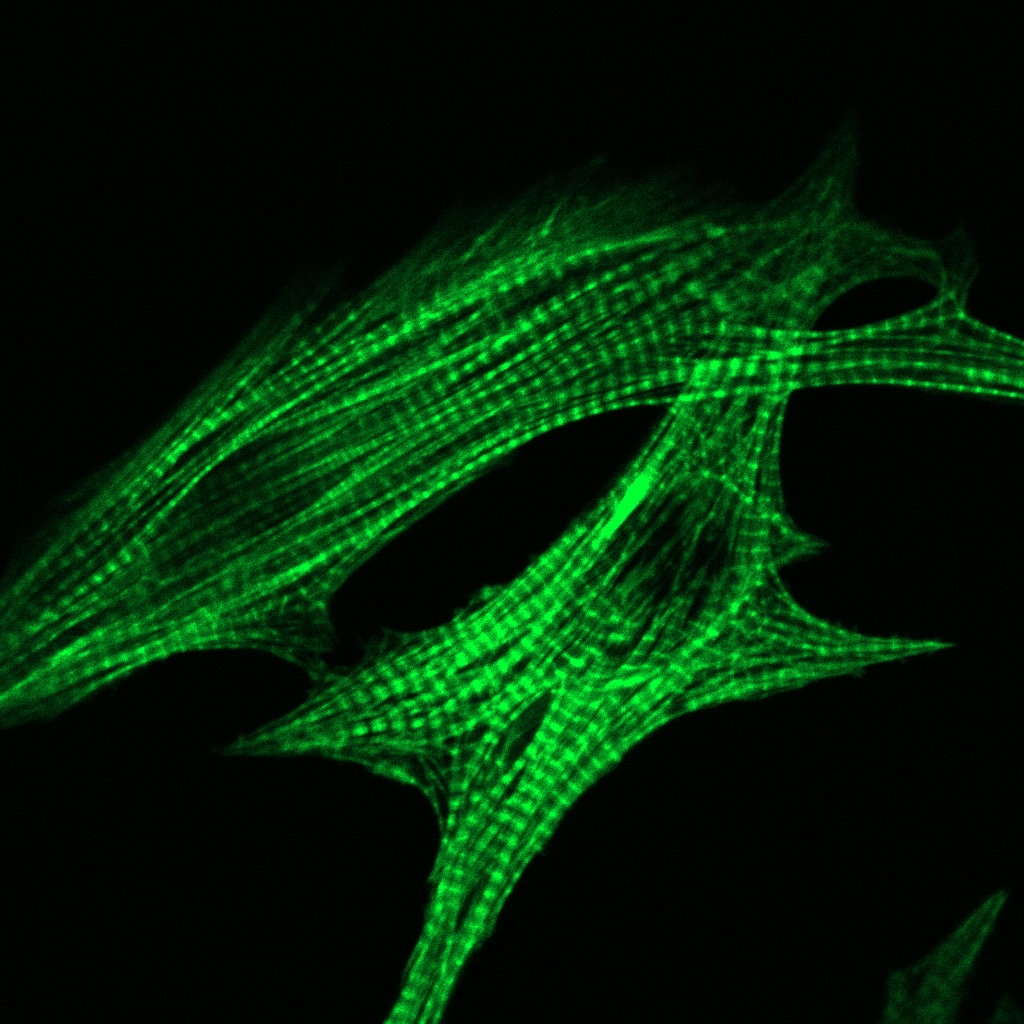

Supplement: Supplementary file 11 — Source data Fig. 8 [file 44321_2025_334_MOESM11_ESM.zip › Figure 8/8J/Ang II+Alpelisib+Ad-RBMS1-Phalloidine.jpeg]

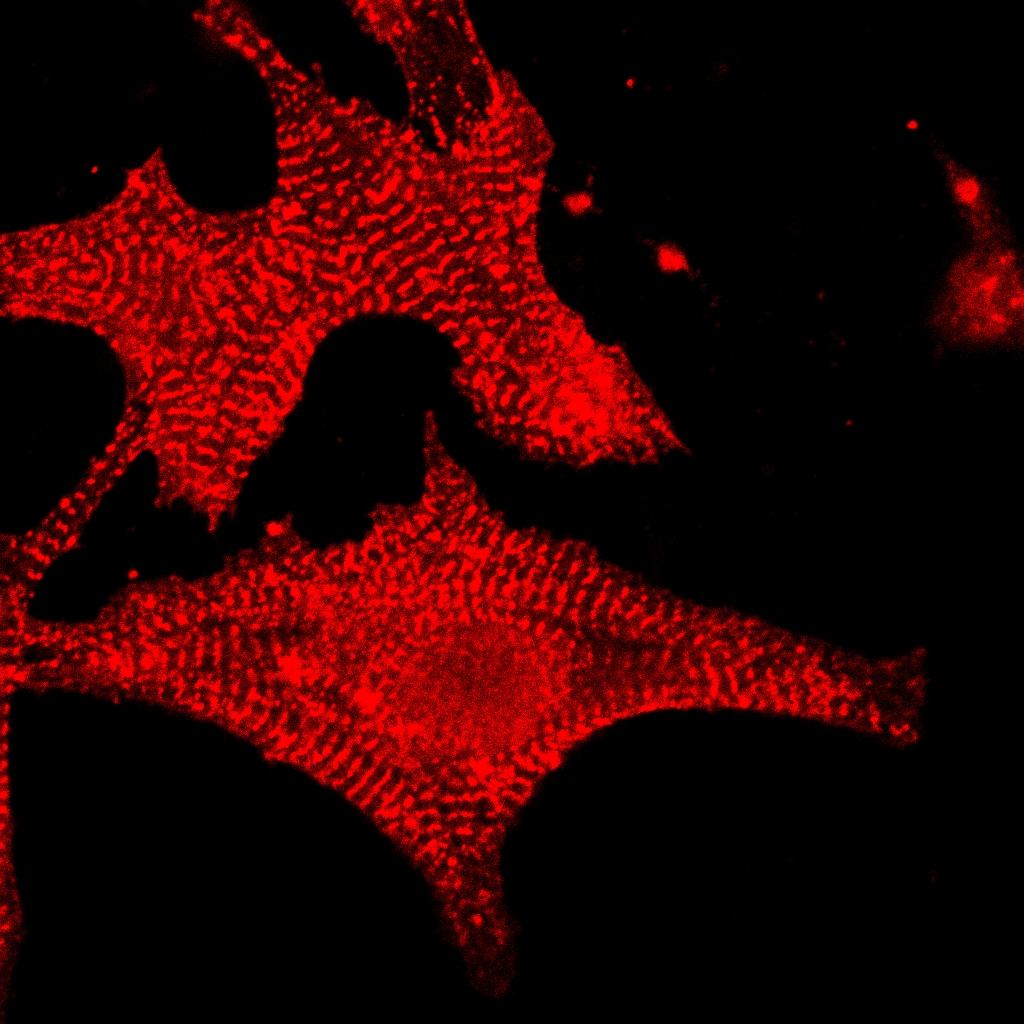

Supplement: Supplementary file 11 — Source data Fig. 8 [file 44321_2025_334_MOESM11_ESM.zip › Figure 8/8J/Ang II+DMSO+Ad-NC-ACTN2.jpeg]

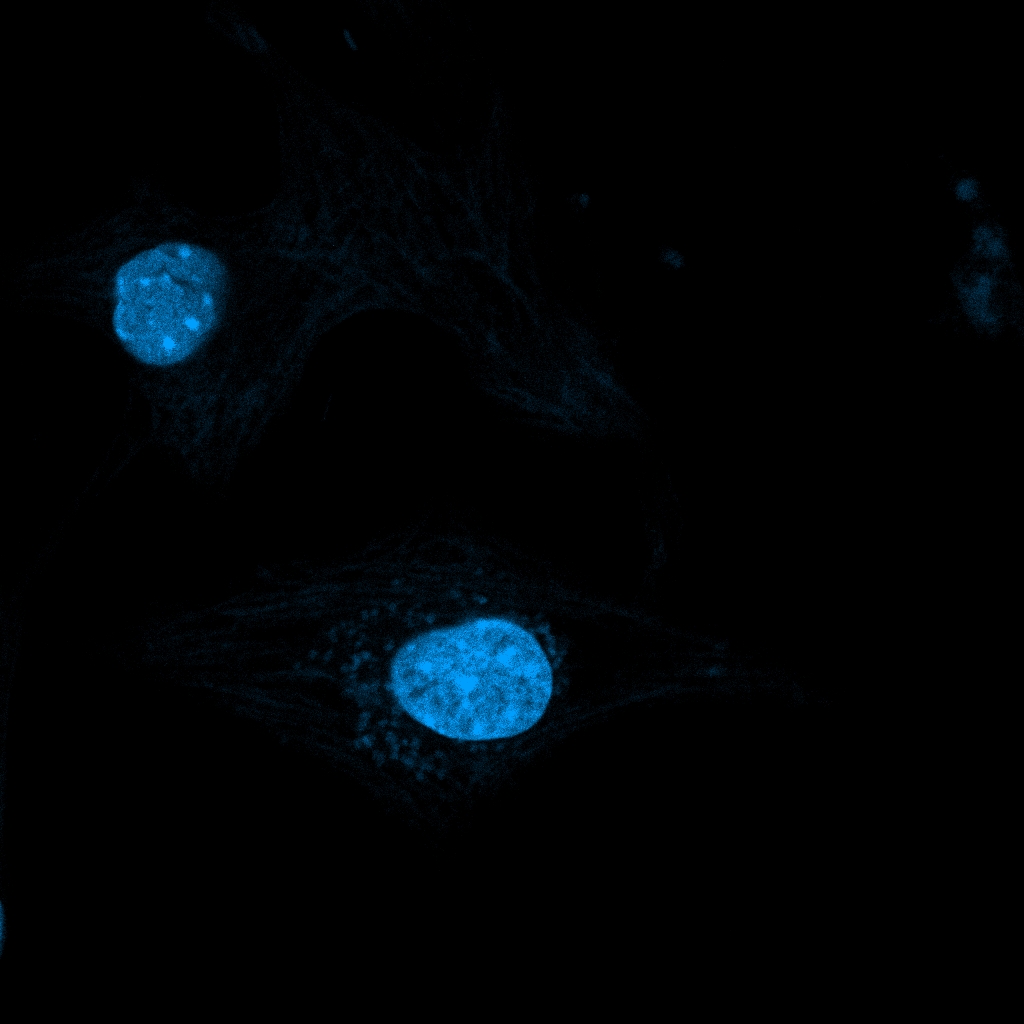

Supplement: Supplementary file 11 — Source data Fig. 8 [file 44321_2025_334_MOESM11_ESM.zip › Figure 8/8J/Ang II+DMSO+Ad-NC-DAPI.jpeg]

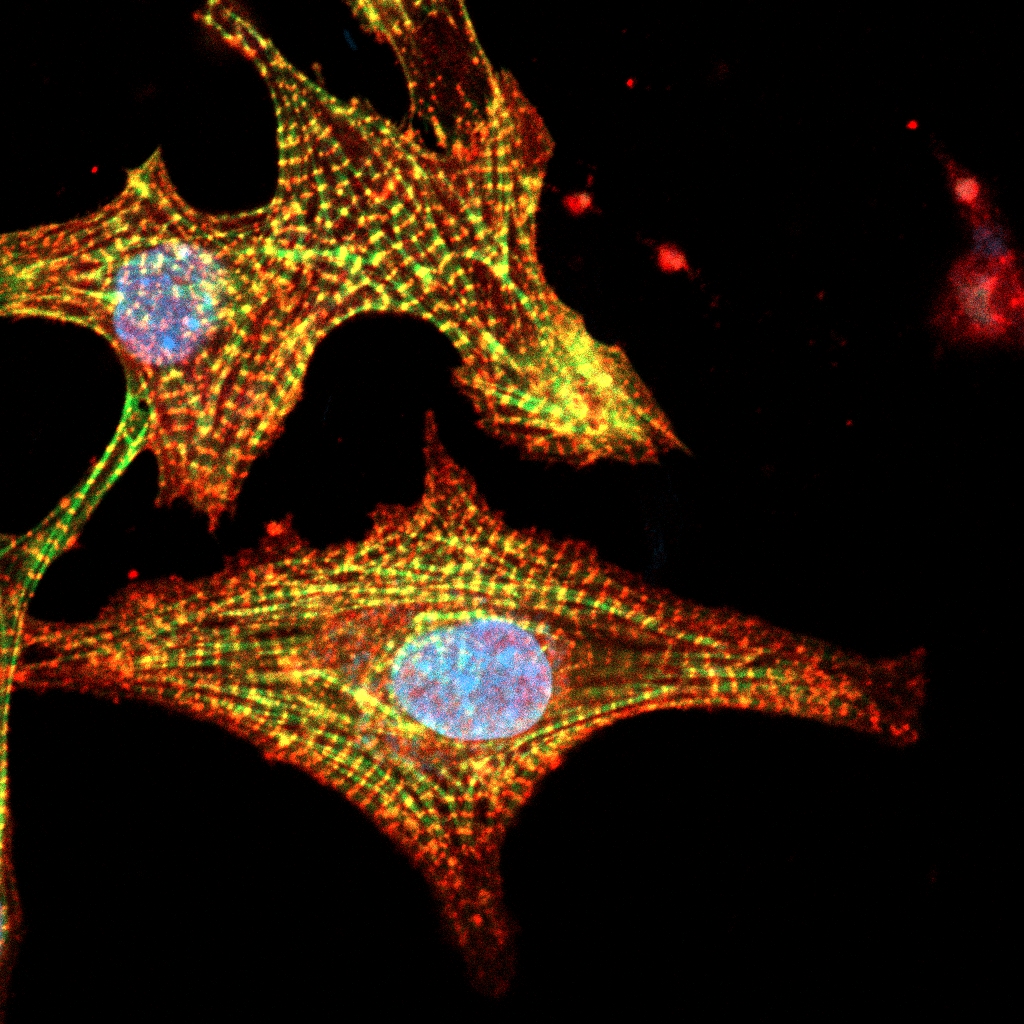

Supplement: Supplementary file 11 — Source data Fig. 8 [file 44321_2025_334_MOESM11_ESM.zip › Figure 8/8J/Ang II+DMSO+Ad-NC-Merge.jpeg]

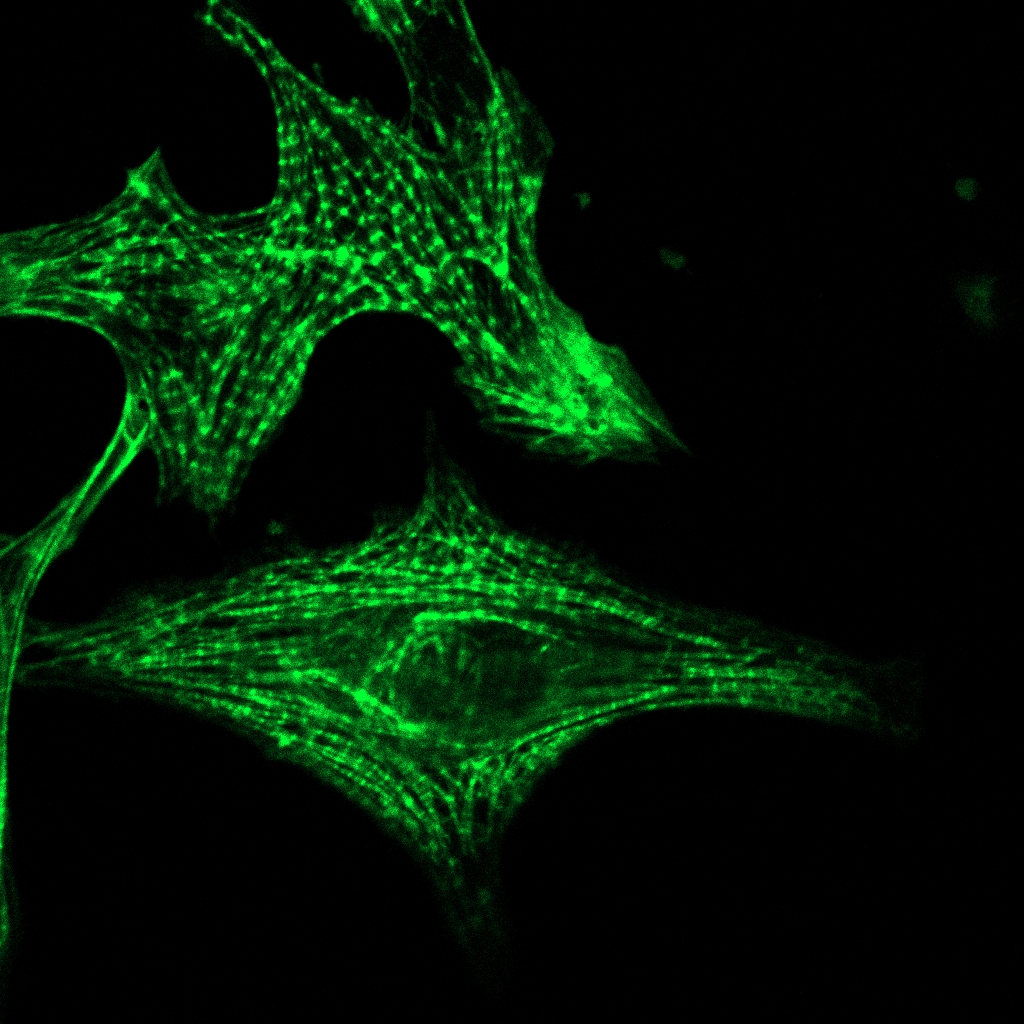

Supplement: Supplementary file 11 — Source data Fig. 8 [file 44321_2025_334_MOESM11_ESM.zip › Figure 8/8J/Ang II+DMSO+Ad-NC-Phalloidine.jpeg]

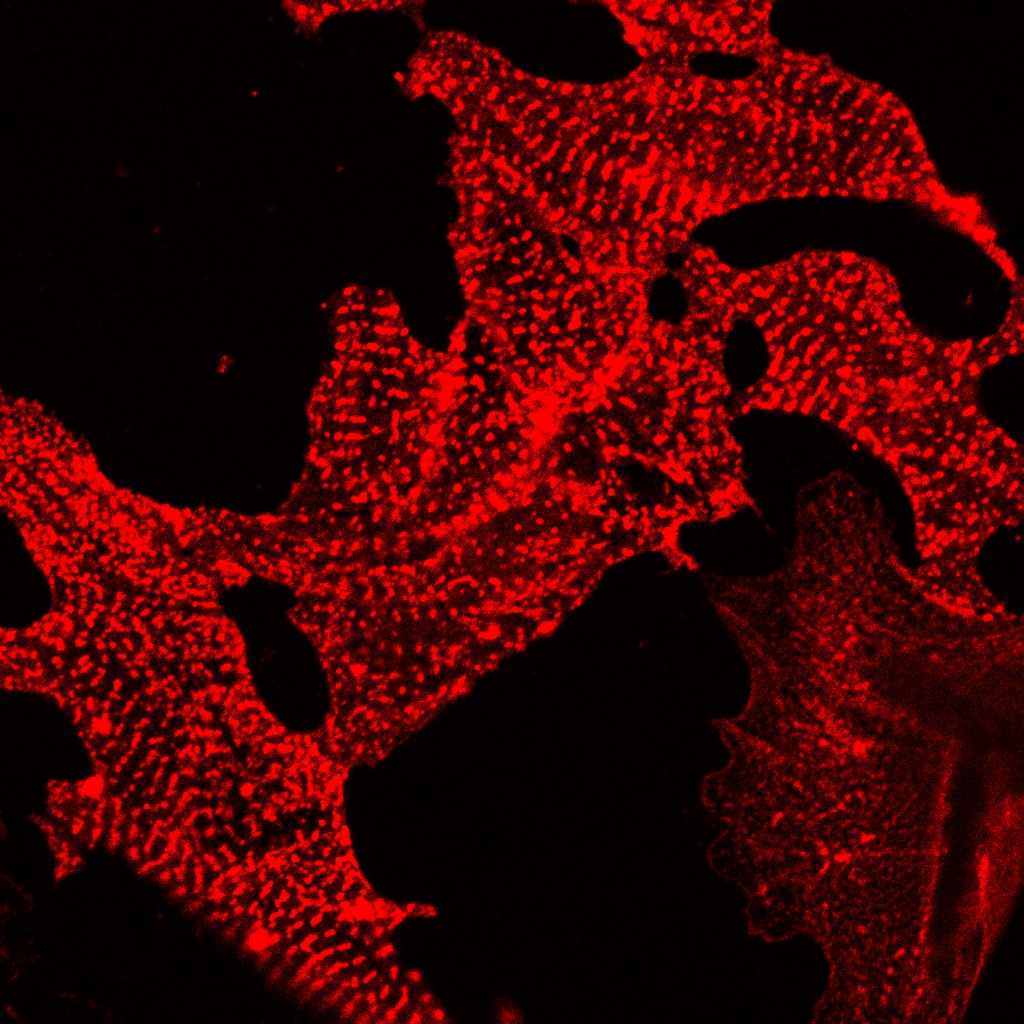

Supplement: Supplementary file 11 — Source data Fig. 8 [file 44321_2025_334_MOESM11_ESM.zip › Figure 8/8J/Ang II+DMSO+Ad-RBMS1-ACTN2.jpeg]

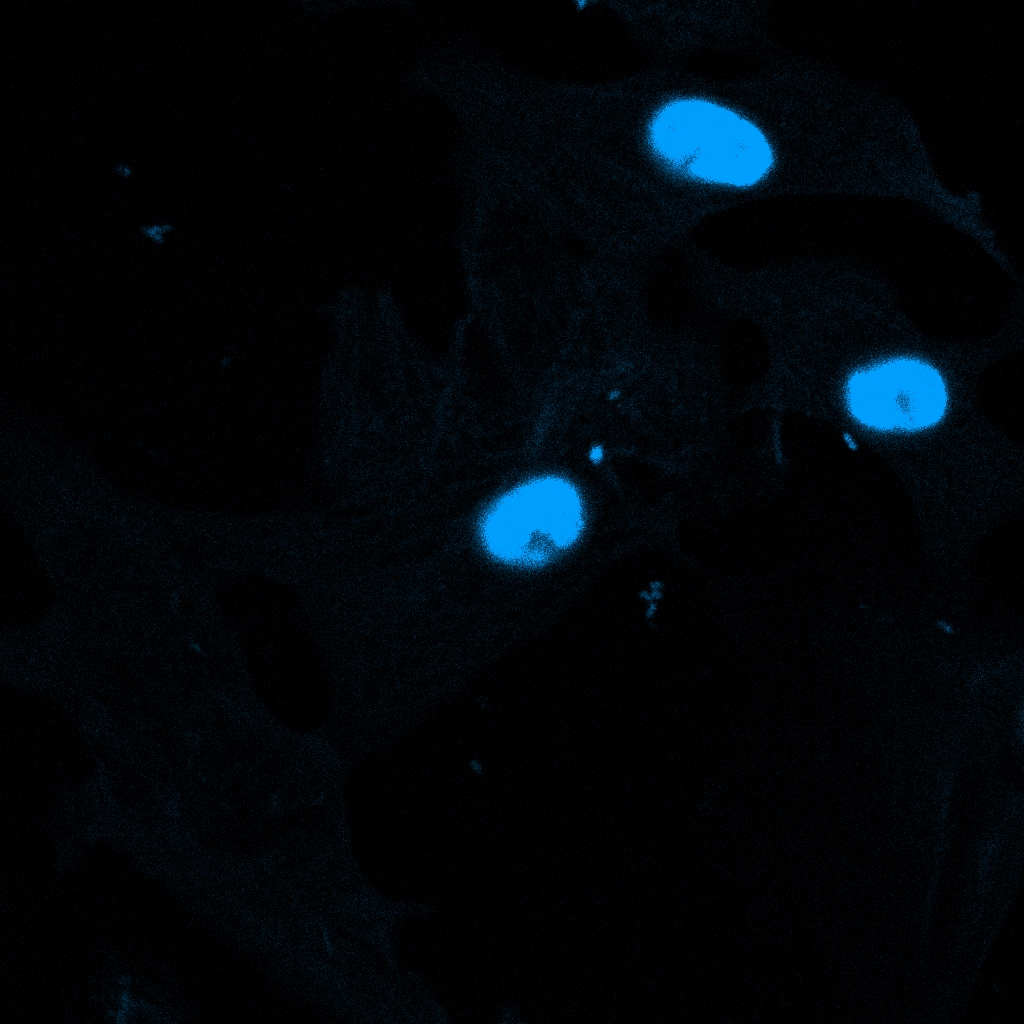

Supplement: Supplementary file 11 — Source data Fig. 8 [file 44321_2025_334_MOESM11_ESM.zip › Figure 8/8J/Ang II+DMSO+Ad-RBMS1-DAPI.jpeg]

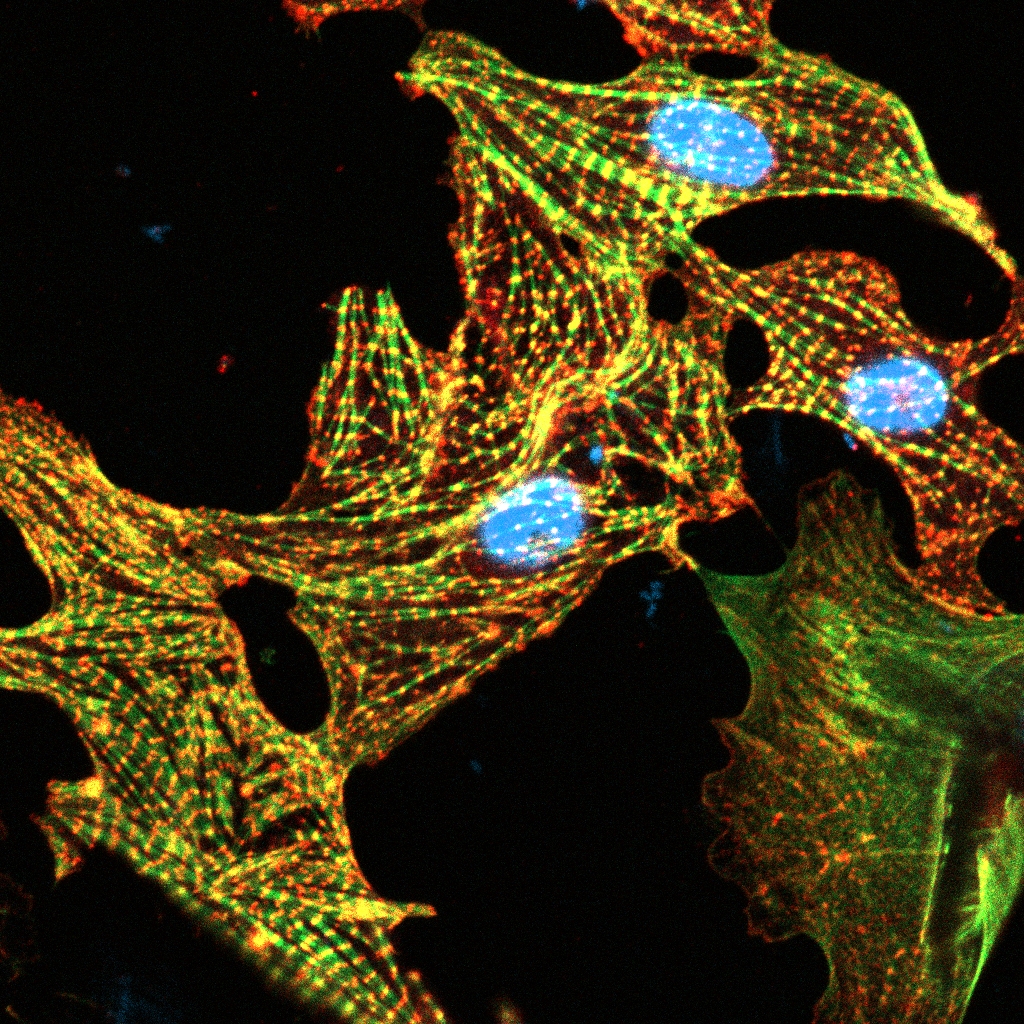

Supplement: Supplementary file 11 — Source data Fig. 8 [file 44321_2025_334_MOESM11_ESM.zip › Figure 8/8J/Ang II+DMSO+Ad-RBMS1-Merge.jpeg]

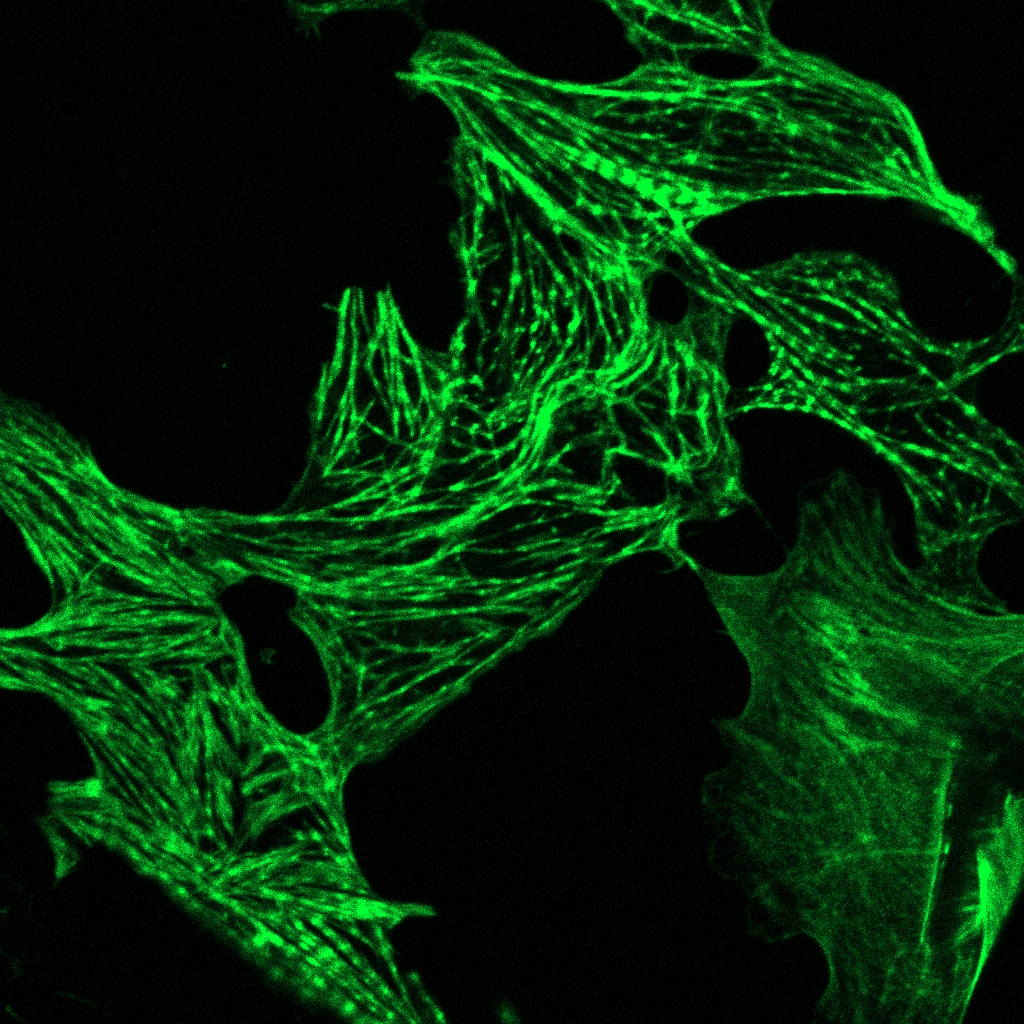

Supplement: Supplementary file 11 — Source data Fig. 8 [file 44321_2025_334_MOESM11_ESM.zip › Figure 8/8J/Ang II+DMSO+Ad-RBMS1-Phalloidine.jpeg]

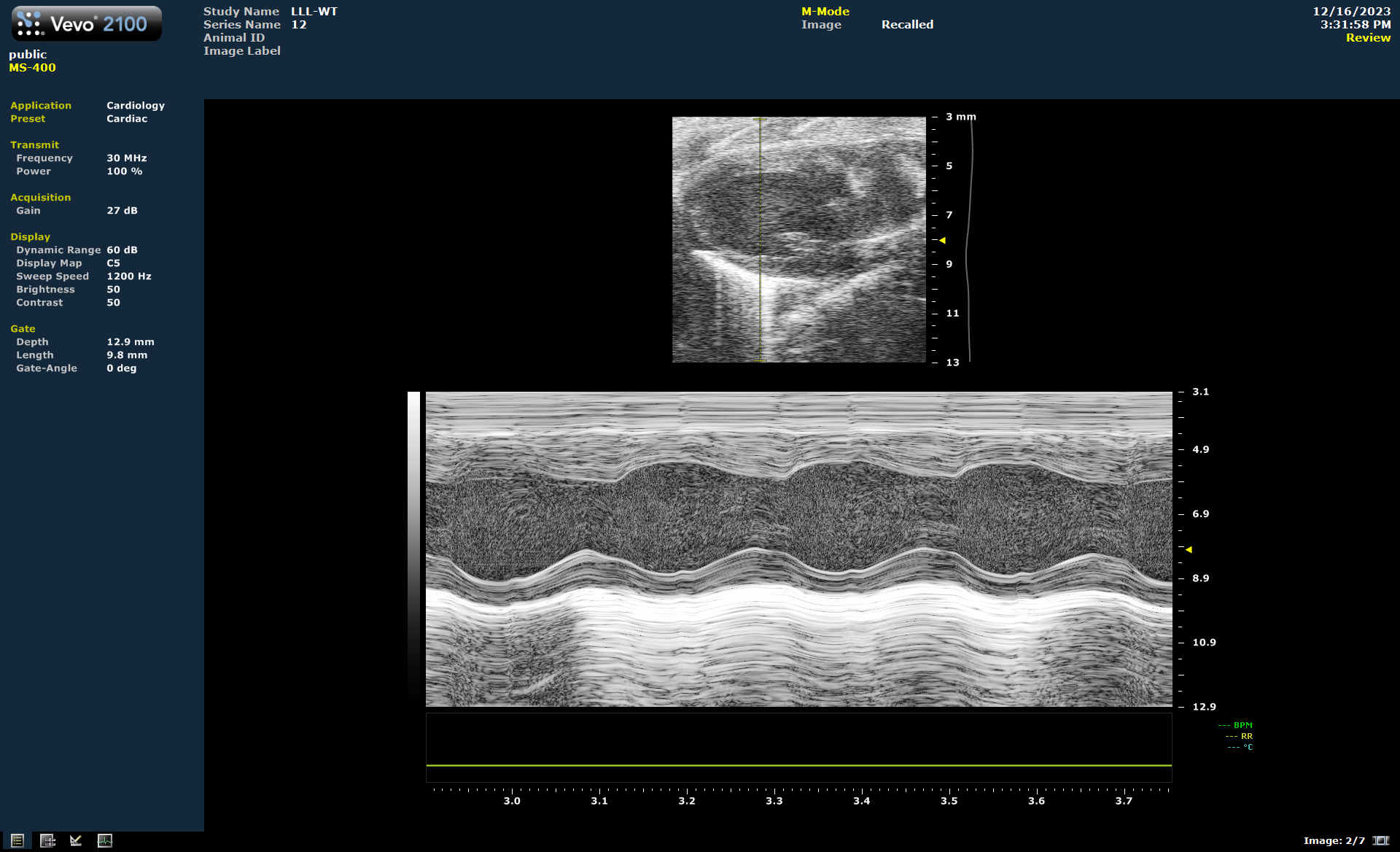

Supplement: Supplementary file 12 — Source data Fig. 9 [file 44321_2025_334_MOESM12_ESM.zip › Figure 9/9A/B Mode/Sham.tif]

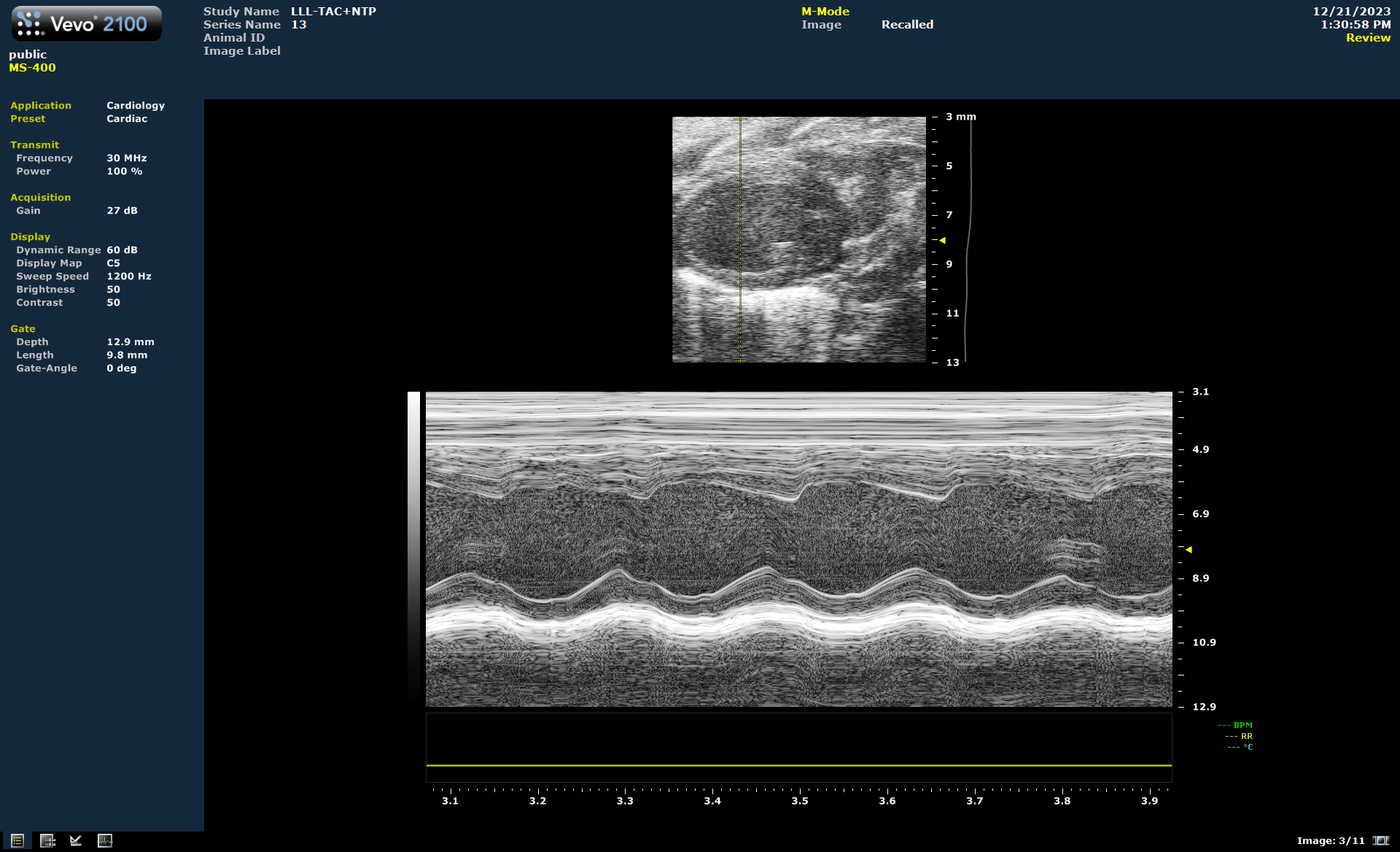

Supplement: Supplementary file 12 — Source data Fig. 9 [file 44321_2025_334_MOESM12_ESM.zip › Figure 9/9A/B Mode/TAC+NTP(5mgkg).tif]

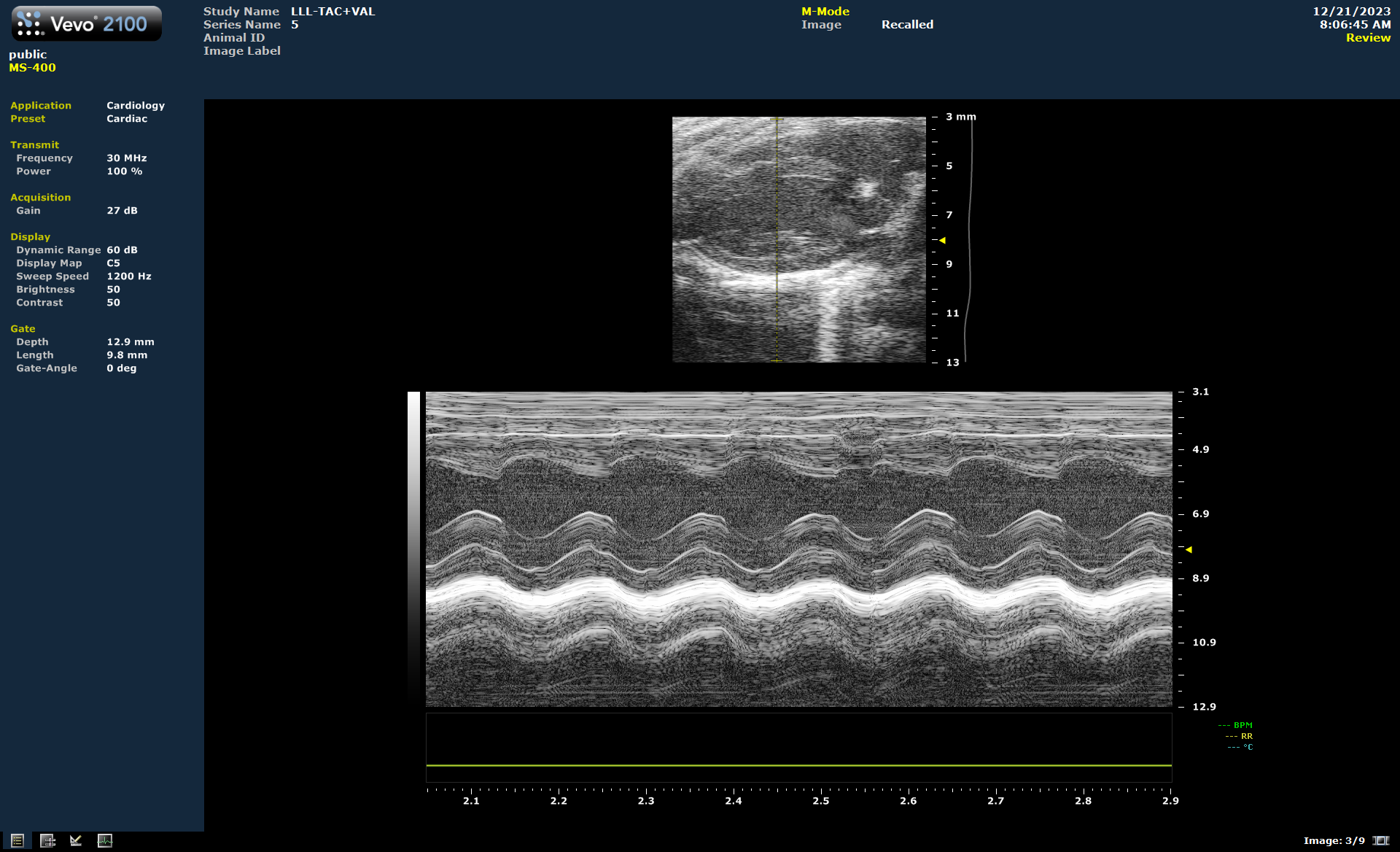

Supplement: Supplementary file 12 — Source data Fig. 9 [file 44321_2025_334_MOESM12_ESM.zip › Figure 9/9A/B Mode/TAC+VAL(10mgkg).tif]

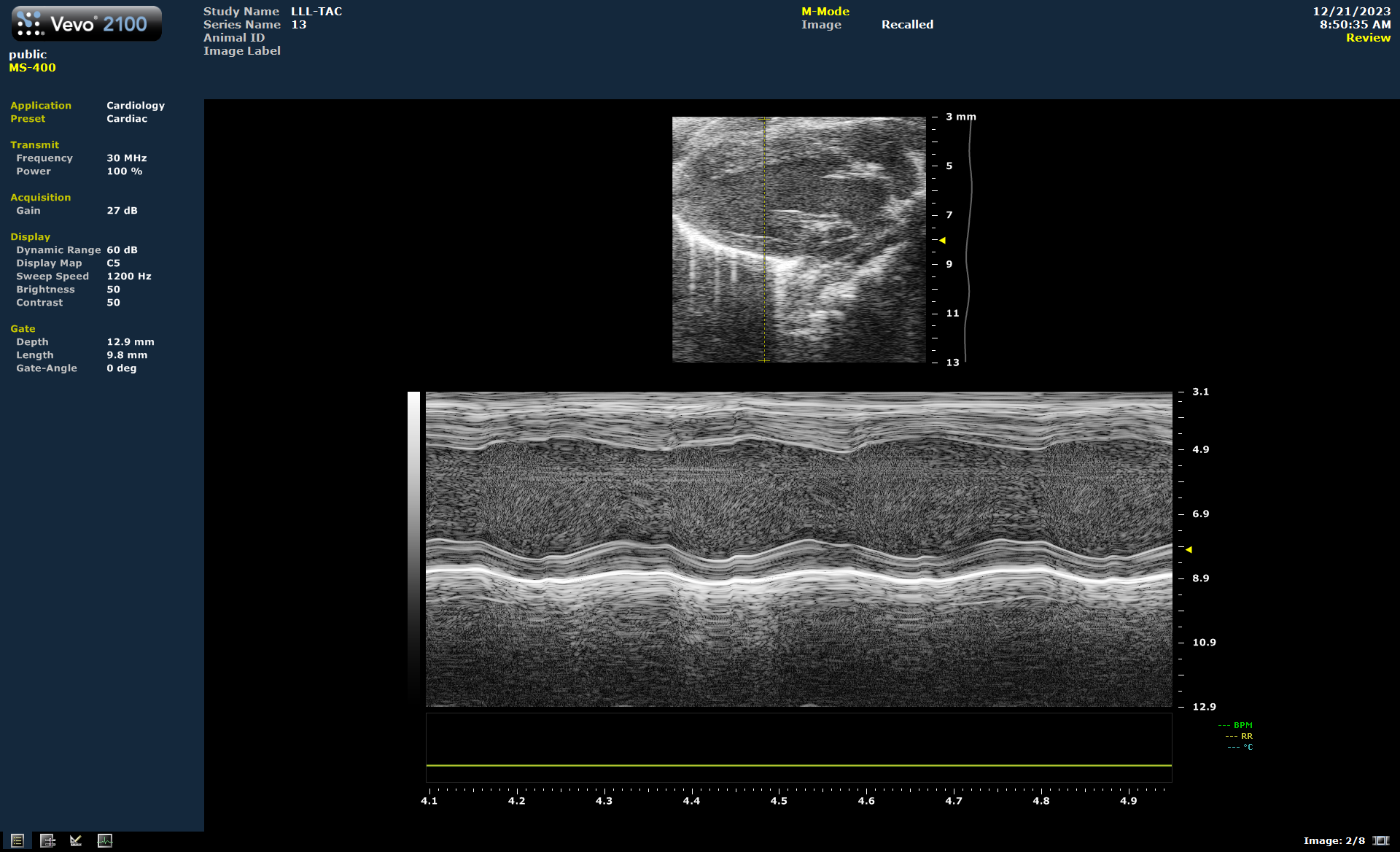

Supplement: Supplementary file 12 — Source data Fig. 9 [file 44321_2025_334_MOESM12_ESM.zip › Figure 9/9A/B Mode/TAC.tif]

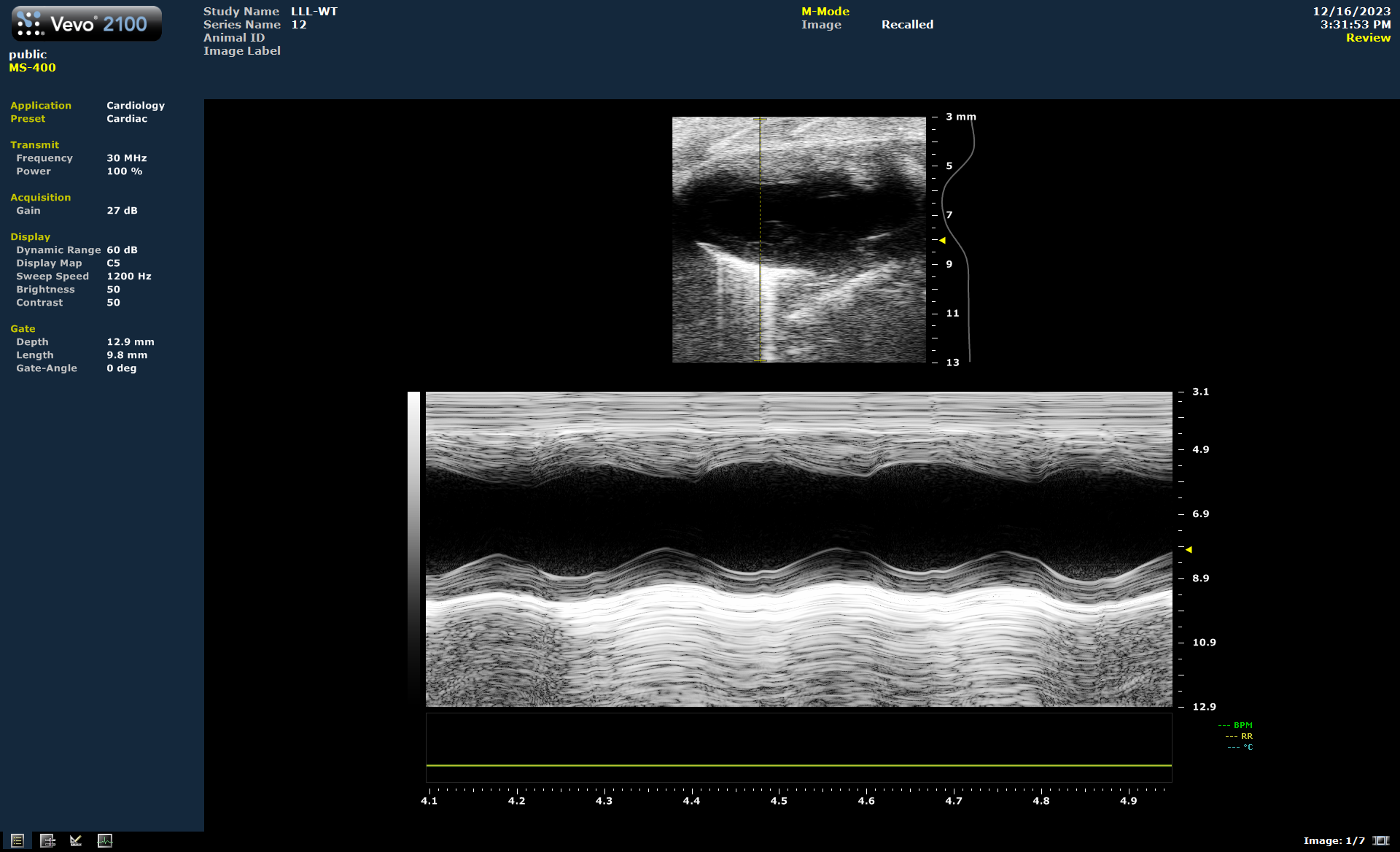

Supplement: Supplementary file 12 — Source data Fig. 9 [file 44321_2025_334_MOESM12_ESM.zip › Figure 9/9A/M Mode/Sham.tif]

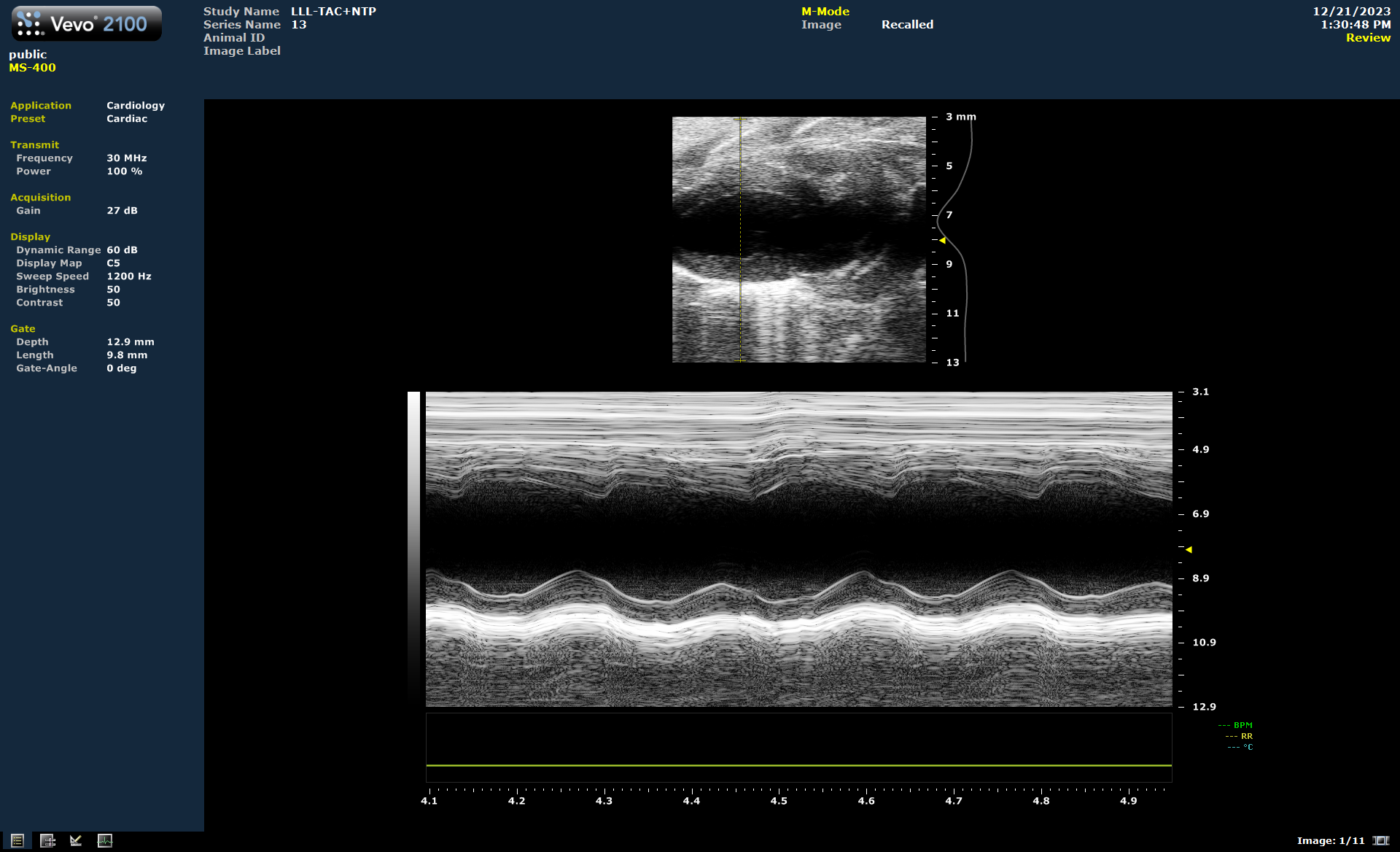

Supplement: Supplementary file 12 — Source data Fig. 9 [file 44321_2025_334_MOESM12_ESM.zip › Figure 9/9A/M Mode/TAC+NTP(5mgkg).tif]

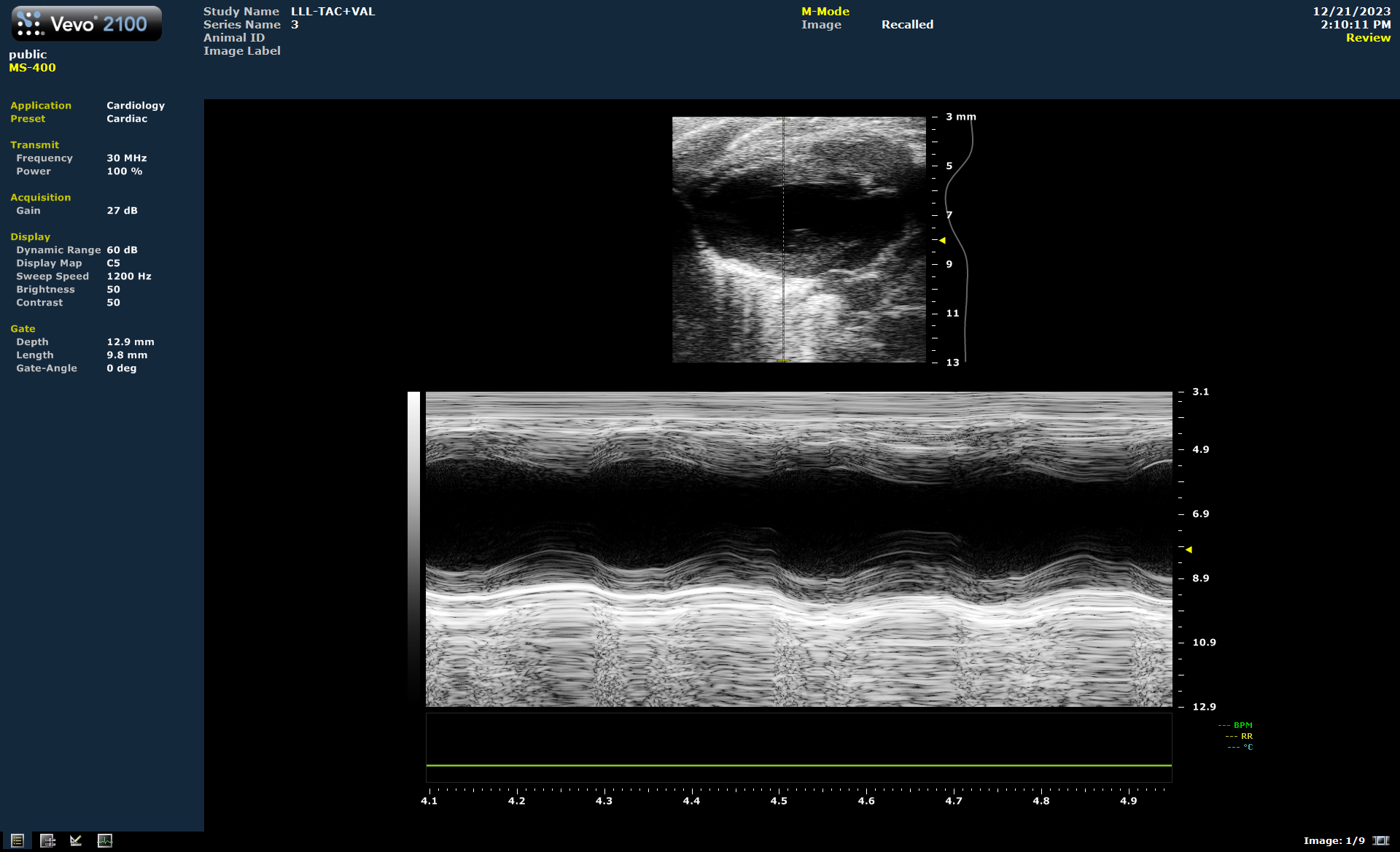

Supplement: Supplementary file 12 — Source data Fig. 9 [file 44321_2025_334_MOESM12_ESM.zip › Figure 9/9A/M Mode/TAC+VAL(5mgkg).tif]

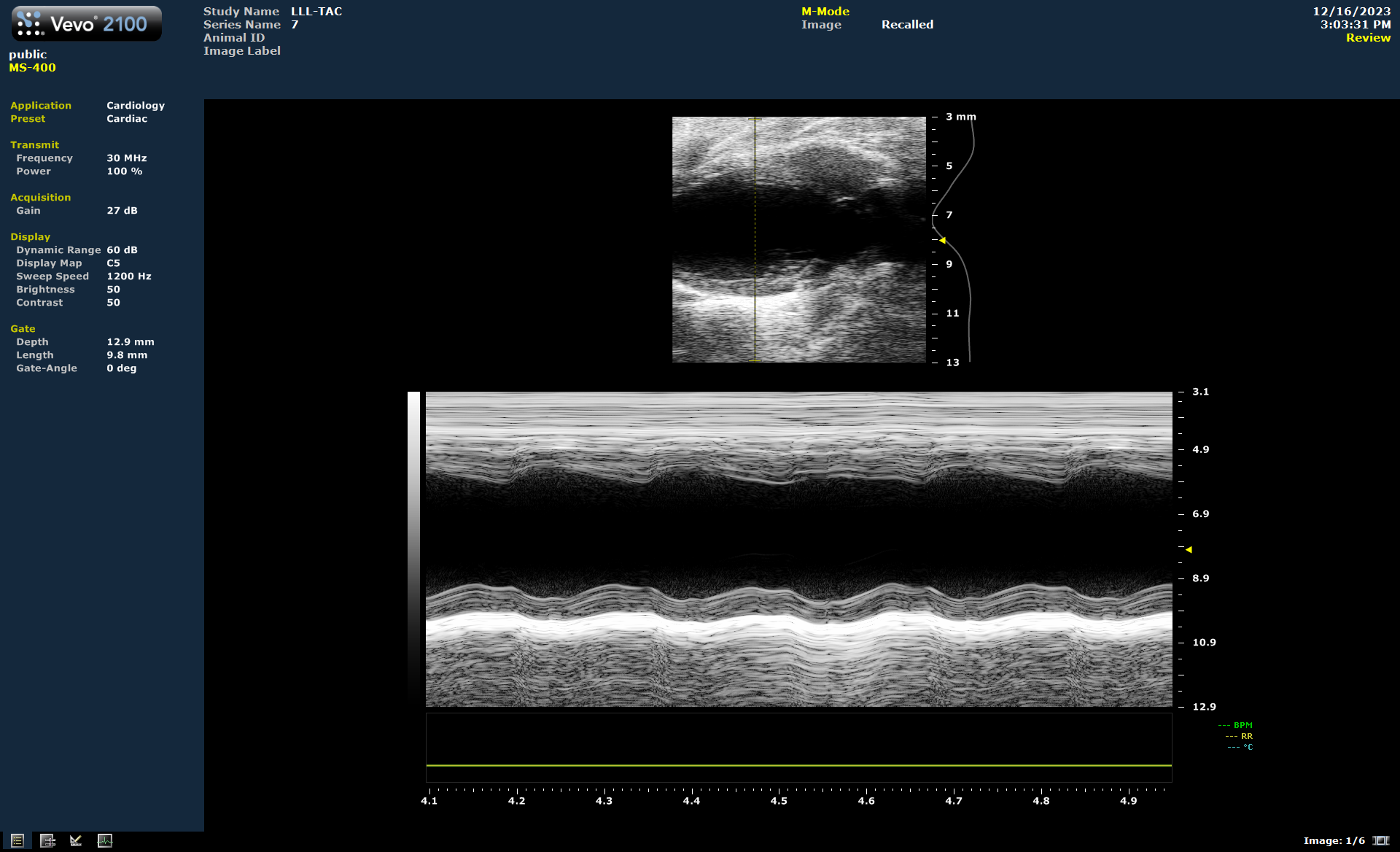

Supplement: Supplementary file 12 — Source data Fig. 9 [file 44321_2025_334_MOESM12_ESM.zip › Figure 9/9A/M Mode/TAC.tif]

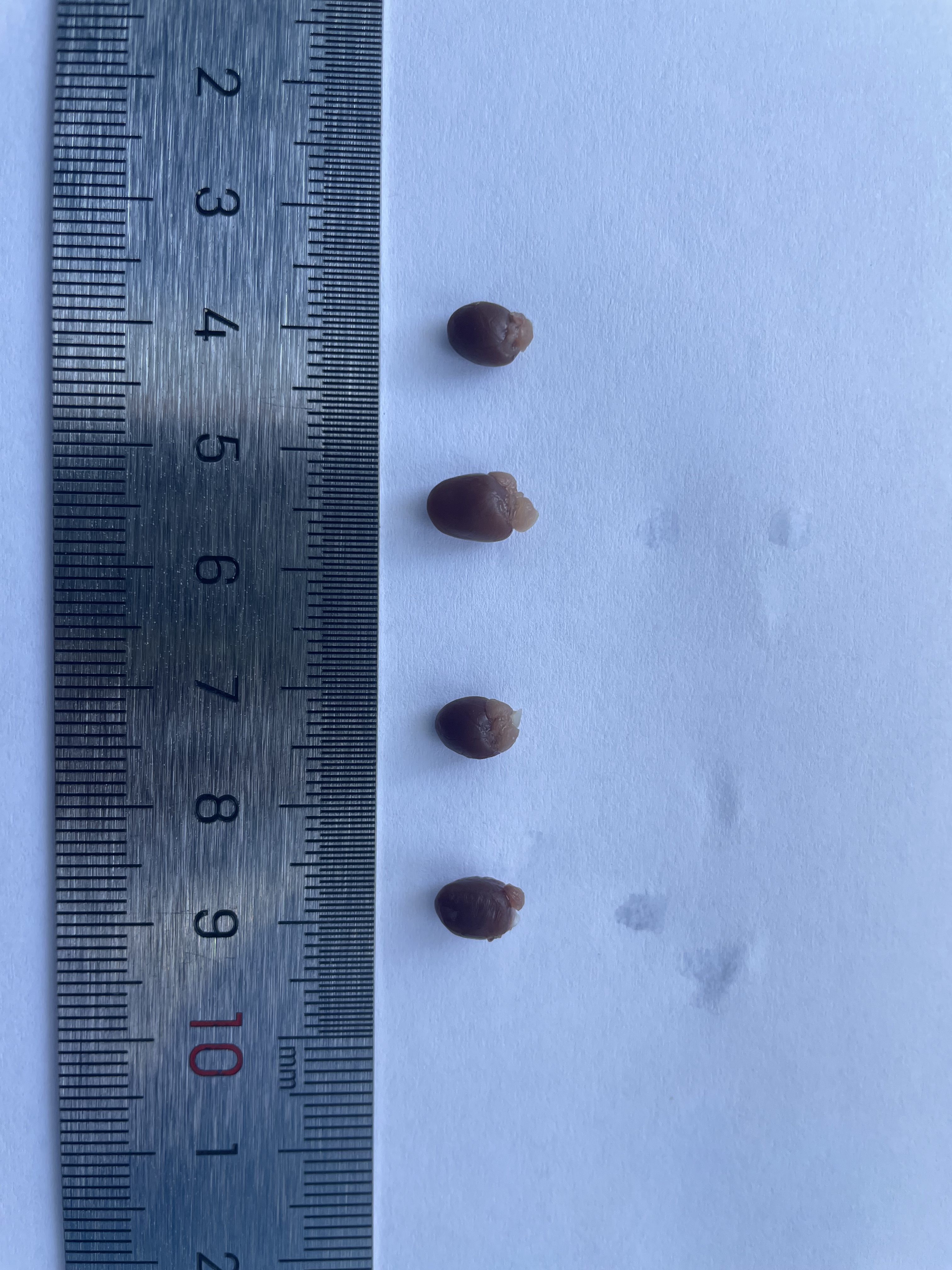

Supplement: Supplementary file 12 — Source data Fig. 9 [file 44321_2025_334_MOESM12_ESM.zip › Figure 9/9D/9D.JPG]
