# Supplementary figures and images for: RBMS1 orchestrates cardiac hypertrophy by facilitating CTTN splice-switching and sarcomere dynamics (part 4 of 4)
Source: EMBO Mol Med. 2025 Nov 10;17(12):3555–85. doi: 10.1038/s44321-025-00334-z (PMC12686484; doi:10.1038/s44321-025-00334-z)

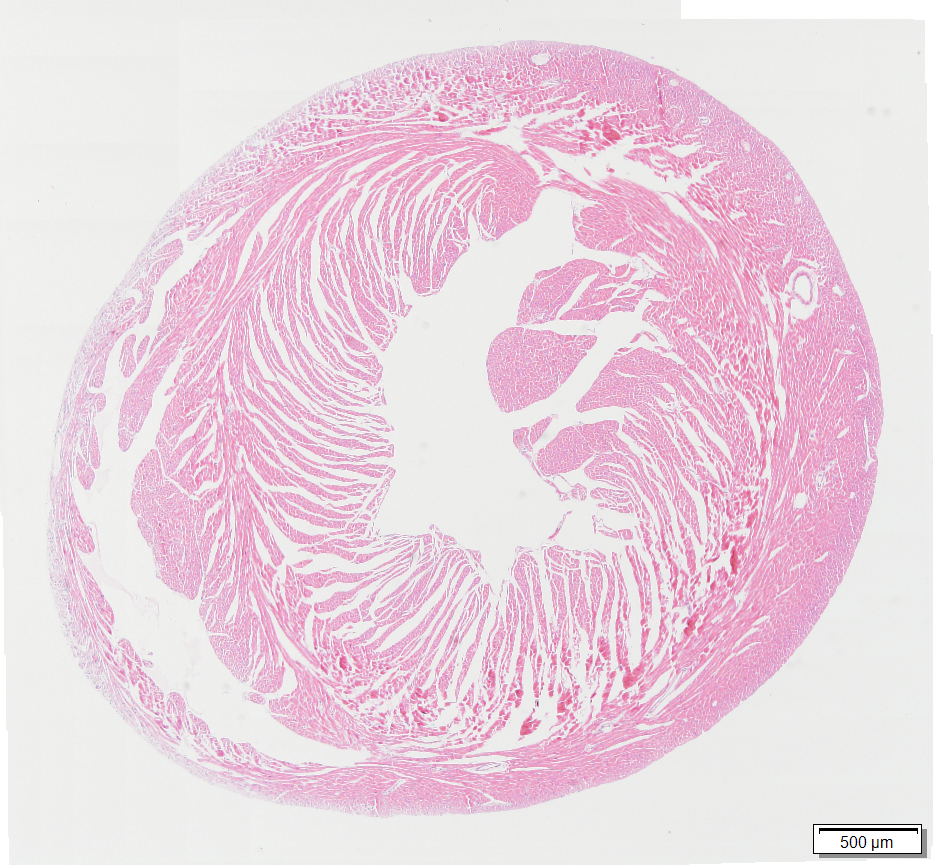

Supplement: Supplementary file 12 — Source data Fig. 9 [file 44321_2025_334_MOESM12_ESM.zip › Figure 9/9F/Cross/Sham.tif]

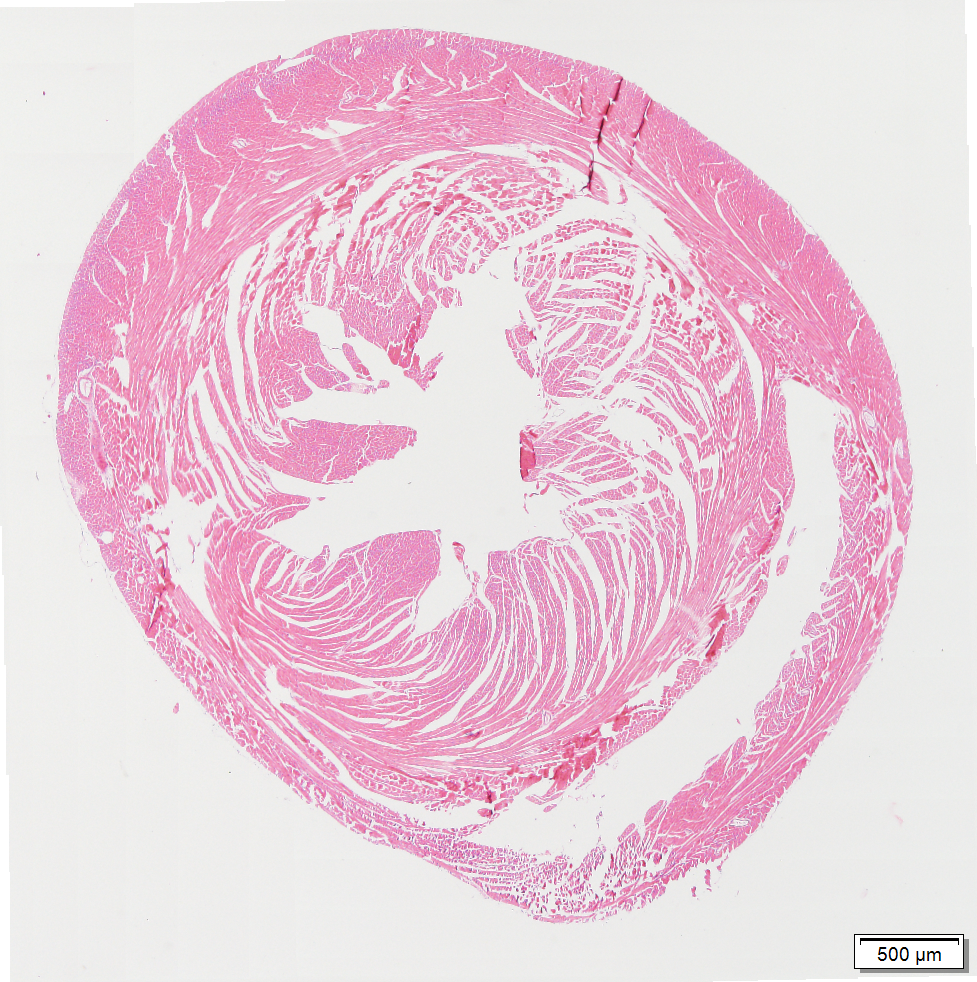

Supplement: Supplementary file 12 — Source data Fig. 9 [file 44321_2025_334_MOESM12_ESM.zip › Figure 9/9F/Cross/TAC+NTP.tif]

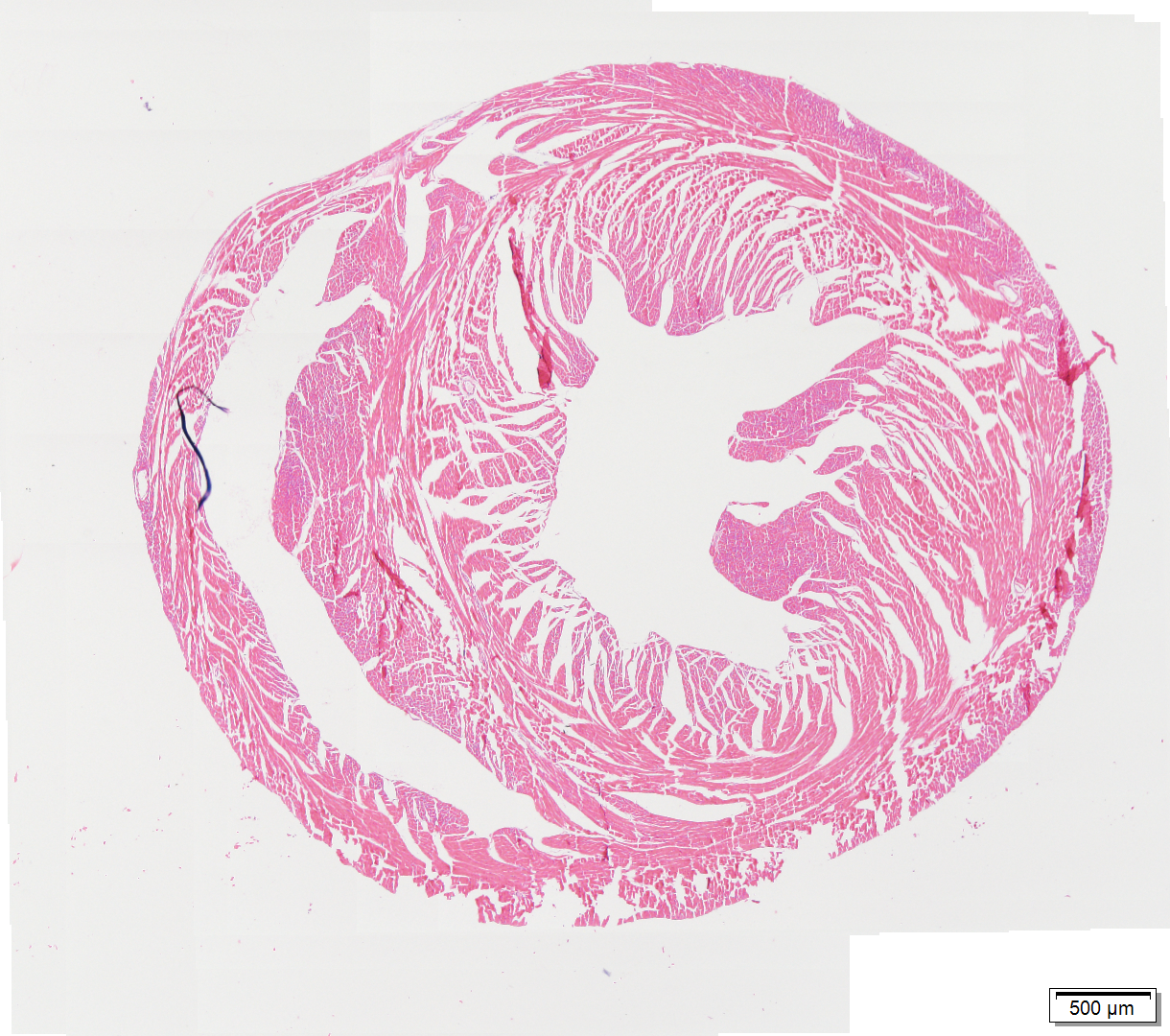

Supplement: Supplementary file 12 — Source data Fig. 9 [file 44321_2025_334_MOESM12_ESM.zip › Figure 9/9F/Cross/TAC+VAL.tif]

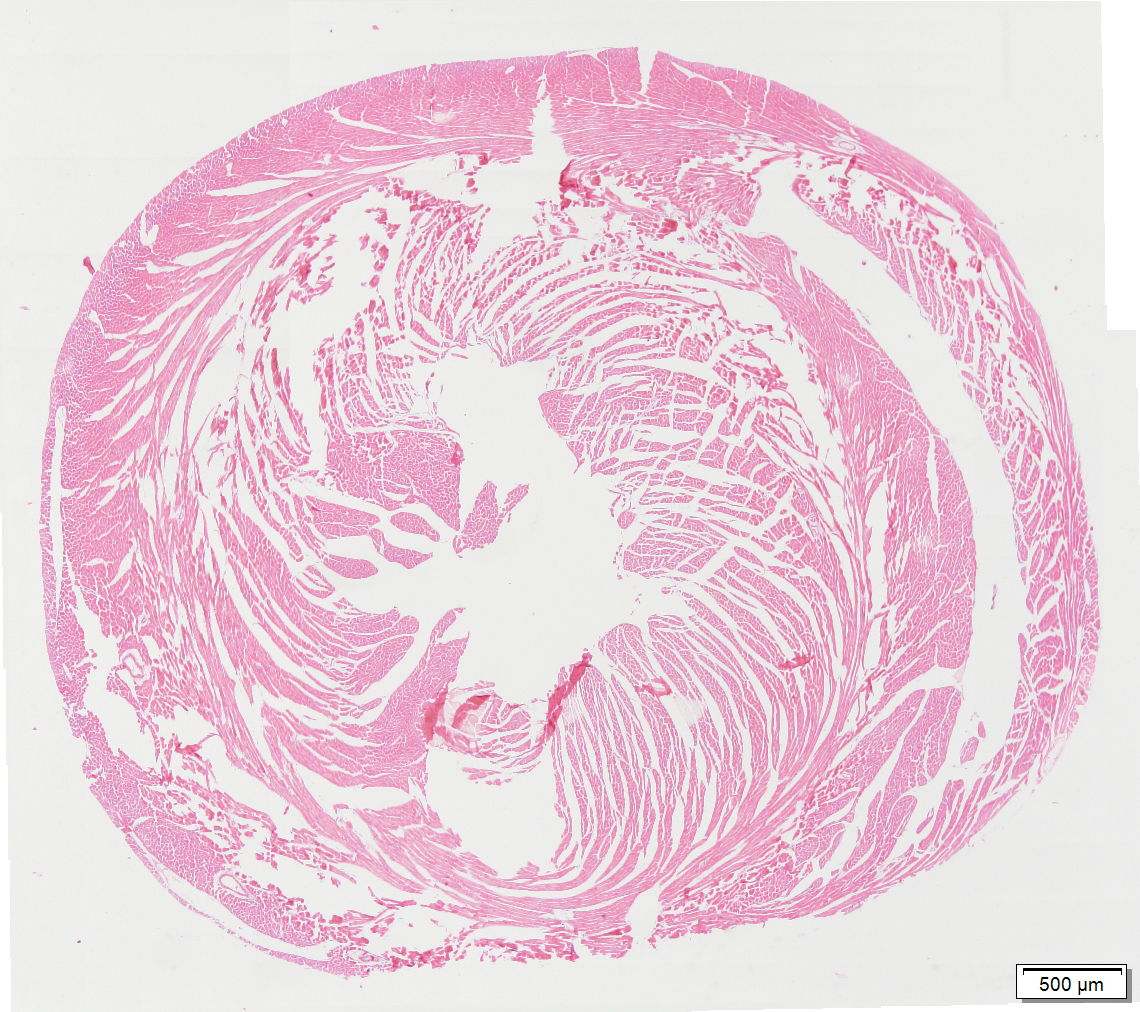

Supplement: Supplementary file 12 — Source data Fig. 9 [file 44321_2025_334_MOESM12_ESM.zip › Figure 9/9F/Cross/TAC.tif]

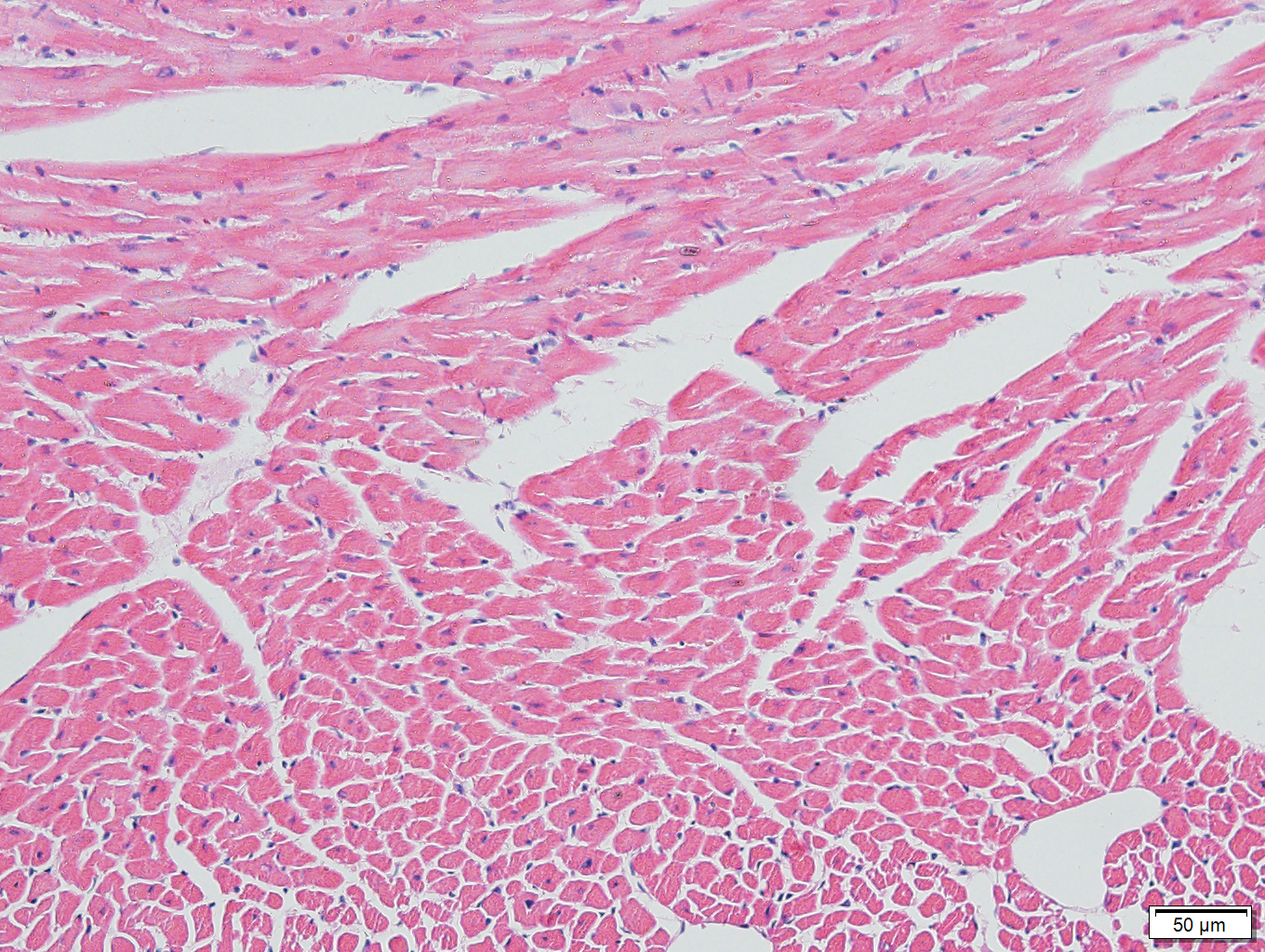

Supplement: Supplementary file 12 — Source data Fig. 9 [file 44321_2025_334_MOESM12_ESM.zip › Figure 9/9F/H&E/Sham.tif]

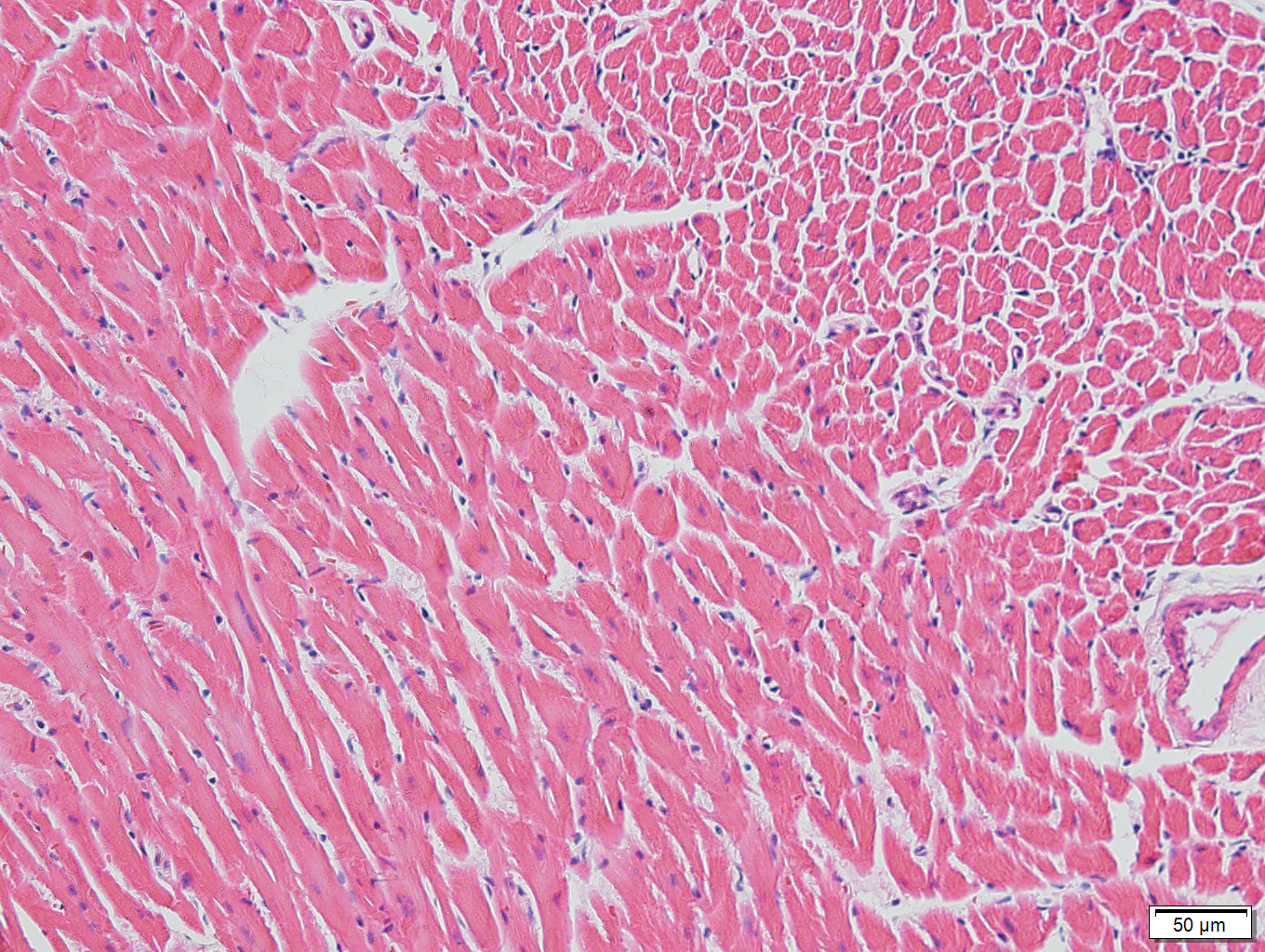

Supplement: Supplementary file 12 — Source data Fig. 9 [file 44321_2025_334_MOESM12_ESM.zip › Figure 9/9F/H&E/TAC+NTP.tif]

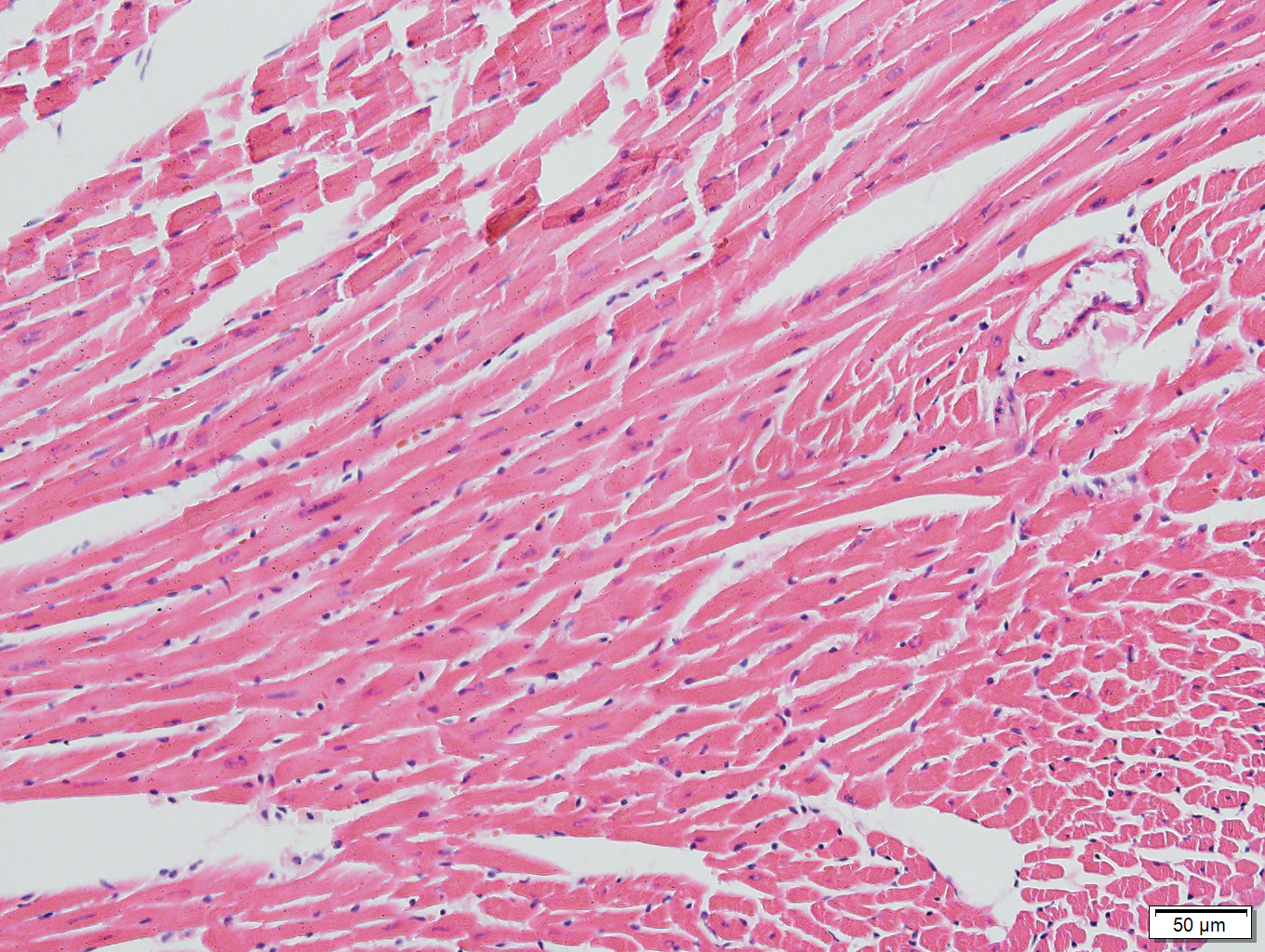

Supplement: Supplementary file 12 — Source data Fig. 9 [file 44321_2025_334_MOESM12_ESM.zip › Figure 9/9F/H&E/TAC+VAL.tif]

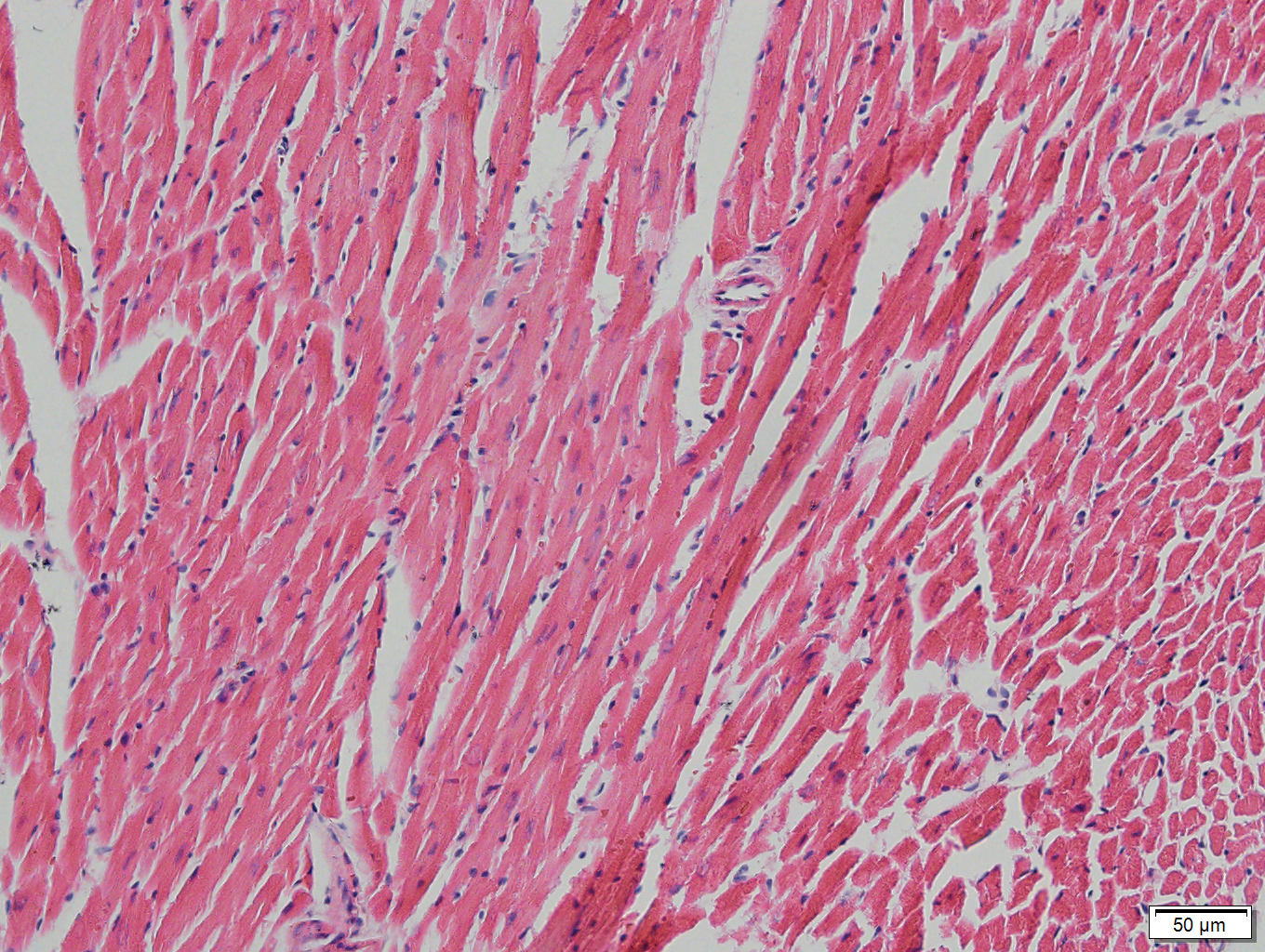

Supplement: Supplementary file 12 — Source data Fig. 9 [file 44321_2025_334_MOESM12_ESM.zip › Figure 9/9F/H&E/TAC.tif]

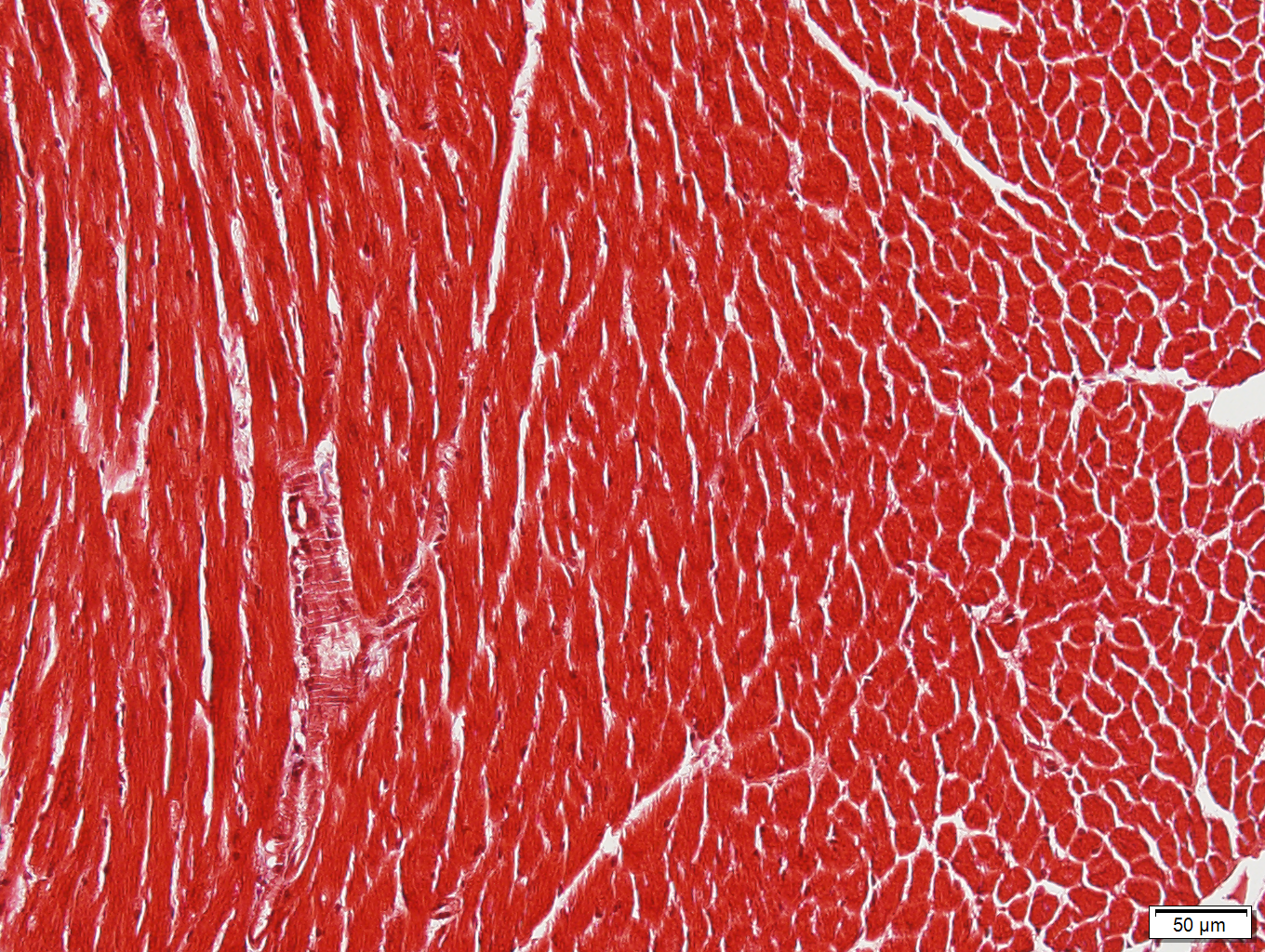

Supplement: Supplementary file 12 — Source data Fig. 9 [file 44321_2025_334_MOESM12_ESM.zip › Figure 9/9G/Interstital/Sham.tif]

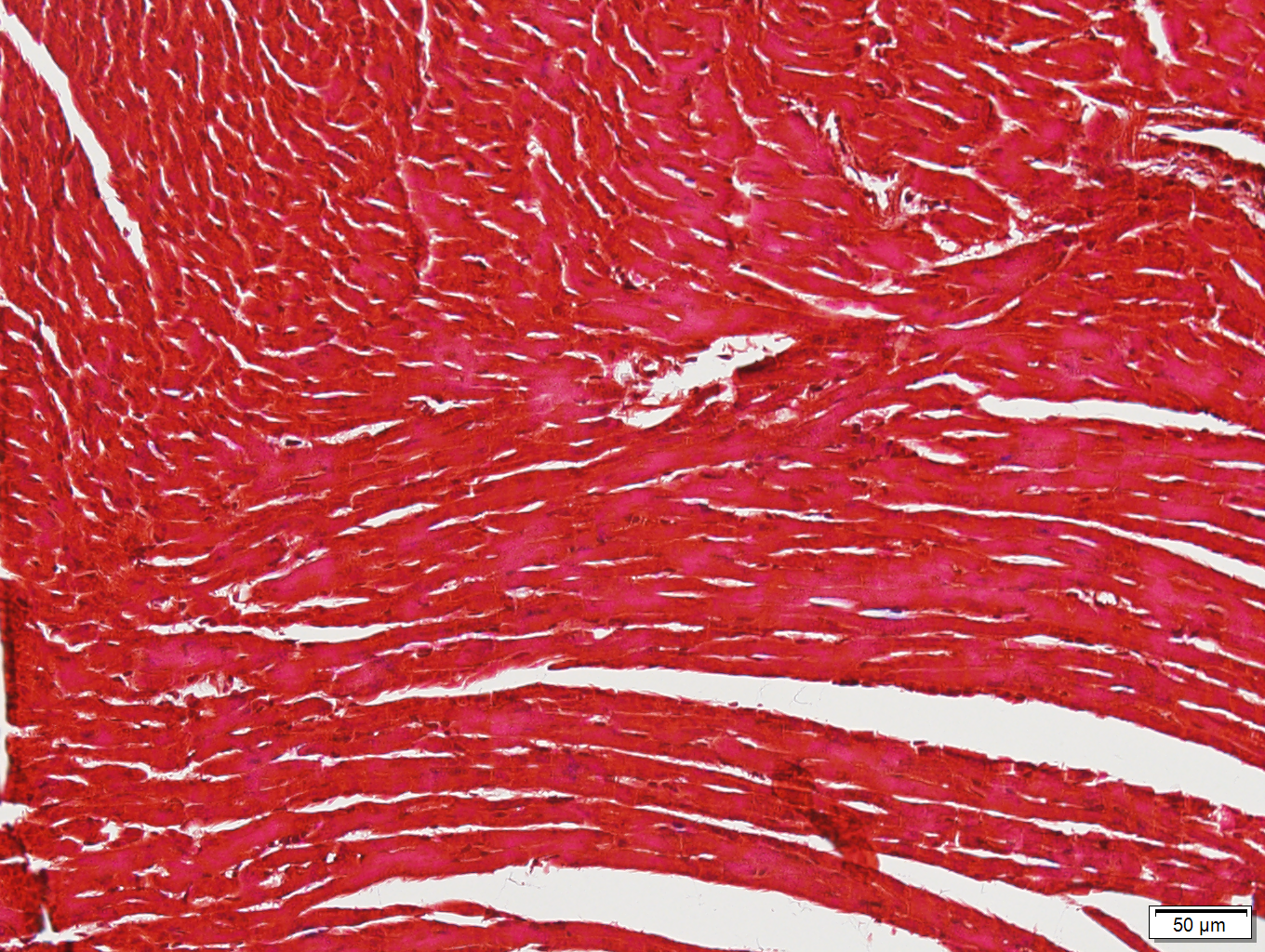

Supplement: Supplementary file 12 — Source data Fig. 9 [file 44321_2025_334_MOESM12_ESM.zip › Figure 9/9G/Interstital/TAC+NTP.tif]

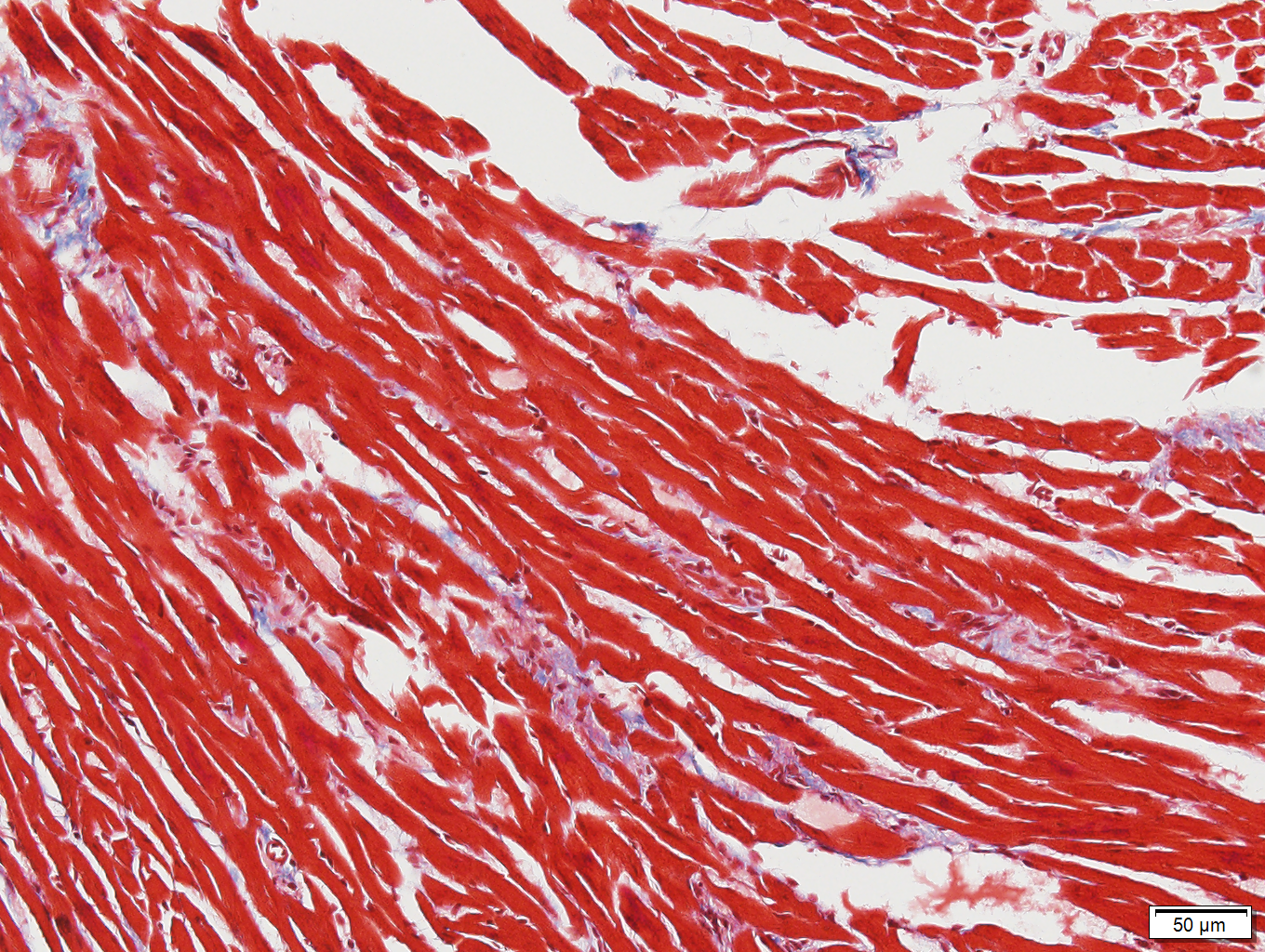

Supplement: Supplementary file 12 — Source data Fig. 9 [file 44321_2025_334_MOESM12_ESM.zip › Figure 9/9G/Interstital/TAC+VAL.tif]

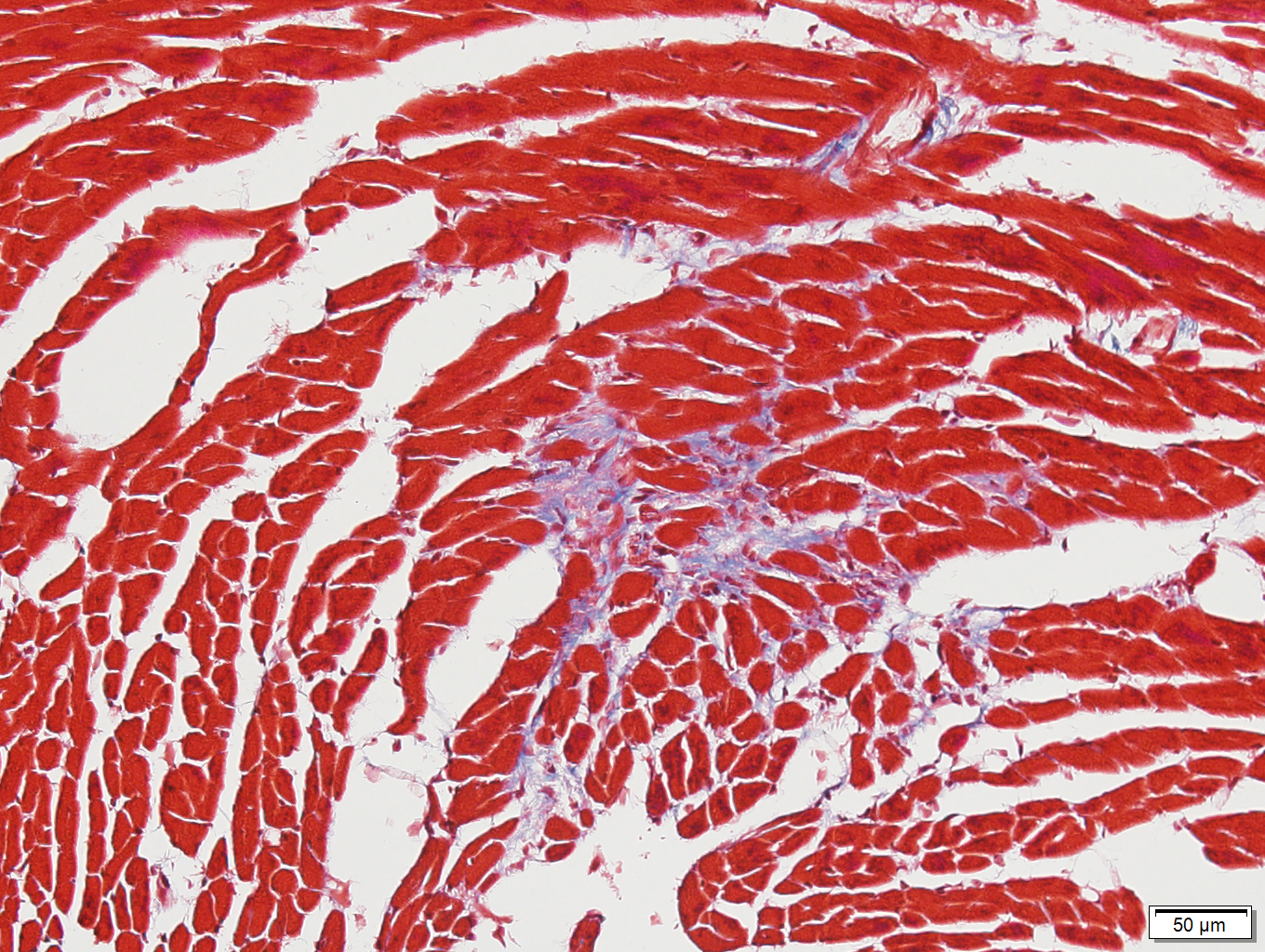

Supplement: Supplementary file 12 — Source data Fig. 9 [file 44321_2025_334_MOESM12_ESM.zip › Figure 9/9G/Interstital/TAC.tif]

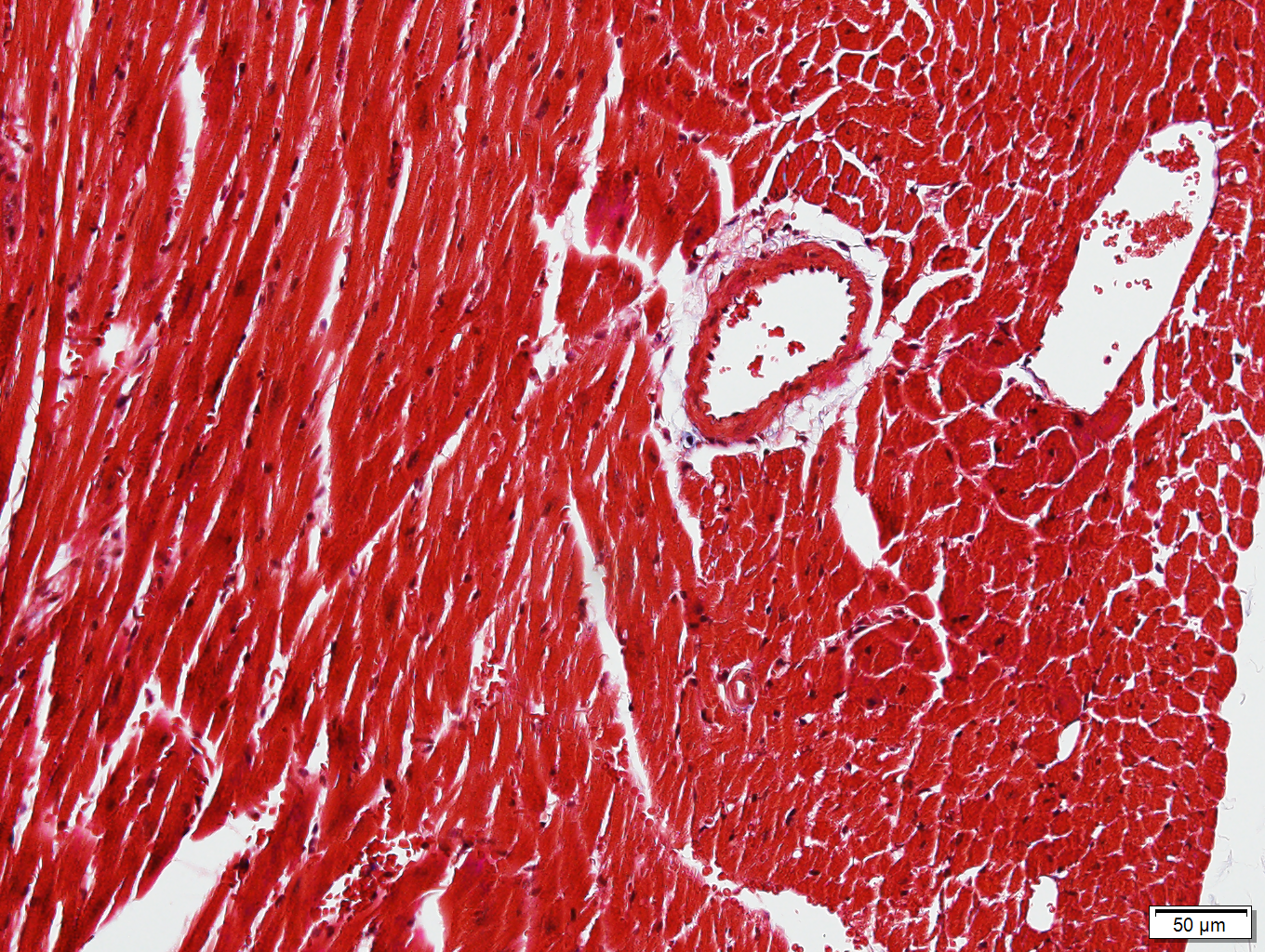

Supplement: Supplementary file 12 — Source data Fig. 9 [file 44321_2025_334_MOESM12_ESM.zip › Figure 9/9G/Perivascular/Sham.tif]

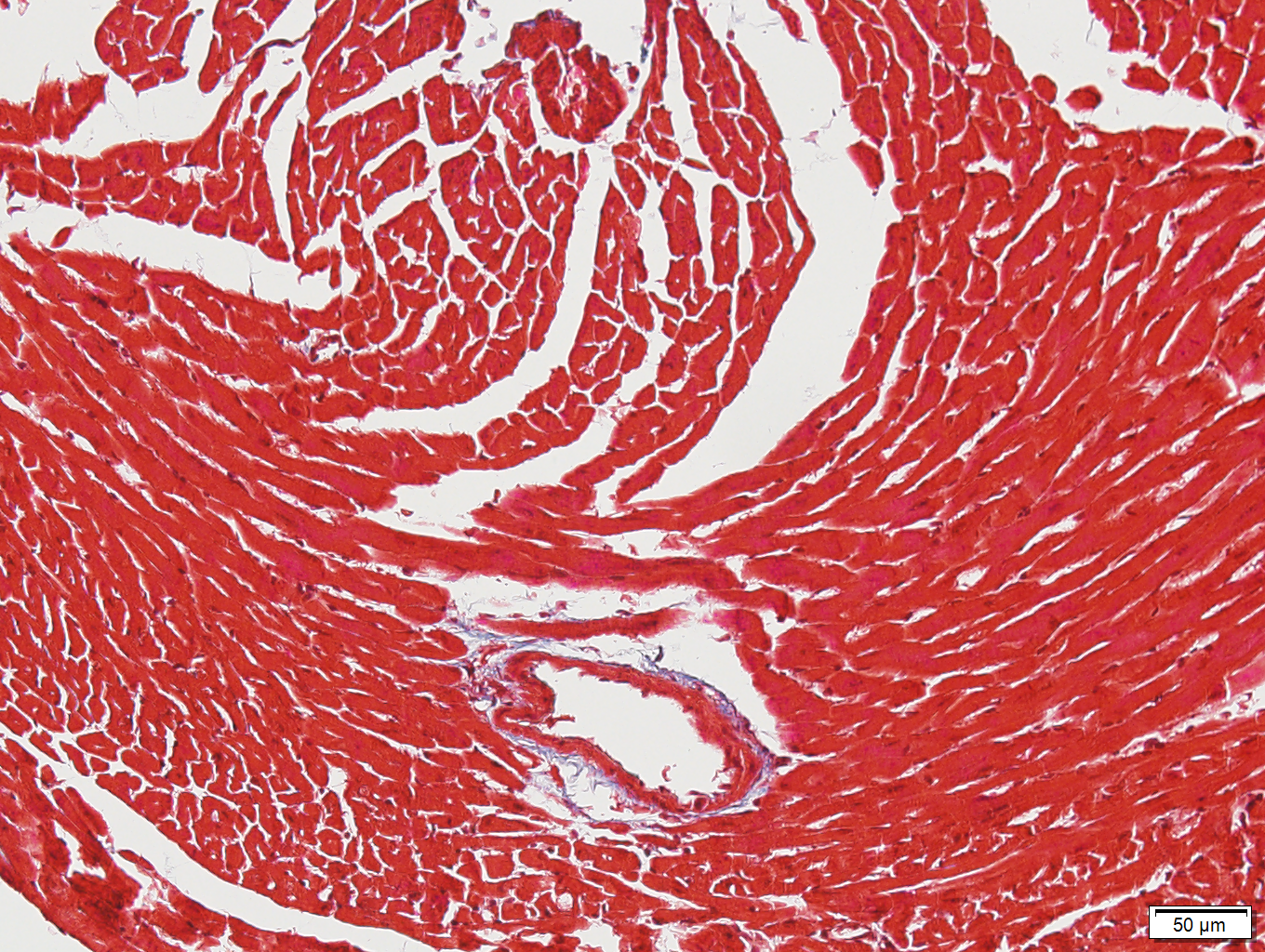

Supplement: Supplementary file 12 — Source data Fig. 9 [file 44321_2025_334_MOESM12_ESM.zip › Figure 9/9G/Perivascular/TAC+NTP.tif]

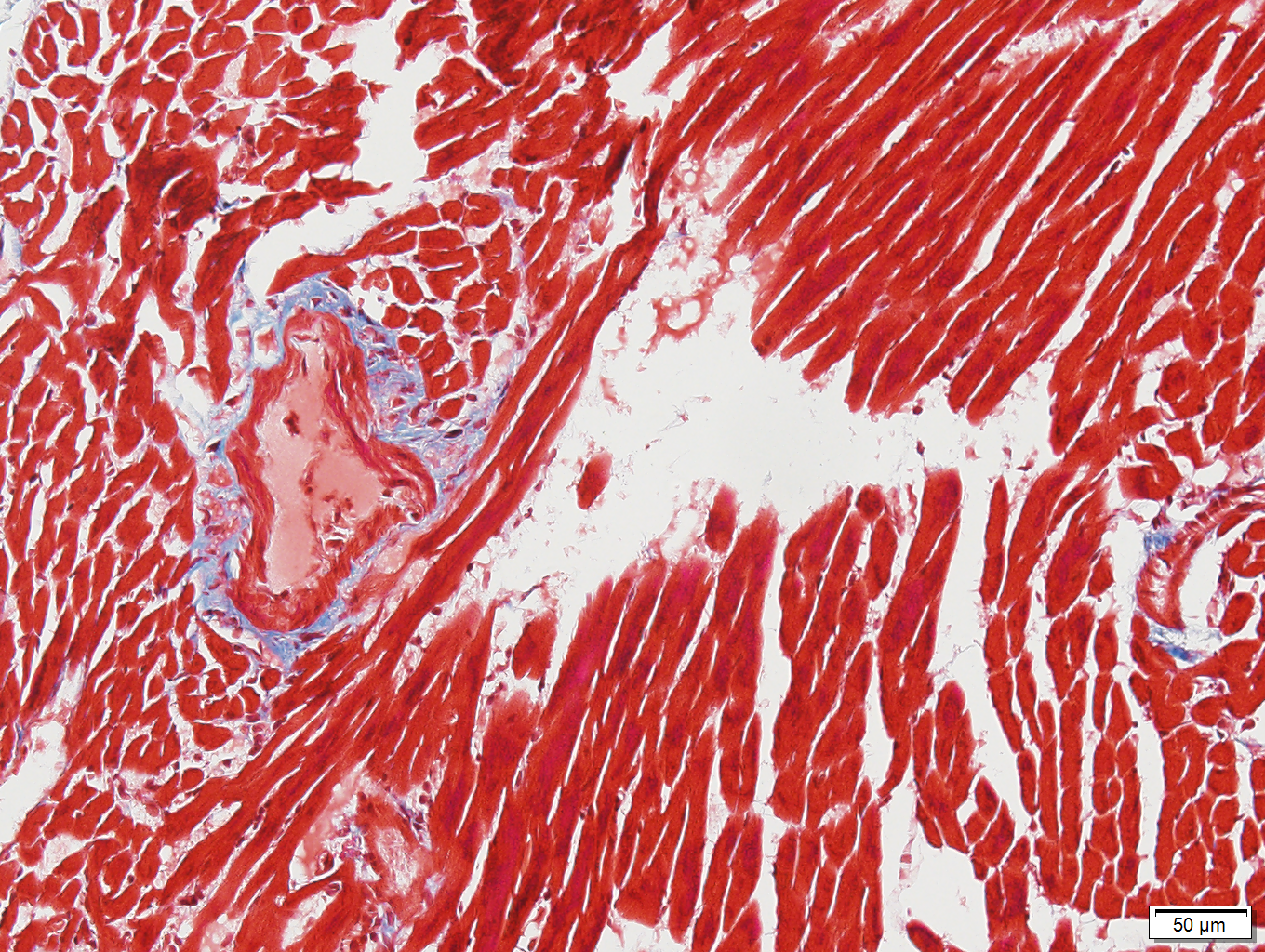

Supplement: Supplementary file 12 — Source data Fig. 9 [file 44321_2025_334_MOESM12_ESM.zip › Figure 9/9G/Perivascular/TAC+VAL.tif]

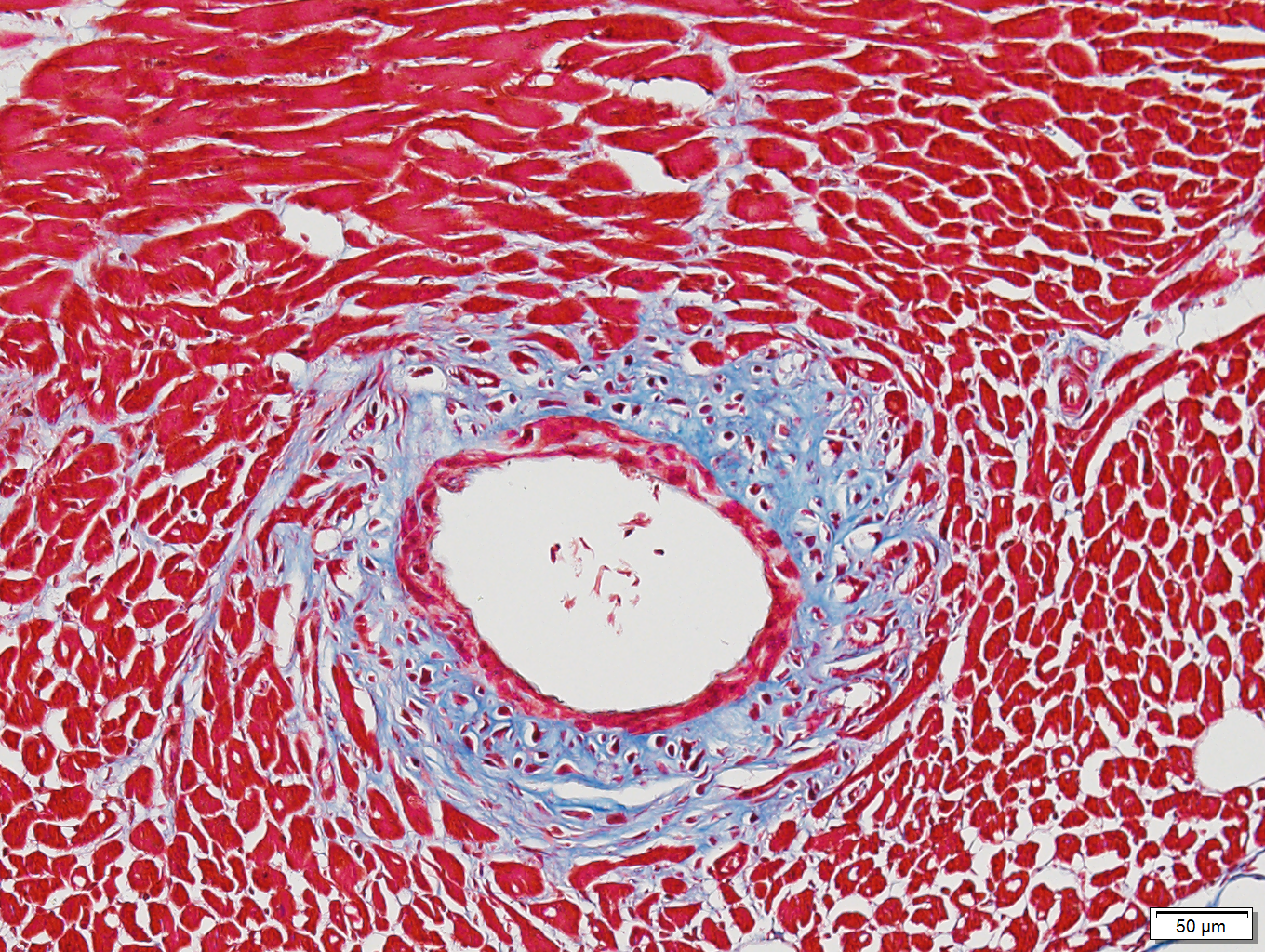

Supplement: Supplementary file 12 — Source data Fig. 9 [file 44321_2025_334_MOESM12_ESM.zip › Figure 9/9G/Perivascular/TAC.tif]

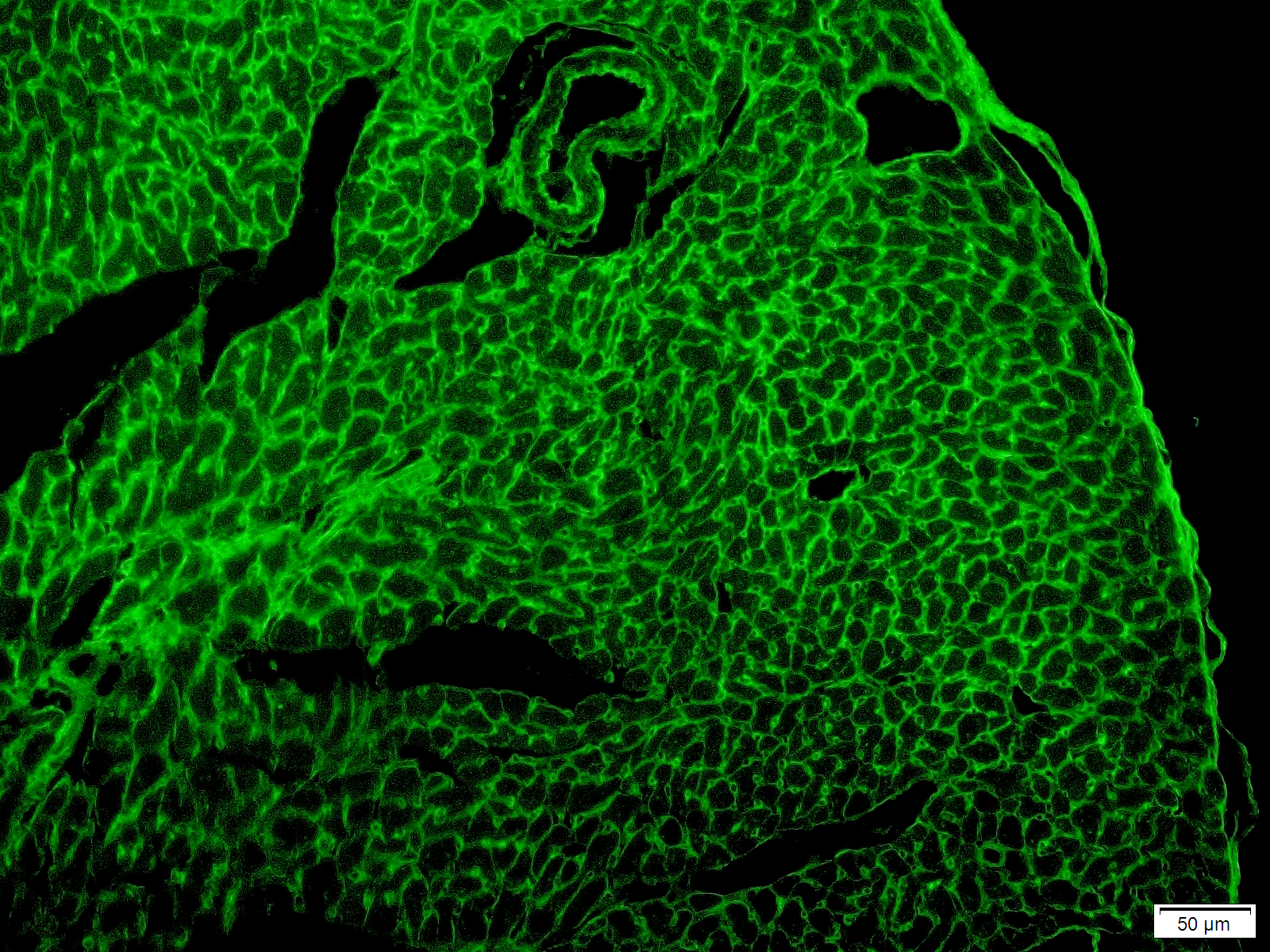

Supplement: Supplementary file 12 — Source data Fig. 9 [file 44321_2025_334_MOESM12_ESM.zip › Figure 9/9I/Sham.tif]

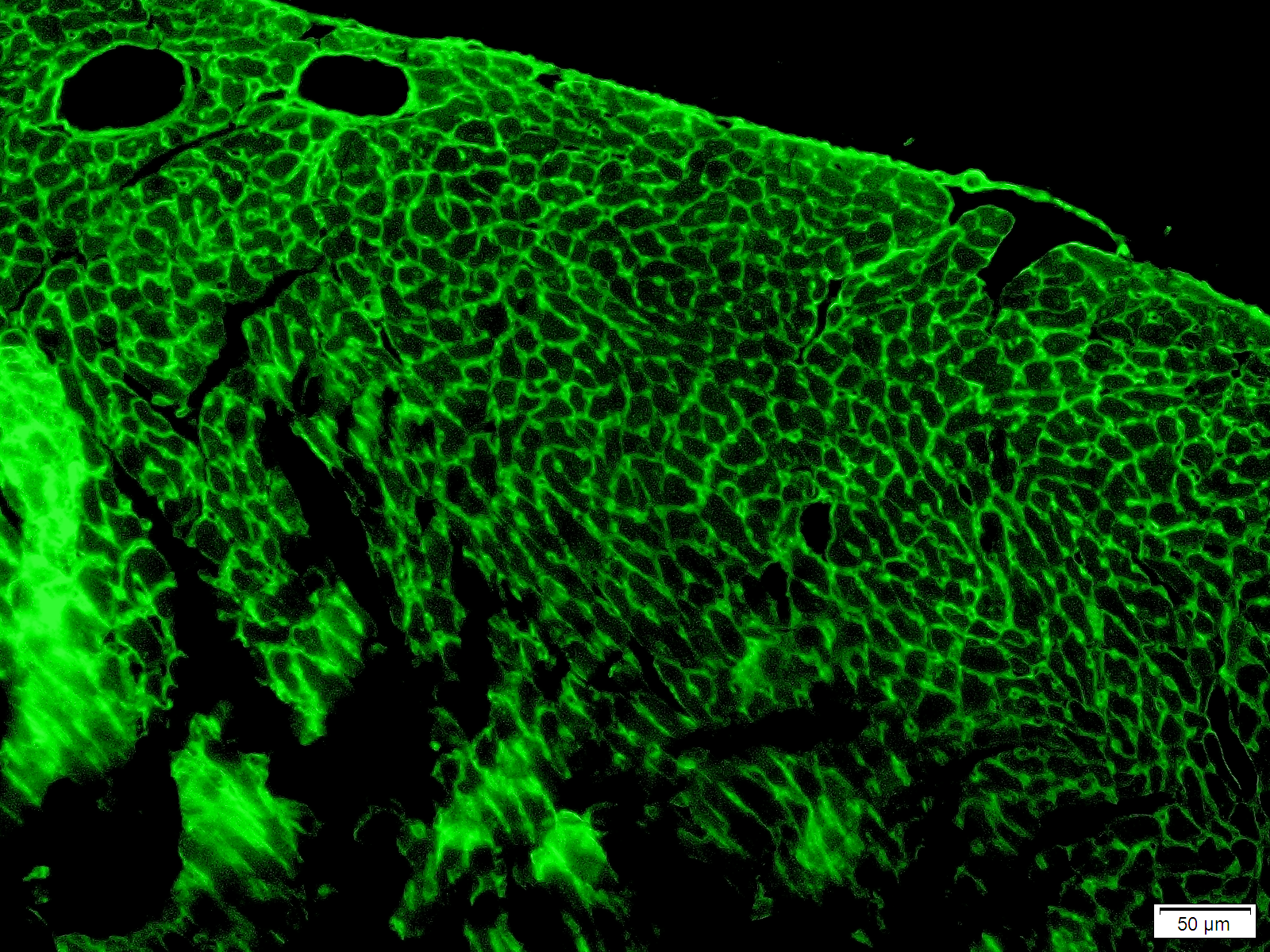

Supplement: Supplementary file 12 — Source data Fig. 9 [file 44321_2025_334_MOESM12_ESM.zip › Figure 9/9I/TAC+NTP.tif]

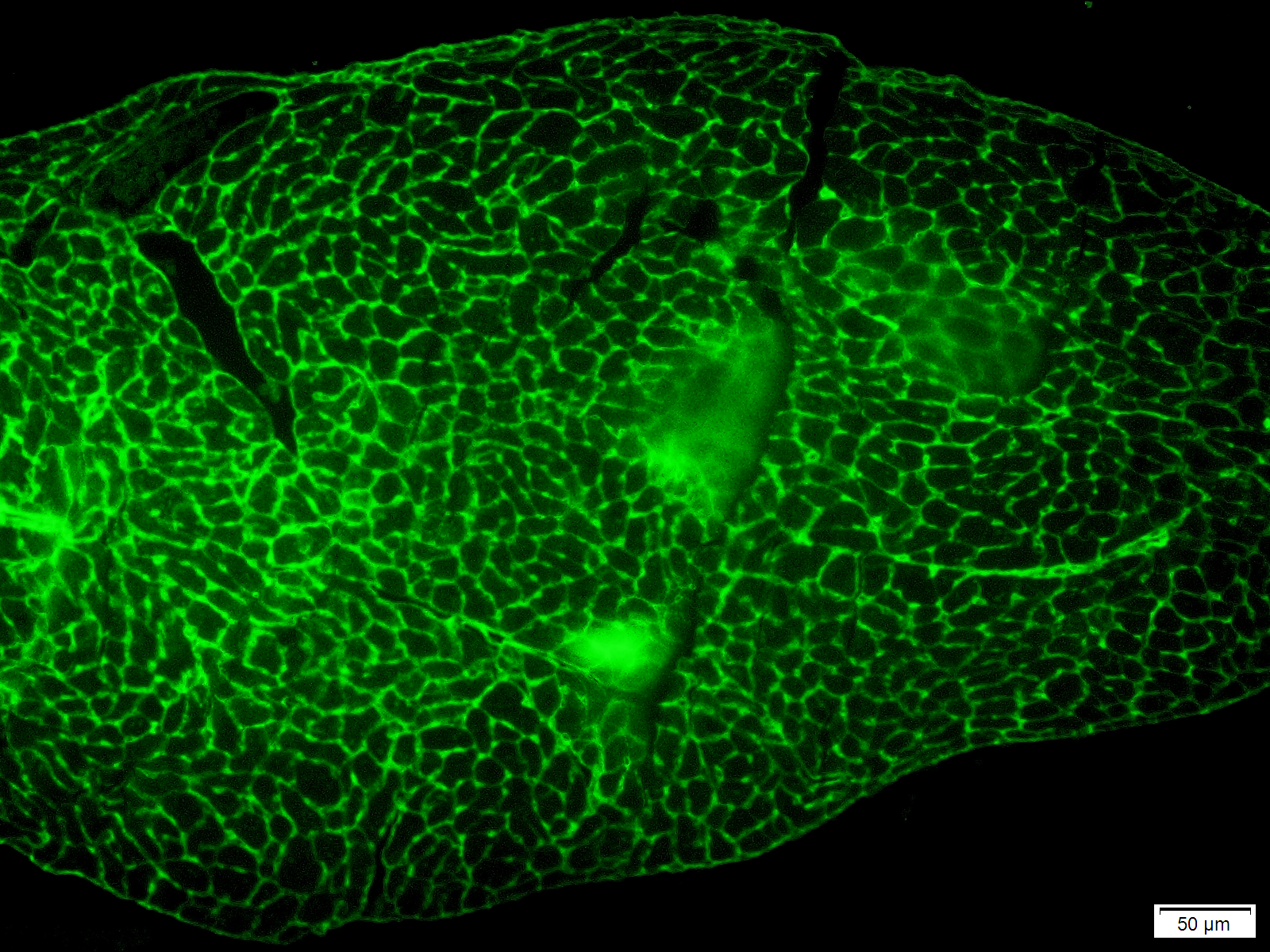

Supplement: Supplementary file 12 — Source data Fig. 9 [file 44321_2025_334_MOESM12_ESM.zip › Figure 9/9I/TAC+VAL.tif]

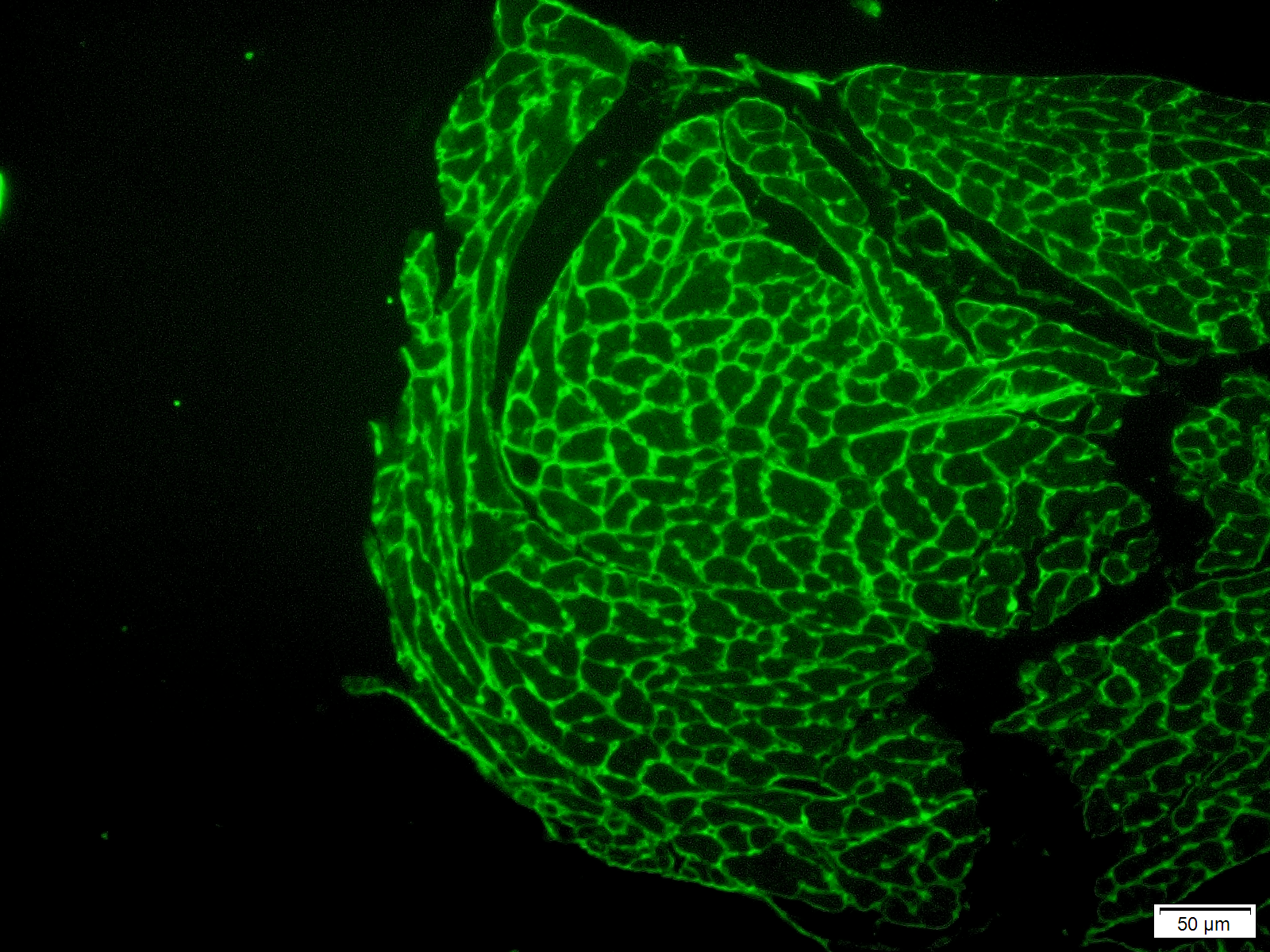

Supplement: Supplementary file 12 — Source data Fig. 9 [file 44321_2025_334_MOESM12_ESM.zip › Figure 9/9I/TAC.tif]

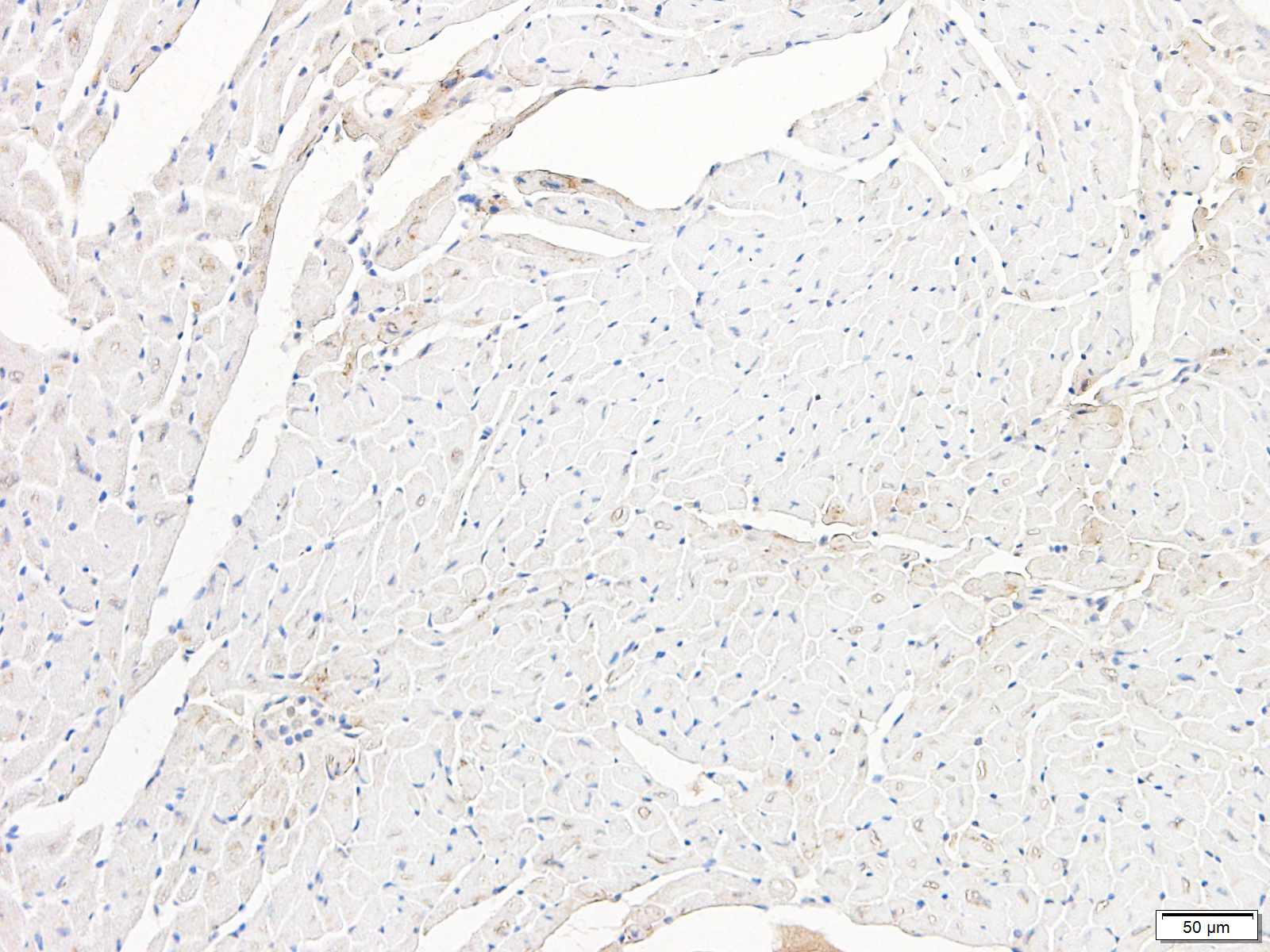

Supplement: Supplementary file 12 — Source data Fig. 9 [file 44321_2025_334_MOESM12_ESM.zip › Figure 9/9K/ANP/Sham.tif]

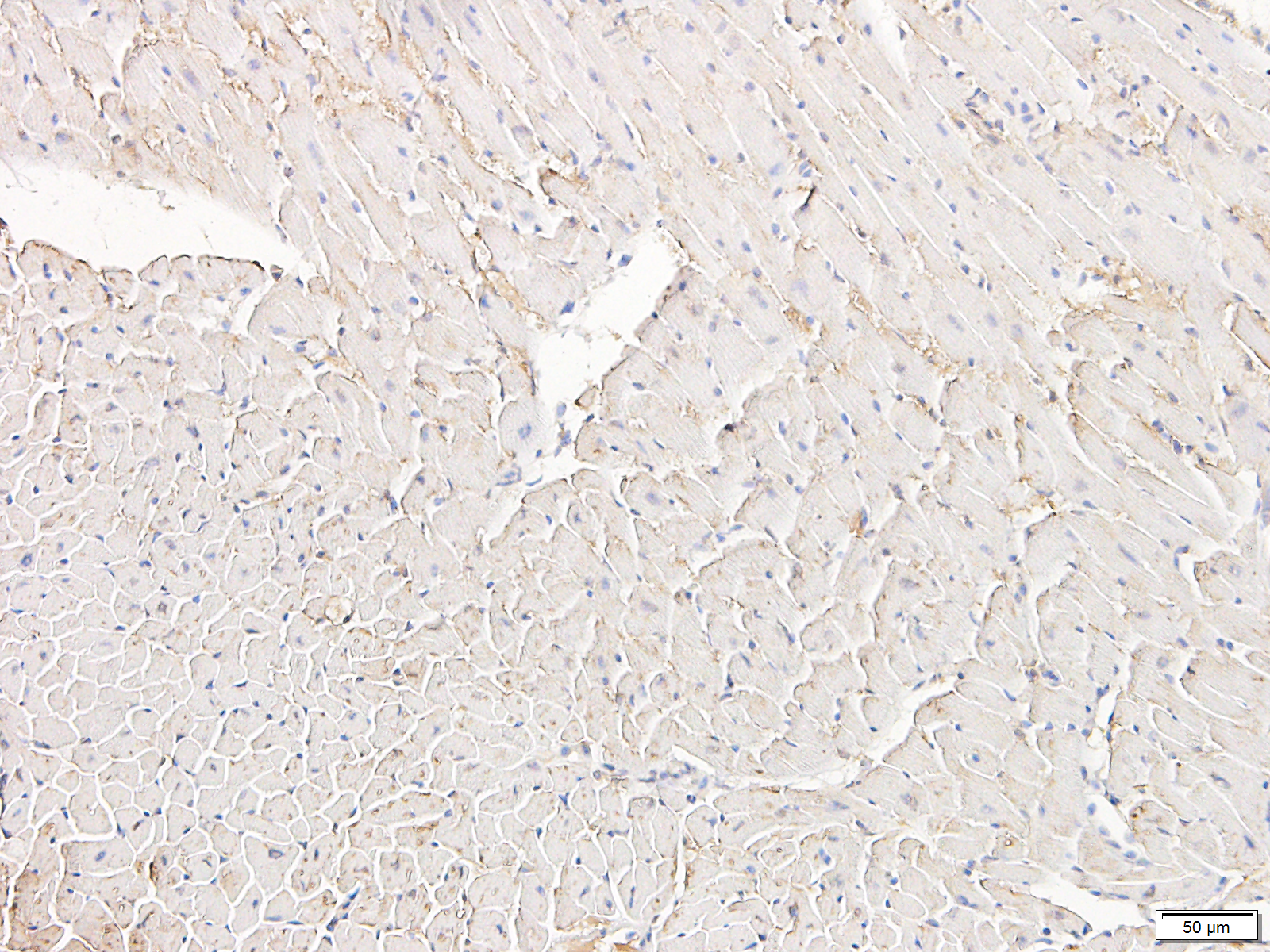

Supplement: Supplementary file 12 — Source data Fig. 9 [file 44321_2025_334_MOESM12_ESM.zip › Figure 9/9K/ANP/TAC+NTP.tif]

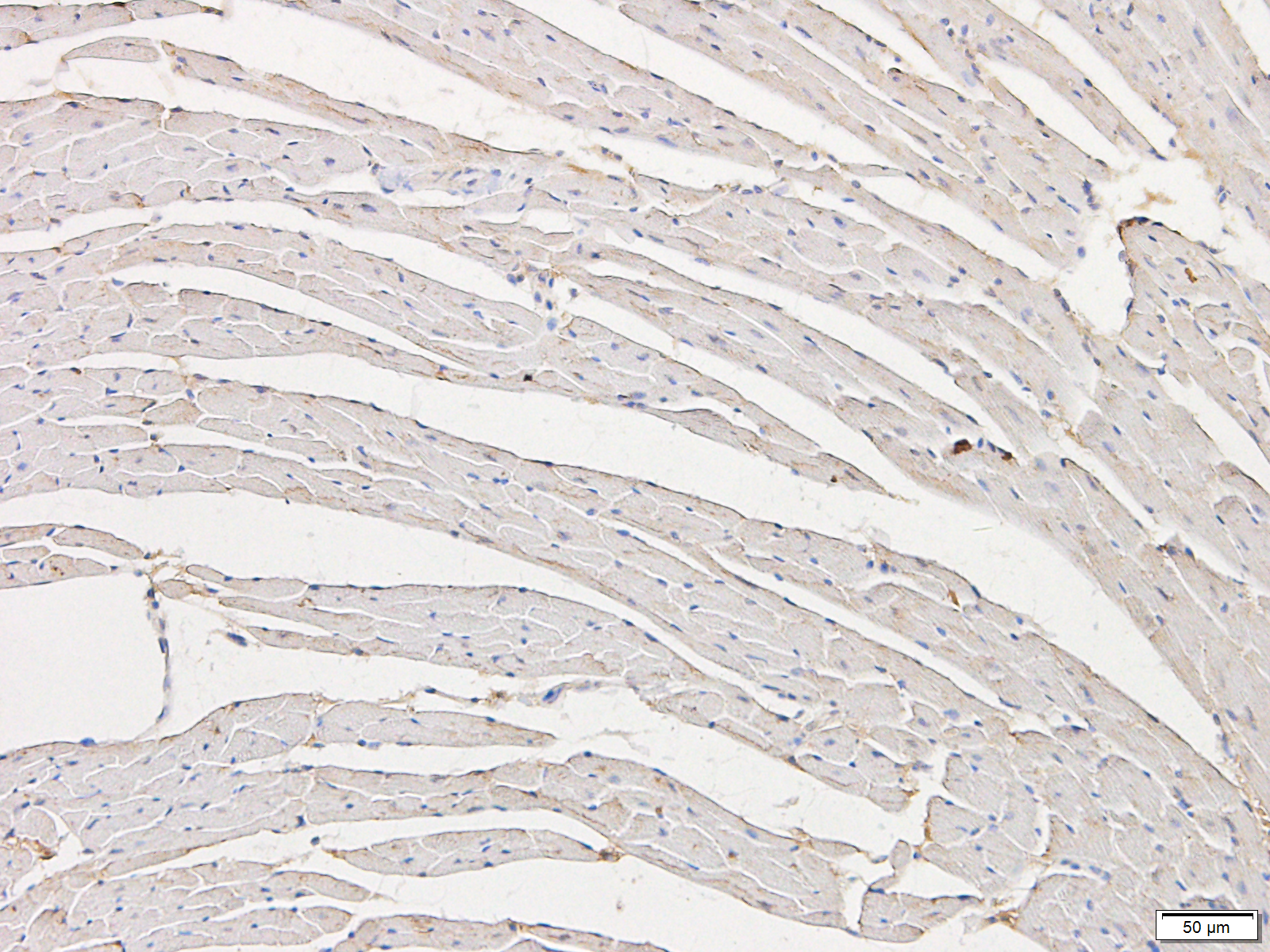

Supplement: Supplementary file 12 — Source data Fig. 9 [file 44321_2025_334_MOESM12_ESM.zip › Figure 9/9K/ANP/TAC+VAL.tif]

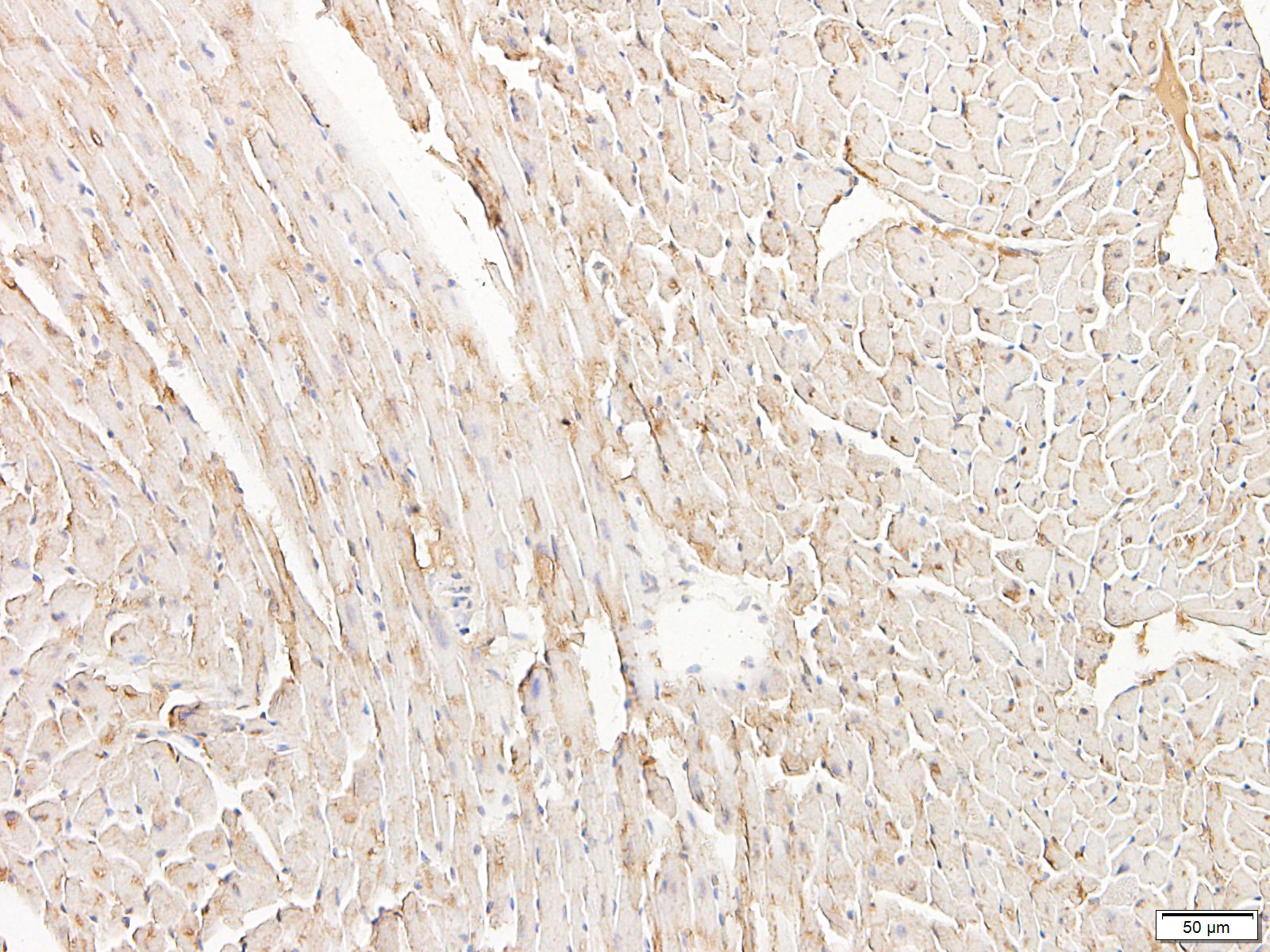

Supplement: Supplementary file 12 — Source data Fig. 9 [file 44321_2025_334_MOESM12_ESM.zip › Figure 9/9K/ANP/TAC.tif]

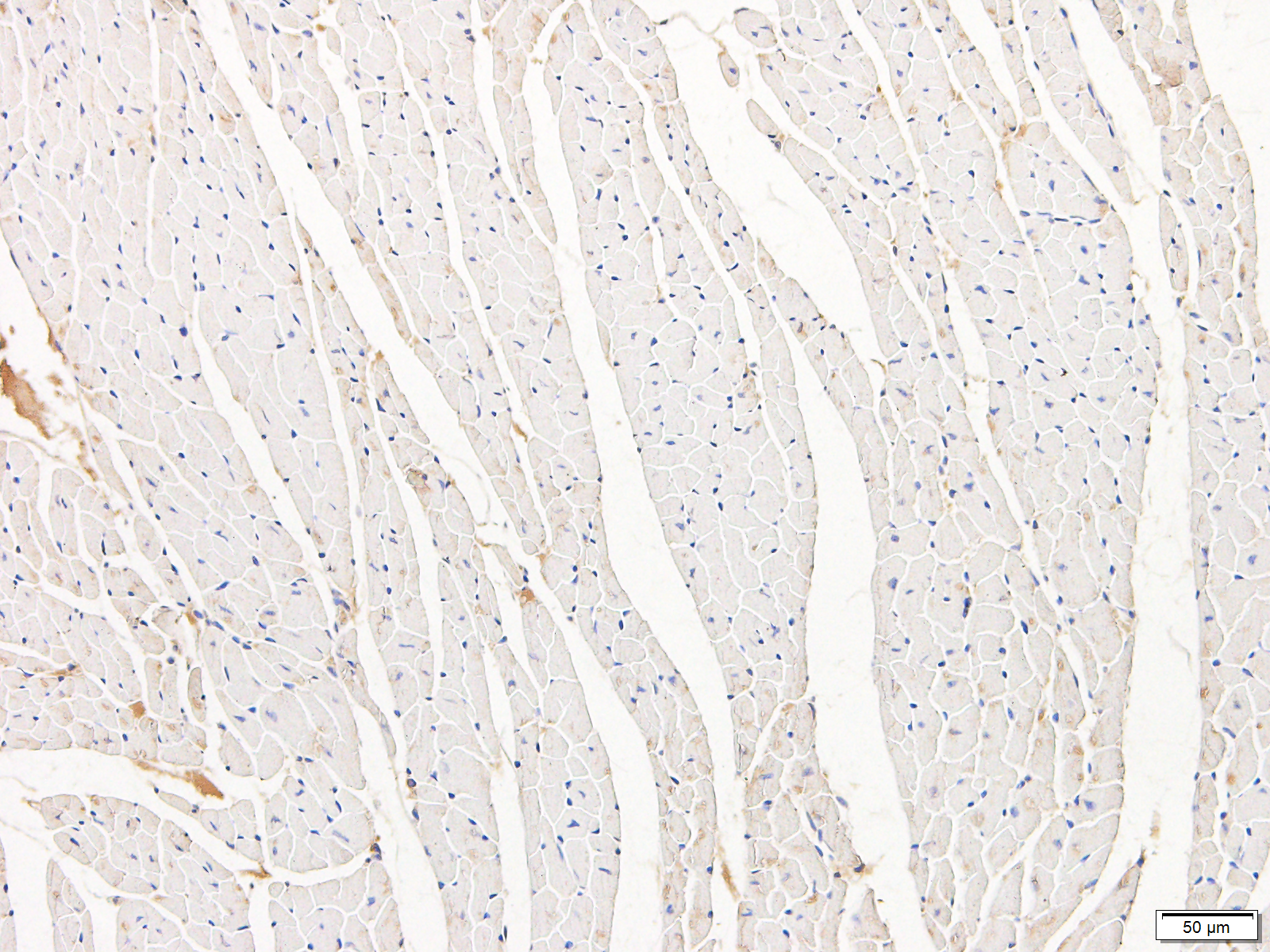

Supplement: Supplementary file 12 — Source data Fig. 9 [file 44321_2025_334_MOESM12_ESM.zip › Figure 9/9K/β-MHC/Sham.tif]

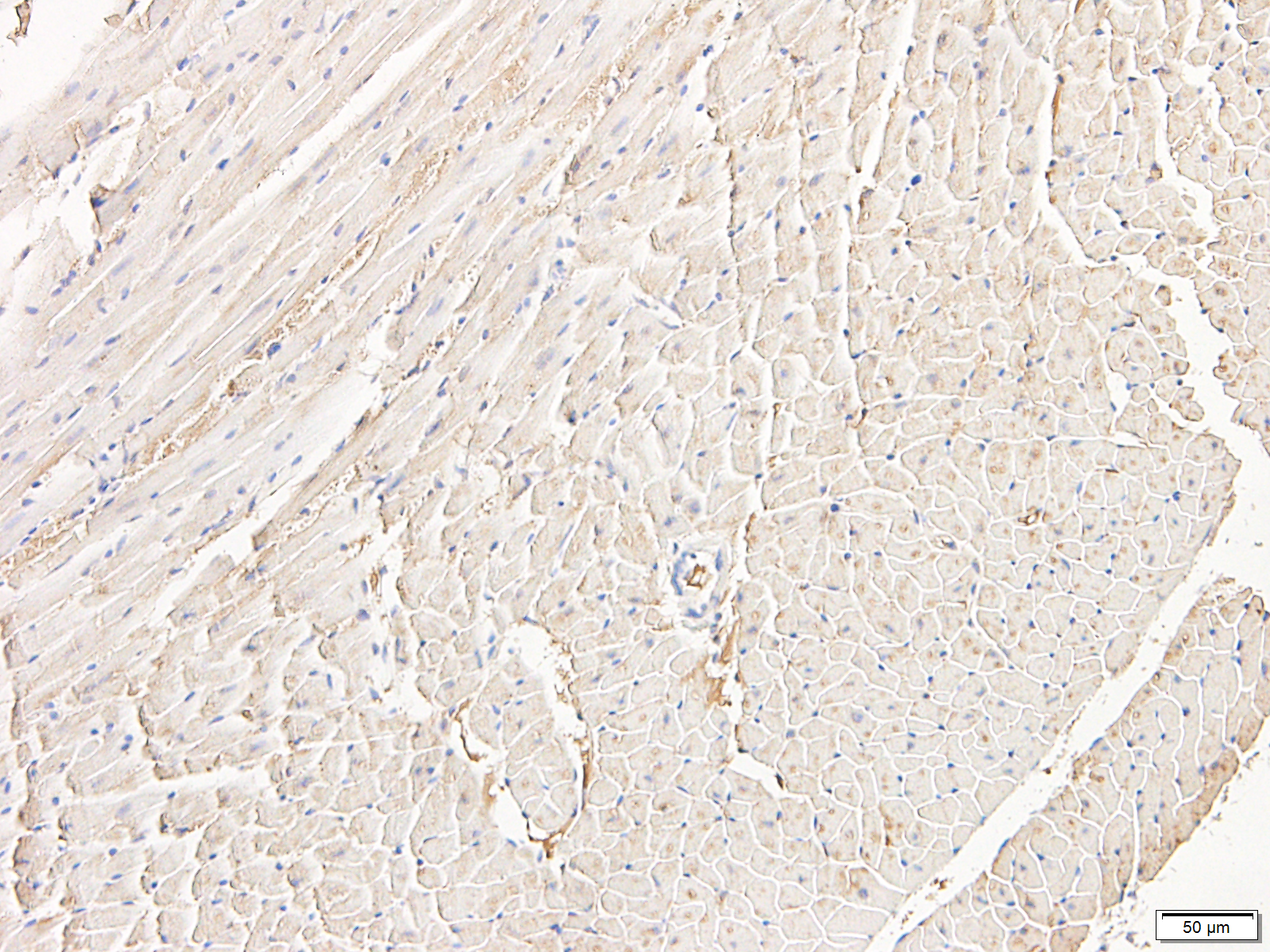

Supplement: Supplementary file 12 — Source data Fig. 9 [file 44321_2025_334_MOESM12_ESM.zip › Figure 9/9K/β-MHC/TAC+NTP.tif]

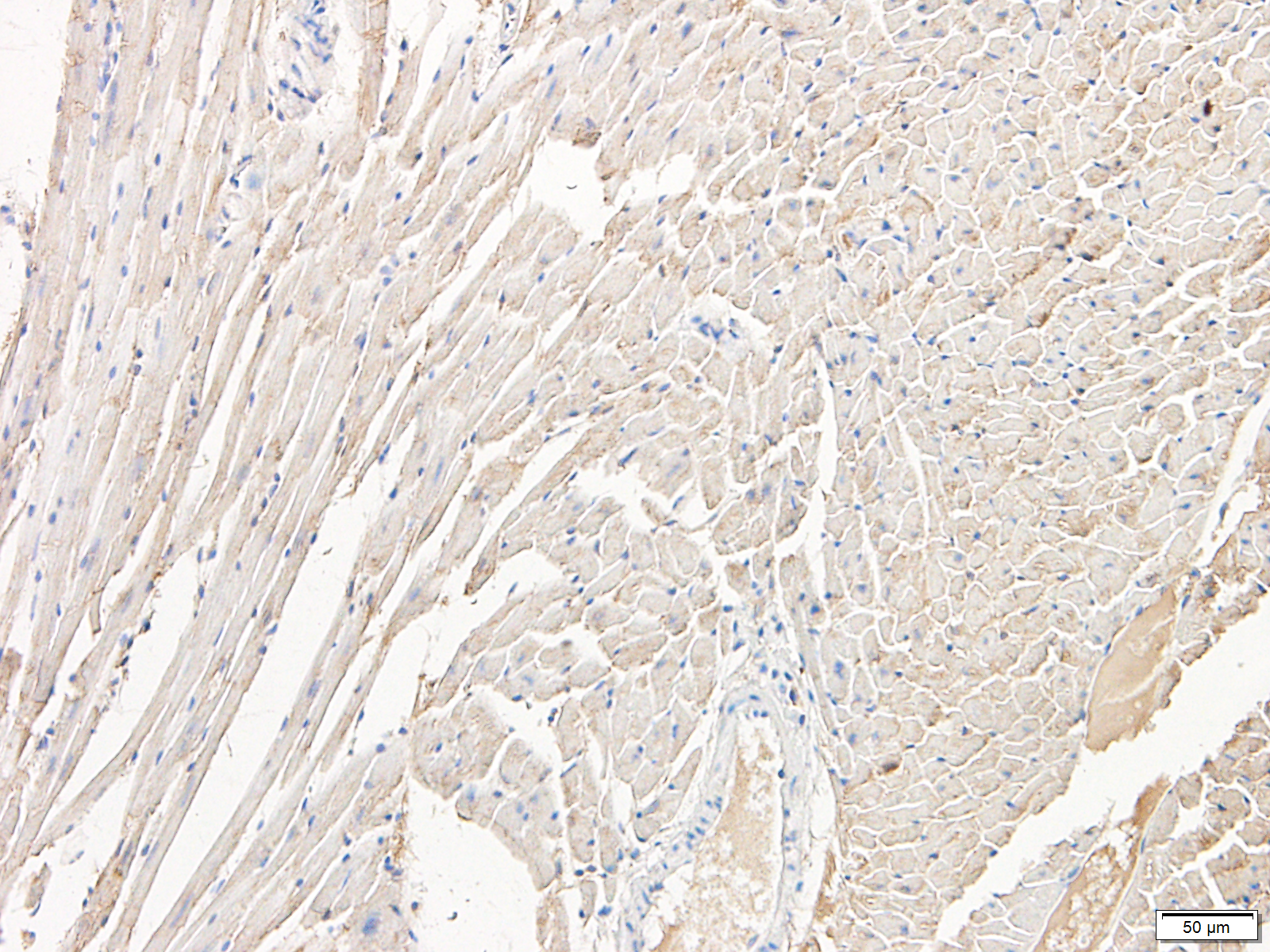

Supplement: Supplementary file 12 — Source data Fig. 9 [file 44321_2025_334_MOESM12_ESM.zip › Figure 9/9K/β-MHC/TAC+VAL.tif]

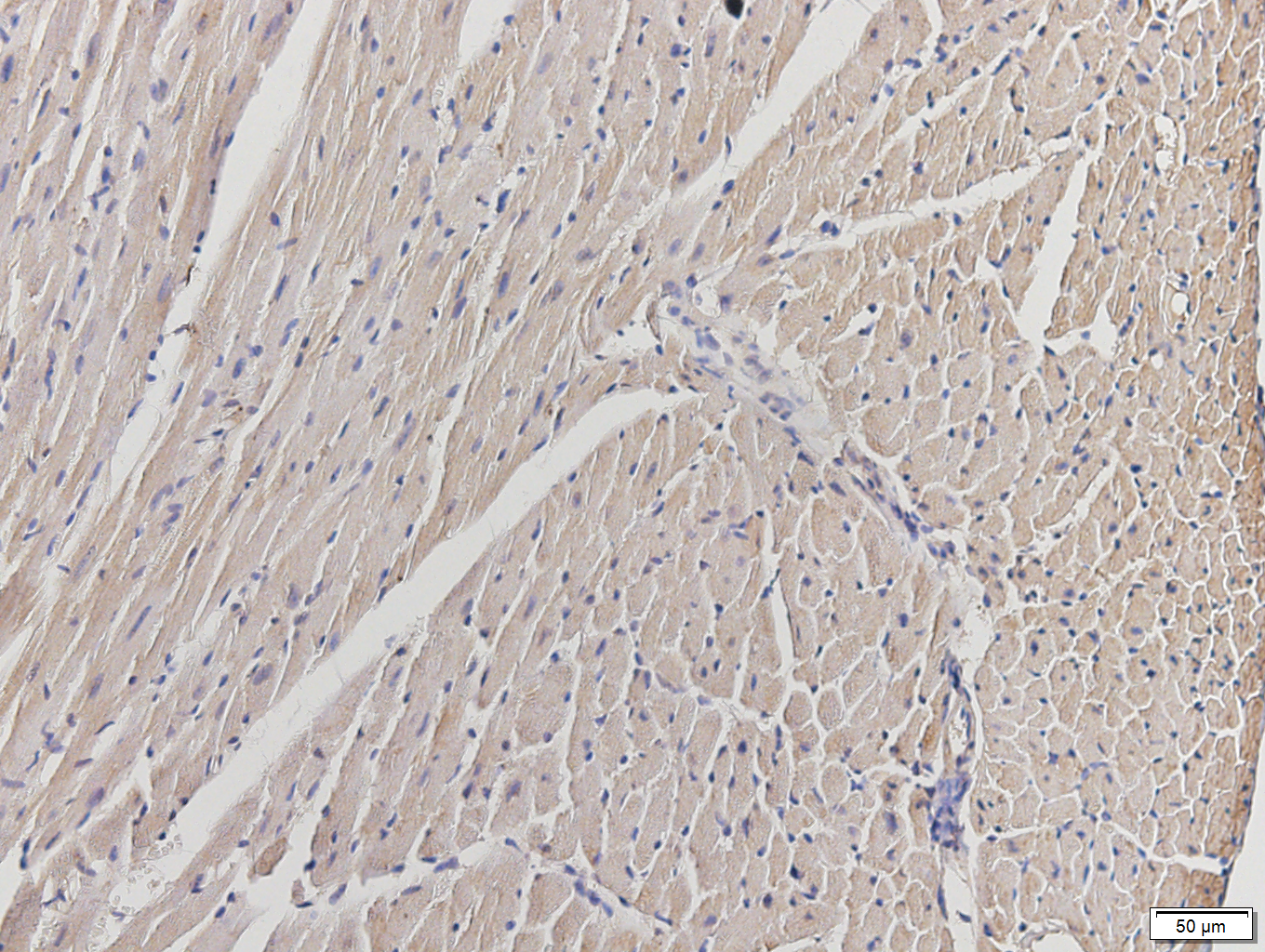

Supplement: Supplementary file 12 — Source data Fig. 9 [file 44321_2025_334_MOESM12_ESM.zip › Figure 9/9K/β-MHC/TAC.tif]

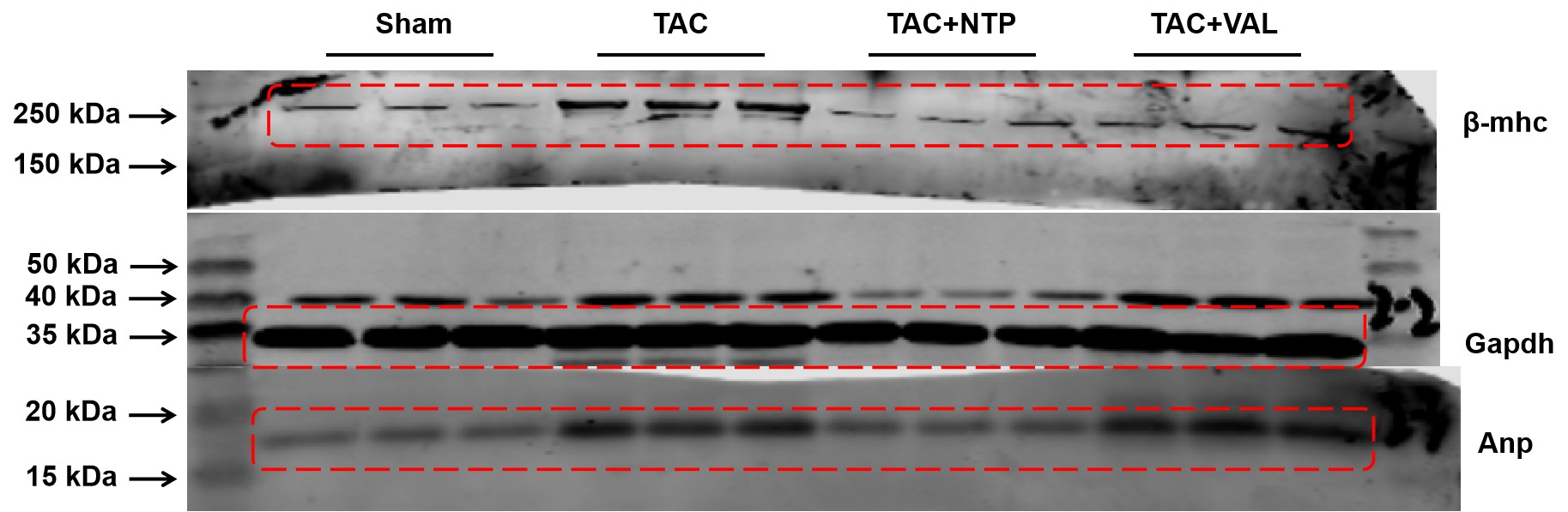

Supplement: Supplementary file 12 — Source data Fig. 9 [file 44321_2025_334_MOESM12_ESM.zip › Figure 9/9M/9M.jpg]

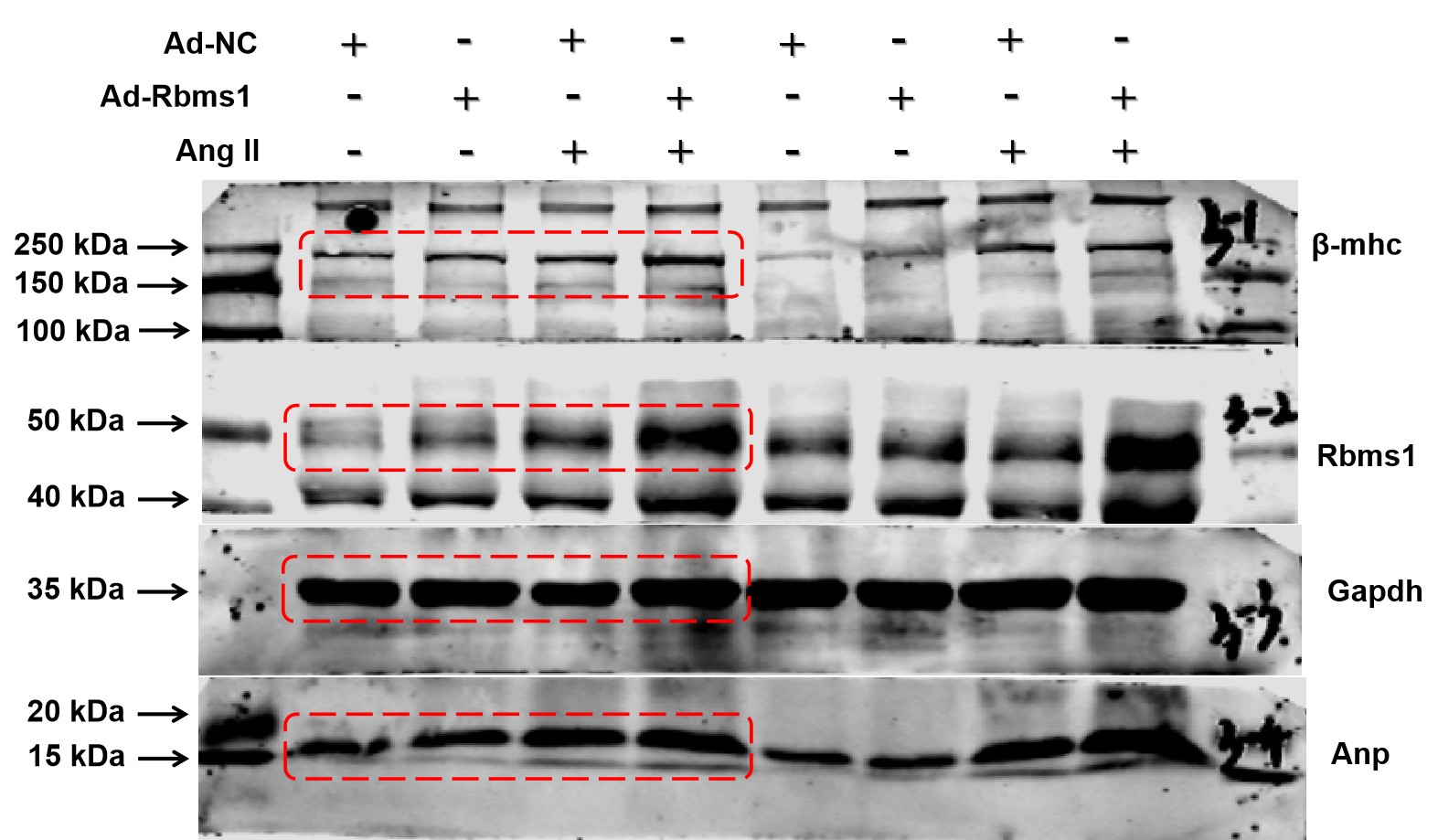

Supplement: Supplementary file 13 — Figure EV1 Source Data [file 44321_2025_334_MOESM13_ESM.zip › EV1A/EV1A.jpg]

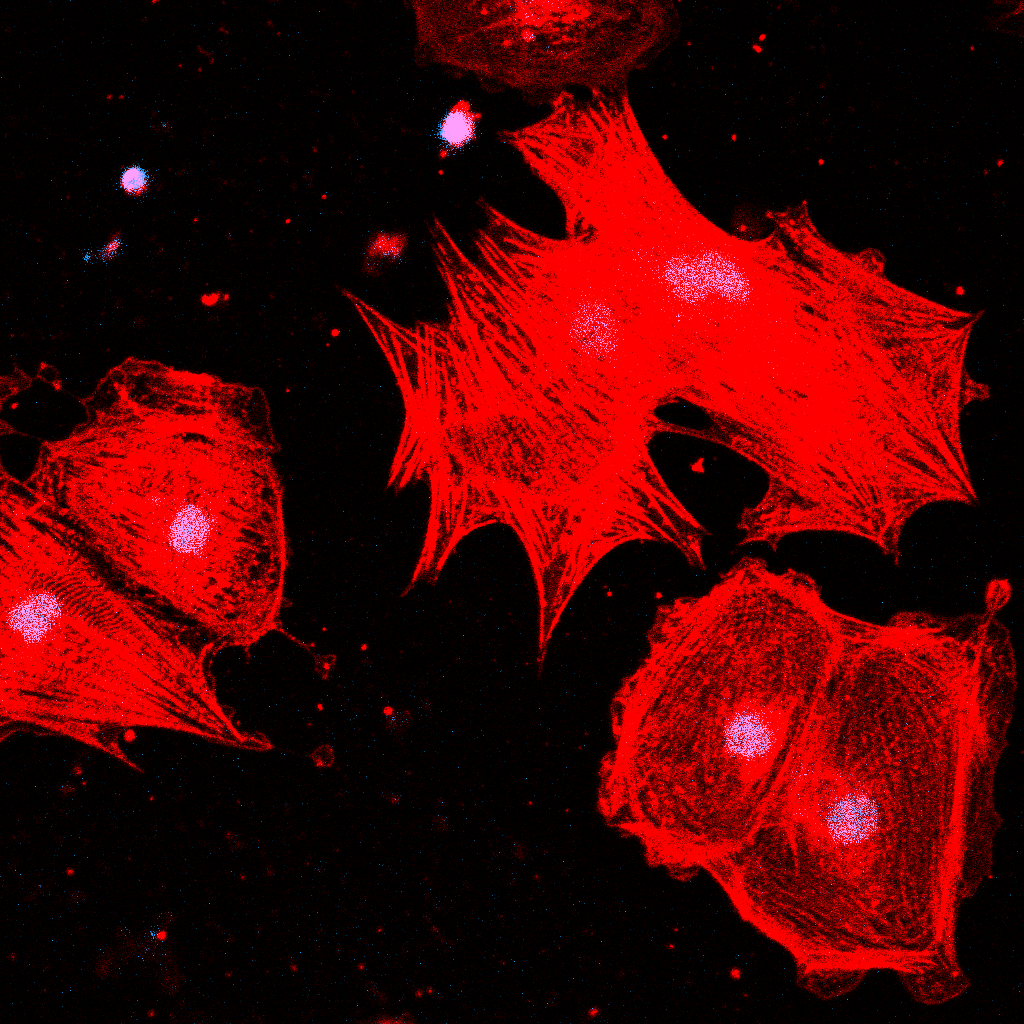

Supplement: Supplementary file 13 — Figure EV1 Source Data [file 44321_2025_334_MOESM13_ESM.zip › EV1D/Ang II+pcDNA3.1.tiff]

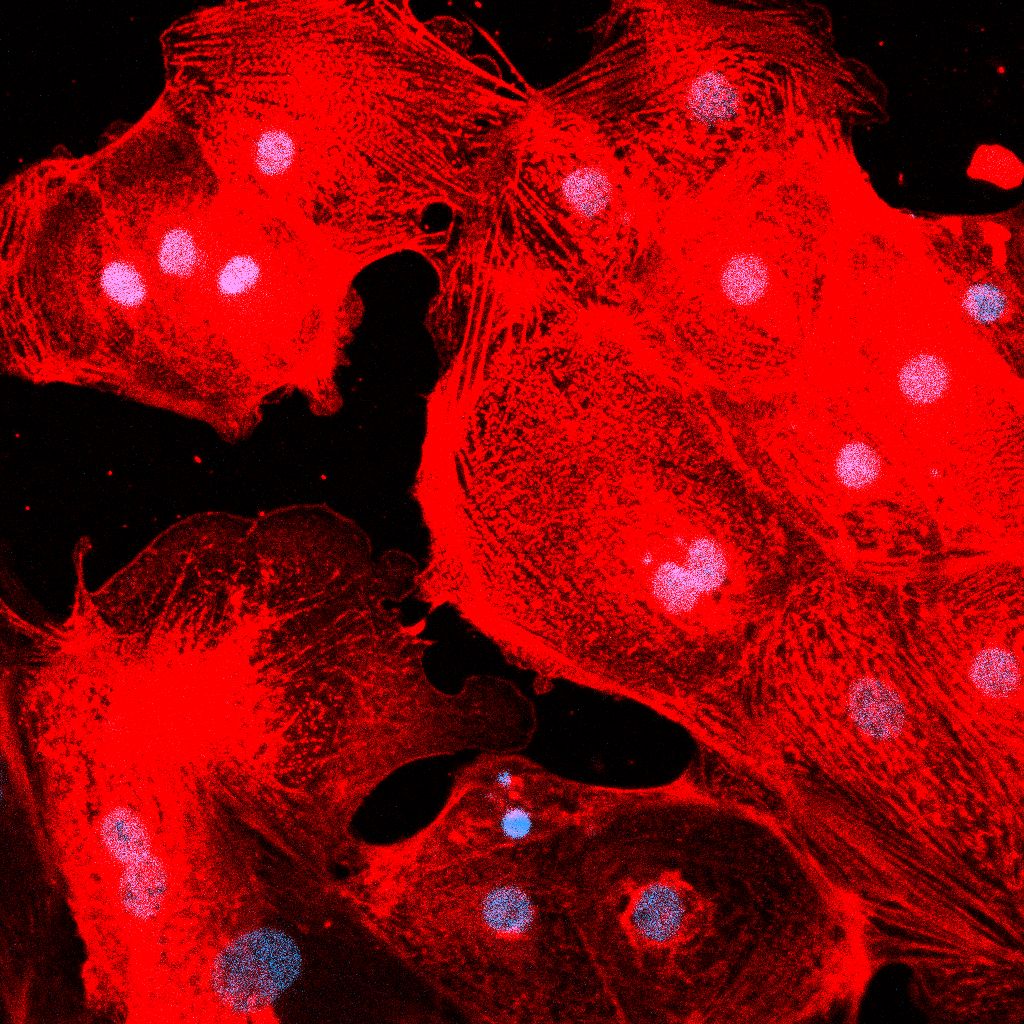

Supplement: Supplementary file 13 — Figure EV1 Source Data [file 44321_2025_334_MOESM13_ESM.zip › EV1D/Ang II+RBMS1.tiff]

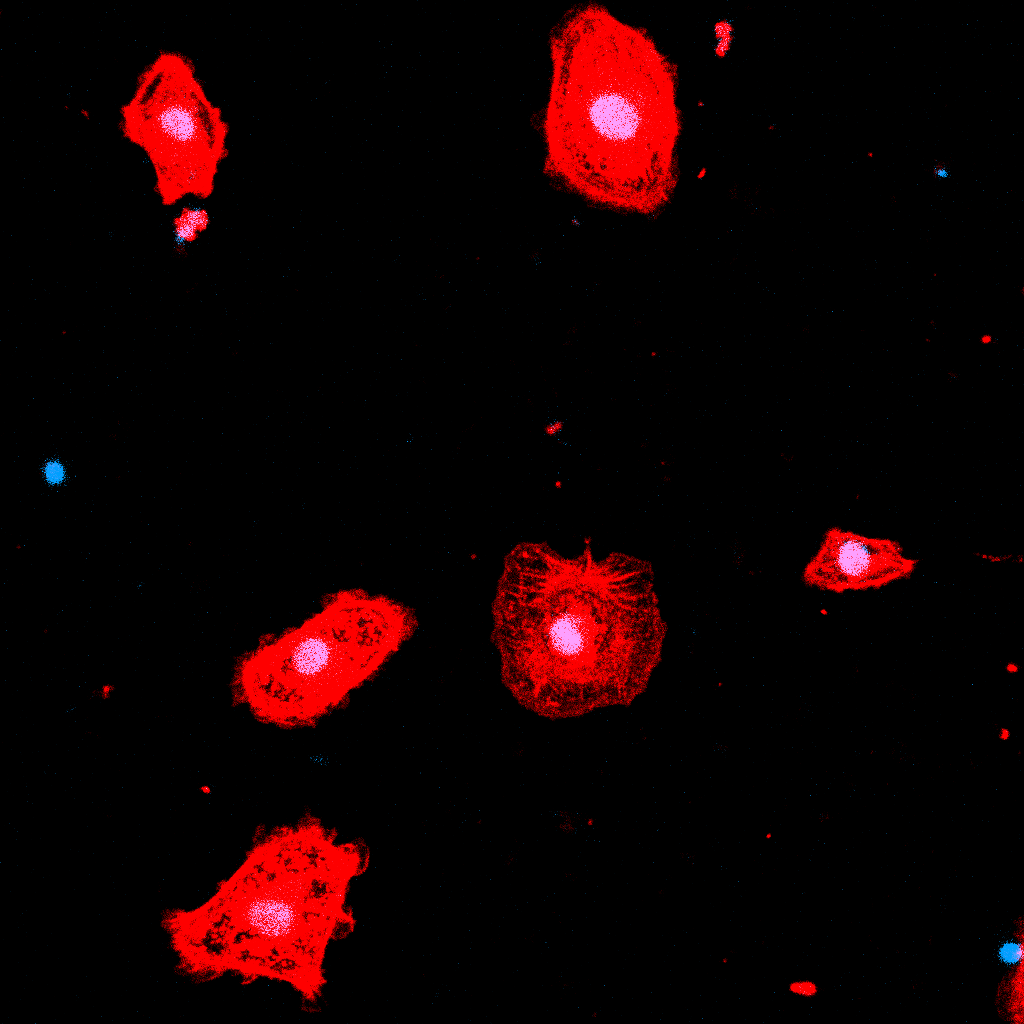

Supplement: Supplementary file 13 — Figure EV1 Source Data [file 44321_2025_334_MOESM13_ESM.zip › EV1D/pcDNA3.1.tiff]

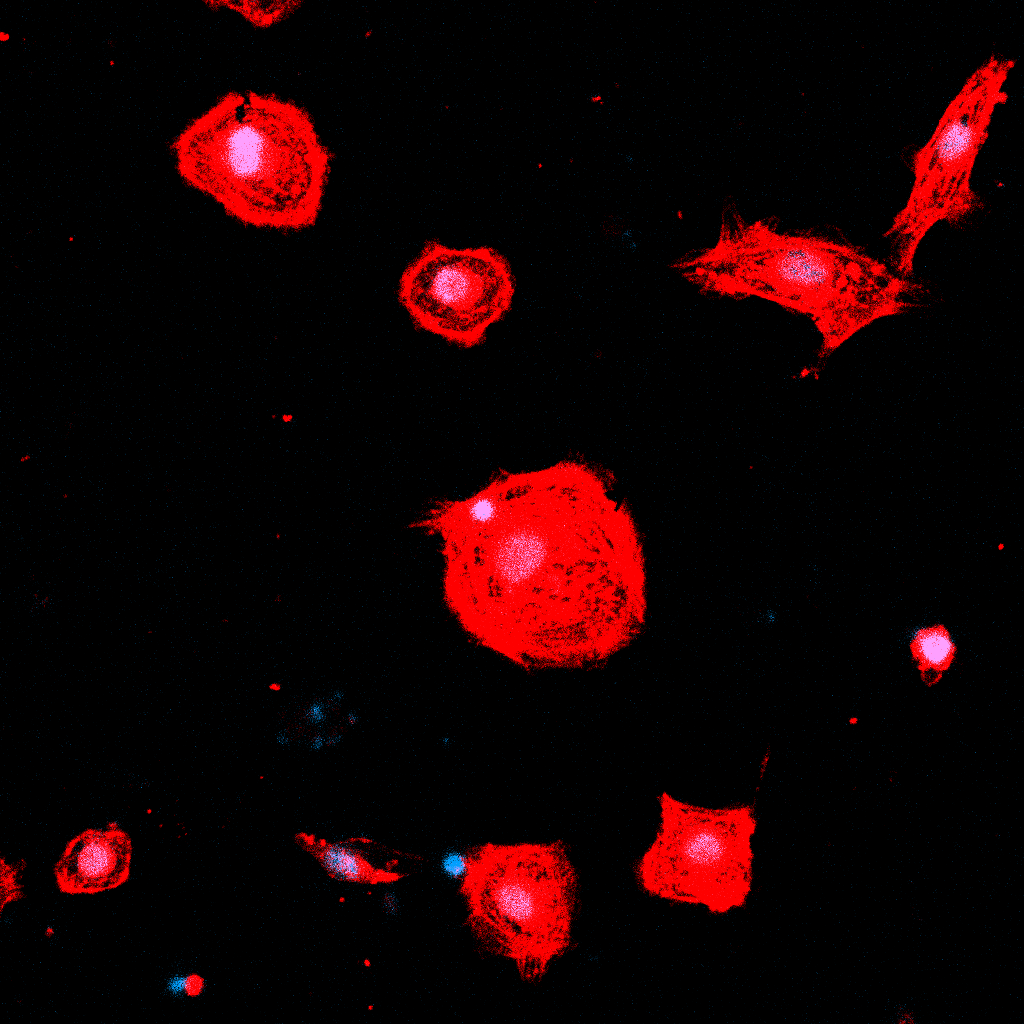

Supplement: Supplementary file 13 — Figure EV1 Source Data [file 44321_2025_334_MOESM13_ESM.zip › EV1D/RBMS1.tiff]

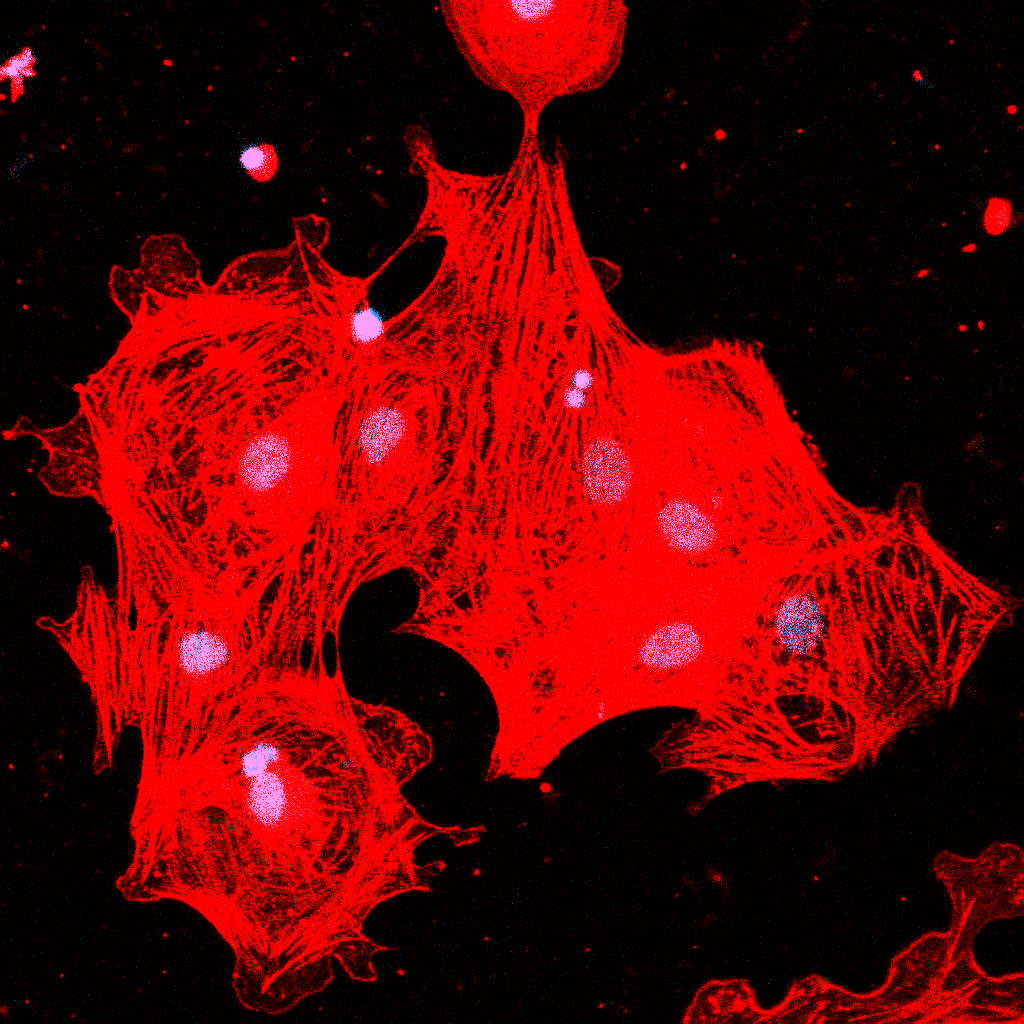

Supplement: Supplementary file 13 — Figure EV1 Source Data [file 44321_2025_334_MOESM13_ESM.zip › EV1F/Ang II+si-NC.tiff]

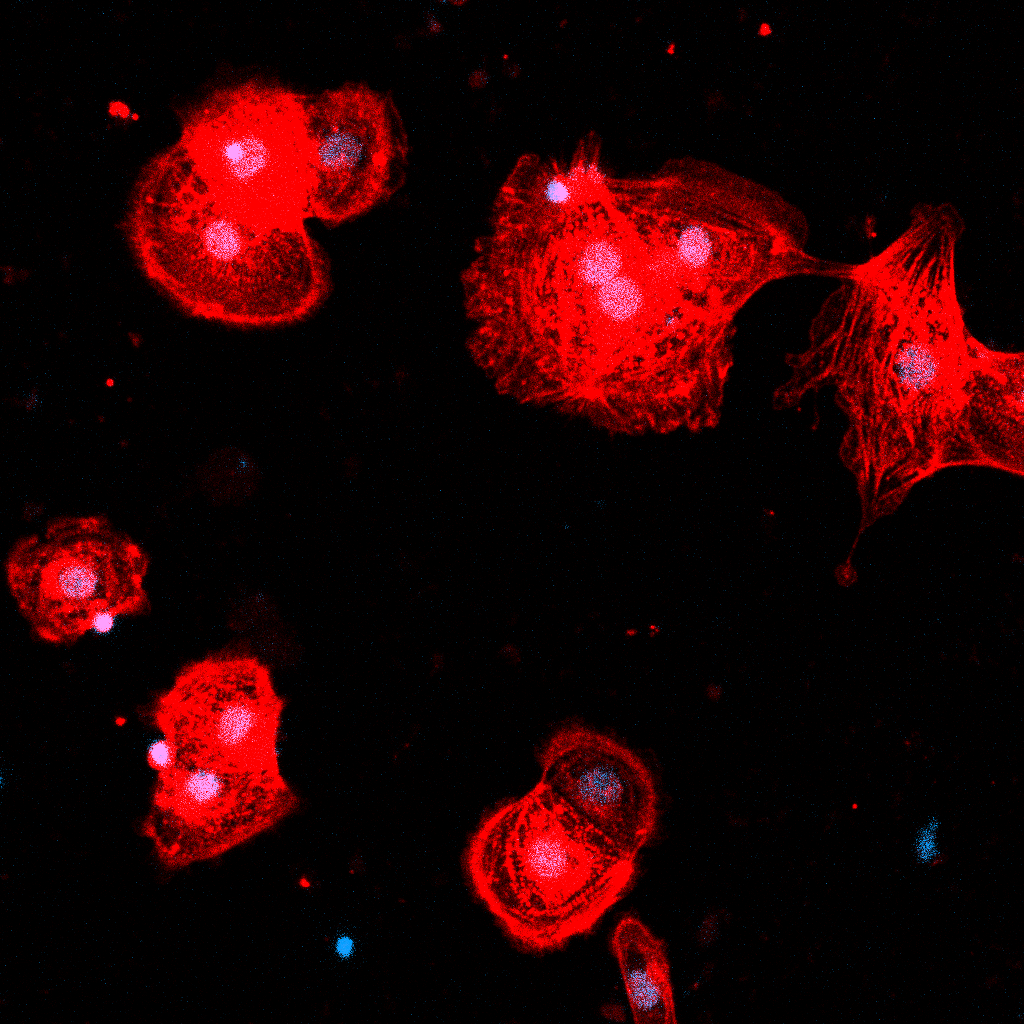

Supplement: Supplementary file 13 — Figure EV1 Source Data [file 44321_2025_334_MOESM13_ESM.zip › EV1F/Ang II+si-RBMS1.tiff]

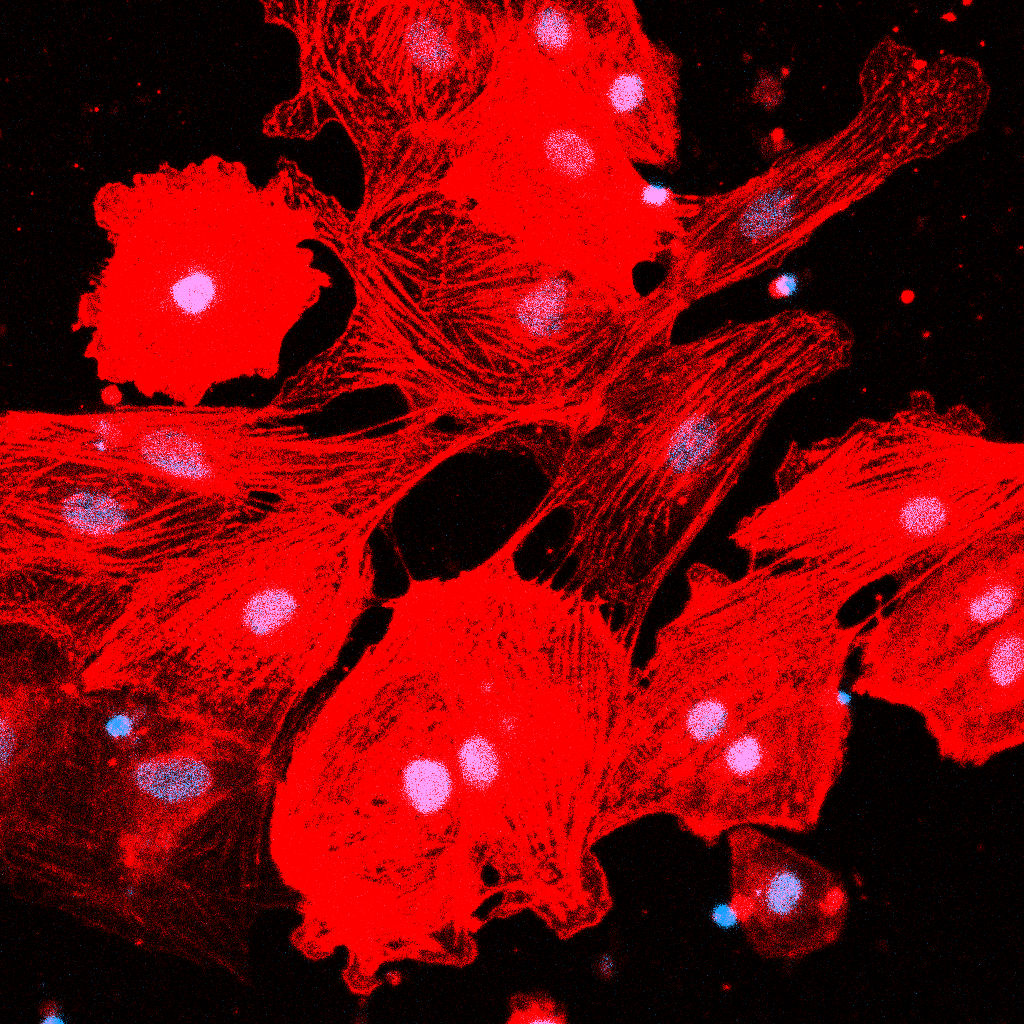

Supplement: Supplementary file 13 — Figure EV1 Source Data [file 44321_2025_334_MOESM13_ESM.zip › EV1F/Ang II.tiff]

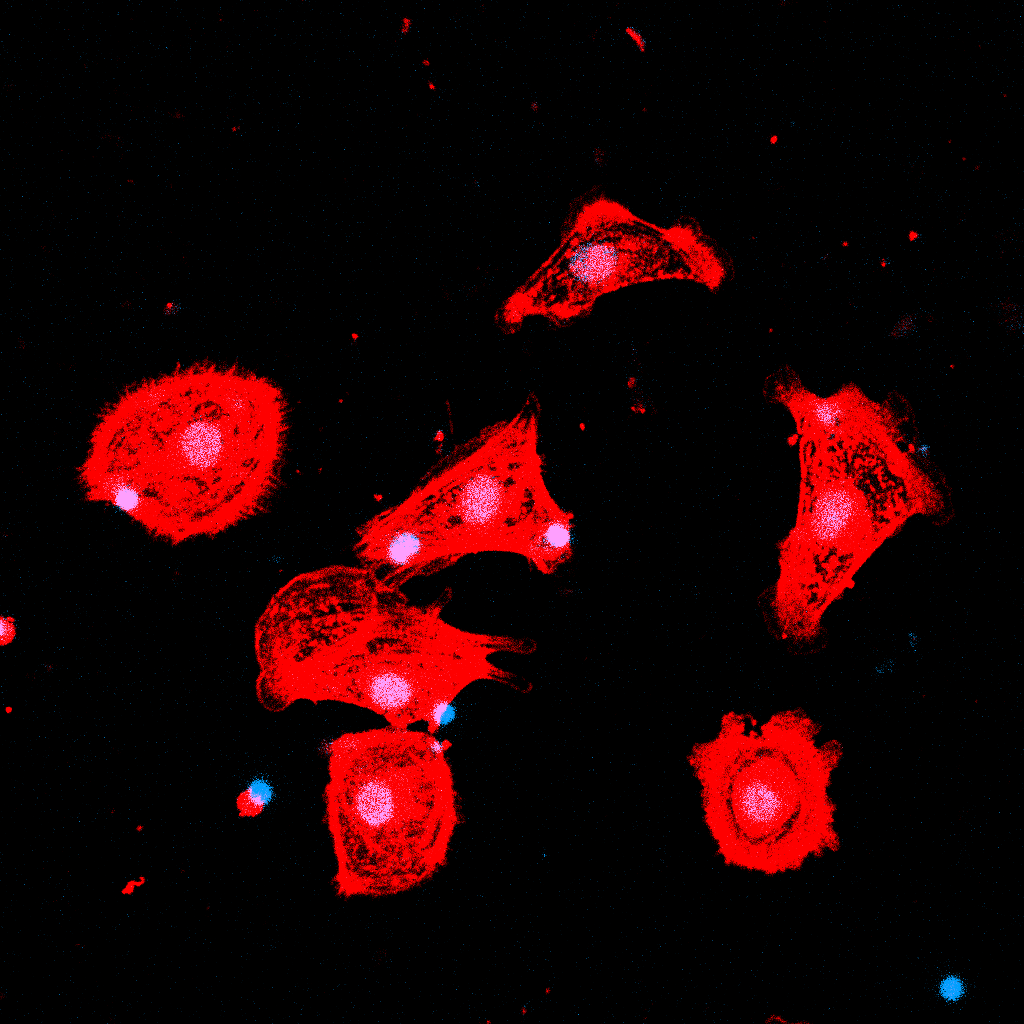

Supplement: Supplementary file 13 — Figure EV1 Source Data [file 44321_2025_334_MOESM13_ESM.zip › EV1F/Control.tiff]

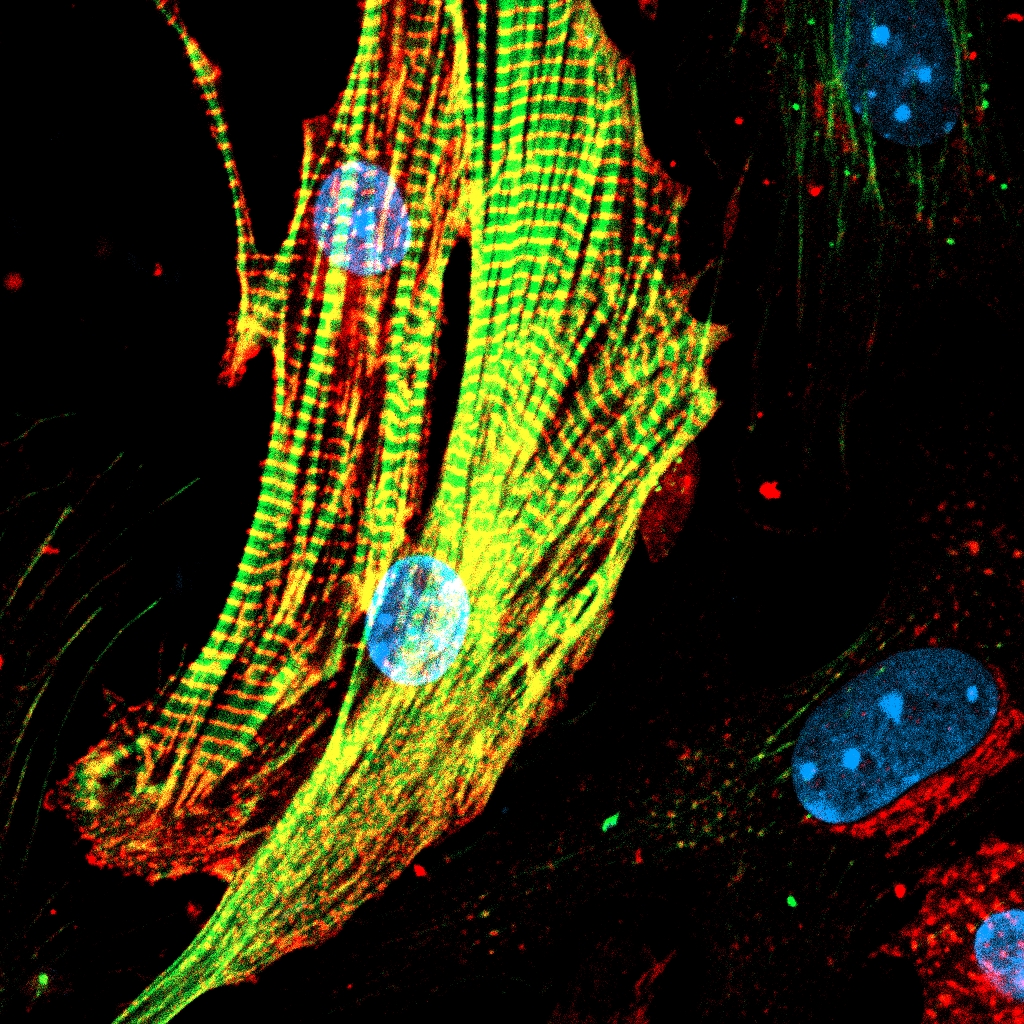

Supplement: Supplementary file 14 — Figure EV2 Source Data [file 44321_2025_334_MOESM14_ESM.zip › EV2C/Ad-NC.jpeg]

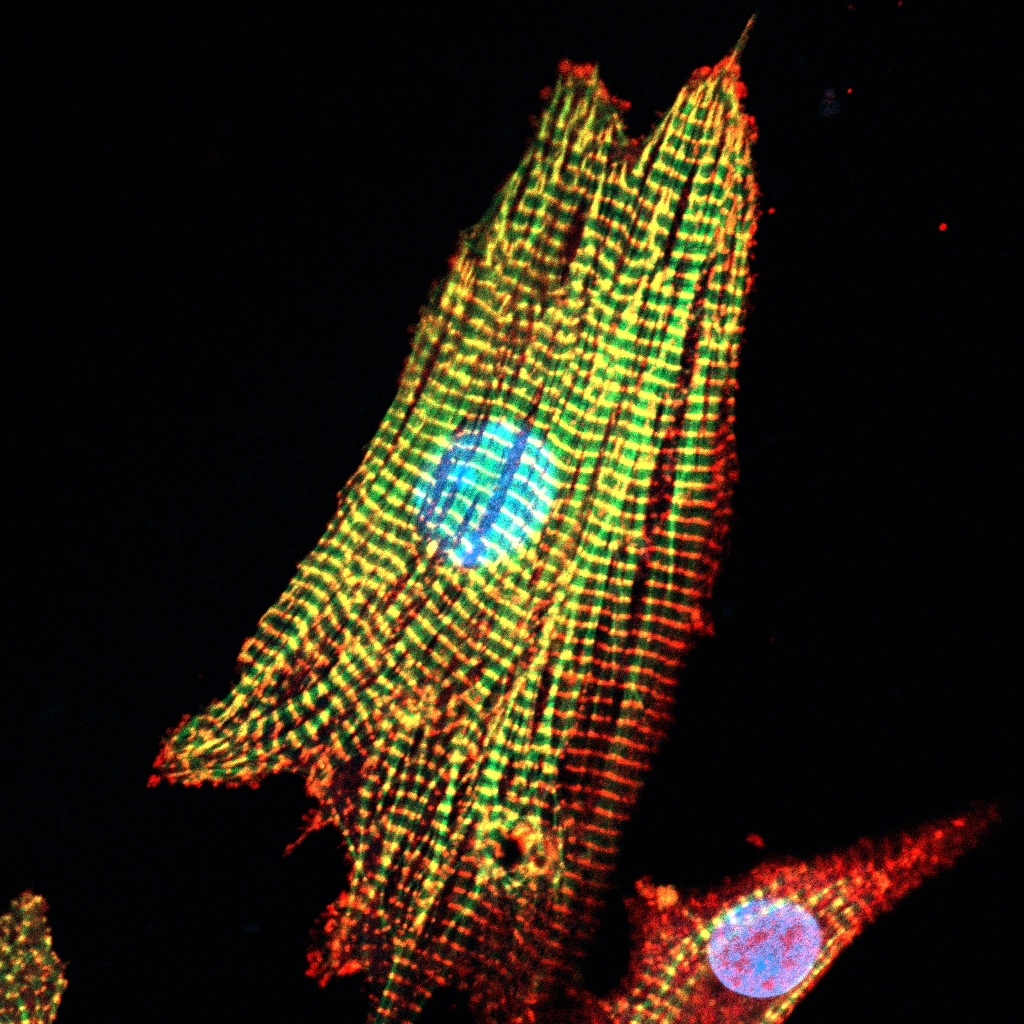

Supplement: Supplementary file 14 — Figure EV2 Source Data [file 44321_2025_334_MOESM14_ESM.zip › EV2C/Ad-RBMS1.jpeg]

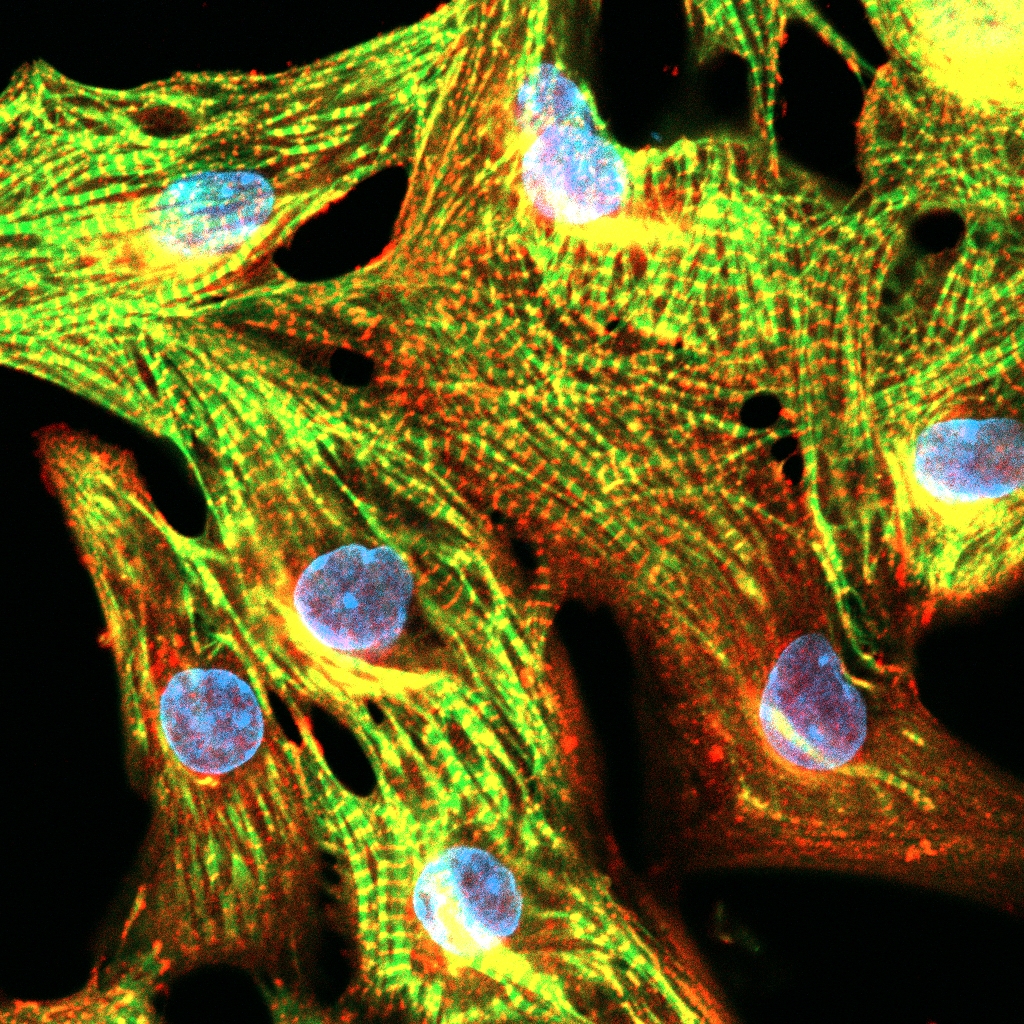

Supplement: Supplementary file 14 — Figure EV2 Source Data [file 44321_2025_334_MOESM14_ESM.zip › EV2C/Ang II+Ad-NC.jpeg]

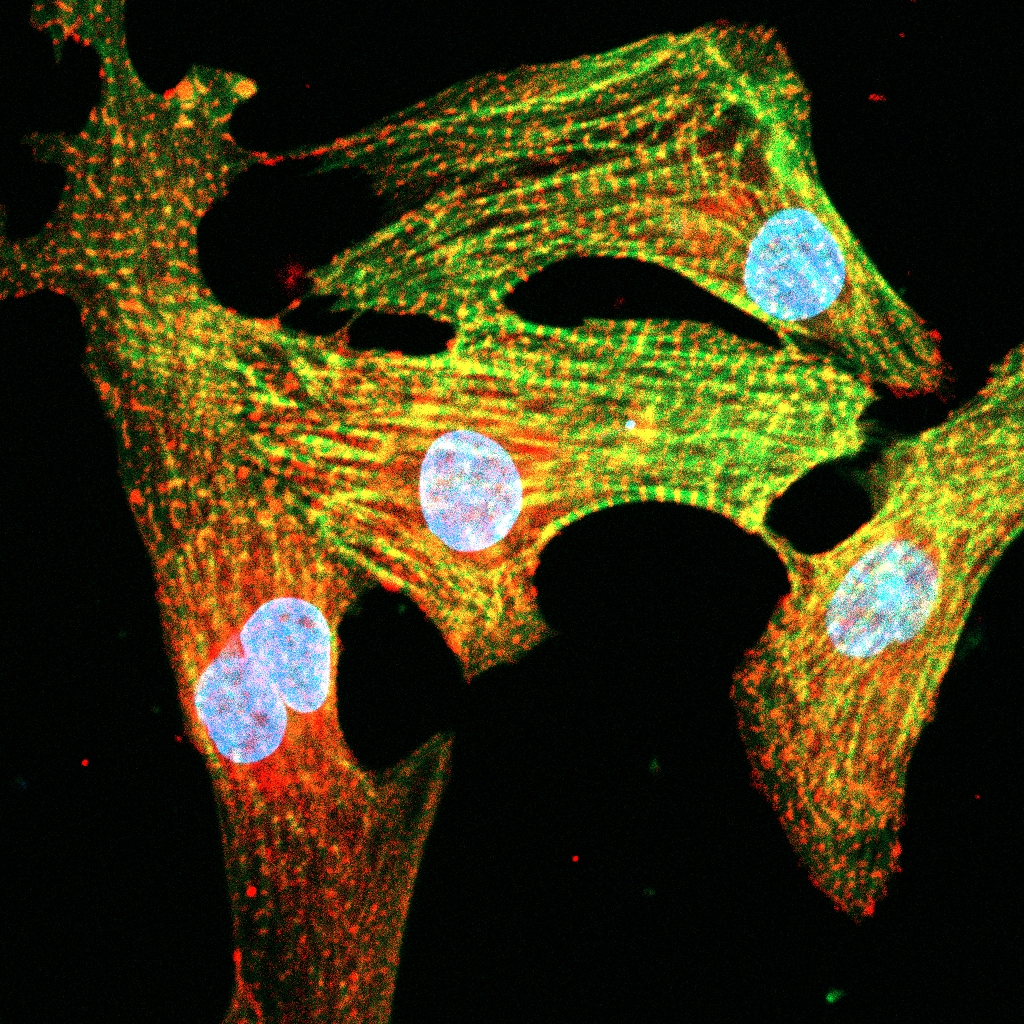

Supplement: Supplementary file 14 — Figure EV2 Source Data [file 44321_2025_334_MOESM14_ESM.zip › EV2C/Ang II+RBMS1.jpeg]

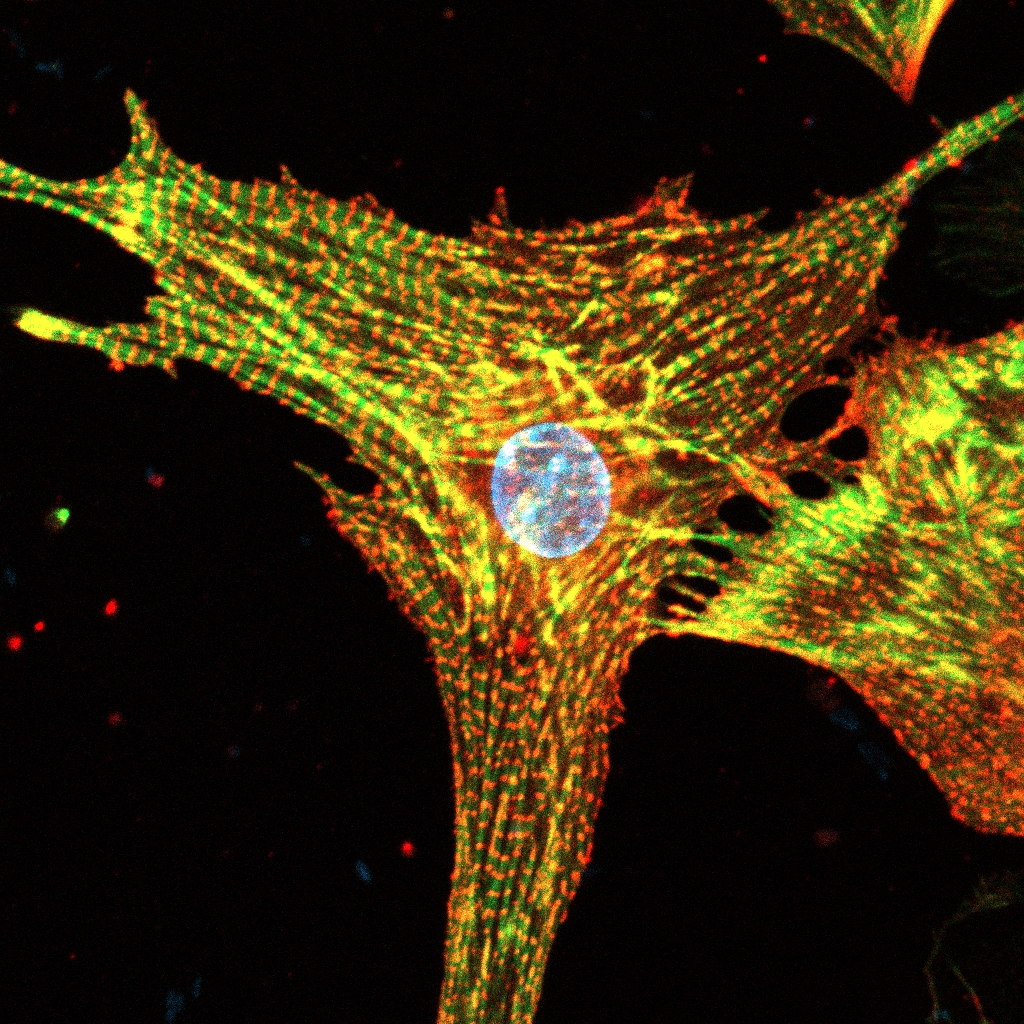

Supplement: Supplementary file 14 — Figure EV2 Source Data [file 44321_2025_334_MOESM14_ESM.zip › EV2E/Ang II+si-NC.jpeg]

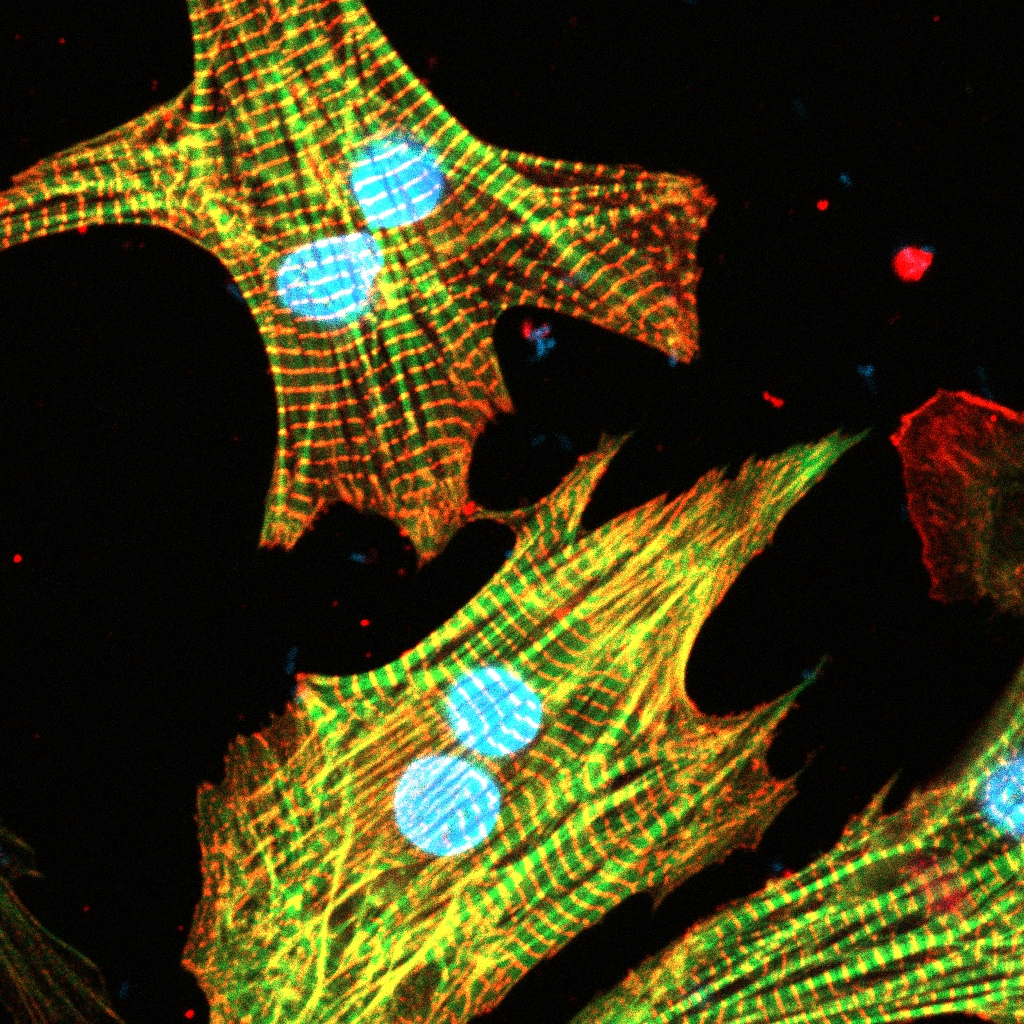

Supplement: Supplementary file 14 — Figure EV2 Source Data [file 44321_2025_334_MOESM14_ESM.zip › EV2E/Ang II+si-RBMS1.jpeg]

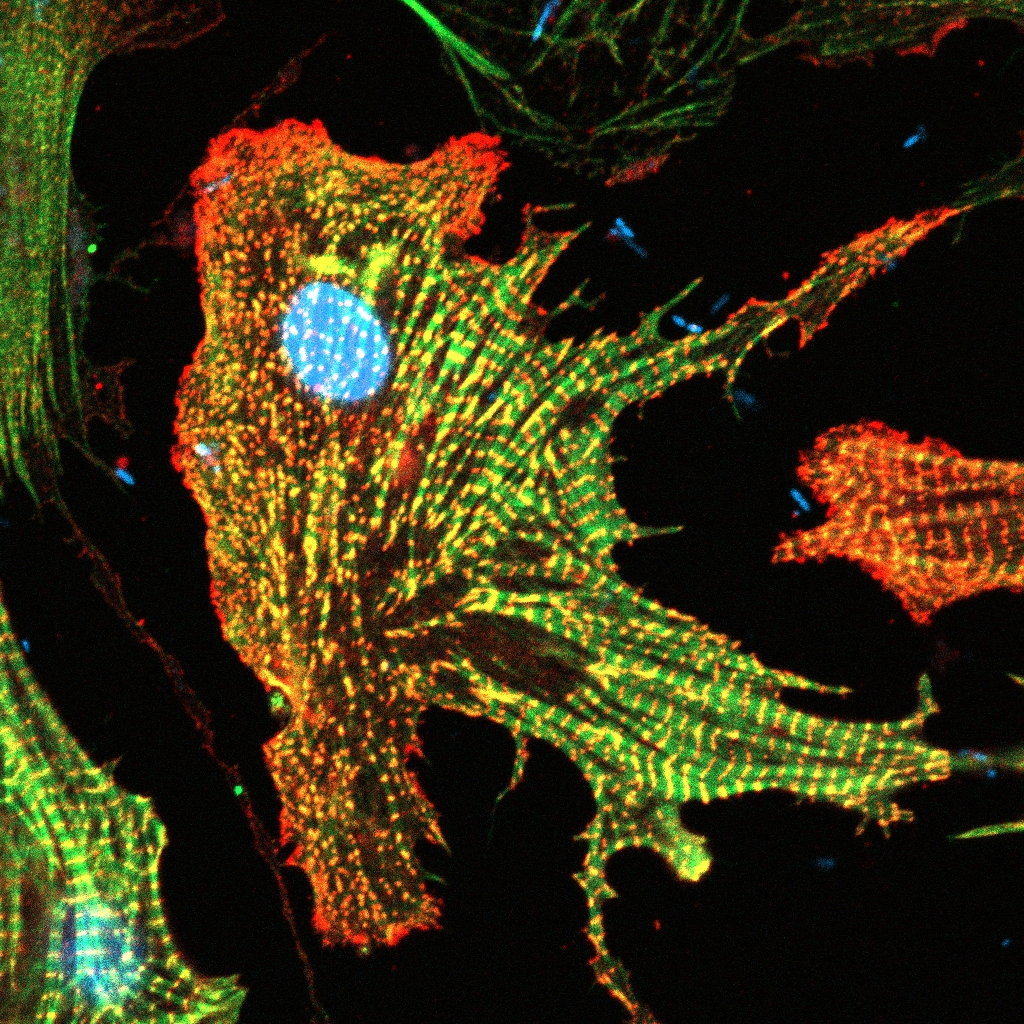

Supplement: Supplementary file 14 — Figure EV2 Source Data [file 44321_2025_334_MOESM14_ESM.zip › EV2E/Ang II.jpeg]

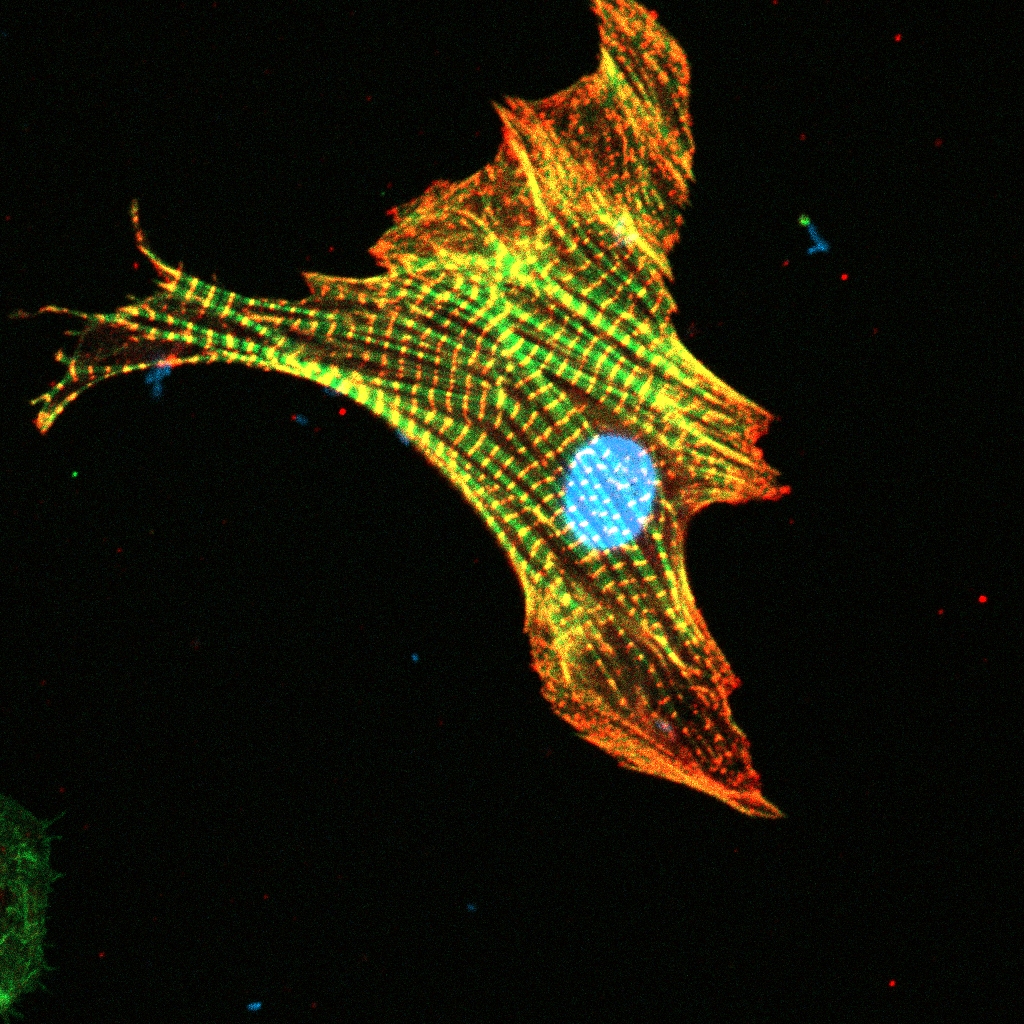

Supplement: Supplementary file 14 — Figure EV2 Source Data [file 44321_2025_334_MOESM14_ESM.zip › EV2E/Control.jpeg]

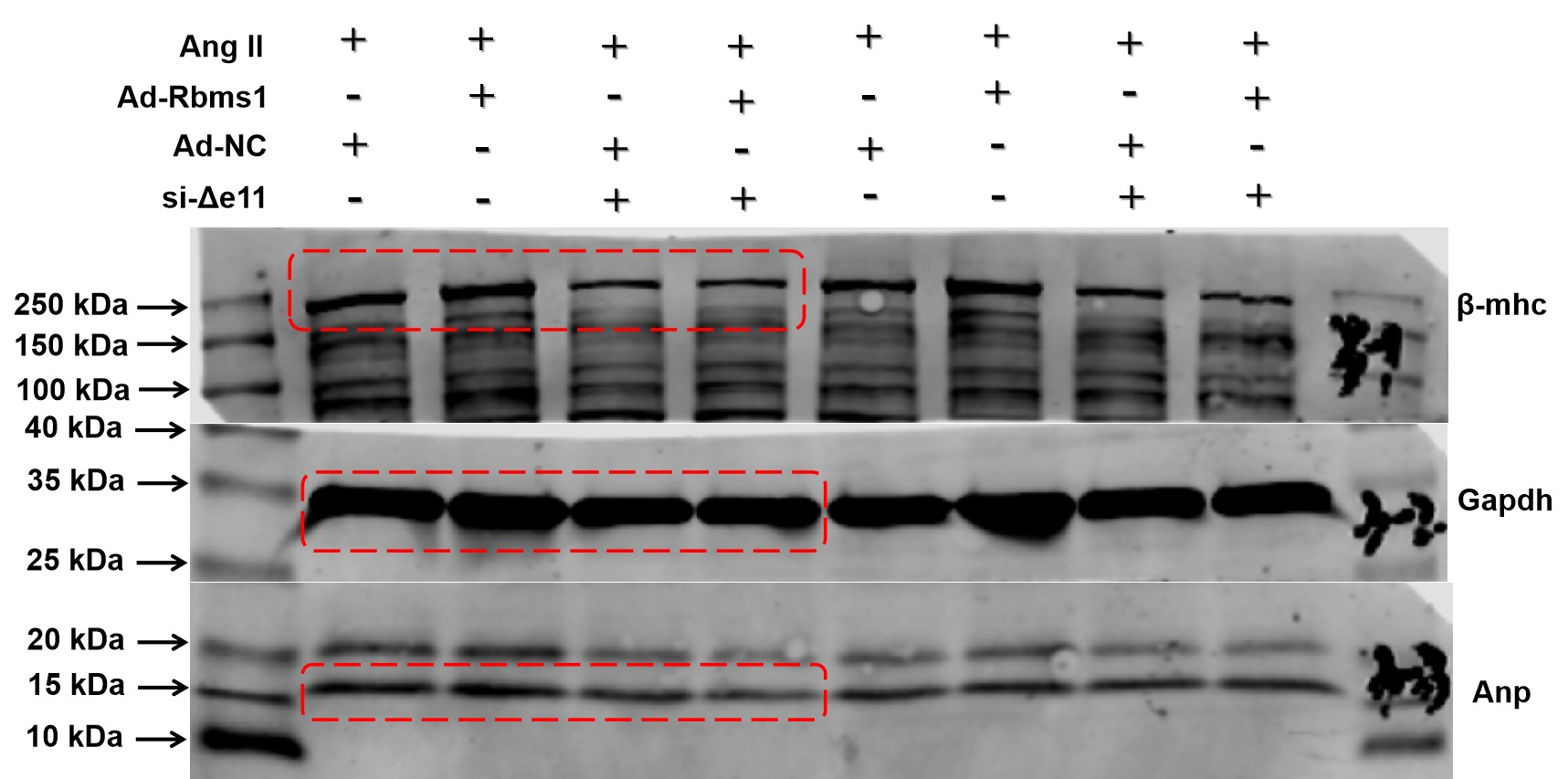

Supplement: Supplementary file 15 — Figure EV3 Source Data [file 44321_2025_334_MOESM15_ESM.zip › EV3A/EV3A.jpg]

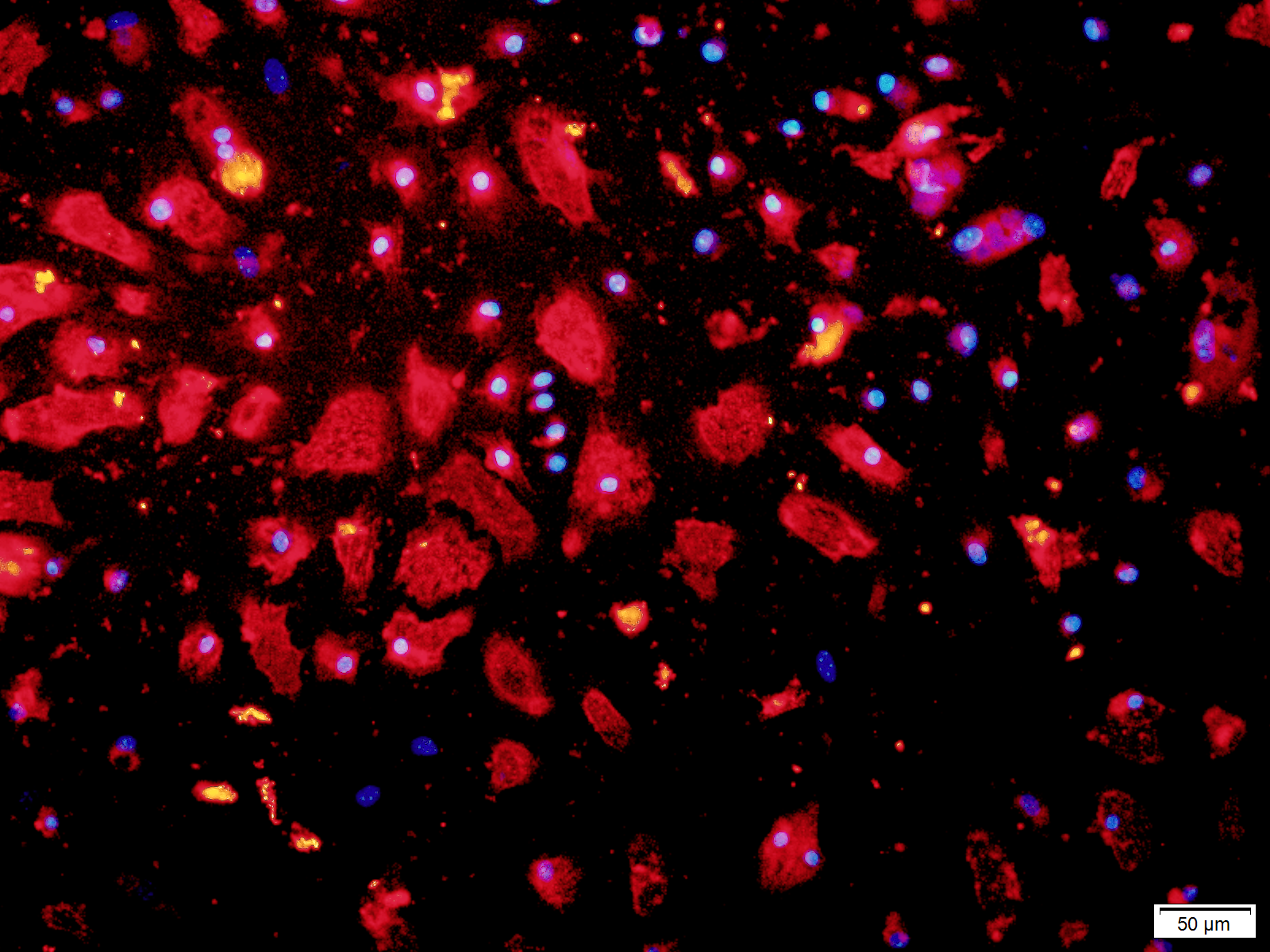

Supplement: Supplementary file 15 — Figure EV3 Source Data [file 44321_2025_334_MOESM15_ESM.zip › EV3C/Ang II+Ad-NC+si-NC.tif]

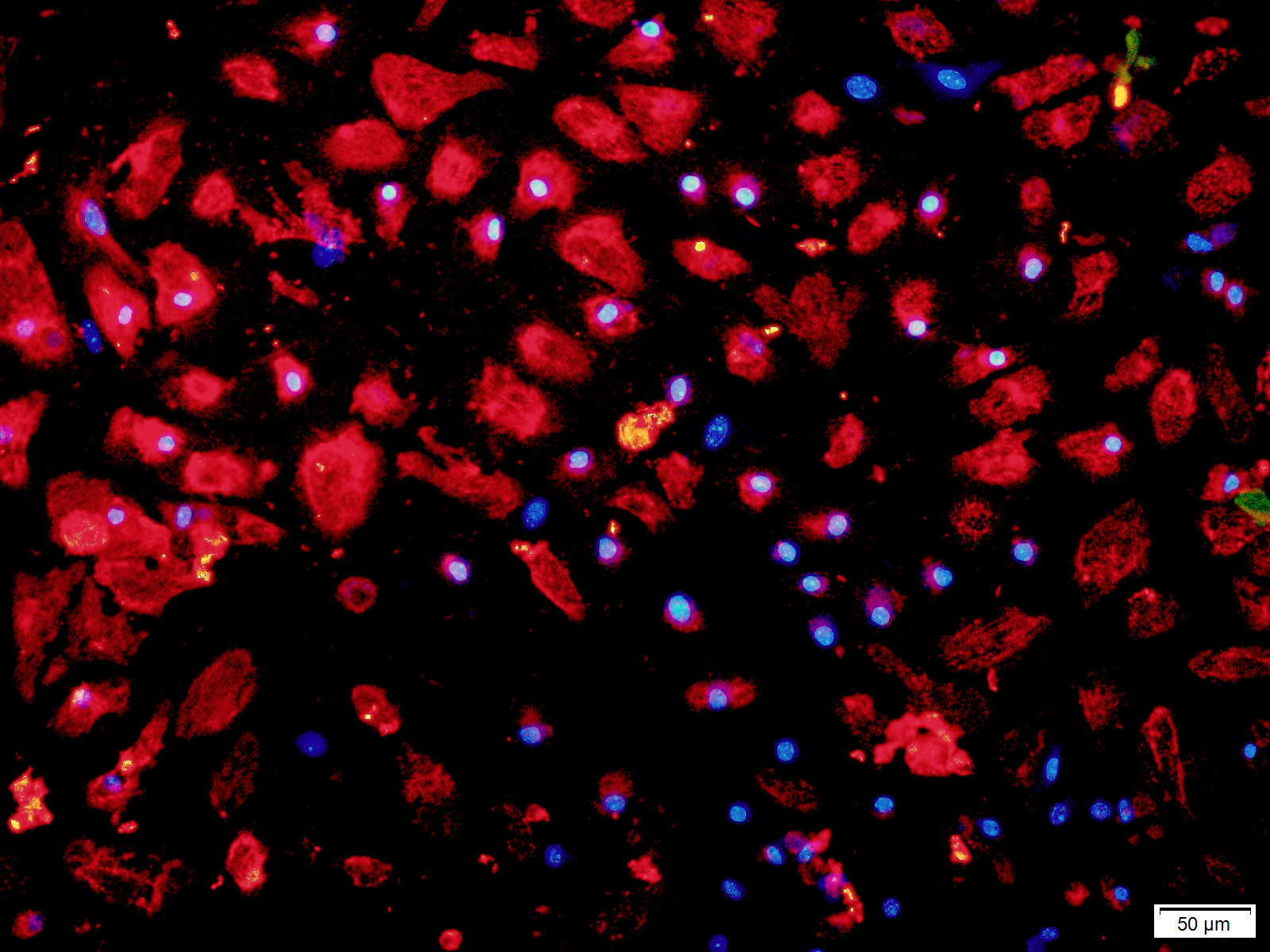

Supplement: Supplementary file 15 — Figure EV3 Source Data [file 44321_2025_334_MOESM15_ESM.zip › EV3C/Ang II+Ad-NC+si-Δe11.tif]

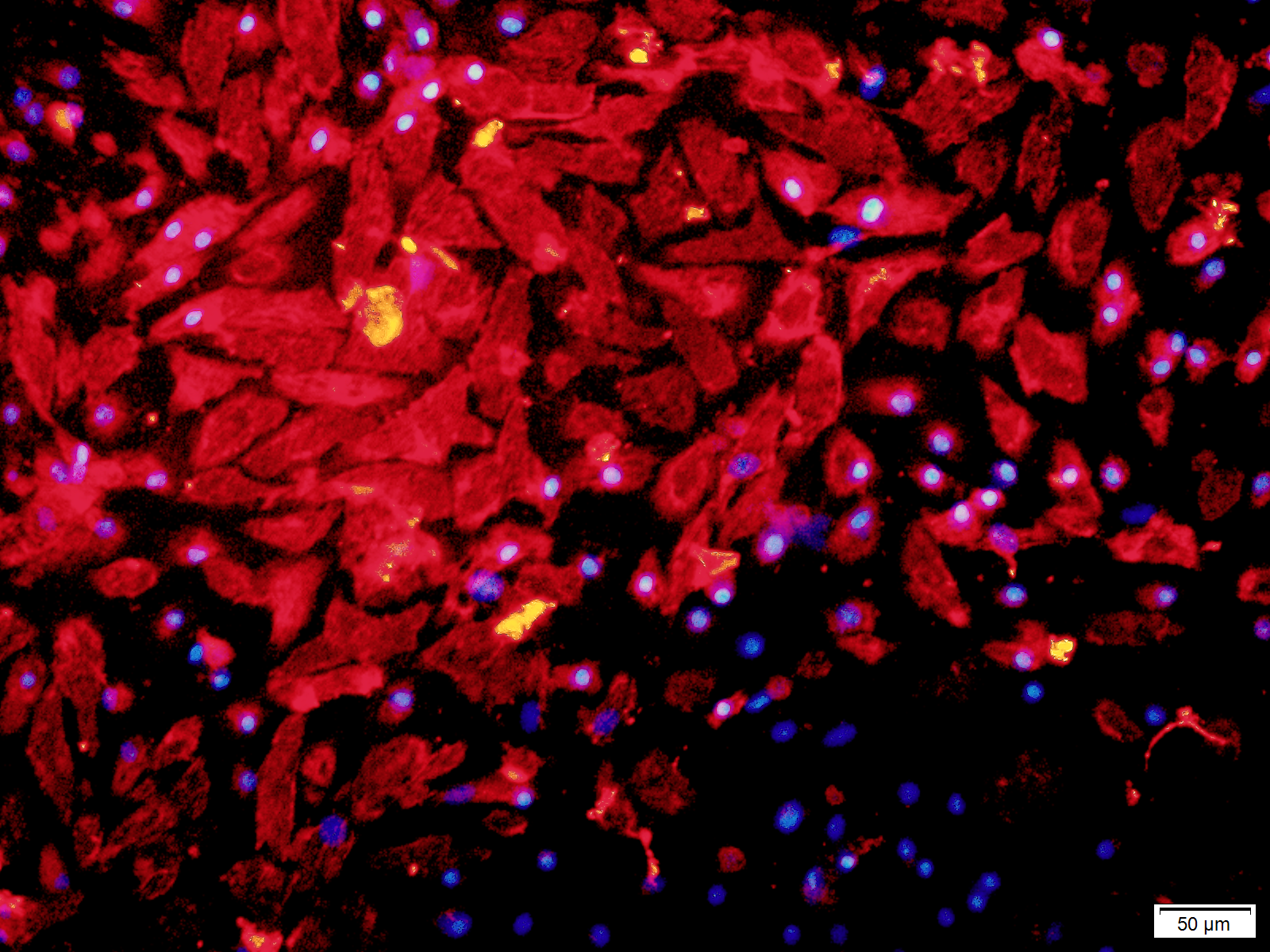

Supplement: Supplementary file 15 — Figure EV3 Source Data [file 44321_2025_334_MOESM15_ESM.zip › EV3C/Ang II+Ad-RBMS1+si-NC.tif]

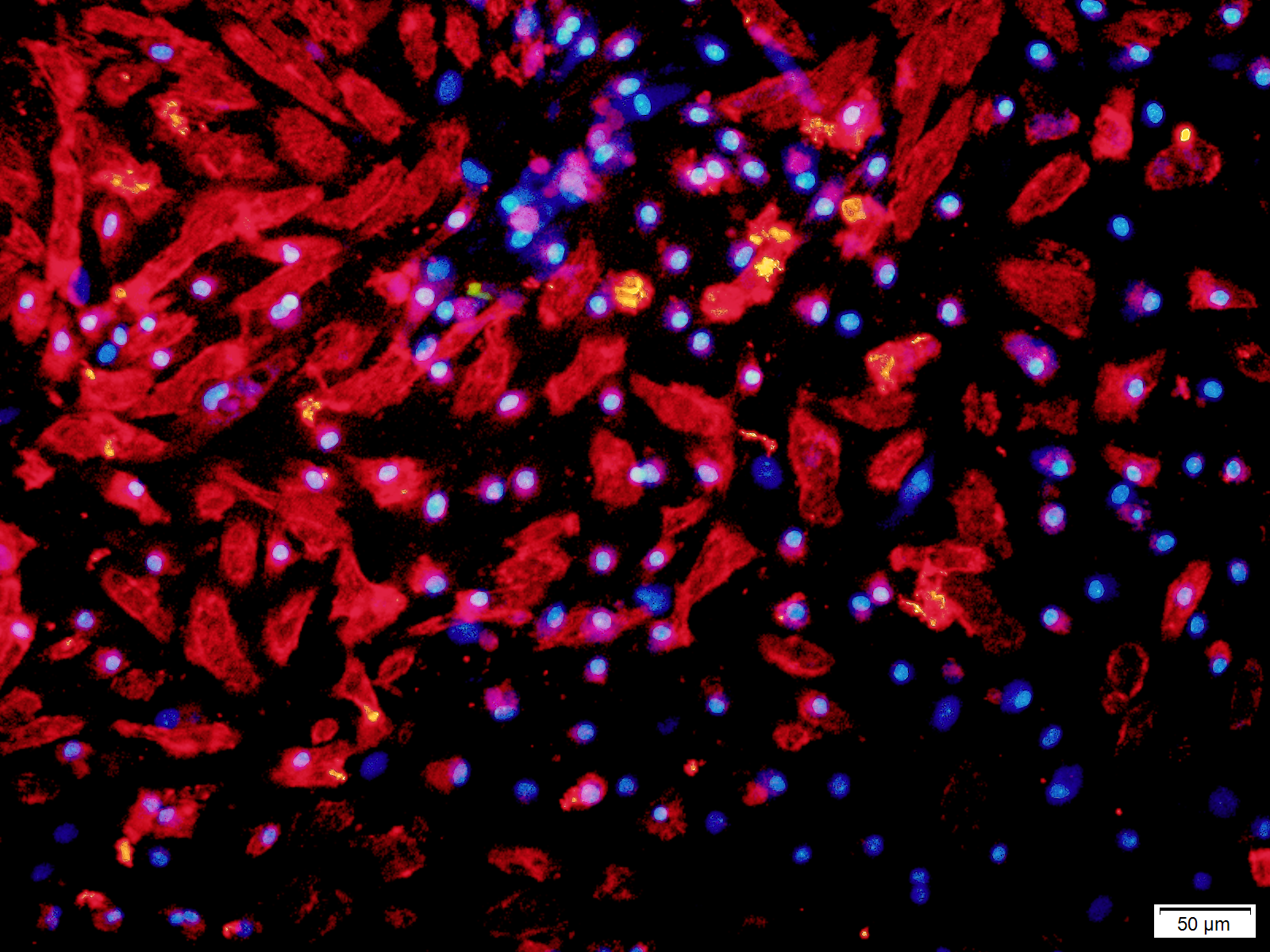

Supplement: Supplementary file 15 — Figure EV3 Source Data [file 44321_2025_334_MOESM15_ESM.zip › EV3C/Ang II+Ad-RBMS1+si-Δe11.tif]

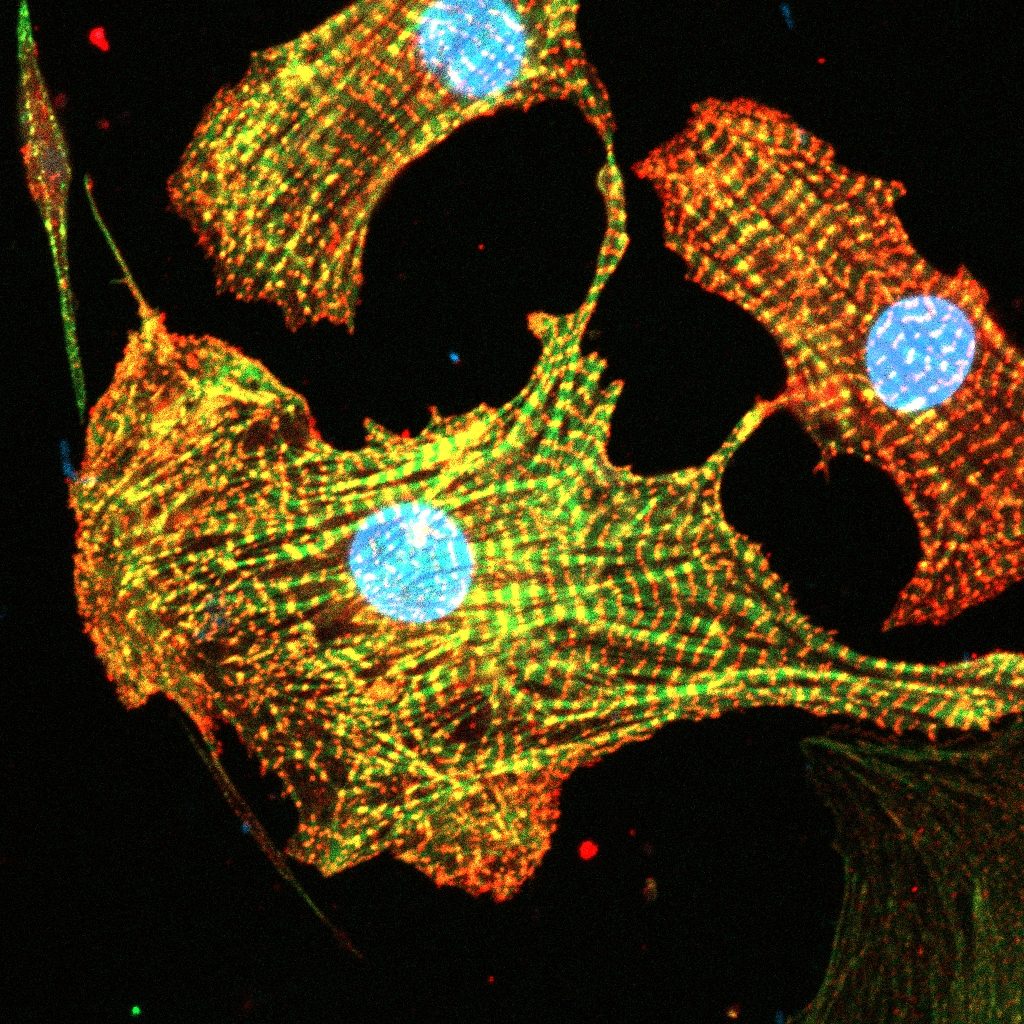

Supplement: Supplementary file 15 — Figure EV3 Source Data [file 44321_2025_334_MOESM15_ESM.zip › EV3E/Ang II+Ad-NC+si-NC.jpeg]

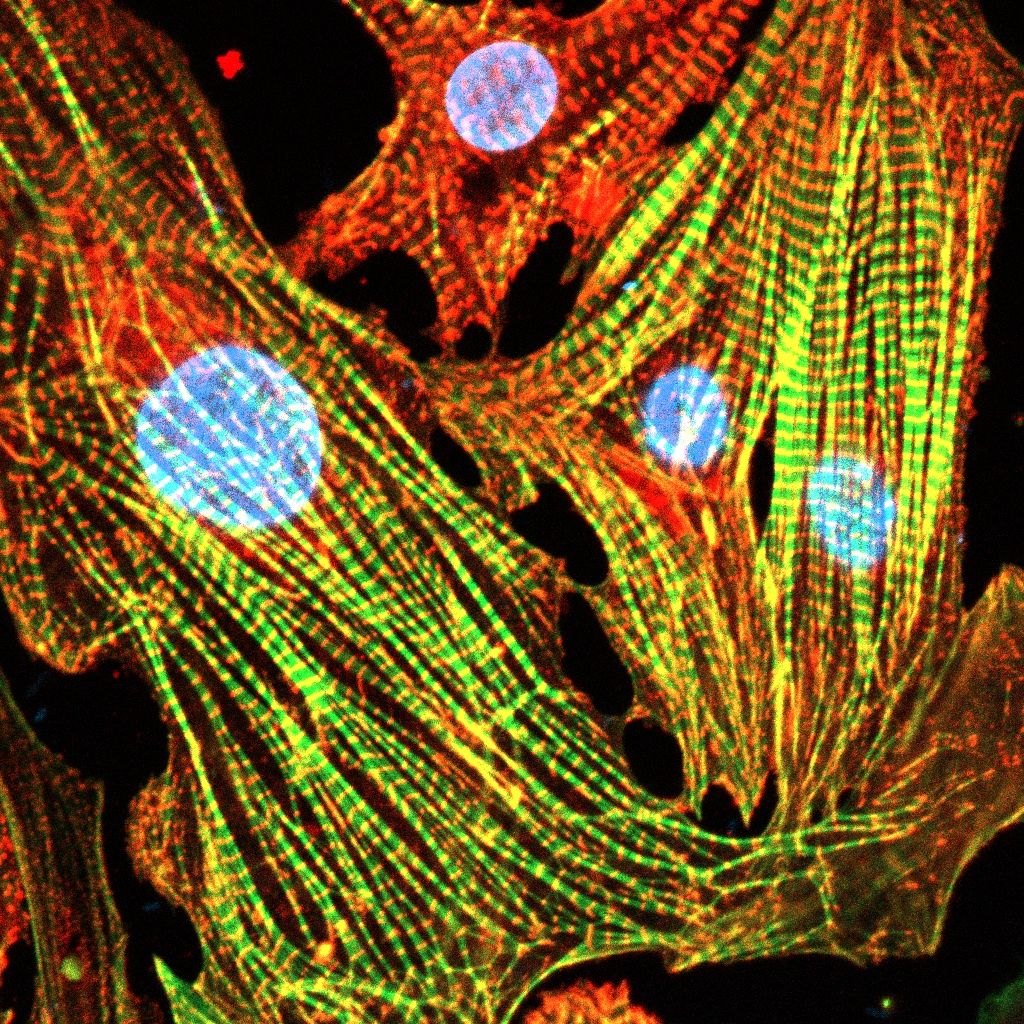

Supplement: Supplementary file 15 — Figure EV3 Source Data [file 44321_2025_334_MOESM15_ESM.zip › EV3E/Ang II+Ad-NC+si-Δe11.jpeg]

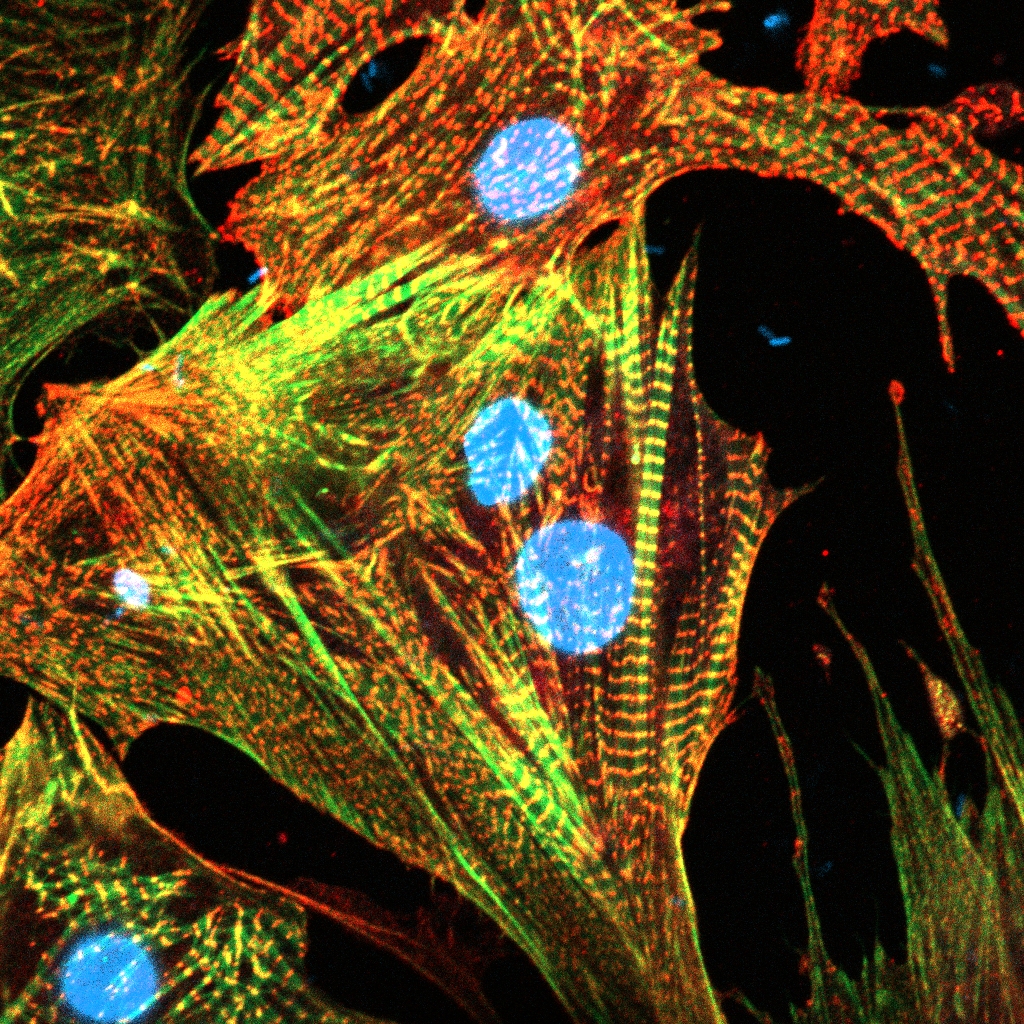

Supplement: Supplementary file 15 — Figure EV3 Source Data [file 44321_2025_334_MOESM15_ESM.zip › EV3E/Ang II+Ad-RBMS1+si-NC.jpeg]

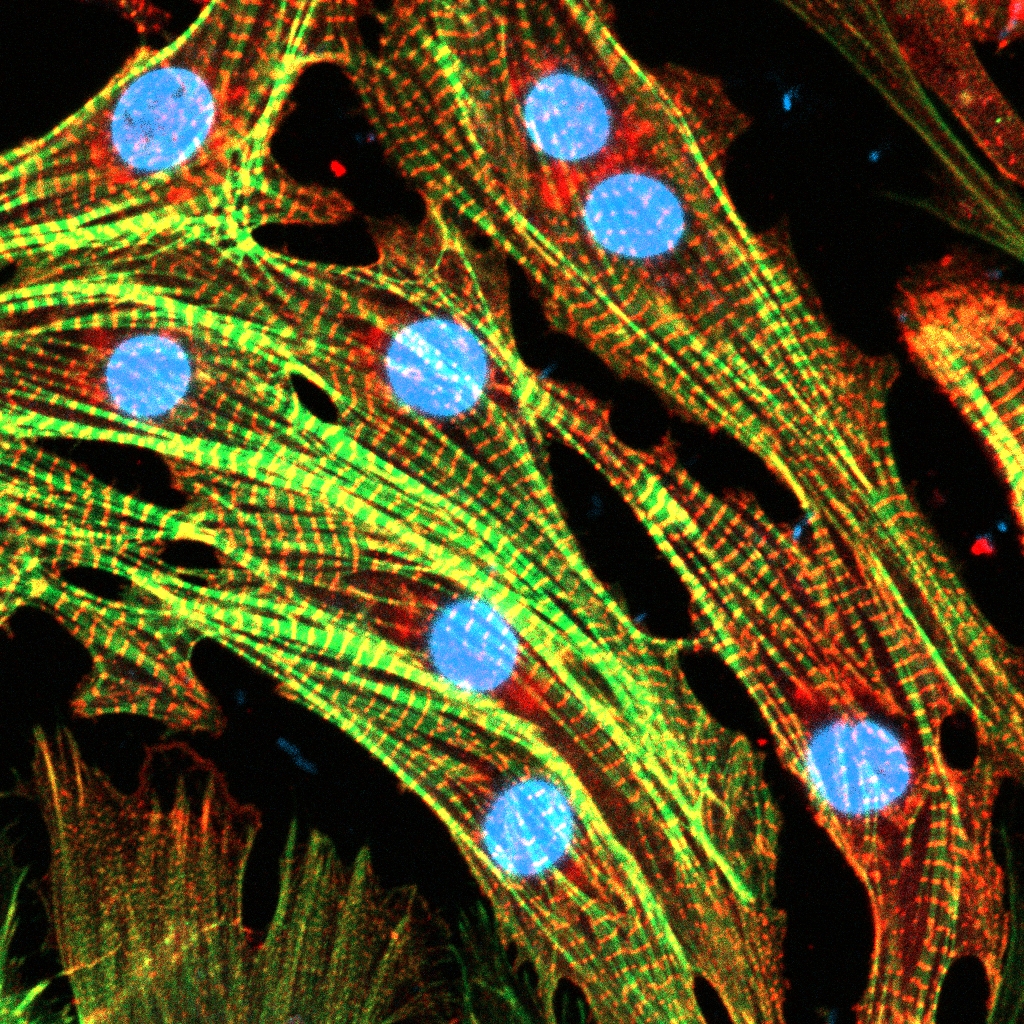

Supplement: Supplementary file 15 — Figure EV3 Source Data [file 44321_2025_334_MOESM15_ESM.zip › EV3E/Ang II+Ad-RBMS1+si-Δe11.jpeg]

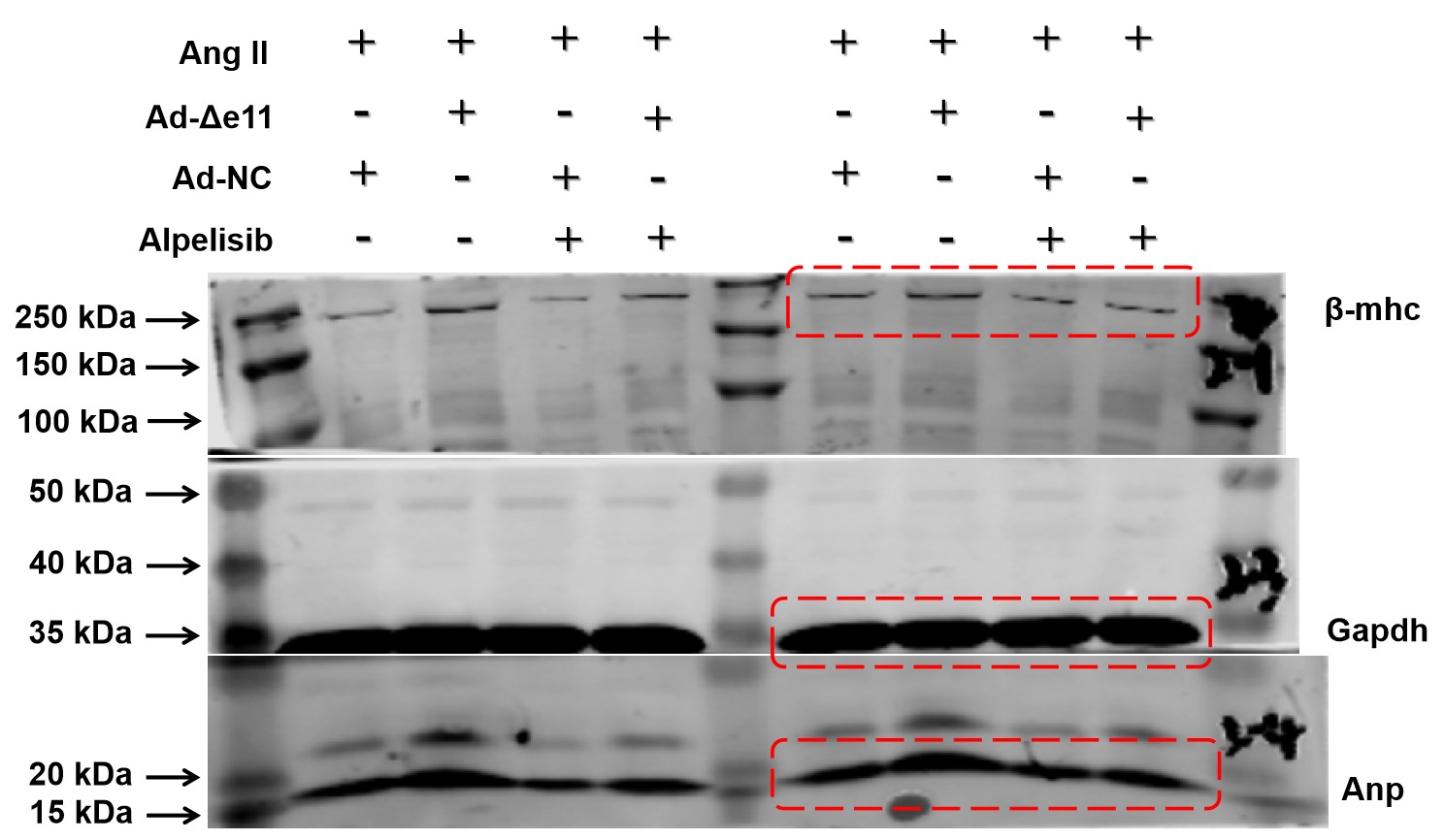

Supplement: Supplementary file 16 — Figure EV4 Source Data [file 44321_2025_334_MOESM16_ESM.zip › EV4A/图片4.jpg]

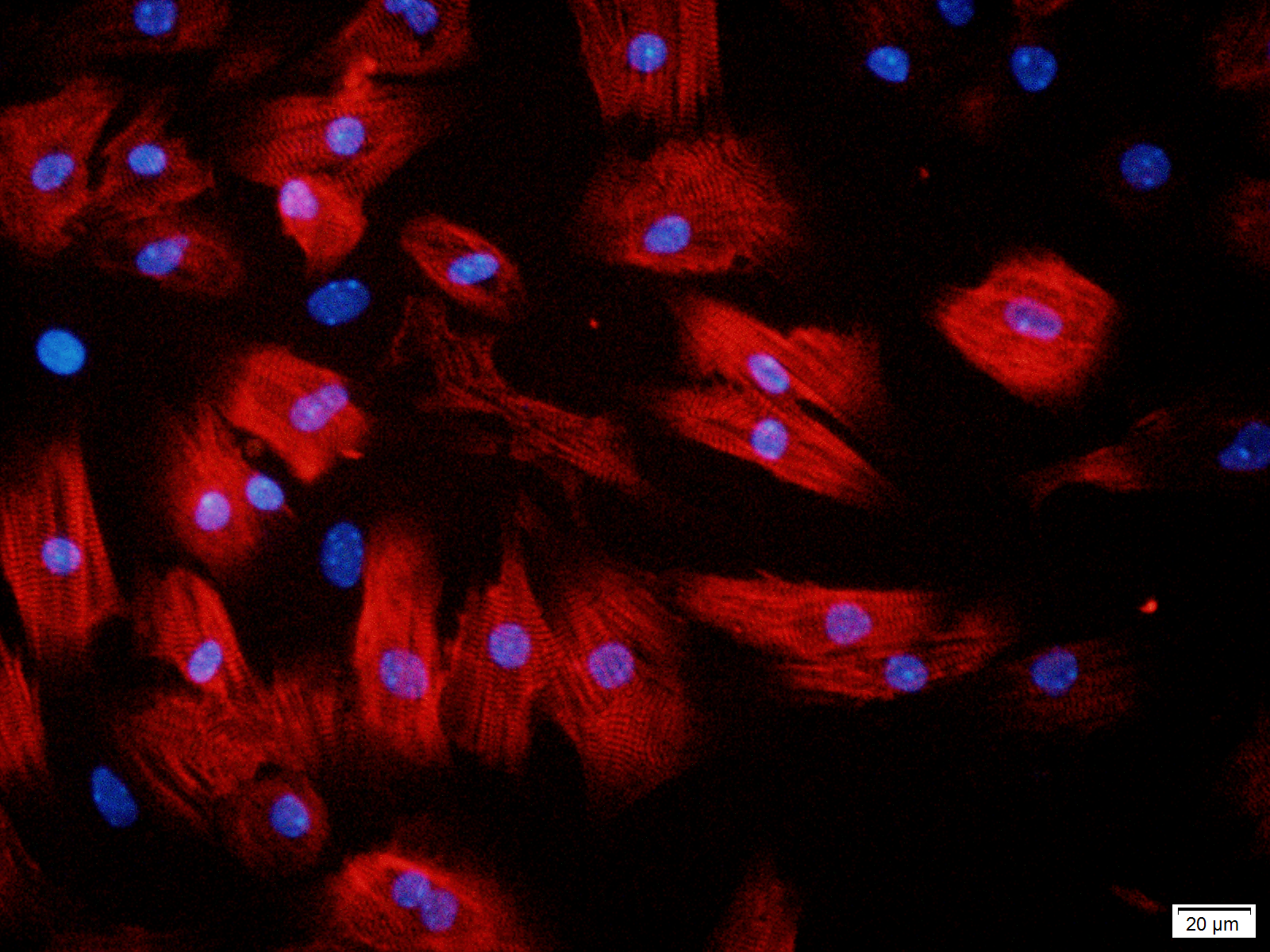

Supplement: Supplementary file 16 — Figure EV4 Source Data [file 44321_2025_334_MOESM16_ESM.zip › EV4C/Ang II+Alpelisib+Ad-NC.tif]

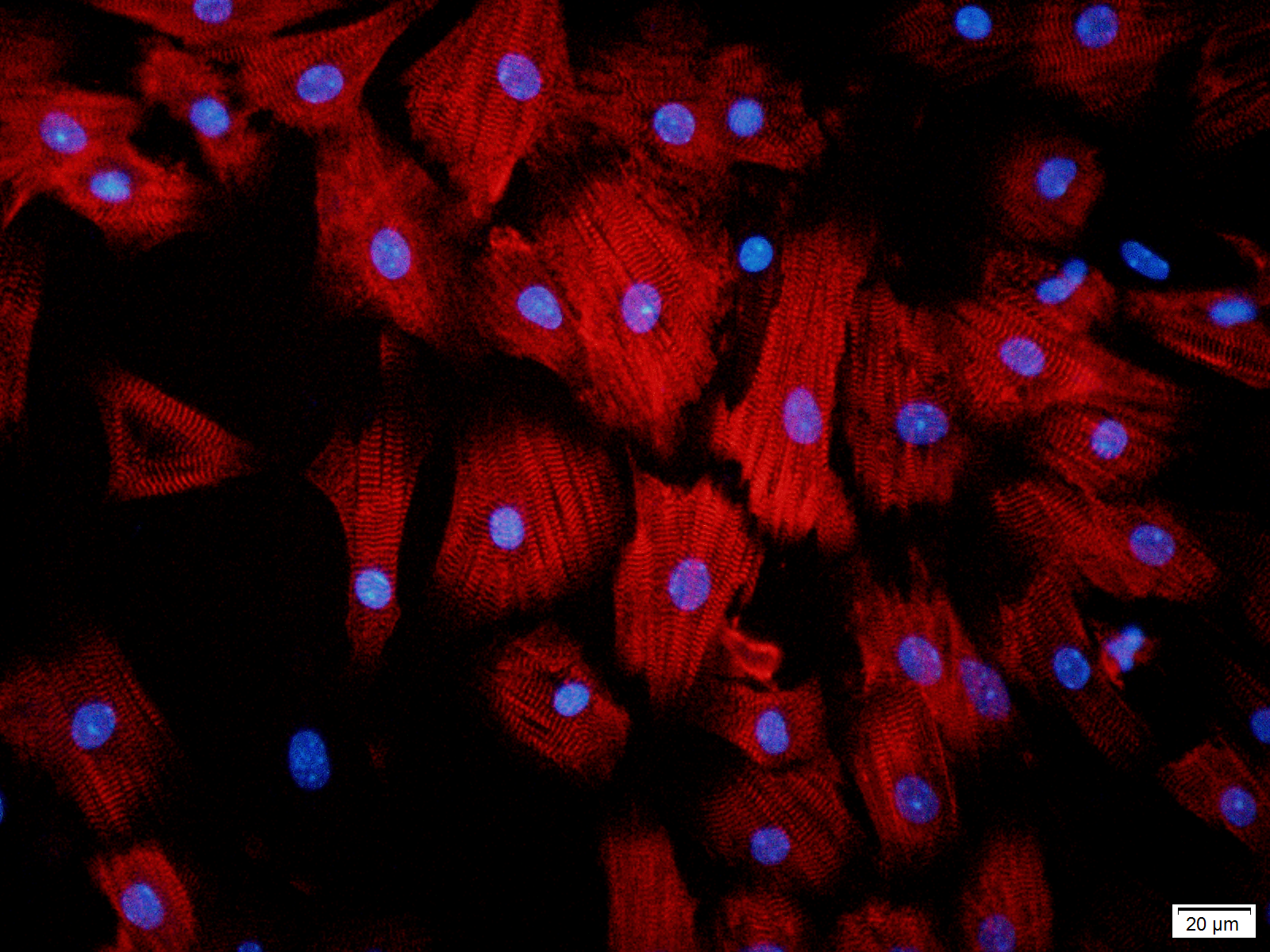

Supplement: Supplementary file 16 — Figure EV4 Source Data [file 44321_2025_334_MOESM16_ESM.zip › EV4C/Ang II+Alpelisib+Ad-Δe11.tif]

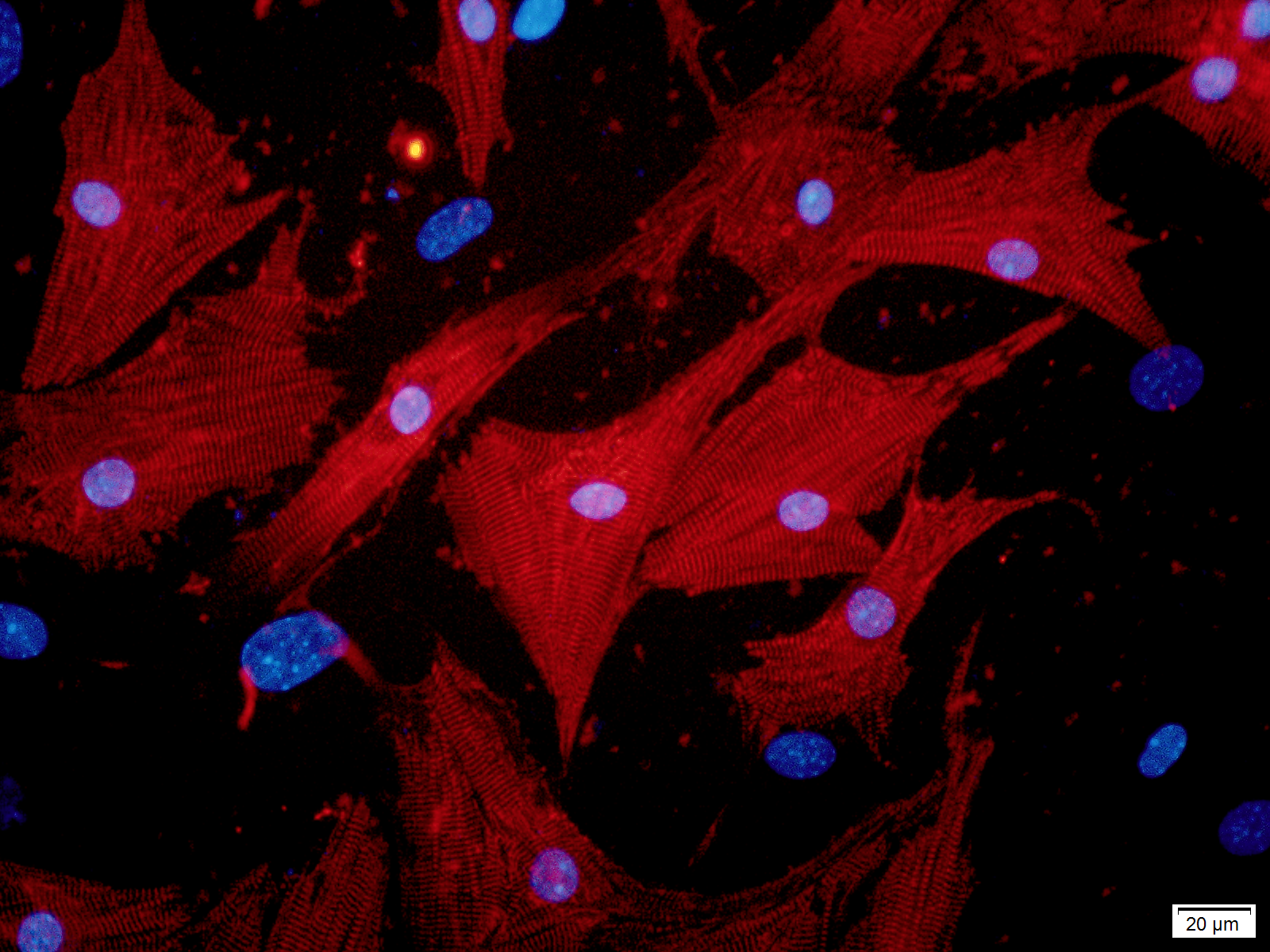

Supplement: Supplementary file 16 — Figure EV4 Source Data [file 44321_2025_334_MOESM16_ESM.zip › EV4C/Ang II+DMSO+Ad-NC.tif]

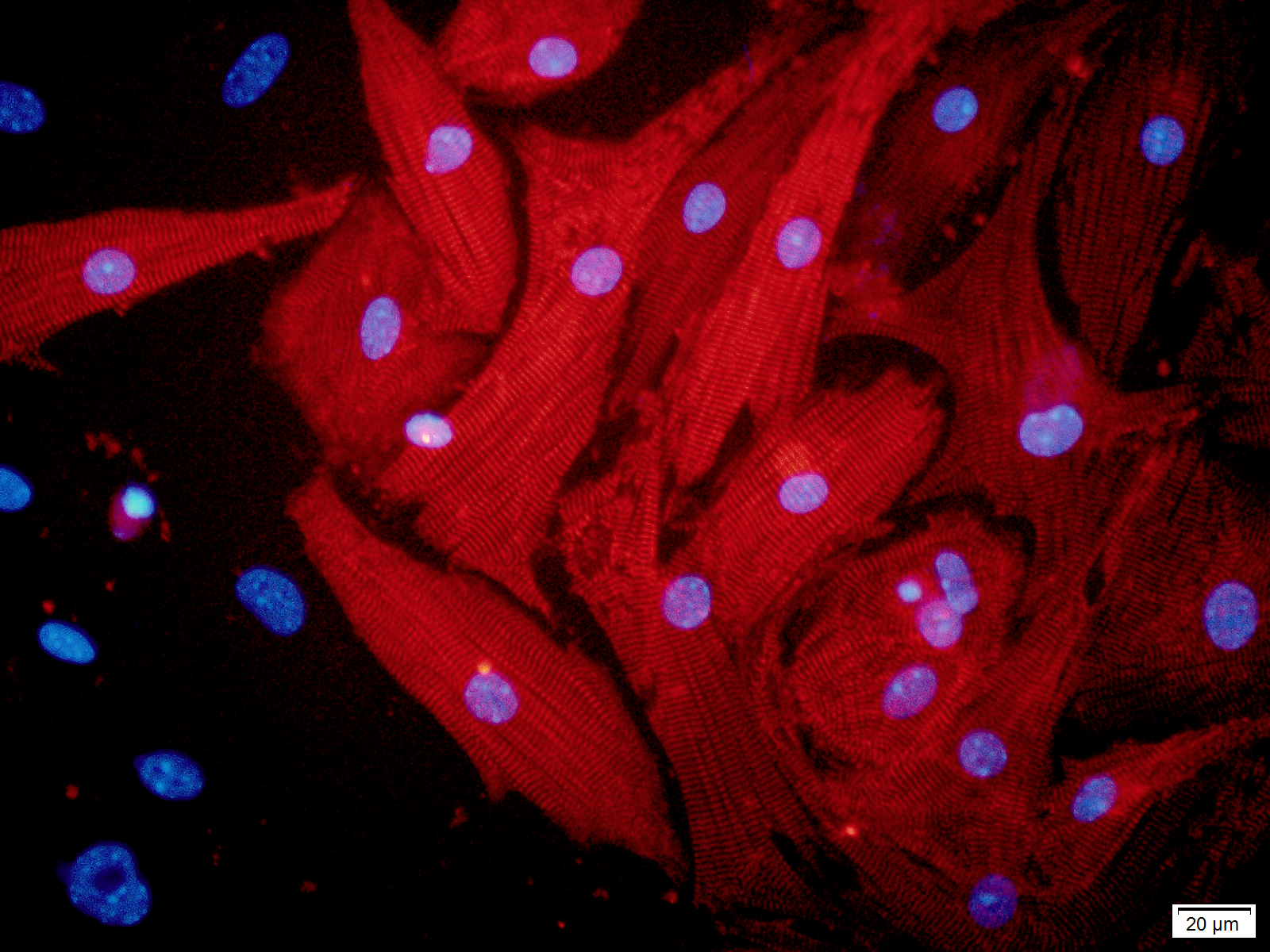

Supplement: Supplementary file 16 — Figure EV4 Source Data [file 44321_2025_334_MOESM16_ESM.zip › EV4C/Ang II+DMSO+Ad-Δe11.tif]

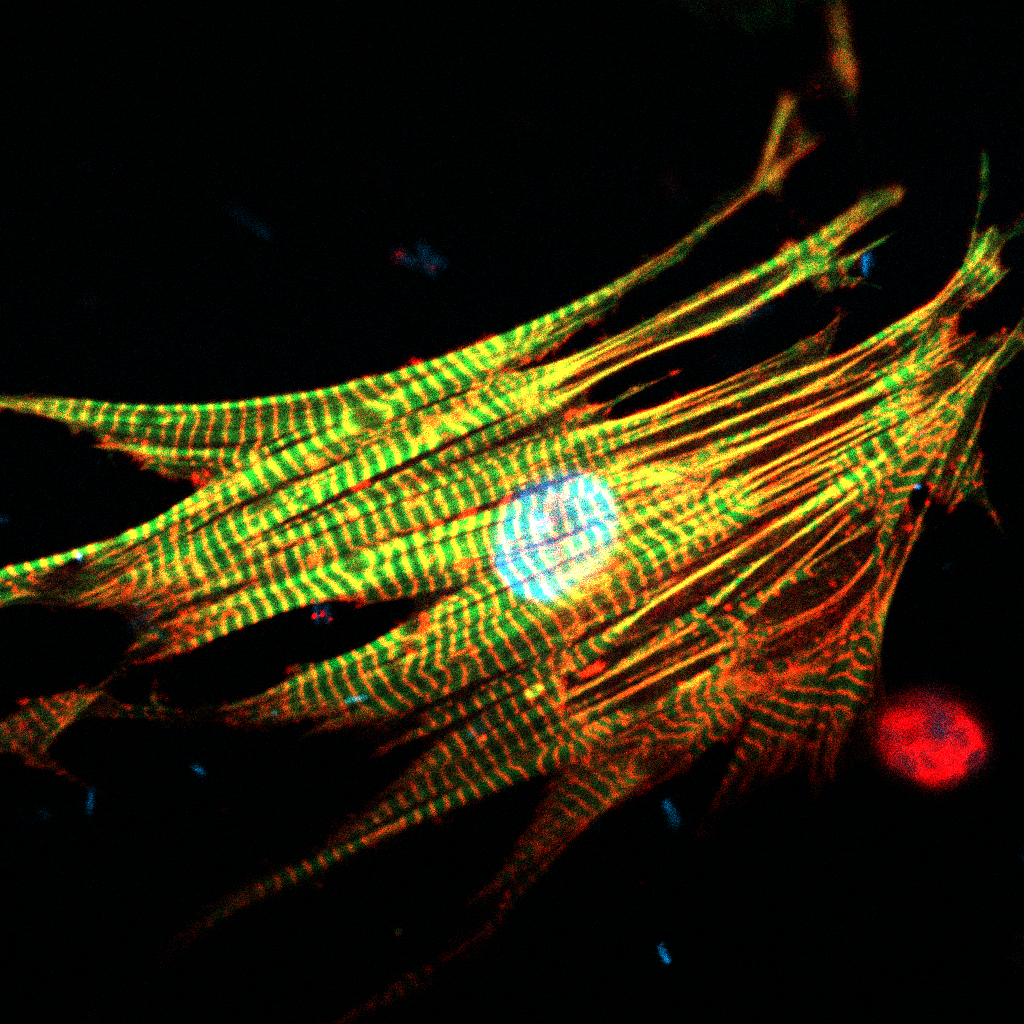

Supplement: Supplementary file 16 — Figure EV4 Source Data [file 44321_2025_334_MOESM16_ESM.zip › EV4E/Ang II+Alpelisib+Ad-NC.tiff]

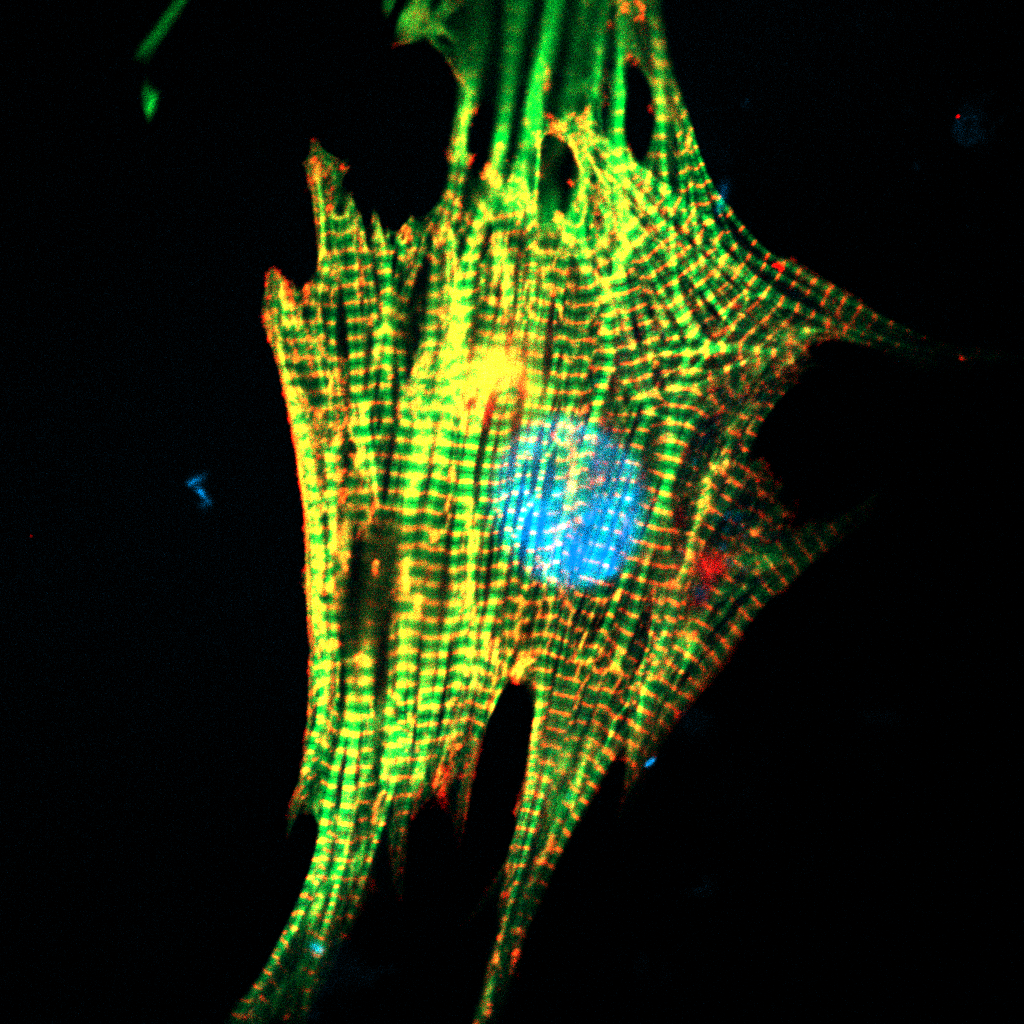

Supplement: Supplementary file 16 — Figure EV4 Source Data [file 44321_2025_334_MOESM16_ESM.zip › EV4E/Ang II+Alpelisib+Ad-Δe11.tiff]

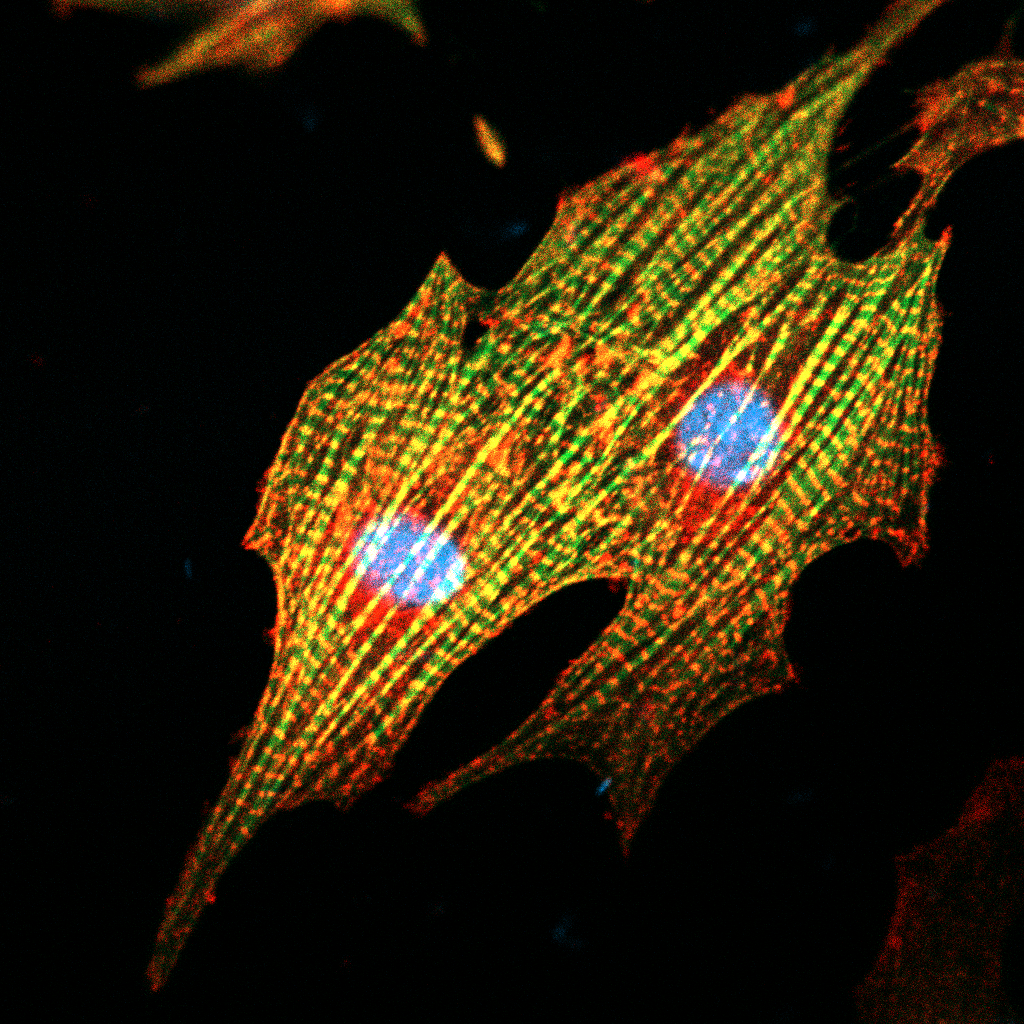

Supplement: Supplementary file 16 — Figure EV4 Source Data [file 44321_2025_334_MOESM16_ESM.zip › EV4E/Ang II+DMSO+Ad-NC.tiff]

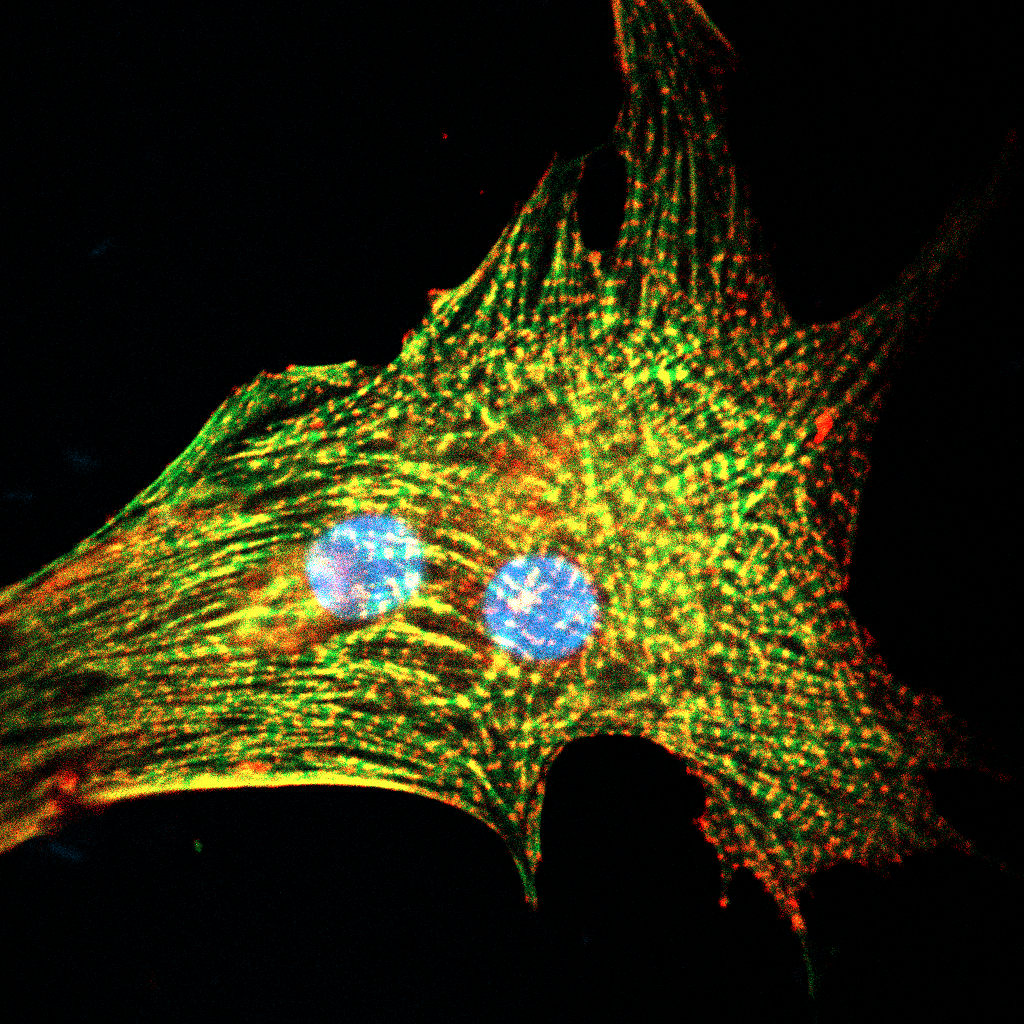

Supplement: Supplementary file 16 — Figure EV4 Source Data [file 44321_2025_334_MOESM16_ESM.zip › EV4E/Ang II+DMSO+Ad-Δe11.tiff]

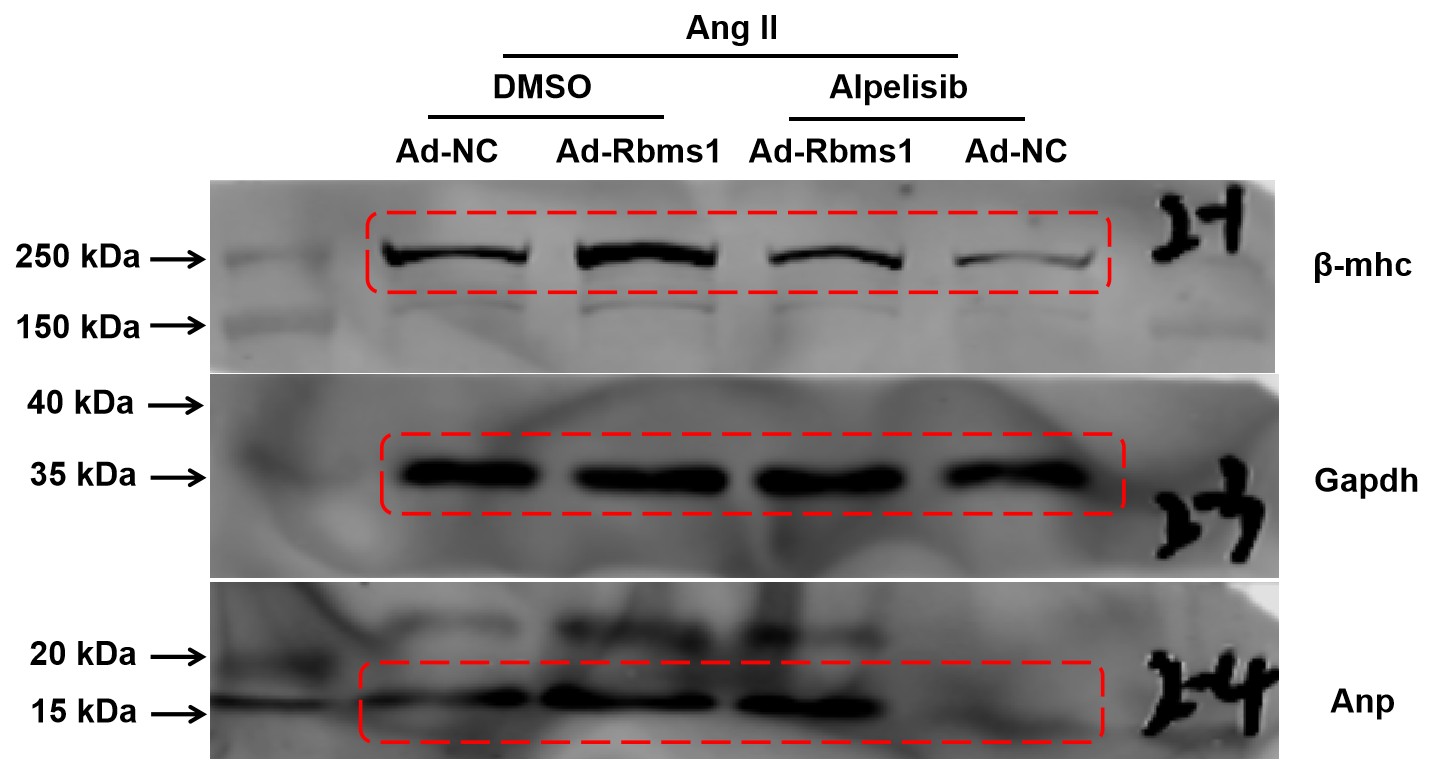

Supplement: Supplementary file 17 — Appendix Figure Source Data [file 44321_2025_334_MOESM17_ESM.zip › Original Blot Source Data/Appendix Fig. S10B/Appendix Fig. S10B.jpg]

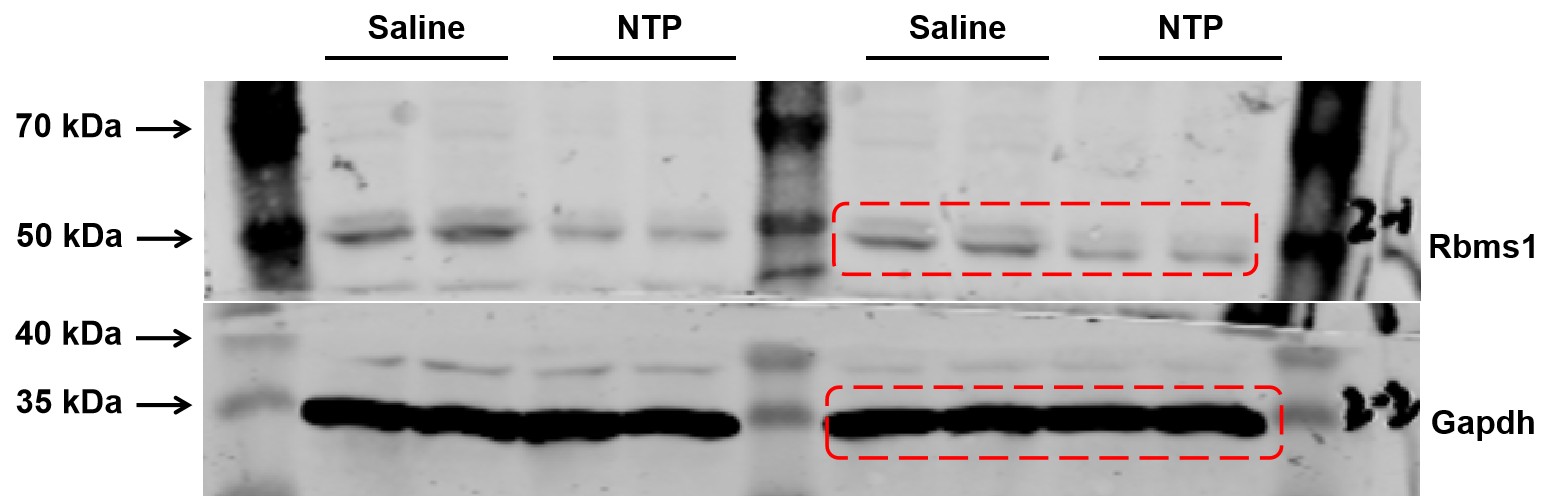

Supplement: Supplementary file 17 — Appendix Figure Source Data [file 44321_2025_334_MOESM17_ESM.zip › Original Blot Source Data/Appendix Fig. S11B/Appendix Fig. S11B.jpg]

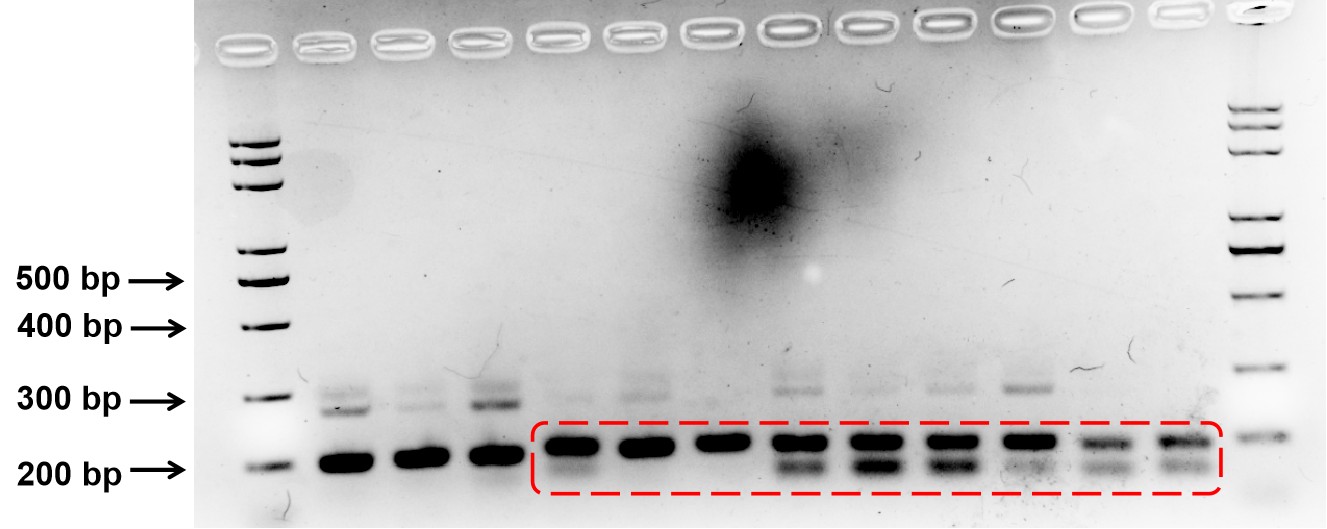

Supplement: Supplementary file 17 — Appendix Figure Source Data [file 44321_2025_334_MOESM17_ESM.zip › Original Blot Source Data/Appendix Fig. S11N/Appendix Fig. S11N.jpg]

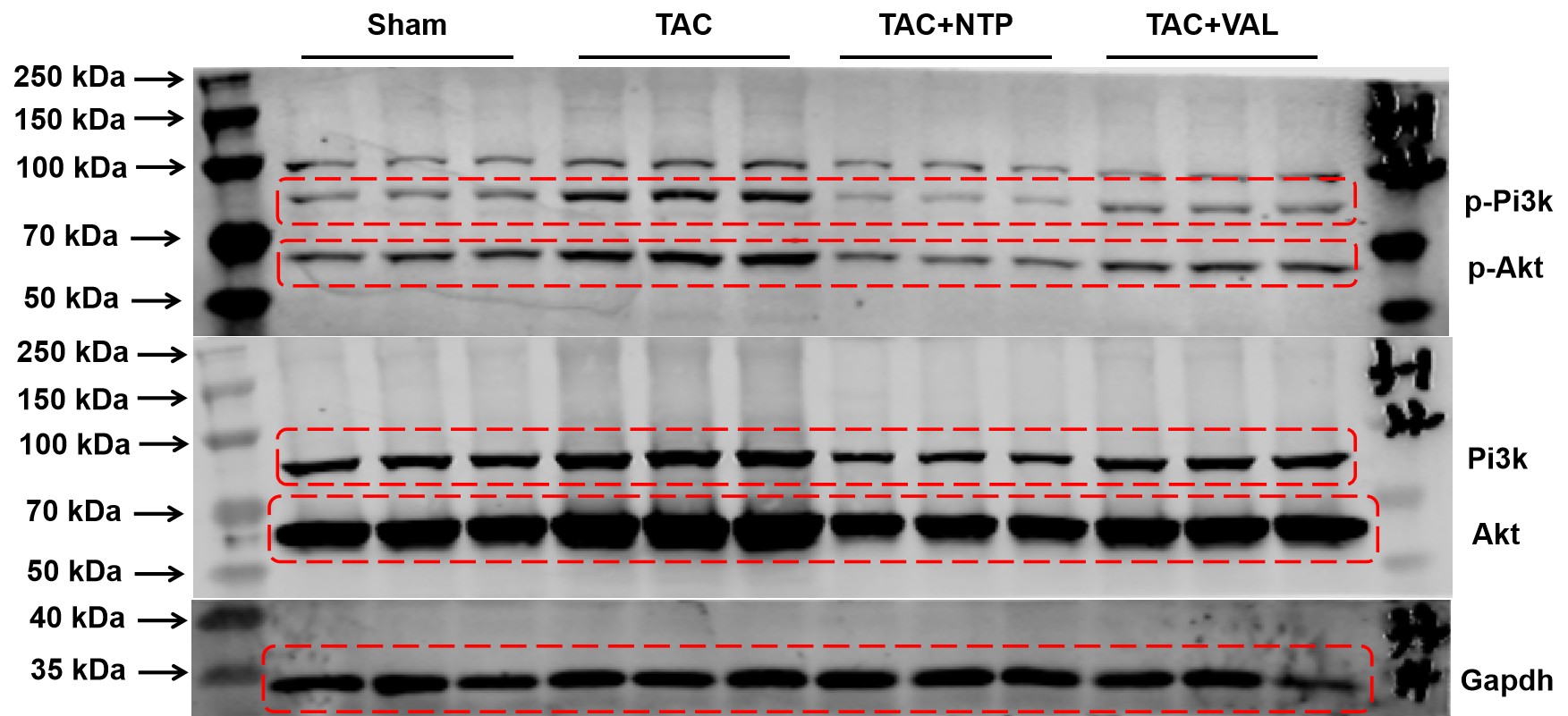

Supplement: Supplementary file 17 — Appendix Figure Source Data [file 44321_2025_334_MOESM17_ESM.zip › Original Blot Source Data/Appendix Fig. S11O/Appendix Fig. S11O.jpg]

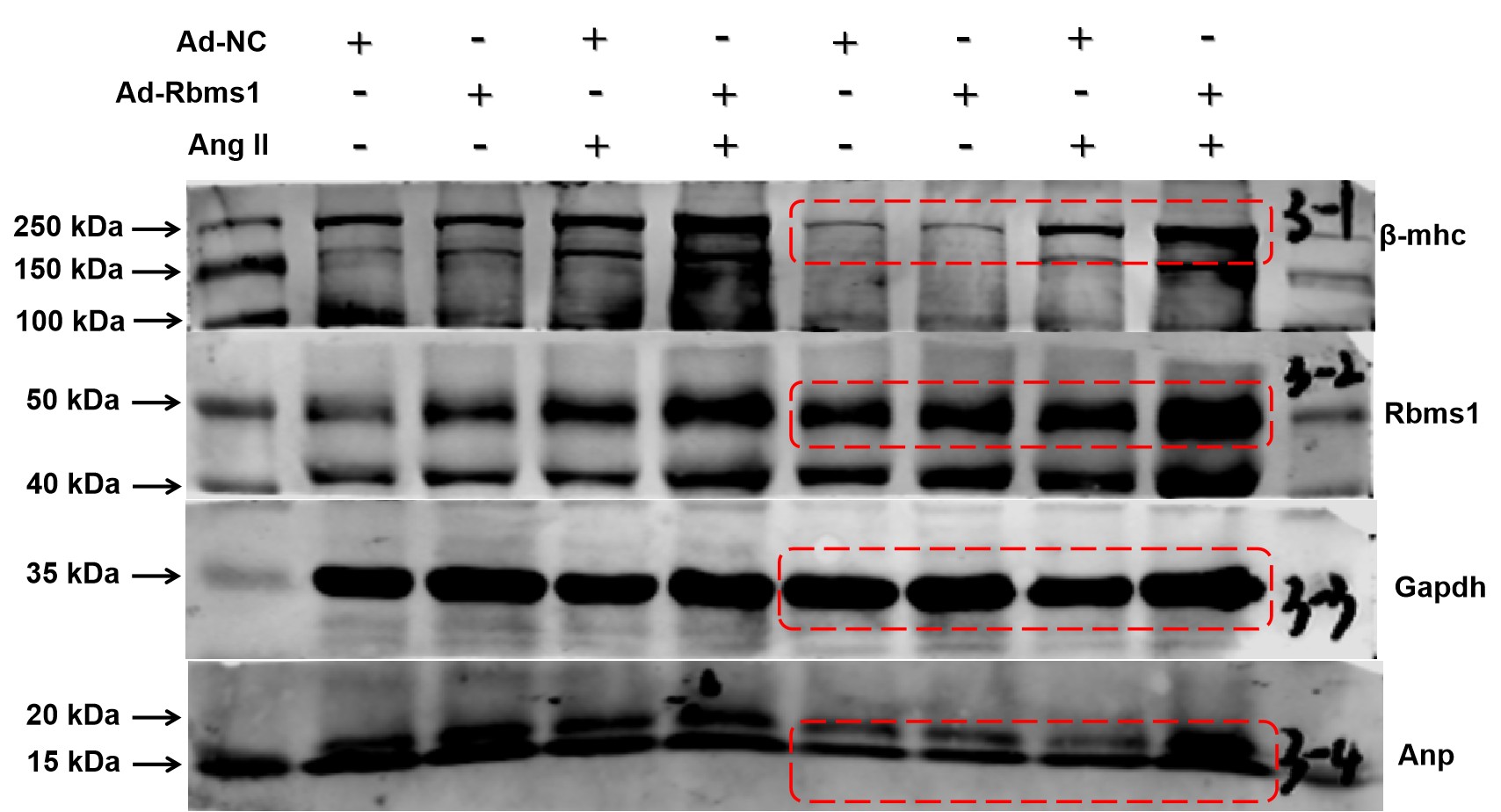

Supplement: Supplementary file 17 — Appendix Figure Source Data [file 44321_2025_334_MOESM17_ESM.zip › Original Blot Source Data/Appendix Fig. S1I/Appendix Fig. S1I.jpg]

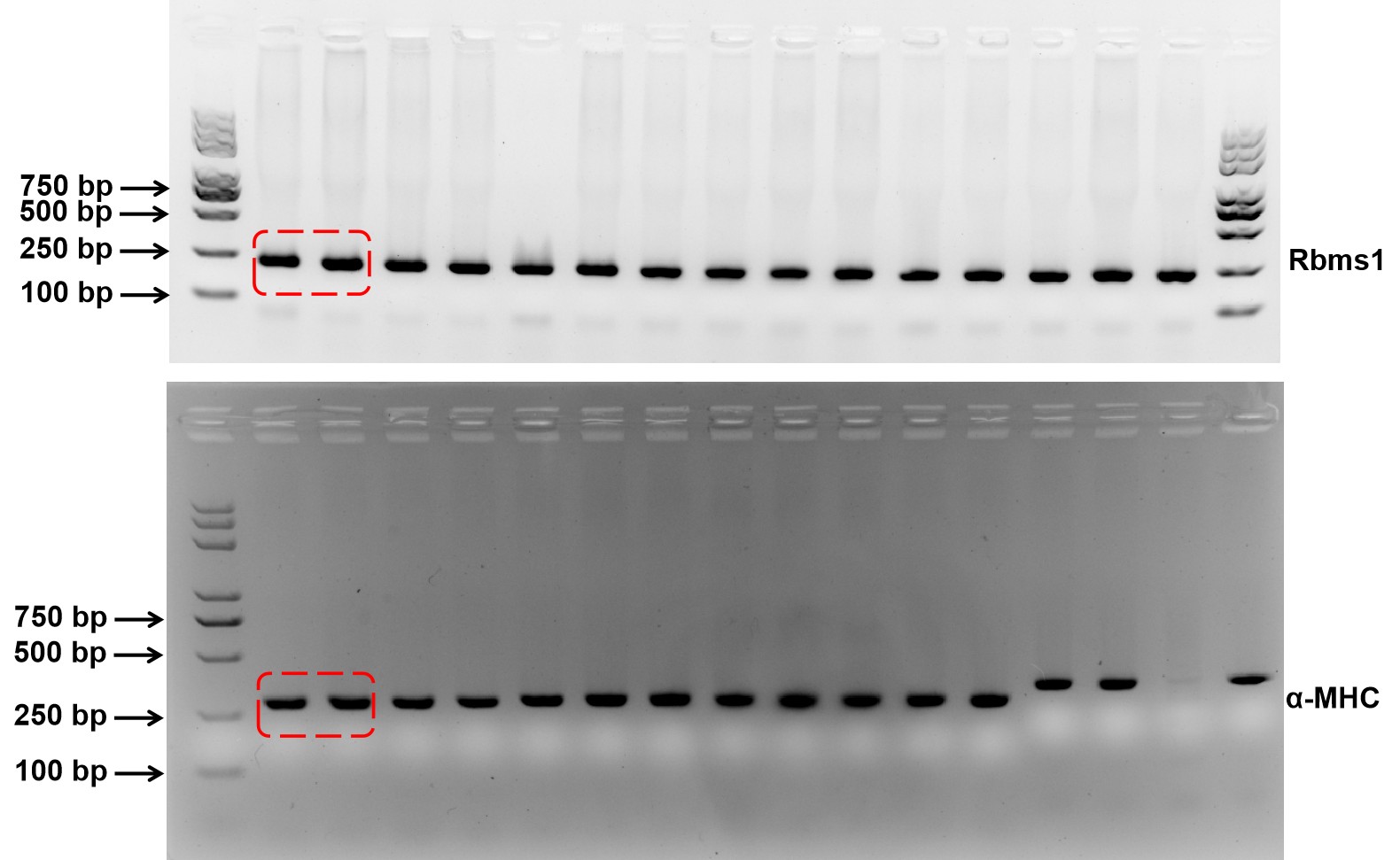

Supplement: Supplementary file 17 — Appendix Figure Source Data [file 44321_2025_334_MOESM17_ESM.zip › Original Blot Source Data/Appendix Fig. S2B/Appendix Fig. S2B.jpg]

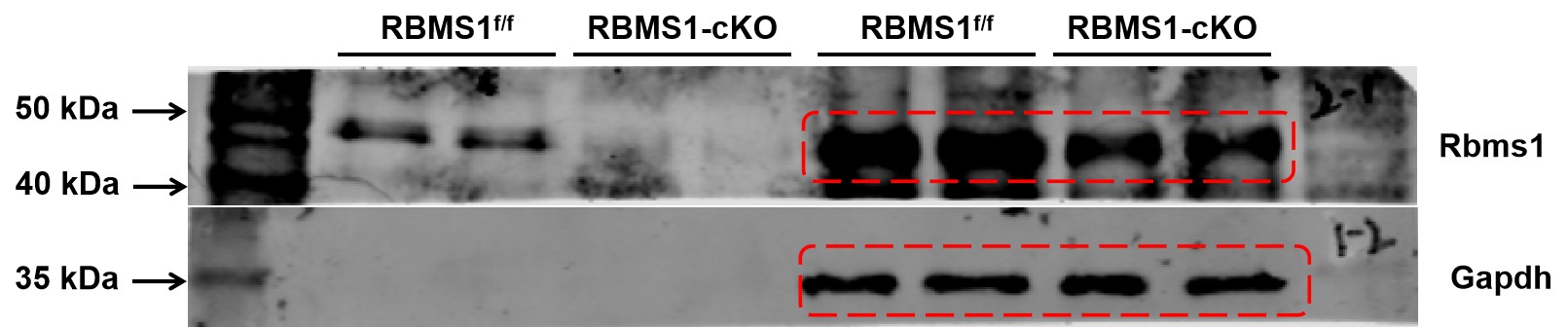

Supplement: Supplementary file 17 — Appendix Figure Source Data [file 44321_2025_334_MOESM17_ESM.zip › Original Blot Source Data/Appendix Fig. S2D/Appendix Fig. S2D.jpg]

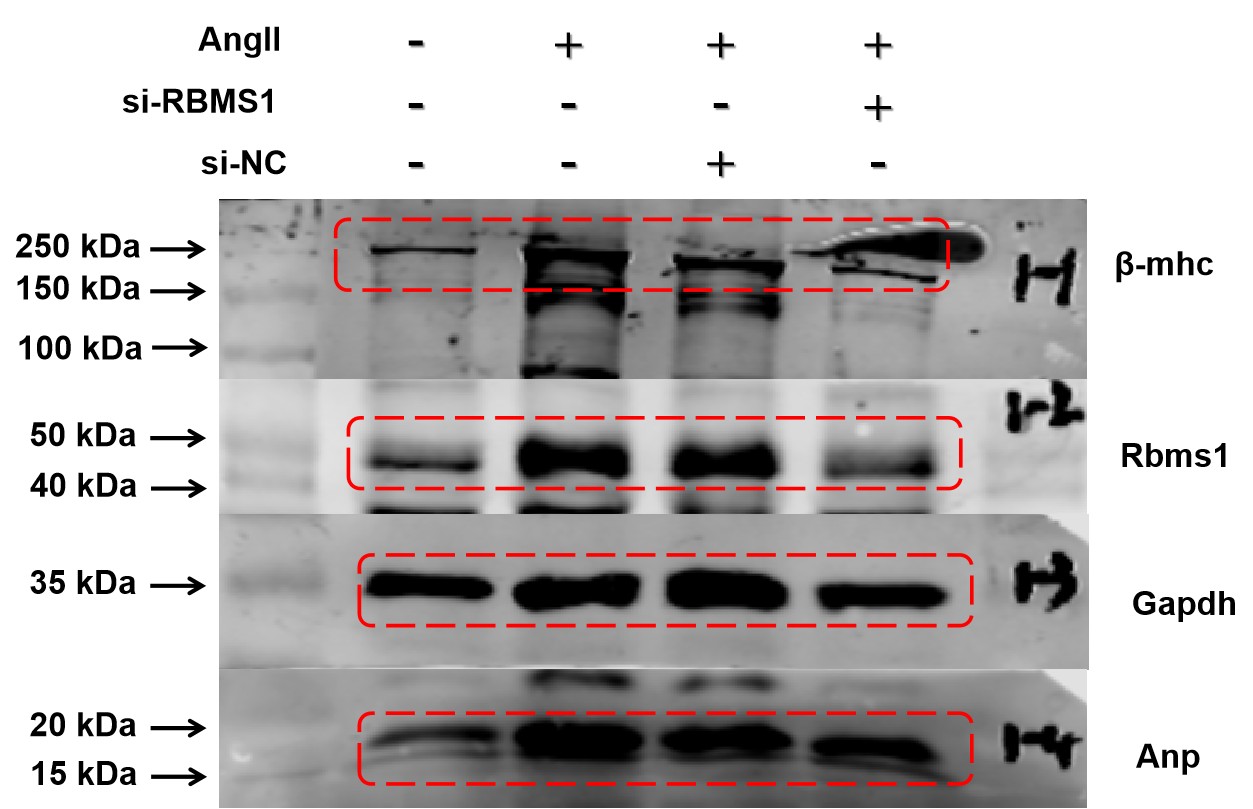

Supplement: Supplementary file 17 — Appendix Figure Source Data [file 44321_2025_334_MOESM17_ESM.zip › Original Blot Source Data/Appendix Fig. S2O/Appendix Fig. S2O.jpg]

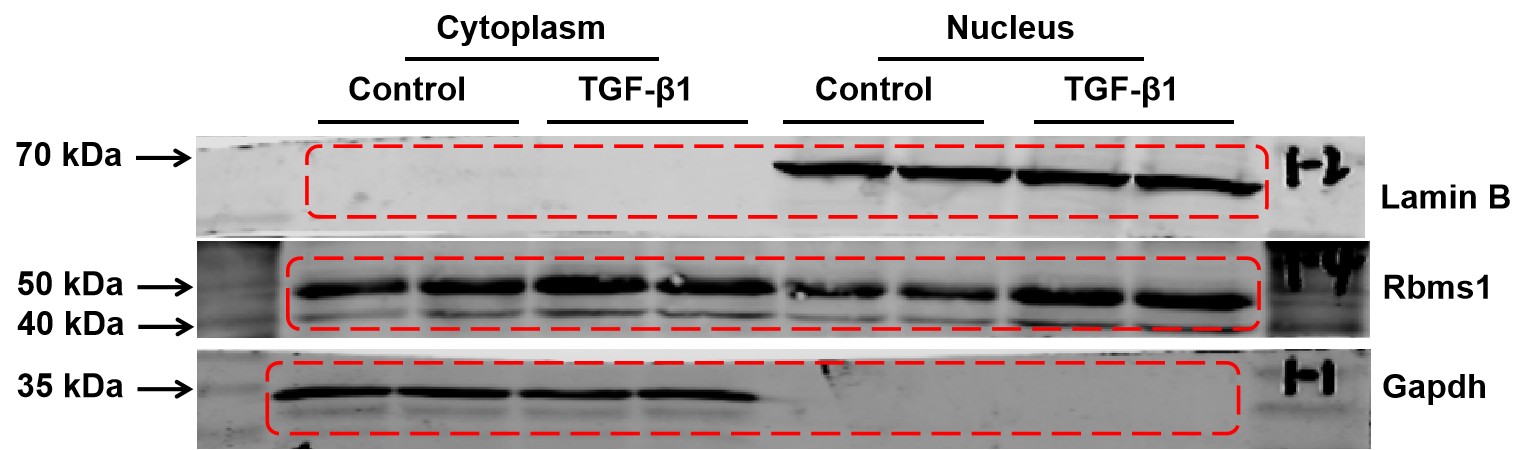

Supplement: Supplementary file 17 — Appendix Figure Source Data [file 44321_2025_334_MOESM17_ESM.zip › Original Blot Source Data/Appendix Fig. S3A/Appendix Fig. S3A.jpg]

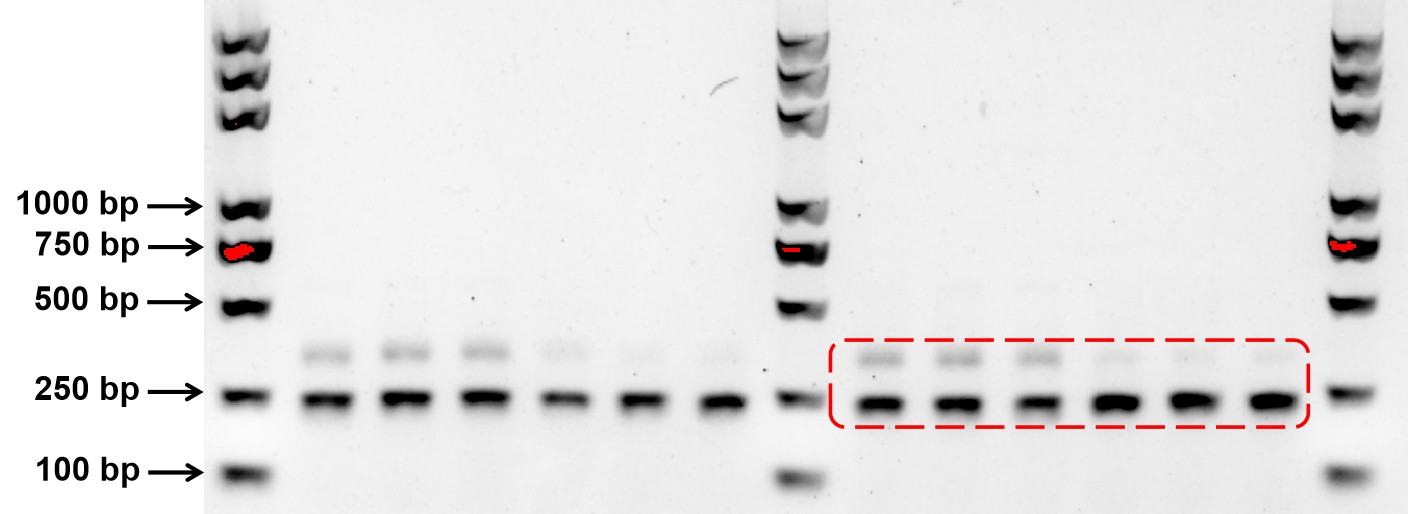

Supplement: Supplementary file 17 — Appendix Figure Source Data [file 44321_2025_334_MOESM17_ESM.zip › Original Blot Source Data/Appendix Fig. S4A/Appendix Fig. S4A.jpg]

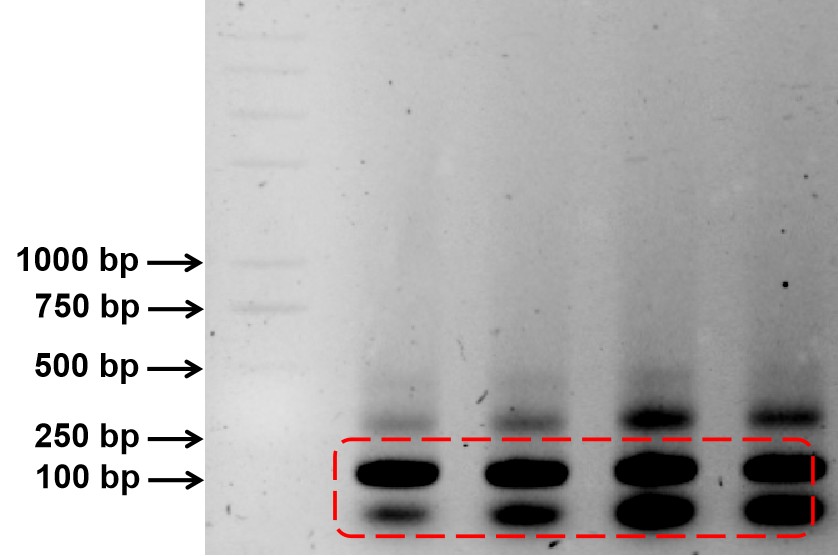

Supplement: Supplementary file 17 — Appendix Figure Source Data [file 44321_2025_334_MOESM17_ESM.zip › Original Blot Source Data/Appendix Fig. S4B/Appendix Fig. S4B.jpg]

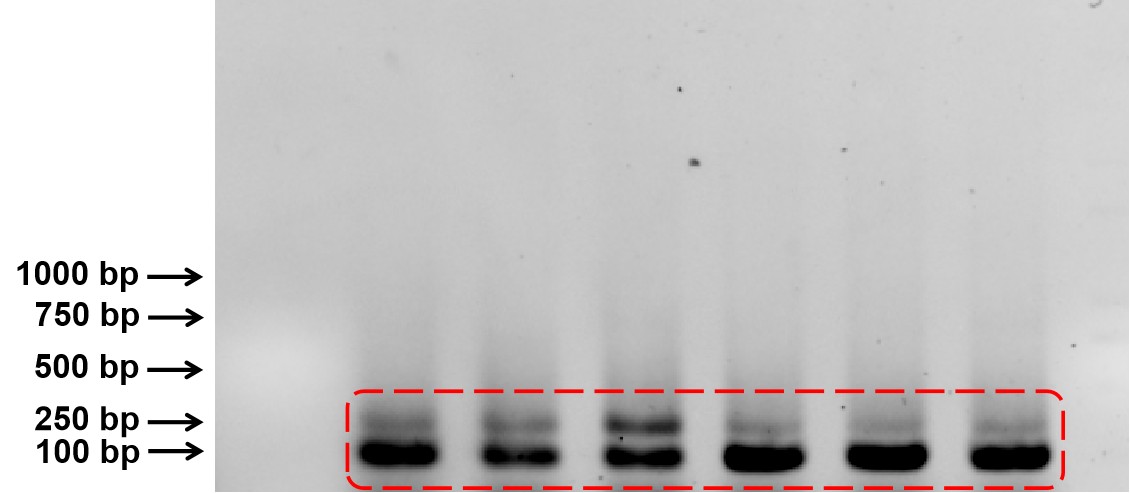

Supplement: Supplementary file 17 — Appendix Figure Source Data [file 44321_2025_334_MOESM17_ESM.zip › Original Blot Source Data/Appendix Fig. S4C/Appendix Fig. S4C.jpg]

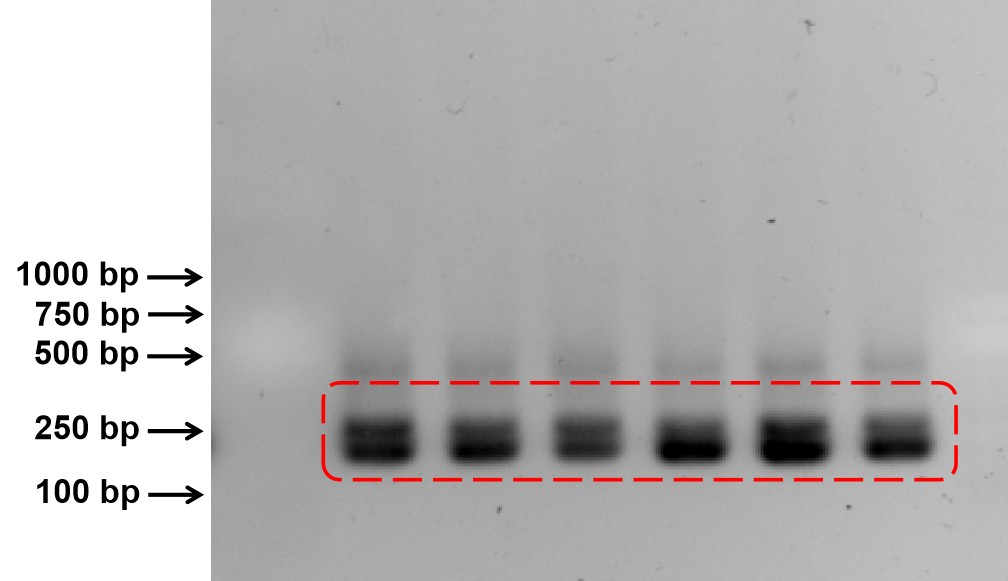

Supplement: Supplementary file 17 — Appendix Figure Source Data [file 44321_2025_334_MOESM17_ESM.zip › Original Blot Source Data/Appendix Fig. S4D/Appendix Fig. S4D.jpg]

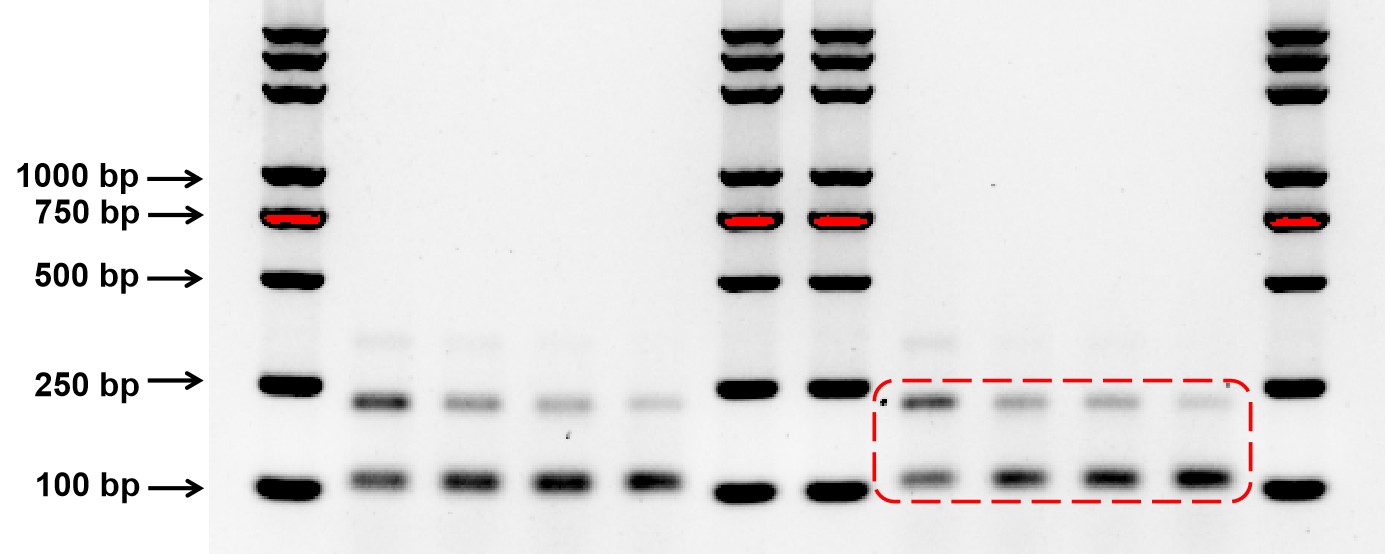

Supplement: Supplementary file 17 — Appendix Figure Source Data [file 44321_2025_334_MOESM17_ESM.zip › Original Blot Source Data/Appendix Fig. S4J/Appendix Fig. S4J.jpg]

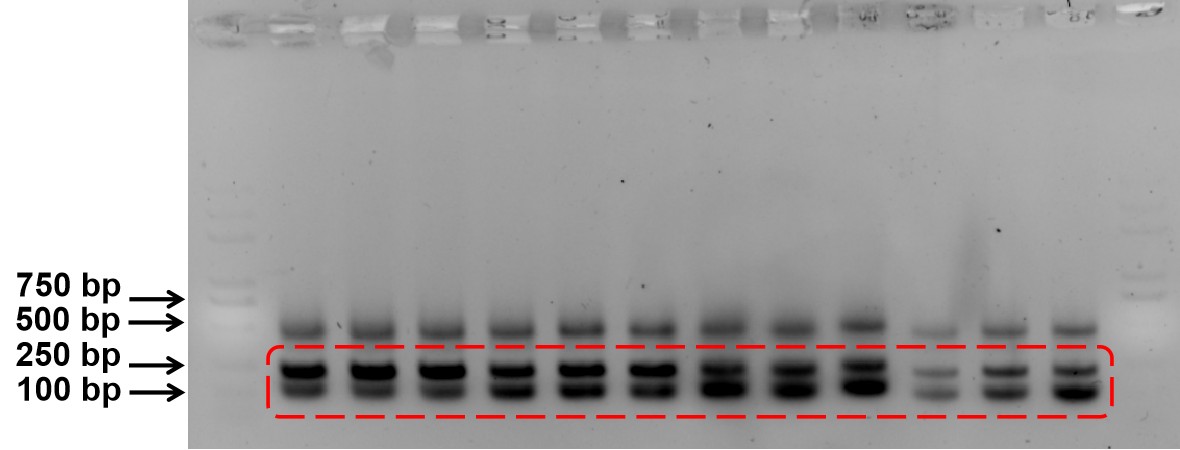

Supplement: Supplementary file 17 — Appendix Figure Source Data [file 44321_2025_334_MOESM17_ESM.zip › Original Blot Source Data/Appendix Fig. S4K/Appendix Fig. S4K.jpg]

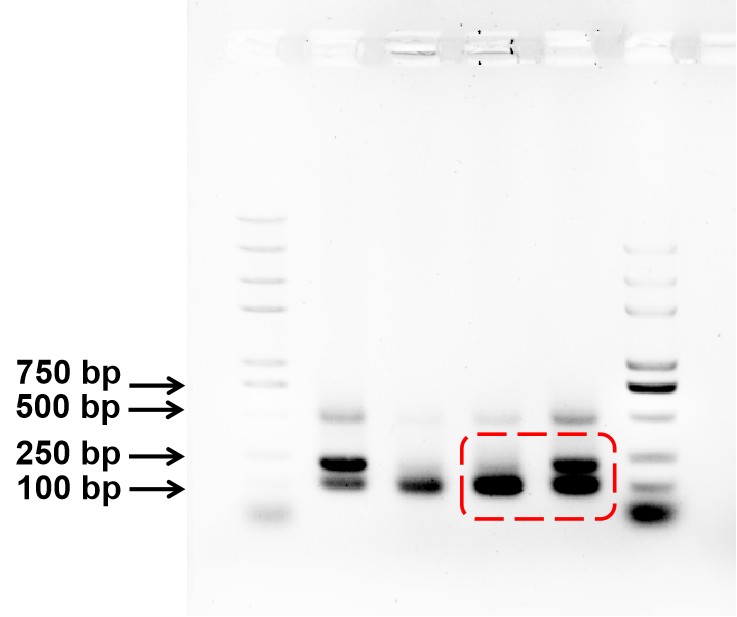

Supplement: Supplementary file 17 — Appendix Figure Source Data [file 44321_2025_334_MOESM17_ESM.zip › Original Blot Source Data/Appendix Fig. S4L/Appendix Fig. S4L.jpg]

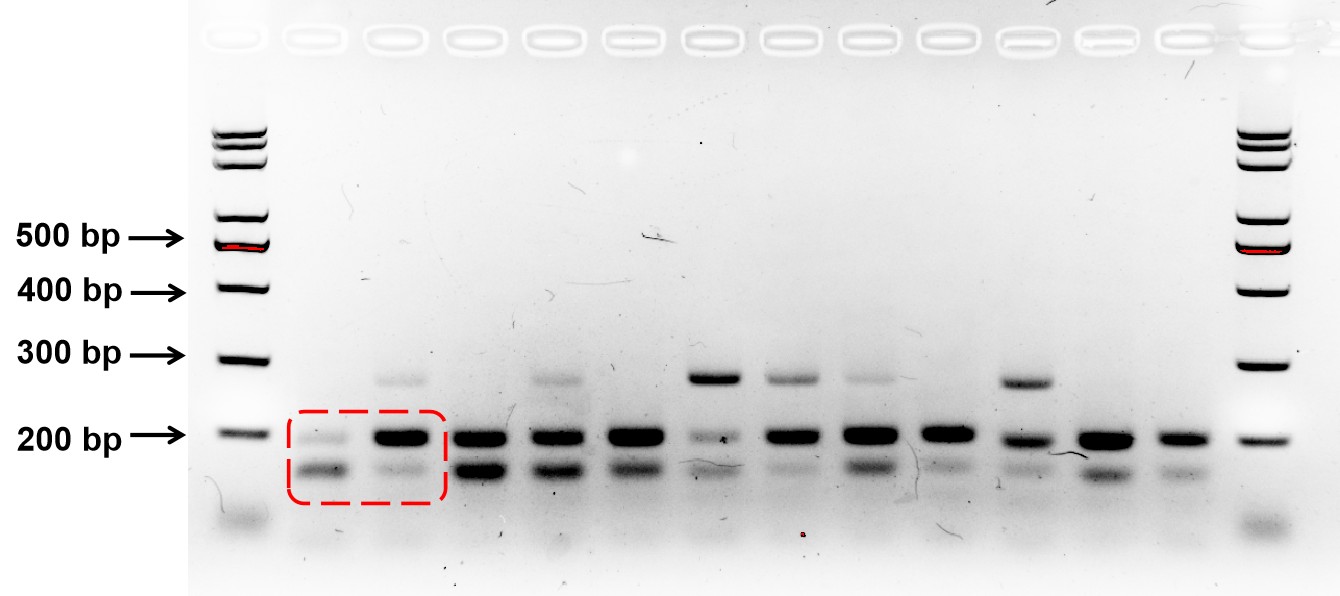

Supplement: Supplementary file 17 — Appendix Figure Source Data [file 44321_2025_334_MOESM17_ESM.zip › Original Blot Source Data/Appendix Fig. S4M/Appendix Fig. S4M.jpg]

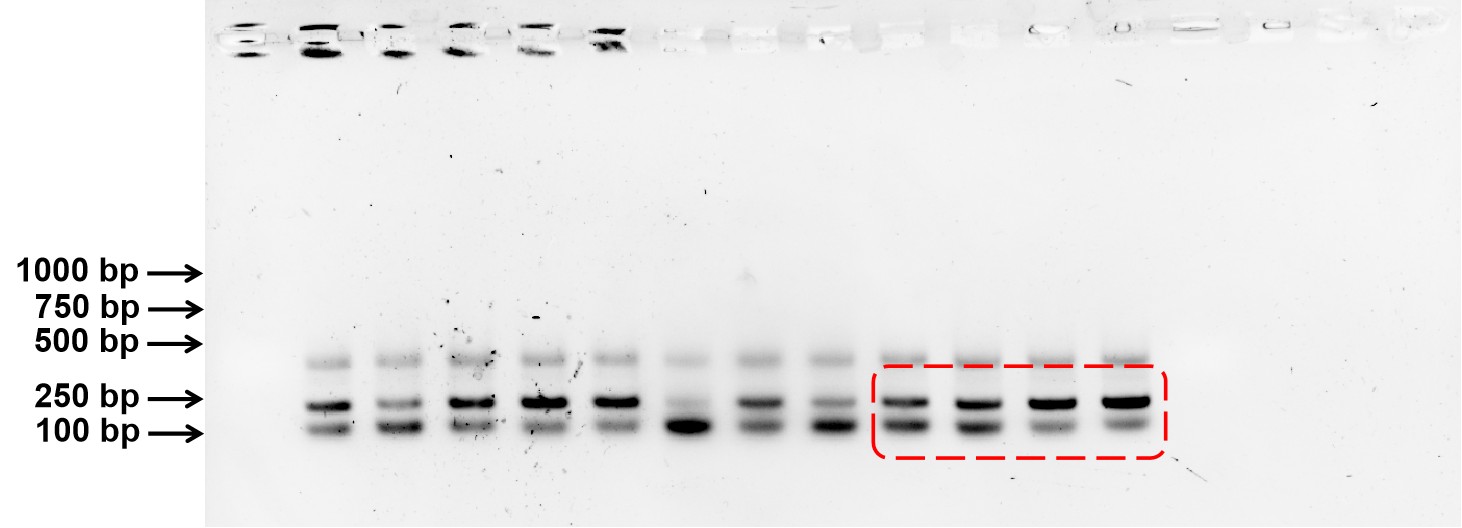

Supplement: Supplementary file 17 — Appendix Figure Source Data [file 44321_2025_334_MOESM17_ESM.zip › Original Blot Source Data/Appendix Fig. S5A/Appendix Fig. S5A.jpg]

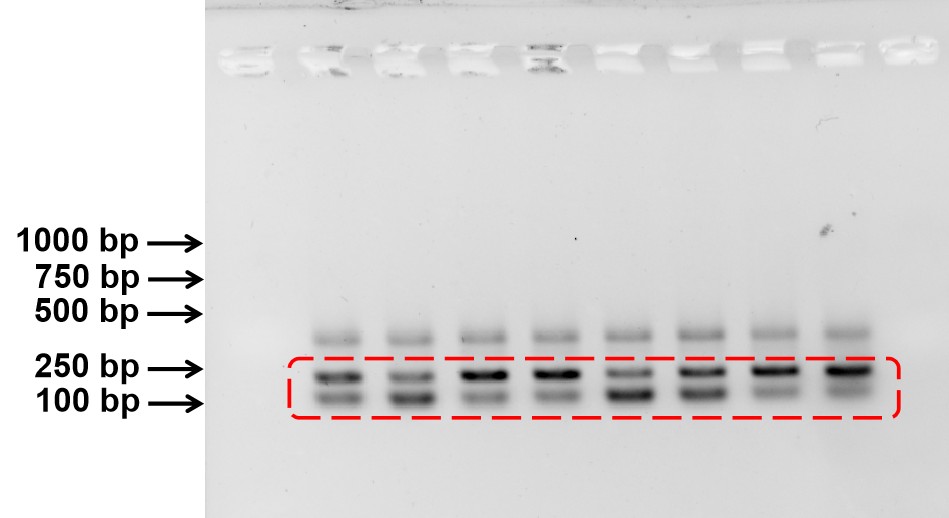

Supplement: Supplementary file 17 — Appendix Figure Source Data [file 44321_2025_334_MOESM17_ESM.zip › Original Blot Source Data/Appendix Fig. S6A/Appendix Fig. S6A.jpg]

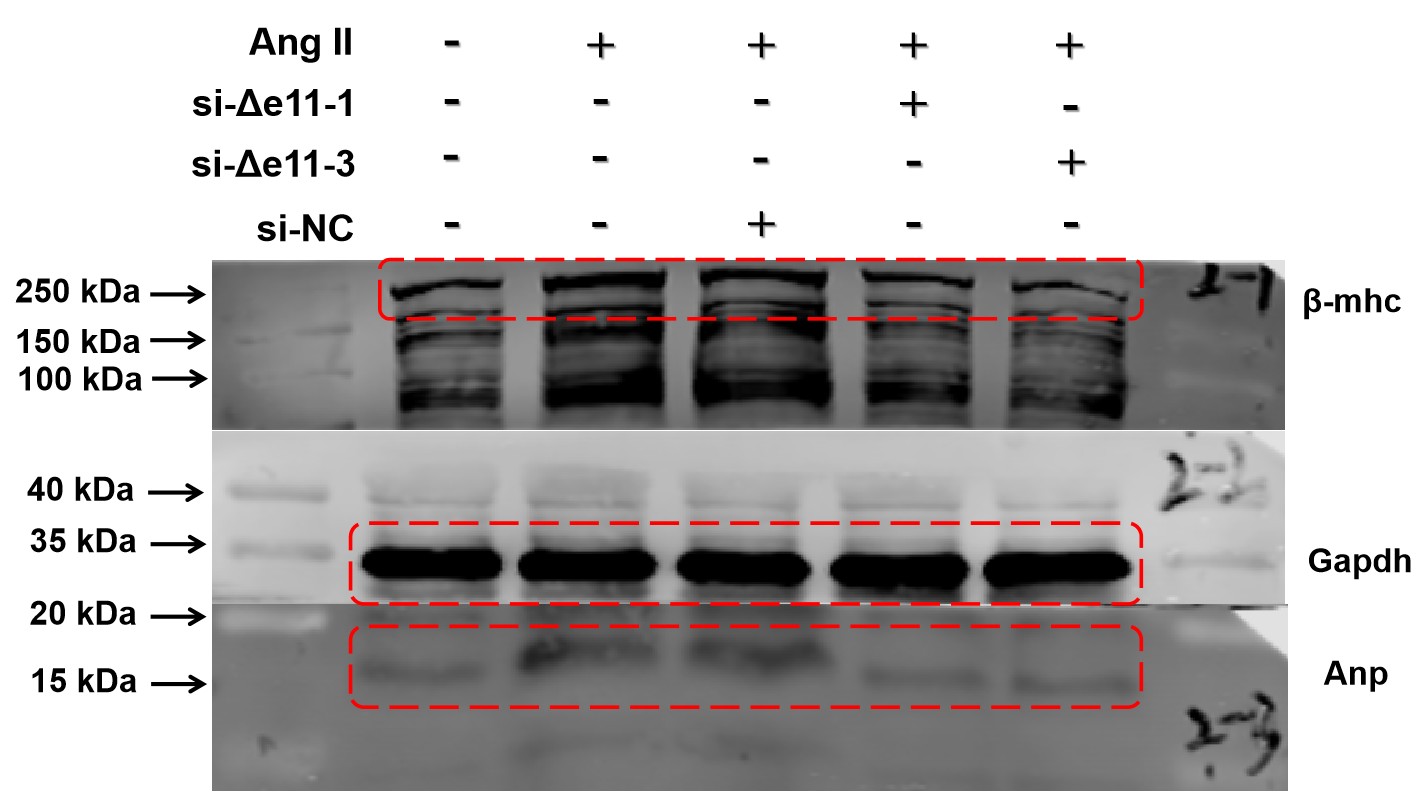

Supplement: Supplementary file 17 — Appendix Figure Source Data [file 44321_2025_334_MOESM17_ESM.zip › Original Blot Source Data/Appendix Fig. S6B/Appendix Fig. S6B.jpg]

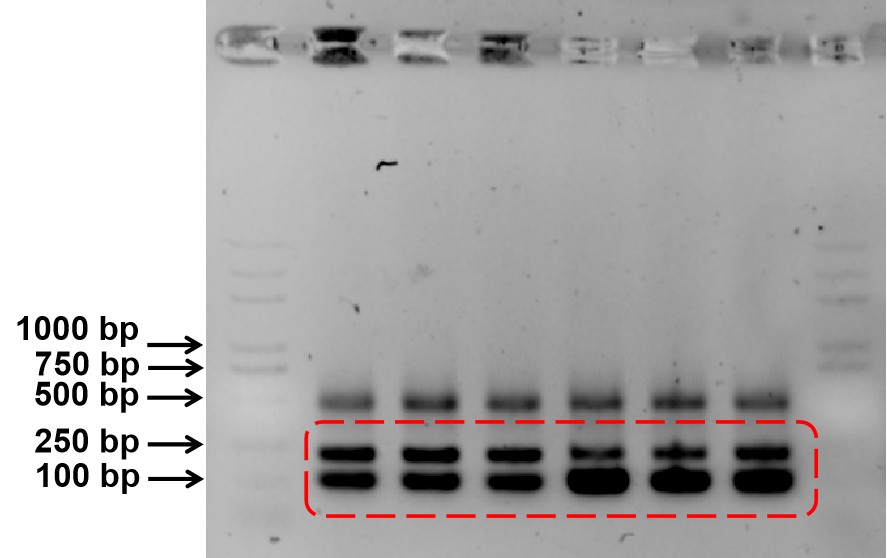

Supplement: Supplementary file 17 — Appendix Figure Source Data [file 44321_2025_334_MOESM17_ESM.zip › Original Blot Source Data/Appendix Fig. S7A/Appendix Fig. S7A.jpg]

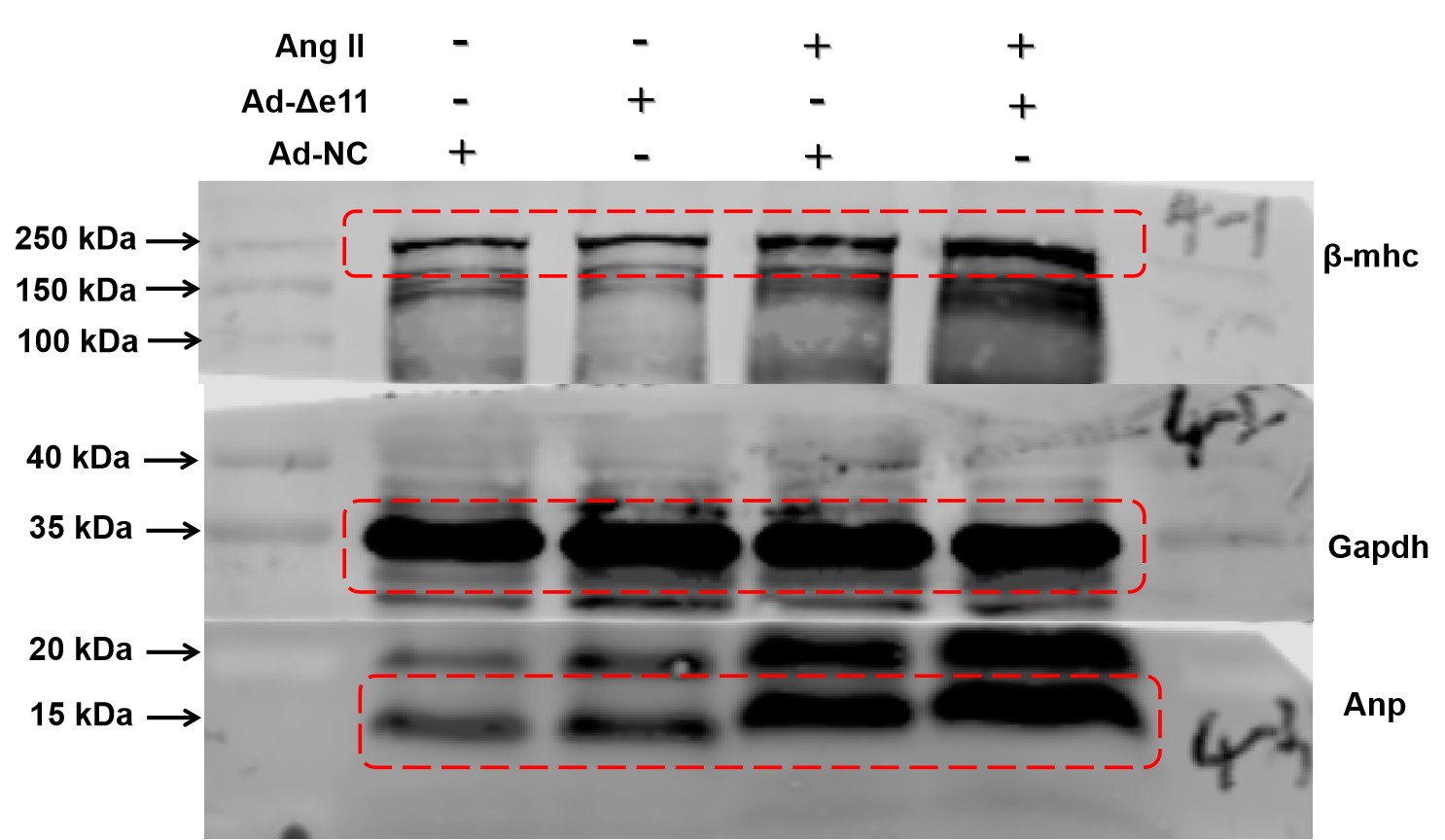

Supplement: Supplementary file 17 — Appendix Figure Source Data [file 44321_2025_334_MOESM17_ESM.zip › Original Blot Source Data/Appendix Fig. S7B/Appendix Fig. S7B.jpg]

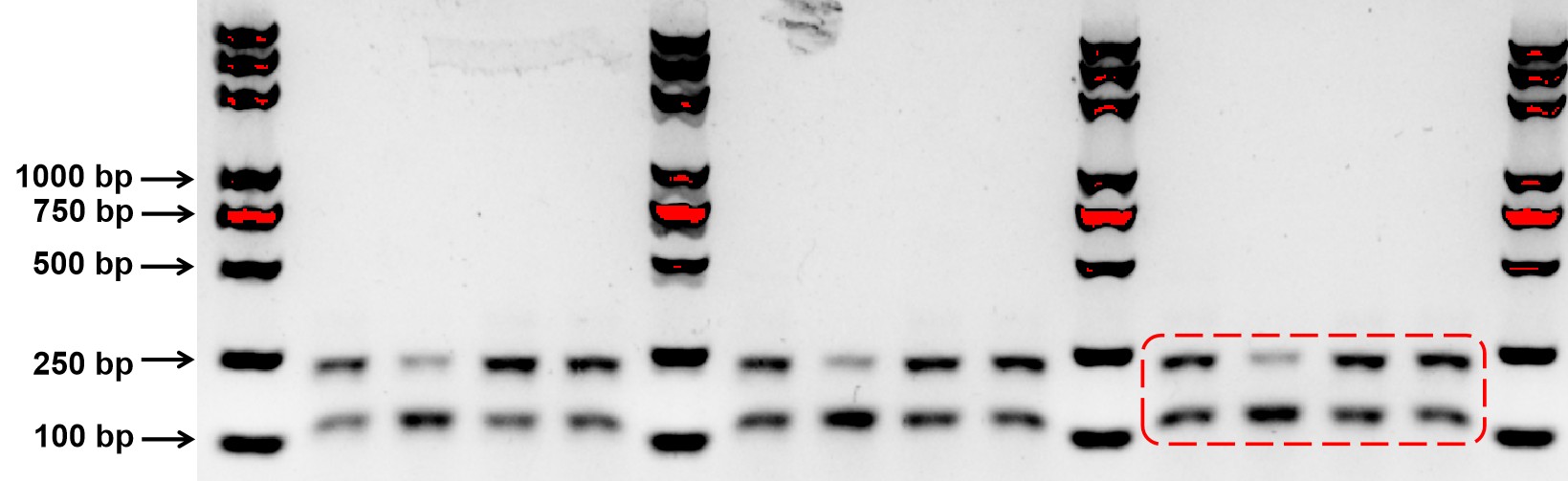

Supplement: Supplementary file 17 — Appendix Figure Source Data [file 44321_2025_334_MOESM17_ESM.zip › Original Blot Source Data/Appendix Fig. S8N/Appendix Fig. S8N.jpg]

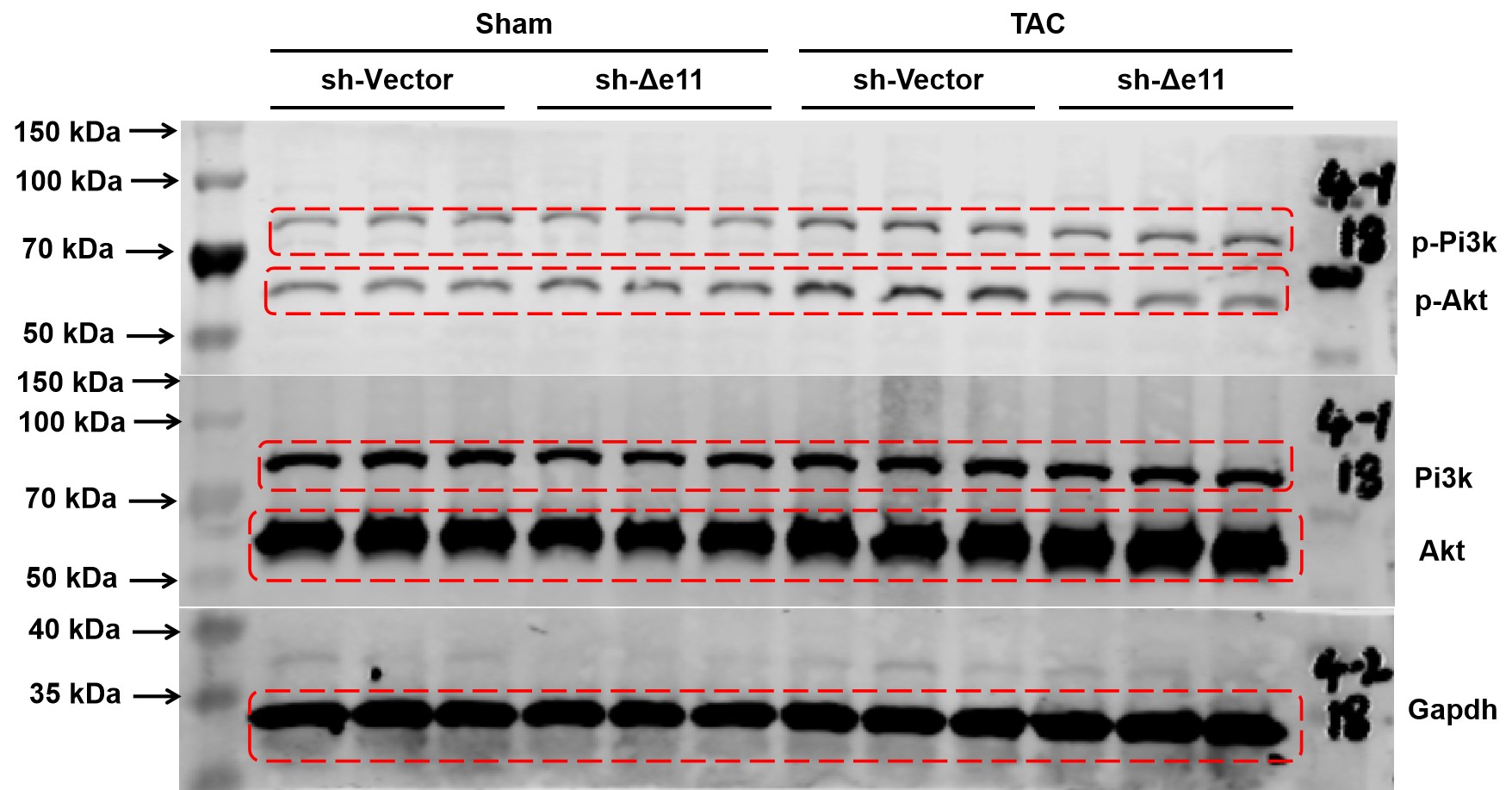

Supplement: Supplementary file 17 — Appendix Figure Source Data [file 44321_2025_334_MOESM17_ESM.zip › Original Blot Source Data/Appendix Fig. S9E/Appendix Fig. S9E.jpg]

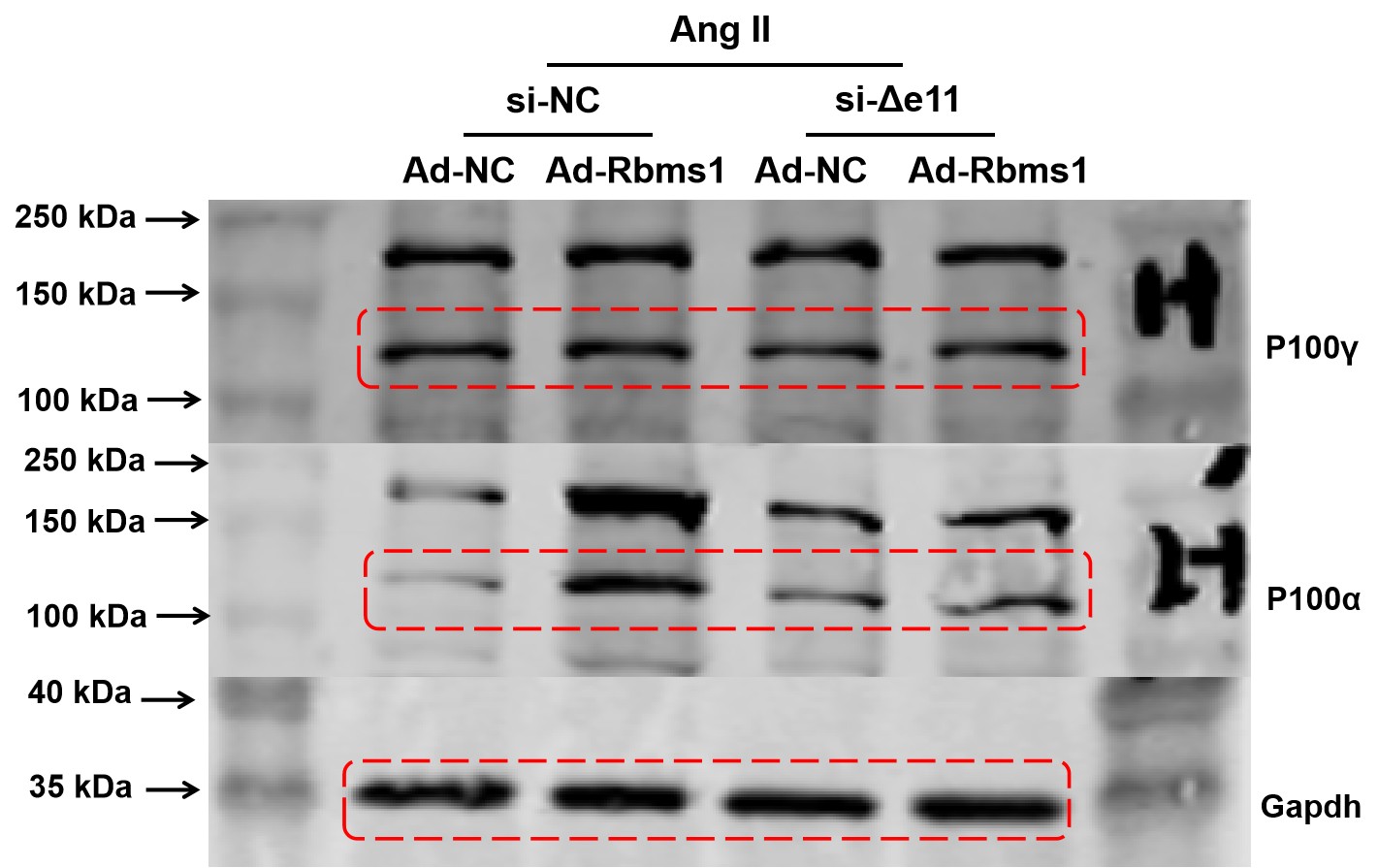

Supplement: Supplementary file 17 — Appendix Figure Source Data [file 44321_2025_334_MOESM17_ESM.zip › Original Blot Source Data/Appendix Fig. S9F/Appendix Fig. S9F.jpg]
