# Supplementary material for: RBMS1 orchestrates cardiac hypertrophy by facilitating CTTN splice-switching and sarcomere dynamics
Source: EMBO Mol Med. 2025 Nov 10;17(12):3555–85. doi: 10.1038/s44321-025-00334-z (PMC12686484; doi:10.1038/s44321-025-00334-z)
Supplement: Supplementary file 18 — Expanded View Figures [file 44321_2025_334_MOESM18_ESM.pdf]

Expanded View Figures

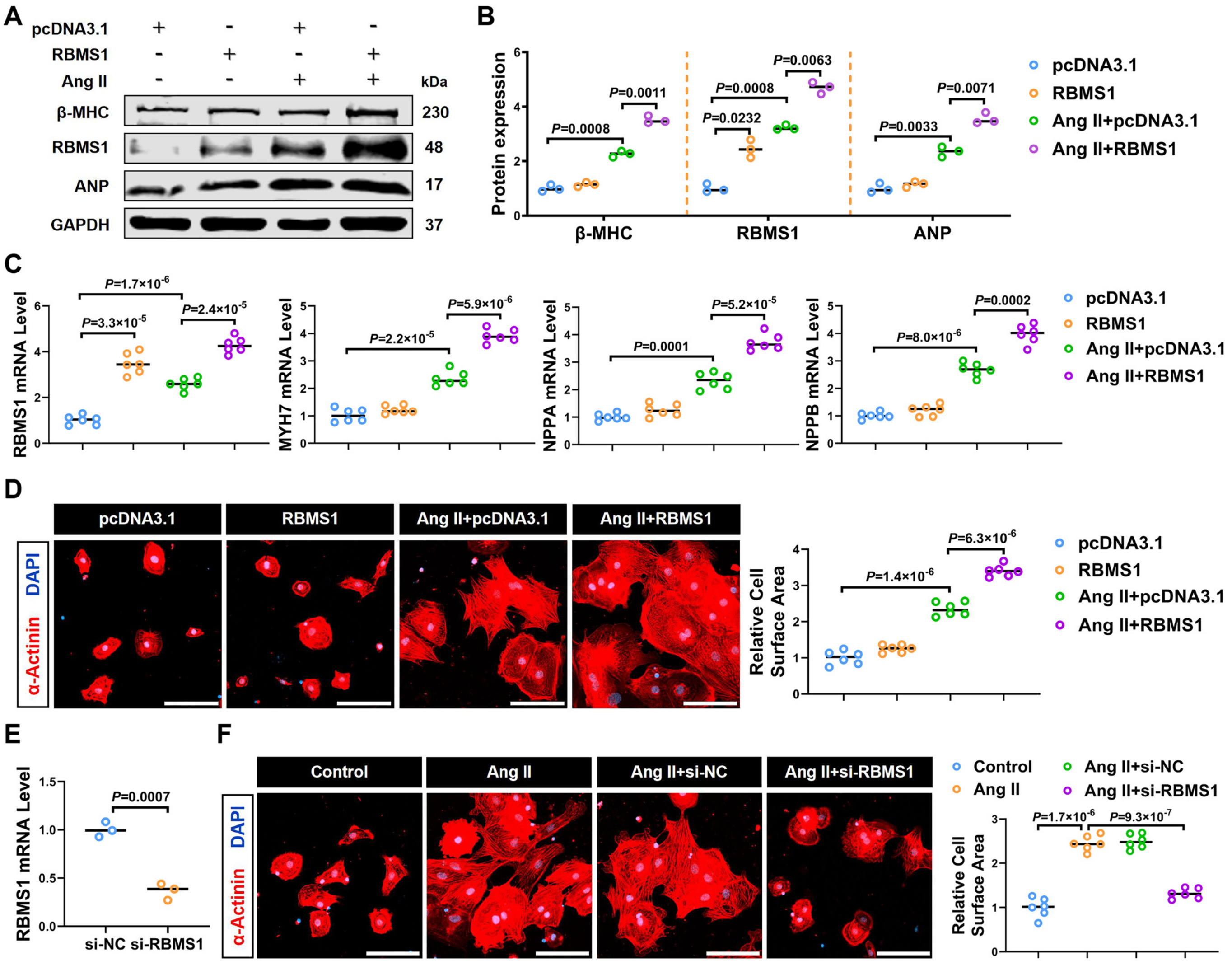

Figure EV1. RBMS1 facilitates cardiac hypertrophy induced by Ang II in hiPSC-CMs.

(**A**, **B**) Western blotting and quantification showing protein levels of β-MHC, RBMS1, and ANP in hiPSC-CMs transfected with RBMS1 in response to Ang II stimulation ($n = 3$). (**C**) Quantification of mRNA levels of RBMS1, MYH7, NPPA, and NPPB ($n = 6$). (**D**) Representative immunofluorescence staining of α-actinin and quantification in hiPSC-CMs transfected with RBMS1 ($n = 6$). Scale bar = 50 μm. (**E**) Quantification of mRNA levels of RBMS1 in hiPSC-CMs transfected with si-NC or si-RBMS1 ($n = 3$). (**F**) Representative immunofluorescence staining of α-actinin and quantification in hiPSC-CMs transfected with si-RBMS1 ($n = 6$). Scale bar = 50 μm. Data information: data are shown as mean ± SEM, *P* values were analyzed with unpaired Student's t test (**E**) and one-way ANOVA test (**B**, **C**, **D**, **F**). A dot represents an independent biological sample. Source data are available online for this figure.

 © The Author(s)

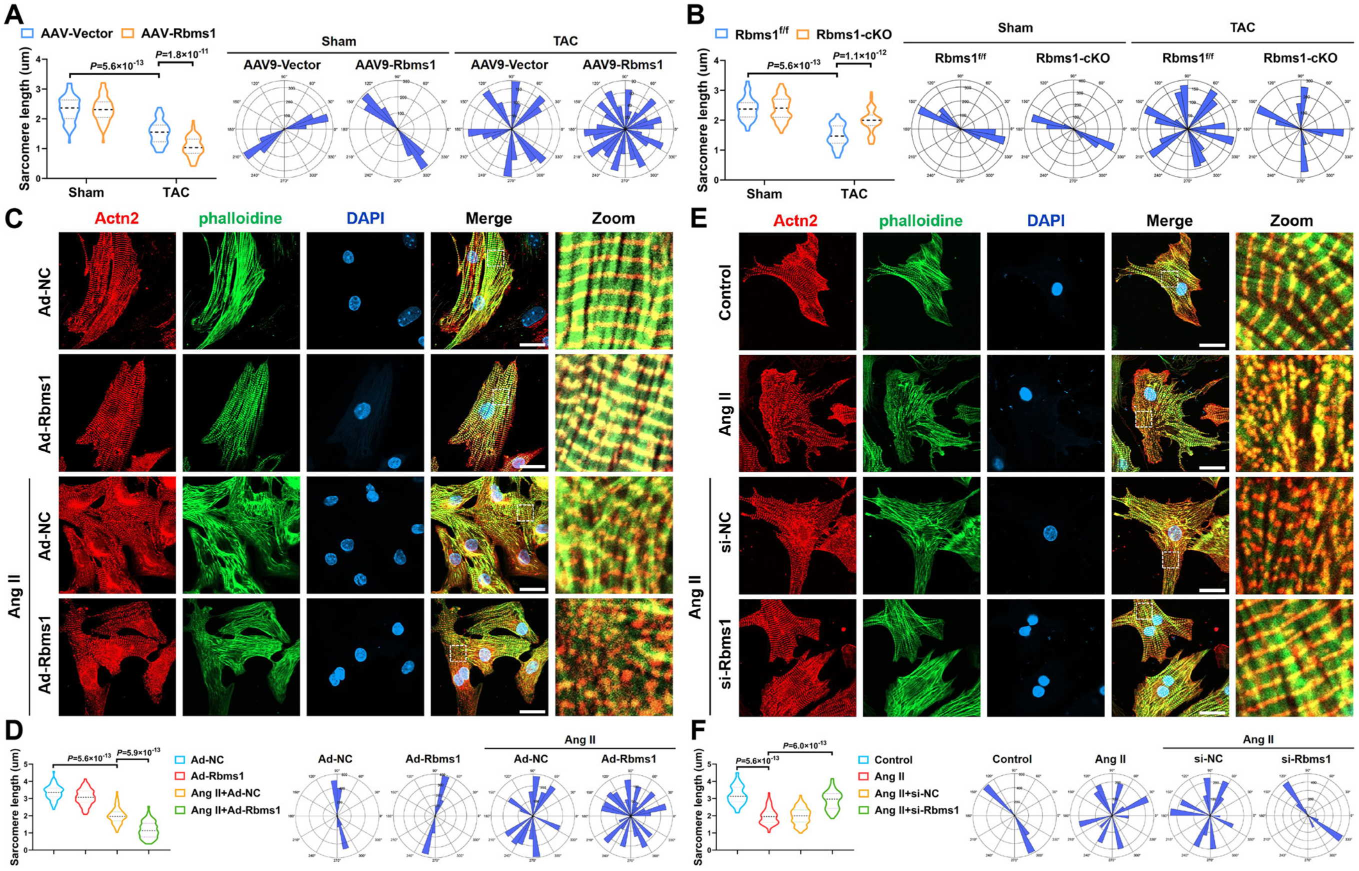

Figure EV2. RBMS1 regulates cardiac hypertrophy via the sarcomere and cytoskeleton of cardiomyocytes.

(**A**) Quantification of sarcomere length and representative polarity histogram of sarcomere organization in RBMS1 overexpression mice treatment with TAC surgery ($n = 70$). (**B**) Quantification of sarcomere length and representative polarity histogram of sarcomere organization in RBMS1-cKO mice treatment with TAC surgery ($n = 70$). (**C**, **D**) Quantification of sarcomere length and representative polarity histogram of sarcomere organization in NMCMs transfected with Ad-RBMS1 in response to Ang II stimulation ($n = 70$). (**E**, **F**) Quantification of sarcomere length and representative polarity histogram of sarcomere organization in NMCMs transfected with si-RBMS1 and subsequently treated with Ang II ($n = 70$). Data information: data are shown as mean ± SEM, *P* values were analyzed with one-way ANOVA test (**A**, **B**, **D**, **F**), polarity histograms were generated by MATLAB R2024b (**A**, **B**, **D**, **F**). Source data are available online for this figure.

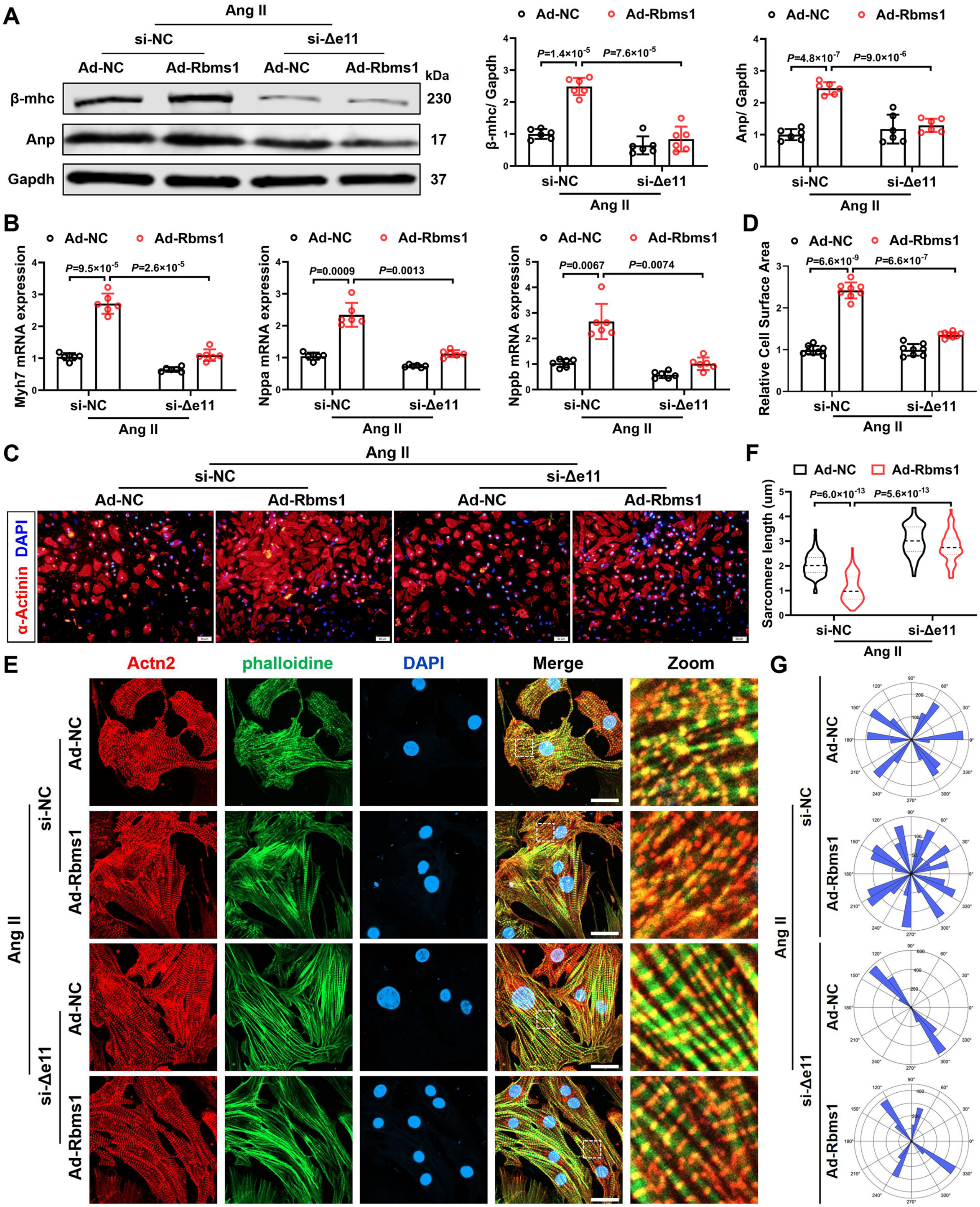
A
Ang II
si-NC
si-Δe11
Ad-NC
Ad-Rbms1
kDa
β-mhc
230
Anp
17
Gapdh
37
β-mhc/ Gapdh
Anp/ Gapdh
B
Myh7 mRNA expression
Nppa mRNA expression
Nppb mRNA expression
D
Relative Cell Surface Area
C
α-Actinin DAPI
F
Sarcomere length (um)
E
Actn2
phalloidine
DAPI
Merge
Zoom
G

Figure EV3. The pro-hypertrophic function of RBMS1 depends on splicing CTTN.

(**A**) Western blotting and quantification showing protein levels of β-MHC and ANP in RBMS1 overexpression NMCMs transfected with si-Δe11 in response to Ang II stimulation ($n = 6$). (**B**) Quantification of mRNA levels of MYH7, NPPA, and NPPB ($n = 6$). (**C**, **D**) Representative immunofluorescence staining of α-actinin and quantification of cell surface area ($n = 8$), scale bar = 50 μm. (**E**) Immunofluorescence staining of ACTN2 and phalloidine showed the disorganization of sarcomere and cytoskeleton in NMCMs, scale bar = 20 μm. (**F**, **G**) Quantification of sarcomere length and representative polarity histogram of sarcomere organization in NMCMs ($n = 70$). Data information: data are shown as mean ± SEM, P values were analyzed with one-way ANOVA test (**A**, **B**, **D**, **F**), polarity histograms were generated by MATLAB R2024b (**G**). Source data are available online for this figure.

© The Author(s)

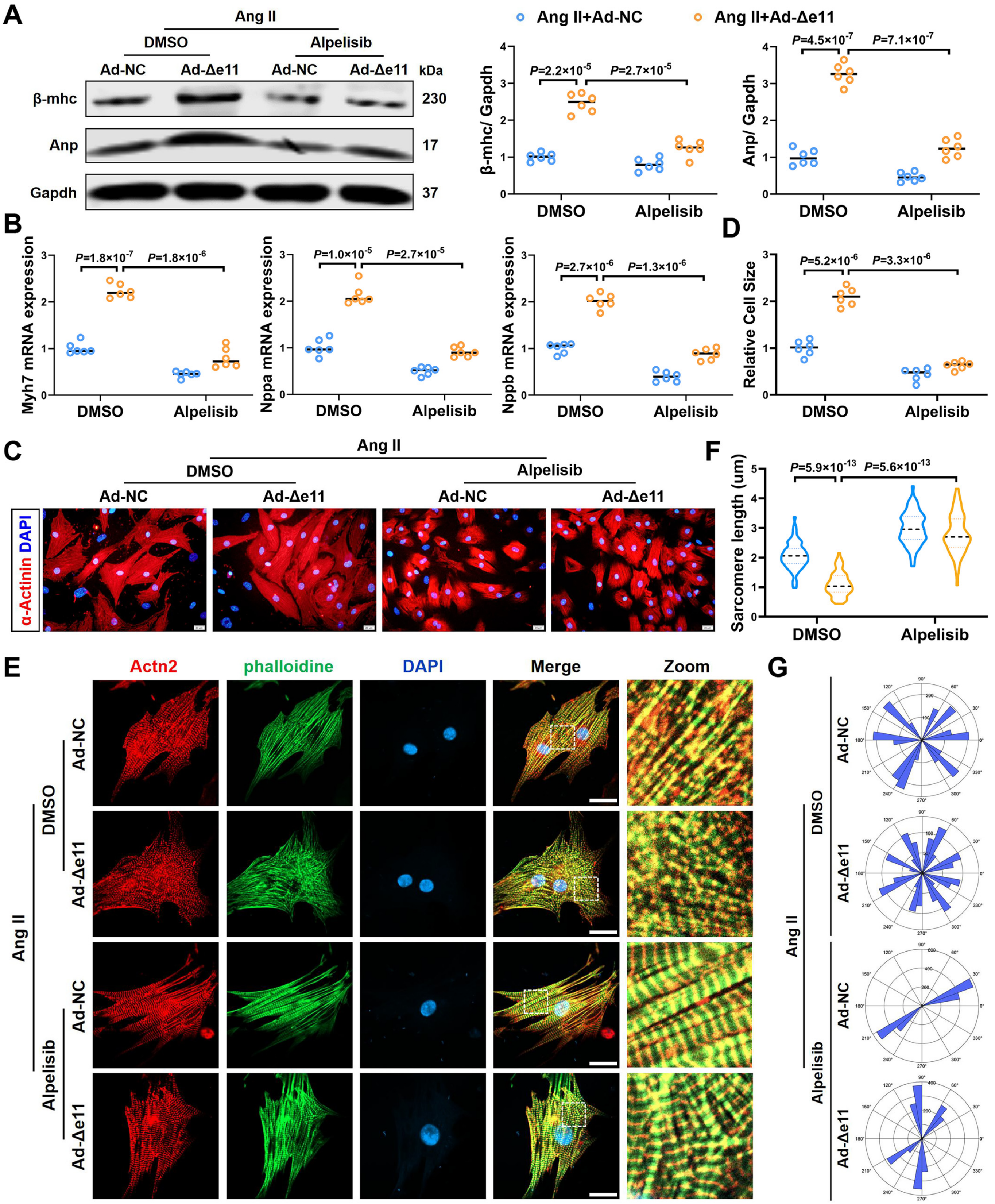
A
Ang II
DMSO
Alpelisib
Ad-NC
Ad-Δe11
kDa
β-mhc
230
Anp
17
Gapdh
37
Ang II+Ad-NC
Ang II+Ad-Δe11
P=2.2×10-5
P=2.7×10-5
β-mhc/ Gapdh
P=4.5×10-7
P=7.1×10-7
Anp/ Gapdh
DMSO
Alpelisib
B
P=1.8×10-7
P=1.8×10-6
Myh7 mRNA expression
P=1.0×10-5
P=2.7×10-5
Nppa mRNA expression
P=2.7×10-6
P=1.3×10-6
Nppb mRNA expression
D
P=5.2×10-6
P=3.3×10-6
Relative Cell Size
C
α-Actinin DAPI
F
P=5.9×10-13
P=5.6×10-13
Sarcomere length (um)
E
Actn2
phalloidine
DAPI
Merge
Zoom
G

Figure EV4. Alpelisib mitigates cardiac hypertrophy induced by CTTN-Δe11.

(**A**) Western blotting and quantification showing protein levels of β-MHC and ANP in CTTN-Δe11 overexpression NMCMs treatment with Alpelisib in response to Ang II stimulation ($n = 6$). (**B**) Quantification of mRNA levels of β-MHC, ANP, and BNP ($n = 6$). (**C**, **D**) Representative immunofluorescence staining of α-actinin and quantification of cell surface area ($n = 6$), scale bar = 50 μm. (**E**) Immunofluorescence staining of ACTN2 and phalloidine showed the disorganization of sarcomere and cytoskeleton, scale bar = 20 μm. (**F**, **G**) Quantification of sarcomere length and representative polarity histogram of sarcomere organization in NMCMs ($n = 70$). Data information: data are shown as mean ± SEM, P values were analyzed with one-way ANOVA test (**A**, **B**, **D**, **F**), polarity histograms were generated by MATLAB R2024b (**G**). Source data are available online for this figure.

© The Author(s)
